# ‘Chemistry at the speed of sound’: automated 1536-well nanoscale synthesis of 16 scaffolds in parallel†

**DOI:** 10.1039/d2gc04312b

**Published:** 2023-01-17

**Authors:** Li Gao, Shabnam Shaabani, Atilio Reyes Romero, Ruixue Xu, Maryam Ahmadianmoghaddam, Alexander Dömling

**Affiliations:** a Department of Drug Design, University of Groningen Groningen The Netherlands; b CATRIN, Department of Innovative Chemistry, Palacký University Olomouc Olomouc Czech Republic alexander.domling@upol.cz

## Abstract

Screening of large and diverse libraries is the ‘bread and butter’ in the first phase of the discovery of novel drugs. However, maintenance and periodic renewal of high-quality large compound collections pose considerable logistic, environmental and monetary problems. Here, we exercise an alternative, the ‘on-the-fly’ synthesis of large and diverse libraries on a nanoscale in a highly automated fashion. For the first time, we show the feasibility of the synthesis of a large library based on 16 different chemistries in parallel on several 384-well plates using the acoustic dispensing ejection (ADE) technology platform. In contrast to combinatorial chemistry, we produced 16 scaffolds at the same time and in a sparse matrix fashion, and each compound was produced by a random combination of diverse large building blocks. The synthesis, analytics, resynthesis of selected compounds, and chemoinformatic analysis of the library are described. The advantages of the herein described automated nanoscale synthesis approach include great library diversity, absence of library storage logistics, superior economics, speed of synthesis by automation, increased safety, and hence sustainable chemistry.

## Abstract 2

Sustainable synthesis of highly diverse screening libraries of small molecules.

## Graphical abstract

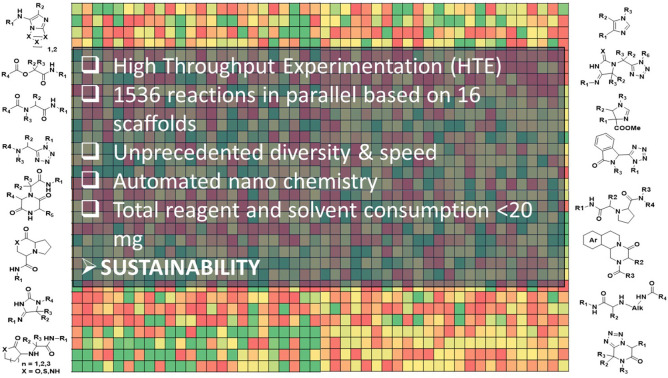

## Introduction

**Fig. 1:**
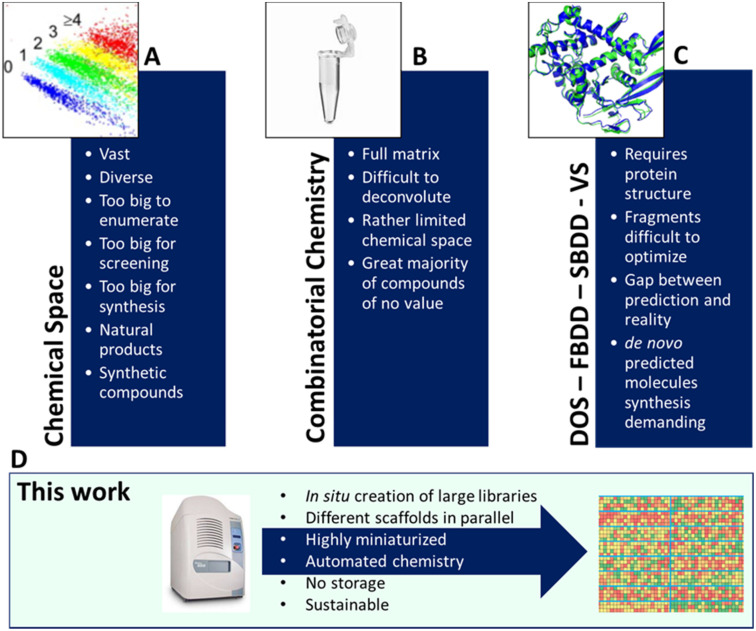
Chemical space and contemporary library synthesis strategies. (A) The vastness of chemical space makes systematic exploitation very demanding. (B) Combinatorial chemistry and DNA-encoded library (DEL). (C) Fragment-based drug design (FBDD), structure-based drug design (SBDD), diversity-oriented synthesis (DOS) and virtual screening (VS). (D) Herein pursued approach involving HT miniaturized multiple chemistries at once using rapid acoustic droplet ejection technology.

Large and diverse compound libraries are used in high throughput screening to discover novel biologically active matter.^1^ Often hits discovered from such compound libraries are the starting point of a medicinal chemistry optimization project which can lead to a drug for unmet medical needs.^2^ Large pharmaceutical compound libraries are usually assembled in-house by contract research organization (CRO) synthesis and through purchase from vendors. However, the assembly by synthesis is lengthy and by purchase expensive and often biased to the limited chemical space of vendors. The quality of a screening library is determined from the chemical space covered, the physicochemical properties, and its size.^1^ Strategies to maintain and enhance the overall quality of screening collections are highly sought after to stay competitive and work in proprietary protectable chemical space.^3^ Contemporary approaches towards a large compound screening space include DNA-encoded chemical library (DEL),^4^ fragment-based drug discovery (FBDD),^5^*de novo* structure-based drug design (SBDD),^6^ diversity-oriented synthesis (DOS),^7^ and virtual screening (Fig. 1).^8^ All the technologies have their advantages and disadvantages.^2^ A popular way to assemble compound libraries uses combinatorial chemistry (CC).^9^ In combinatorial chemistry, all possible combinations of available building blocks are combined around one chemical scaffold, and a dense matrix is formed. Such libraries often lack sufficient diversity and monotonous substituent placement is seen, when building around one or a few scaffolds. DEL technology makes perfect use of combinatorial chemistry by applying systematic variations of building blocks to create extensive libraries in a tiny volume. Combinatorial chemistry and DEL were successful at delivering starting points to develop drugs for clinical trials and the market.^4^ DOS aims to produce libraries based on skeletal diversity, often leaning on natural product-derived motifs by using modular syntheses involving a few steps (‘build-couple-pair’) and typically from common stereochemically complex precursors. In contrast, fragment libraries have already repeatedly proven their value by delivering clinical drugs.^5^ However, fragment library screening and hit optimization are demanding because fragments typically weakly bind to their target. Hence, significant effort has to go into stepwise building of more complex compounds to yield the required potency. Often fragment linking technologies are a not-straightforward undertaking and fail to exhibit the expected gains of binding potency.^10^ Virtual screening (VS) is making considerable progress and ultra-large libraries can be screened to obtain novel inhibitors.^11^ However, physics-based *in silico* screening of billion-sized libraries in an exhaustive manner is cost and time prohibitive. While machine learning can be combined with VS to feasibly tackle even larger chemical space, in the end synthesis and screening of the multi-step hits of non-commercially accessible compounds comprise the true bottleneck.^12^ Due to the major progress in protein crystallography, structure–activity-relationship (SAR) by crystallography in a high throughput (HT) mode is feasible at a shorter timeframe.^13^ Primarily static fragment libraries have been used for enzyme active sites or even protein–protein interactions.^14^ Routine times of around 2–3 weeks from soaking to multi cocrystal structure determinations are now possible. Recently, the integration of HT synthesis and HT protein crystallography has emerged as a promising novel approach to shorten the ubiquitous ‘design–make–test–analyze’ cycle (DMTA).^15^

While there is no ‘one-size-fits-all’ library method, we are reporting an alternative technology involving miniaturized and automated nanoscale syntheses applied to many different chemistries, and multicomponent reactions (MCRs), in parallel. In classical approaches, one scaffold is elaborated at a time. Here we produce exemplary libraries based on 16 different scaffolds at the same time, thus yielding unprecedented scaffold and library diversity. Moreover, due to the miniaturization aspect, the approach reduces the ecological footprint of chemistry enormously while increasing the speed of high quality library production. The rationale for choosing MCR is that this reaction class is well known to provide excellent scaffold diversity, based on many building blocks which are commercially available and have tremendous functional group compatibility. Indeed, the scaffold diversity of MCR compares favorably with sequential syntheses in the pharmaceutical industry relying on only a few well-characterized reactions.^16^

## Results

Several well-defined, robust MCRs are selected for synthesis based on previous experience in our laboratory (Table 1).^17^

**Table 1:** Structures of 16 MCR scaffolds for miniaturized high throughput chemistry. The reaction schemes, components, and names are given. The building block classes R's are shown in different colors and numbers to indicate their repeated use in different MCRs. Stereoscopic views of each scaffold obtained with Pymol are shown with methyl substituents. Blue = nitrogen, red = oxygen, white = hydrogen, and other colors = carbons. Representative references of these 16 MCR scaffolds are cited as ref. 8–23 in the ESI.†

No.	Reaction scheme	3D view
1	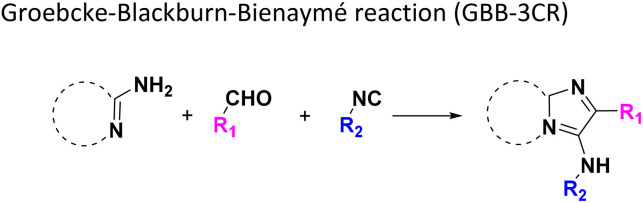	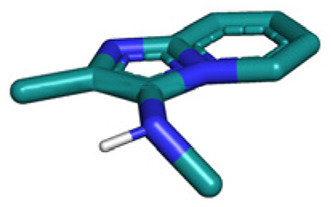
2	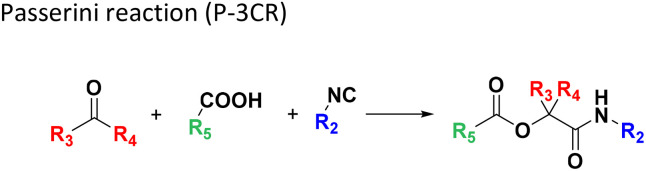	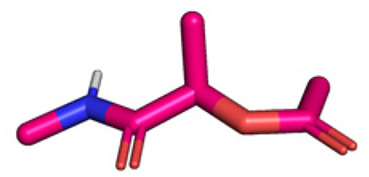
3	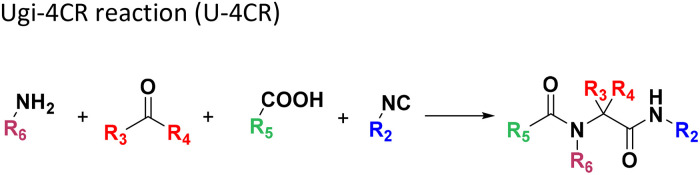	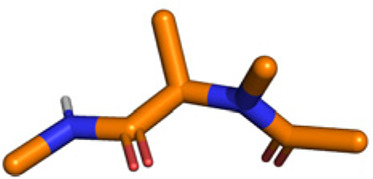
4	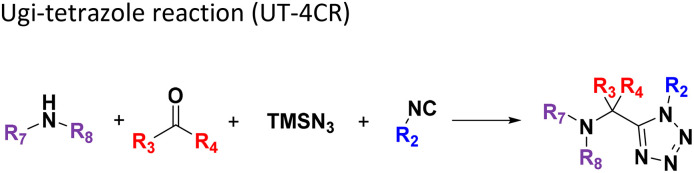	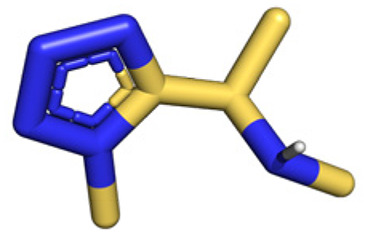
5	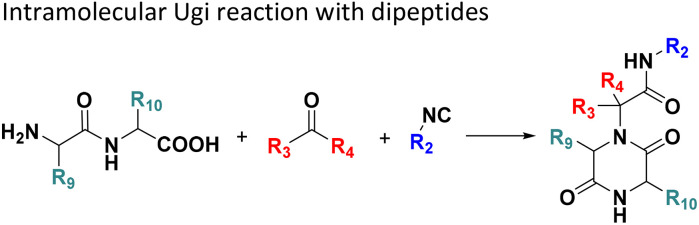	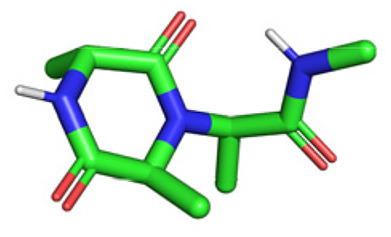
6	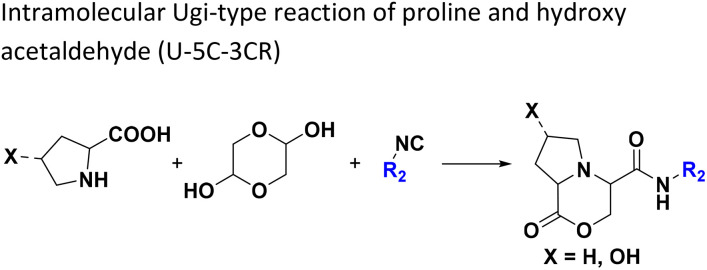	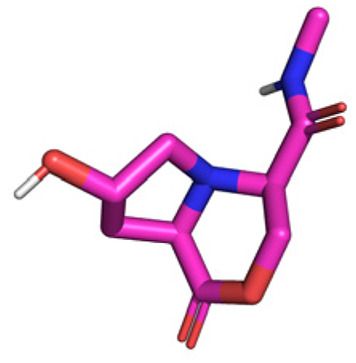
7	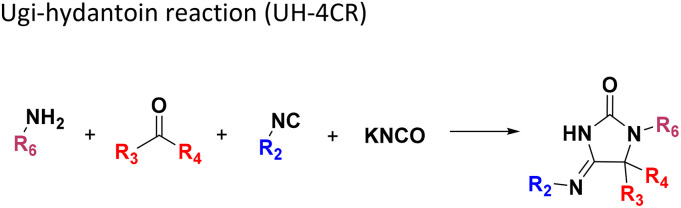	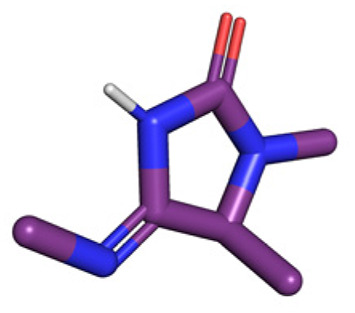
8	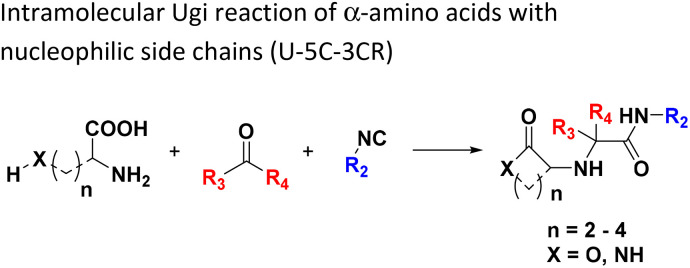	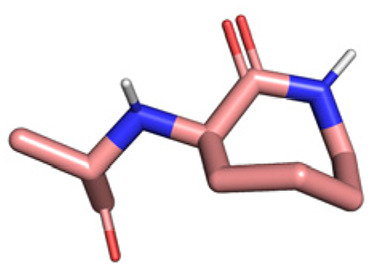
9	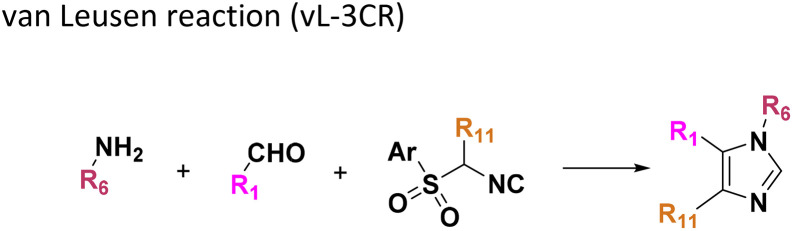	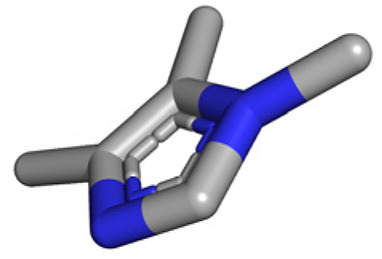
10	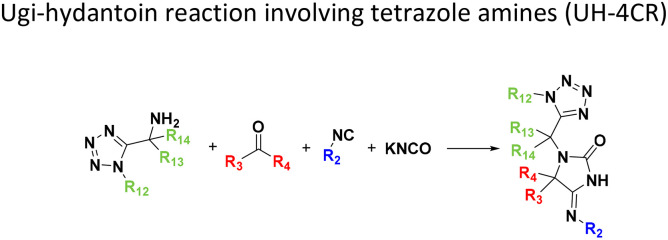	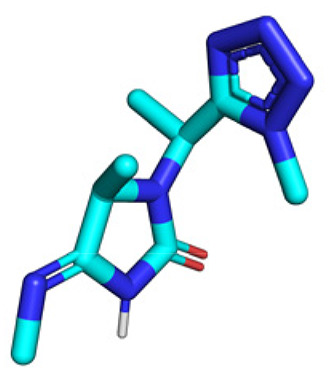
11	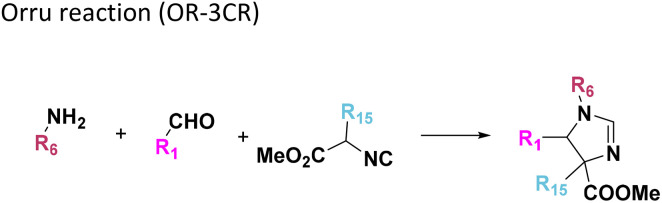	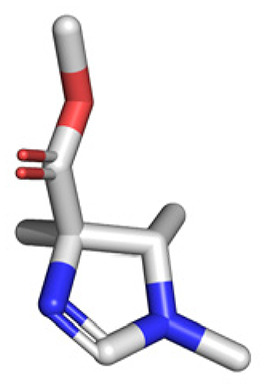
12	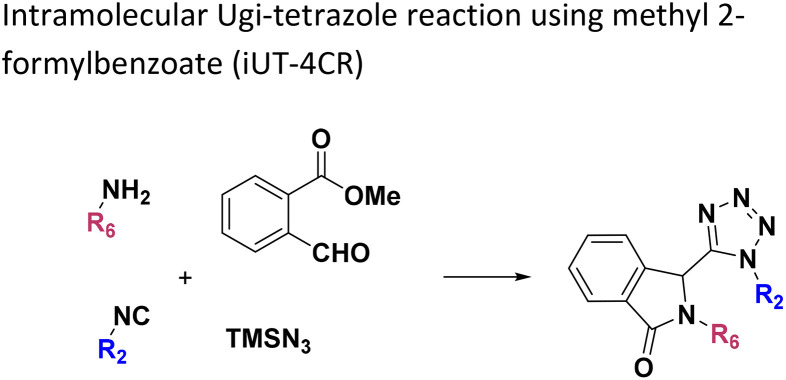	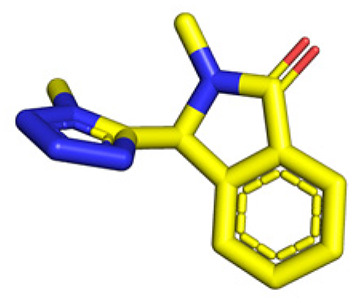
13	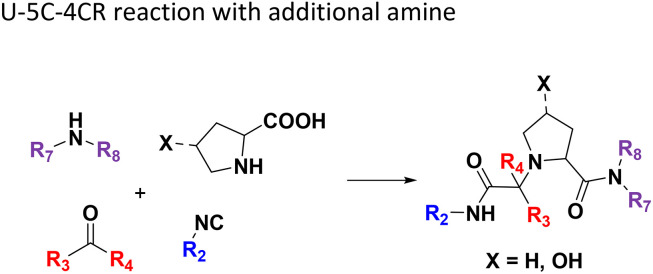	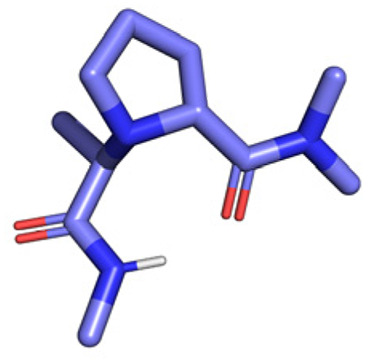
14	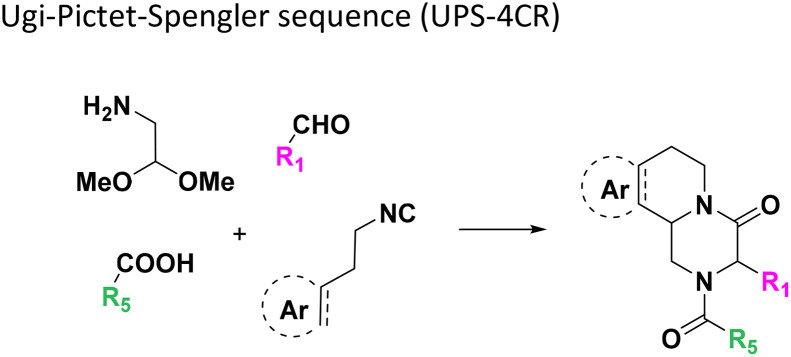	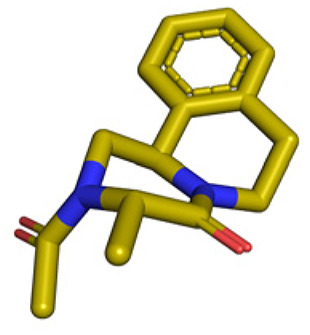
15	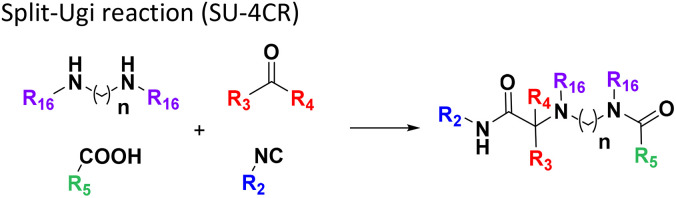	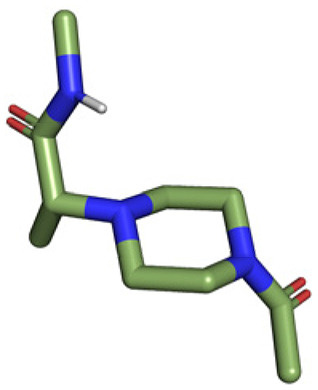
16	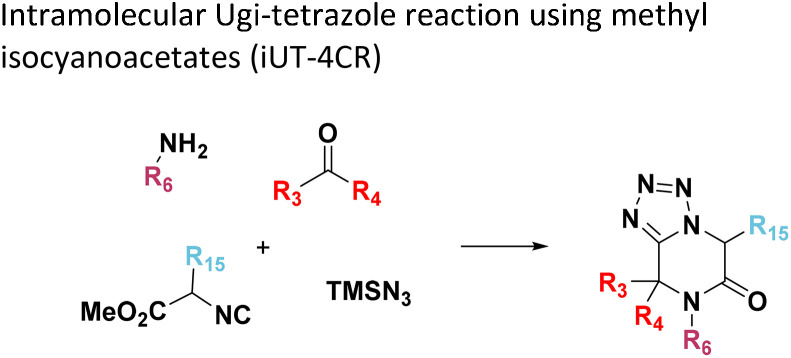	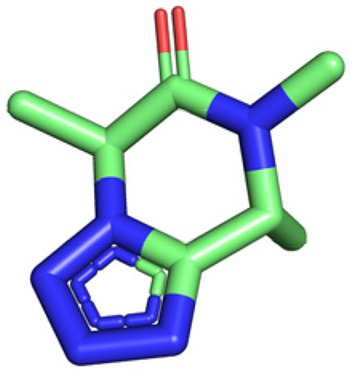

All reactions have in common the use of isocyanides (Isocyanide-based Multicomponent Reactions, IMCRs).^18^ The reactions were chosen to reflect a balanced 3D-scaffold and -substituent space ranging from flat heteroaromatic (Table 1: reactions (1), (4), (7) and (9)), non-flat mono heterocyclic (Table 1: reactions (5), (8), (11) and (13)), bicyclic not annulated (Table 1: reactions (10) and (12)), bicyclic annulated (Table 1: reactions (6) and (16)), and polycyclic (Table 1: reaction (14)) to non-cyclic scaffolds (Table 1: reactions (2), (3) and (15)).

**Fig. 2:**
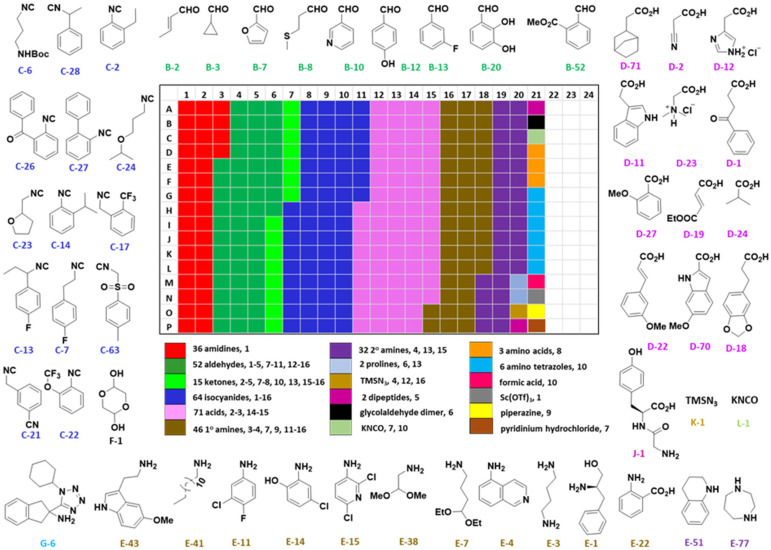
Selected representatives of the 336 building blocks, and reagents distributed on a 384-well source plate. The colors indicate the different building block classes. The total number of each building block and the reaction number where each building block is used are mentioned (*e.g.* below the heat map “36 amidines, 1” means 36 amidines are used in reaction (1), Table 1).

The synthesis was performed on an Echo 555 acoustic dispensing platform. In acoustic droplet ejection (ADE), precise nanodroplets are formed by applying acoustic waves to the stock solutions of the building blocks in the source plate and transported above to the inverted destination plate, where the reaction is taking place.^19^ A total of 336 building blocks and reagents were used here, including cyclic 5- and 6-membered aromatic amidines (36), aldehydes (52) and ketones (15), isocyanides (64), carboxylic acids (71), 78 amines (46 primary and 32 secondary amines), all fitting into one 384-well source plate (Fig. 2, ESI, section 2.3†). Due to their rapid evaporation, volatile solvents such as methanol, THF or DCM are not suitable for ADE technology. Hence, we chose ethylene glycol in most cases as a ‘transporter solvent’ for stock solution preparation, or 2-methoxyethanol or dimethoxyethane when the building block was not soluble in ethylene glycol. All soluble building blocks were prepared as 0.5 M solutions or otherwise diluted to 0.25 M or 0.16 M solutions. The stock solutions were kept in 384-well source plates sealed with tape and kept at −20 °C before combining them in a corresponding 384-well destination plate using an Echo 555 liquid handler. The scale of each reaction per well was 300, 375, or 500 nanomoles directed by the number of components/reagents transferred. Each of the 16 MCR scaffolds was designed for 96 different products to investigate the substrate scope and limitations by choosing the corresponding building blocks. Therefore, four destination 384-well plates were filled by the Echo 555 liquid handler to generate 1536 reactions in total. In a sparse matrix approach, 1536 reactions were performed, however, reflecting only a small fraction of ∼0.1 *per mille* of the possible combinations based on the 16 different chemistries (Table 1). Indeed, the 332 building blocks can be theoretically combined to give 16 446 250 products. Once the transfer of the starting material to the destination plates was complete (∼150 min for each 384-well plate), 10 μL of the appropriate solvent for each reaction was added using a multichannel pipette (ESI, section 2.5†). For example, MeOH was added to GBB-3CR, Ugi-4CR, and UT-4CR (Table 1: reactions (1), (3) and (4)) and CHCl_3_ was added to Passerini-3CR (Table 1: reaction (2)). The destination plates were sealed and placed for 12 h at 21 °C on an orbital shaker. After desealing, the solvent (MeOH and CHCl_3_) was evaporated for further analytics. To the intramolecular Ugi reaction with dipeptides, trifluoroethanol (TFE) was added (Table 1: (5)). One scaffold (Table 1: reaction (14)) is in fact a two-step reaction sequence consisting of an initial Ugi-4CR, followed by an acid-catalyzed Pictet–Spengler reaction.^20^ In this case, 10 μL of MeOH was added to the initial Ugi reaction. The destination plate was sealed and placed for 12 h at 21 °C on an orbital shaker. After desealing to let the MeOH evaporate (∼3 h evaporation time), 10 μL of formic acid was added as a catalyst for the Pictet–Spengler reaction. The reaction plate was sealed again and kept at 60 °C for 2 h. The plate was kept at −20 °C for further analytics, after desealing and formic acid evaporation (∼3 h), (Table 1: reaction (14)). The experimental details of each scaffold of the 16 MCR reactions on the plates are described in the ESI (section 2.5).†

The building blocks were chosen to optimize diversity in terms of electronic structures (electron donating–withdrawing), and steric (small, large, cyclic, acyclic) and orthogonal (halogen, hydroxyl, amine, carboxylic acid, heterocycle) functional groups (Fig. 2).

**Fig. 3:**
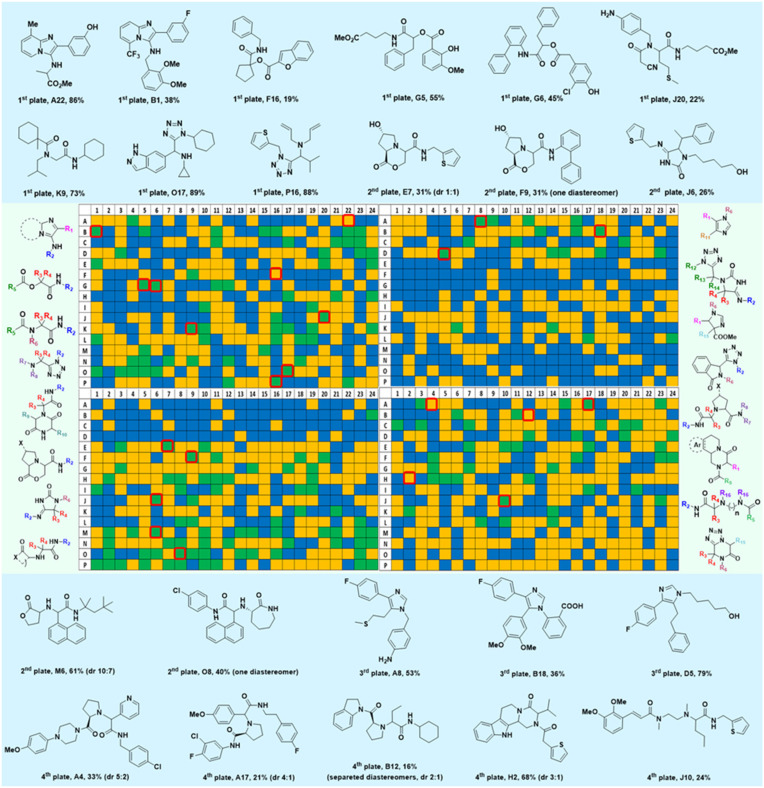
1536 reactions based on 16 different chemistries for the parallel assembly of a highly diverse library of small molecules. The generic scaffolds are shown in the green boxes left and right of the 1536 well plate. The MS-analysis is plotted on the 1536-well plate based on a three-color code (blue = no product formation, yellow = medium product formation, green = major product formation). The structures of the randomly resynthesized compounds (red boxes) from each scaffold on a mmol scale are shown in blue boxes together with their isolated yields.

For analytics of the 1536 reactions, 100 μL of ethylene glycol was added to each well using a multichannel pipette. The reaction wells were automatically analyzed by SFC-MS overnight using our previously described python script (Fig. 3).^21^

**Fig. 4:**
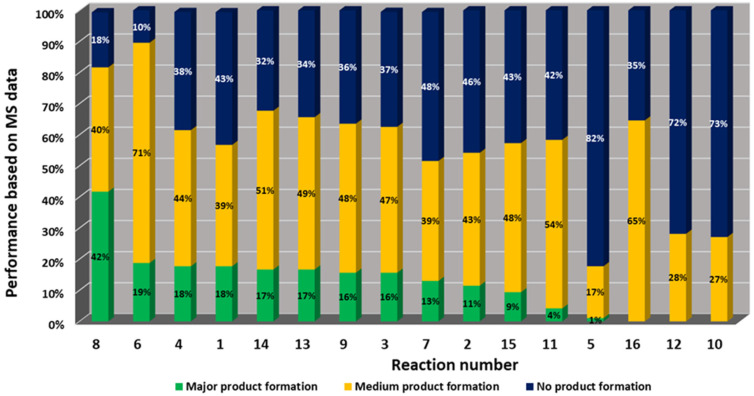
Statistical reaction analysis: performance of 16 MCR reactions based on MS analysis in descending order.

Mass spectrometry analysis of the reactions gives information on the performance of the different chemistries and the reactivity of the different building blocks and combinations under the performed reaction conditions (Fig. 4). It was found that amongst the 16 performed chemistries, 13 performed well, producing the products in major or medium quantities. Exemplary reactions are discussed in the following.

The GBB-3CR (Table 1: reaction (1)) is a popular reaction in medicinal chemistry which was used in the discovery of tool compounds for the first bromodomain of human BRD4^22^ and was instrumental in the development of the clinical autotaxin inhibitor Ziritaxesta® that is used for the treatment of idiopathic pulmonary fibrosis.^23^ A recent comprehensive review reveals that GBB-3CR is a versatile MCR variation of the Ugi reaction, which can be performed under a broad range of conditions. However, there is no ‘one-size-fits-all’ procedure, but the exact reaction conditions (catalyst, temperature, solvent) have to be adjusted to the building blocks used. For example, more than 20 different Lewis and Bronsted acid catalysts have been reported to be of use in the GBB-3CR.^24^ In particular, the nature of the five or six membered aromatic amidine plays a crucial role and the yields can vary largely depending on the reaction conditions. Nonetheless, we choose the following generalized conditions for the nanoscale syntheses daring non-optimal reaction conditions for all building block combinations: 0.166 M of all starting materials in ethylene glycol, 10 mol% Sc(OTf)_3_, room temperature, 12 h.

**Fig. 5:**
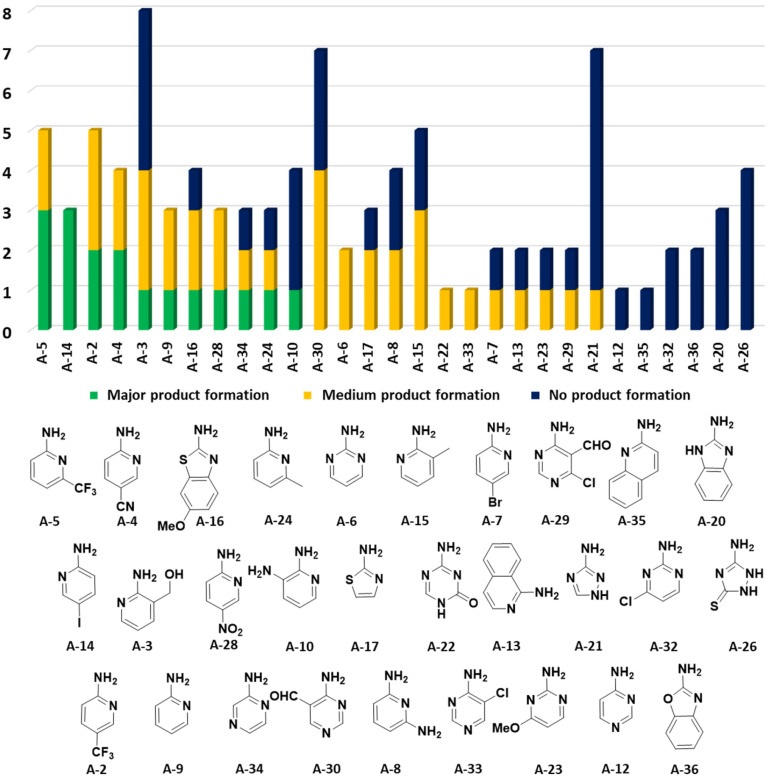
Performance of the cyclic amidine building blocks in the GBB-3CR. The number of reactions is indicated on the *Y*-axis and the nature of the amidine on the *X*-axis.

In the 96 GBB-3CRs, product formation was indicated by MS analysis in 57%, and in 18% as the major product. Analysis of the amidine structures reveals that 2-aminopyridine with electron-withdrawing groups (NO_2_, CF_3_, CN, I) reacted well (Fig. 5). Aminotriazoles (A-21, A-26) on the other hand reacted not at all or showed medium reaction success. This can be explained by the general electron-deficient nature of the triazole heterocycles and the mechanism of the GBB-3CR which requires the condensation of the exocyclic triazole nitrogen with the aldehyde component. Not surprisingly, triazole-based GBB-3CR reactions, previously, were performed under elevated temperatures or microwave assistance.^25^

The high success rate (major and medium product formation 57%) was rather surprising, in view of the strong dependency of the GBB-3CR performance on the nature of the Bronsted or Lewis acid catalysts, the solvent and the temperature, besides the electronic and steric influences of the nature of the building blocks.^24^

The intramolecular Ugi reaction of α-amino acids (U-5C-3CR, Table 1, reaction (8)) with suitable nucleophilic side chains is an interesting Ugi variation for several reasons.^26^ First, it utilizes chiral pool α-amino acids which direct the newly formed stereocenter at a high diastereomeric ratio. Second, the nucleophilic α-amino acid side chain is involved in the formation of 5- to 7-membered rings. Third, some members of this family, are known to exhibit potent biological activity, *e.g.*, GABA mimics with potent *in vivo* anticonvulsant activity.^27^

**Fig. 6:**
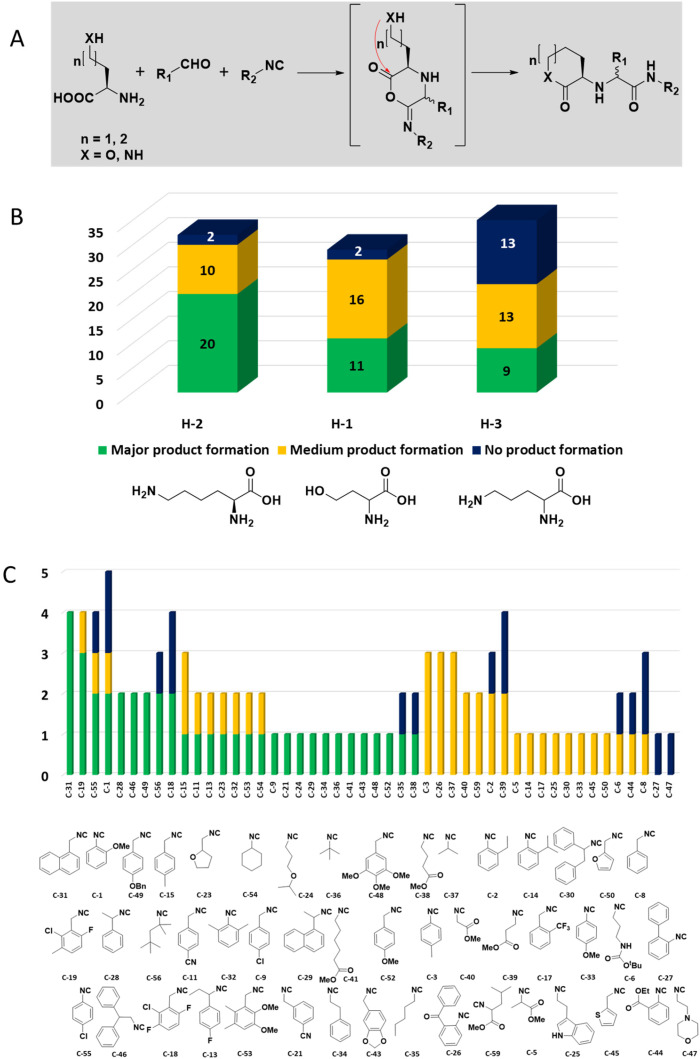
Intramolecular U-5C-3CR performance of three different α-amino acids with nucleophilic side chains. (A) Synthesis scheme with the key α-adduct intermediate. (B) Statistics of the performance by the α-amino acid building block as analyzed by the peak height of direct MS injection. (C) Reactivity/performance of the isocyanides in descending order.

For this Ugi MCR variation lysine, penta homo-serine, ornithine, and homo-cysteine have been described as multi-functional substrates in different publications, but no comparative study was performed ever.^28–32^ In our HT synthesis we used available lysine, penta homo-serine, and ornithine, yielding the corresponding 7- and 6-membered lactones and 5-membered δ-lactam, respectively. Interestingly, the 7-membered lactones were formed best, followed by the 5-membered δ-lactams and the 6-membered lactones (Fig. 6).

Amongst the 16 performed chemistries, the diketopiperazine (DKP) formation using dipeptides in the Ugi reaction, the Orru 3-component reaction, the methyl 2-formylbenzoate UT-4CR and the tetrazolo ketopiperazine resulted in mostly medium or no product formation (Fig. 4). Potential reasons for their poor performance are discussed here. The intramolecular Ugi reaction of dipeptides leading to DKP formation (Table 1, reaction (5)) was described only one time in the literature.^33^ A closer inspection of the work reveals that very specific conditions were applied, a mixture of [Bmim]PF_6_ and TFE (CF_3_CH_2_OH) was used as a solvent system and the reaction was performed under microwave conditions. Ethylene glycol at room temperature were the conditions used here and are far from the optimized conditions yielding the DKP. The Orru 3-component reaction (Table 1, reaction (11)) of α-acidic isocyanides, primary amines and aldehydes has been often described but is rather solvent specific and mostly involved the use of chlorinated solvents and often Ag^I^ catalysis.^34^ The intramolecular Ugi tetrazole reaction involving *o*-formylbenzoic acid methylester (Table 1, reaction (12)), described twice, also employs drastic reaction conditions (refluxing solvent, or NaOEt) to accomplish lactam ring formation.^35^ Under the herein applied conditions no product formation was observed. Finally, the successful transformation into fused tetrazolo ketopiperazine (Table 1, reaction (16)) required prolonged refluxing conditions in methanol.^36^

### Scalability of the 16 reactions

Given the highly miniaturized nature of ADE, reproducibility on a larger scale needs to be addressed, which is done here through the scale-up of a selection of wells to validate translation into more traditional conditions. To show the scalability of the nanoscale reactions, we randomly resynthesized 22 compounds with featured structures chosen from the 16 scaffolds in the 1536-plate. All products were resynthesized on a 1 mmol scale and the yields were determined after chromatographic purification by weighing (Fig. 3). All the resynthesized compounds are unprecedented and based on known chemistries. J20 (1^st^ plate) incorporates the cyanoacetamide moiety, which is a popular motif in covalent inhibitors, for example in the Janus kinase inhibitor Tofacitinib.^37^ G5 and G6 (1^st^ plate) feature free phenolic hydroxyl groups which are perfectly compatible functional groups under the mild reaction conditions of the P-3CR. Likewise, esters (F16, G5, G6, J20), aniline-NH_2_ (J20), alkenes (P16), primary aliphatic hydroxyl (J6), secondary hydroxyl (E7, F9), secondary amine (O8, O17), tertiary amine (A4, A17, B12, J10, O17, F16, E7, F9), thioether (J20, A8), and free carboxylic acid (B18) can be incorporated problem-free into the products not requiring tedious protecting group chemistries. Particularly noteworthy is the unprecedented smooth incorporation of the free carboxylic acid (B18) and free aniline (J20) without any protecting group manipulations, resulting from the anthranilic acid E-22 and 4-aminobenzylamine building block E-26 in the vL-3CR (Table 1, reaction (9)). Several heterocycles are featured in the resynthesized compounds, including thiophene (P16), indole (H2), tetrahydroindole (B12), piperazine (A4, H2), pyrrolidine (A4, A17, B12, E7, F9), imidazole (A8, B18, D5), and γ-lactones (M6, O8), δ-lactam (O8), tetrazole (P16, O17), imidazopyridine (A22, B1), benzofurane (F16), morpholine (E7, F9), azaindole (O17), and pyridine (A4), underscoring the broad usefulness of IMCR in producing diverse libraries. In summary all 22 nanoscale reactions selected for resynthesis could be synthesized and characterized on a mmol scale as well.

### Chemoinformatic analysis

The calculation and analysis of physicochemical descriptors is an important step in library design and can help to avoid compounds with poor properties, leading to poor solubility, absorption, issues with screening assays, or causing pan assay interference (PAIN).^3^ To investigate the physiochemical and biopharmaceutical diversity of the 1536 compounds based on 16 scaffolds, we conducted a comparative chemoinformatic analysis with Food and Drug Administration (FDA) approved drugs (ESI, section 5.1†).^38,39^ The calculated and analyzed descriptors include molecular weight (MW), partition coefficient (cLog *P*), number of hydrogen bond acceptors/donors (NHA/NHD), total polar surface area (TPSA), and number of rotatable bonds (NumRotBonds) and partition of a chemical compound between the lipid and aqueous phases (cLog *D*). Initially we investigated the dependency between cLog *P* and MW which are the first indicators of solubility and membrane permeability during hit optimization. Using our random-compound-generator algorithm, all scaffolds produce more lipophilic compounds as the MW increases (Fig. S7†), except for some compounds clustered at the top of the chart as the polarity increases. Noteworthily, 33% reside in a preferred area of MW < 500 Dalton and cLog *P* 2–4. Interestingly, certain scaffolds are narrowly distributed (6, 14), whereas others show a broad distribution (3, 4, 16). Next, we conducted a more in-depth diversity study by assessing the distribution of the other five properties (Fig. S8†) and quantifying the statistical significance between them (Fig. S9, ESI, sections 5.2 and 5.8†). This resulted in a total of 952 possible pair-wise comparisons for each property between the 1536 compounds based on 16 scaffolds and the FDA compounds. More than half of these comparisons showed highly significant statistical differences except for cLog *D*. This implies a broad spectrum of molecular diversity in terms of MW, membrane permeability, flexibility, polarity, NHA and NHD which are crucial parameters for binding ligand/receptor interaction and aqueous solubility. Furthermore, we studied the drug likeliness profile of each molecule with Lipinski, Ghose^40^ and Muegge scores^41^ (ESI, section 5.3†). Importantly, our results confirm that the 11 scaffolds produce more than 50% of compounds that pass all three biopharmaceutical filters (Fig. S10 and S11†), and hence ‘druglike’ molecules.

We also assessed the Central Nervous System Multi-Parameter Optimization (CNS MPO) desirability score as illustrated in Fig. S12.†^42^ We differentiated the compounds between high CNS MPO (>5) and low CNS MPO (≤2) scores (Fig. S11A†).^43^ Thus, compounds with CNS MPO > 5 could cross the blood–brain barrier (BBB) and exhibit pharmacological activity in the central nervous system (CNS). Most scaffolds display low CNS MPO scores in at least 75% of the compounds, because they were not designed specifically for CNS penetration (Fig. S13A†). Interestingly, scaffold 16 displayed a higher CNS MPO score (31%) than the FDA drugs (26%), while scaffold 10 returned the lowest score (0%). These data indicate that reaction (16) could produce molecules highly penetrable through the BBB.

As shown by PCA analysis in Fig. S13B,† both structural flexibility (NumRotBond) and basicity (basic p*K*_a_) were found to be the most representative variables in influencing BBB penetration of scaffold 16 over scaffold 10. To investigate even further which properties positively affect scaffold 16 CNS MPO score, we conducted a principal component analysis (PCA) (ESI, section 5.4†) employing TPSA, MW, NumRotBonds, cLog *D*, cLog *P* and Strongest basic p*K*_a_ as descriptors. The first three principal components described 93.1% of the variance within the data (Table S1†) with the first component carrying a high load from MW (0.963) followed by Log *P* (0.820), NumRotbonds (0.791), Log *D* (0.767) and the strongest basic p*K*_a_ (0.570) (Table S2†). The data points are centered around the origin with no dispersion or observable outliers. Interestingly, a clear separation of the molecules of reaction (16) from reaction (10) is visible. Surprisingly, the highest contribution to the first (27%) and second largest components (33%) is from the MW and TPSA (Table S3†). Taking it together, we hypothesized that scaffold 16 is advantaged by less conformational freedom since tetrazole is *ortho*-fused with the piperazinone ring. We speculate that the general structural rigidity contributes importantly to the BBB. For example, the scaffold of 16 molecules consists of two *ortho*-fused rings, a tetrazole, and a piperazinone. As a result, they are subject to less freedom in conformational space rather than those of scaffold 10. Additionally, scaffold 16 contains fewer exit points than scaffold 10 (4 *vs.* 6) where polar and bulky functional groups can be installed. This allows scaffold 16 molecules to more easily permeate the dense phospholipid layer of the BBB.

**Fig. 7:**
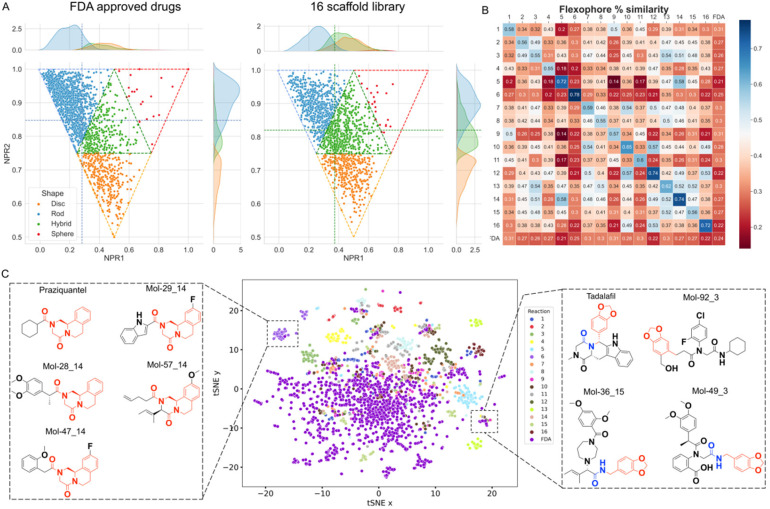
Chemoinformatic analysis and chemical diversity of our 16 scaffold library produced by ADE technology. (A) Averaged normalized ratios of principal moments of inertia (NPR) of FDA approved drugs and our library. The kernel density distribution of the two principal moments is displayed. The average shape between all points is identified by the intersection of the density values calculated on both axes (dashed lines). Molecules occupying the “rod”, “sphere”, “hybrid”, and “disc” triangles are colored as blue, red, green, and orange dots. (B) Pharmacophoric similarity matrix indicated on a divergent scale from blue (high similarity) to brick red (low similarity). (C) *t*-Distributed stochastic neighbor embedding (*t*SNE) chart showing the diversity of our library *versus* the FDA approved drugs. The two boxes contain clusters of similar molecules to the FDA approved drugs Praziquantel and Tadalafil. Examples of structures corresponding to clusters densely populated by the reference group (purple) and the 16 scaffold library are framed in the scatter plot.

Next, we performed an exhaustive shape analysis. The complementarity of the molecule and receptor shape is an important prerequisite for efficient binding. Normalized ratios of the three main moments of inertia (NPR) address the diversity of receptor shapes of a general screening library and were calculated (ESI, section 5.5†). On the basis of the abundance and arrangement of rings, alkyl chains or internal cyclization a scaffold library can be intuitively classified into three categories, namely spheroidal, discoidal, and flat.^44^ As shown in Fig. 7A, the molecules adopt specific molecular shapes. The molecule count presented in Table S6† shows that FDA approved drugs primarily tend to assume a rod-like shape (65%), followed by discoidal (19%), hybrid (16%) and spherical (1%) shapes. On the other hand, our library based on 16 scaffolds assume a rod (41%), hybrid (34%), disc (24%) and sphere (2%) shape with more compounds in the last three spatial regions. Overall, the barycenter, calculated from the averages of the kernel densities for both moments of inertia, confirms that the FDA approved drugs tend to be more rod-like whilst the 16 scaffold library populate a region of hybrid forms. To investigate the different trends, we conducted a comparative non-parametric Kolmogorov–Smirnov test to elucidate whether the cumulative distribution function (CDF) values for each shape belong to the same distribution (ESI, section 5.8†). The differences among the rod, sphere and disc shapes are highly statistically relevant (*p*-values < 0.001, Table S7†), indicating that our library can produce indeed higher molecular shape diversity than the FDA approved drugs, with more spherical and hybrid shapes and less rod-like or discoidal shapes.

Understanding the pharmacophore distribution of a chemical space is an additional way to assess the shape diversity of libraries. For this, we investigated the pharmacophoric diversity of our scaffolds using Flexophore descriptors (Fig. 7B, ESI, section 5.6†).^45^ The highest values of structural similarity are distributed along the diagonal of the matrix, except for the FDA group. This is because each of the 16 scaffolds has been designed to share one common scaffold, while the majority of FDA approved drugs are not built on common scaffolds. Noteworthily, 51% of the cross-comparisons between different scaffolds in the array show pharmacophoric similarity below the average calculated over the entire matrix (0.36) (Table S4†). These data indicate a wide pharmacophoric diversity and a lower propensity to yield similar molecules with different reactions. This implies that our library based on the selection of 16 scaffolds leads indeed to highly dissimilar molecules.

Yet another way to assess the structural similarity between the 16 scaffolds and FDA drugs is by *t*-distributed stochastic neighbor embedding (*t*SNE). To find any similar substructures between our library and the FDA approved drugs, we have employed SkeletonSpheres descriptors (ESI, section 5.7†).^46^ The data points corresponding to the 16 scaffolds are distributed more widely than the reference FDA group (Fig. 7C) indicating that our synthetic approach covers a chemical space not yet explored by current drugs. On the other hand, it is possible to discover close analogues of FDA approved drugs in our 16-scaffold library. An example is given by Mol-8, Mol-13, Mol-39 and Mol-91 from scaffold 14 sharing the same substructure with the anti-schistosomiasis drug Praziquantel, previously synthesized by Cao *et al.* employing the same reaction.^20^

Analysis of the neighbor tree map (Fig. S14†) highlights another potential therapeutical application based on the pharmacophoric similarity of our nano-scale library to FDA approved drugs. For example, Mol-87 from the Ugi-hydantoin reaction shares 0.92 Flexophore similarity with Doxapram, an analectic drug used to stimulate respiration in alveolar hypoventilation. *Vice versa*, the closest FDA drugs to Mol-18, Mol-31 and Mol-76 obtained from the Orru reaction find approved applications as muscle relaxants to treat injuries (Chlorphenesin, 0.91), antihistamines for the treatment of the relief of nasal congestion (*e.g.*, Antazoline, 0.91), analgesic or anti-inflammatory (Mefenamic acid 0.90, Fenoprofen 0.91) and calcium channel blocker for containment of ischemic damage (Nimodipine 0.93). Interestingly, the van Leusen scaffold (Table 1: 9) retained the largest number of compounds like FDA drugs. For example, the pharmacophoric motifs that Mol-36 shares with its hits are a benzene ring and/or a basic center, embedding pi-stacking and ionic features, respectively. Several of these hits are prescribed as cardiostimulants like Mephentermine (0.99) or heartbeat correctors such as Mexiletine (0.94), anorectics for the treatment of obesity such as Phentermine (0.99) and Phendimetrazine (0.98), psychostimulants for the treatment of attention deficit hyperactivity disorder (*e.g.*, Amphetamine, Methamphetamine, Dextroamphetamine, all with 0.99 Flexophore similarity), and antidepressants (*e.g.*, Phenelzine, 0.93). Other isolated applications include anti-poisoning by organophosphate Pralidoxime (0.99), and treatment of arterial hypertension (Bethanidine 0.95) and appetite suppression by Phenmetrazine (0.97, now withdrawn for its abuse). Finally, Mol-2 is sharing 0.97 and 0.92 of Flexophore similarity to Methoxamine and Tramadol, for the treatment of hypotension and moderate to severe pain, respectively.

## Discussion

The physical presentation and exploration of chemical space is usually a sequential and slow process. We have previously introduced ADE in the organic chemistry of small molecules with the advantages of its extraordinary degree of automation and scale to perform many reactions at the nanoscale. We used ADE in focused projects to automatically scout and elaborate the scope and limitations of new compounds including isoquinolines^47^ or quinazolines.^48^ Moreover, we used ADE for the selective synthesis of libraries of compound classes, such as boronic acids,^49^ indoles^50^ or covalent inhibitors at the nanoscale.^51^ We also described the *in situ* synthesis and screening of nanoscale GBB-scaffold based libraries for the discovery of potent protein–protein interaction antagonists in a ‘direct-to-biology’ approach.^52^ On an unprecedented breath, here we describe for the first time the automated acoustic dispending-enabled nanoscale synthesis of multiple different scaffolds in parallel. In contrast to classical library synthesis approaches, not one scaffold is synthesized at one time, but 16 different scaffolds based on 16 different chemistries, considerably accelerating synthetic chemistry and widening the chemical space. The acceleration and miniaturization of synthetic chemistry are becoming significant parameters to discover novel drugs and materials in an age of big data and artificial intelligence driven discovery and manufacturing in a timely and economical fashion.^53,54^ The analysis of the 1536 reactions provides insight into the scope and limitations of compatible building blocks and subsequently can be instrumental for reaction optimization.^55^ ‘Chemistry at the speed of sound’ can be used for the generation of diverse compound libraries for the identification of biologically active compounds or material properties. Indeed, our chemoinformatic analysis of the 16 scaffolds supports the high diversity and drug-likeliness of our *in situ* synthesized chemical space. While we employed here just 16 different chemistries, it is conceivable that the scaffold diversity can be further increased (*e.g.* 48 × 32 = 1536) to produce even more diverse libraries ‘on the fly’ in a high density format. Library synthesis as shown here is practical, as for each scaffold type several derivatives are presented. In the case of the identified screening hits, neighbors from the same scaffold class allow a first rapid SAR. Our ADE approach also allows for a fast evaluation of the scope and limitations of multiple reactions in parallel. Under the reaction conditions used here, 12 out of 16 scaffolds worked well in more than 50% of the cases, whereas 4 scaffolds resulted in less than 30% successful transformations. Detailed analysis of the generated ‘big data’ can help to understand the compatibility of electronic and steric features in the different building blocks. High throughput analysis of more than 1000 reactions enables high throughput experimentation to optimize the reaction conditions for library synthesis or to deselect building blocks which repeatedly do not result in successful product formation. Another important advantage of this library synthesis approach is its sustainability. One of the 12 principles of green chemistry is ‘to better prevent waste than to treat or clean up waste after it is created’. Clearly, during the process of property optimization, unavoidably, hundreds or even thousands of derivatives have to be synthesized and tested. On a classical mmol scale, thousands of compounds are generated in the discovery phase, and the synthesis of 1536 compounds would use hundreds of kg of highly valuable building blocks, solvents and purification materials as well as consumables to produce 20 mg of each compound. Amazingly, our campaign of synthesizing 1536 novel compounds at the nanoscale used a total of ∼7 mg of building blocks and ∼20 mg of solvent. The high prices of building blocks are a true economic burden. For example, a 96-well plate of pre-weighed building blocks on a 0.2 mmol scale easily costs ∼15 000 USD. However, high building block diversity is a prerequisite for the construction of diverse general screening libraries. Moreover, due to the fully automated synthesis set-up, the contact time of the bench chemist with the toxic solvent and chemical fumes is reduced to a minimum. ‘Chemistry at the speed of sound’ can therefore considerably help to reduce the environmental footprint of organic synthesis for drug discovery. Importantly, automated and fast microscale purification in support of high-throughput medicinal chemistry was recently described to avoid potential false positives through the screening of unpurified compounds.^56^ Given the speed of ADE and the structural scaffold diversity of IMCR it is conceivable that many more chemistries can be performed in parallel. However, the true advantage of the herein described HT miniaturized high diversity chemistry will become apparent by the integration with HT screening and machine learning guidance to optimize compound properties to reduce the time and effort and increase the throughput and sustainability of the ubiquitous ‘design-make-test-analyze’ cycle.^57^

## Conflicts of interest

The authors declare no competing financial interest.

## Supplementary Material

GC-025-D2GC04312B-s001
