# Correction: ‘Chemistry at the speed of sound’: automated 1536-well nanoscale synthesis of 16 scaffolds in parallel

**DOI:** 10.1039/d3gc90037a

**Published:** 2023-05-03

**Authors:** Li Gao, Shabnam Shaabani, Atilio Reyes Romero, Ruixue Xu, Maryam Ahmadianmoghaddam, Alexander Dömling

**Affiliations:** a Department of Drug Design, University of Groningen Groningen The Netherlands a.s.s.domling@rug.nl

## Abstract

Correction for ‘‘Chemistry at the speed of sound’: automated 1536-well nanoscale synthesis of 16 scaffolds in parallel’ by Li Gao *et al.*, *Green Chem.*, 2023, **25**, 1380–1394, https://doi.org/10.1039/D2GC04312B.

The authors regret that there was an inaccuracy present in the affiliation information for one of the authors, Alexander Dömling, in the original manuscript.

The work was performed at the Department of Drug Design, University of Groningen, Groningen, The Netherlands (a.s.s.domling@rug.nl) before the transition of Alexander Dömling to their current institution, CATRIN, Innovative Chemistry Group, Palacký University Olomouc, Olomouc, Czech Republic (alexander.domling@upol.cz). The corrected list of author information and affiliations for this article is as shown here.

The Royal Society of Chemistry apologises for these errors and any consequent inconvenience to authors and readers.

## Supplementary Material

