# Supplementary material for: ‘Chemistry at the speed of sound’: automated 1536-well nanoscale synthesis of 16 scaffolds in parallel
Source: Green Chem. 2023 Jan 17;25(4):1380–94. doi: 10.1039/d2gc04312b (PMC9940305; doi:10.1039/d2gc04312b)
Supplement: GC-025-D2GC04312B-s001 [file GC-025-D2GC04312B-s001.pdf]

Electronic Supplementary Material (ESI) for Green Chemistry.
This journal is © The Royal Society of Chemistry 2023

'Chemistry at the speed of sound': Automated 1536-Well Nanoscale Synthesis of 16 Scaffolds in Parallel

Li Gao,^ Shabnam Shaabani,^ Atilio Romero, Ruixue Xu, Maryam Ahmadianmoghaddam, and Alexander Dömling*

Pharmacy Department, Drug Design Group, University of Groningen, A. Deusinglaan 1, 9700 AD Groningen, The Netherlands

^ authors contributed equally

*Correspondence to: *a.s.s.domling@rug.nl*

Table of contents

1. General information 3
2. Nano-scale automated chemistry 3
   2.1. General materials 3
   2.2. Instrumentation 3
   2.3. Structures of the building blocks 4
   2.4. Stock solution preparation 8
   2.5. Nano-scale synthesis 9
   2.6. Pick list preparation 13
      Fig. S6. Heat maps with product structures. 71
   2.7. Quality control (QC) 72
      2.7.1. Examples of SFC-MS analytics directly out of the destination plates I, II, III and IV 73
   2.8. Automated analysis of mass spectrometry data 97
      2.8.1. Preparation 97
      2.8.2. Processing mzXML files into spectra 97
      2.8.3. Prediction procedure 97
      2.8.4. Python code 97
   2.9. Heat maps 98
3. Statistical reaction analysis 100
4. Milligram scale reactions 134
   4.1. Synthetic procedures and analytical data 134
      4.1.1. GBB-3CR 134
      4.1.2. P-3CR 134
      4.1.3. U-4CR 135
      4.1.4. UT-4CR 136
      4.1.5. U-5C-3CR of proline and hydroxy acetaldehyde 136
      4.1.6. UH-4CR 137
      4.1.7. Intramolecular U-5C-3CR of α-amino acids with nucleophilic side chains 137
      4.1.8. vL-3CR 138
      4.1.9. U-5C-4CR with additional amine 139
      4.1.10. UPS-4CR 140
      4.1.11. SU-4CR 141
   4.2. ^{1}H and ^{13}C NMR spectra 142
5. Computational chemistry 165
   5.1. Dataset preparation 165
   5.2. Molecular descriptors 165
   5.3. Bioavailability filters 165
   5.4. Principal component analysis 165
   5.5. Normalized principal moments of inertia and cumulative distribution 165
   5.6. Similarity analysis 166
   5.7. t-distributed Stochastic Neighbor Embedding 166
   5.8. Statistical Analysis 166

References 179

1. General information

All reagents and solvents were purchased from commercial suppliers and used without any purification unless otherwise noted. All isocyanides were prepared in house by either performing the Ugi,[1-4] Hofmann[5,6] or our recently described Leukart-Wallach reductive amination procedure.[7]
Other reagents were purchased from Sigma Aldrich, ABCR, Acros, Fluorochem and AK Scientific and were used without further purification. Nuclear magnetic resonance spectra were recorded on a Bruker Avance 500 spectrometer ^{1}H NMR (500 MHz), ^{13}C NMR (126 MHz). Chemical shifts for ^{1}H NMR were reported as δ values, and coupling constants were in hertz (Hz); the following abbreviations were used for spin multiplicity: s = singlet, br s = broad singlet, d = doublet, t = triplet, q = quartet, dd = double of doublets, m = multiplet. Chemical shifts for ^{13}C NMR reported in ppm relative to the solvent peak. Analytical thin-layer chromatography was performed using precoated silica gel 60 F_{254} plates (Merck, Darmstadt), and the spots were visualized with UV light at 254 nm or alternatively by staining with potassium permanganate, or ninhydrin solutions. Column chromatography was carried out with silica gel 60 (0.040-0.063 mm, 230-400 mesh). Electrospray ionization mass spectra (ESI-MS) were recorded on a Waters Investigator Semi-prep 15 SFC-MS instrument. High-resolution mass spectra were recorded using an LTQ-Orbitrap-Velos Pro (Thermo Fisher Scientific) in ESI-positive mode at a resolution of 60000 at m/z 400.

2. Nano-scale automated chemistry

2.1. General materials

Stock solutions were prepared in glass flat bottom vials (Screening devices, Catalog#: 9920-812FBT, 2.0 mL (Topas) Plate) and they were kept at -20 °C.

Nanomole-scale chemistry was performed using Echo qualified 384-well polypropylene microplate (Labcyte, Catalog#: PP-0200, clear, flat bottom).

384-Well source and destination plates were sealed by a sealing tape (Thermo Scientific, Catalog#: 232701, polyolefin acrylate) and were kept at -20 °C.

2.2. Instrumentation

The Echo 555 liquid handler (Labcyte) was used in order to transfer nL droplets of starting materials from the 384-well source plate to the 384-well destination plate.

2.3. Structures of the building blocks

Amidines

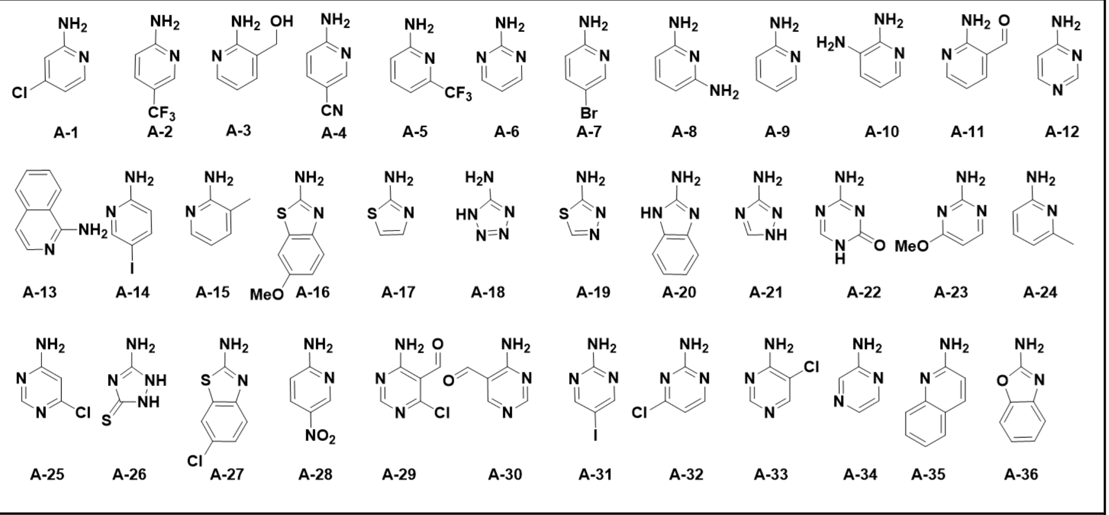

Aldehydes

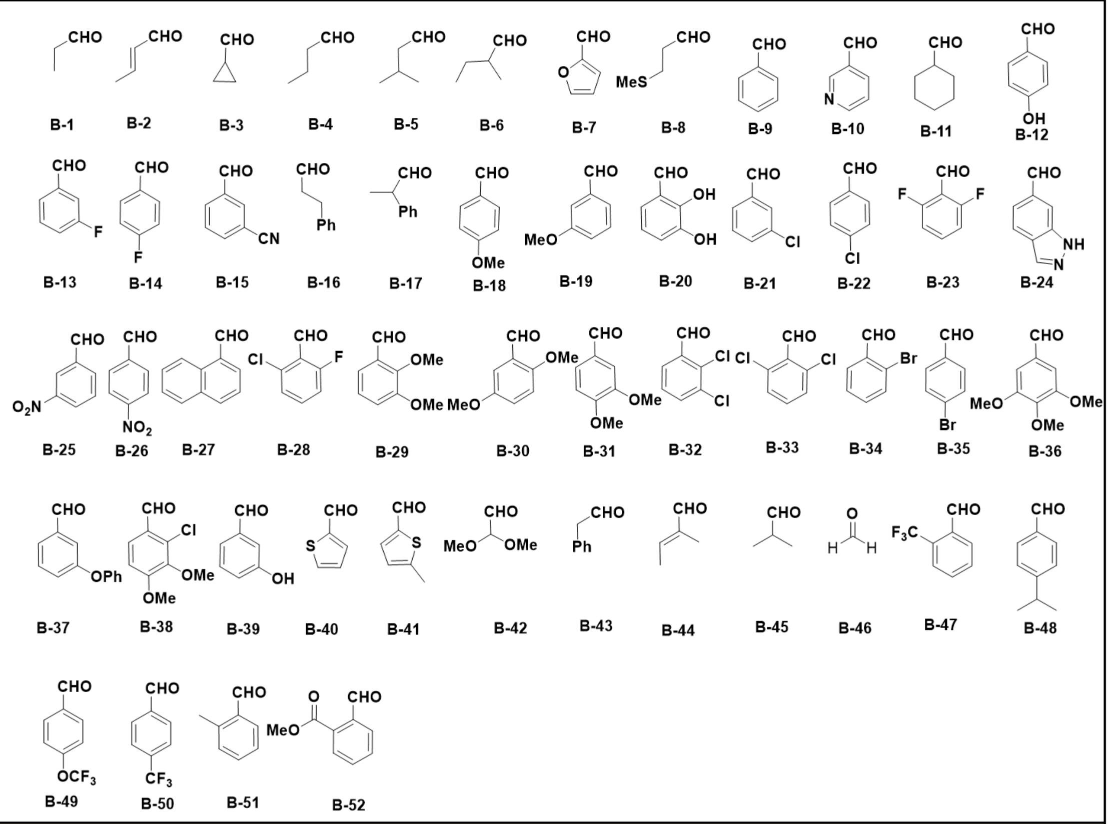

Ketones

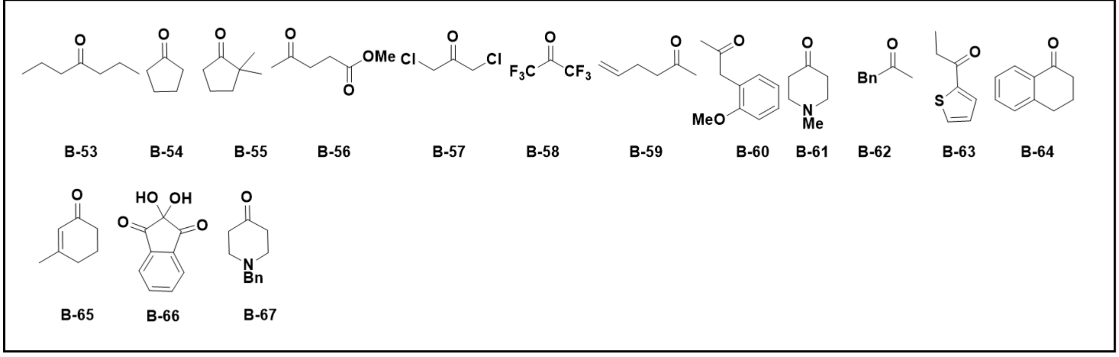

Isocyanides

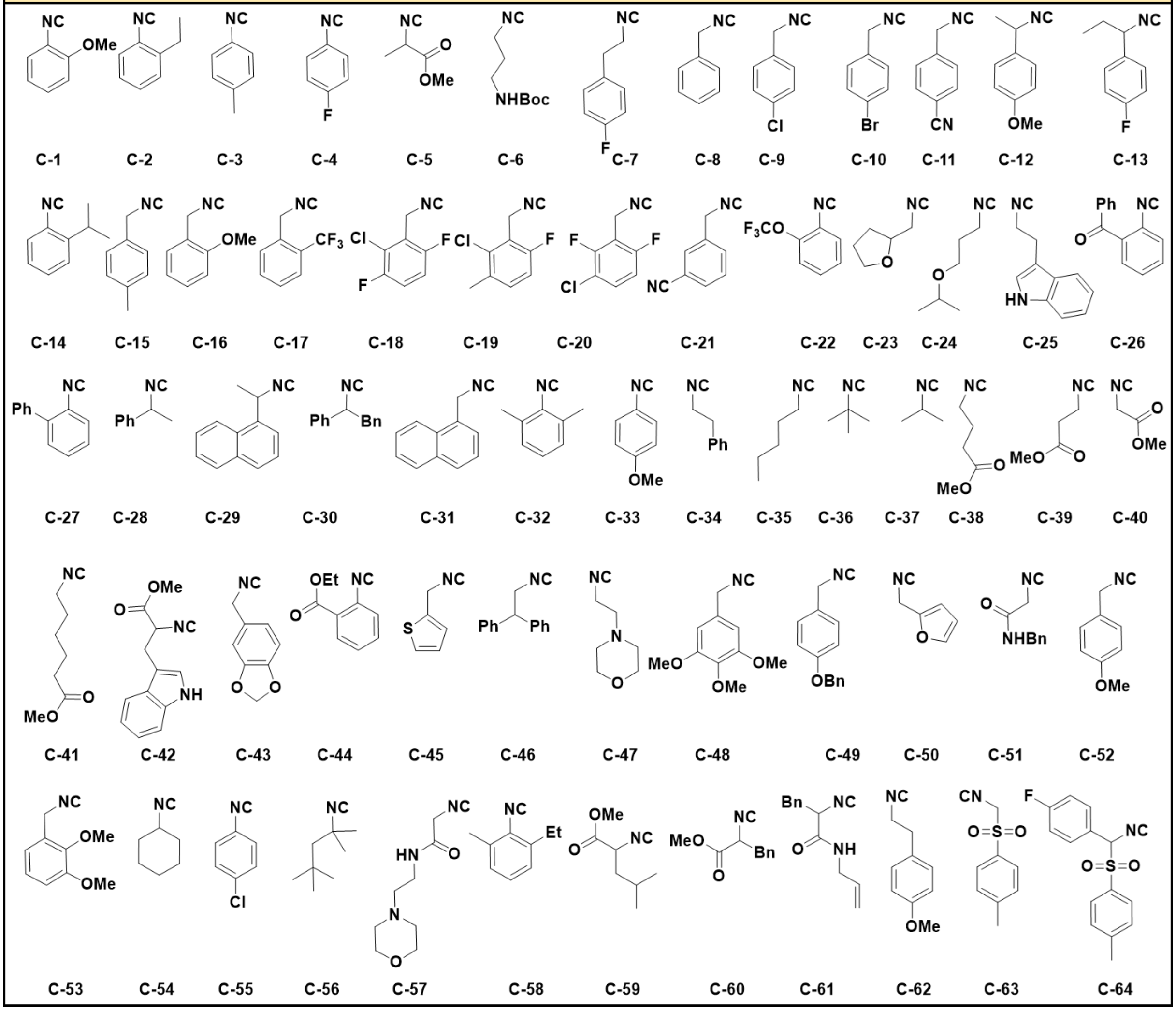

Carboxylic acids

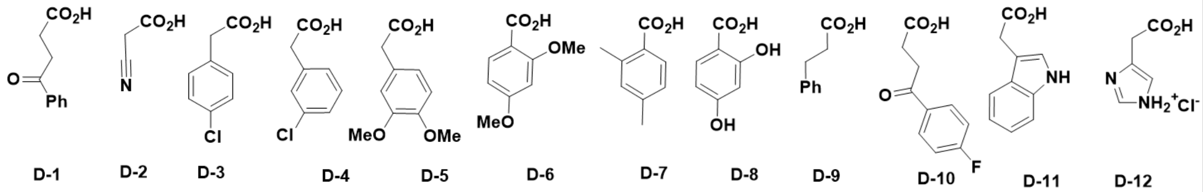

D-1 D-2 D-3 D-4 D-5 D-6 D-7 D-8 D-9 D-10 D-11 D-12

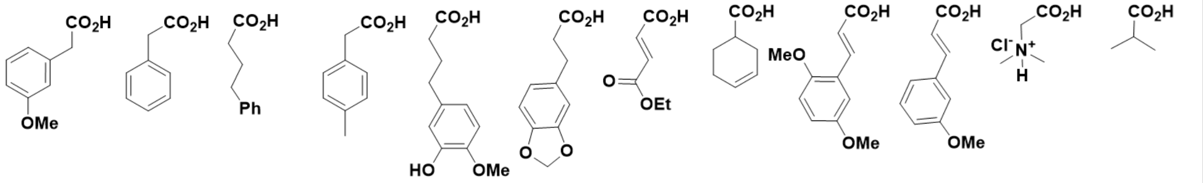

D-13 D-14 D-15 D-16 D-17 D-18 D-19 D-20 D-21 D-22 D-23 D-24

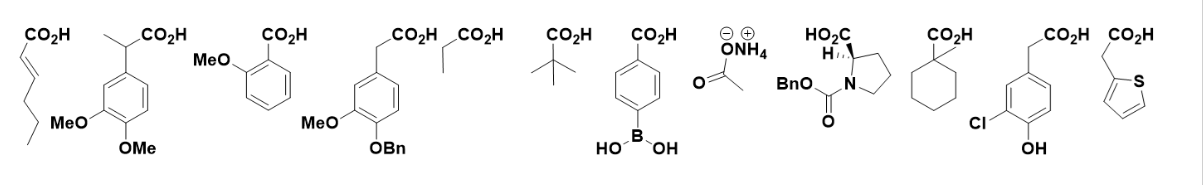

D-25 D-26 D-27 D-28 D-29 D-30 D-31 D-32 D-33 D-34 D-35 D-36

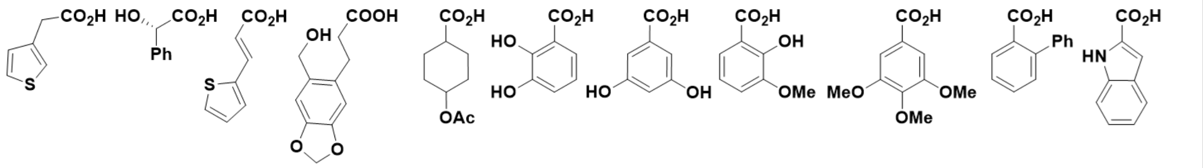

D-37 D-38 D-39 D-40 D-41 D-42 D-43 D-44 D-45 D-46 D-47

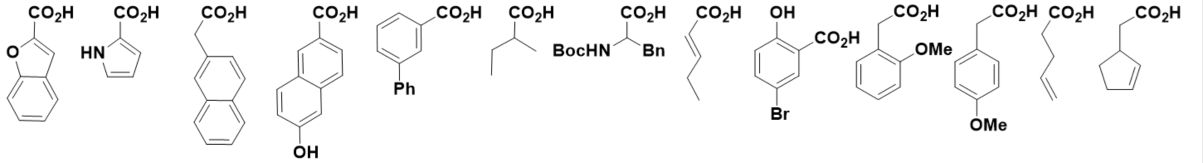

D-48 D-49 D-50 D-51 D-52 D-53 D-54 D-55 D-56 D-57 D-58 D-59 D-60

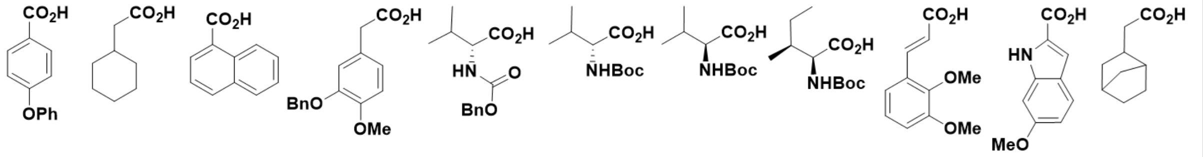

D-61 D-62 D-63 D-64 D-65 D-66 D-67 D-68 D-69 D-70 D-71

Primary amines

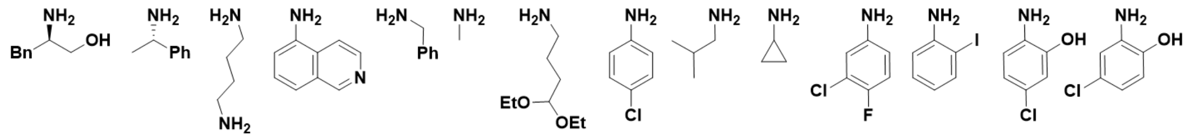

E-1 E-2 E-3 E-4 E-5 E-6 E-7 E-8 E-9 E-10 E-11 E-12 E-13 E-14

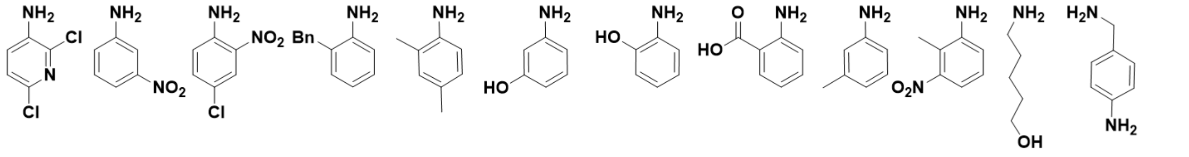

E-15 E-16 E-17 E-18 E-19 E-20 E-21 E-22 E-23 E-24 E-25 E-26

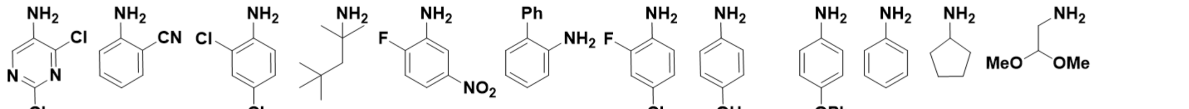

E-27 E-28 E-29 E-30 E-31 E-32 E-33 E-34 E-35 E-36 E-37 E-38

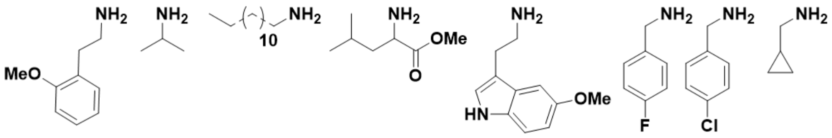

E-39 E-40 E-41 E-42 E-43 E-44 E-45 E-46

Secondary amines

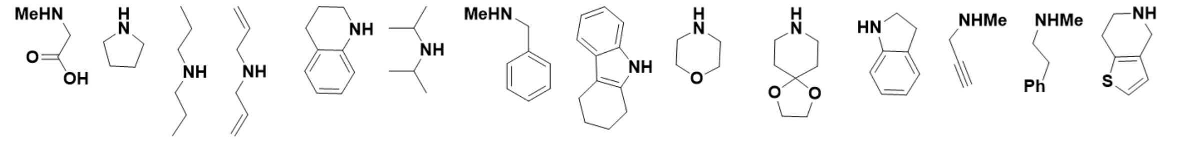

E-47 E-48 E-49 E-50 E-51 E-52 E-53 E-54 E-55 E-56 E-57 E-58 E-59 E-60

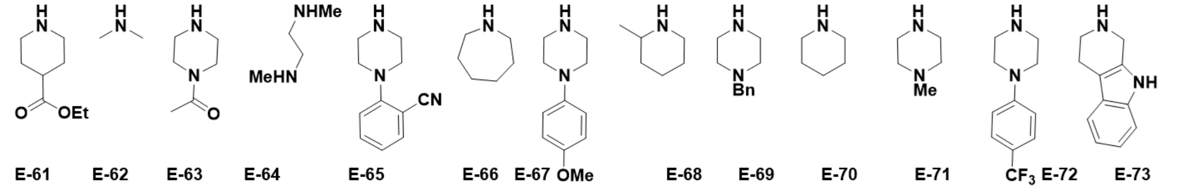

E-61 E-62 E-63 E-64 E-65 E-66 E-67 E-68 E-69 E-70 E-71 E-72 E-73

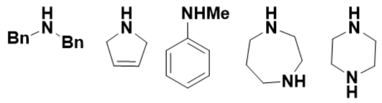

E-74 E-75 E-76 E-77 E-78

Hydroxyacetaldehyde dimer

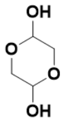

F-1

Tetrazole amines

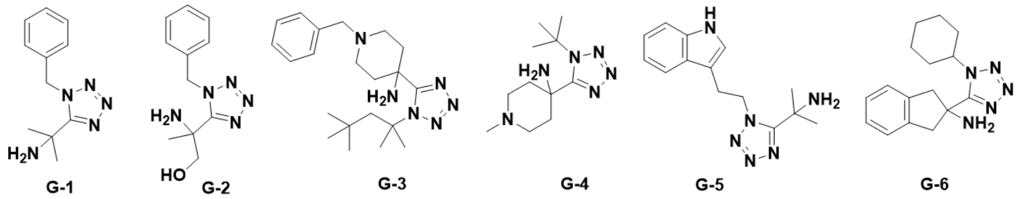

G-1 G-2 G-3 G-4 G-5 G-6

α-Amino acids with nucleophilic side chains

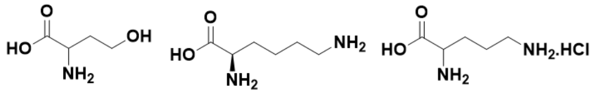

H-1 H-2 H-3

Prolines

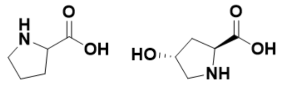

I-1 I-2

Dipeptides

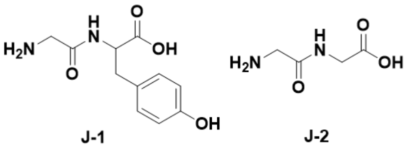

J-1 J-2

2.4. Stock solution preparation

Stock solutions of amidines (**A2-A22, A34-A36**) were prepared as 0.5 M in ethylene glycol. Due to the insolubility of some amidines in ethylene glycol, their stock solutions were instead prepared as 0.5 M: **A1** in ethylene glycol/2-methoxyethanol (1:2); **A23-A27** in 2-methoxyethanol. Due to the insolubility of some of the amidines in 0.5 M 2-methoxyethanol, their stock solutions were diluted to 0.25 M (**A28-A30**) and 0.16 M (**A31-A33**), respectively.

Stock solutions of aldehydes and ketones (**B1, B3-B13, B18, B19, B21, B23, B40, B41, B45-B51, B53-B61, B65**) were prepared as 0.5 M in ethylene glycol. Due to insolubility of some aldehydes and ketones in ethylene glycol, their stock solutions were instead prepared as 0.5 M: **B67** in ethylene glycol/2-methoxyethanol (4:1); **B2, B15-B17, B28, B34, B35, B37, B42, B64** in ethylene glycol/2-methoxyethanol (2:1); **B14, B27, B29-B32, B39, B43, B44, B63** in ethylene glycol/2-methoxyethanol (1:1); **B25, B36** in ethylene glycol/2-methoxyethanol (1:2); **B62** in ethylene glycol/2-methoxyethanol (2:3); **B20, B22, B24, B26, B33, B38, B52, B66** in 2-methoxyethanol.

Stock solutions of isocyanides (**C1-C9, C12-C15, C22-C25, C30-C45, C47, C50, C52, C54, C56, C57-C59, C62)** were prepared as 0.5 M in ethylene glycol. Due to the insolubility of some isocyanides in ethylene glycol, their stock solutions were instead prepared as 0.5 M: **(C10, C11, C16-C21, C26-C29, C46, C48, C49, C51, C53, C55, C60, C61, C64)** in 2-methoxyethanol; **C63** in ethylene glycol/2-methoxyethanol (1:2).

Stock solutions of carboxylic acids (**D1-D11, D13-D22, D24-D39, D41-D68, D71)** were prepared as 0.5 M in 2-methoxyethanol. Due to insolubility of some carboxylic acids (**D12, D23, D40, D69, D70**) in 0.5 M 2-methoxyethanol, their stock solutions were diluted to 0.25 M.

Stock solutions of primary amines (**E2, E3, E5-E11, E14, E18, E23, E29, E36, E37, E38, E40-E42, E44-E46**) were prepared as 0.5 M in ethylene glycol. Due to insolubility of some primary amines in ethylene glycol, their stock solutions were instead prepared as 0.5 M: **E19** in ethylene glycol/2-methoxyethanol (5:1); **E30, E33** in ethylene glycol/2-methoxyethanol (3:1); **E20, E25, E26, E28** in ethylene glycol/2-methoxyethanol (2:1); **E21, E31, E32, E34, E35, E39** in ethylene glycol/2-methoxyethanol (1:1); **E1, E4, E12, E13, E16, E17, E22, E24, E27** in ethylene glycol/2-methoxyethanol (1:2); **E15, E43** in 2-methoxyethanol.

Stock solutions of all secondary amines (**E47-E72, E74-E77**) were prepared as 0.5 M in ethylene glycol except **E73** which was prepared as 0.5 M in 2-methoxyethanol and **E78** which was prepared as 0.5 M in ethylene glycol/2-methoxyethanol (1:1).

Stock solution of hydroxylacetaldehyde dimer (**F1**) was prepared as 0.5 M in ethylene glycol.

Stock solutions of aminotetrazoles were prepared as 0.5 M: **G1, G2, G4, G6** in dimethoxyethane; **G3, G5** in ethylene glycol/dimethoxyethane (1:2).

Stock solutions of α-amino acids with nucleophilic side chains were prepared as 0.5 M: **H1,H2** in ethylene glycol; **H3** in water. In order to make a free base, to the stock solution of **H3**, 1 eq of K_2CO_3 was added.

Stock solutions of Prolines (**I1, I2**) were prepared as 0.5 M in water.

Stock solutions of dipeptides were prepared as 0.5 M: **J1** in ethylene glycol and **J2** in water.

Stock solutions of KNCO and Py.HCl were prepared as 1 M in water. Stock solutions of $Sc(OTf)_3$ and $TMSN_3$ were prepared as 0. 5 M in ethylene glycol.

2.5. Nano-scale synthesis

The stock solutions were dispensed to a 384-well source plate using Eppendorf multi-channel pipettes (50 µL per well). The Echo 555 was used to transfer the starting materials into the corresponding well in the destination plate.

Destination plate I

Groebcke-Blackburn-Bienaymé reaction (GBB-3CR) was performed in wells A1-D24 of destination plate I. For GBB-3CR, amidines (1 eq, 1000 nL), aldehydes (1 eq, 1000 nL), $Sc(OTf)_3$ (10 mol%, 100 nL) and isocyanides (1 eq, 1000 nL) were transferred into the corresponding well in the destination plate, respectively.[8] In case of diluted amidines with 0.25 M and 0.16 M concentration, 2000 nL and 3000 nL were transferred, respectively.

Passerini reaction (P-3CR) was performed in wells E1-H24 of destination plate I. For P-3CR, oxo components (1 eq, 1000 nL), carboxylic acids (1 eq, 1000 nL), and isocyanides (1 eq, 1000 nL) were transferred into the corresponding well in the destination plate, respectively.[9] In case of diluted carboxylic acids with 0.25 M concentration, 2000 nL were transferred.

Ugi-4CR reaction (U-4CR) was performed in wells I1-L24 of destination plate I. For U-4CR, primary amines (1 eq, 750 nL), oxo components (1 eq, 750 nL), carboxylic acids (1 eq, 750 nL) and isocyanides (1 eq, 750 nL) were transferred into the corresponding well in the destination plate, respectively.[10] In case of diluted carboxylic acids with 0.25 M concentration, 1500 nL was transferred. In wells K1-L24, formaldehyde was used as oxo component.

Ugi-tetrazole reaction (UT-4CR) was performed in wells M1-P24 of destination plate I. For UT-4CR, amines (1 eq, 750 nL), oxo components (1 eq, 750 nL), isocyanides (1 eq, 750 nL) and $TMSN_3$ (1 eq, 750 nL) were transferred into the corresponding well in the destination plate, respectively.[11]

Once the starting material transfer to destination plate I, using Echo 555 was completed (~150 min), 10 µL of MeOH using a multichannel pipette was added to GBB-3CR, Ugi-4CR, UT-4CR (wells A1-D24, I1-P24) and 10 µL $CHCl_3$ was added to Passerini-3CR (wells E1-H24). The destination plate was sealed and was then placed for 12 h at 21 °C on an orbital shaker. Then, it was desealed to let the MeOH and $CHCl_3$ evaporated. After ~3 h evaporation time, destination plate I was sealed and was kept at -20 °C.

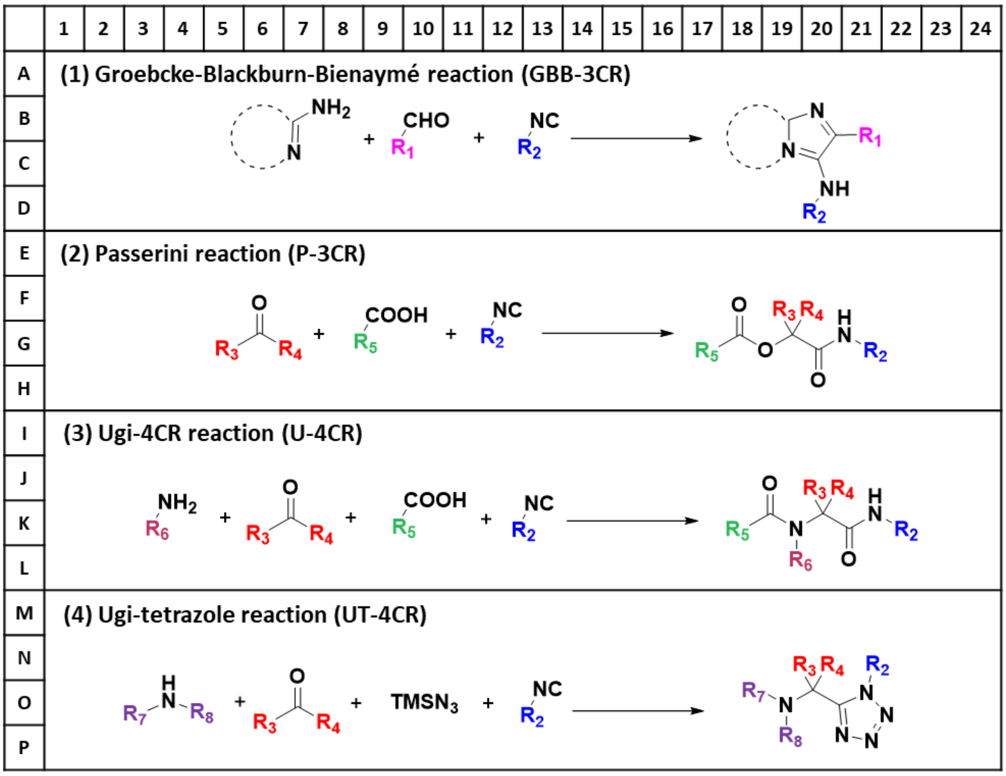

Fig. S1. Reactions in destination plate I.

Destination plate II

Intramolecular Ugi reaction with dipeptides was performed in wells A1-D24 of destination plate II. Dipeptides (1 eq, 1000 nL), oxo components (1 eq, 1000 nL) and isocyanides (1 eq, 1000 nL) were transferred into the corresponding well in the destination plate, respectively.[12]

Intramolecular Ugi-type reaction of proline and hydroxyacetaldehyde (U-5C-3CR) was performed in wells E1-H24 of destination plate II. Prolines (1 eq, 1000 nL), hydroxyacetaldehyde (1 eq, 1000 nL) and isocyanides (1 eq, 1000 nL) were transferred into the corresponding well in the destination plate, respectively.[13]

Ugi-hydantoin reaction (UH-4CR) was performed in wells I1-L24 of destination plate II. 600 nL of each starting material (primary amine, oxo component, KNCO, Py.HCl and isocyanide) was transferred into the corresponding well in the destination plate.[14]

Intramolecular Ugi-type reaction of α-amino acids with nucleophilic side chains (U-5C-3CR) was performed in wells M1-P24 of destination plate II. α-Amino acids with nucleophilic side chains (1 eq,

1000 nL), oxo components (1 eq, 1000 nL) and isocyanides (1 eq, 1000 nL) were transferred into the corresponding well in the destination plate, respectively.[15]

Once the starting material transfer to destination plate II, using Echo 555 was completed (~150 min), 10 µL of MeOH was added to UH-4CR (wells I1-L24) and to the rest of the plate 10 µL trifluoroethanol using a multichannel pipette was added. The destination plate was sealed and was then placed for 12 h at 21 °C on an orbital shaker. Then, it was desealed to let the MeOH and TFE evaporated. After ~5 h evaporation time, destination plate II was sealed and was kept at -20 °C.

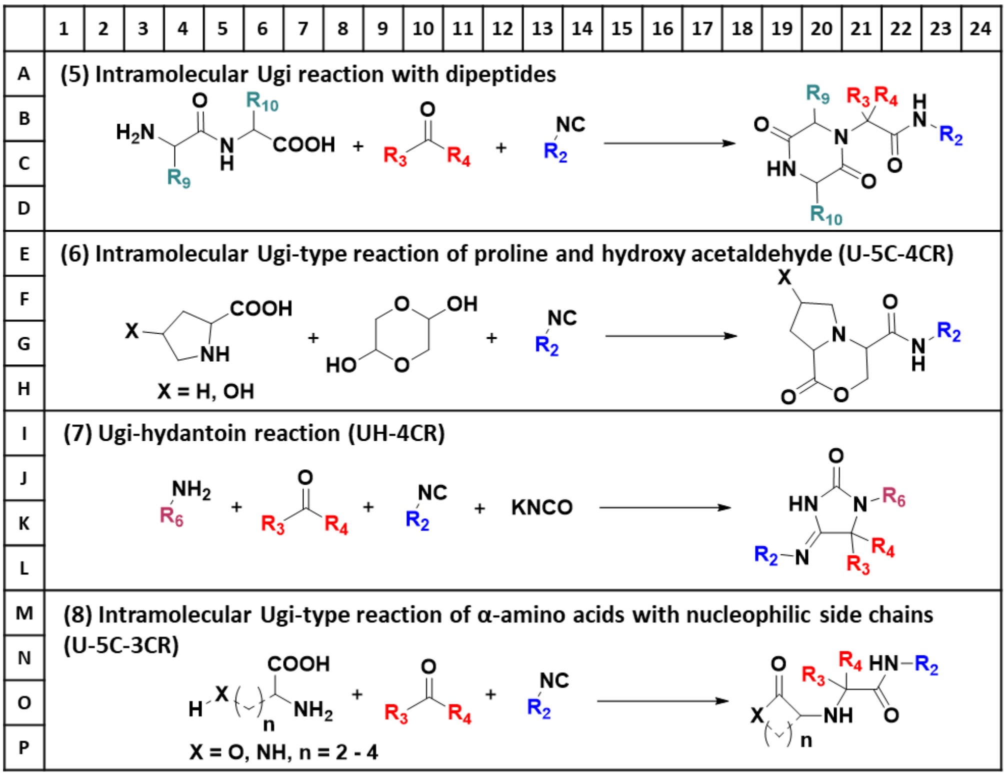

Fig. S2. Reactions in destination plate II.

Destination plate III

van Leusen reaction (vL-3CR) was performed in wells A1-D24 of destination plate III. Aldehydes (1 eq, 750 nL), primary amines (1 eq, 750 nL), tosmic isocyanides (1 eq, 750 nL) and piperazine (1.5 eq, 1125 nL) were transferred into the corresponding well in the destination plate, respectively.[16]

Ugi-hydantoin reaction involving tetrazole amines (UH-4CR) was performed in wells E1-H24 of destination plate II. 600 nL of each starting material (tetrazole amine, oxo component, isocyanide, KNCO and Py.HCl) was transferred into the corresponding well in the destination plate.[17]

Orru reaction (OR-3CR) was performed in wells I1-L24 of destination plate III. Aldehydes (1 eq, 1000 nL), primary amines (1 eq, 1000 nL) and α-acidic isocyanides (1 eq, 1000 nL) were transferred into the corresponding well in the destination plate, respectively.[18]

Intramolecular Ugi-tetrazole reaction using methyl 2-formylbenzoate (iUT-4CR) was performed in wells M1-P24 of destination plate III. Primary amines (1 eq, 750 nL), methyl 2-formylbenzoate (1 eq, 750 nL), isocyanides (1 eq, 750 nL) and $TMSN_3$ (1 eq, 750 nL) were transferred into the corresponding well in the destination plate, respectively.[19]

Once the starting material transfer to destination plate III, using Echo 555 was completed (~150 min), 10 µL of dichloromethane was added to OR-3CR (wells I1-L24) and to the rest of the plate 10 µL

MeOH using a multichannel pipette was added. The destination plate was sealed and was then placed for 12 h at 21 °C on an orbital shaker. Then, it was desealed to let the MeOH and dichloromethane evaporated. After ~3 h evaporation time, destination plate III was sealed and was kept at -20 °C.

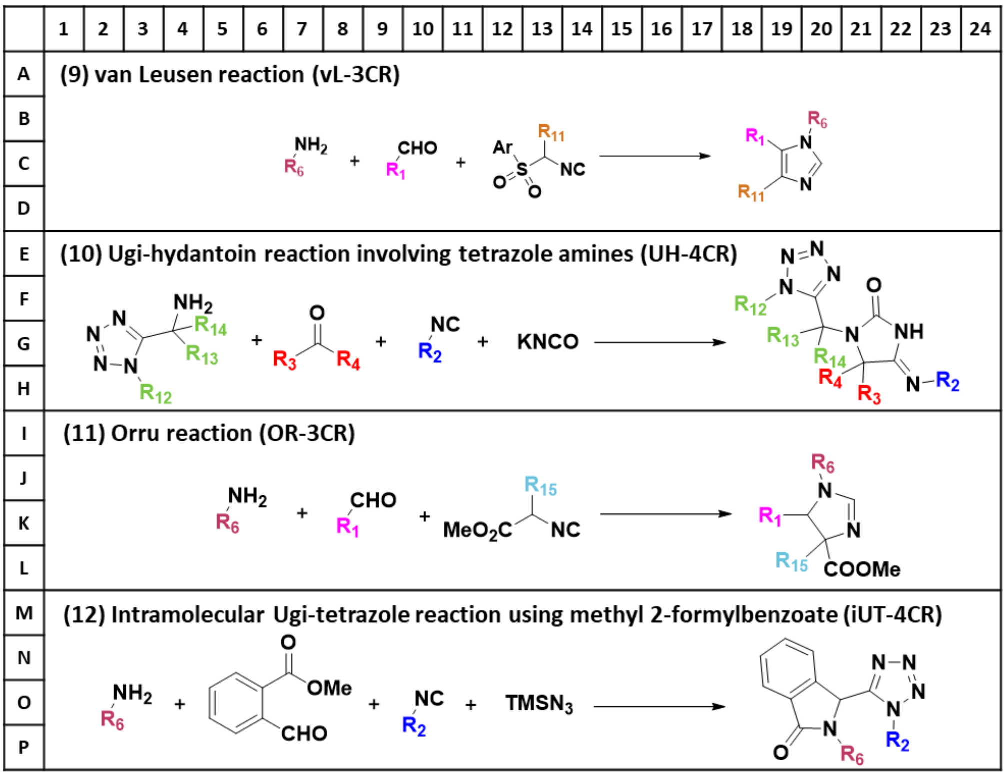

Fig. S3. Reactions in destination plate III.

Destination plate IV

U-5C-4CR reaction with additional amine was performed in wells A1-D24 of destination plate IV. Proline (1 eq, 750 nL), oxo component (1 eq, 750 nL), isocyanide (1 eq, 750 nL) and amine (1 eq, 750 nL) were transferred into the corresponding well in the destination plate, respectively.[20]

Ugi-Pictet-Spengler sequence (UPS-4CR) was performed in wells E1-H24 of destination plate IV. Aldehydes (1 eq, 750 nL), aminoacetaldehyde dimethyl acetal (1 eq, 750 nL), carboxylic acids (1 eq, 750 nL) and hetero/phenylethyl isocyanide (1 eq, 750 nL) were transferred into the corresponding well in the destination plate, respectively.[21]

Split-Ugi reaction (SU-4CR) was performed in wells I1-L24 of destination plate IV. Bis-secondary amines (1 eq, 750 nL), oxo xomponents (1 eq, 750 nL), carboxylic acids (1 eq, 750 nL) and isocyanide (1 eq, 750 nL) were transferred into the corresponding well in the destination plate, respectively.[22]

Intramolecular Ugi-tetrazole reaction using methyl isocyanoacetates (iUT-4CR) was performed in wells M1-P24 of destination plate IV. Aldehydes (1 eq, 750 nL), primary aliphatic and benzylic amines (1 eq, 750 nL), α-aminoacid derived isocyanides (1 eq, 750 nL) and $TMSN_3$ (1 eq, 750 nL) were transferred into the corresponding well in the destination plate, respectively.[23]

Once the starting material transfer to the destination plate IV, using Echo 555 was completed (~150 min), 10 µL of MeOH was added to the U-5C-4CR reaction with additional amine and the Ugi-Pictet-Spengler sequence (UPS-4CR) (wells A1-H24) and to the rest of the plate 10 µL ethylene glycol using

a multichannel pipette was added. The destination plate was sealed and was then placed for 12 h at 21 °C on an orbital shaker. Then, it was desealed to let the MeOH evaporated. After ~3 h evaporation time, 10 µL of formic acid was added to UPS-4CR (wells E1-H24), the destination plate IV was sealed, and kept at 60 °C for 2 h. Then it was desealed to let the formic acid evaporated, re-sealed and was kept at -20 °C.

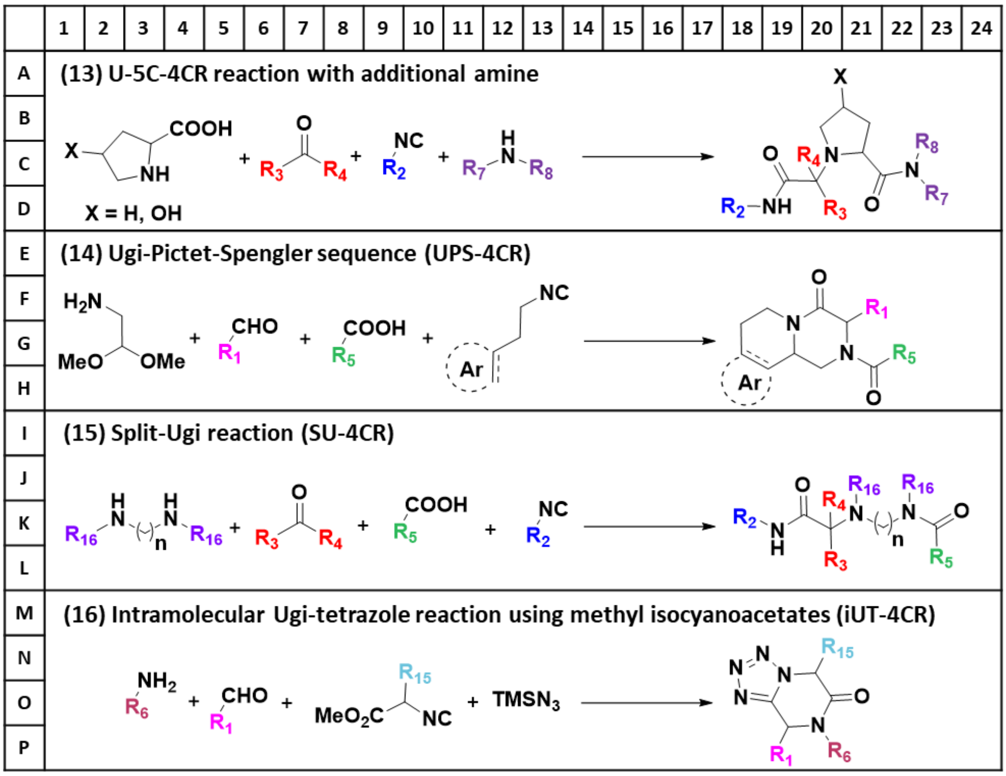

Fig. S4. Reactions in destination plate IV.

2.6. Pick list preparation

Labcyte Echo plate reformat software using custom mapping mode with the run protocol as defined by a pick list was used (Fig. S5A).

In order to generate a random library of products (N=96), a modified version of our previously reported program RandReactor was used.[24] The smiles files of the starting materials with the corresponding location in the source plate and mrv file of reaction were the input of the RandReactor program. The smiles file of the randomly generated products with their corresponding locations in the source and destination plate were the output of the RandReactor program. The smiles file was converted to a csv file which was the required format for Labcyte Echo plate reformat software (Fig. S5B). The structures of the products are shown in Fig. S6.

A

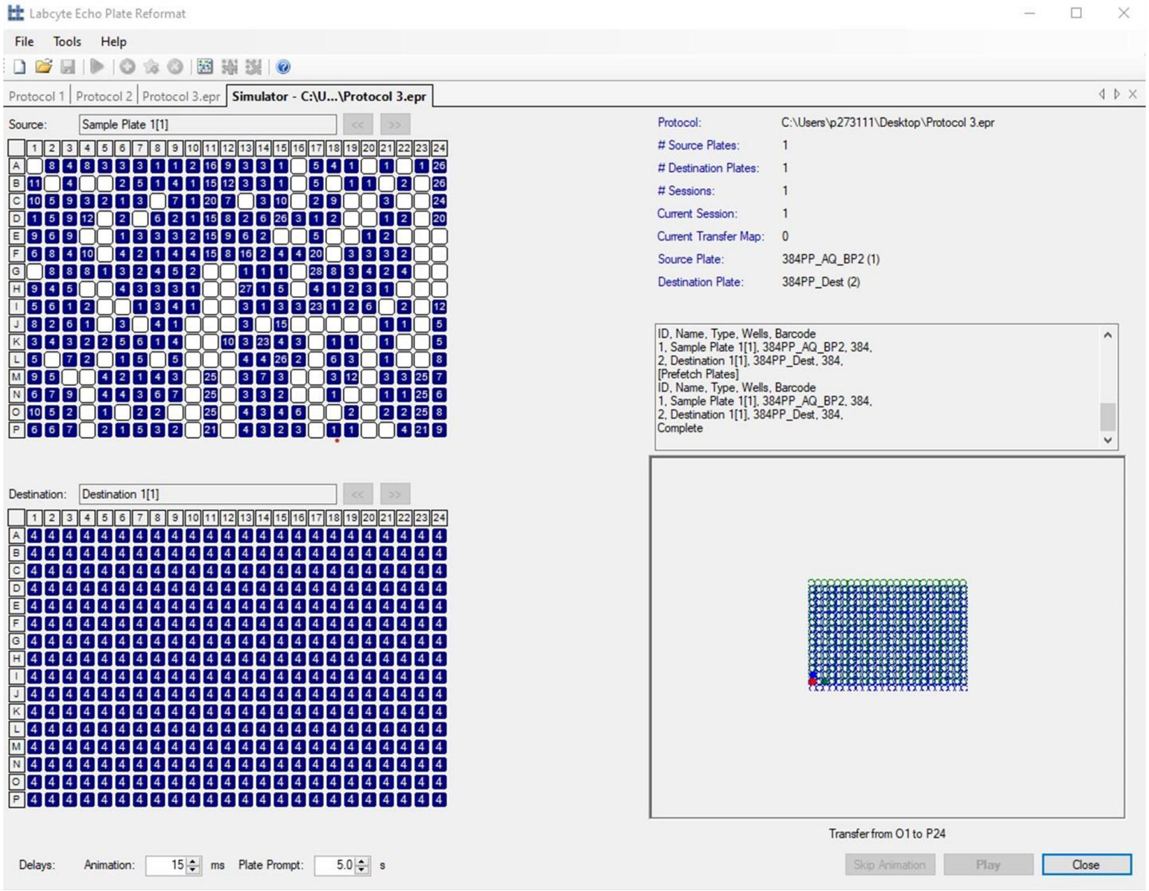

B

	A	B	C	D	E	F	G	H	I	J
1	Source Plate Name	Source Well	Destination Plate Name	Destination well	Transfer Volume					
2	Sample Plate 1	A24	Destination 1	A1	750					
3	Sample Plate 1	P1	Destination 1	A1	750					
4	Sample Plate 1	L13	Destination 1	A1	750					
5	Sample Plate 1	N22	Destination 1	A1	750					
6	Sample Plate 1	B24	Destination 1	A2	750					
7	Sample Plate 1	F3	Destination 1	A2	750					
8	Sample Plate 1	M14	Destination 1	A2	750					
9	Sample Plate 1	M22	Destination 1	A2	750					
10	Sample Plate 1	C24	Destination 1	A3	750					
11	Sample Plate 1	F12	Destination 1	A3	750					
12	Sample Plate 1	O16	Destination 1	A3	750					
13	Sample Plate 1	M18	Destination 1	A3	750					
14	Sample Plate 1	C24	Destination 1	A4	750					
15	Sample Plate 1	L1	Destination 1	A4	750					
16	Sample Plate 1	J13	Destination 1	A4	750					
17	Sample Plate 1	G22	Destination 1	A4	750					
18	Sample Plate 1	C24	Destination 1	A5	750					
19	Sample Plate 1	L3	Destination 1	A5	750					
20	Sample Plate 1	M14	Destination 1	A5	750					
21	Sample Plate 1	H19	Destination 1	A5	750					
22	Sample Plate 1	B24	Destination 1	A6	750					
23	Sample Plate 1	B1	Destination 1	A6	750					
24	Sample Plate 1	M15	Destination 1	A6	750					
25	Sample Plate 1	D22	Destination 1	A6	750					
26	Sample Plate 1	C24	Destination 1	A7	750					
27	Sample Plate 1	H3	Destination 1	A7	750					
28	Sample Plate 1	F17	Destination 1	A7	750					
29	Sample Plate 1	K19	Destination 1	A7	750					
30	Sample Plate 1	C24	Destination 1	A8	750					

Fig. S5. (**A**) Labcyte Echo plate reformat software, showing on top the source plate and below the destination plate IV; (**B**) Picklist in csv format required for Labcyte Echo plate reformat software.

Destination plate I

	A	B	C	D
**1**	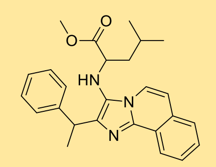	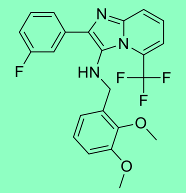	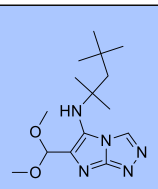	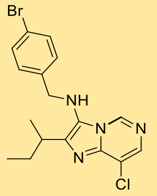
**2**	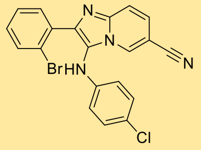	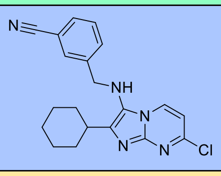	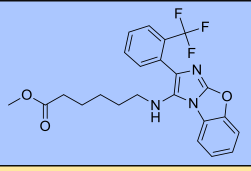	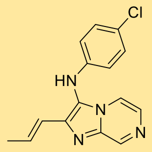
**3**	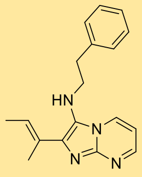	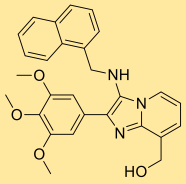	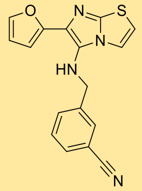	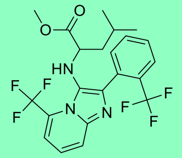
**4**	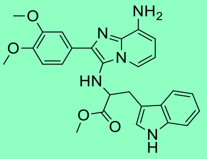	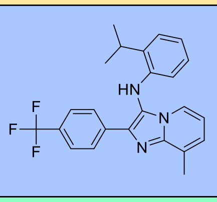	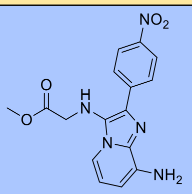	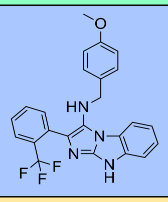
**5**	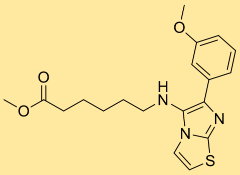	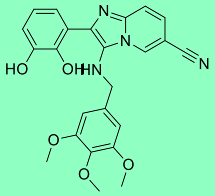	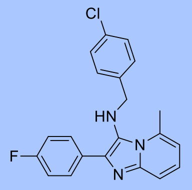	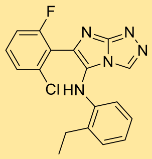
**6**	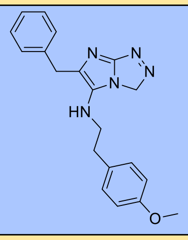	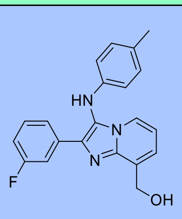	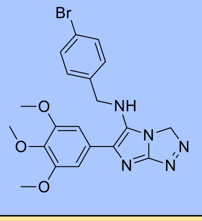	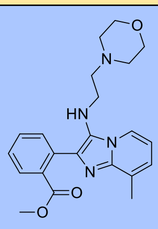
**7**	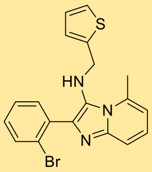	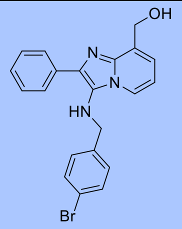	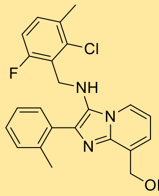	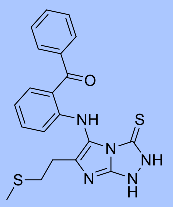
**8**	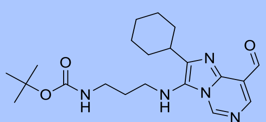	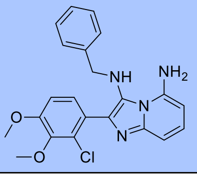	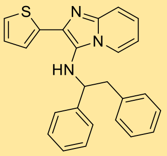	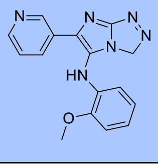

**9**	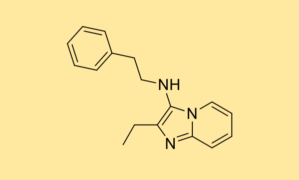	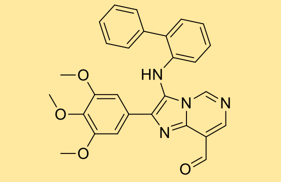	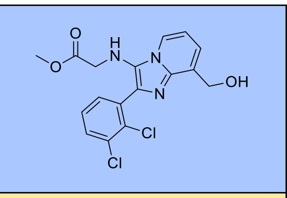	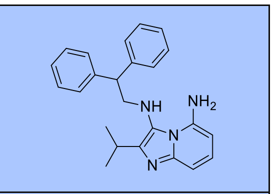
**10**	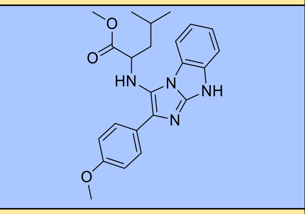	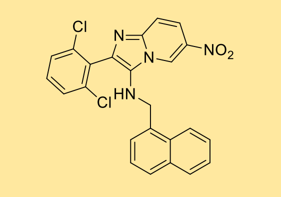	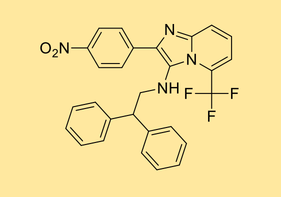	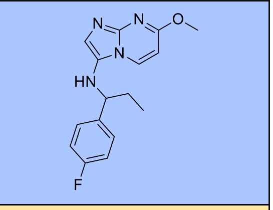
**11**	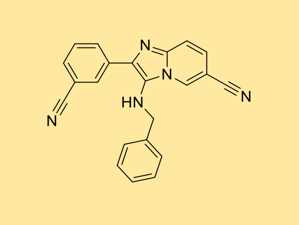	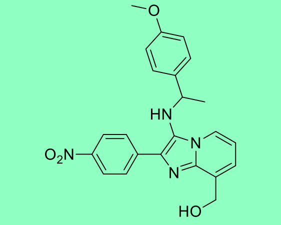	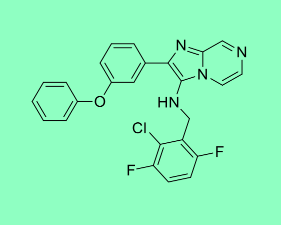	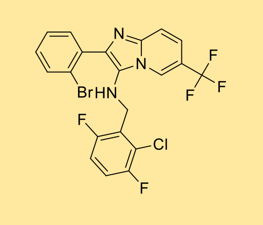
**12**	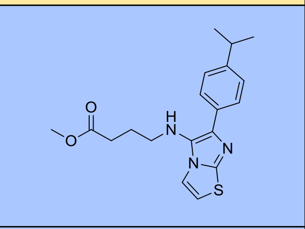	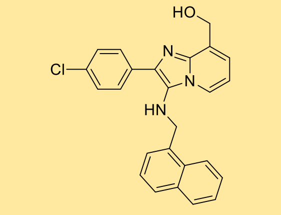	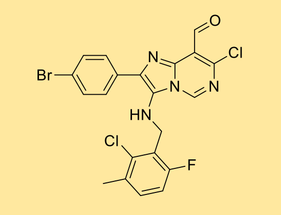	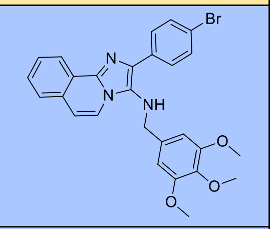
**13**	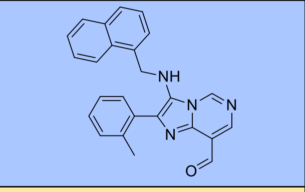	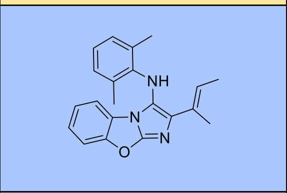	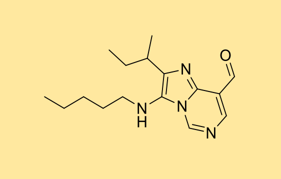	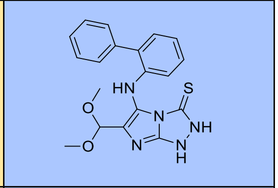
**14**	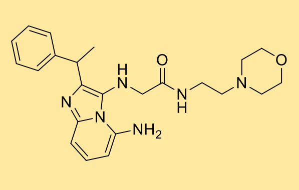	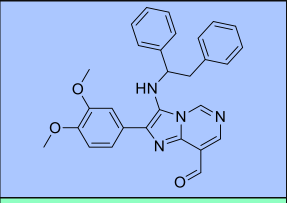	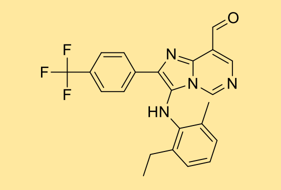	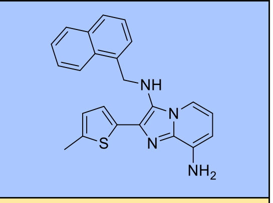
**15**	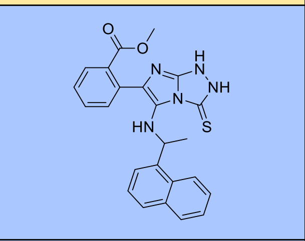	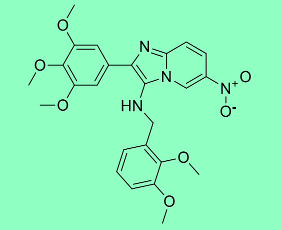	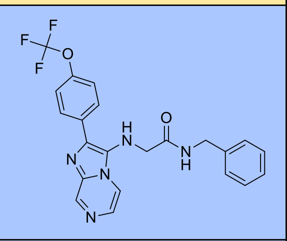	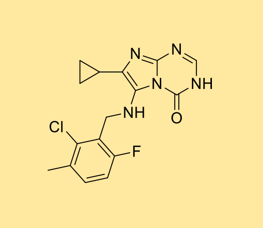
**16**	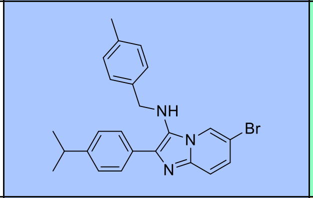	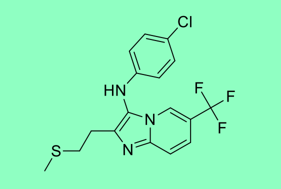	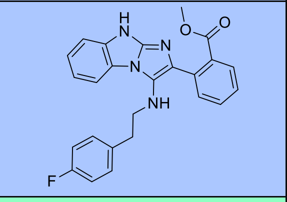	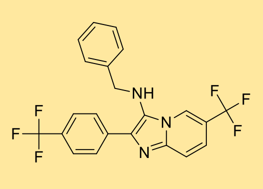
**17**	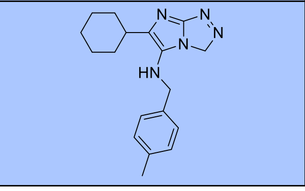	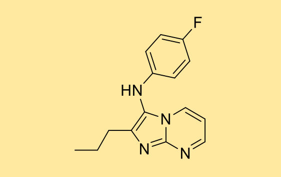	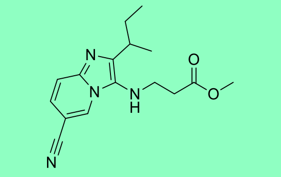	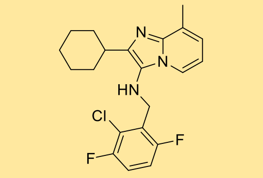

**18**	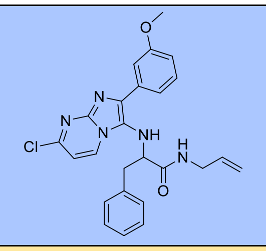	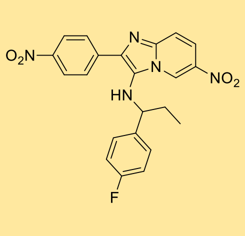	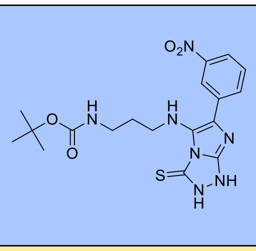	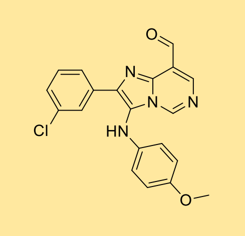
**19**	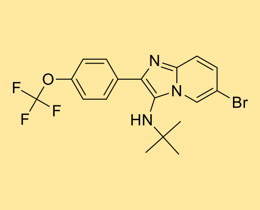	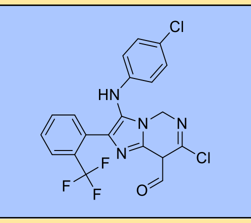	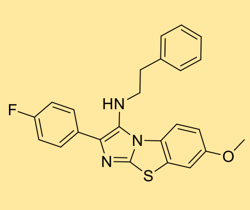	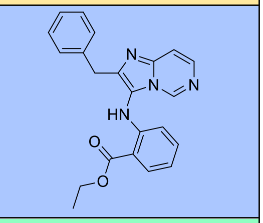
**20**	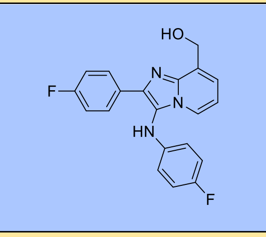	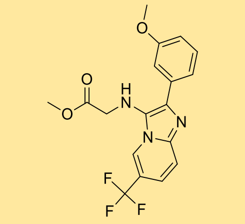	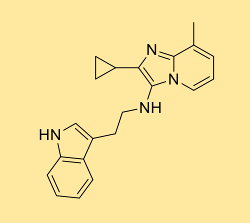	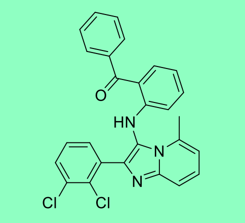
**21**	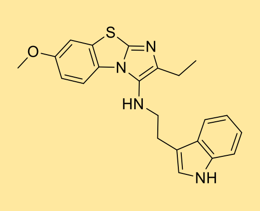	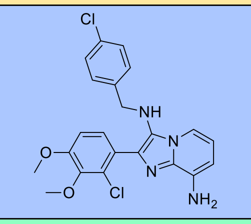	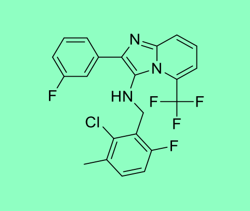	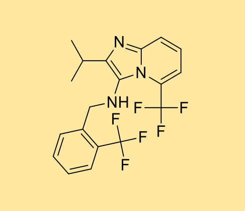
**22**	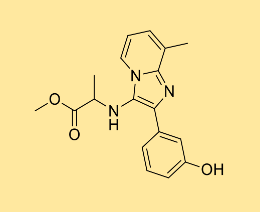	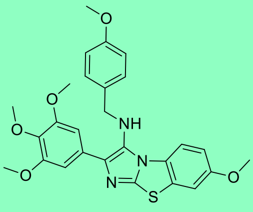	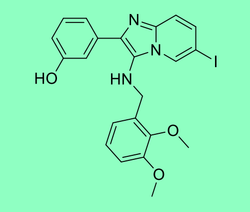	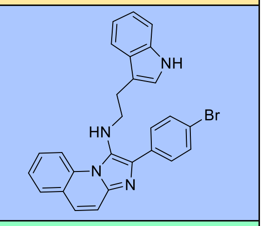
**23**	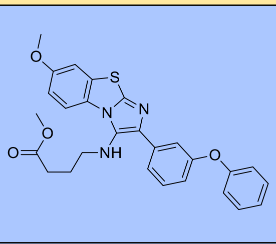	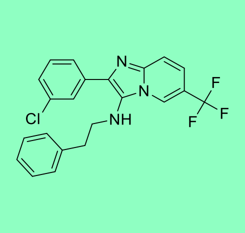	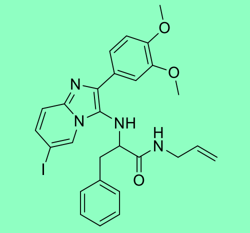	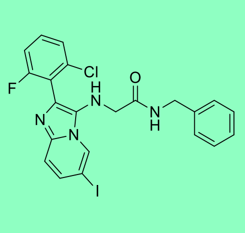
**24**	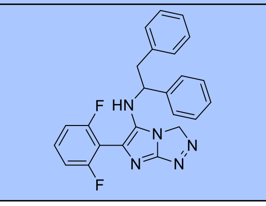	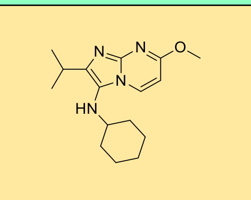	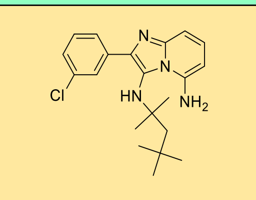	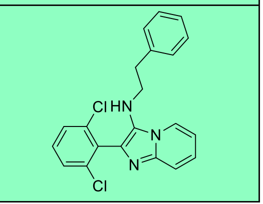

	E	F	G	H
**1**	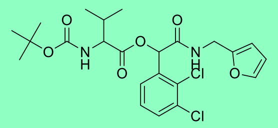	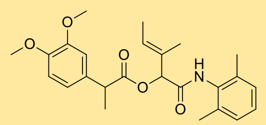	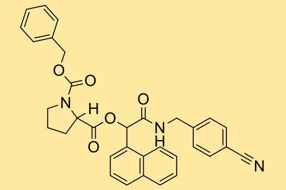	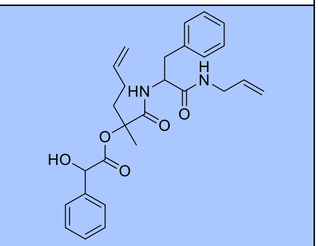
**2**	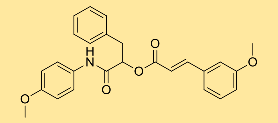	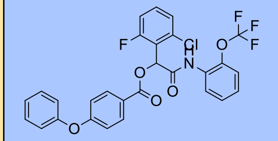	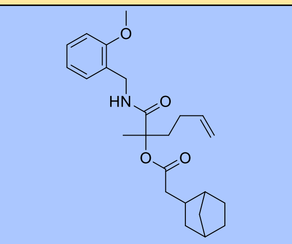	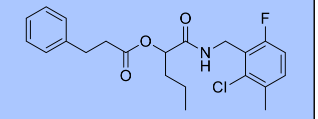
**3**	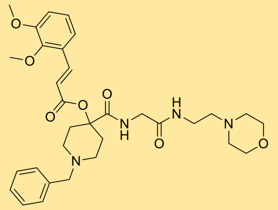	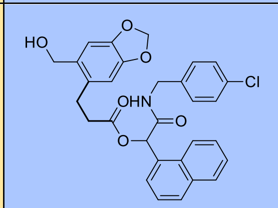	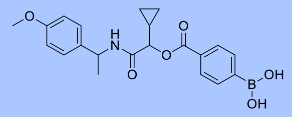	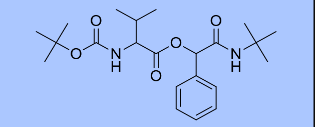
**4**	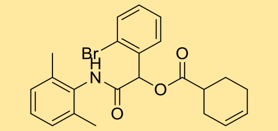	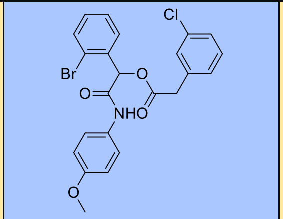	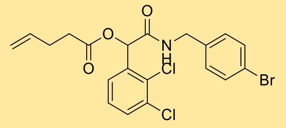	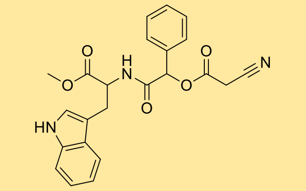
**5**	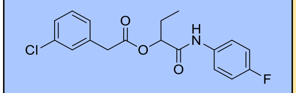	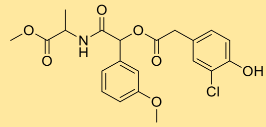	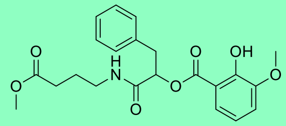	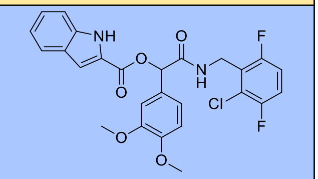
**6**	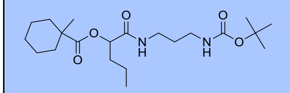	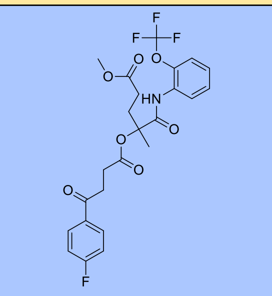	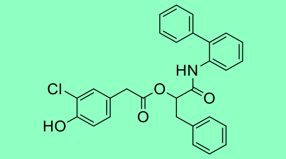	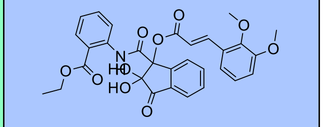
**7**	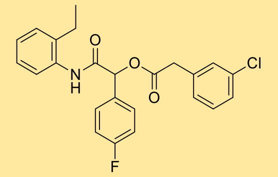	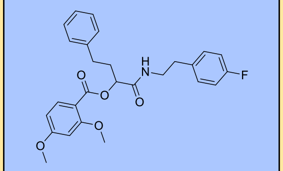	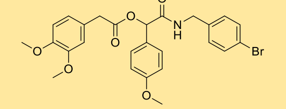	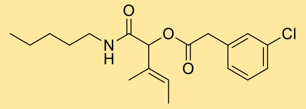

**8**	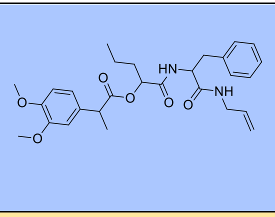	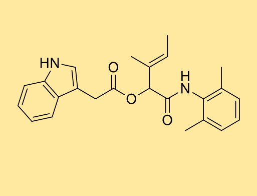	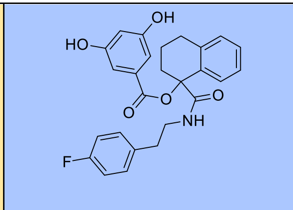	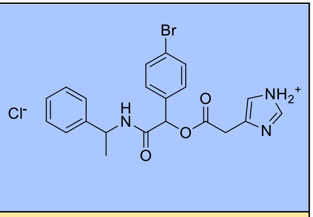
**9**	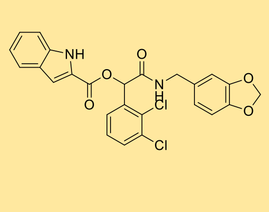	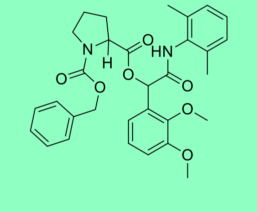	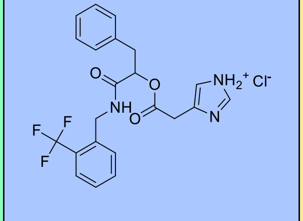	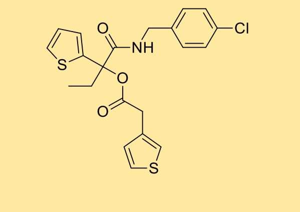
**10**	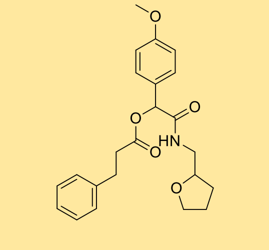	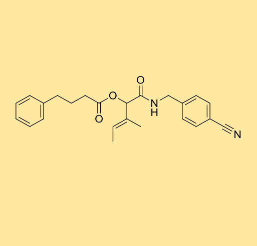	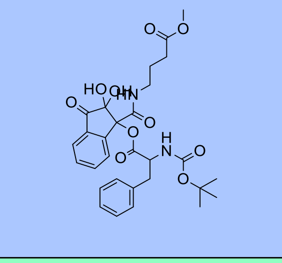	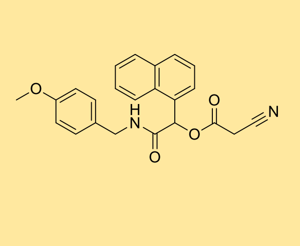
**11**	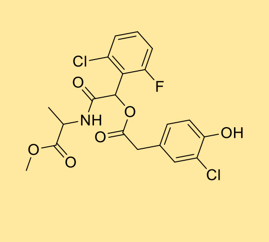	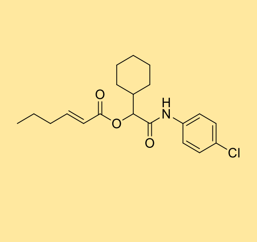	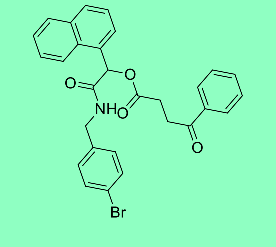	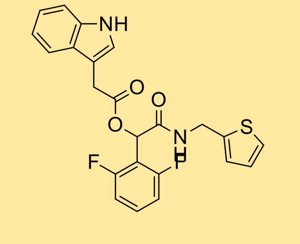
**12**	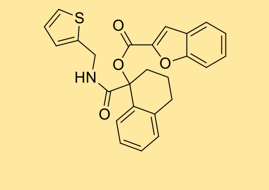	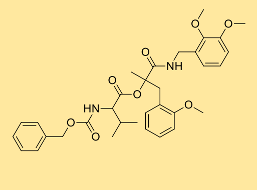	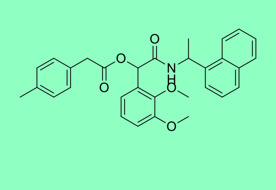	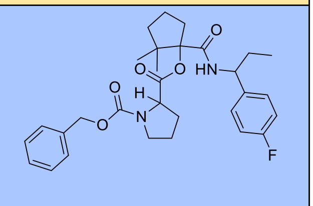
**13**	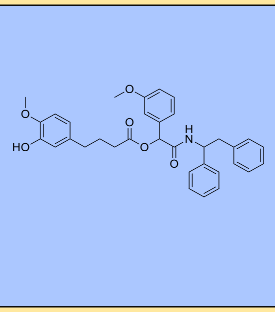	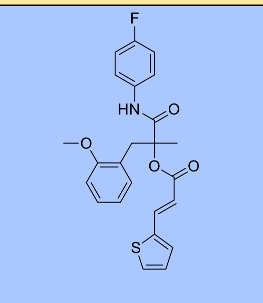	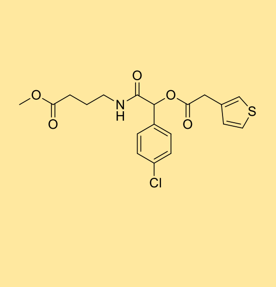	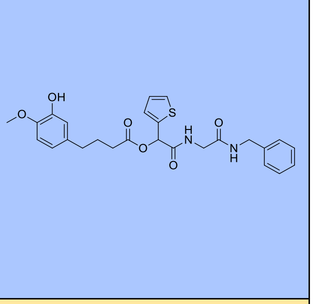
**14**	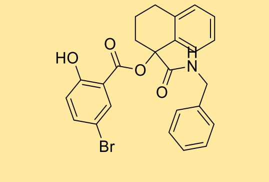	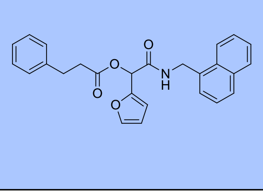	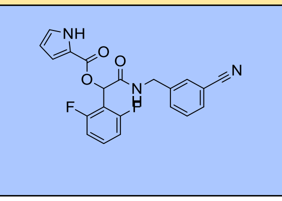	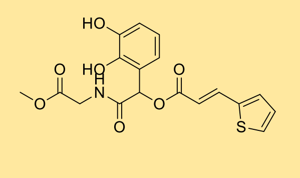

**15**	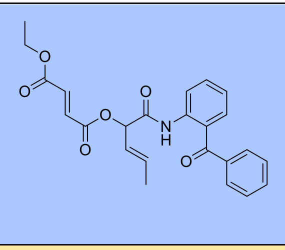	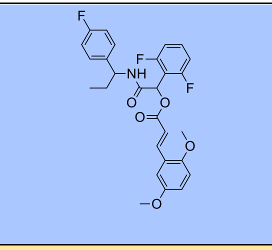	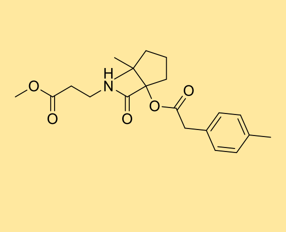	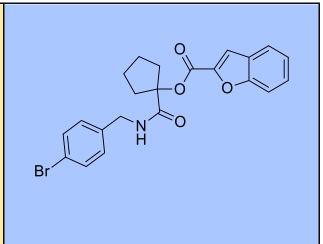
**16**	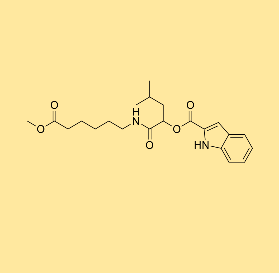	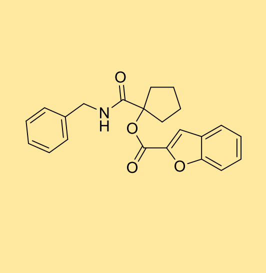	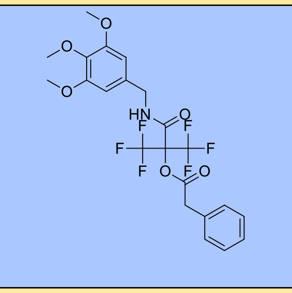	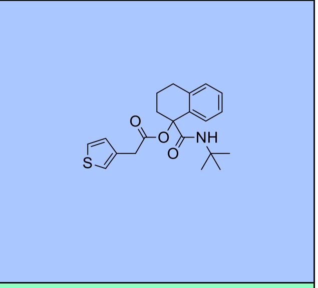
**17**	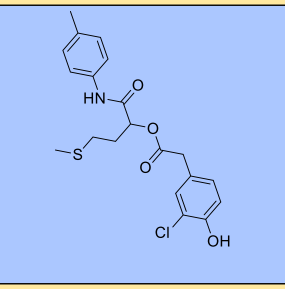	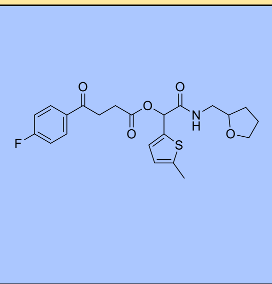	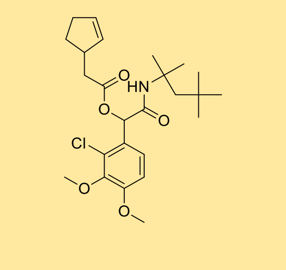	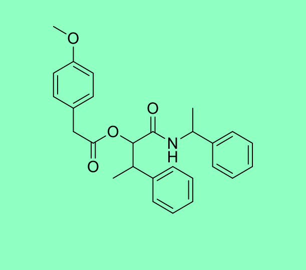
**18**	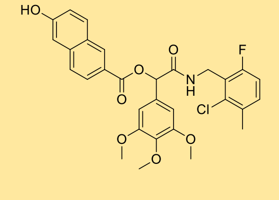	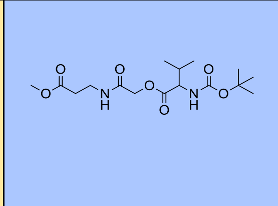	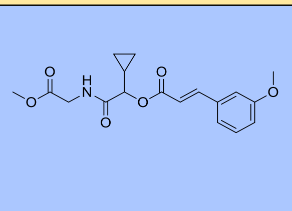	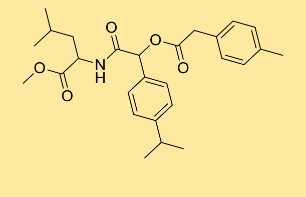
**19**	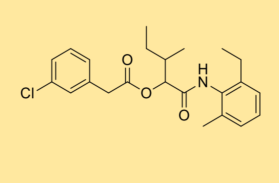	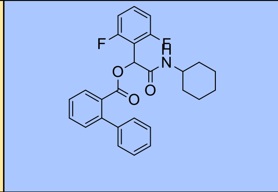	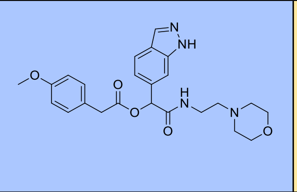	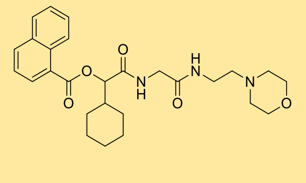
**20**	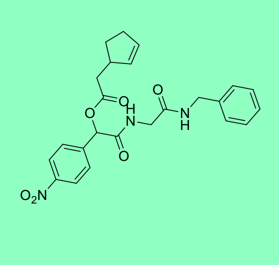	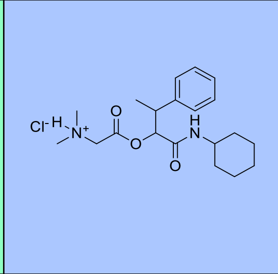	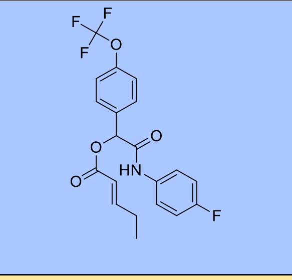	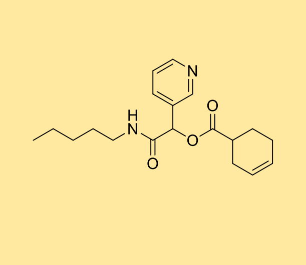
**21**	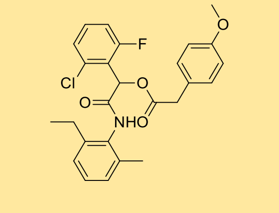	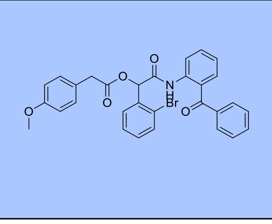	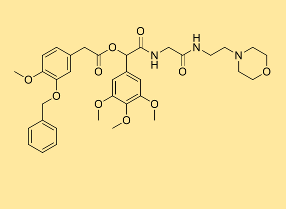	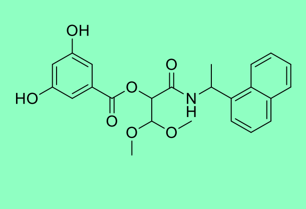

**22**	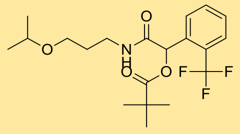	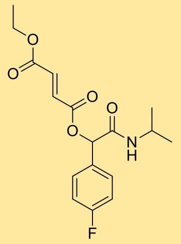	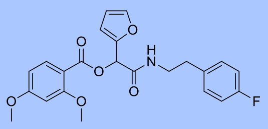	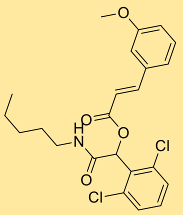
**23**	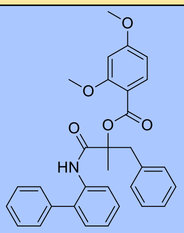	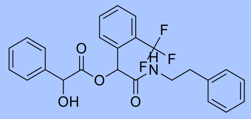	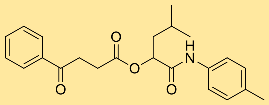	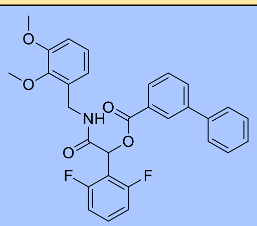
**24**	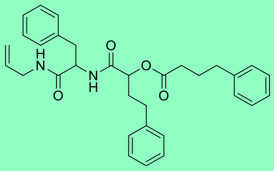	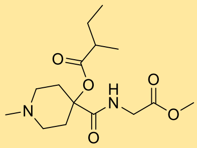	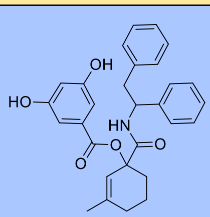	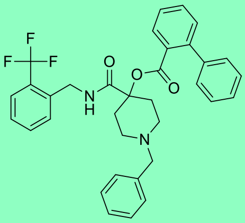

	I	J	K	L
**1**	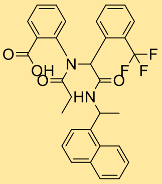	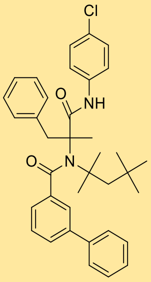	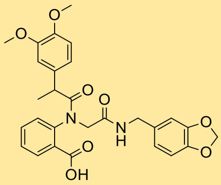	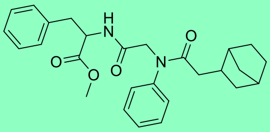
**2**	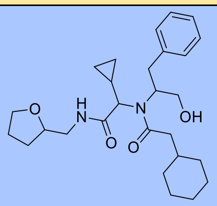	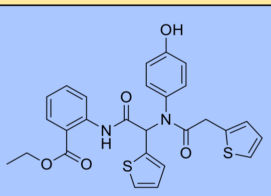	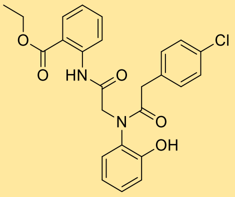	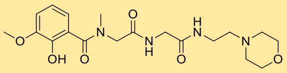
**3**	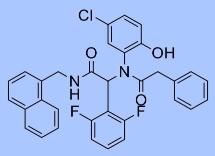	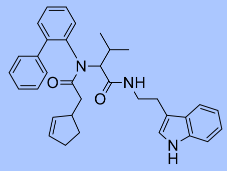	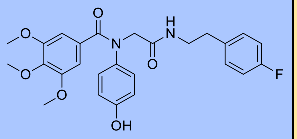	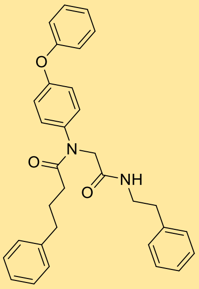

**4**	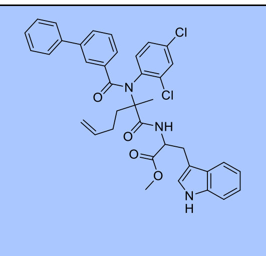	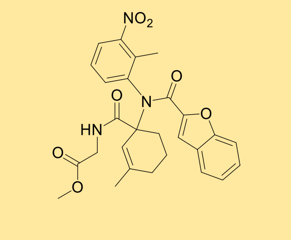	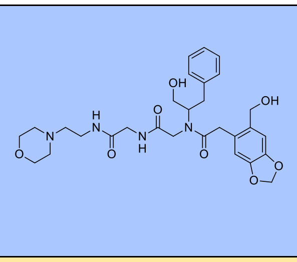	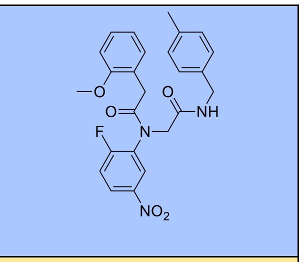
**5**	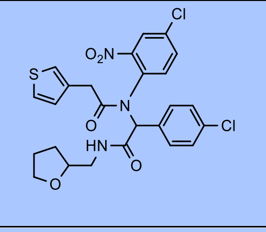	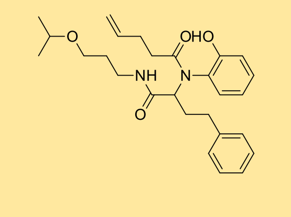	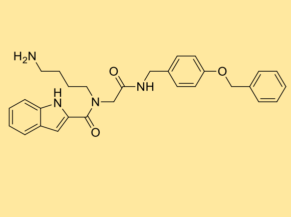	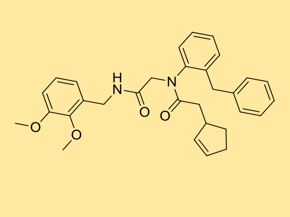
**6**	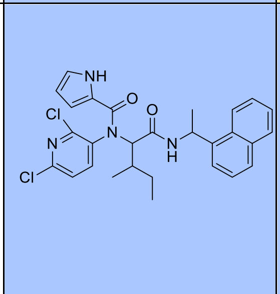	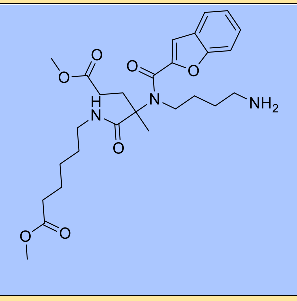	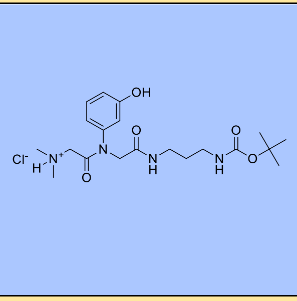	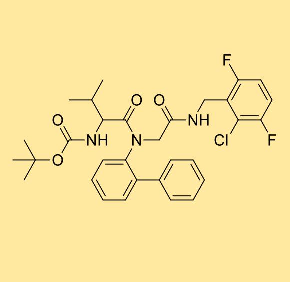
**7**	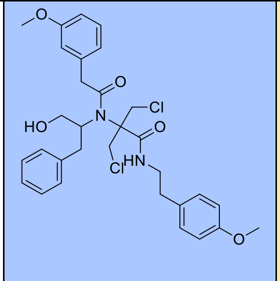	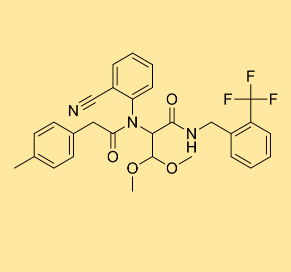	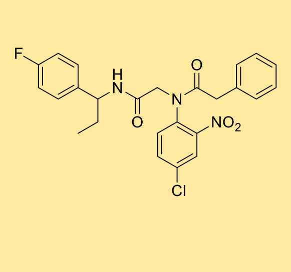	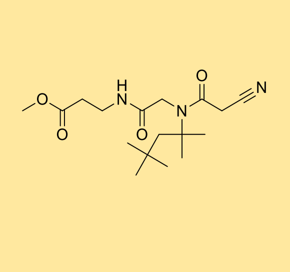
**8**	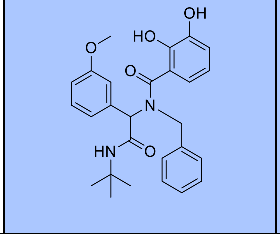	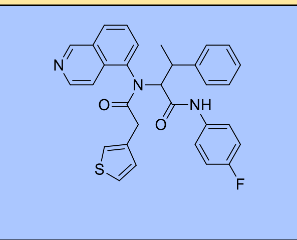	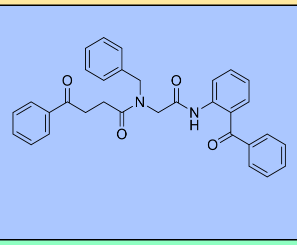	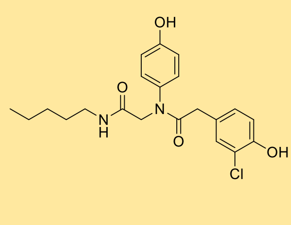
**9**	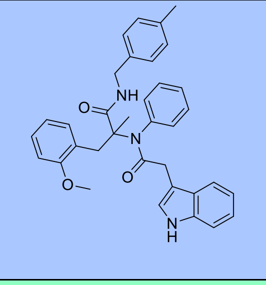	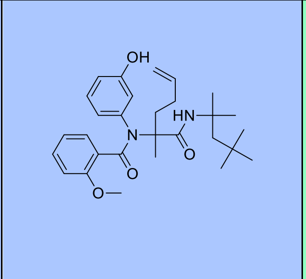	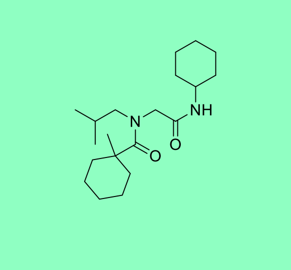	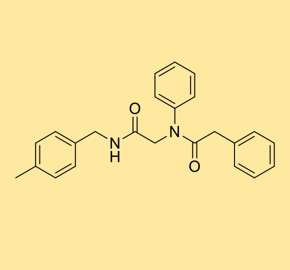
**10**	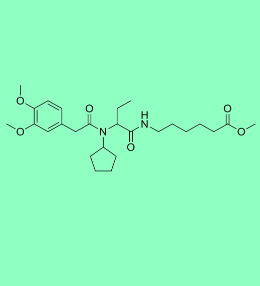	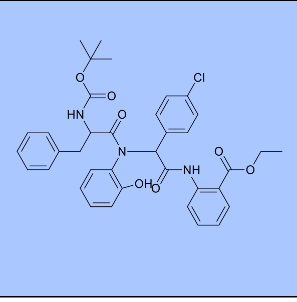	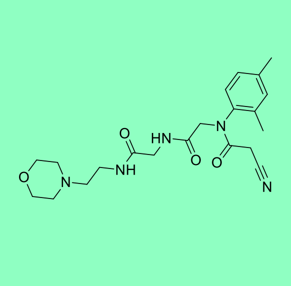	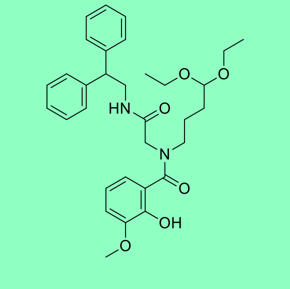

**11**	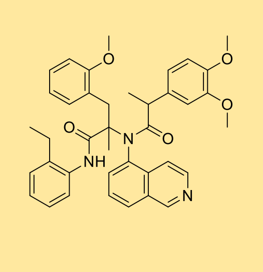	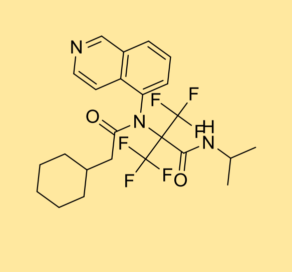	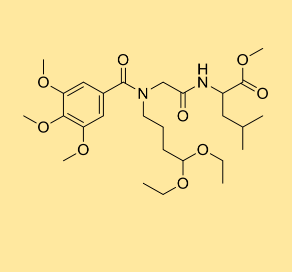	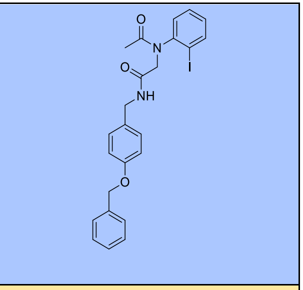
**12**	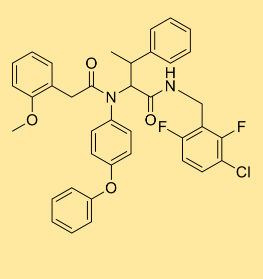	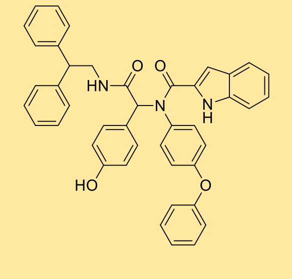	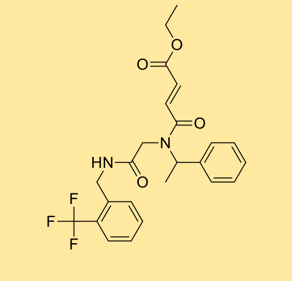	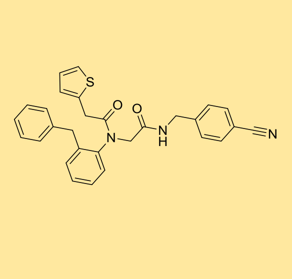
**13**	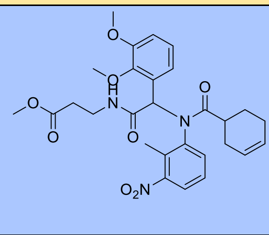	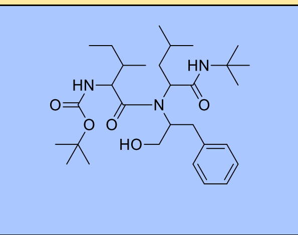	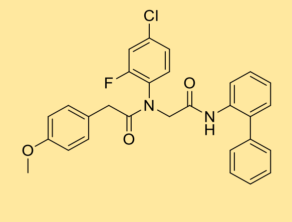	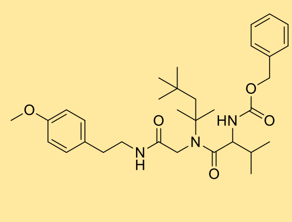
**14**	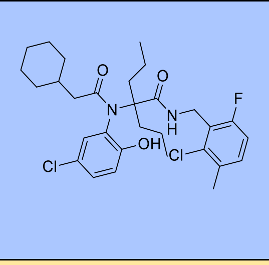	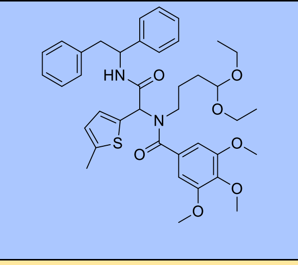	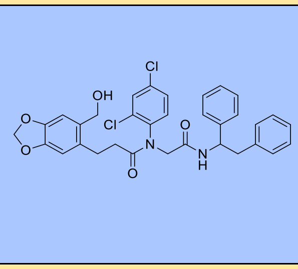	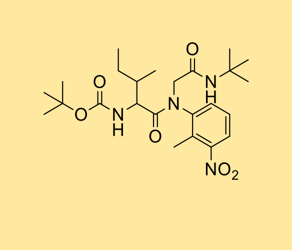
**15**	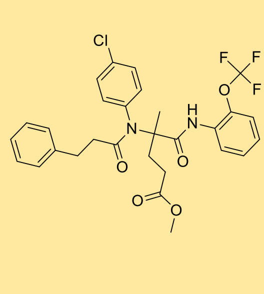	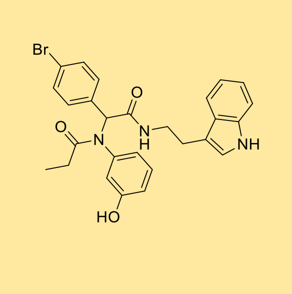	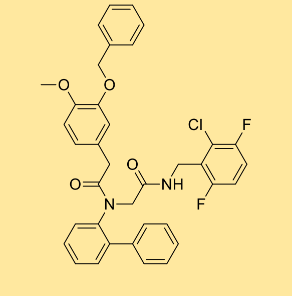	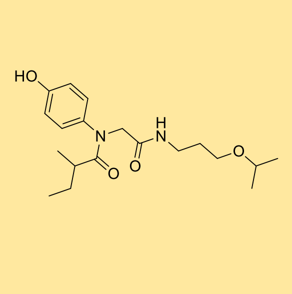
**16**	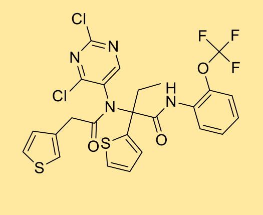	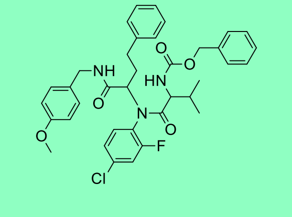	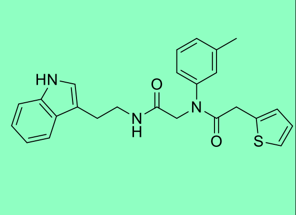	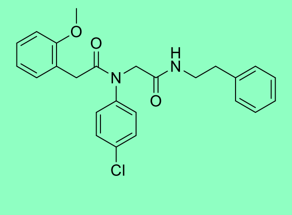
**17**	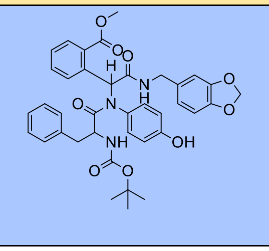	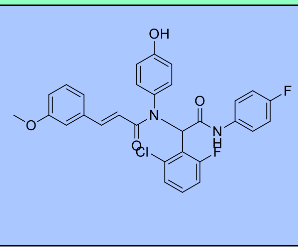	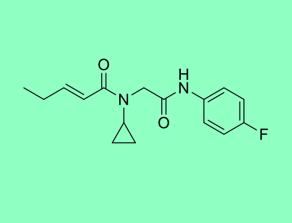	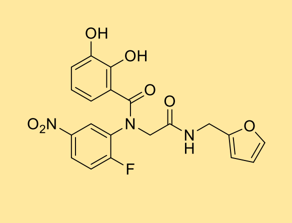

**18**	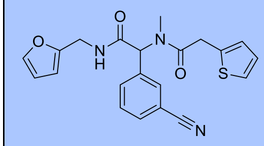	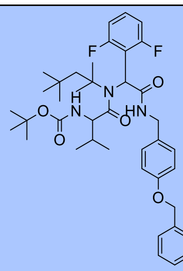	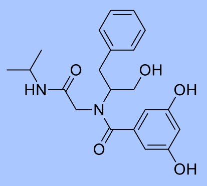	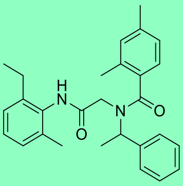
**19**	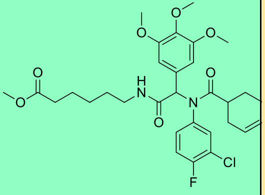	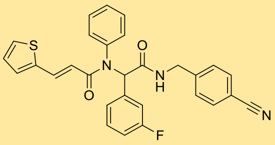	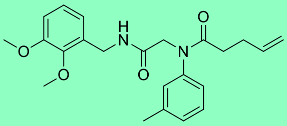	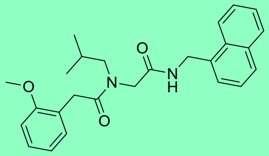
**20**	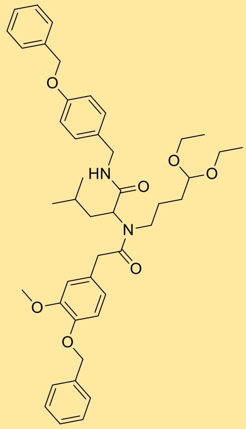	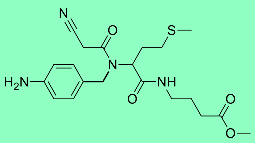	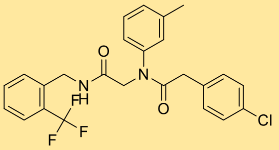	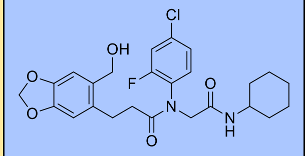
**21**	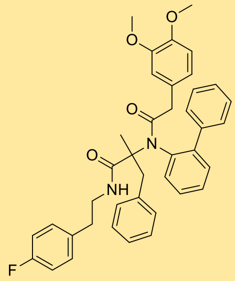	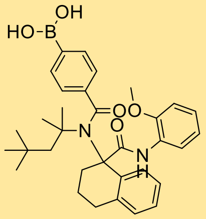	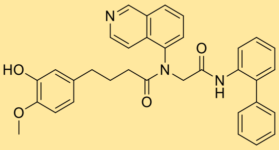	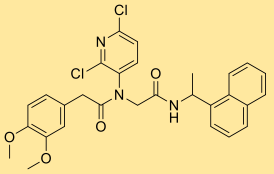
**22**	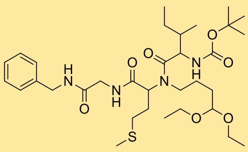	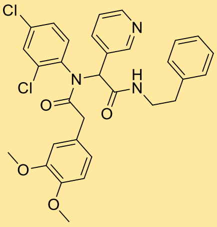	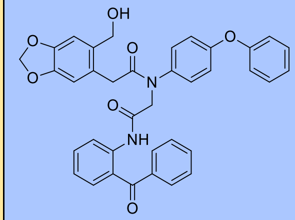	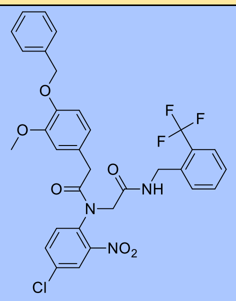

**23**	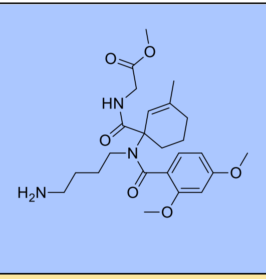	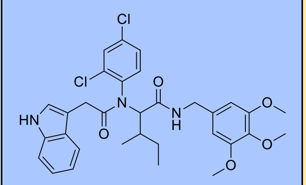	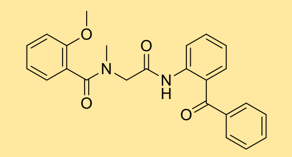	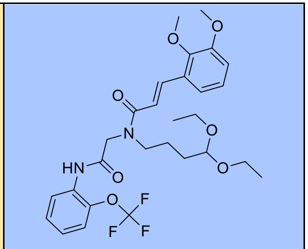
**24**	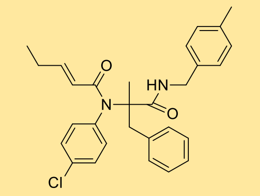	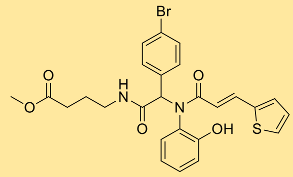	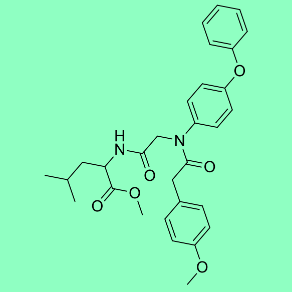	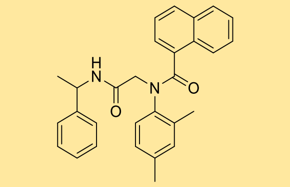

	**M**	**N**	**O**	**P**
**1**	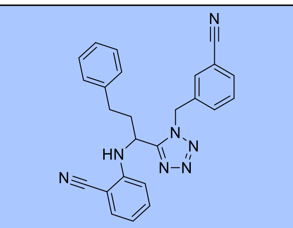	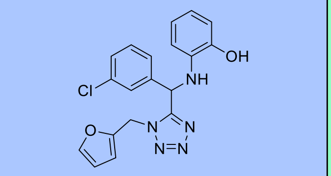	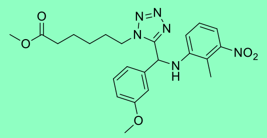	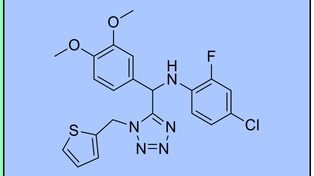
**2**	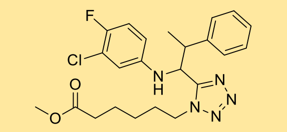	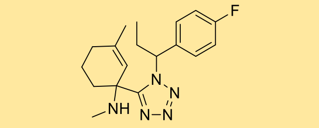	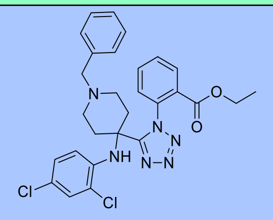	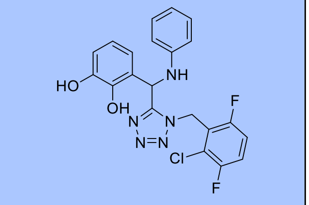
**3**	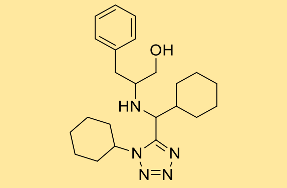	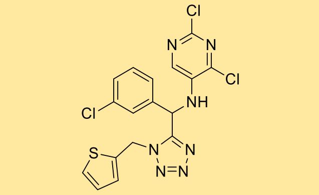	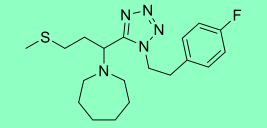	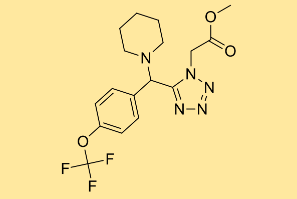
**4**	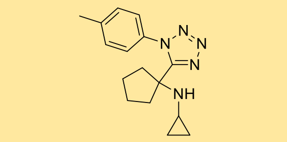	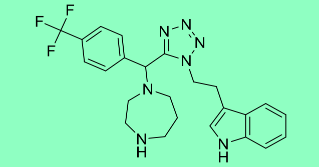	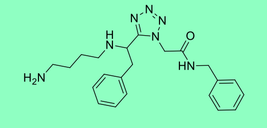	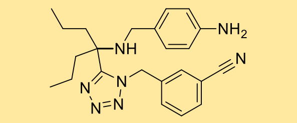
**5**	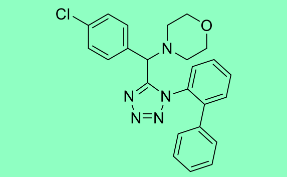	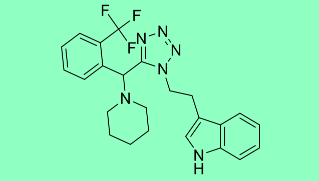	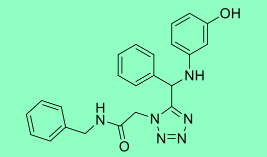	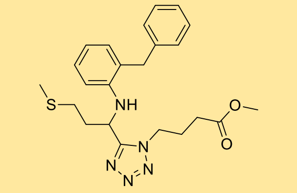

**6**	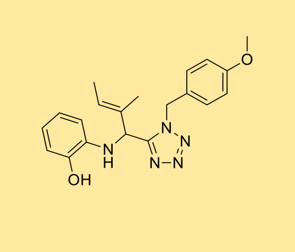	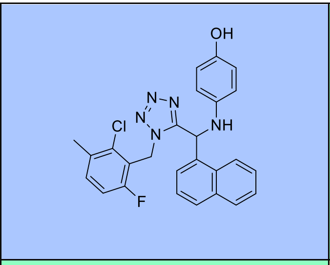	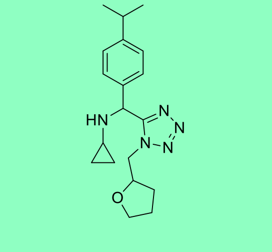	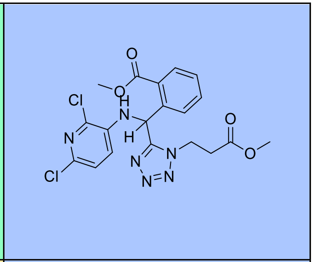
**7**	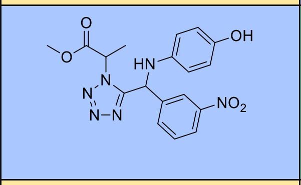	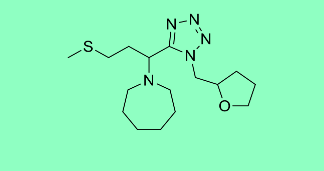	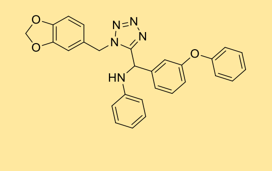	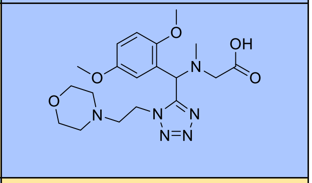
**8**	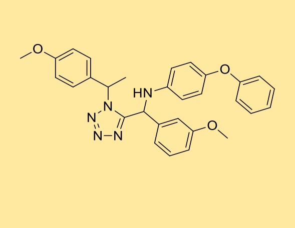	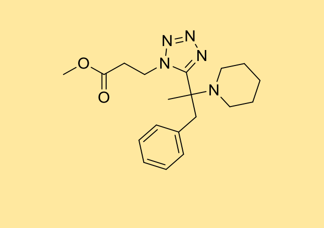	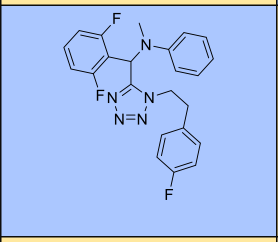	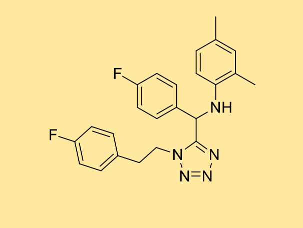
**9**	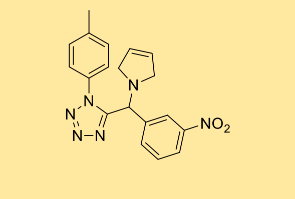	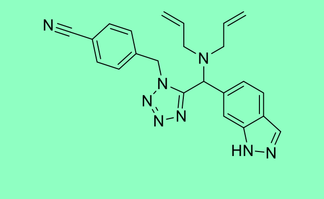	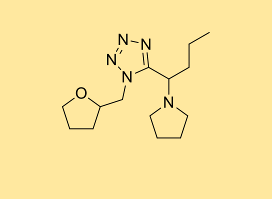	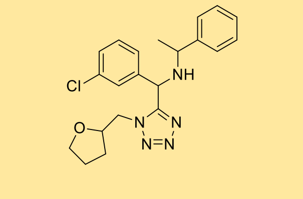
**10**	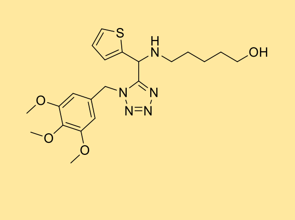	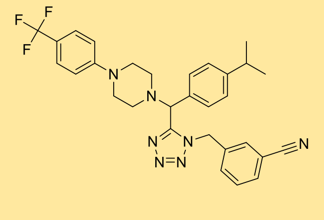	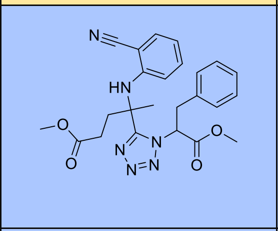	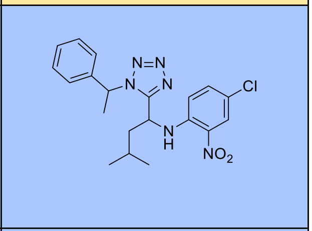
**11**	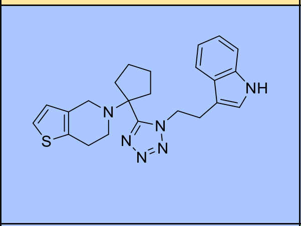	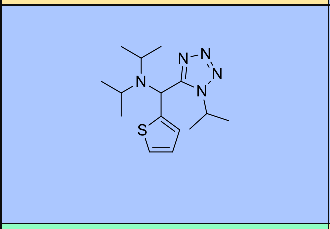	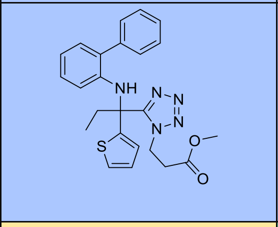	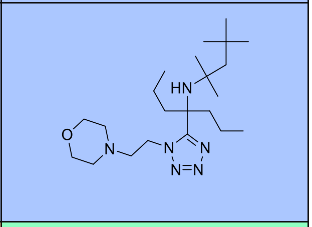
**12**	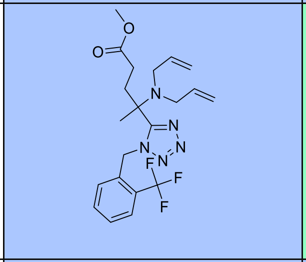	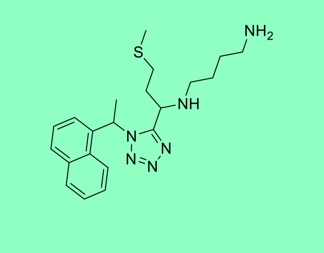	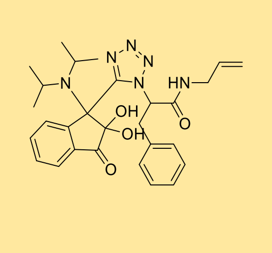	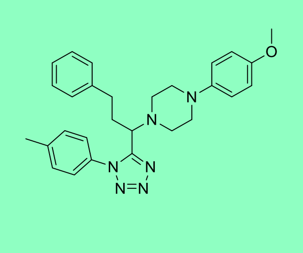
**13**	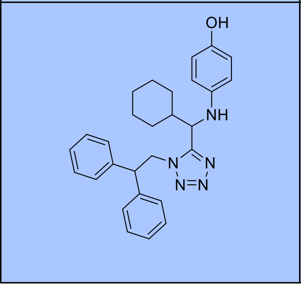	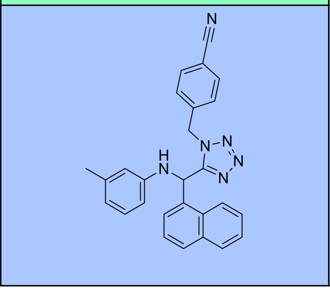	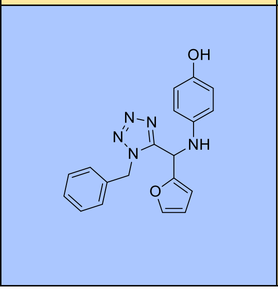	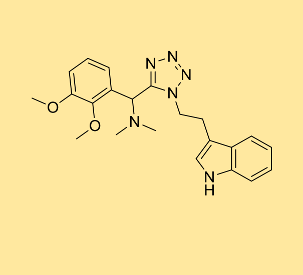

**14**	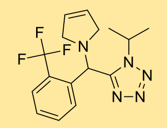	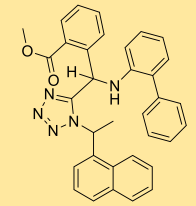	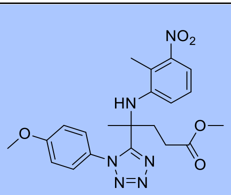	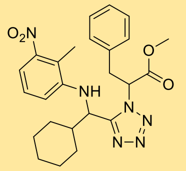
**15**	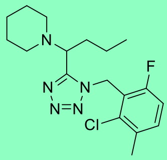	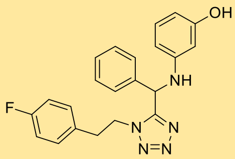	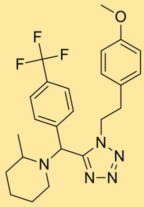	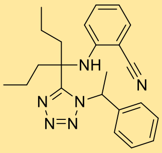
**16**	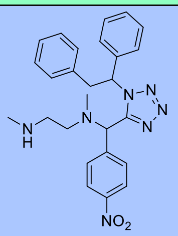	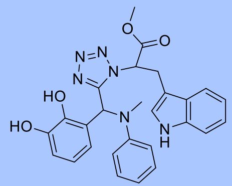	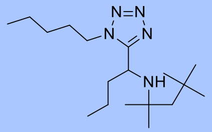	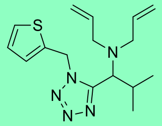
**17**	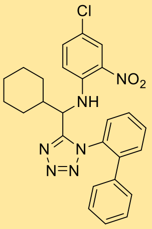	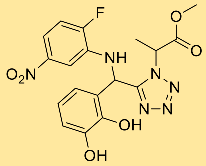	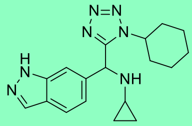	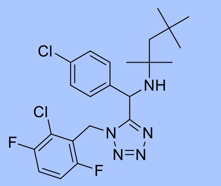
**18**	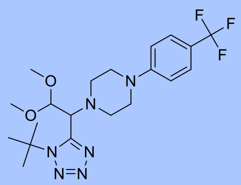	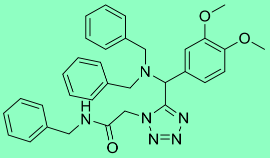	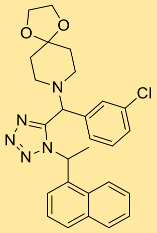	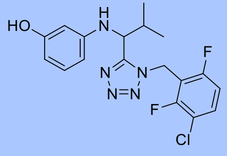
**19**	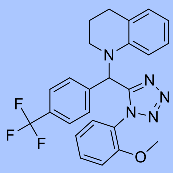	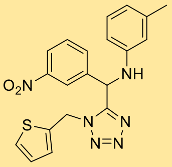	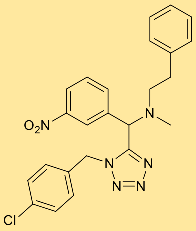	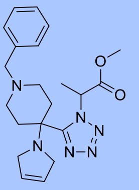
**20**	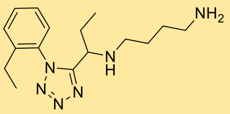	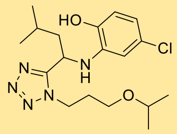	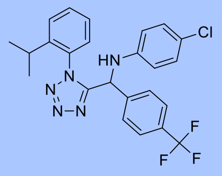	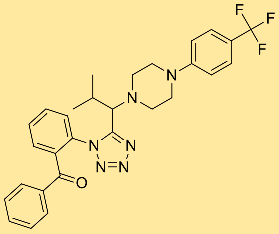

**21**	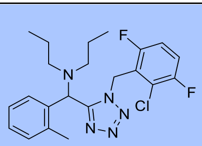	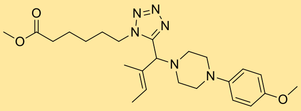	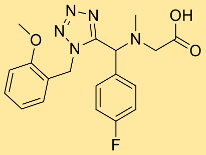	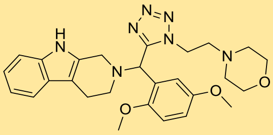
**22**	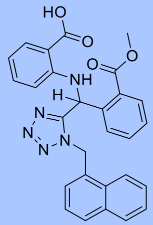	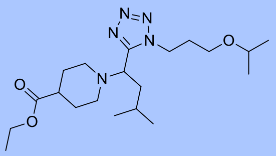	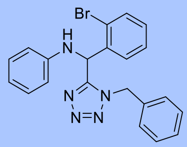	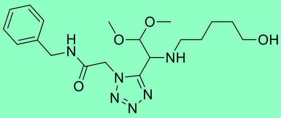
**23**	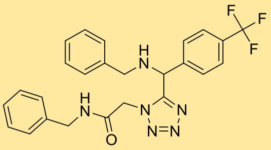	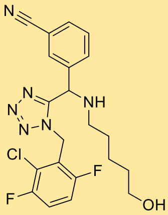	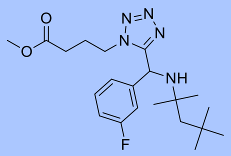	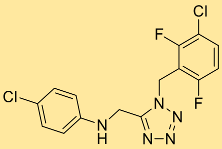
**24**	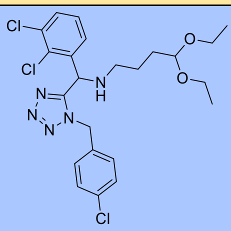	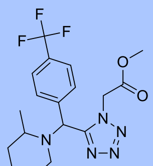	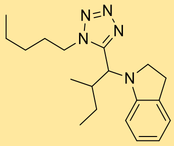	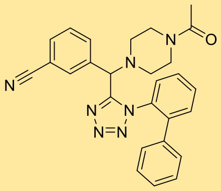

Destination plate II

	A	B	C	D
**1**	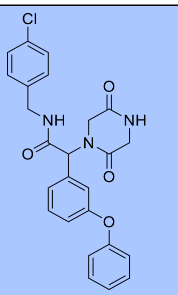	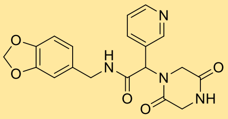	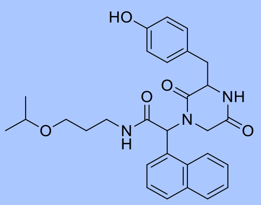	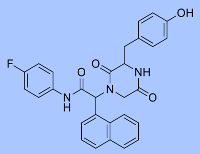
**2**	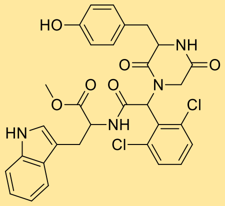	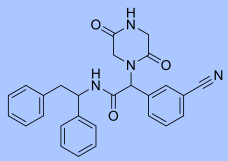	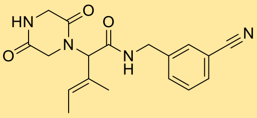	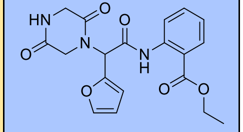

**3**	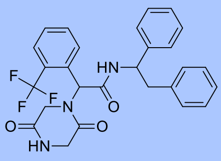	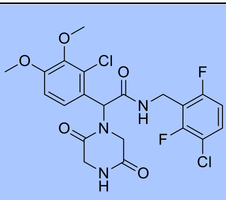	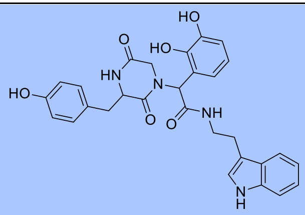	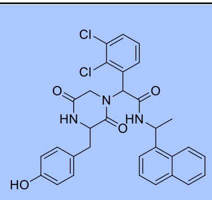
**4**	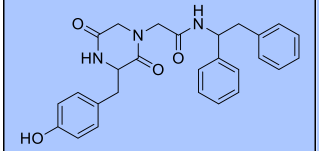	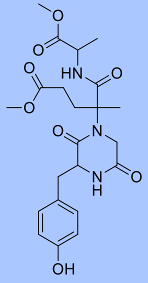	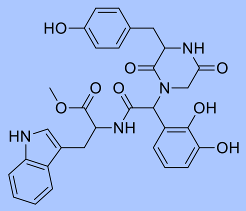	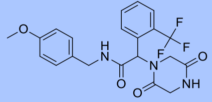
**5**	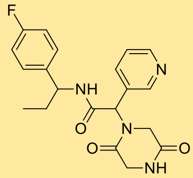	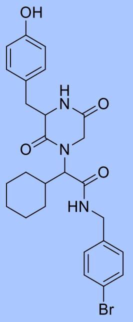	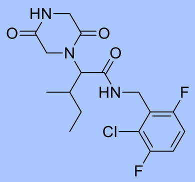	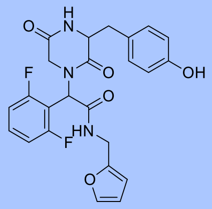
**6**	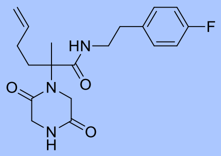	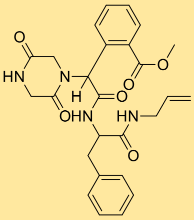	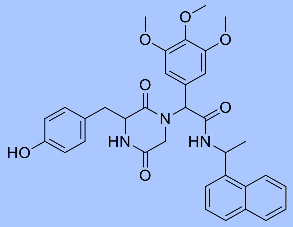	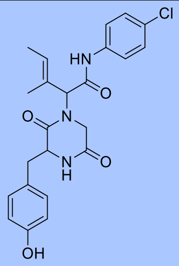
**7**	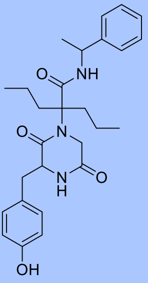	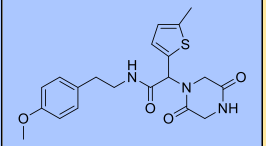	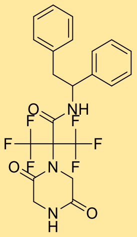	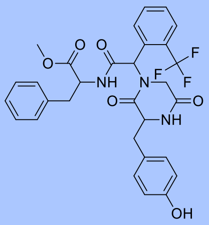
**8**	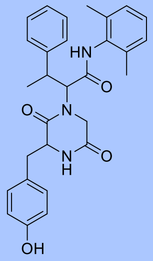	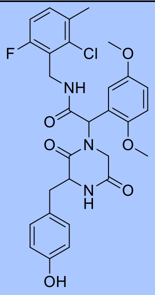	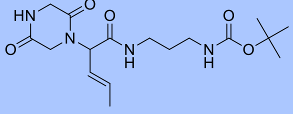	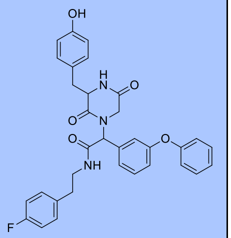

**9**	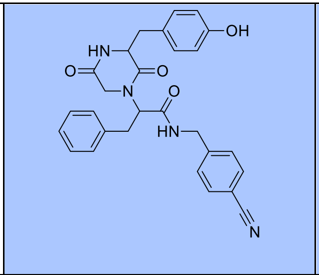	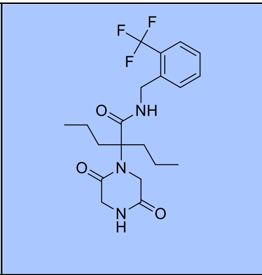	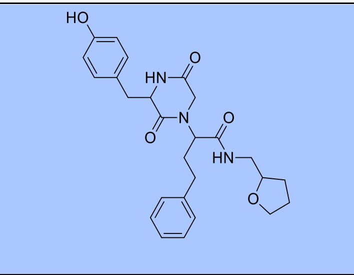	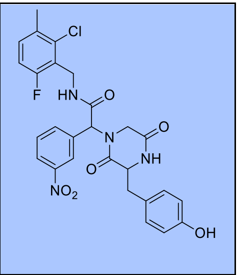
**10**	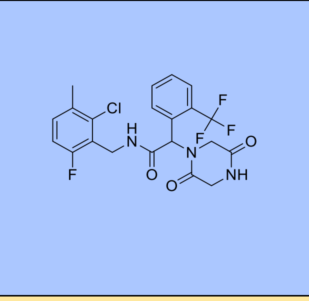	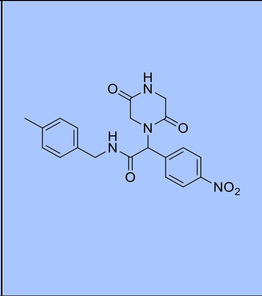	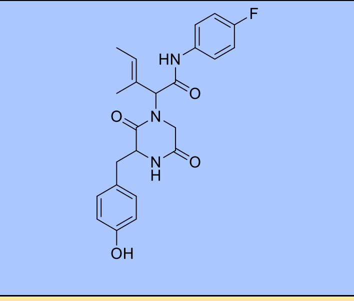	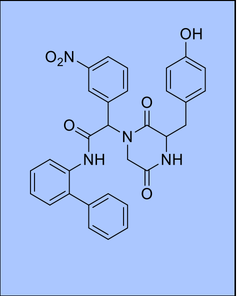
**11**	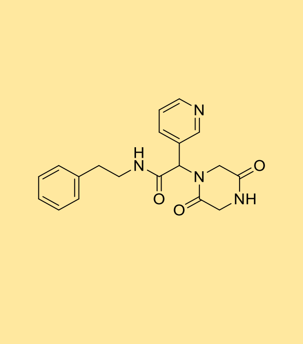	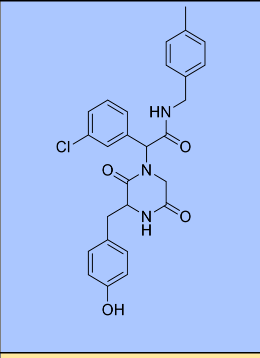	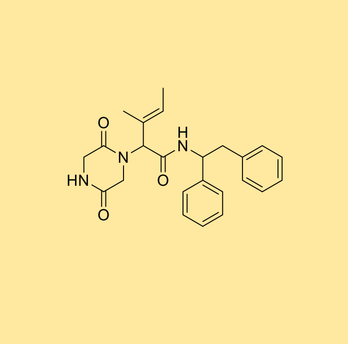	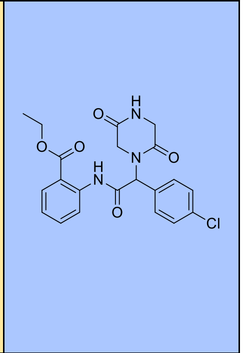
**12**	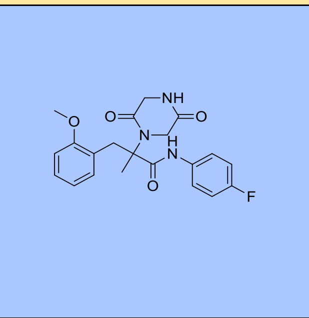	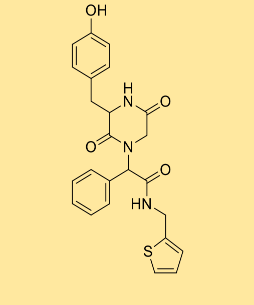	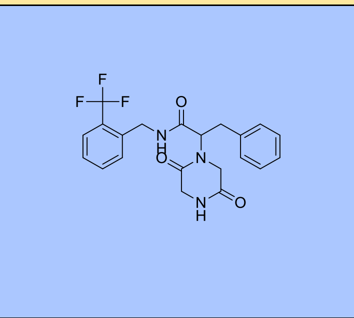	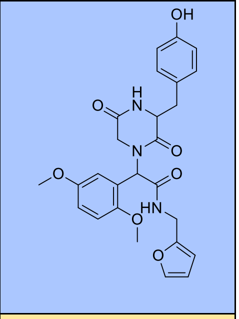
**13**	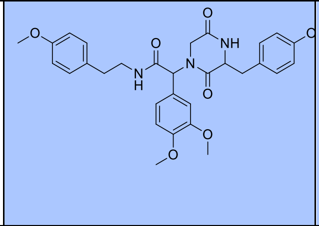	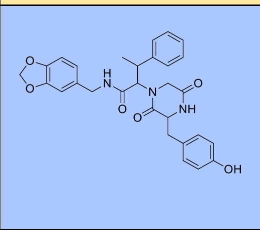	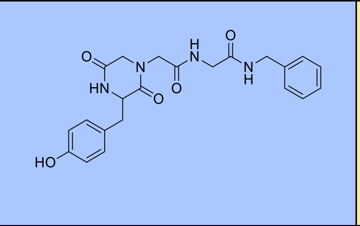	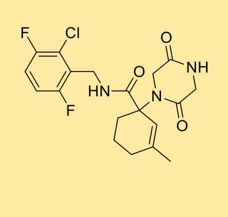
**14**	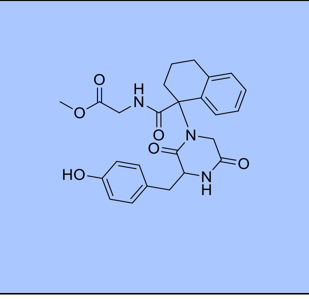	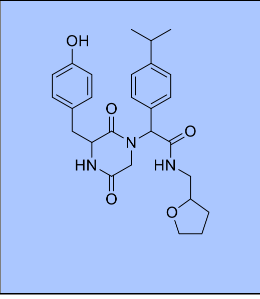	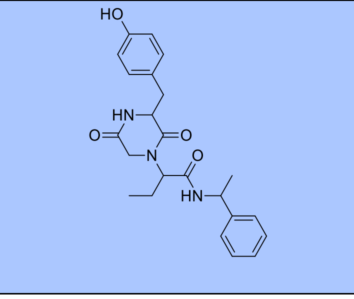	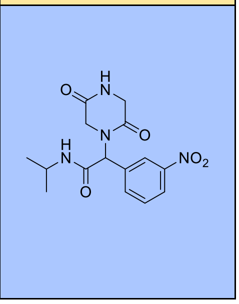

**15**	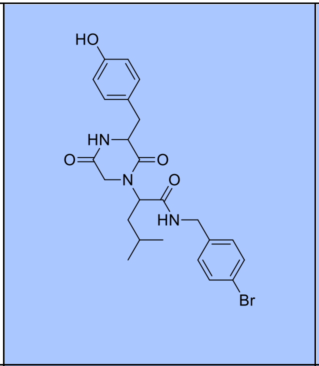	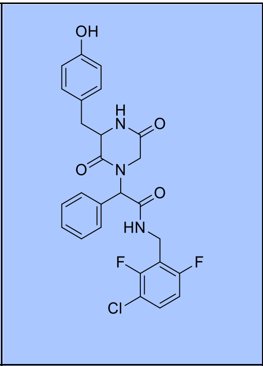	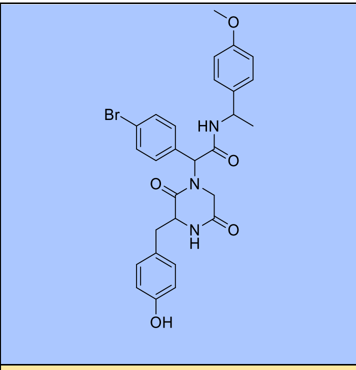	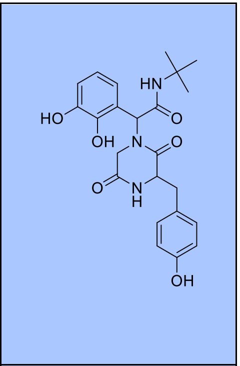
**16**	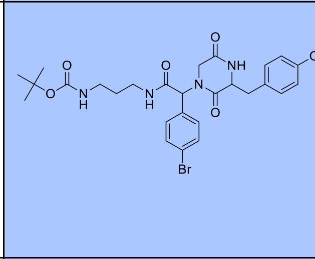	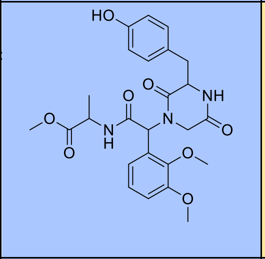	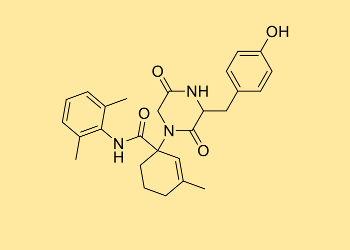	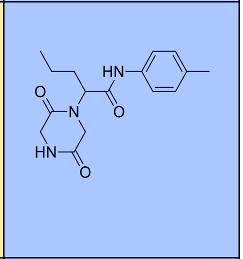
**17**	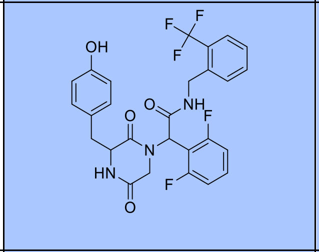	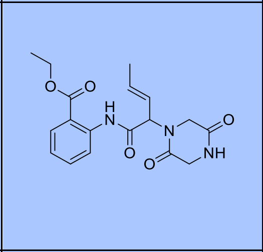	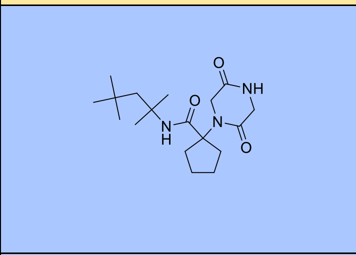	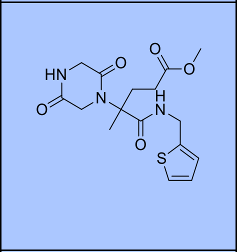
**18**	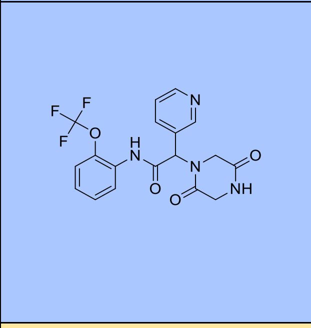	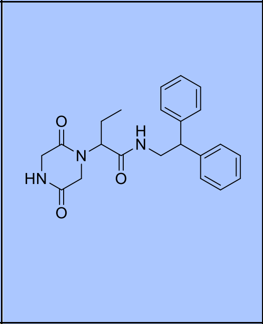	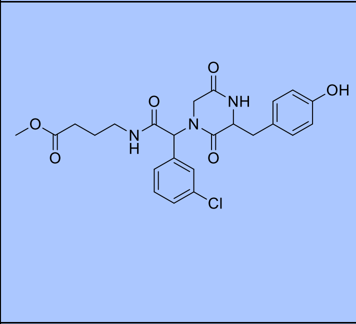	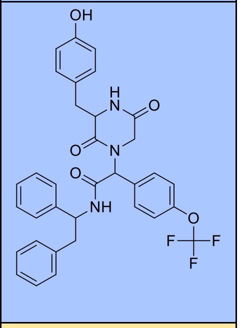
**19**	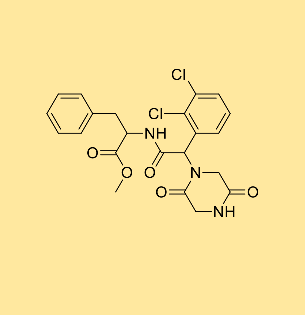	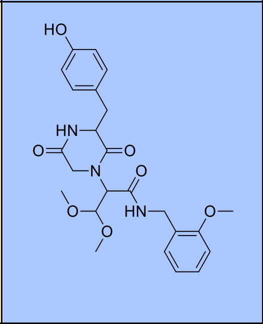	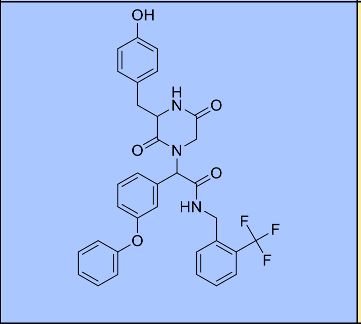	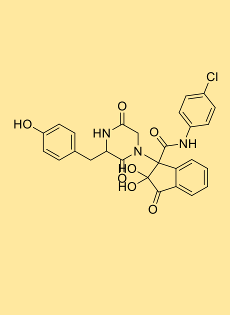
**20**	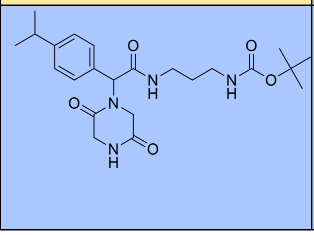	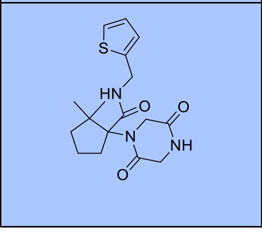	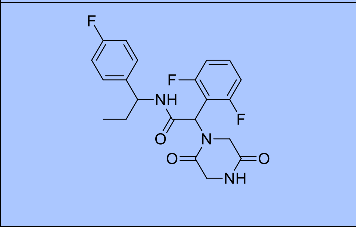	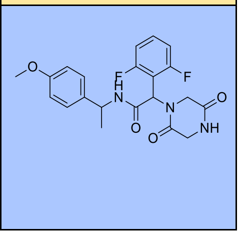

**21**	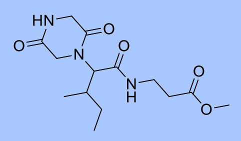	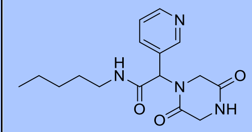	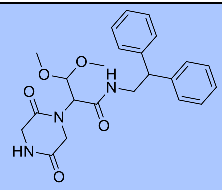	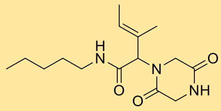
**22**	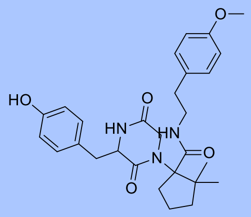	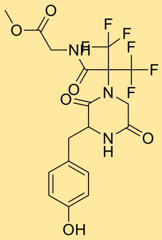	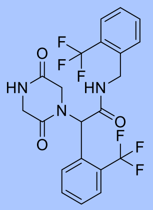	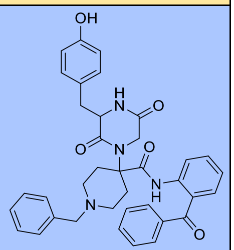
**23**	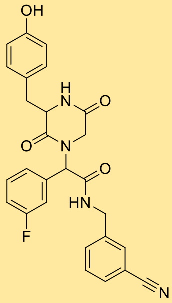	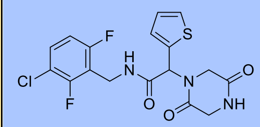	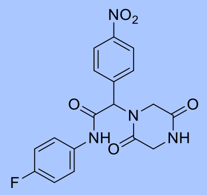	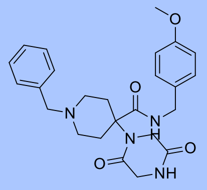
**24**	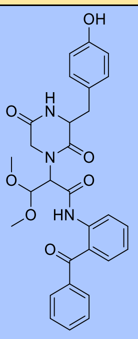	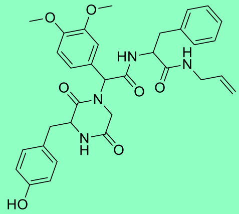	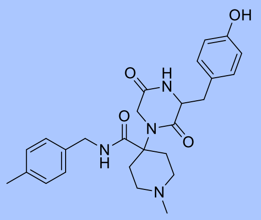	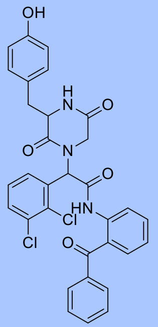

	E	F	G	H
**1**	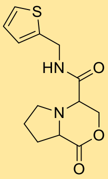	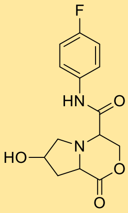	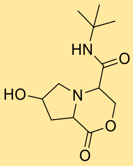	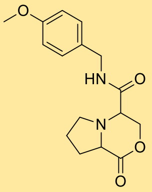
**2**	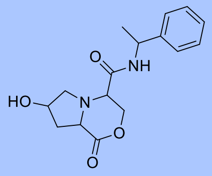	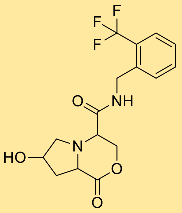	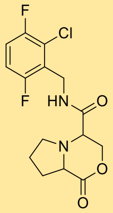	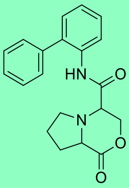

**3**	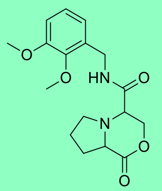	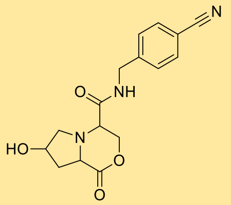	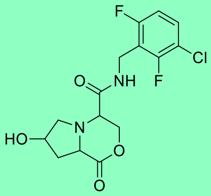	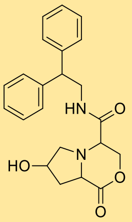
**4**	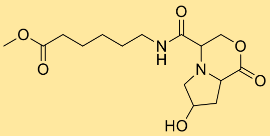	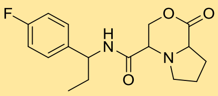	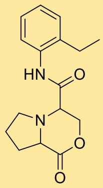	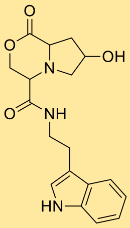
**5**	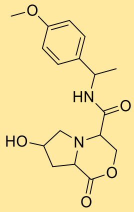	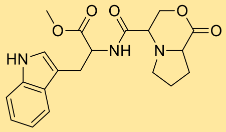	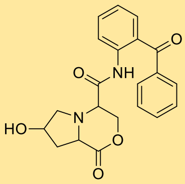	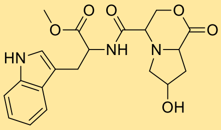
**6**	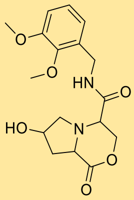	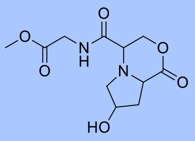	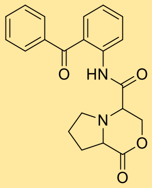	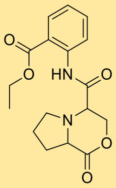
**7**	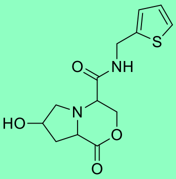	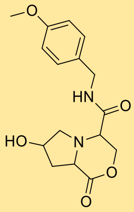	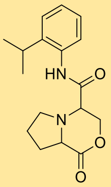	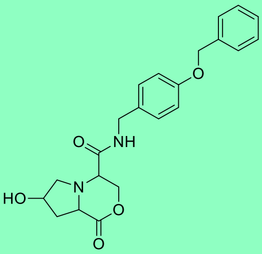
**8**	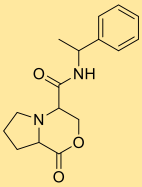	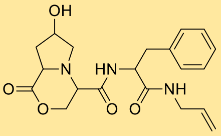	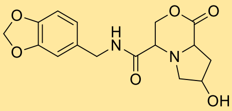	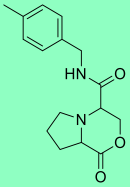
**9**	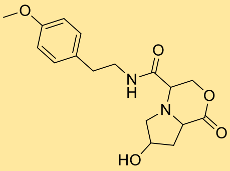	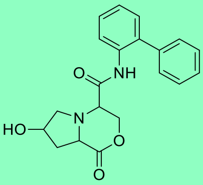	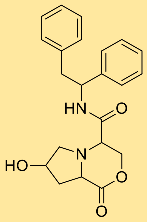	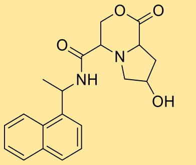

**10**	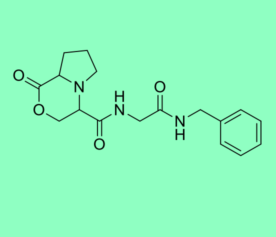	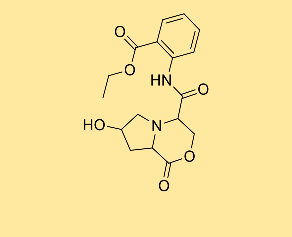	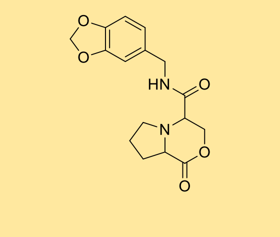	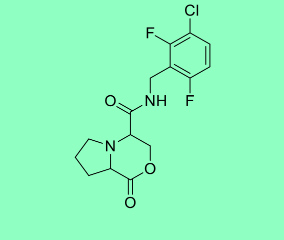
**11**	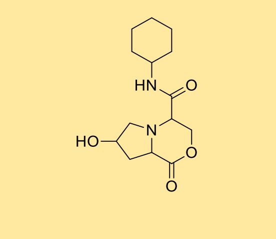	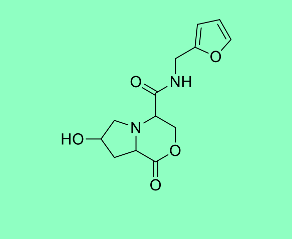	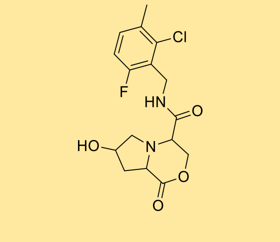	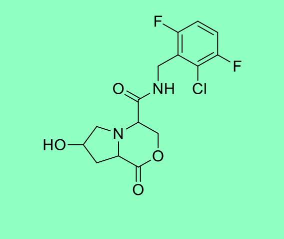
**12**	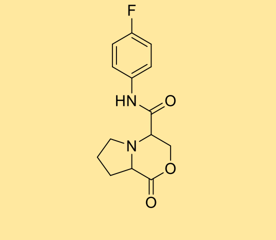	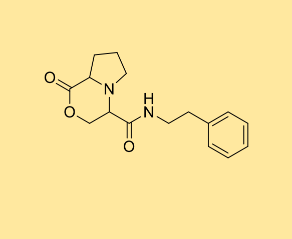	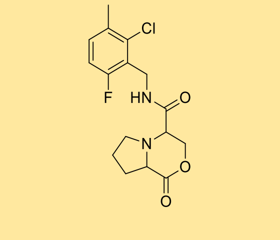	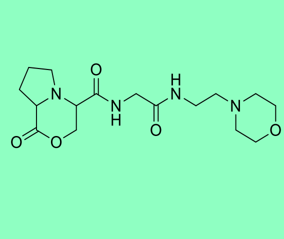
**13**	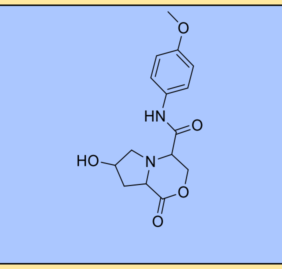	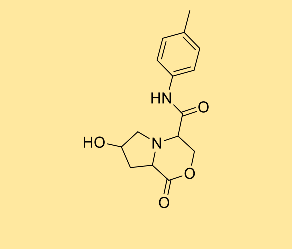	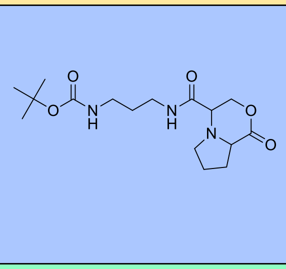	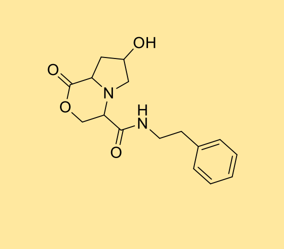
**14**	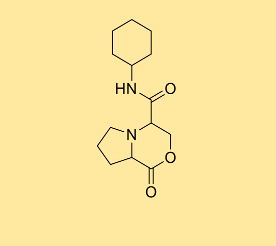	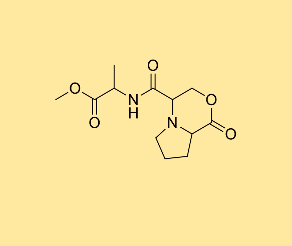	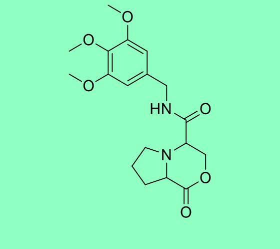	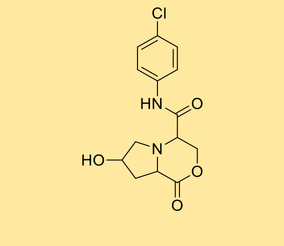
**15**	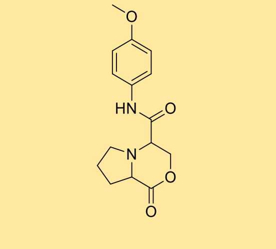	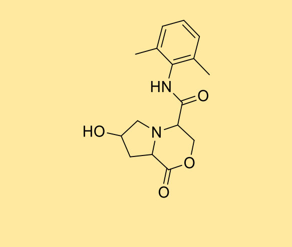	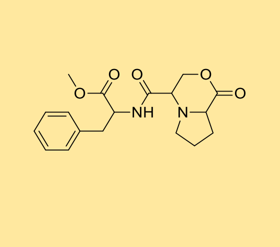	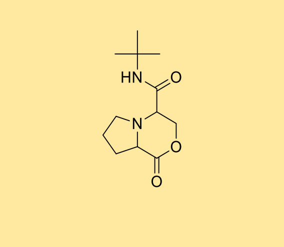
**16**	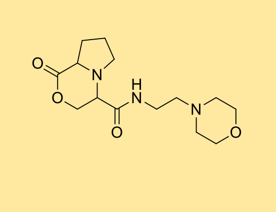	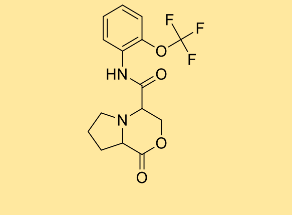	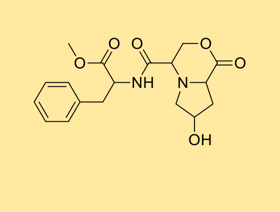	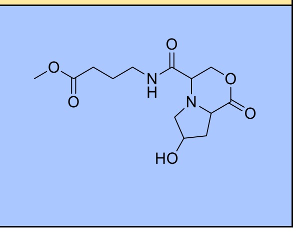

**17**	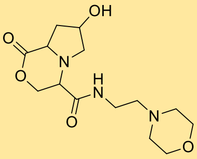	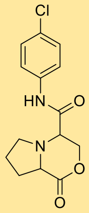	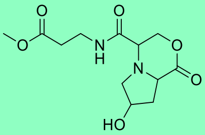	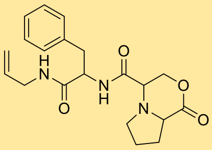
**18**	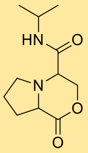	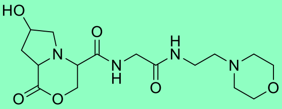	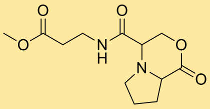	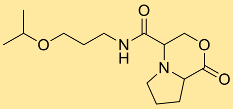
**19**	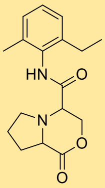	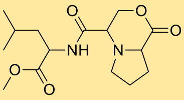	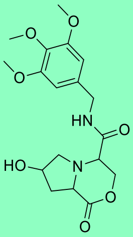	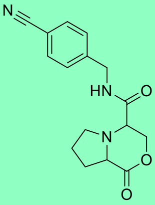
**20**	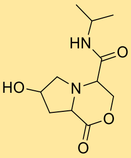	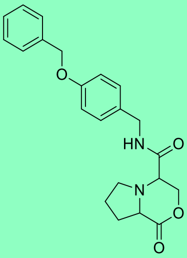	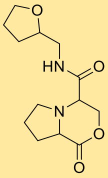	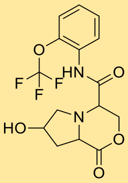
**21**	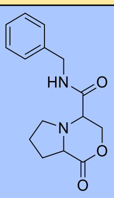	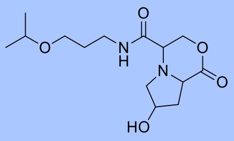	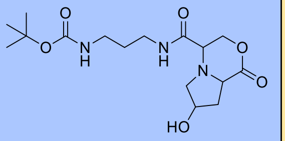	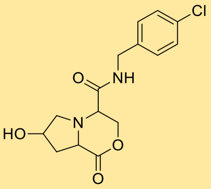
**22**	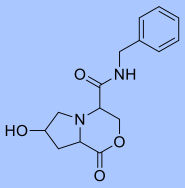	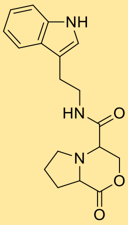	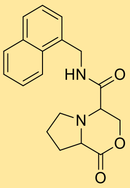	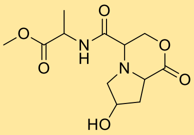
**23**	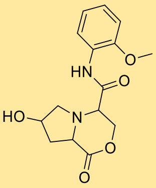	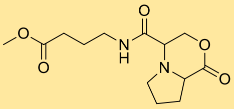	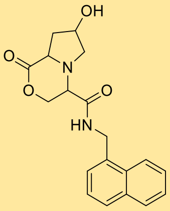	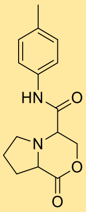

**24**	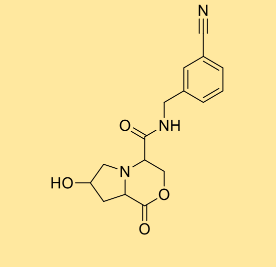	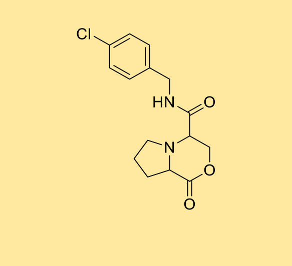	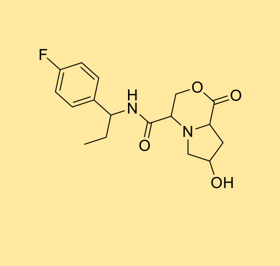	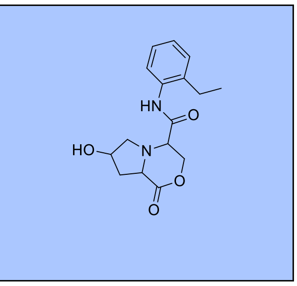

	I	J	K	L
**1**	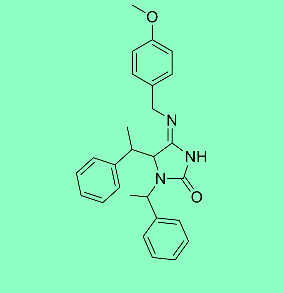	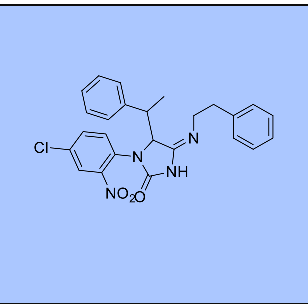	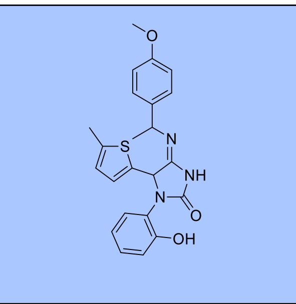	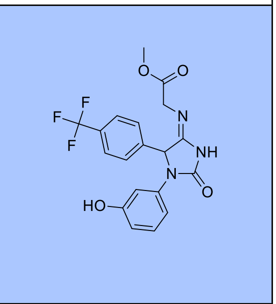
**2**	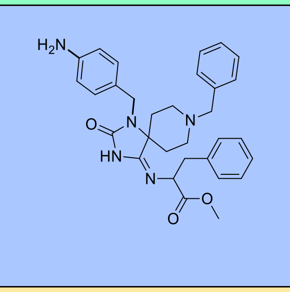	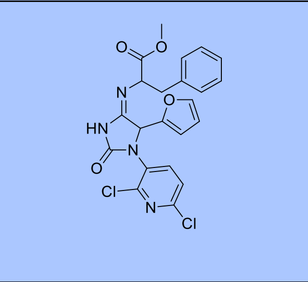	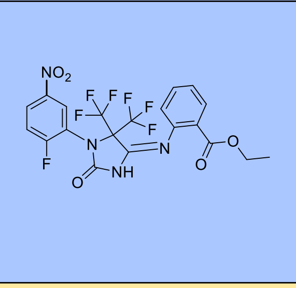	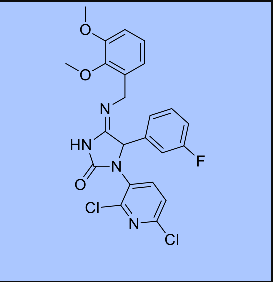
**3**	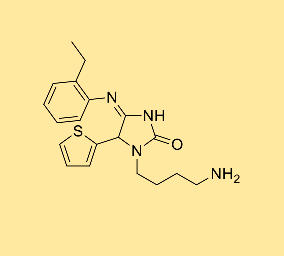	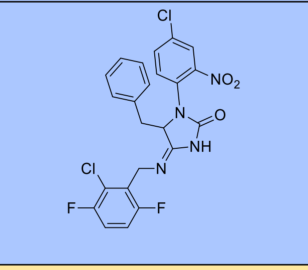	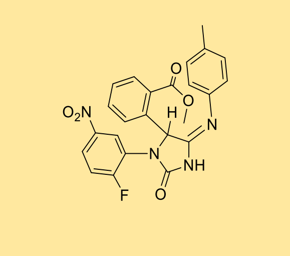	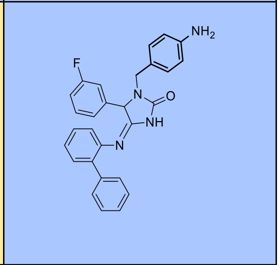
**4**	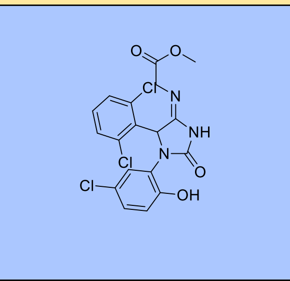	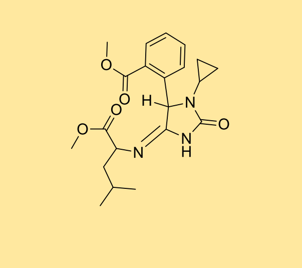	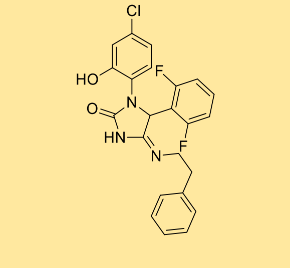	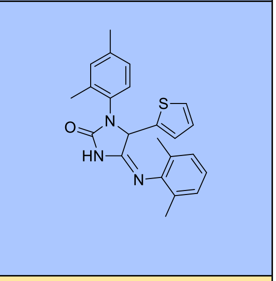
**5**	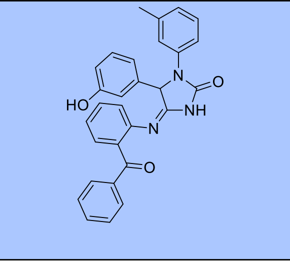	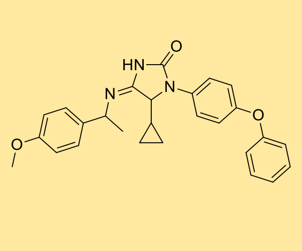	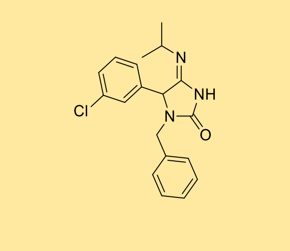	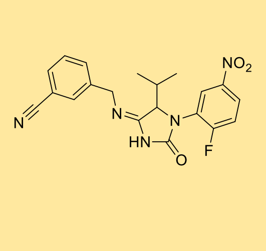

**6**	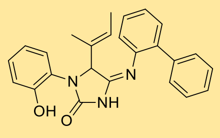	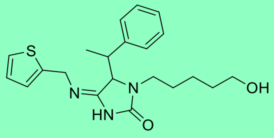	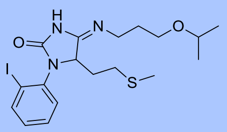	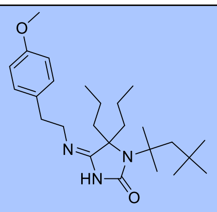
**7**	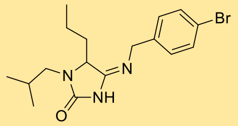	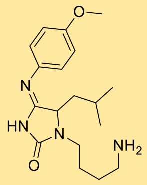	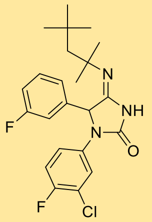	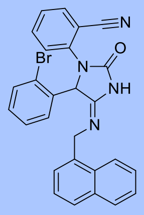
**8**	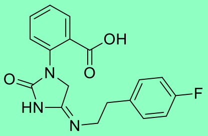	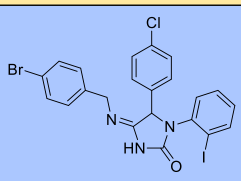	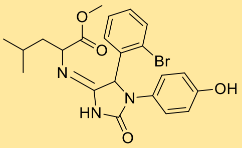	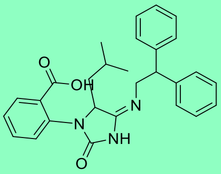
**9**	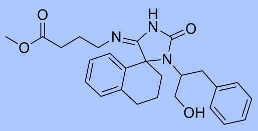	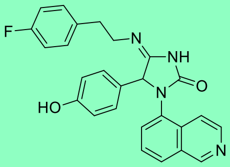	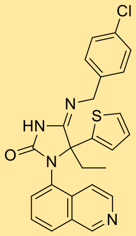	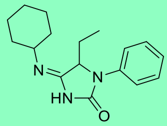
**10**	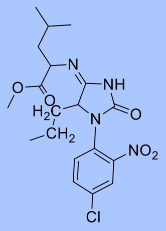	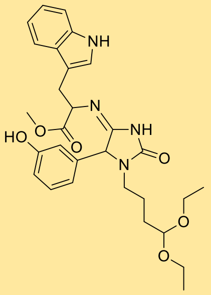	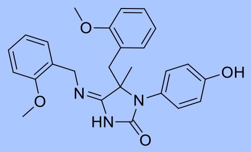	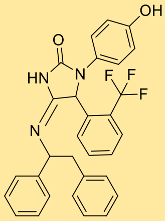
**11**	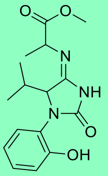	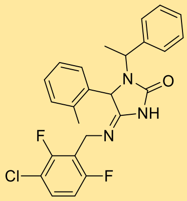	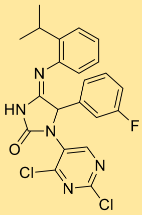	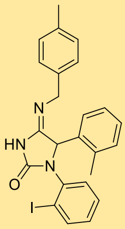
**12**	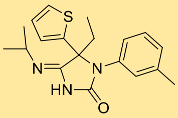	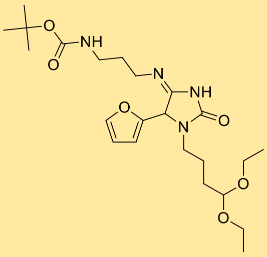	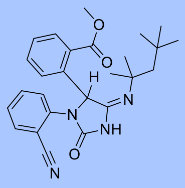	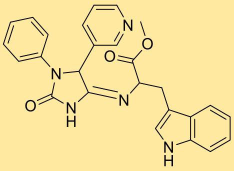

**13**	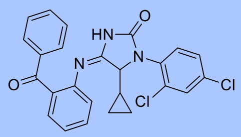	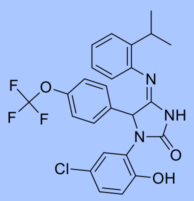	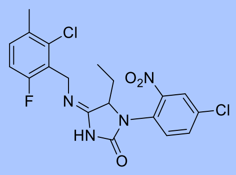	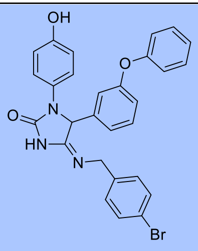
**14**	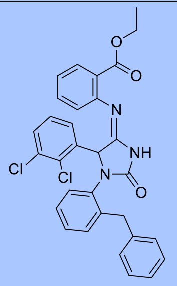	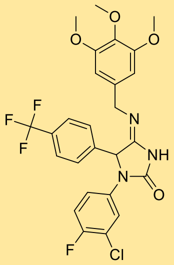	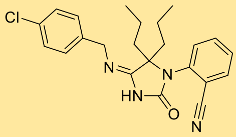	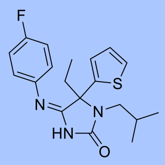
**15**	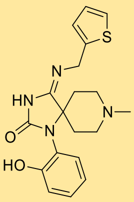	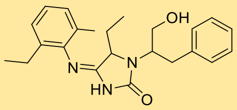	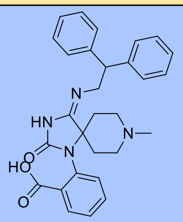	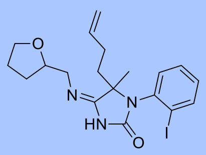
**16**	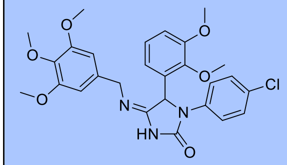	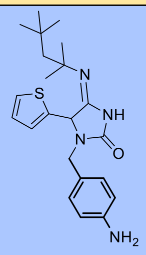	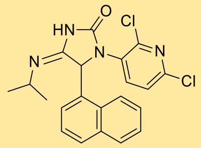	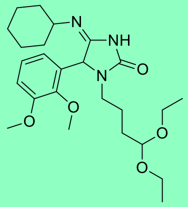
**17**	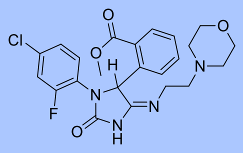	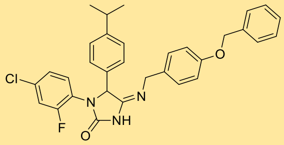	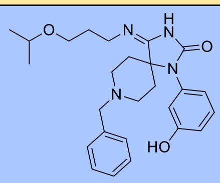	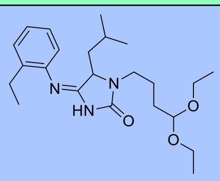
**18**	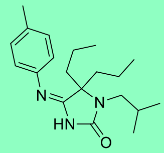	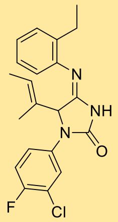	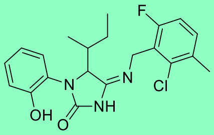	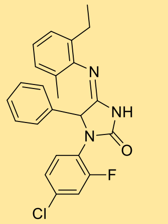
**19**	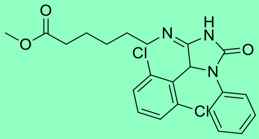	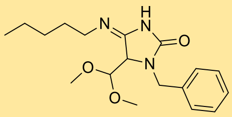	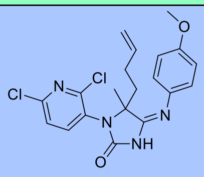	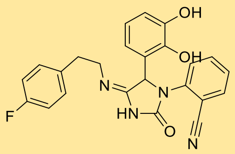

**20**	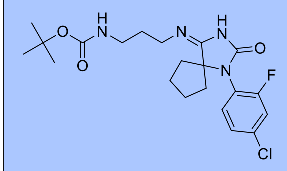	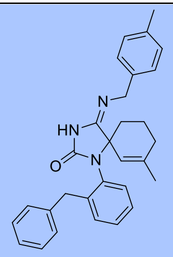	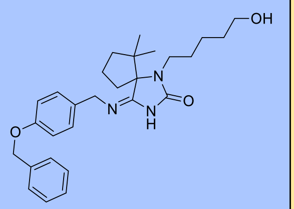	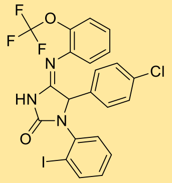
**21**	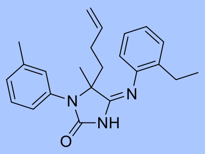	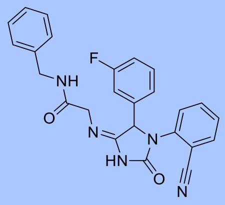	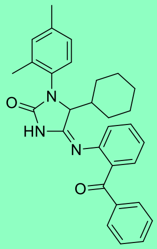	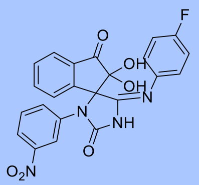
**22**	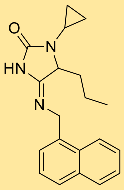	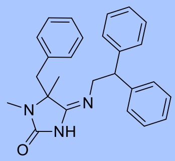	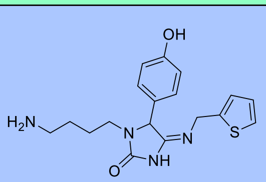	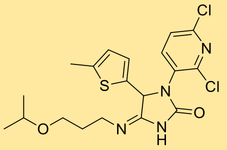
**23**	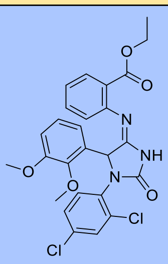	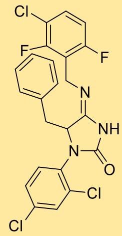	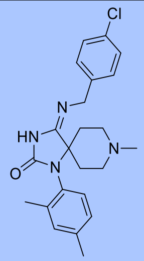	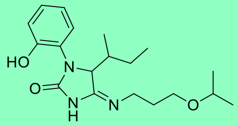
**24**	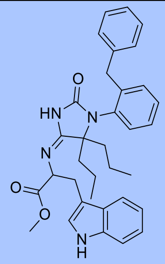	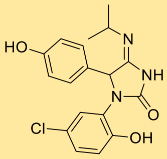	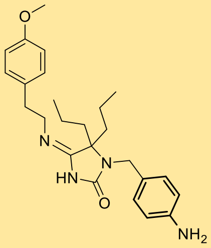	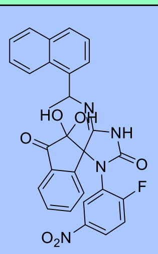

	M	N	O	P
**1**	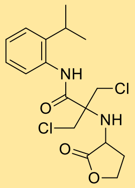	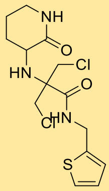	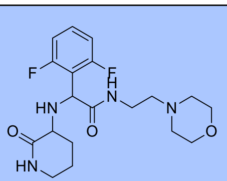	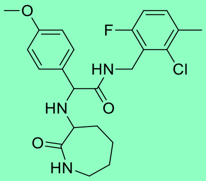

**2**	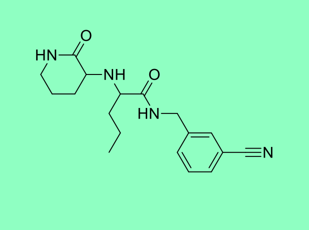	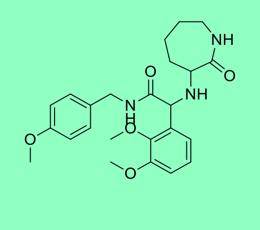	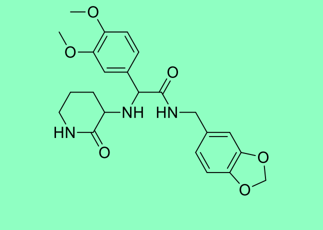	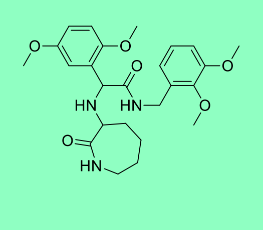
**3**	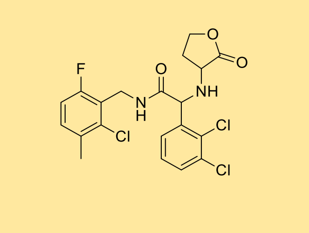	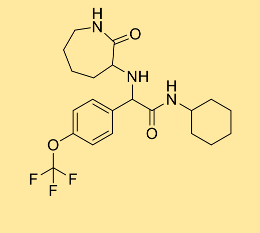	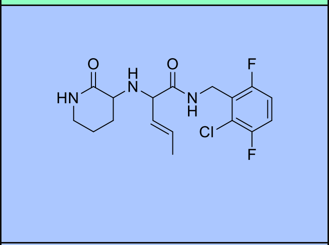	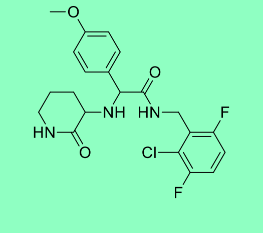
**4**	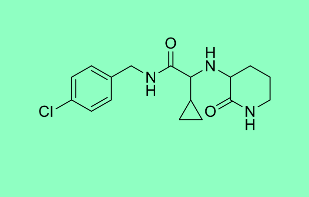	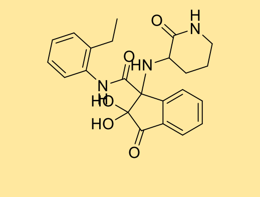	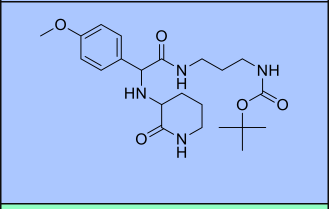	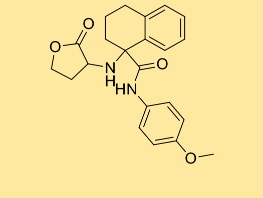
**5**	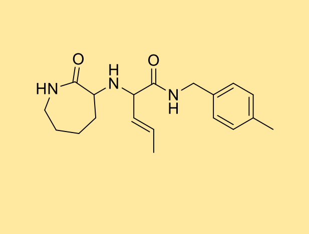	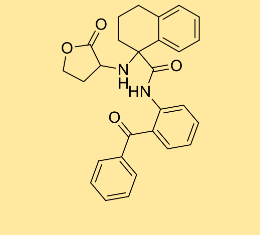	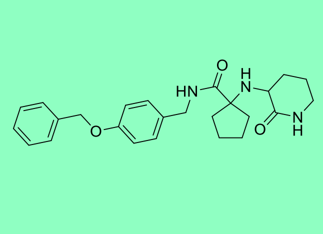	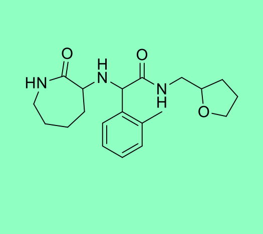
**6**	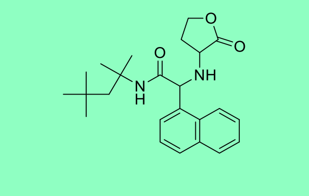	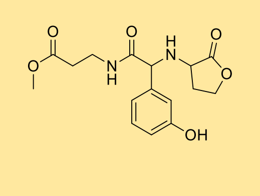	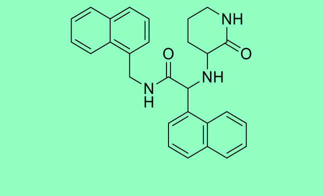	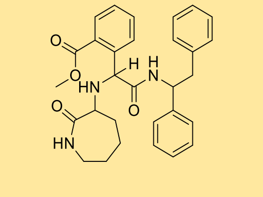
**7**	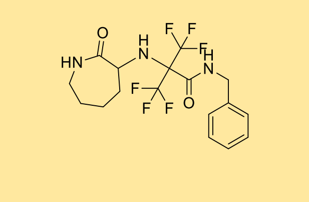	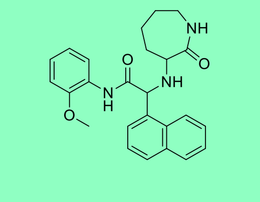	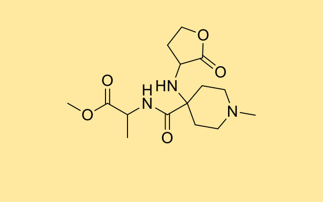	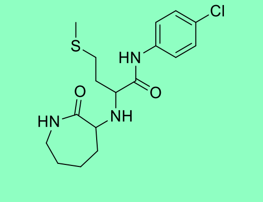
**8**	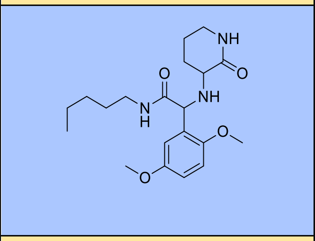	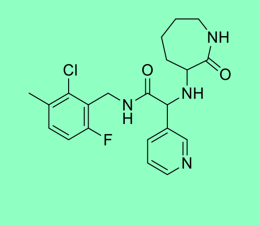	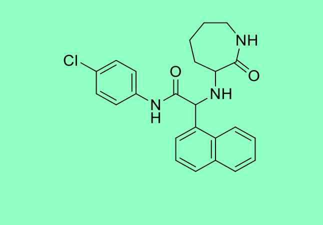	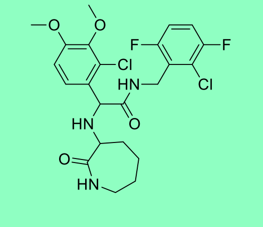
**9**	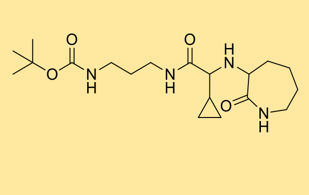	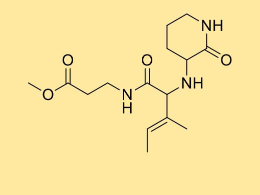	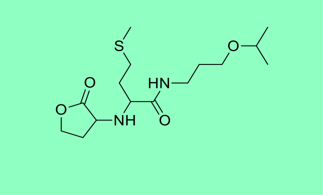	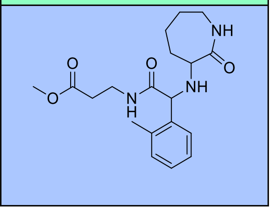

**10**	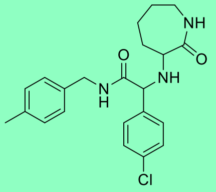	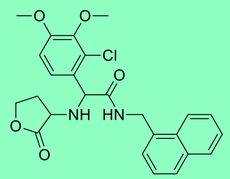	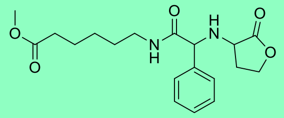	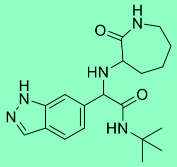
**11**	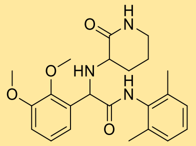	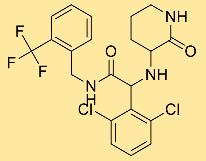	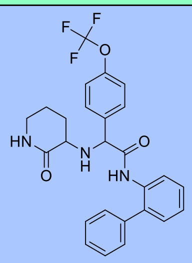	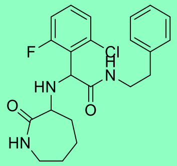
**12**	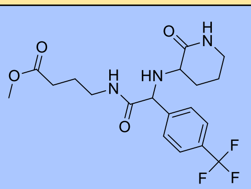	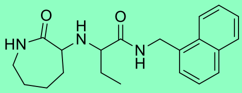	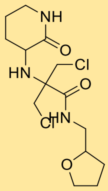	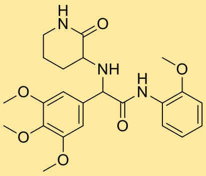
**13**	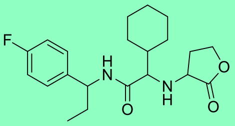	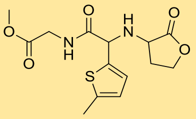	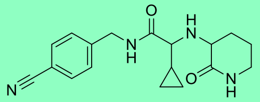	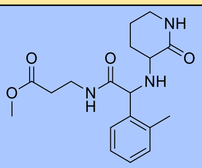
**14**	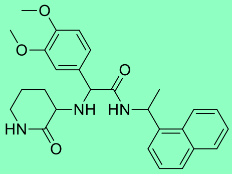	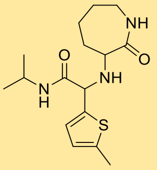	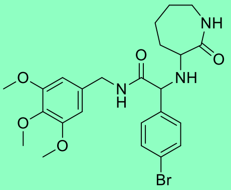	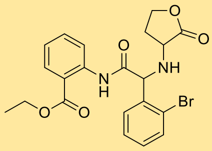
**15**	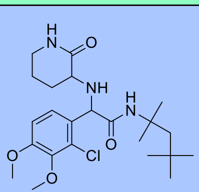	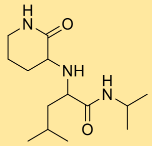	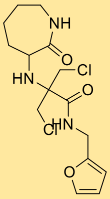	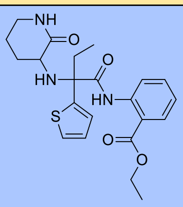
**16**	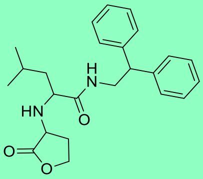	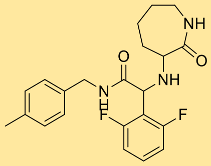	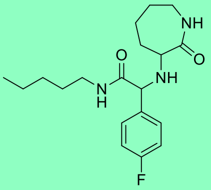	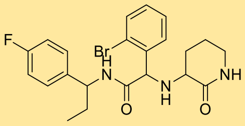
**17**	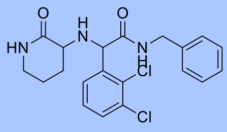	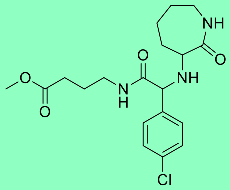	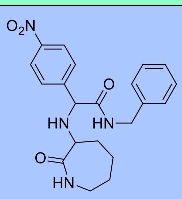	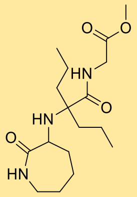

**18**	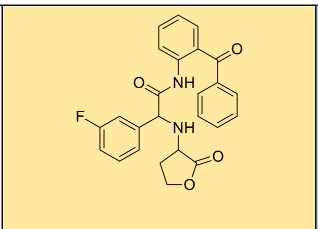	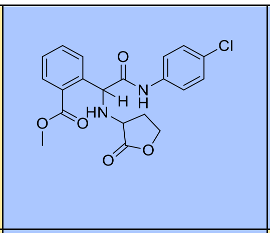	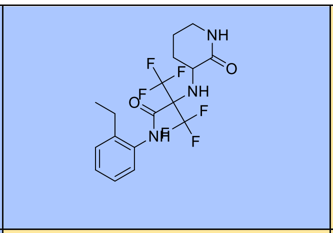	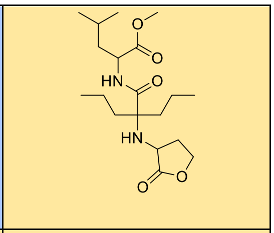
**19**	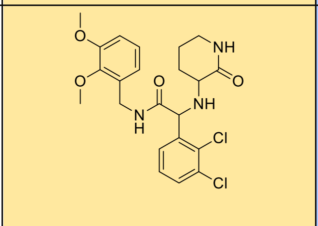	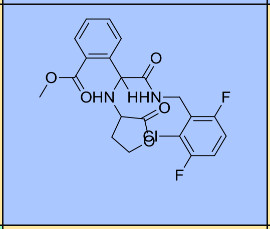	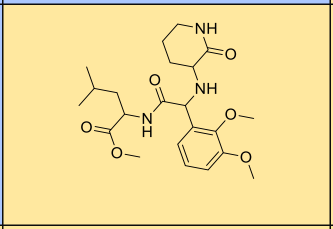	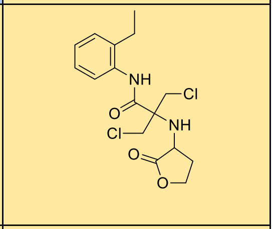
**20**	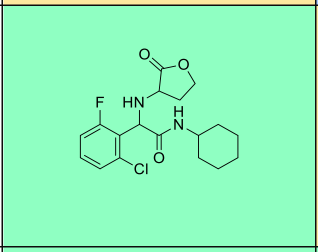	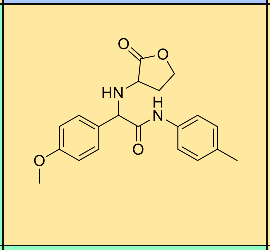	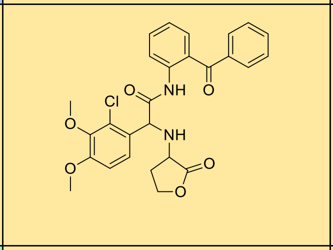	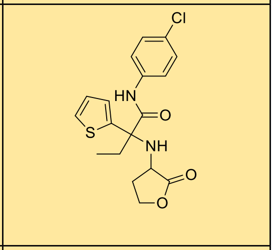
**21**	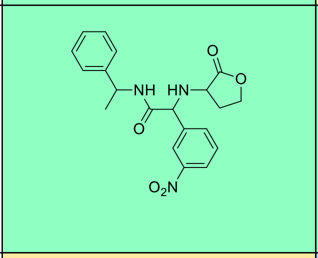	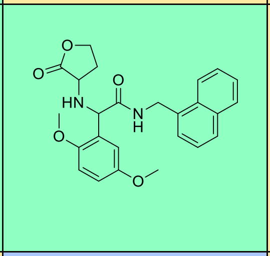	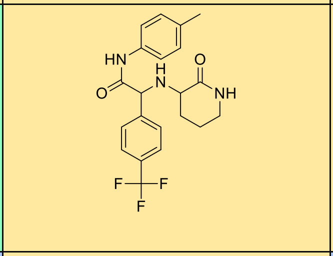	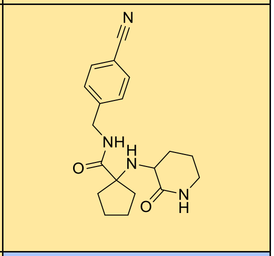
**22**	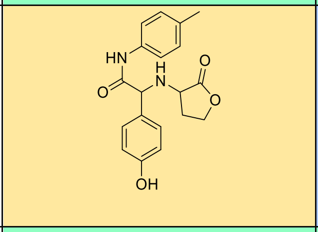	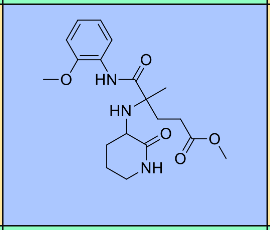	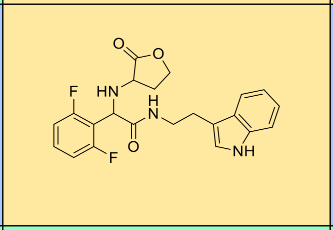	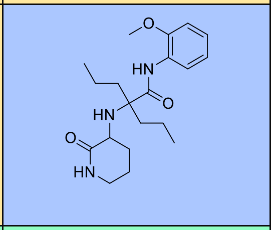
**23**	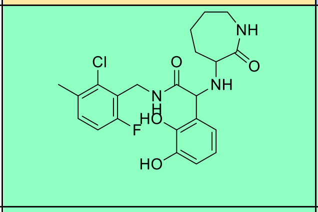	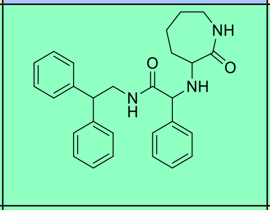	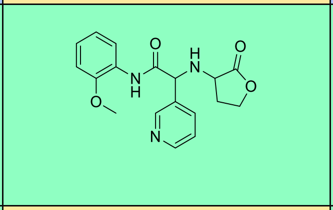	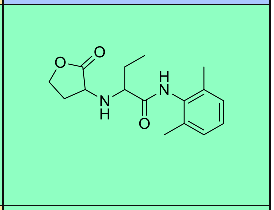
**24**	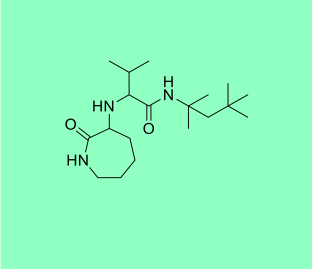	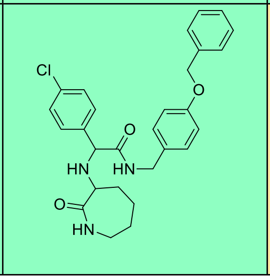	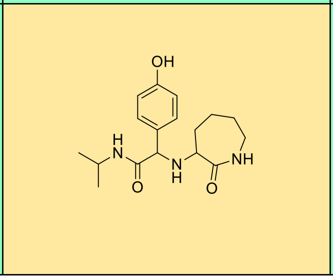	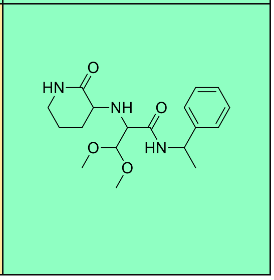

Destination plate III

	A	B	C	D
1	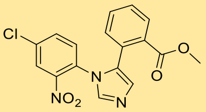	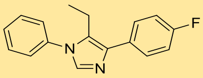	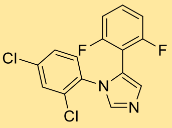	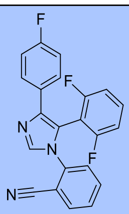
2	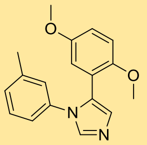	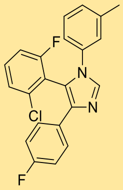	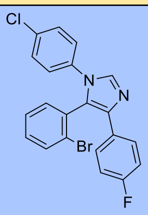	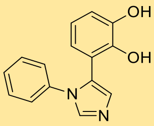
3	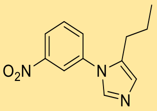	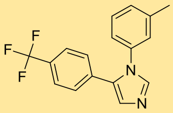	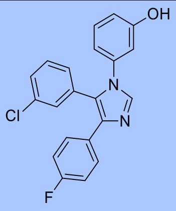	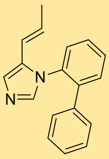
4	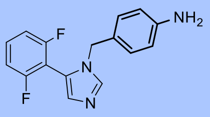	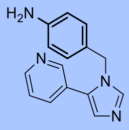	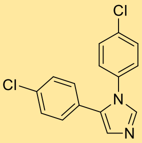	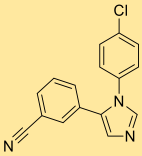
5	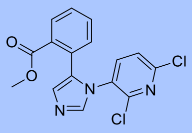	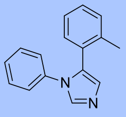	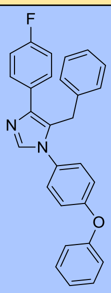	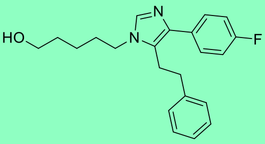
6	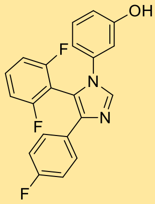	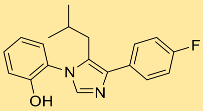	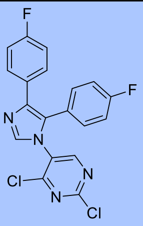	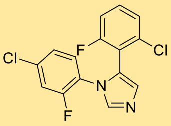
7	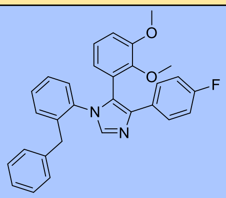	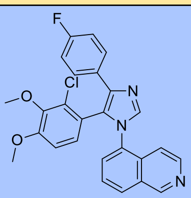	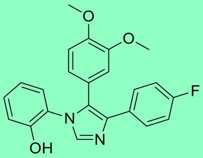	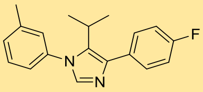

**8**	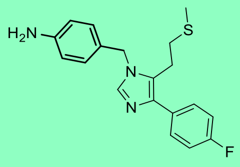	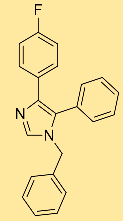	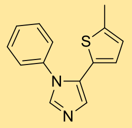	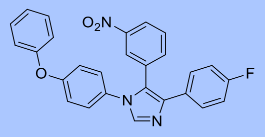
**9**	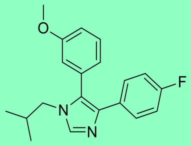	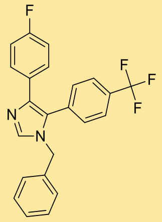	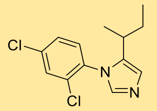	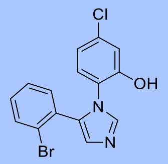
**10**	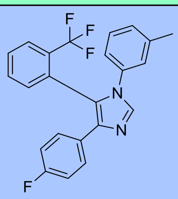	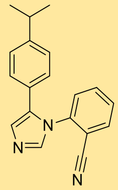	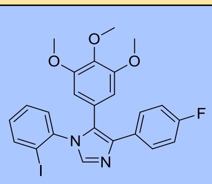	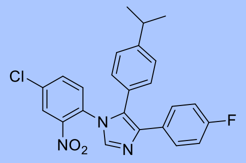
**11**	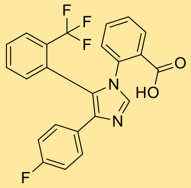	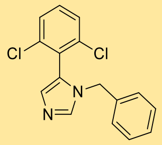	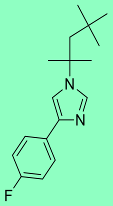	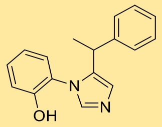
**12**	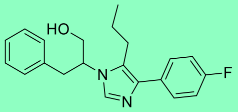	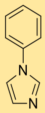	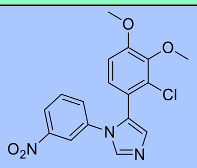	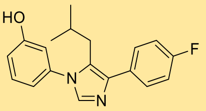
**13**	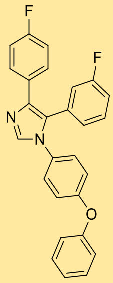	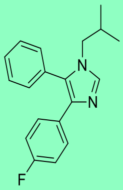	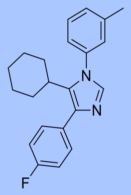	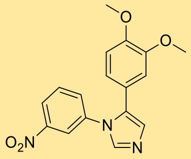
**14**	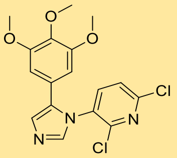	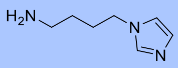	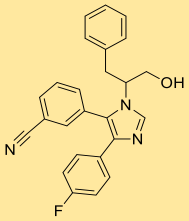	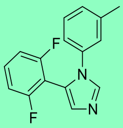

**15**	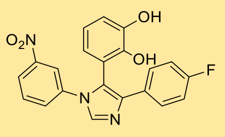	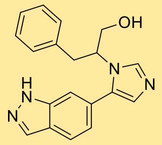	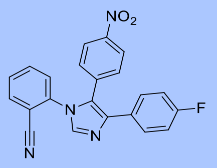	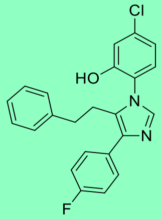
**16**	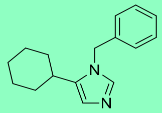	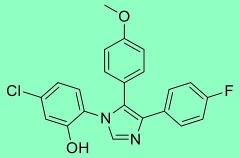	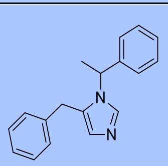	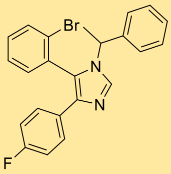
**17**	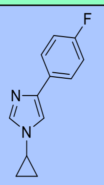	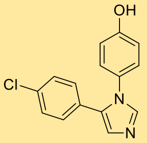	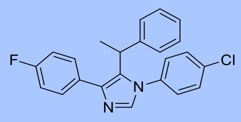	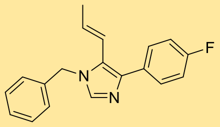
**18**	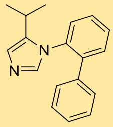	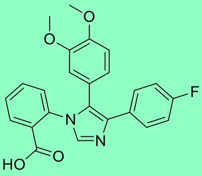	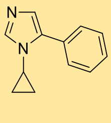	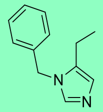
**19**	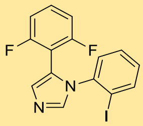	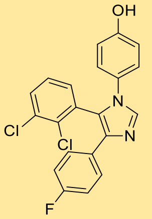	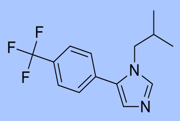	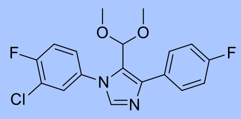
**20**	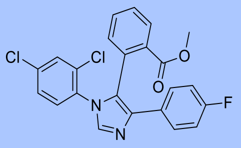	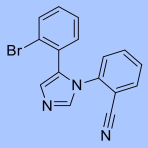	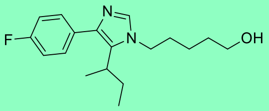	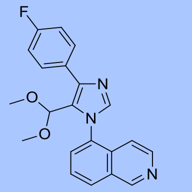
**21**	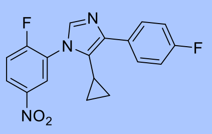	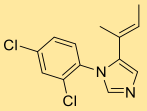	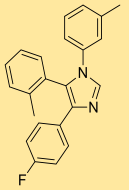	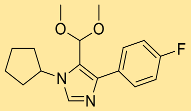
**22**	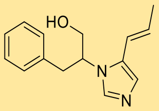	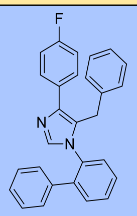	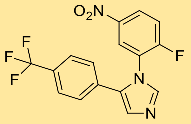	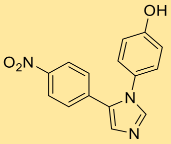

**23**	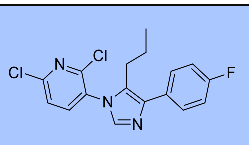	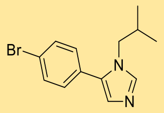	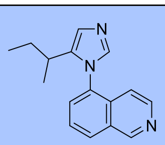	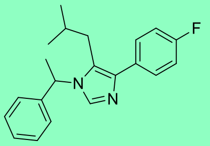
**24**	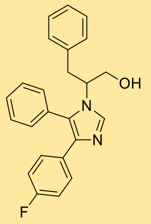	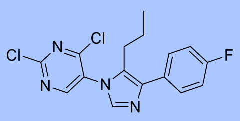	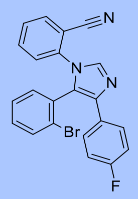	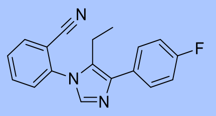

	E	F	G	H
**1**	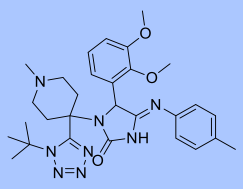	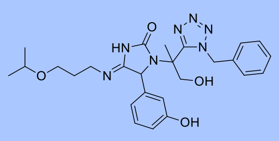	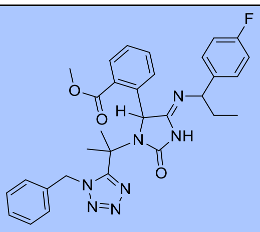	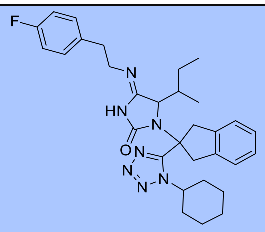
**2**	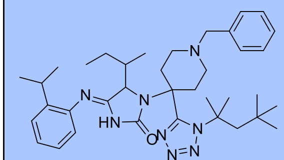	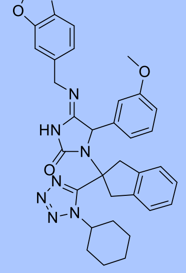	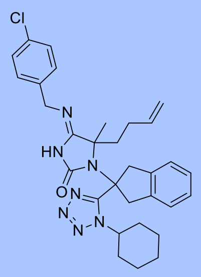	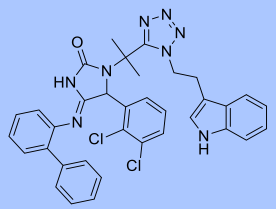
**3**	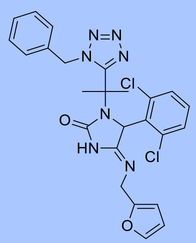	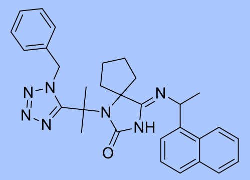	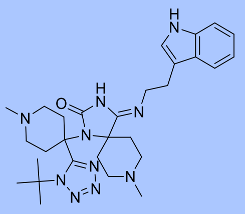	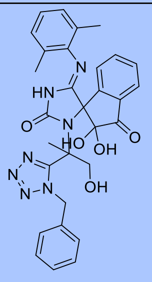
**4**	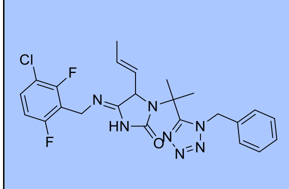	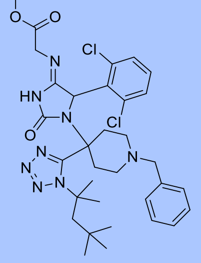	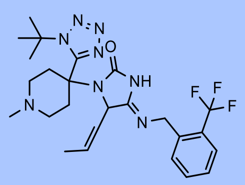	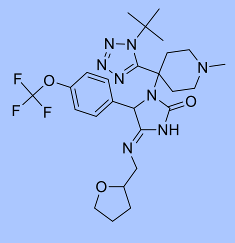

**5**	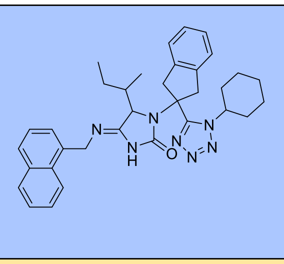	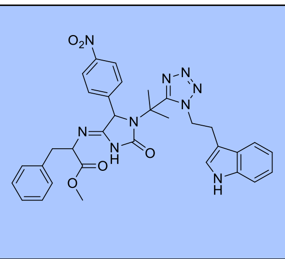	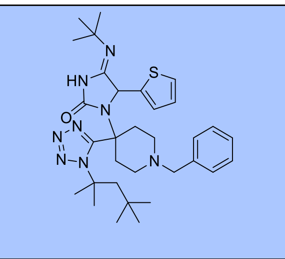	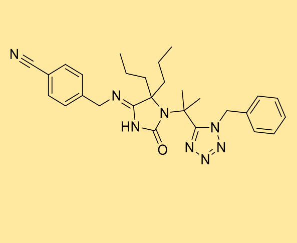
**6**	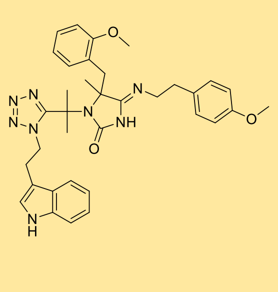	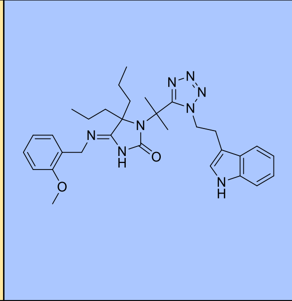	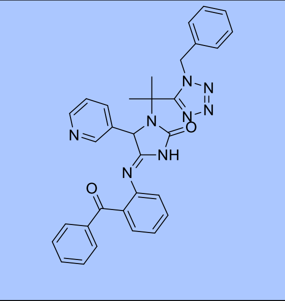	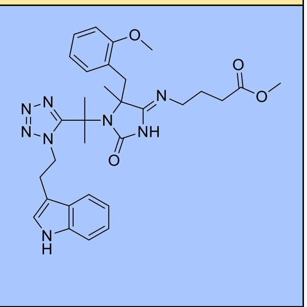
**7**	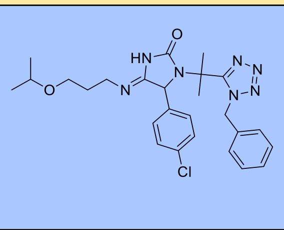	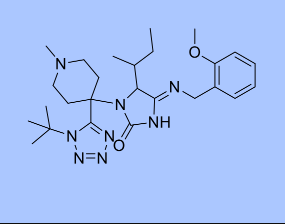	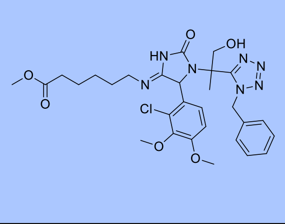	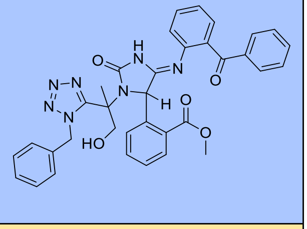
**8**	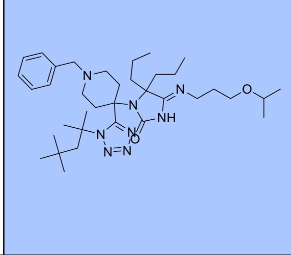	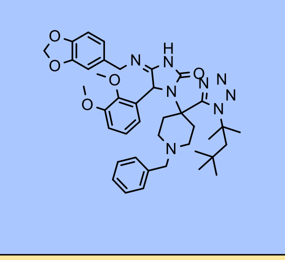	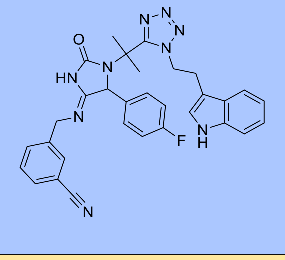	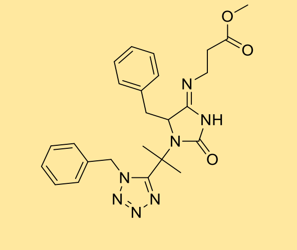
**9**	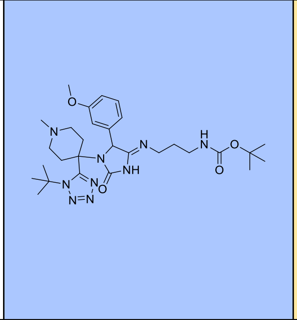	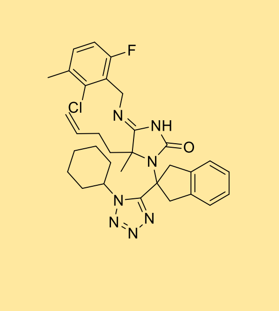	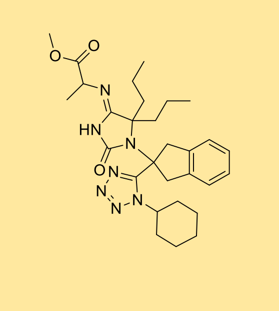	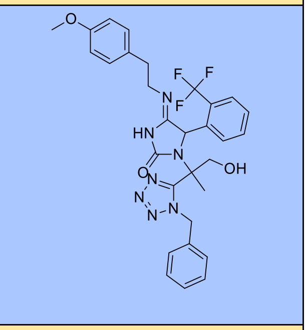
**10**	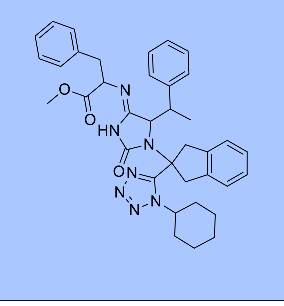	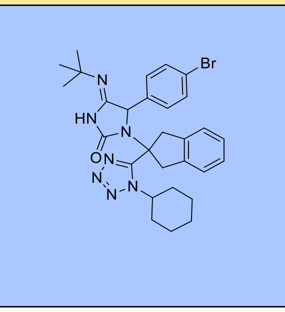	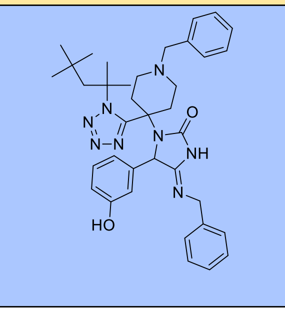	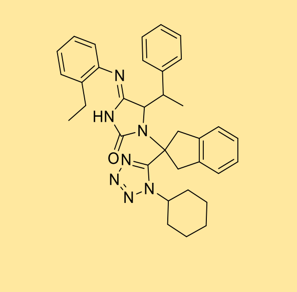

**11**	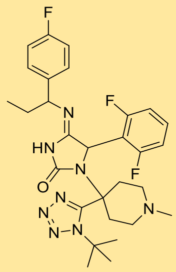	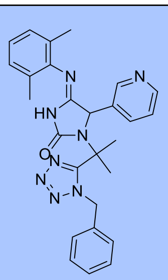	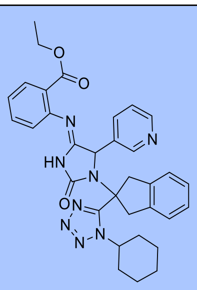	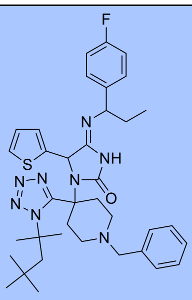
**12**	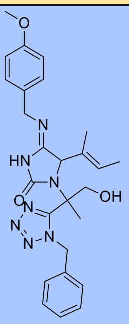	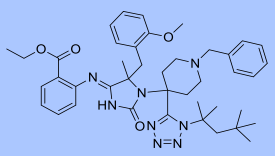	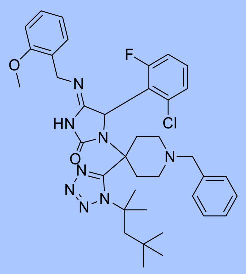	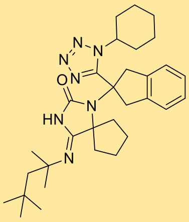
**13**	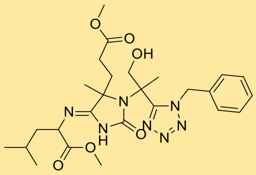	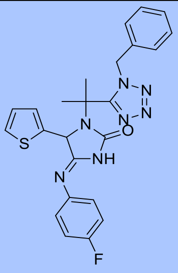	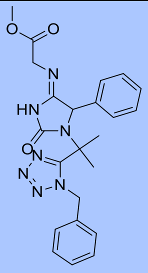	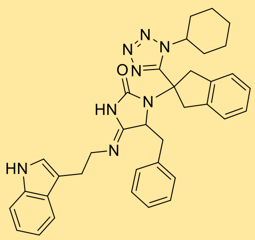
**14**	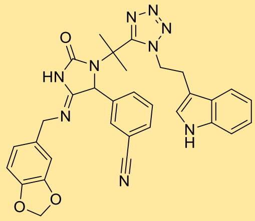	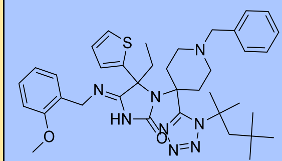	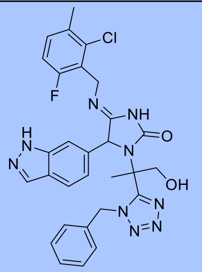	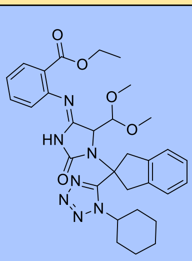
**15**	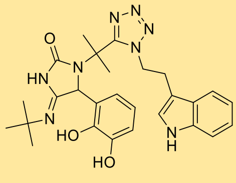	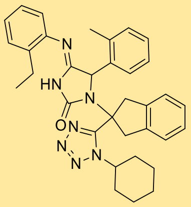	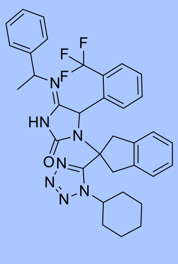	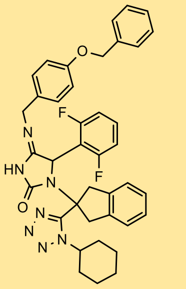
**16**	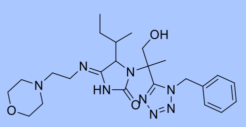	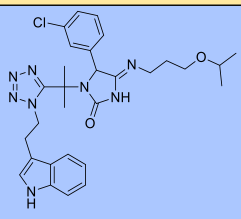	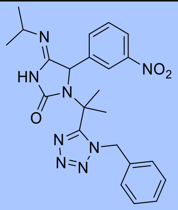	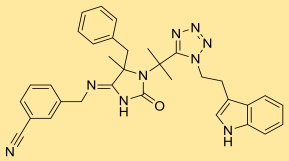

**17**	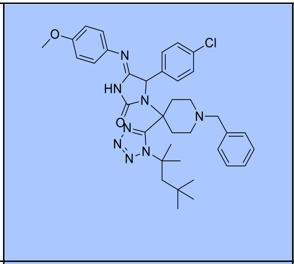	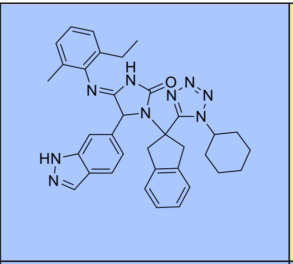	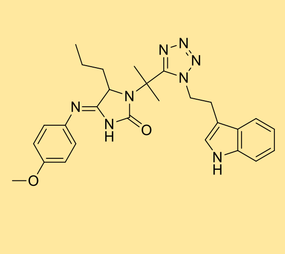	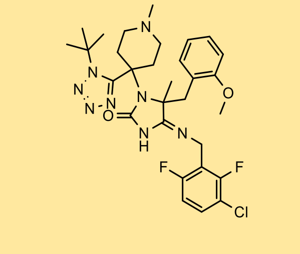
**18**	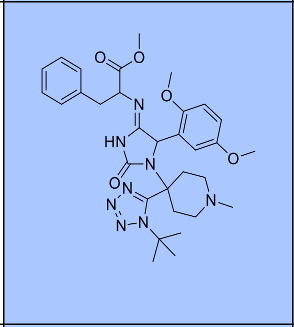	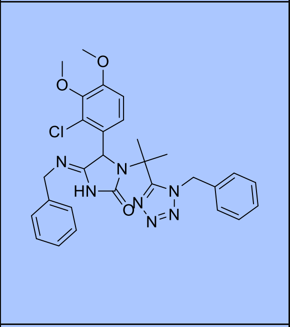	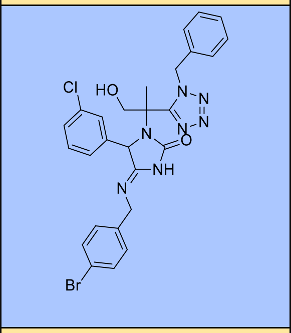	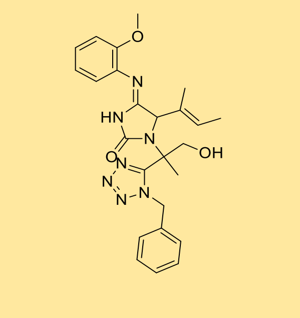
**19**	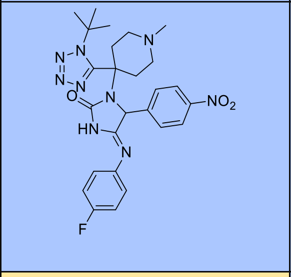	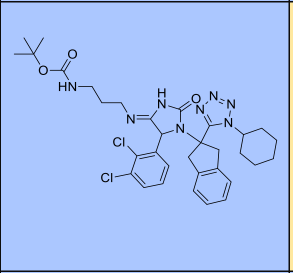	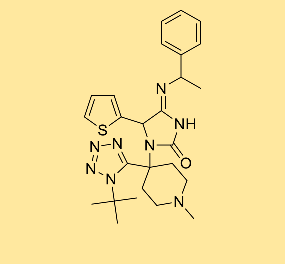	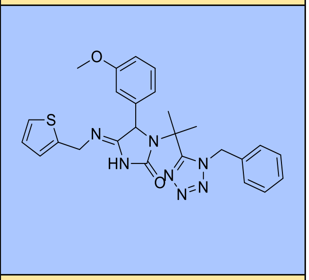
**20**	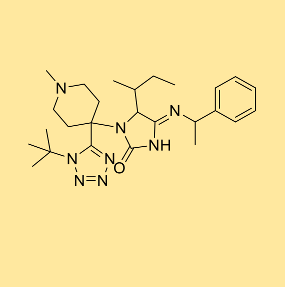	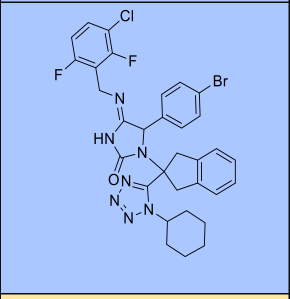	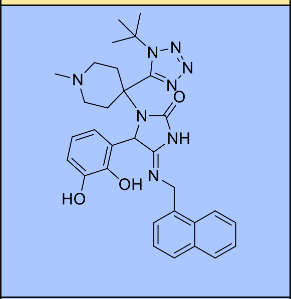	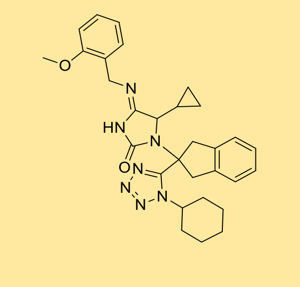
**21**	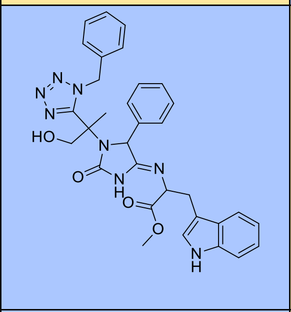	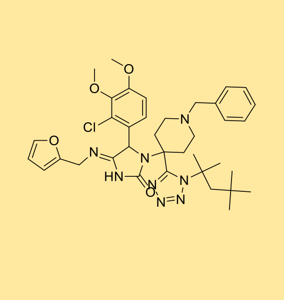	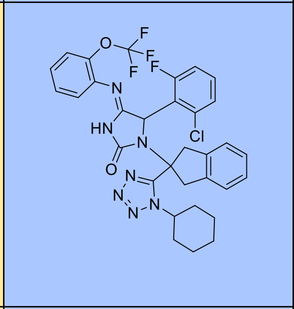	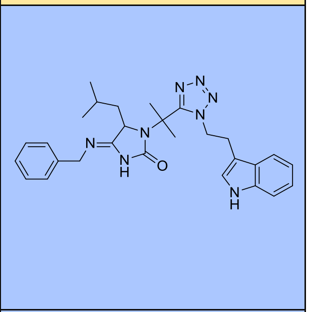
**22**	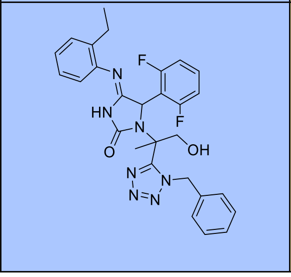	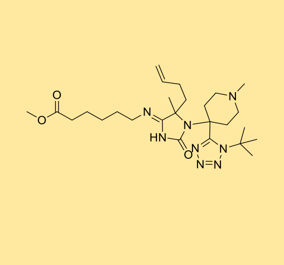	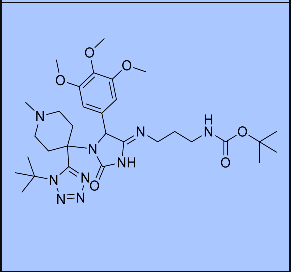	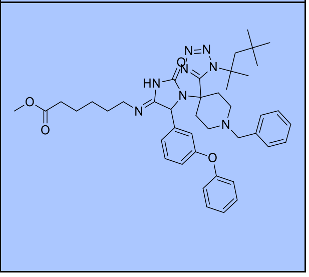

**23**	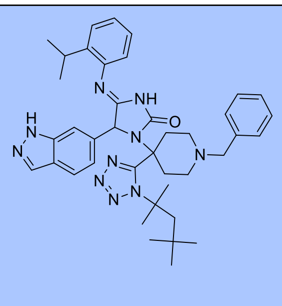	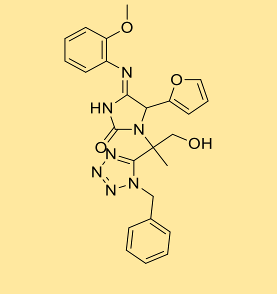	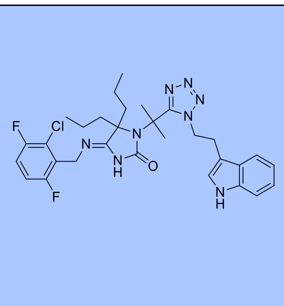	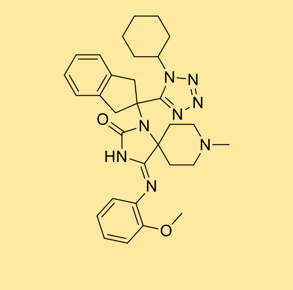
**24**	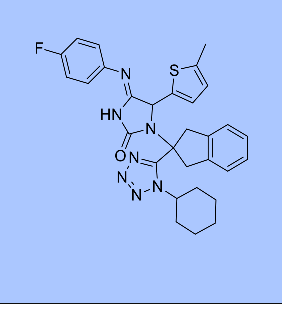	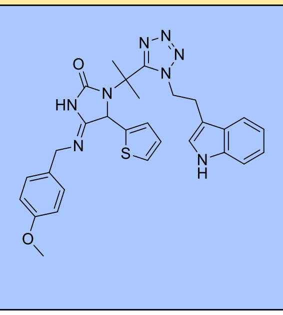	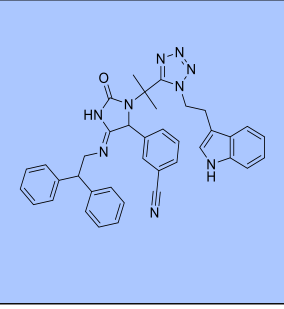	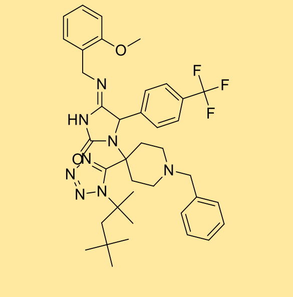

	**I**	**J**	**K**	**L**
**1**	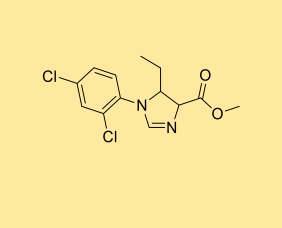	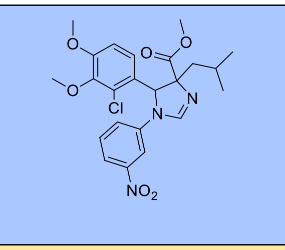	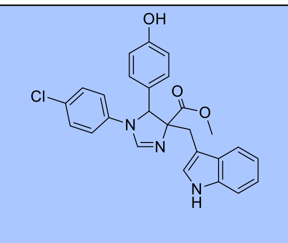	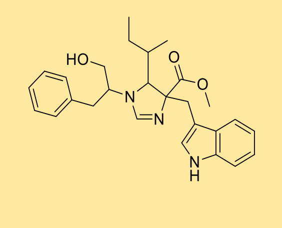
**2**	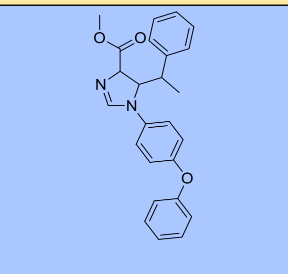	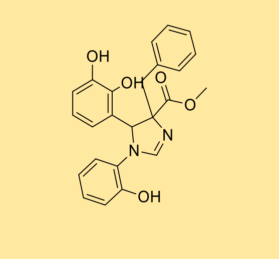	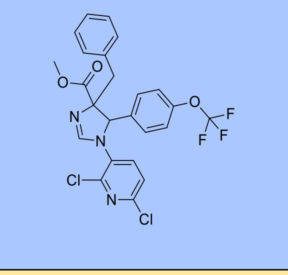	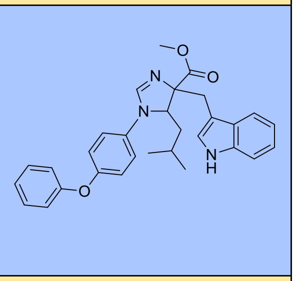
**3**	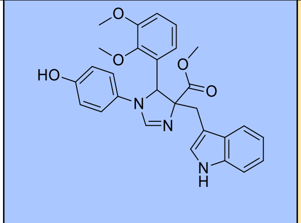	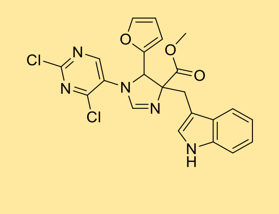	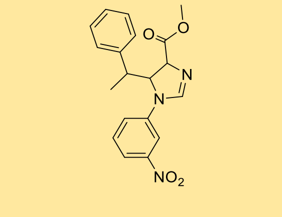	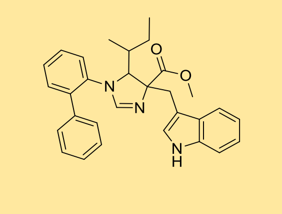
**4**	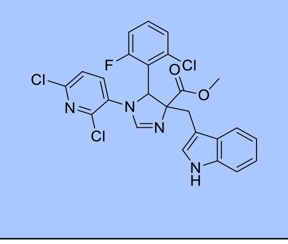	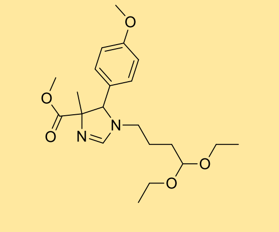	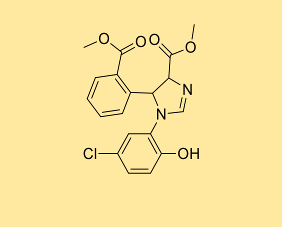	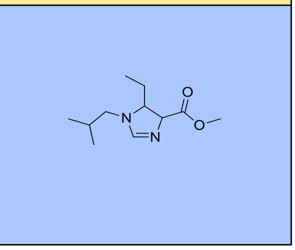

**5**	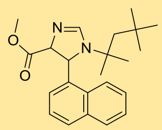	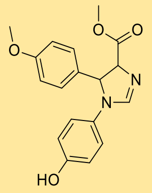	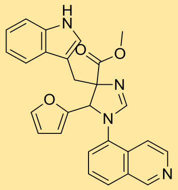	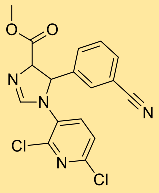
**6**	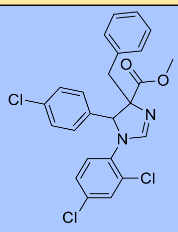	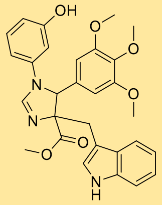	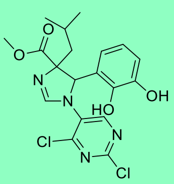	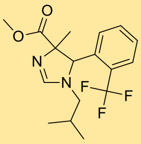
**7**	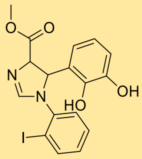	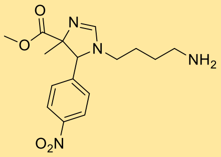	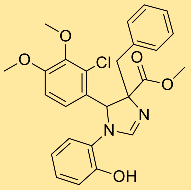	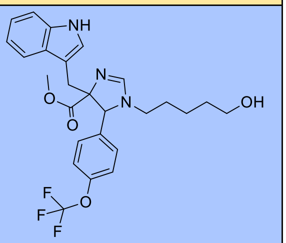
**8**	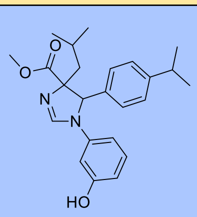	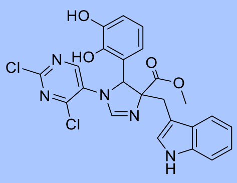	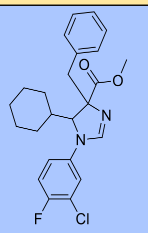	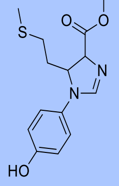
**9**	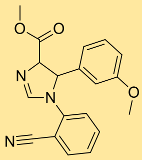	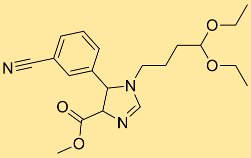	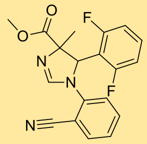	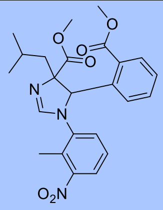
**10**	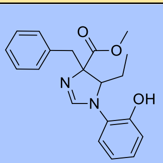	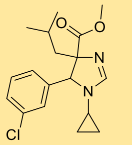	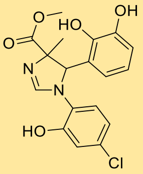	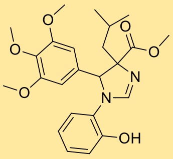
**11**	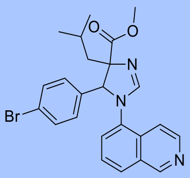	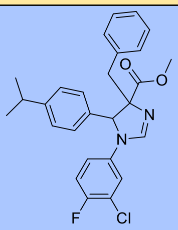	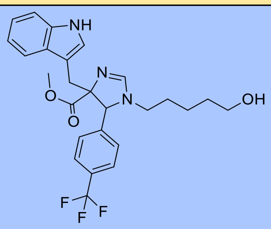	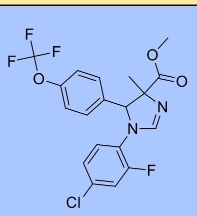

**12**	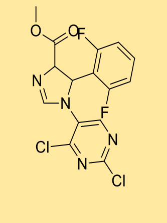	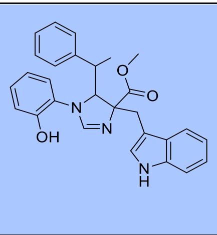	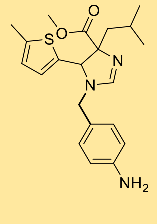	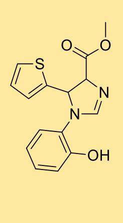
**13**	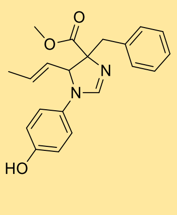	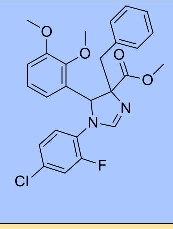	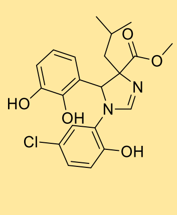	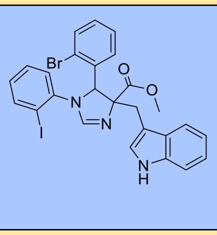
**14**	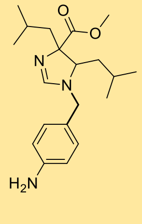	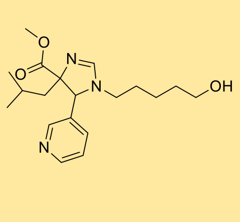	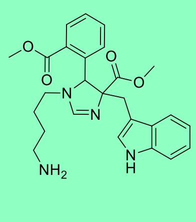	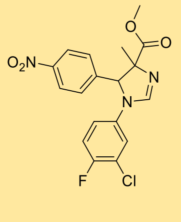
**15**	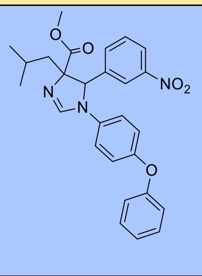	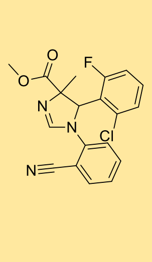	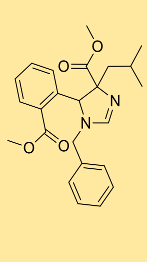	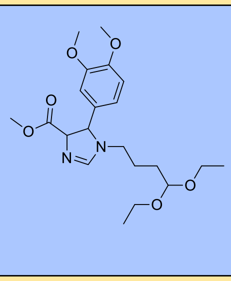
**16**	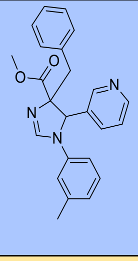	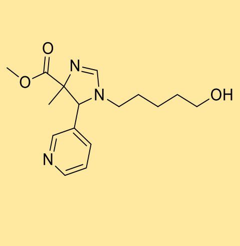	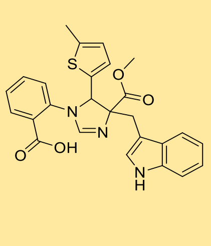	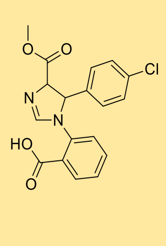
**17**	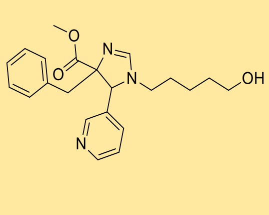	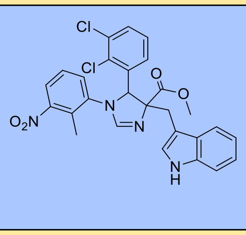	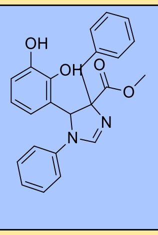	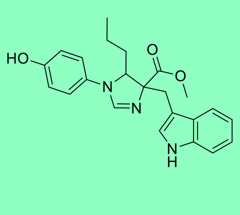
**18**	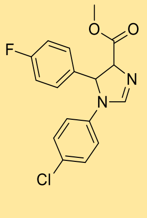	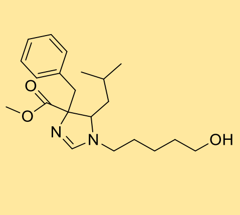	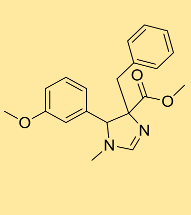	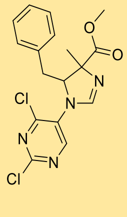

**19**	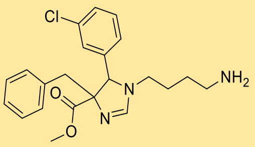	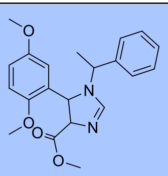	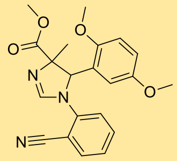	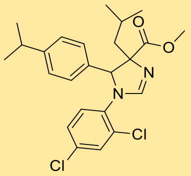
**20**	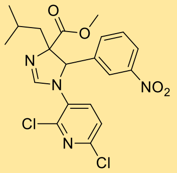	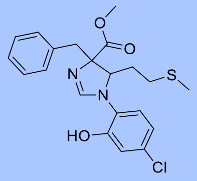	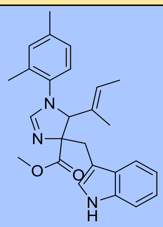	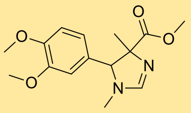
**21**	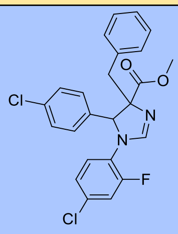	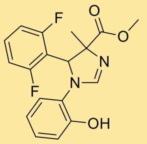	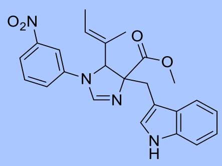	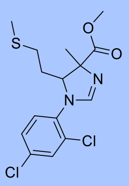
**22**	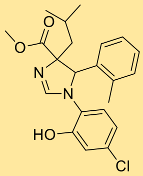	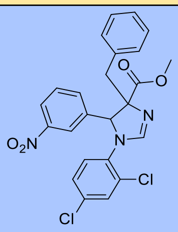	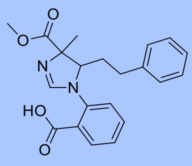	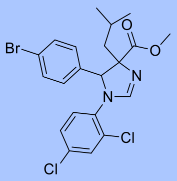
**23**	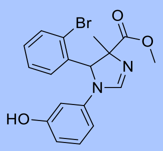	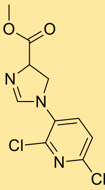	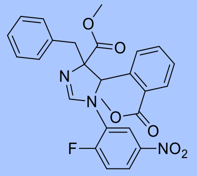	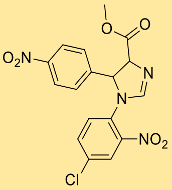
**24**	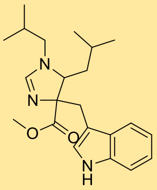	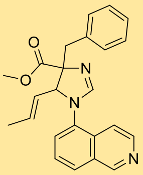	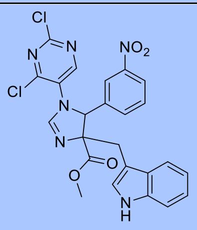	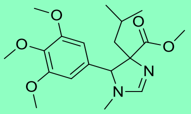

	**M**	**N**	**O**	**P**
**1**	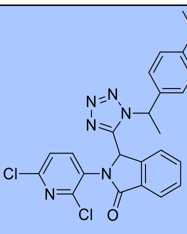	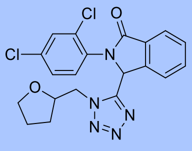	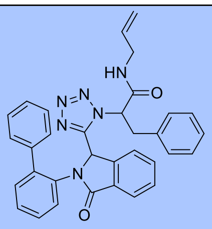	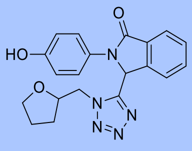

**2**	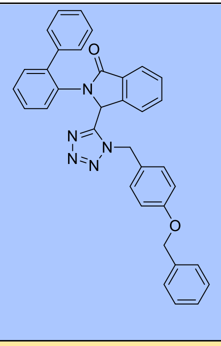	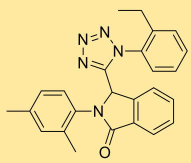	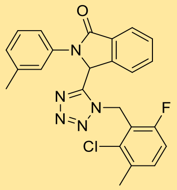	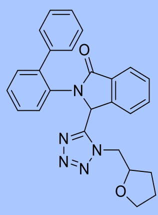
**3**	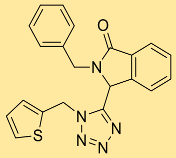	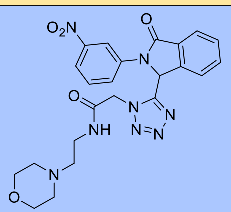	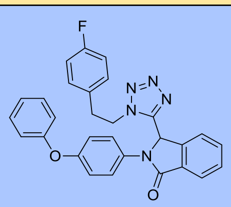	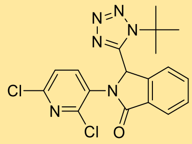
**4**	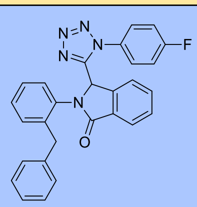	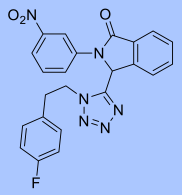	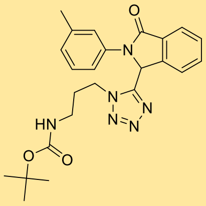	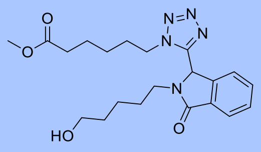
**5**	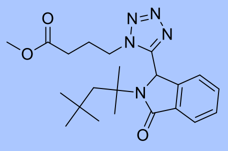	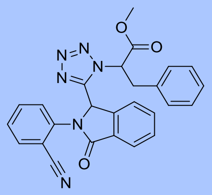	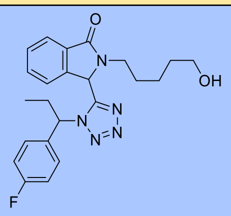	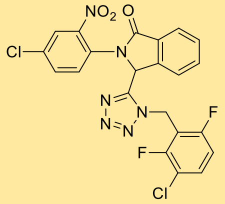
**6**	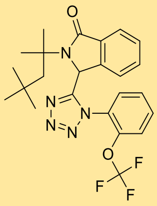	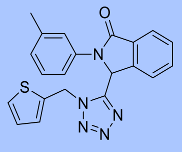	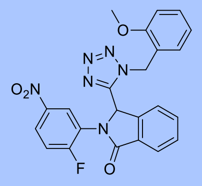	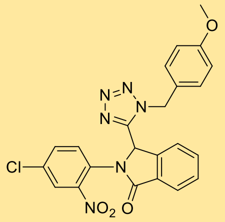
**7**	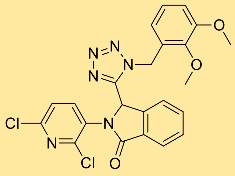	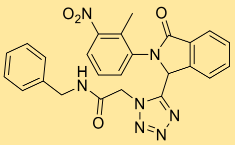	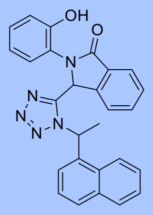	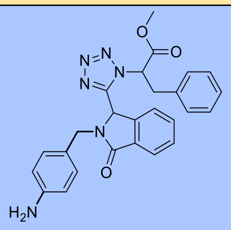
**8**	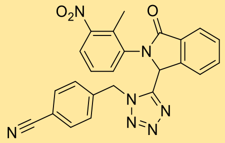	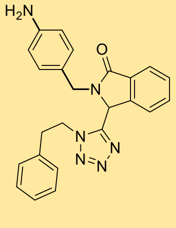	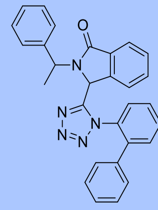	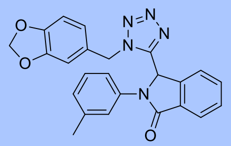

**9**	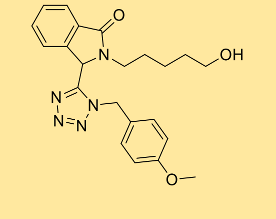	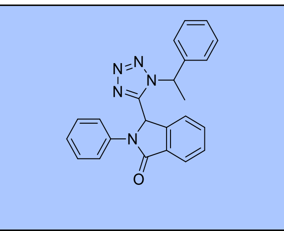	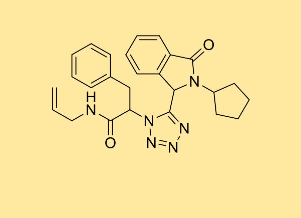	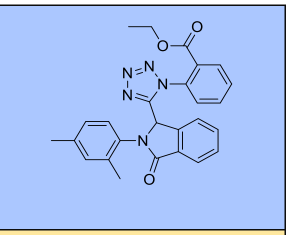
**10**	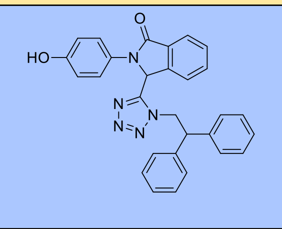	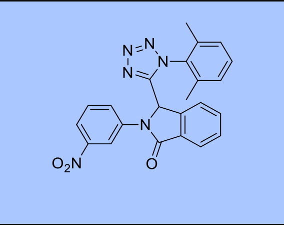	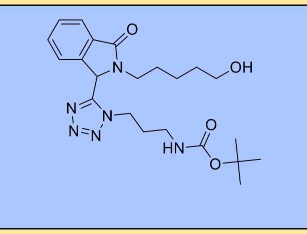	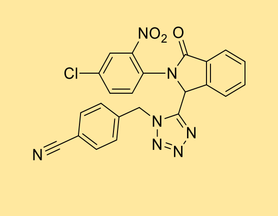
**11**	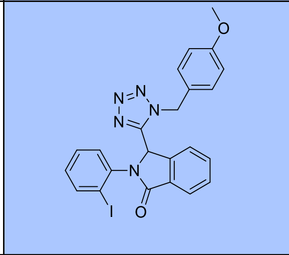	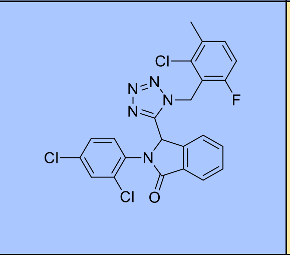	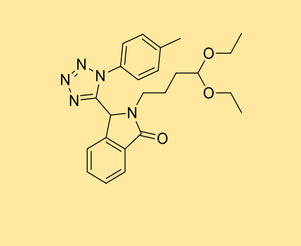	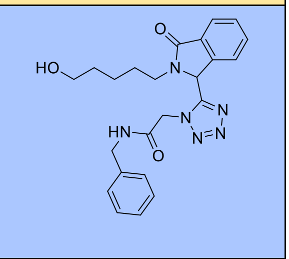
**12**	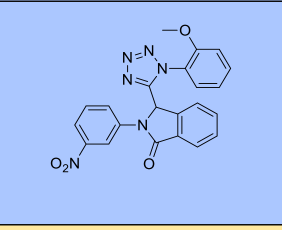	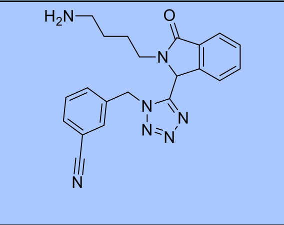	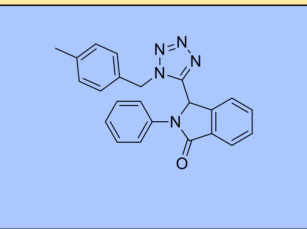	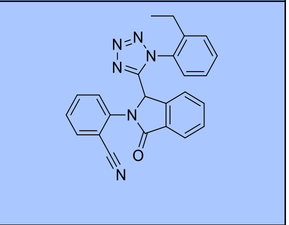
**13**	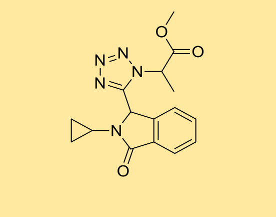	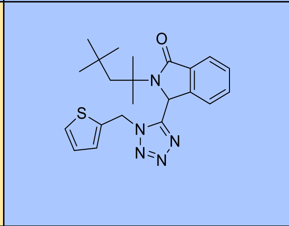	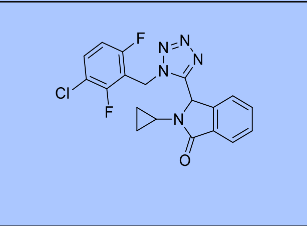	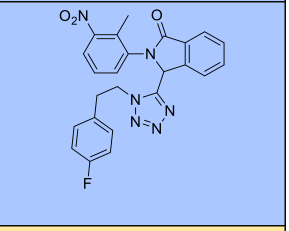
**14**	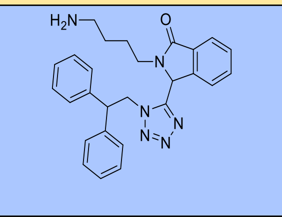	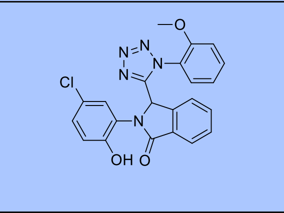	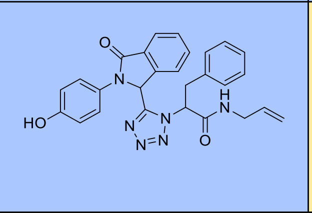	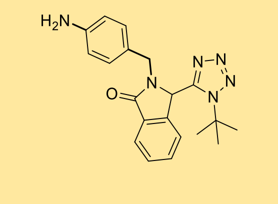
**15**	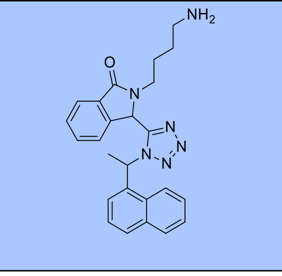	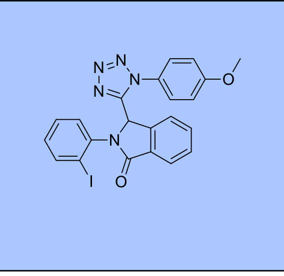	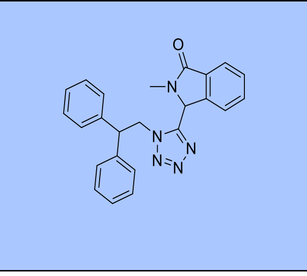	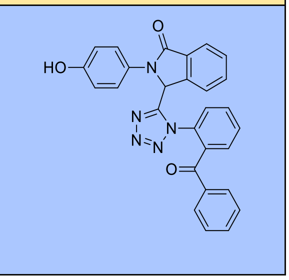

**16**	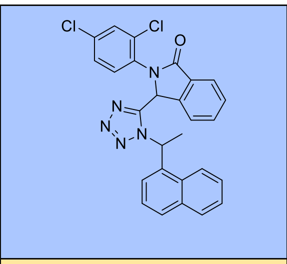	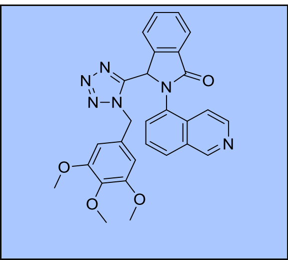	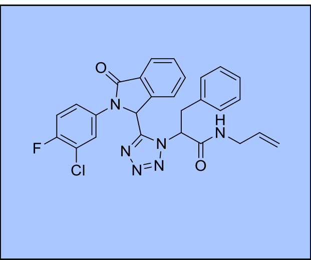	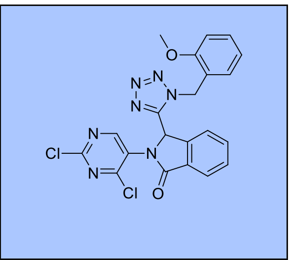
**17**	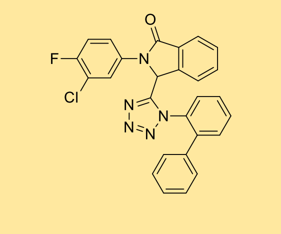	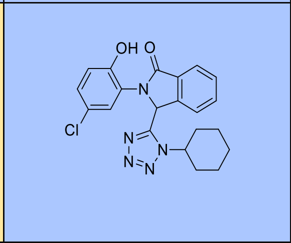	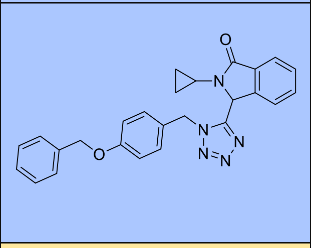	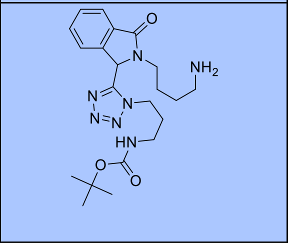
**18**	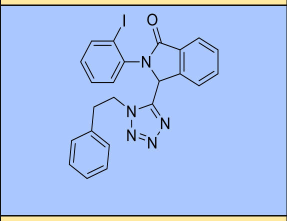	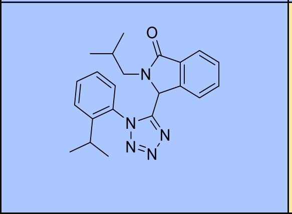	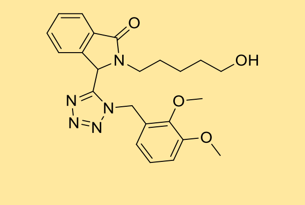	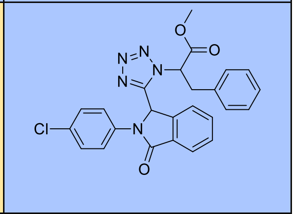
**19**	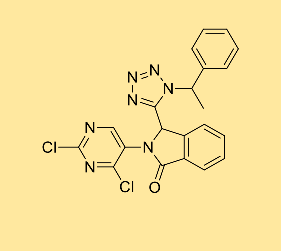	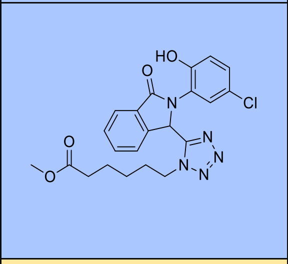	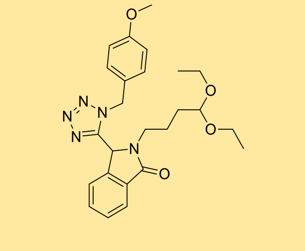	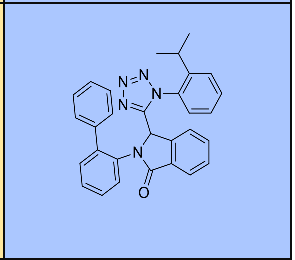
**20**	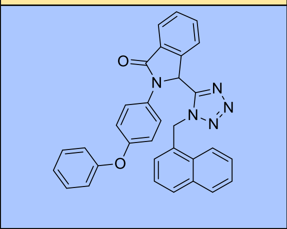	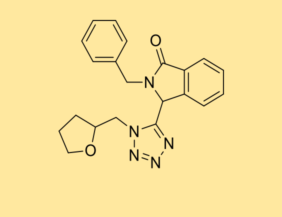	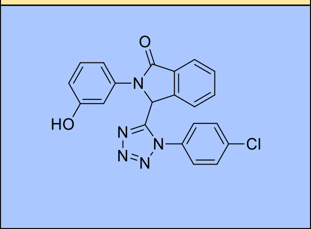	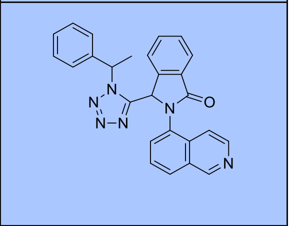
**21**	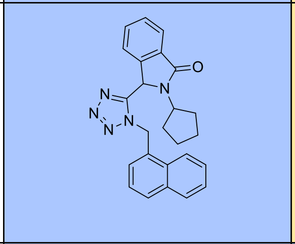	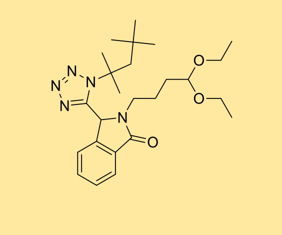	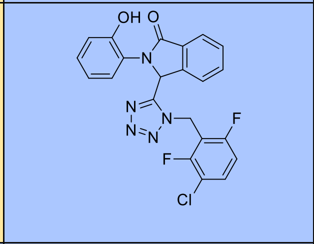	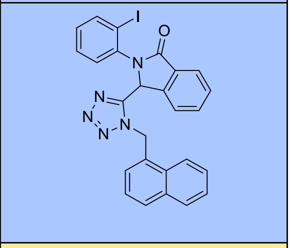
**22**	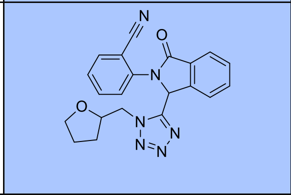	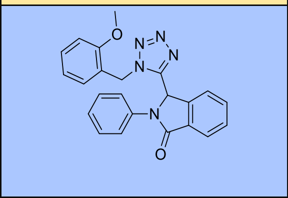	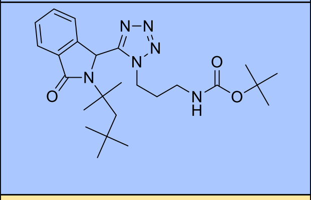	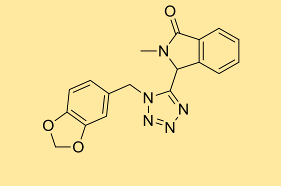
**23**	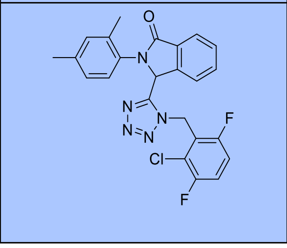	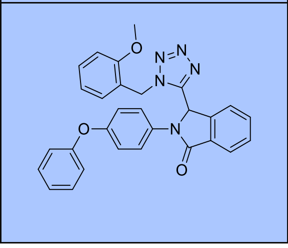	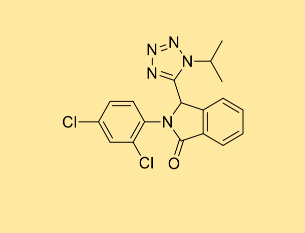	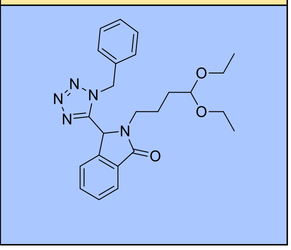

**24**	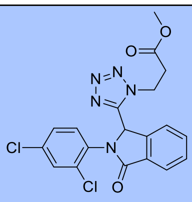	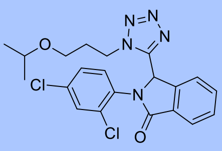	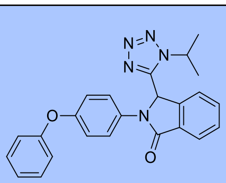	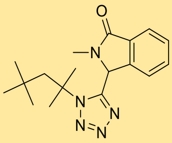

Destination plate IV

	A	B	C	D
**1**	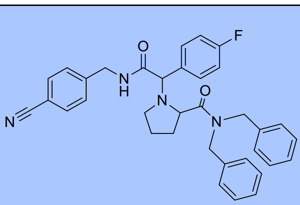	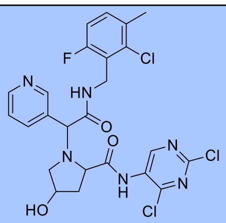	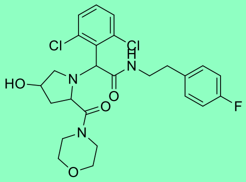	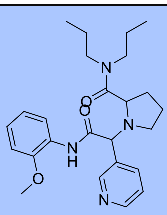
**2**	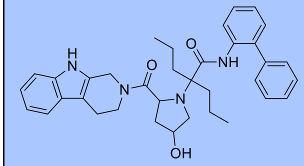	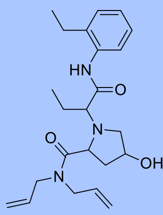	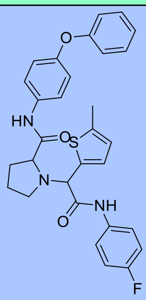	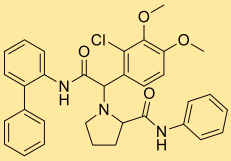
**3**	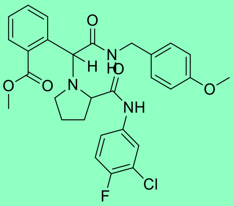	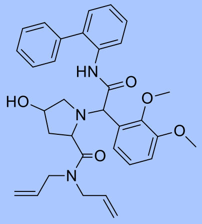	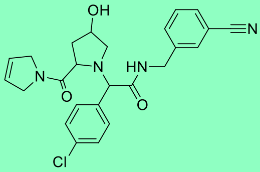	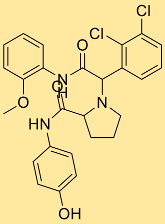
**4**	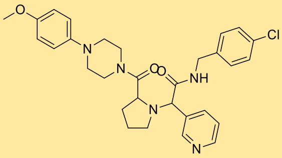	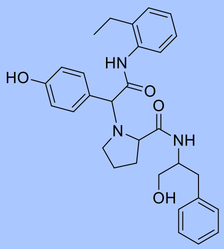	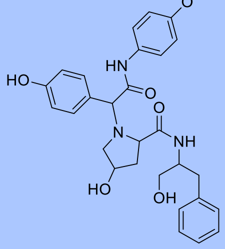	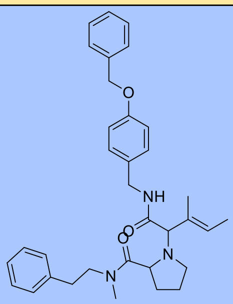
**5**	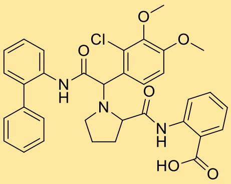	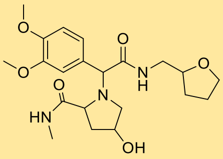	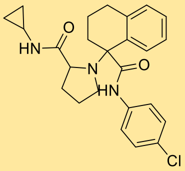	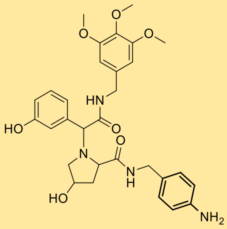

**6**	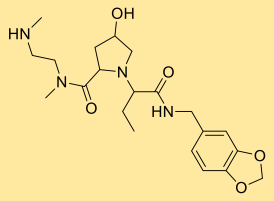	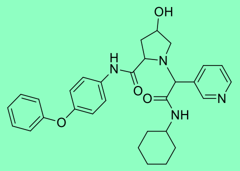	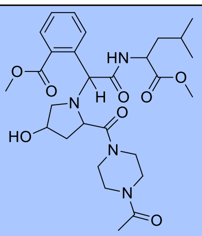	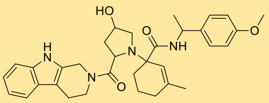
**7**	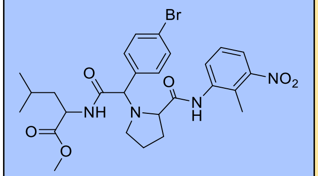	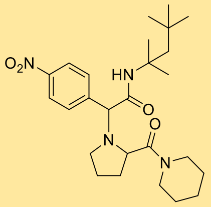	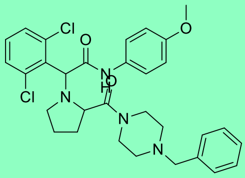	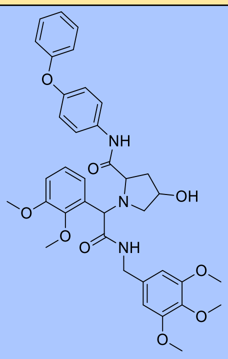
**8**	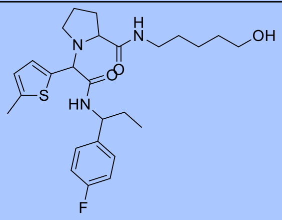	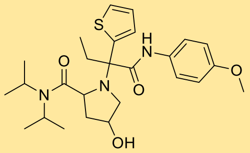	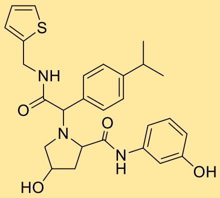	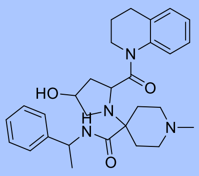
**9**	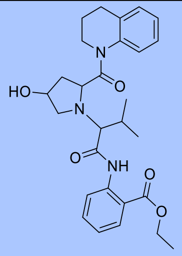	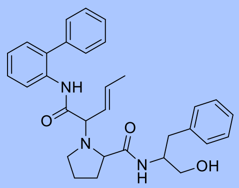	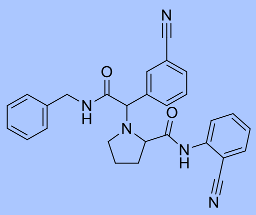	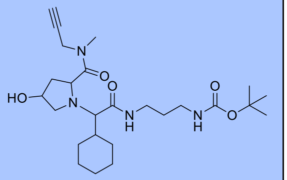
**10**	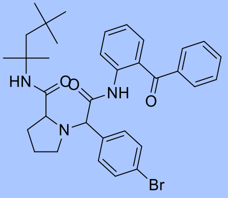	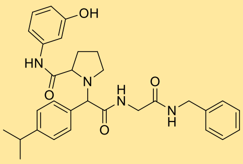	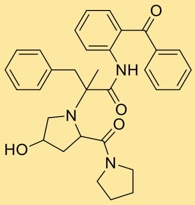	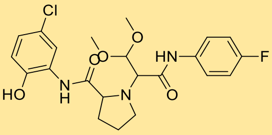
**11**	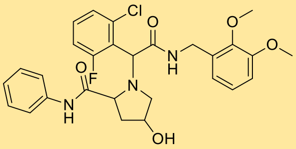	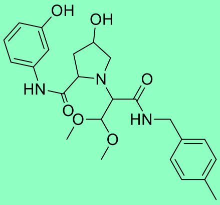	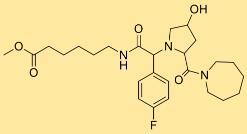	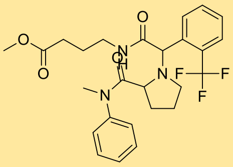

**12**	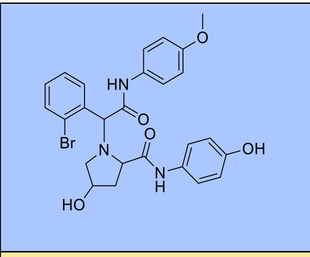	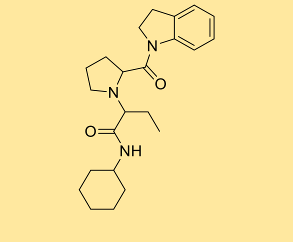	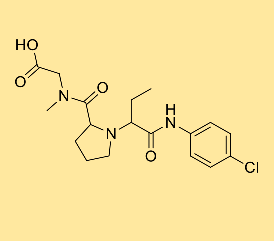	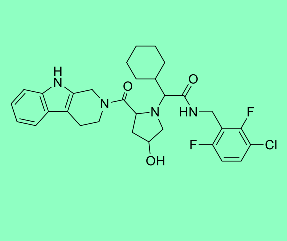
**13**	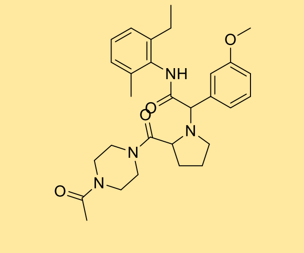	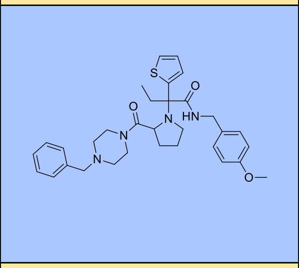	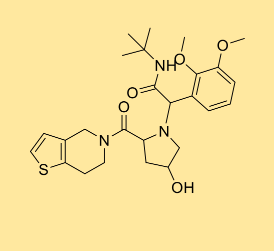	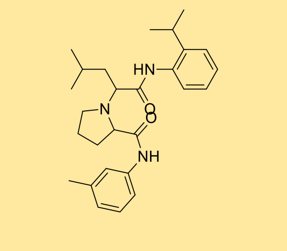
**14**	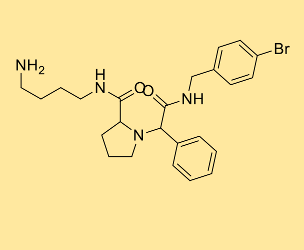	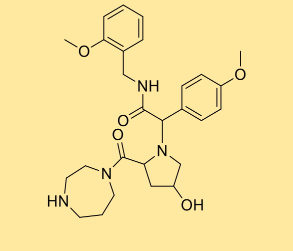	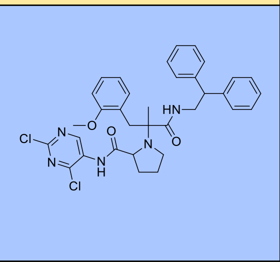	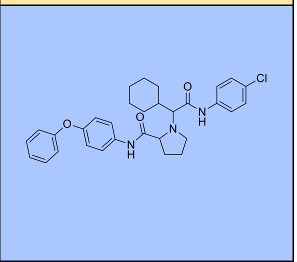
**15**	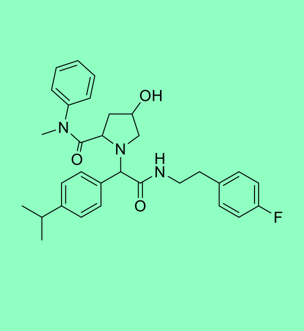	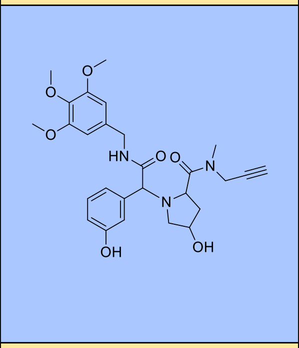	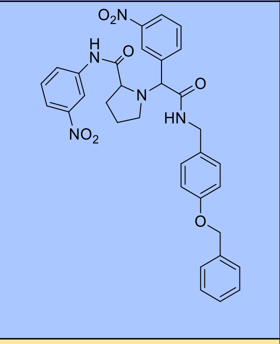	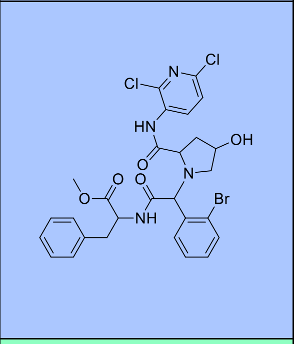
**16**	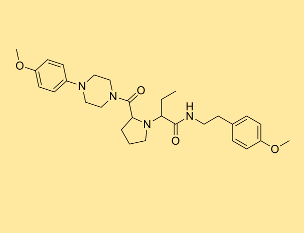	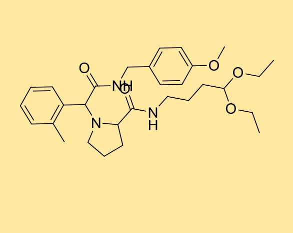	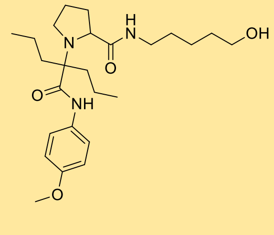	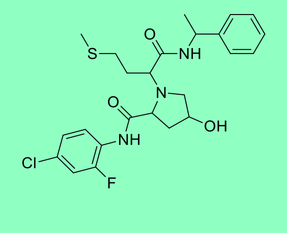
**17**	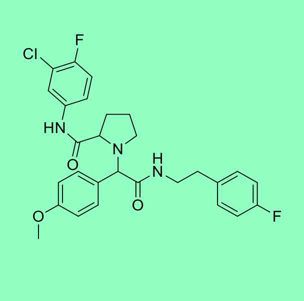	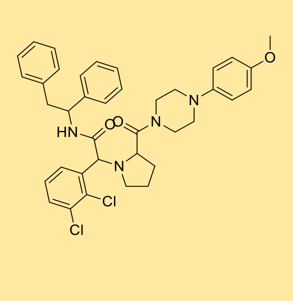	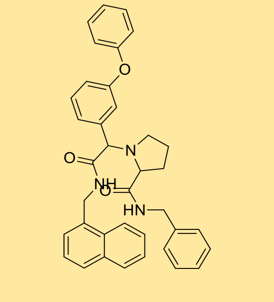	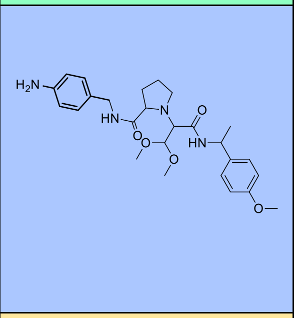
**18**	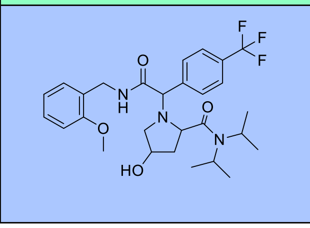	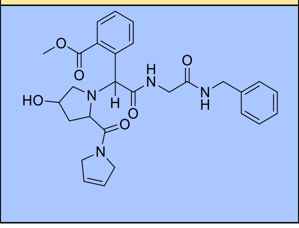	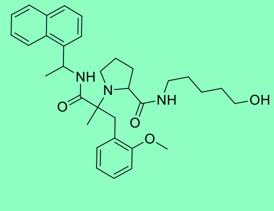	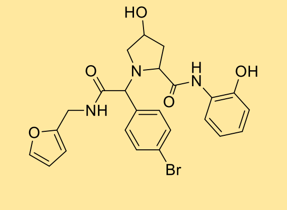

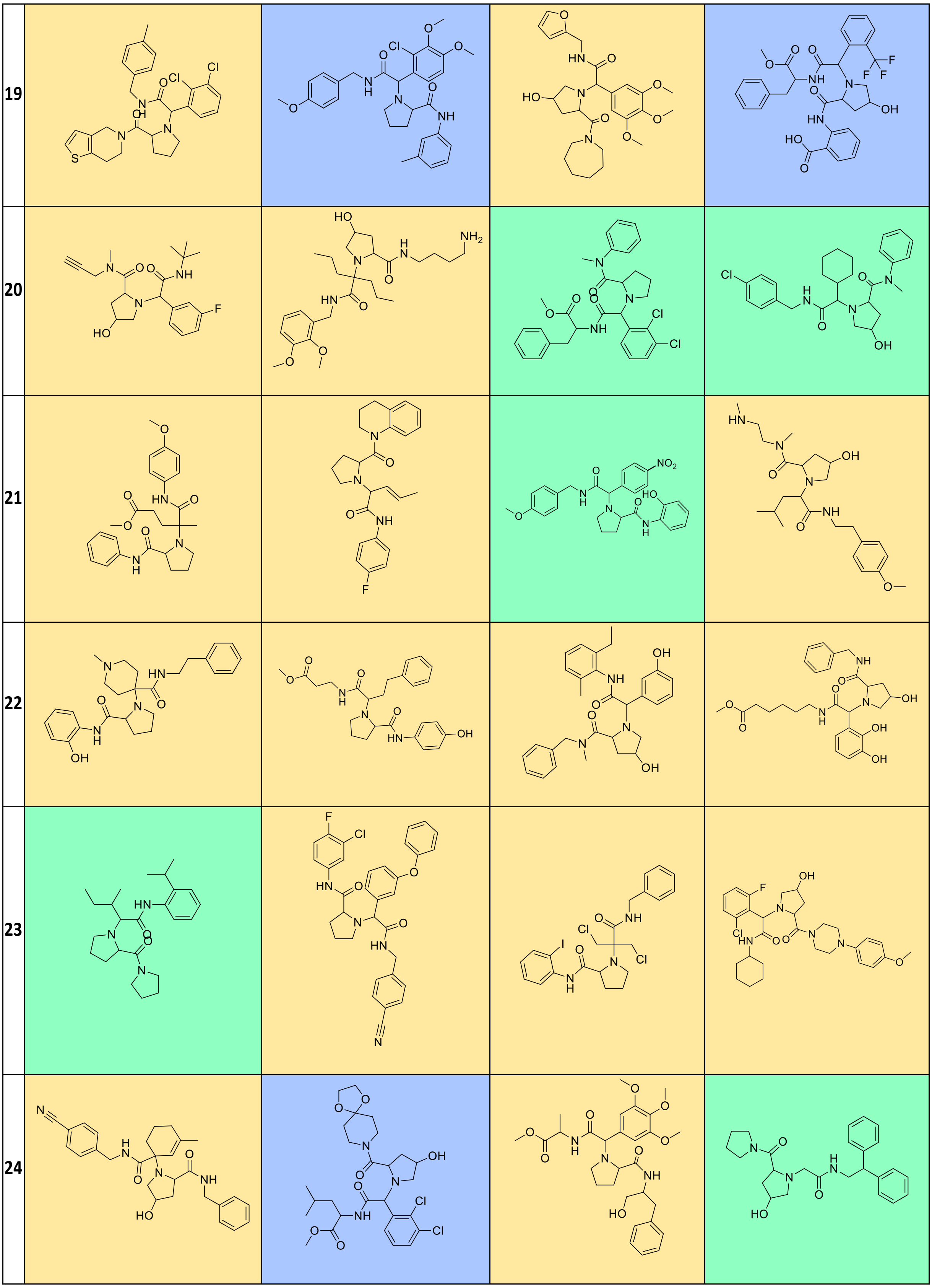
19
20
21
22
23
24

	E	F	G	H
**1**	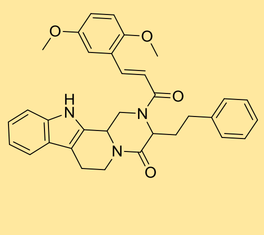	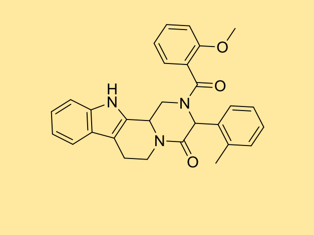	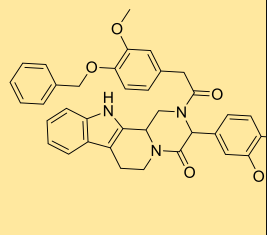	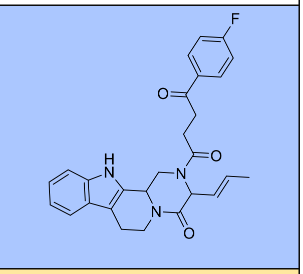
**2**	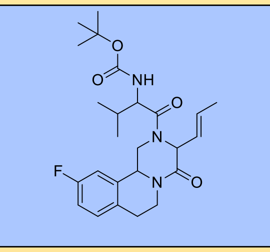	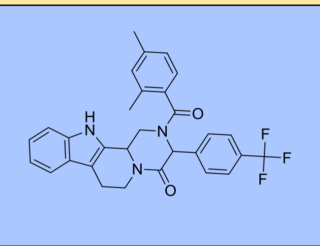	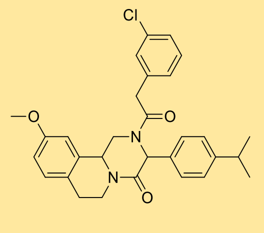	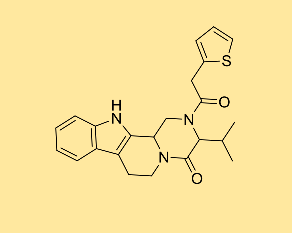
**3**	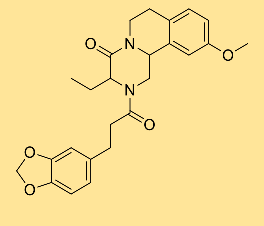	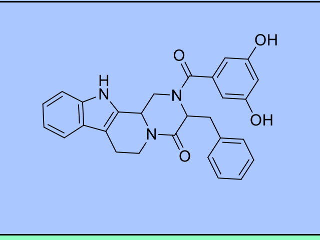	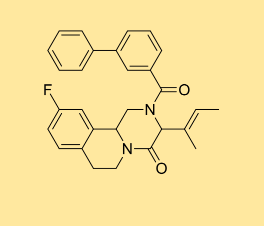	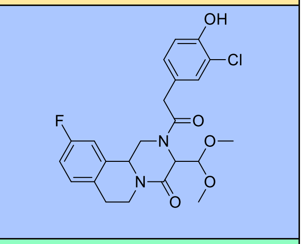
**4**	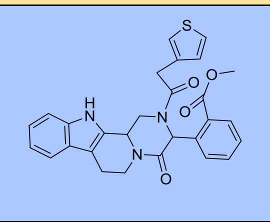	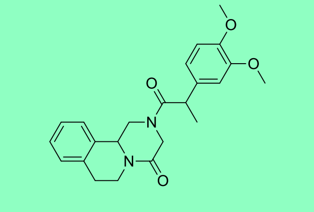	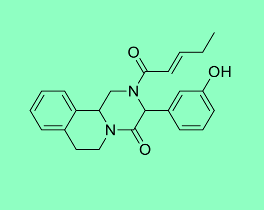	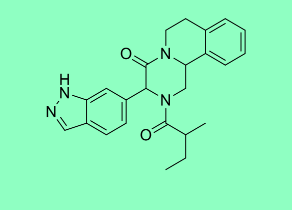
**5**	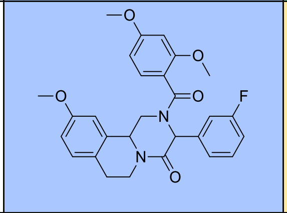	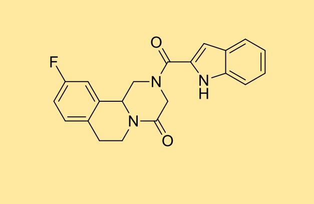	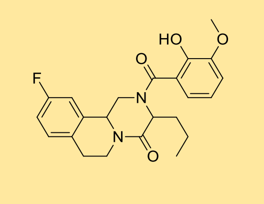	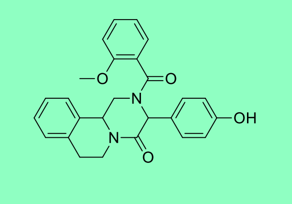
**6**	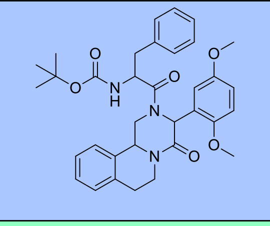	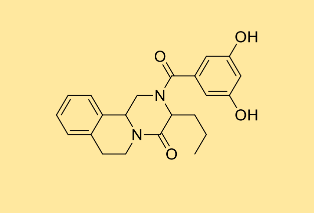	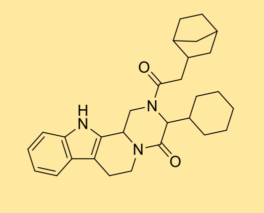	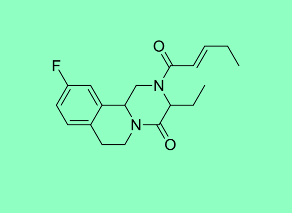
**7**	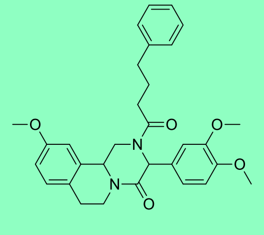	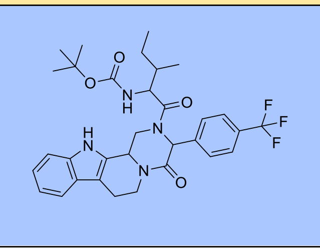	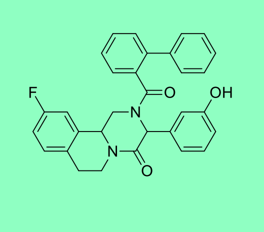	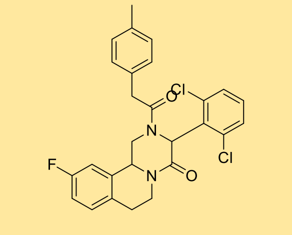

**8**	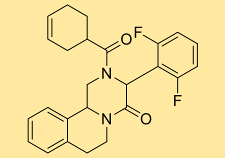	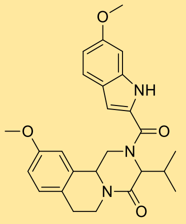	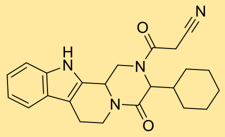	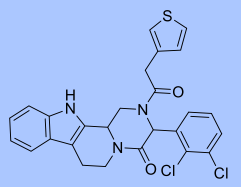
**9**	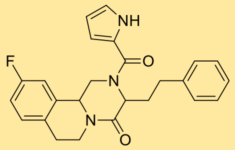	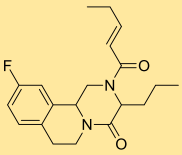	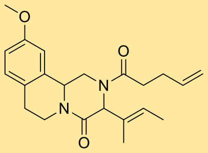	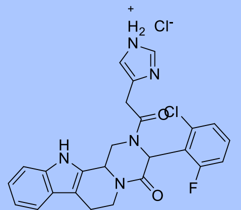
**10**	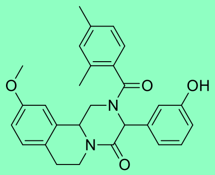	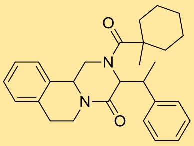	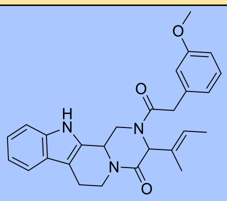	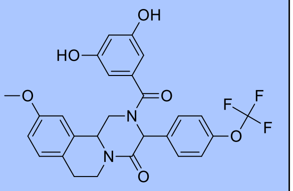
**11**	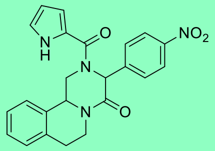	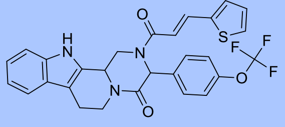	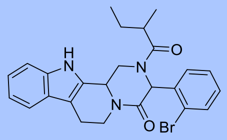	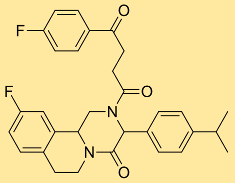
**12**	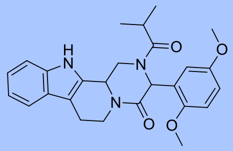	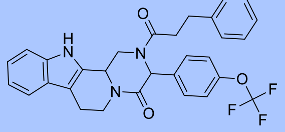	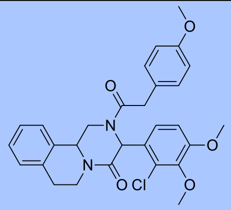	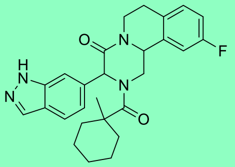
**13**	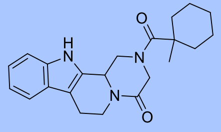	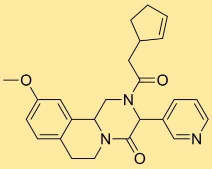	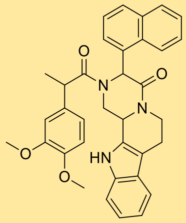	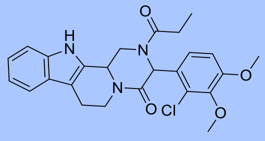
**14**	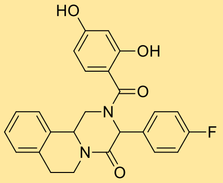	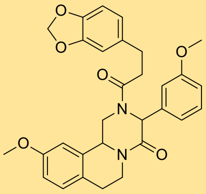	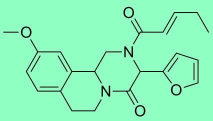	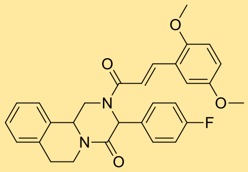

**15**	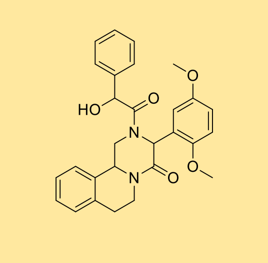	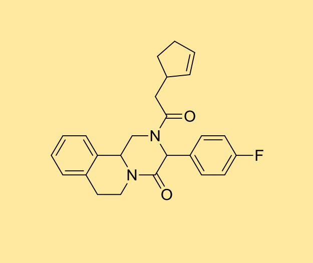	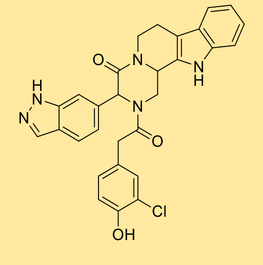	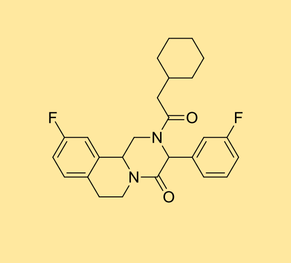
**16**	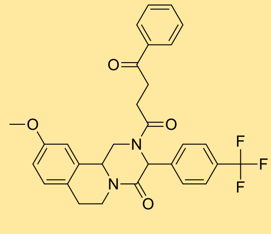	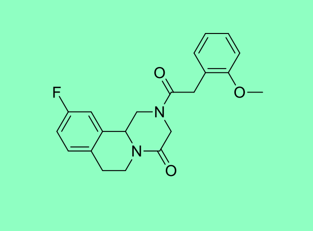	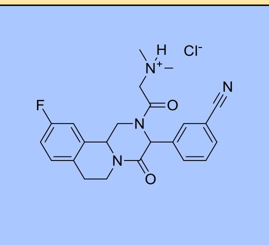	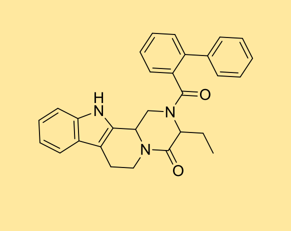
**17**	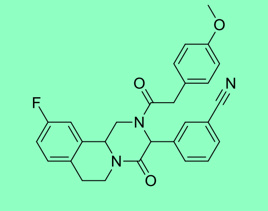	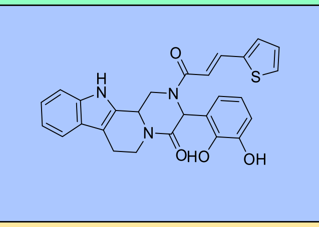	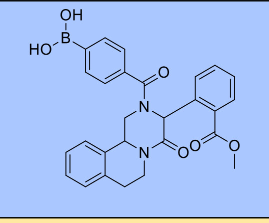	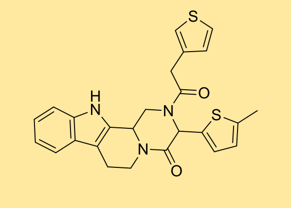
**18**	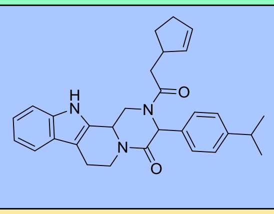	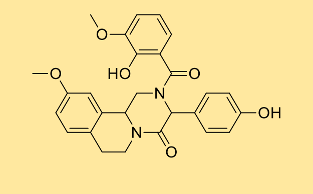	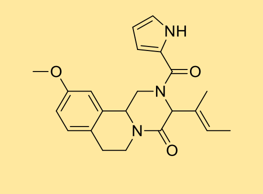	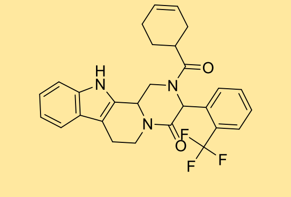
**19**	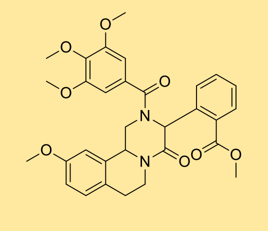	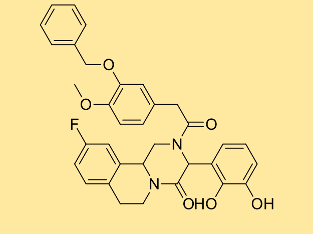	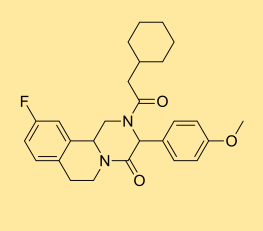	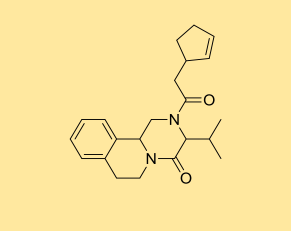
**20**	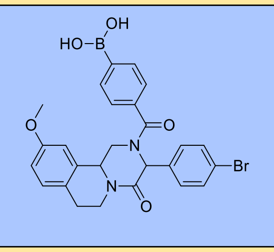	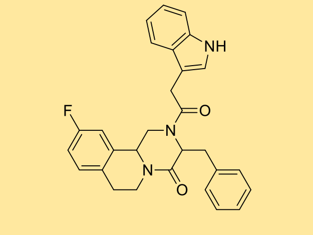	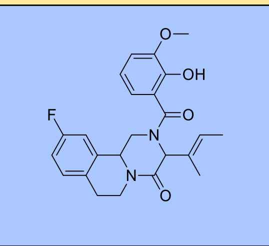	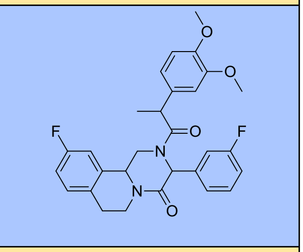
**21**	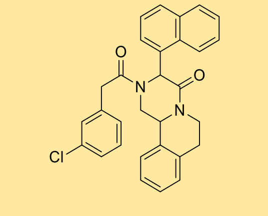	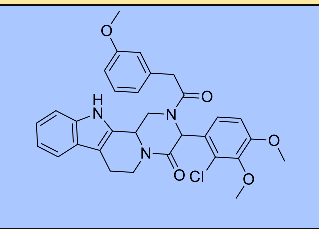	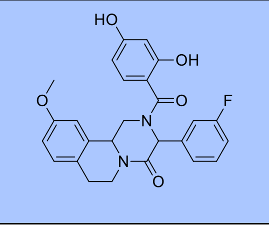	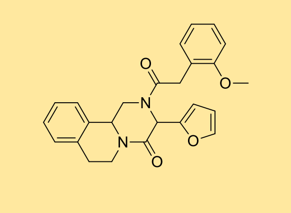

**22**	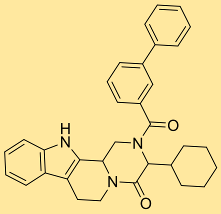	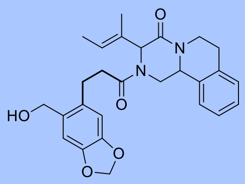	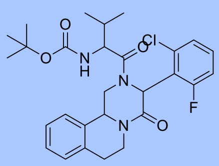	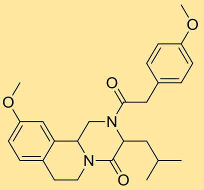
**23**	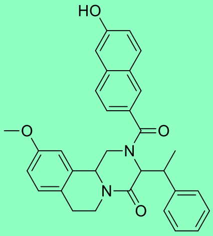	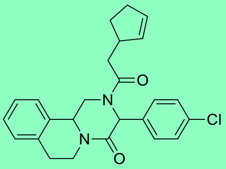	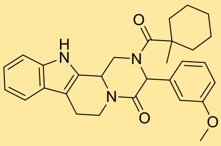	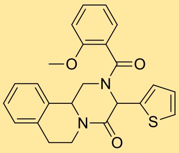
**24**	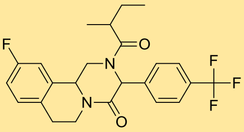	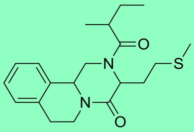	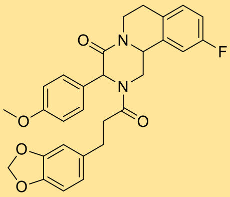	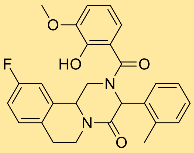

	I	J	K	L
**1**	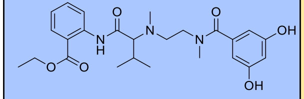	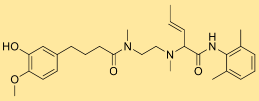	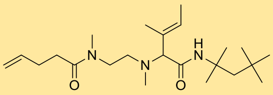	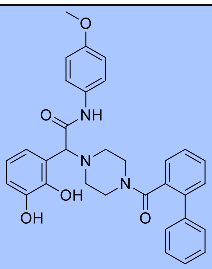
**2**	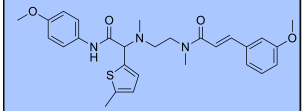	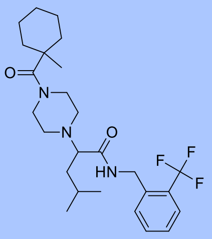	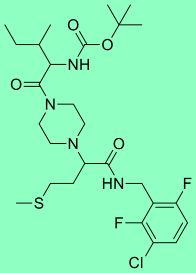	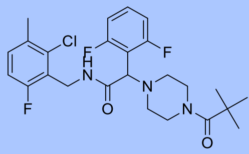
**3**	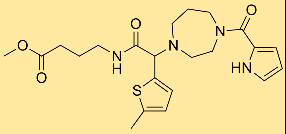	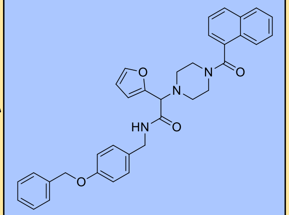	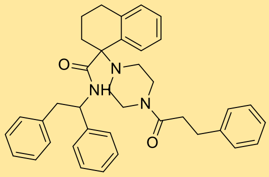	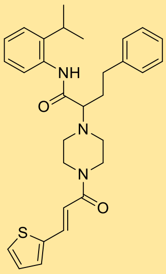

**4**	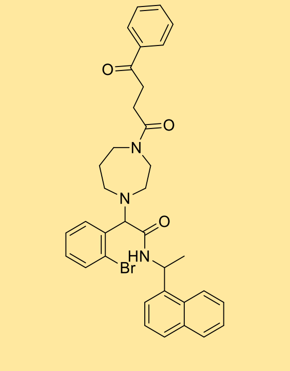	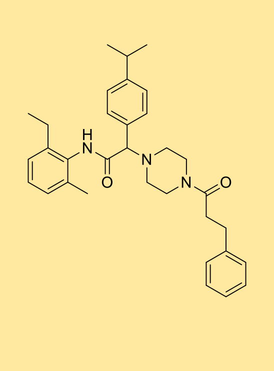	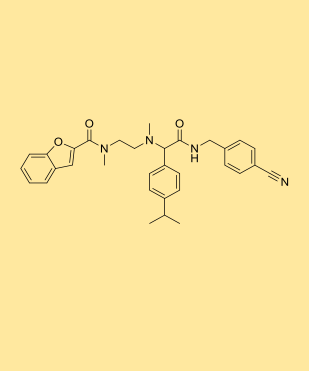	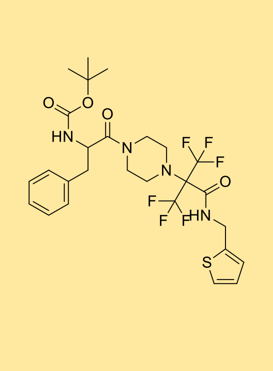
**5**	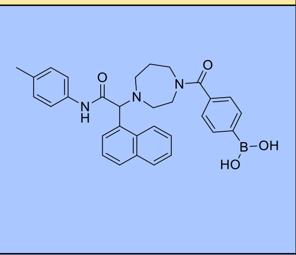	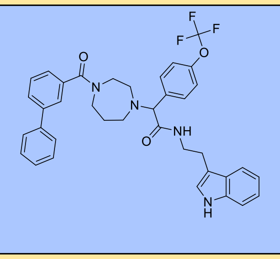	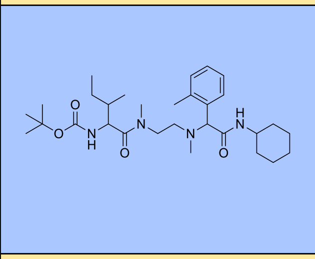	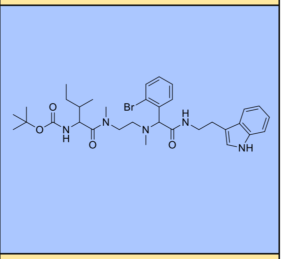
**6**	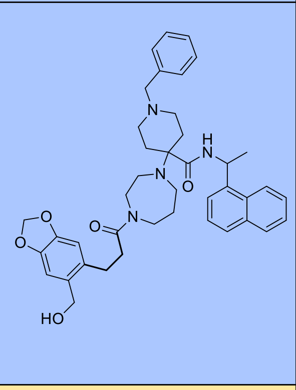	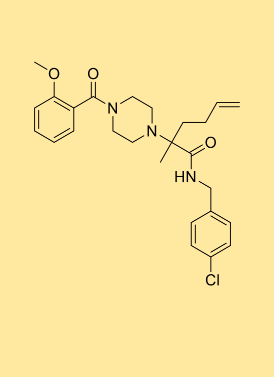	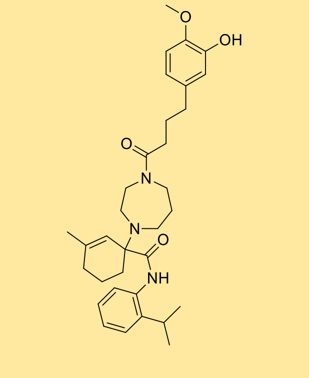	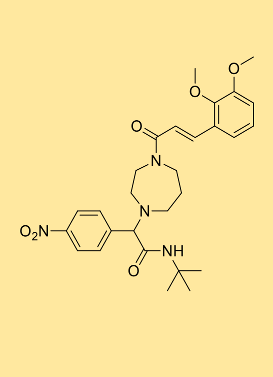
**7**	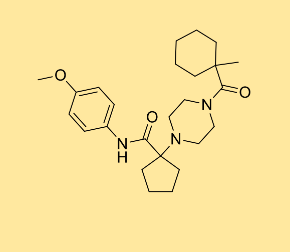	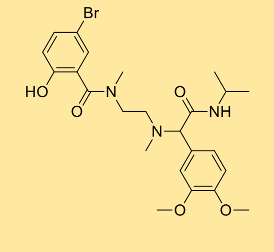	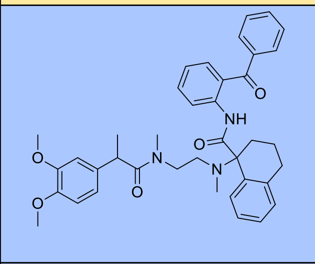	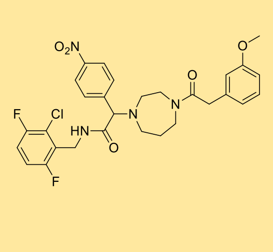
**8**	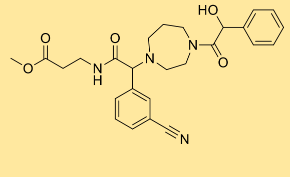	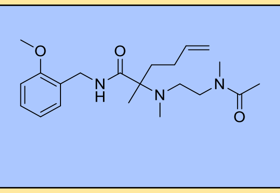	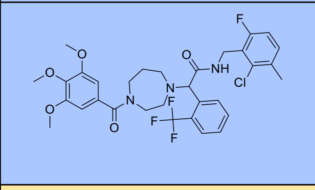	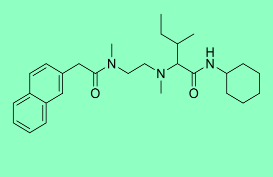
**9**	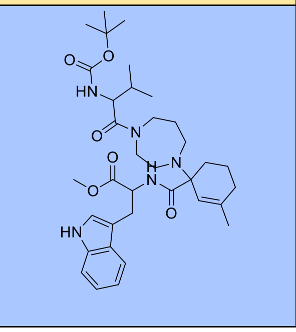	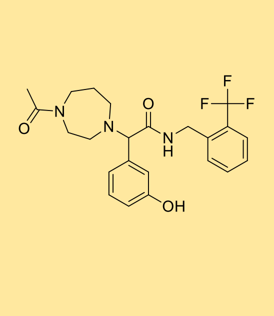	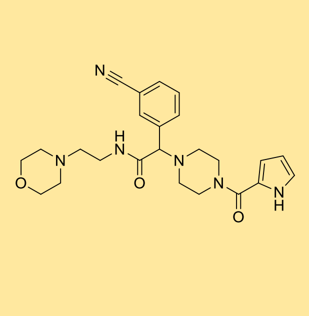	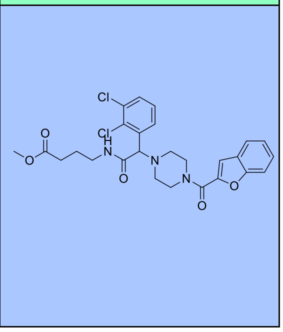

**10**	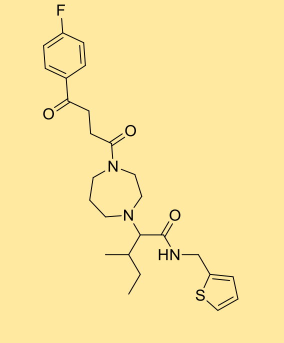	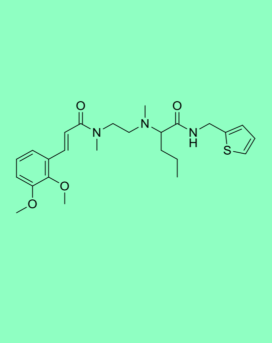	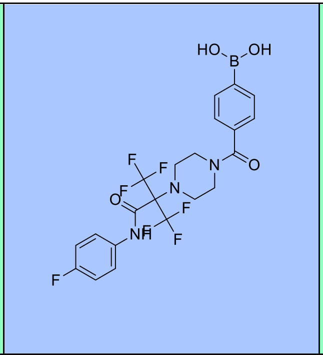	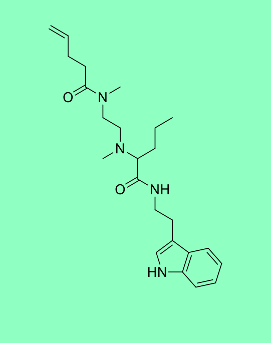
**11**	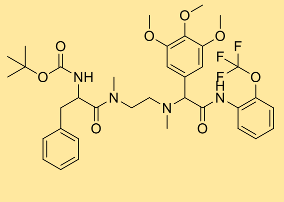	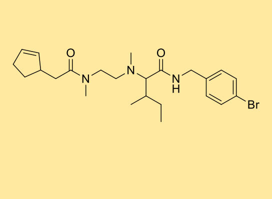	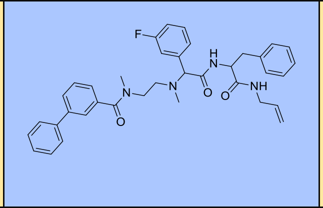	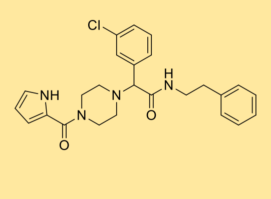
**12**	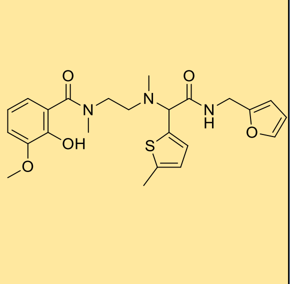	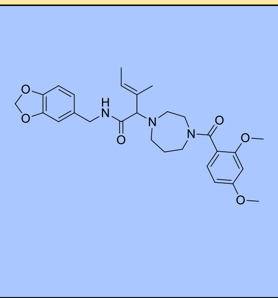	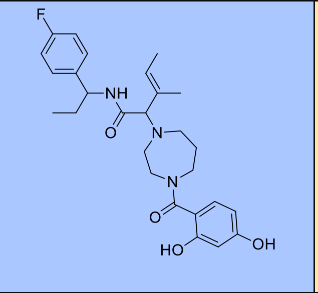	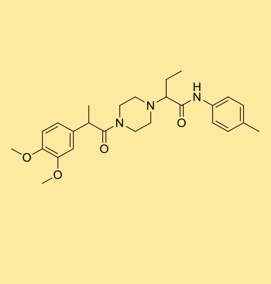
**13**	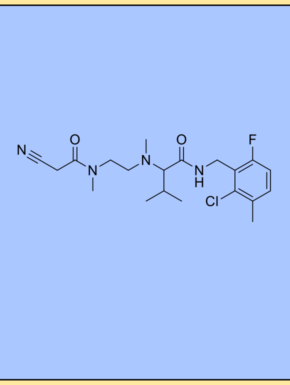	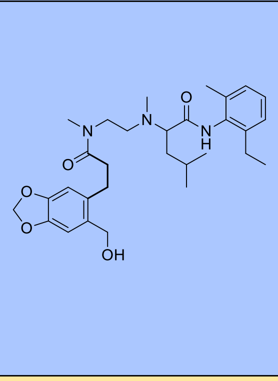	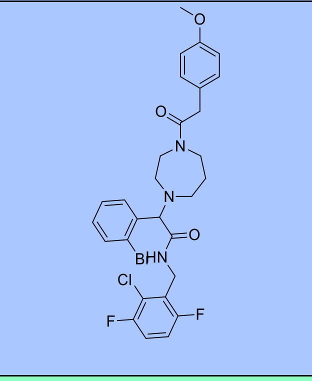	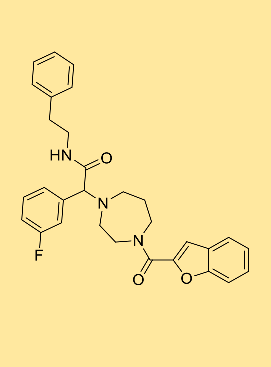
**14**	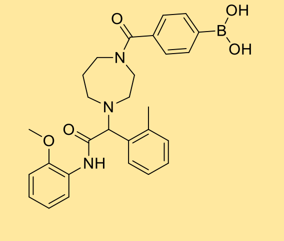	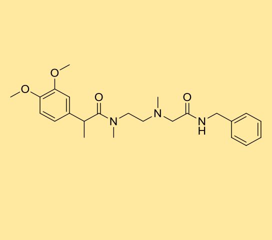	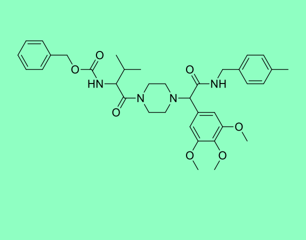	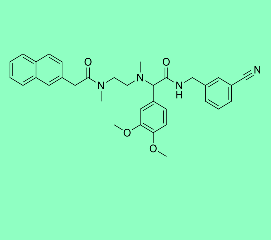
**15**	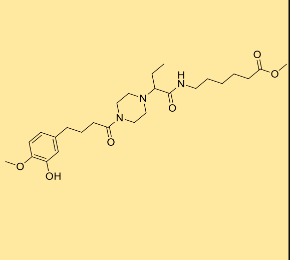	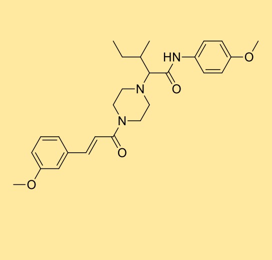	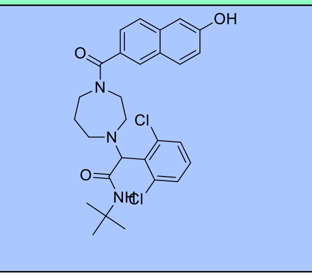	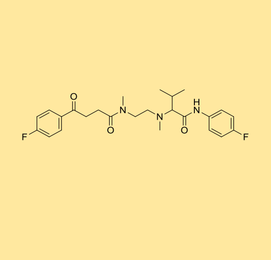

**16**	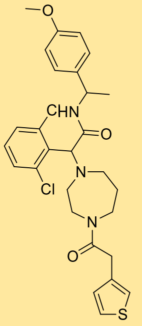	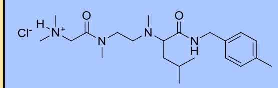	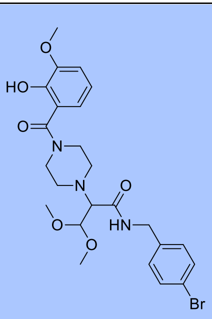	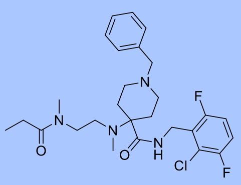
**17**	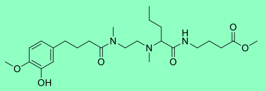	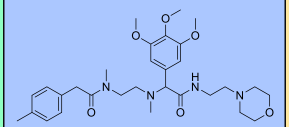	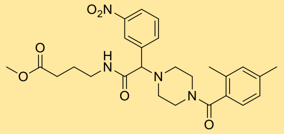	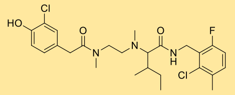
**18**	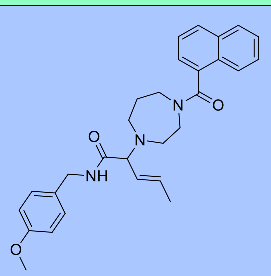	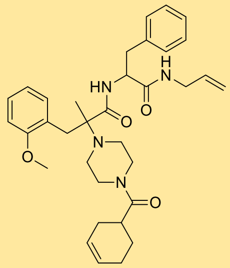	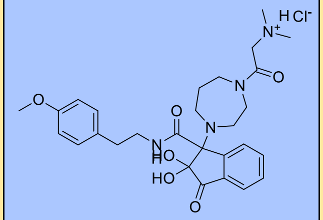	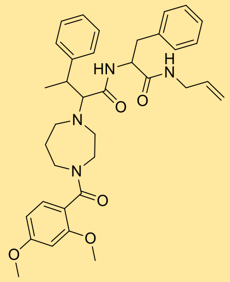
**19**	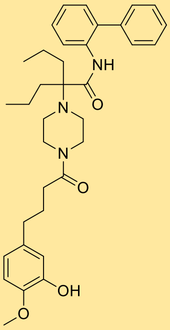	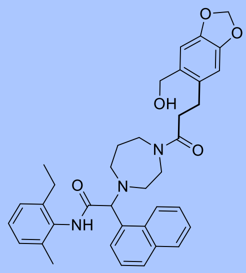	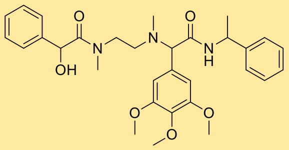	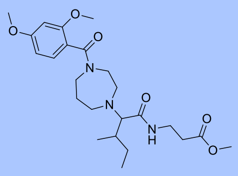
**20**	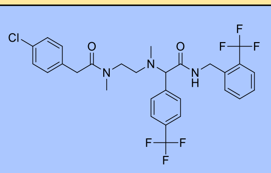	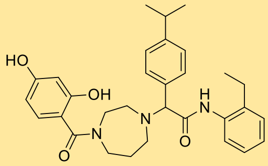	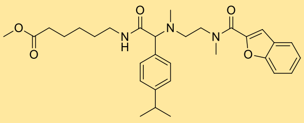	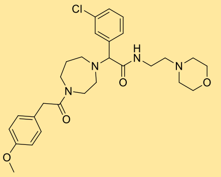
**21**	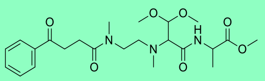	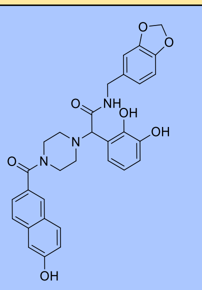	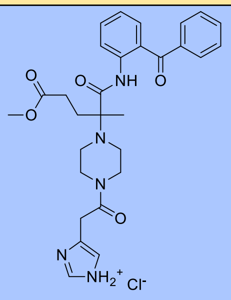	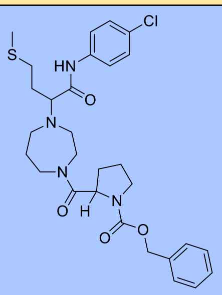

**22**	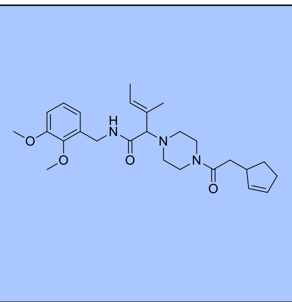	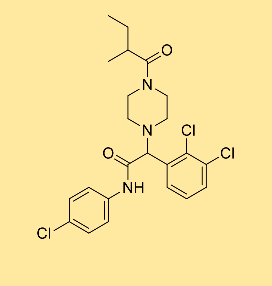	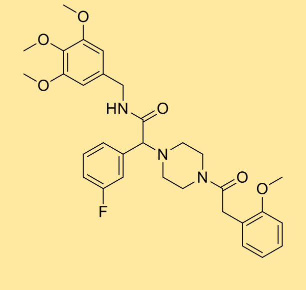	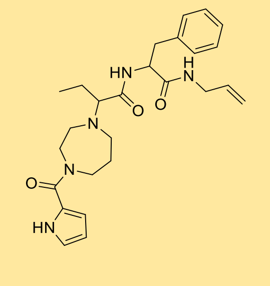
**23**	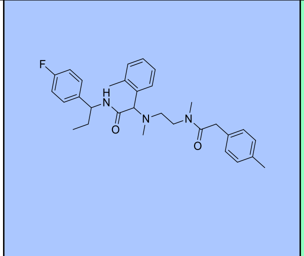	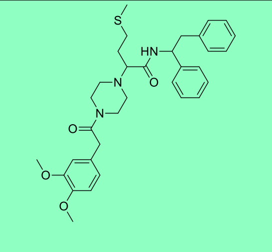	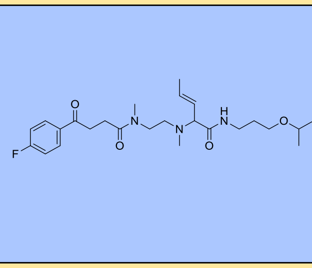	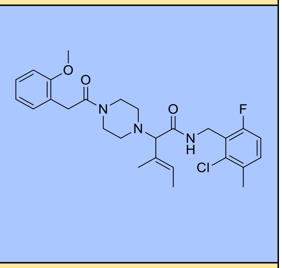
**24**	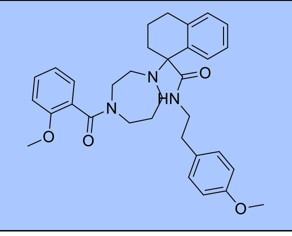	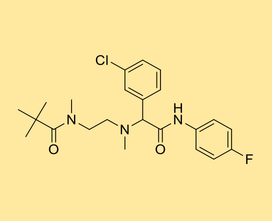	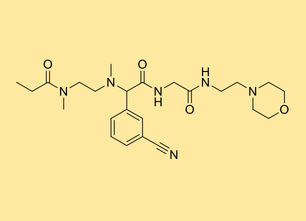	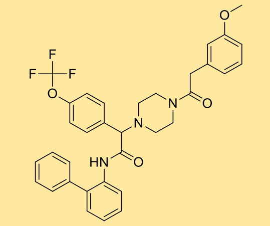

	M	N	O	P
**1**	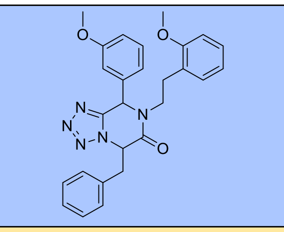	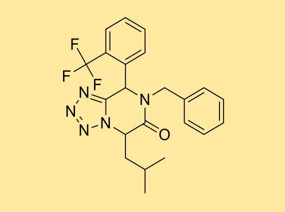	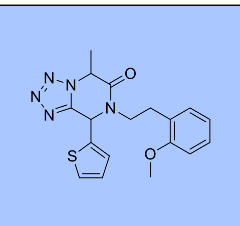	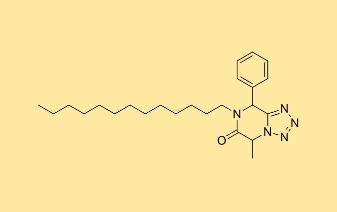
**2**	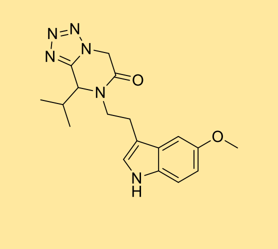	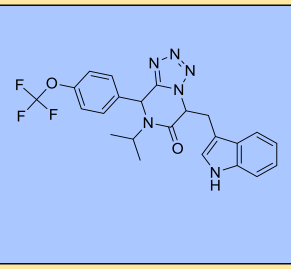	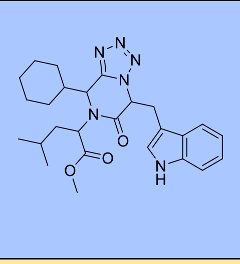	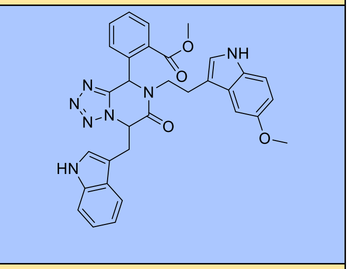
**3**	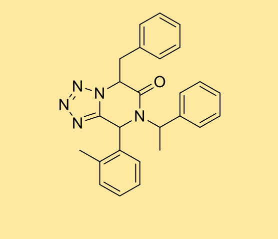	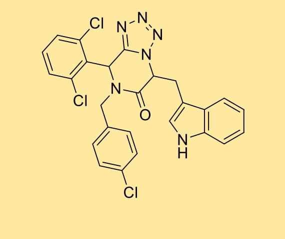	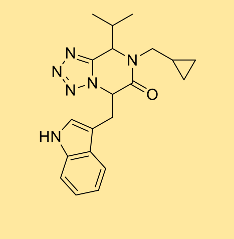	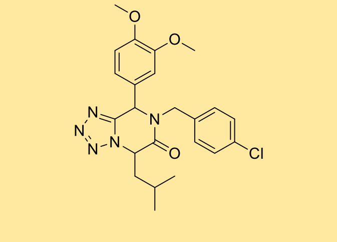
**4**	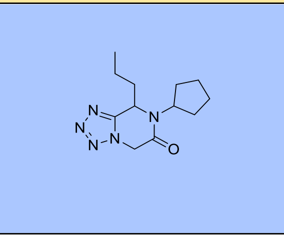	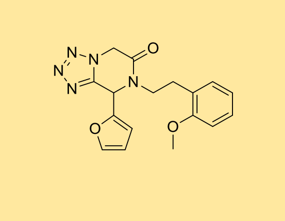	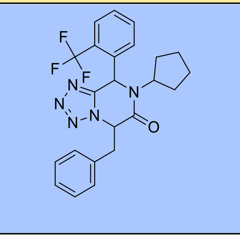	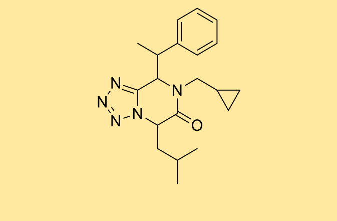

**5**	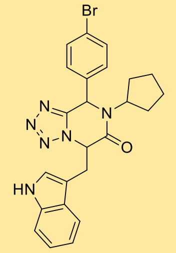	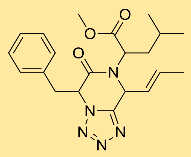	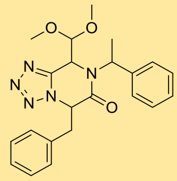	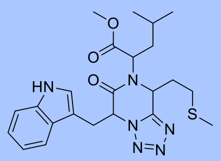
**6**	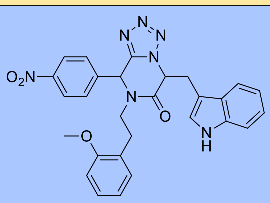	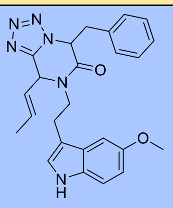	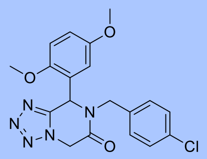	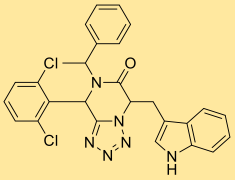
**7**	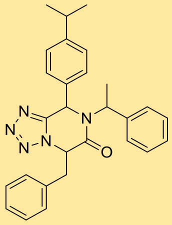	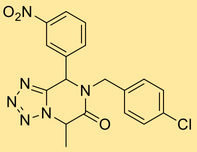	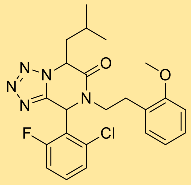	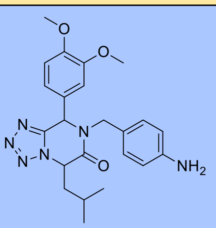
**8**	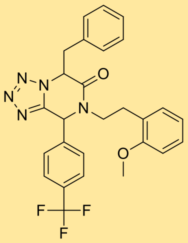	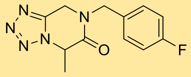	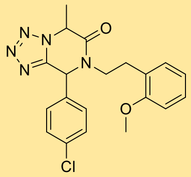	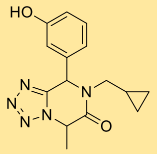
**9**	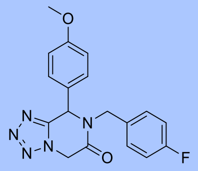	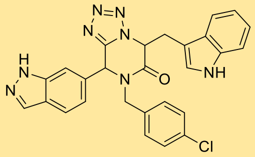	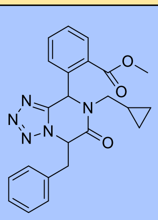	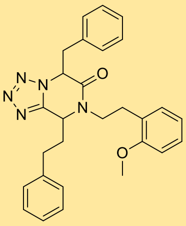
**10**	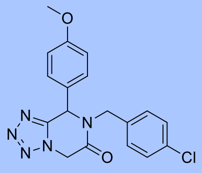	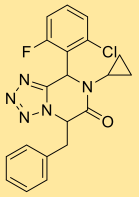	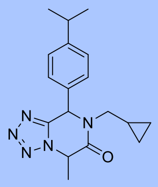	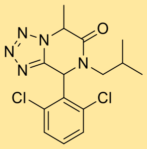
**11**	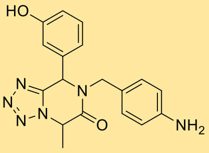	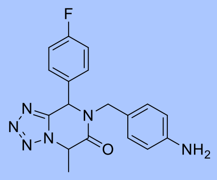	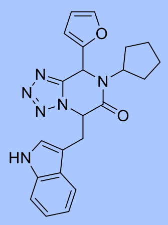	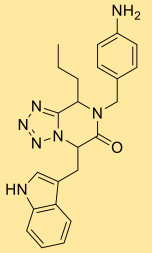

**12**	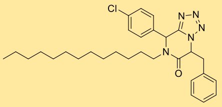	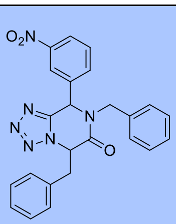	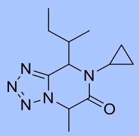	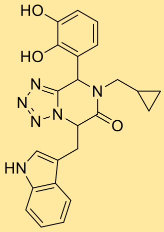
**13**	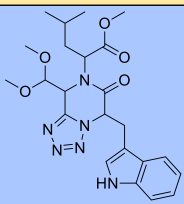	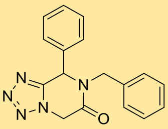	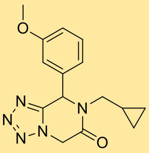	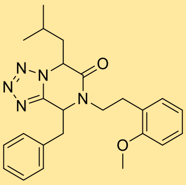
**14**	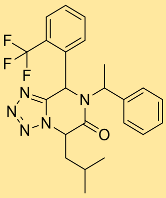	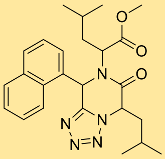	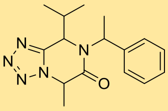	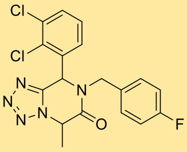
**15**	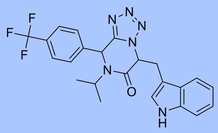	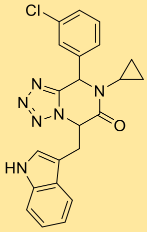	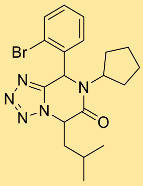	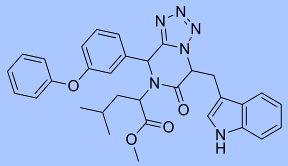
**16**	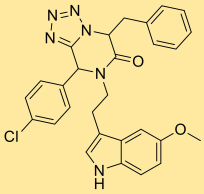	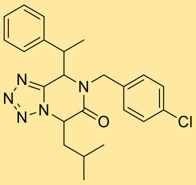	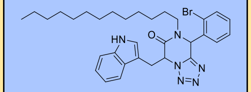	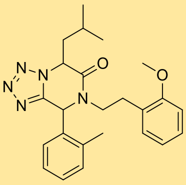
**17**	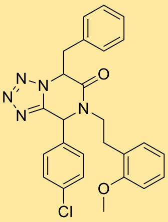	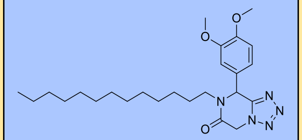	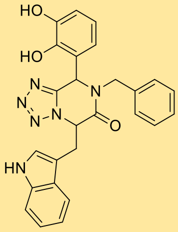	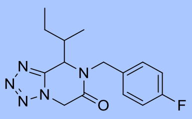
**18**	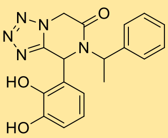	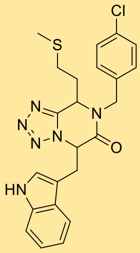	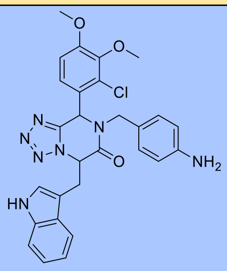	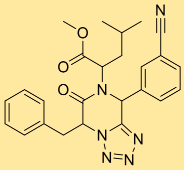

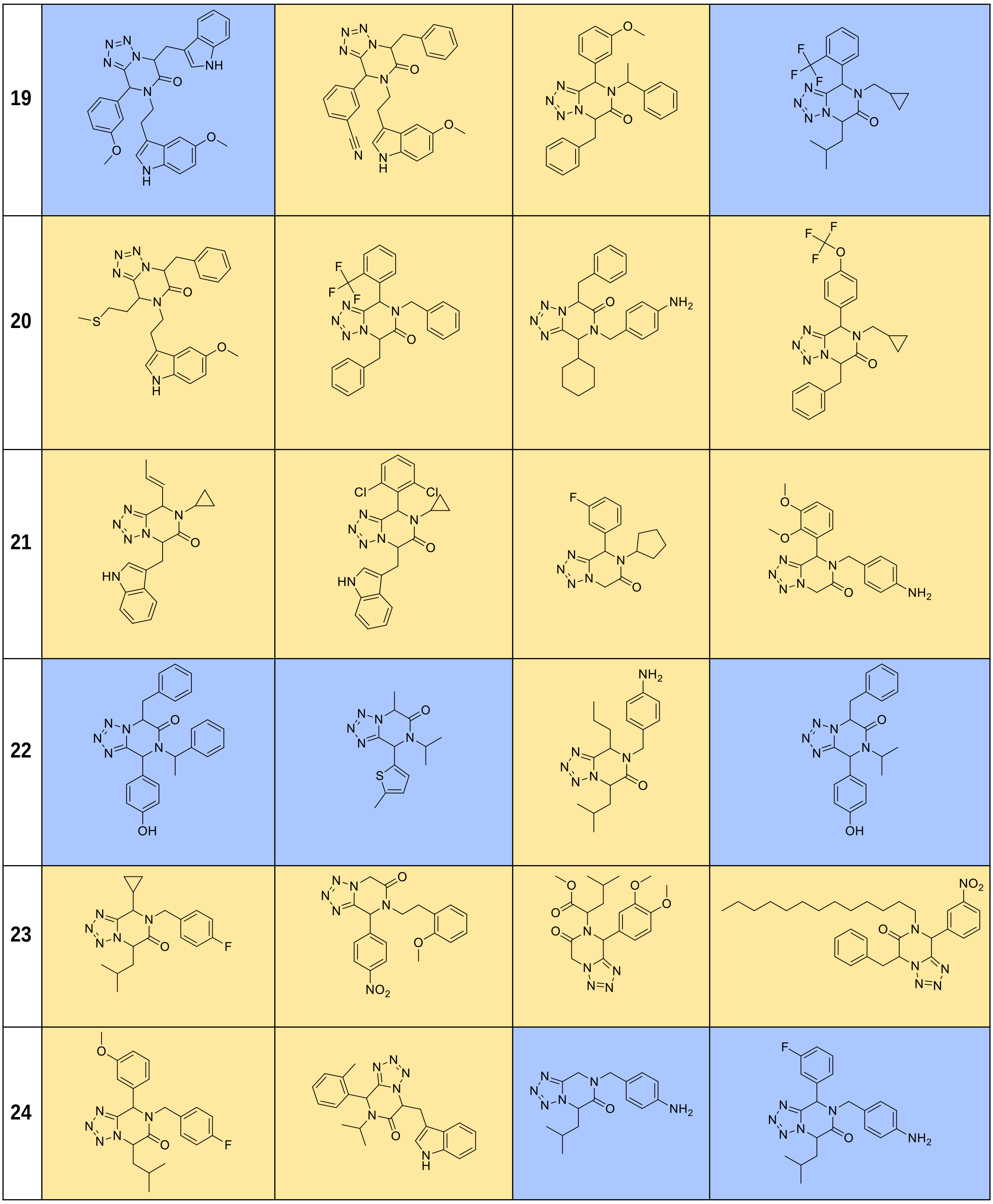

Fig. S6. Heat maps with product structures, green for major product formation, yellow for medium product formation and blue for no product formation.

2.7. Quality control (QC)

The analytics of all wells were performed by SFC-UV-MS. Mass spectra were measured on a Waters Investigator Supercritical Fluid Chromatograph with a 3100 MS Detector (ESI^+) via flow injection analysis (FIA) and MassLynx software.

Conditions: eluent composition: MeOH, 2% H_2O, 0.1% formic acid; run time: 2 min; flow rate: 1 mL/min.

Each well of the destination plate was diluted with 100 µL ethylene glycol and then the chromatographic analysis was done by SFC-MS using an autosampler.

The SFC analytic of one well took ~2 min, resulting in an overall measuring time for the 1536 wells of around 51.2 h.

2.7.1.Examples of SFC-MS analytics directly out of the destination plates I, II, III and IV

plate I, B22 (green)

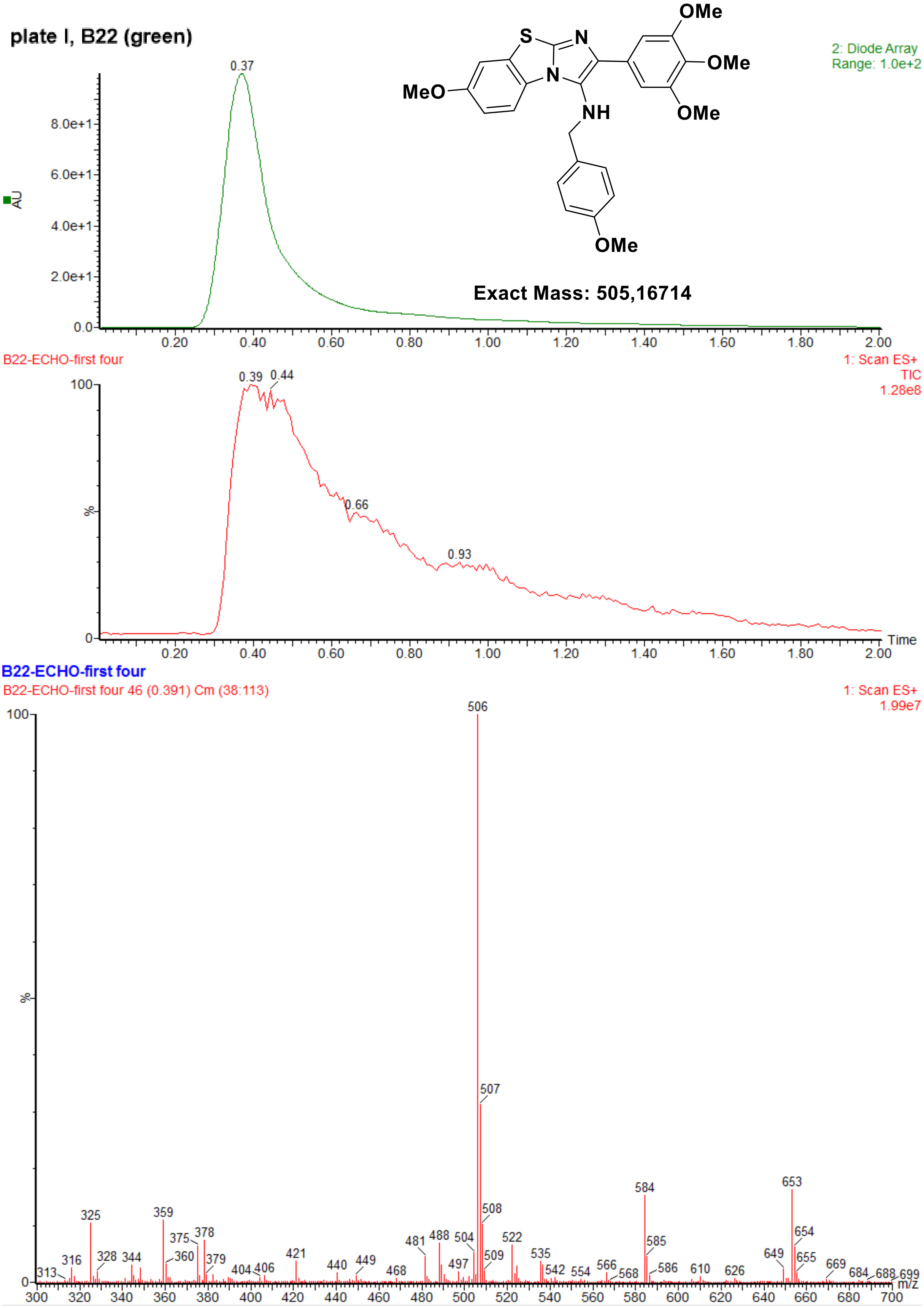

plate I, D3 (green)

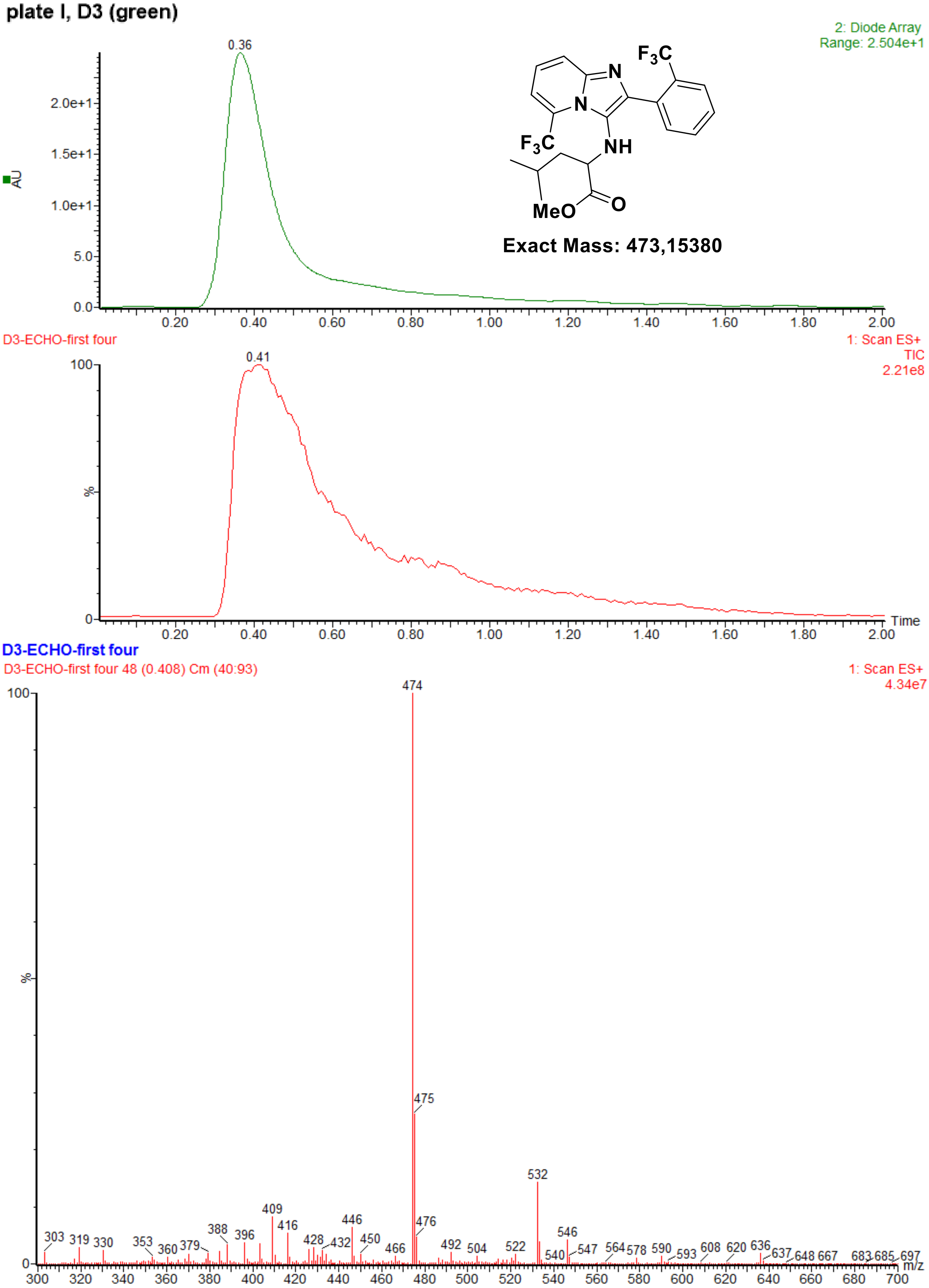

plate I, E1 (green)

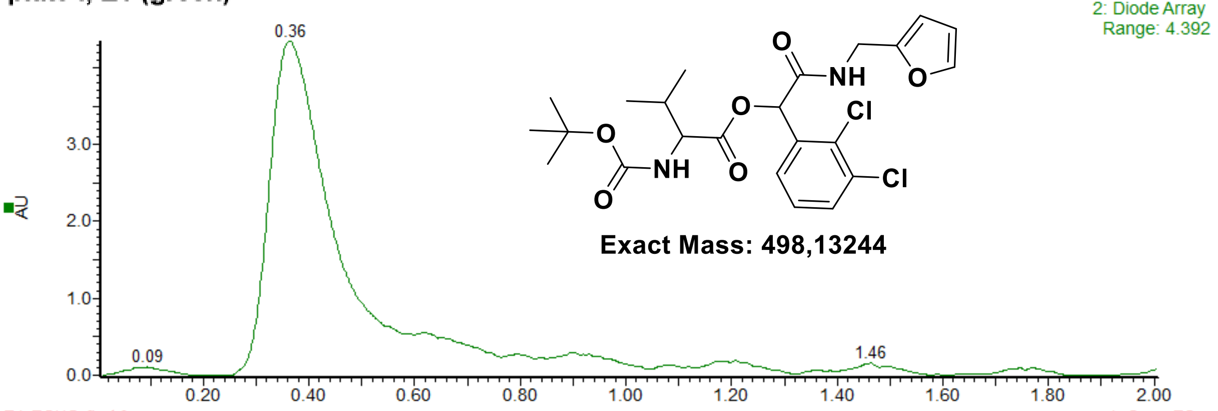

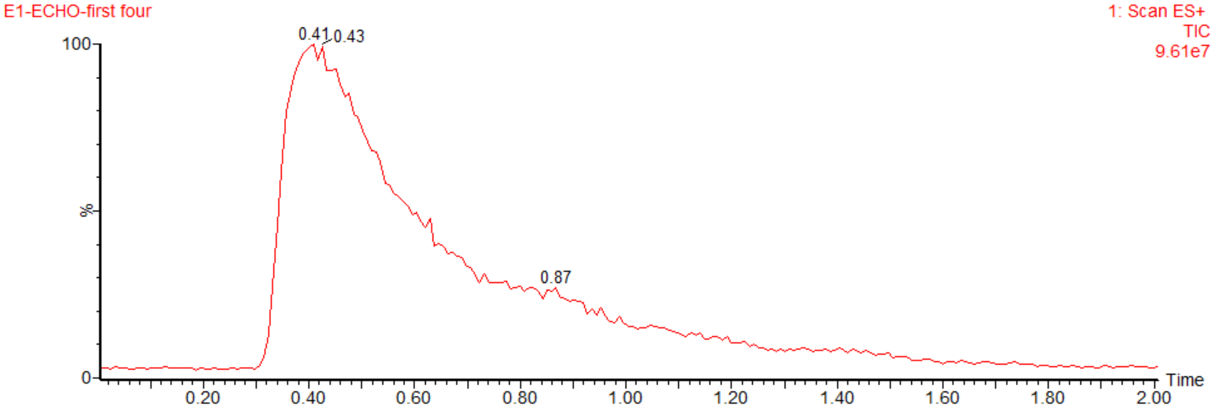

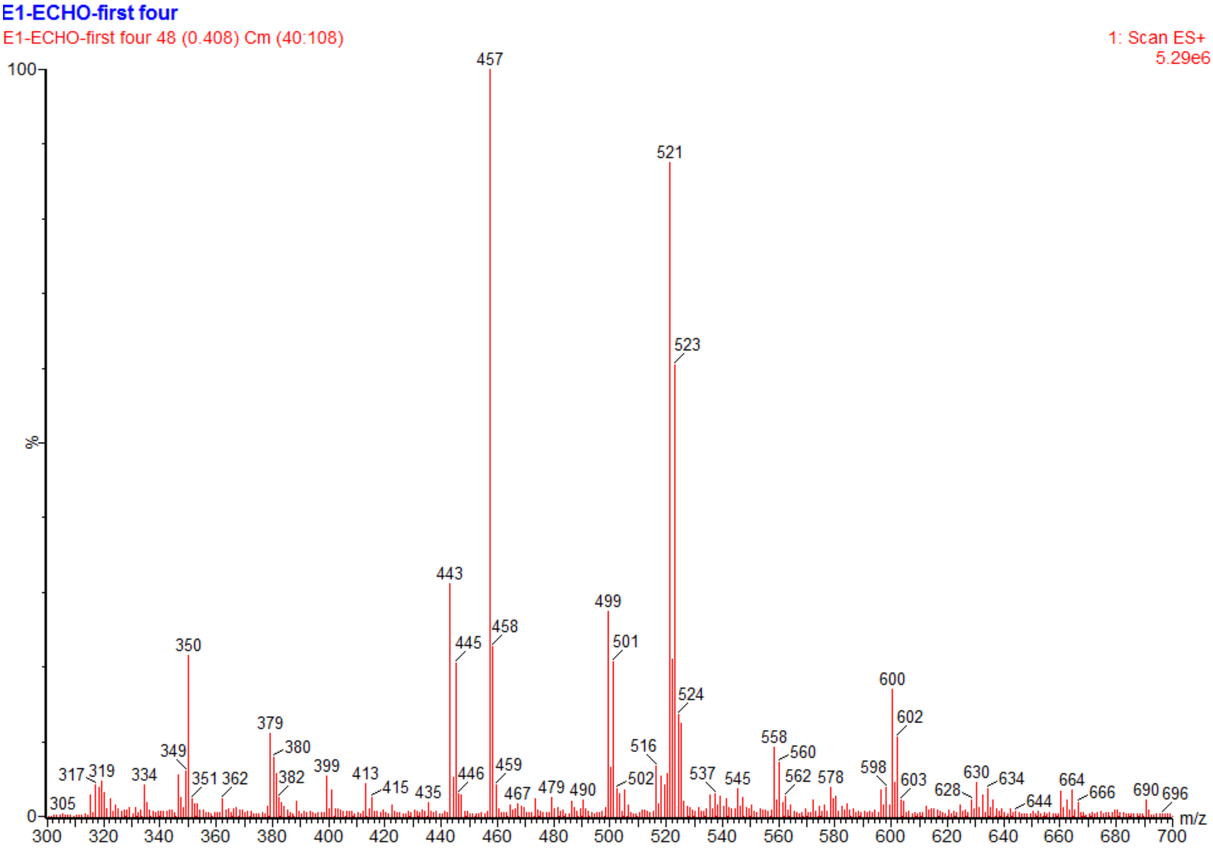

plate I, G12 (green)

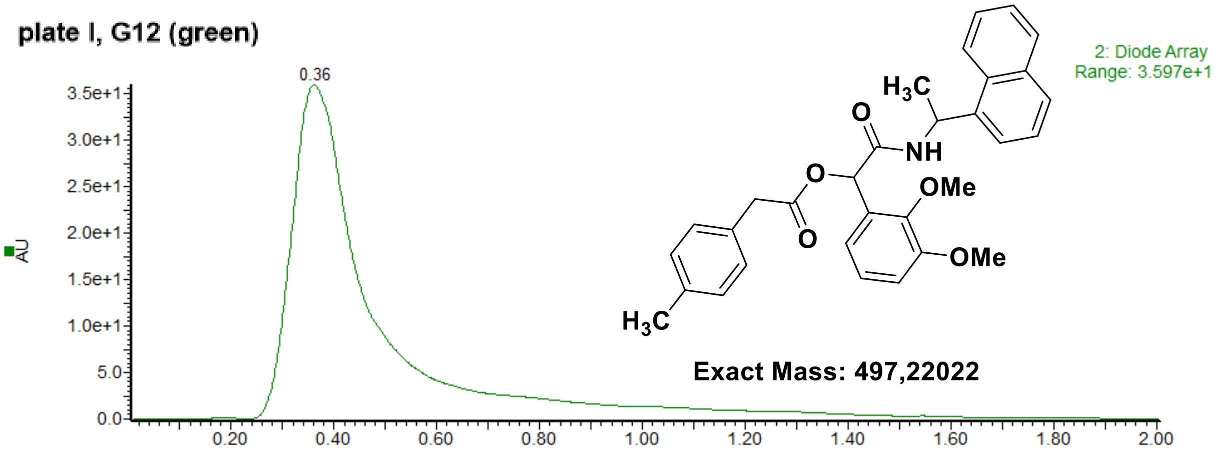

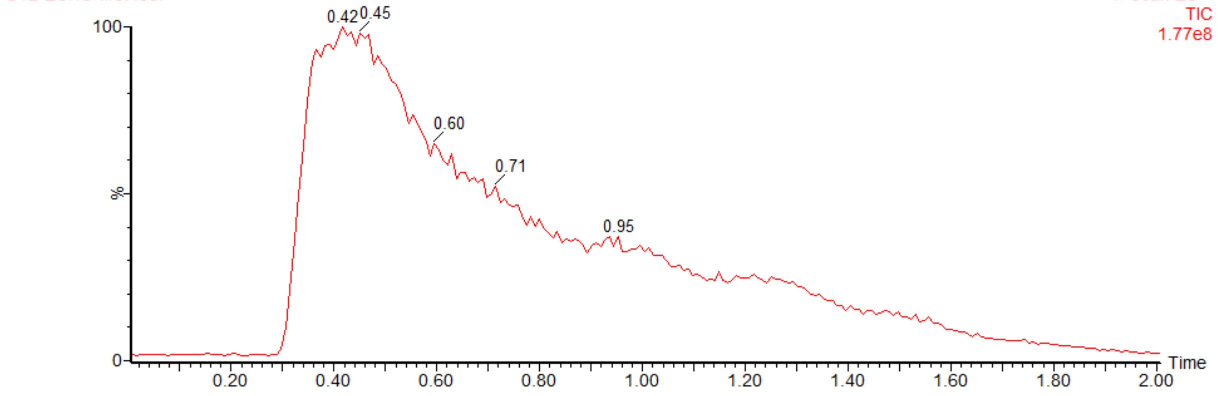

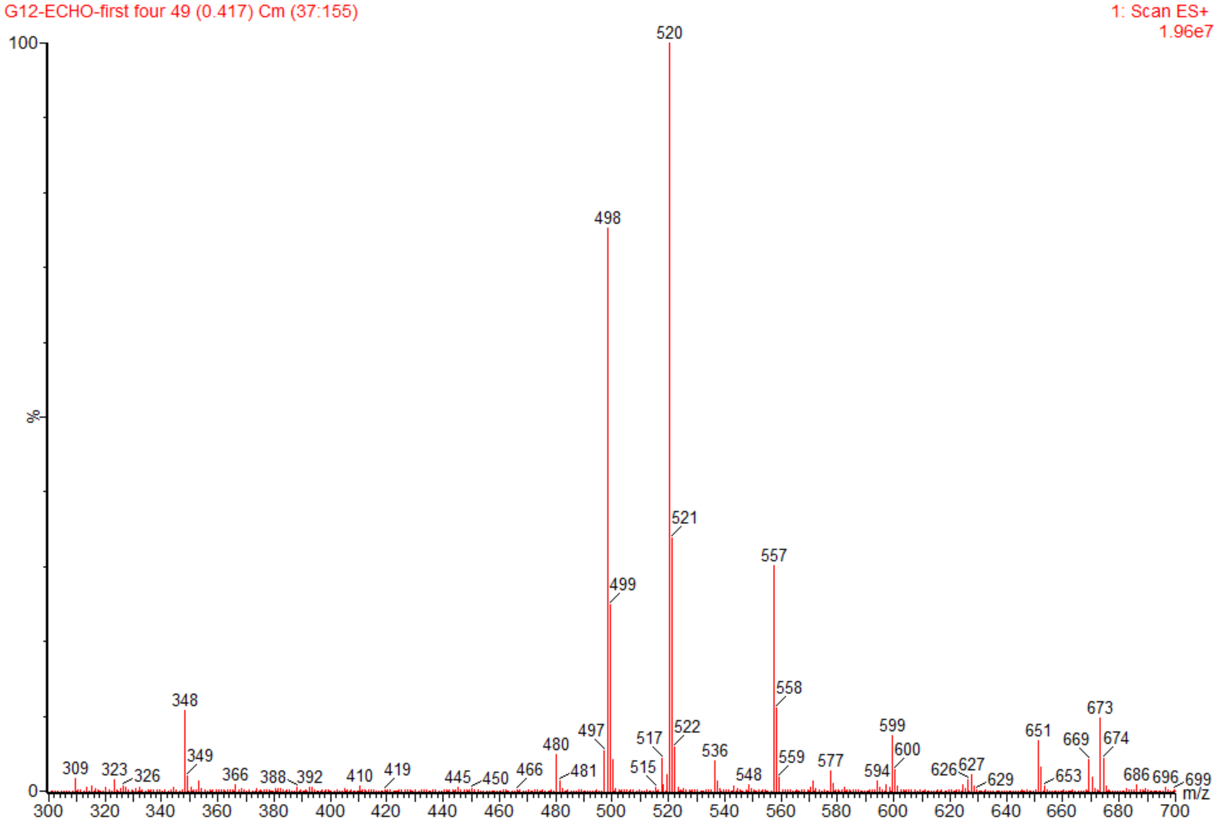

plate I, H17 (green)

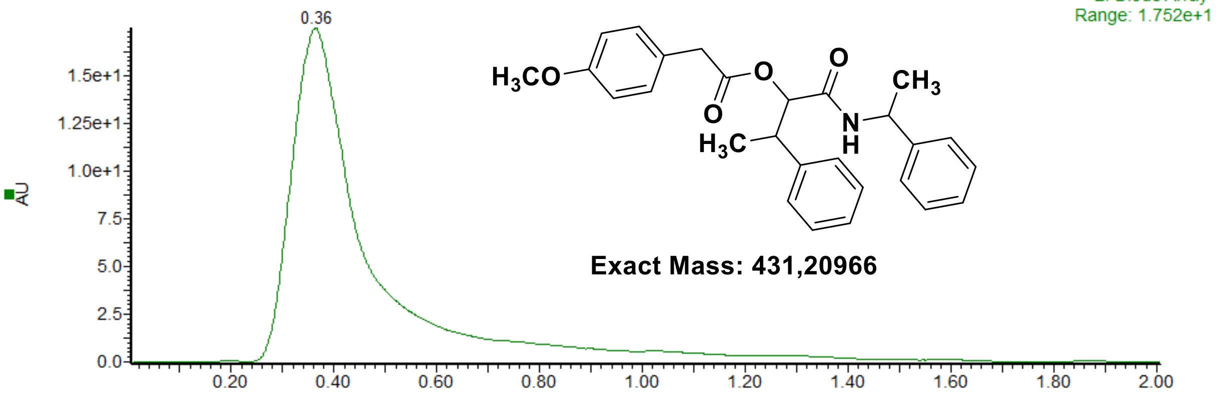

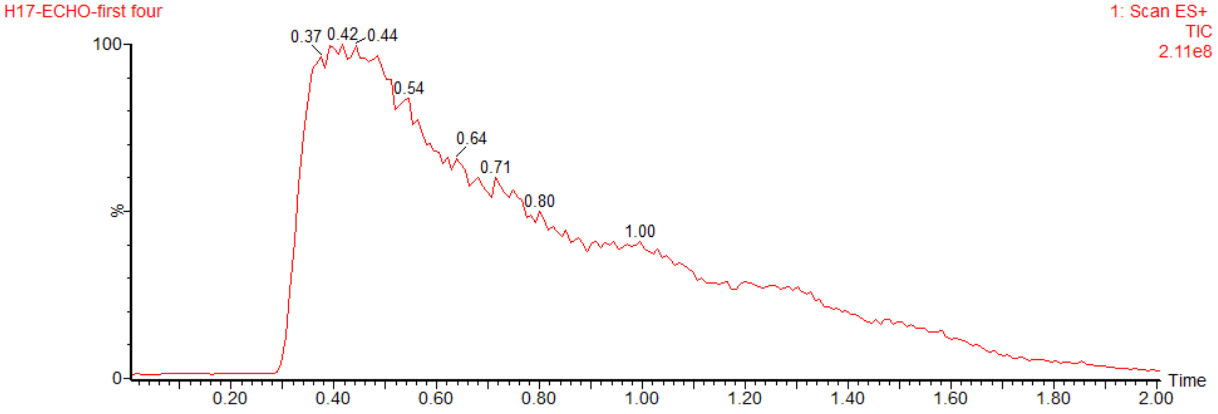

H17-ECHO-first four

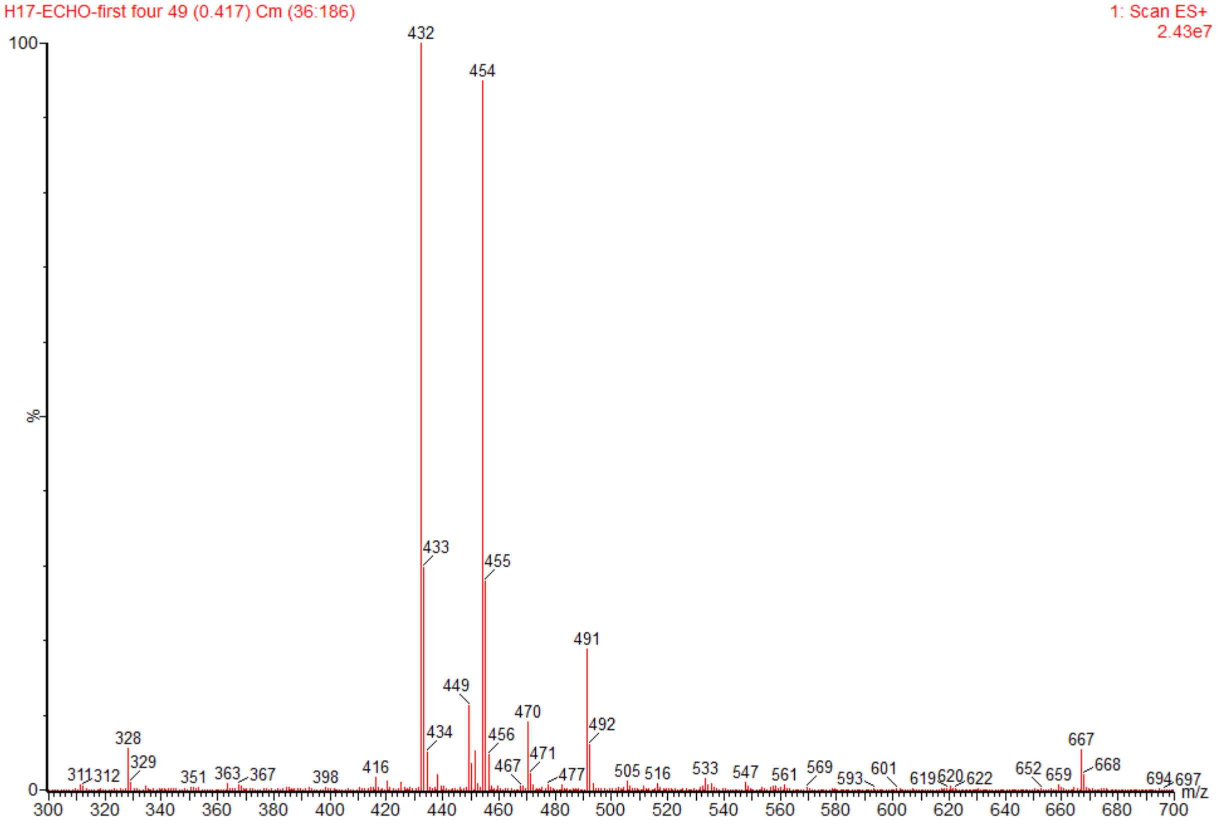

plate I, J5 (green)

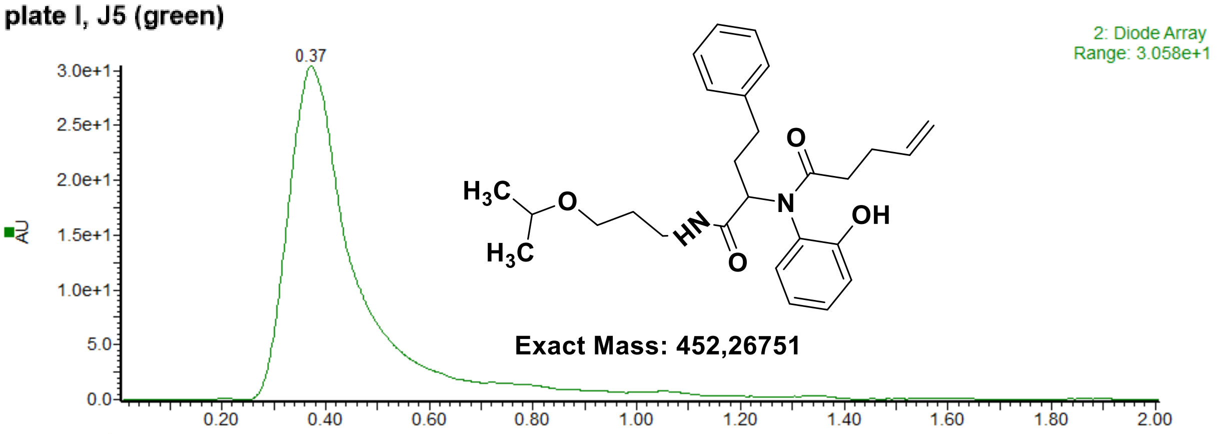

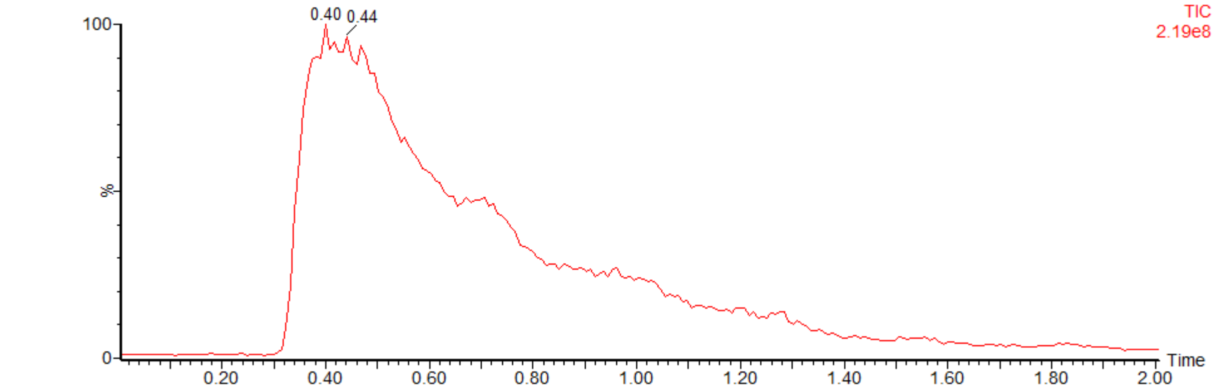

J5-ECHO-first four

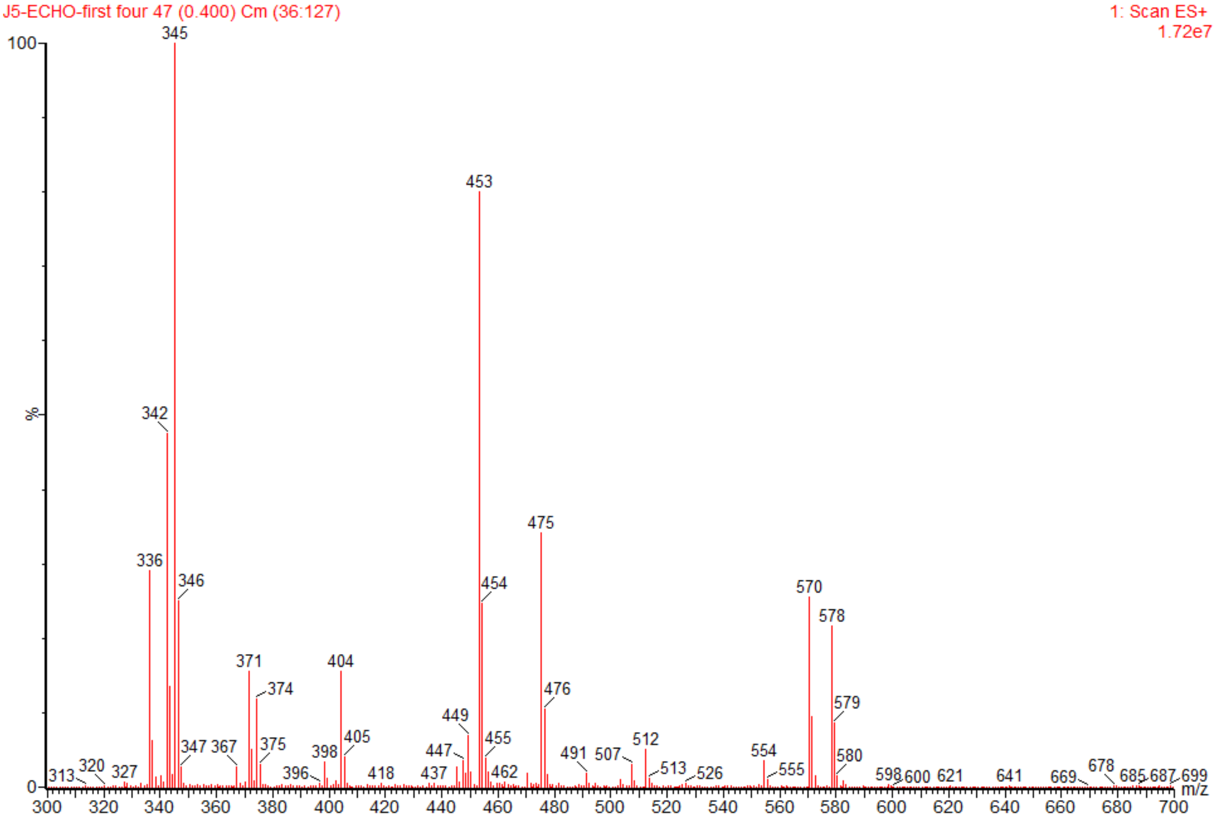

plate I, K10 (green)

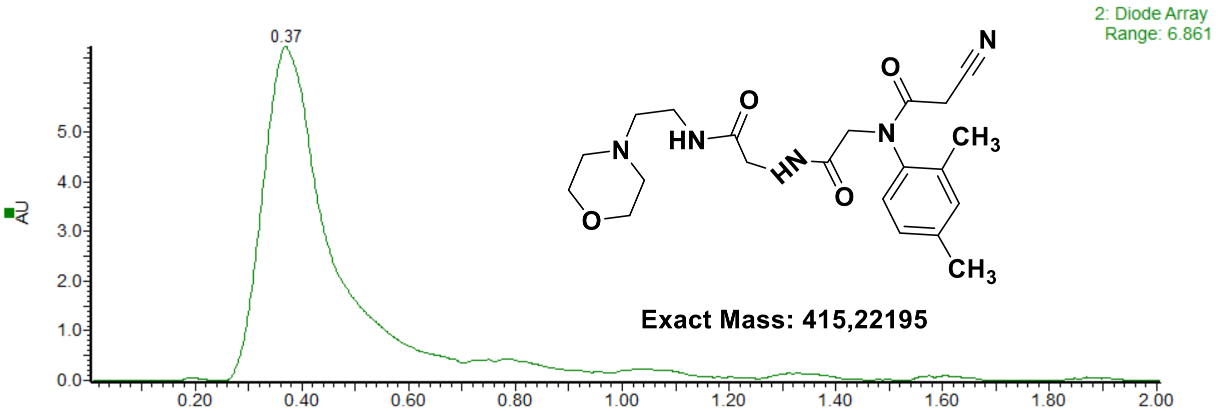

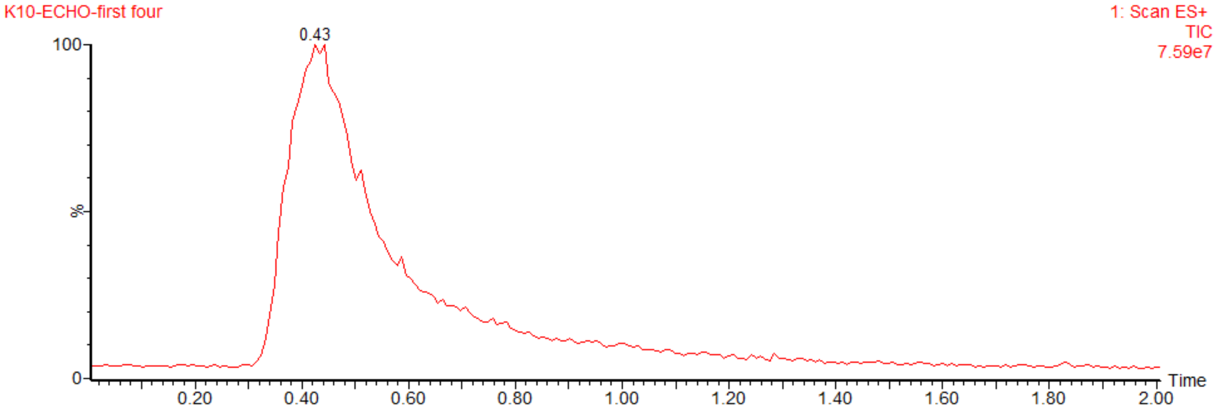

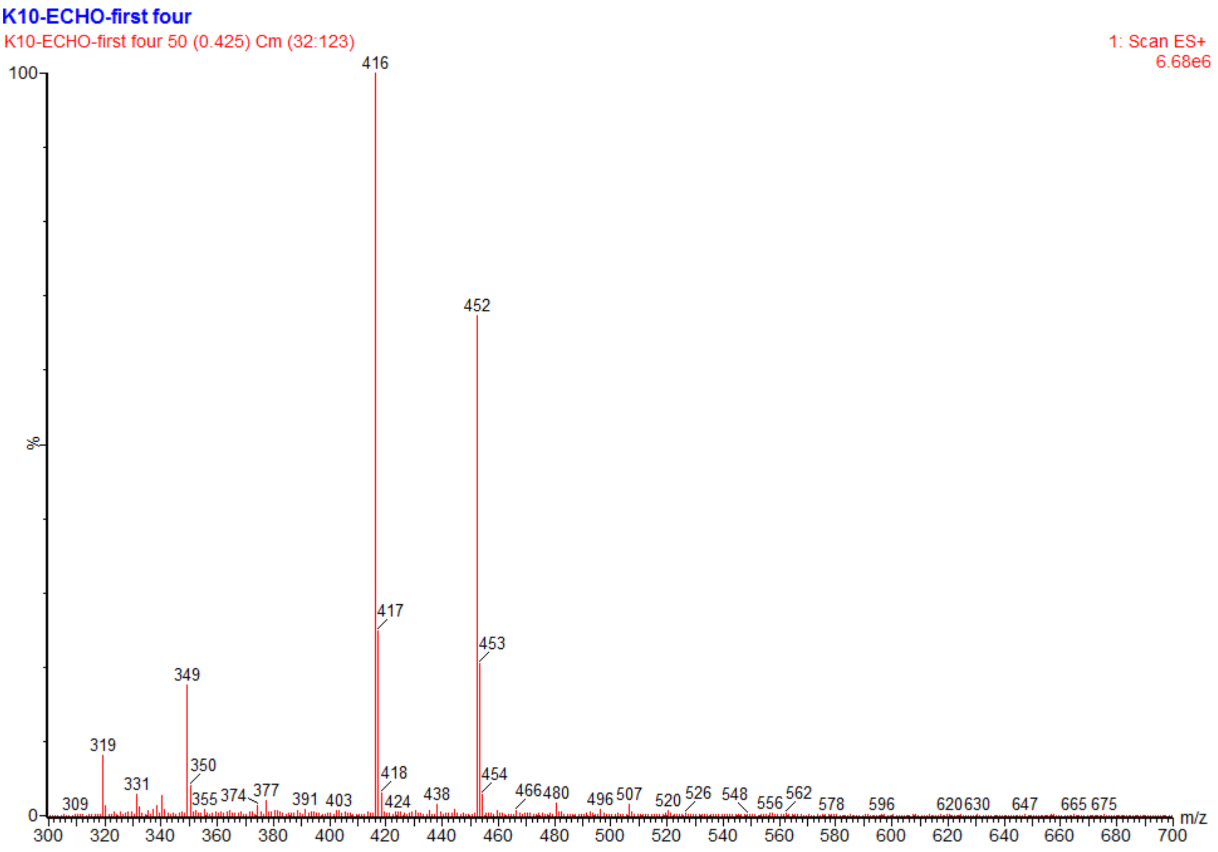

plate I, O5 (green)

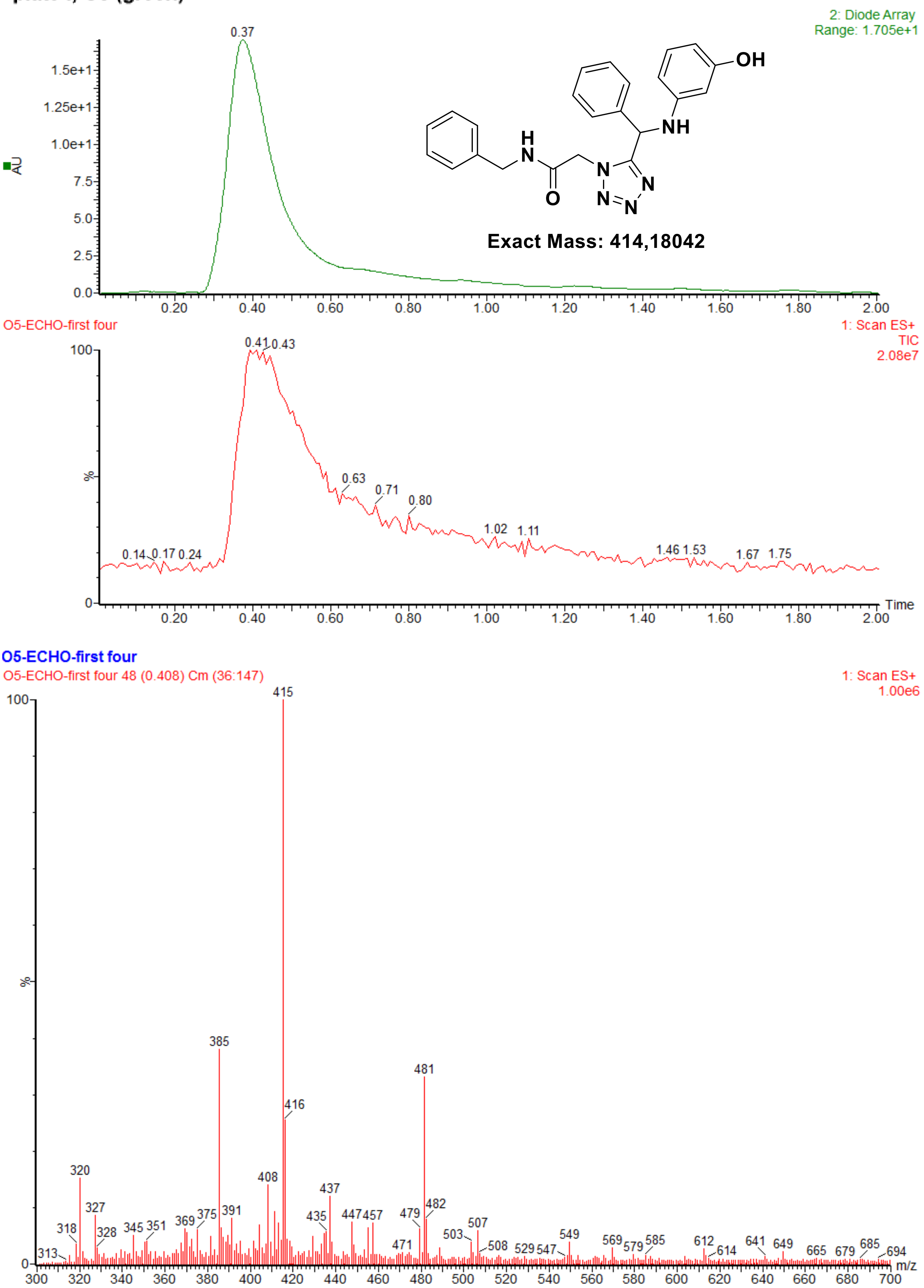

plate I, P22 (green)

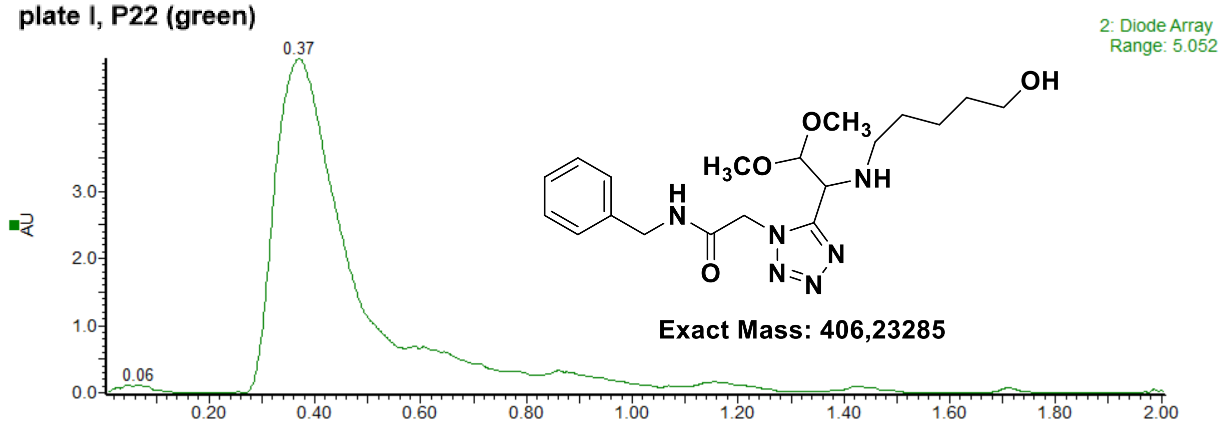

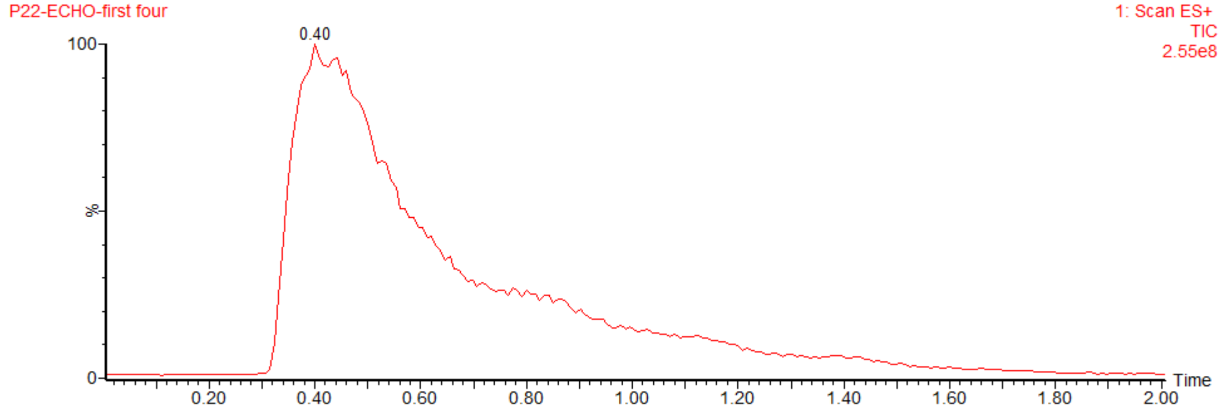

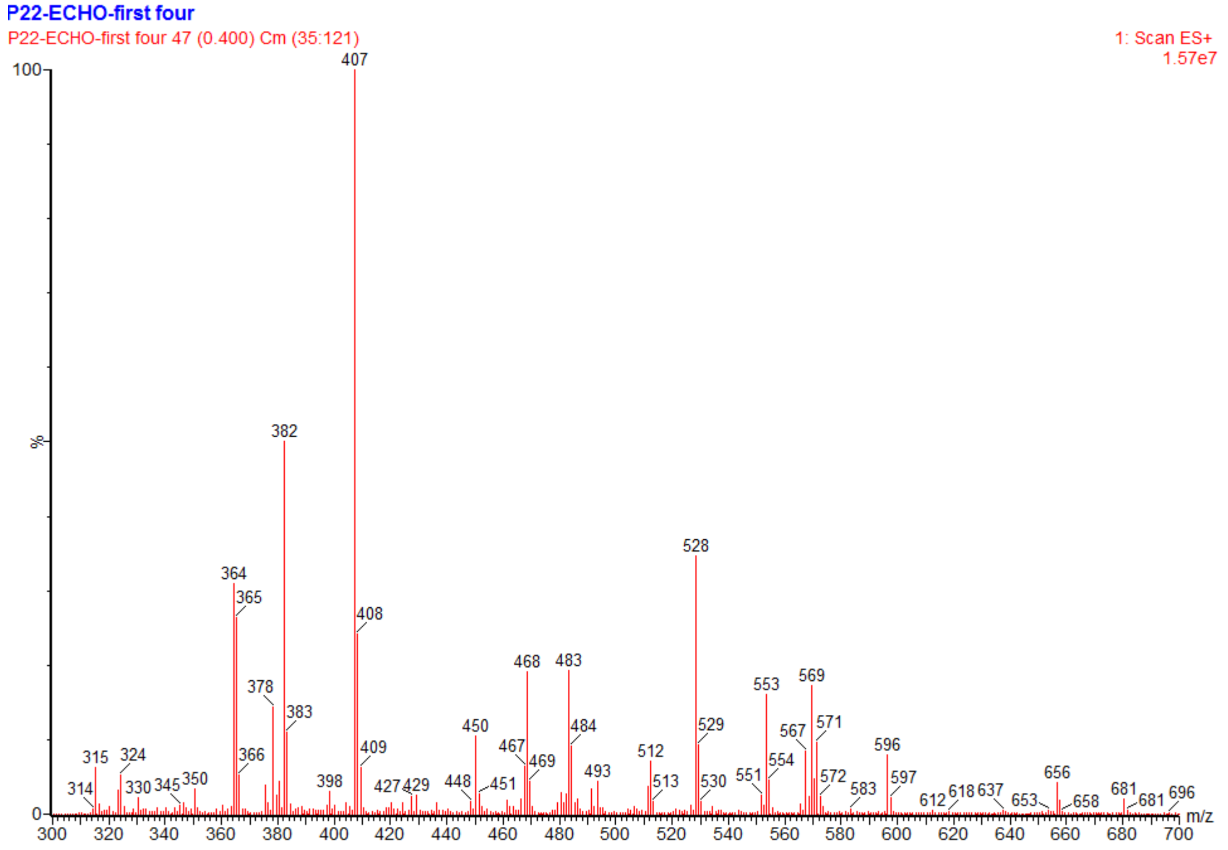

Plate II, F18 (green)

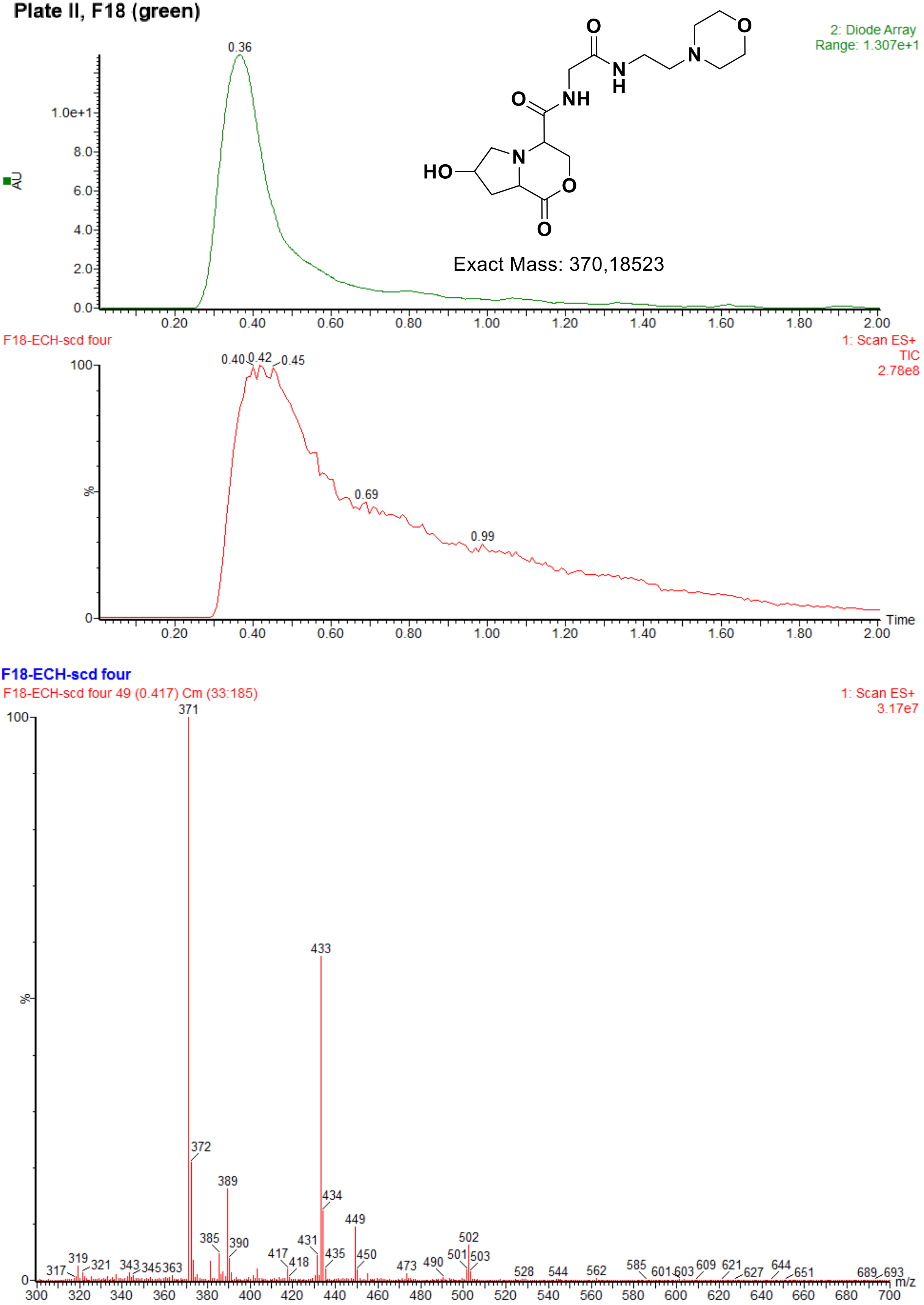

Plate II, I1 (green)

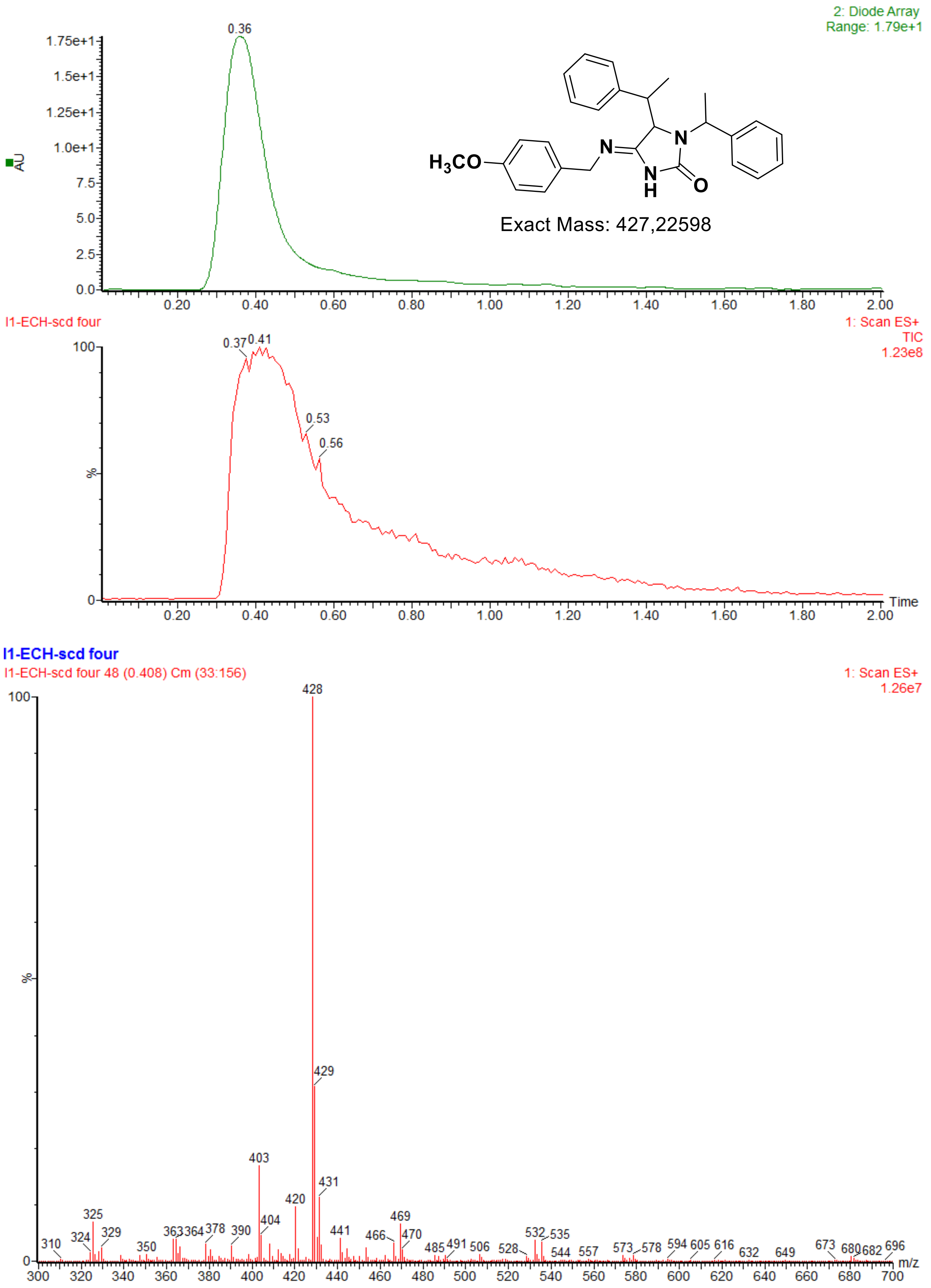

Plate II, K18 (green)

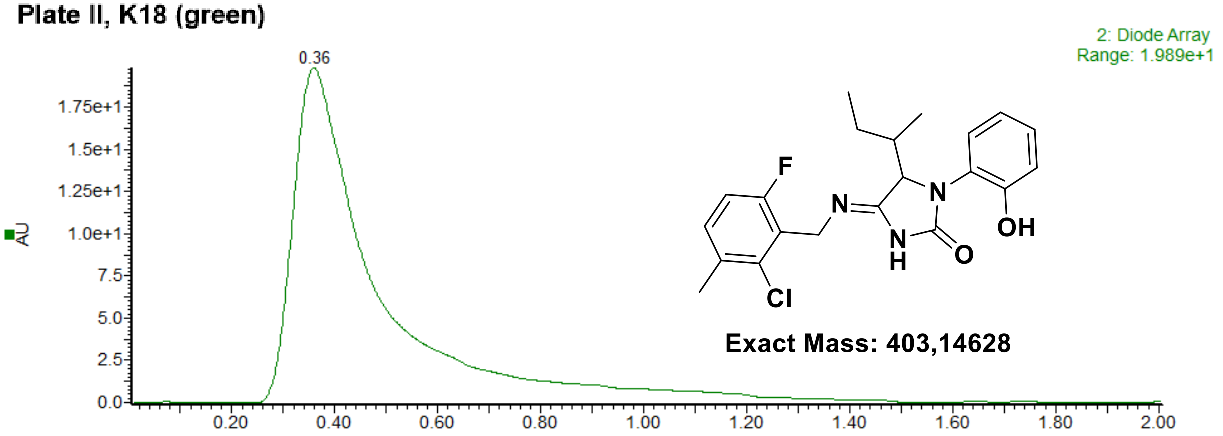

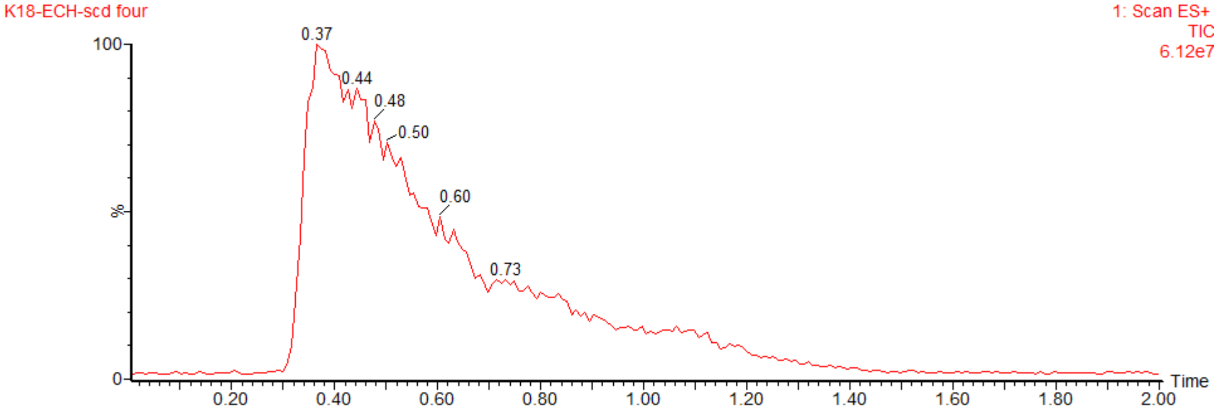

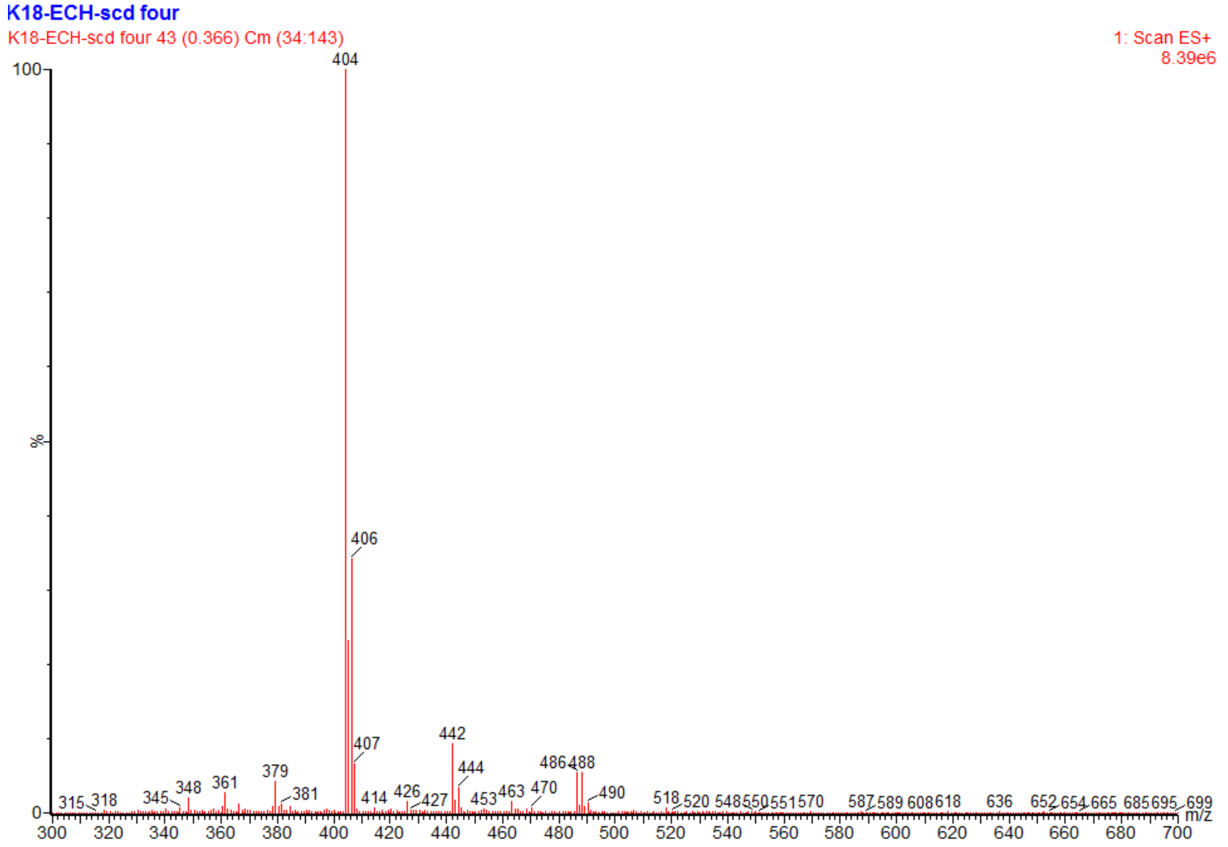

Plate II, M10 (green)

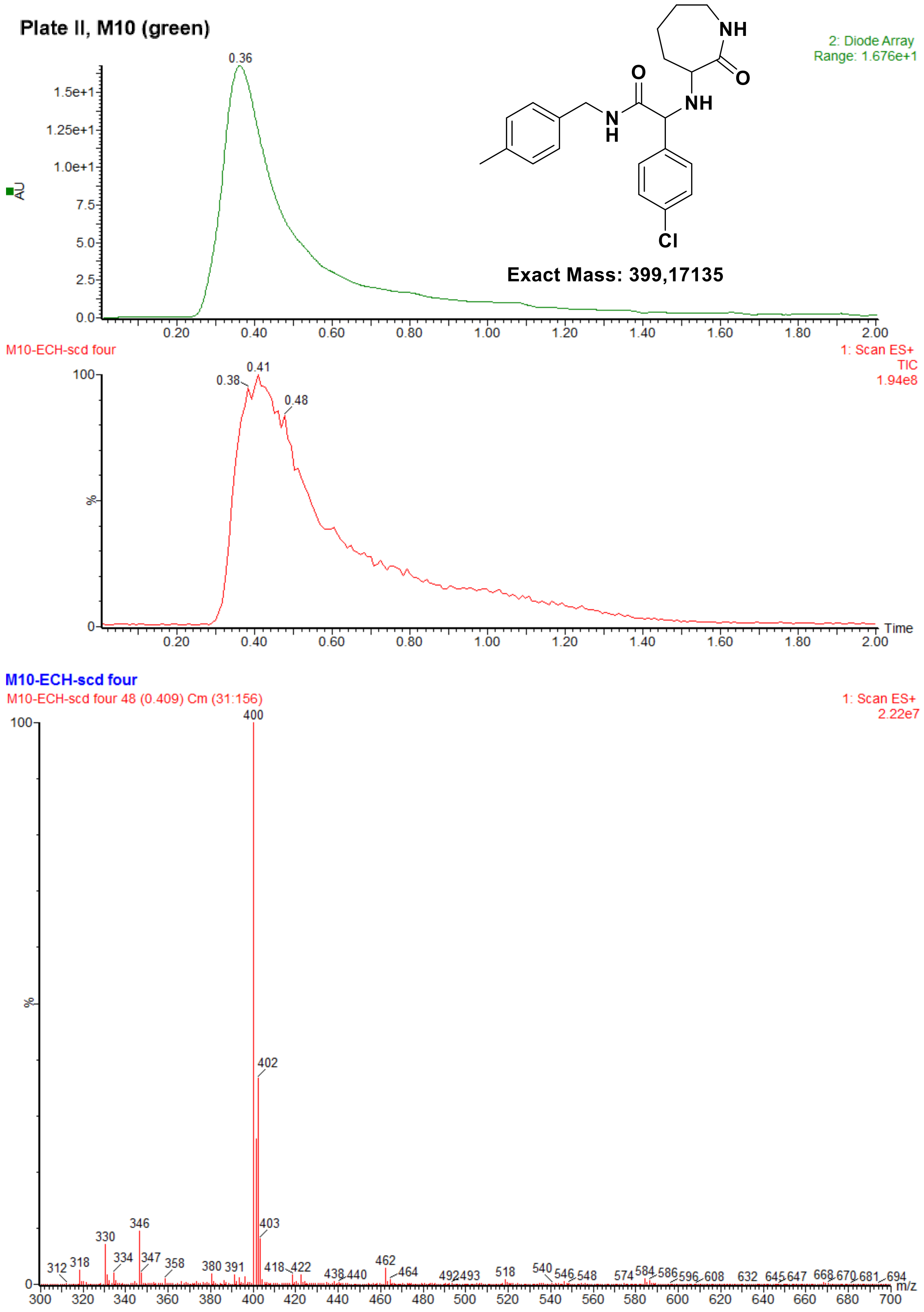

Plate II, P11 (green)

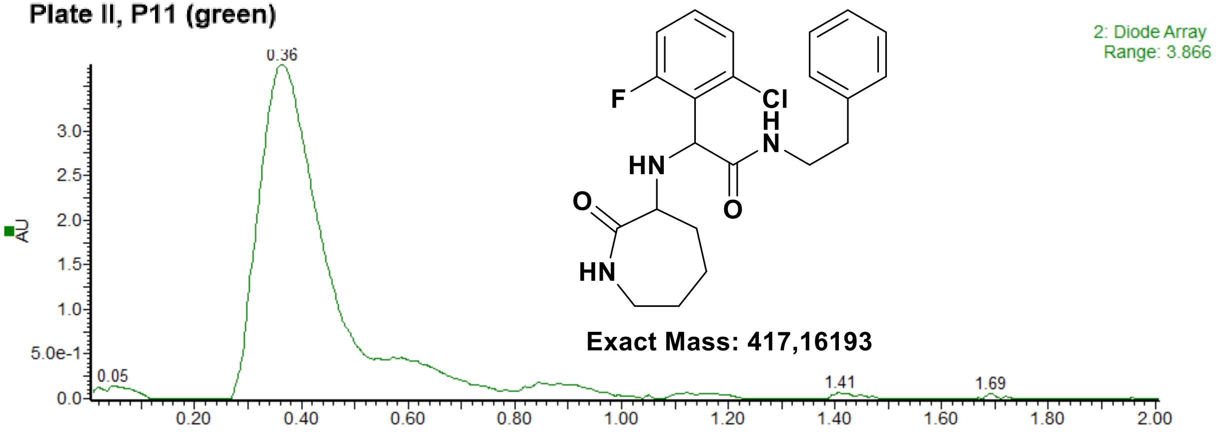

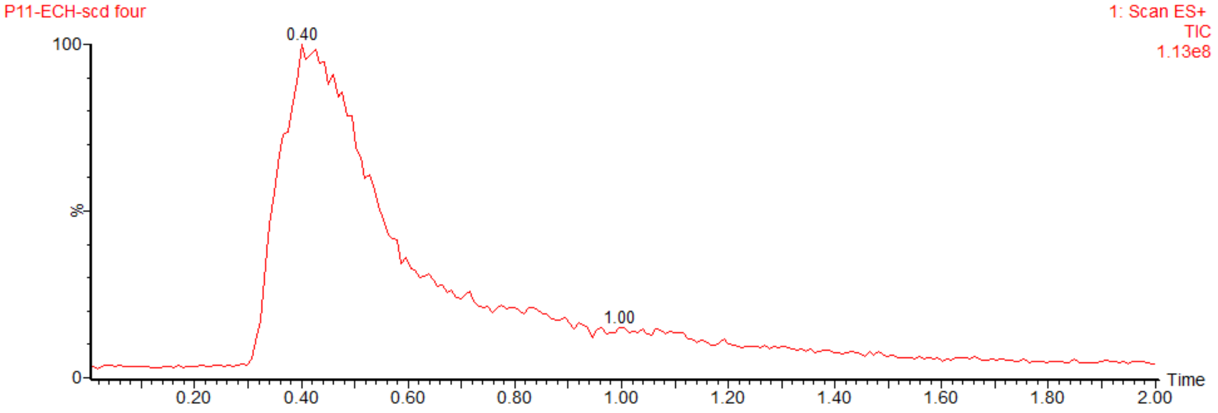

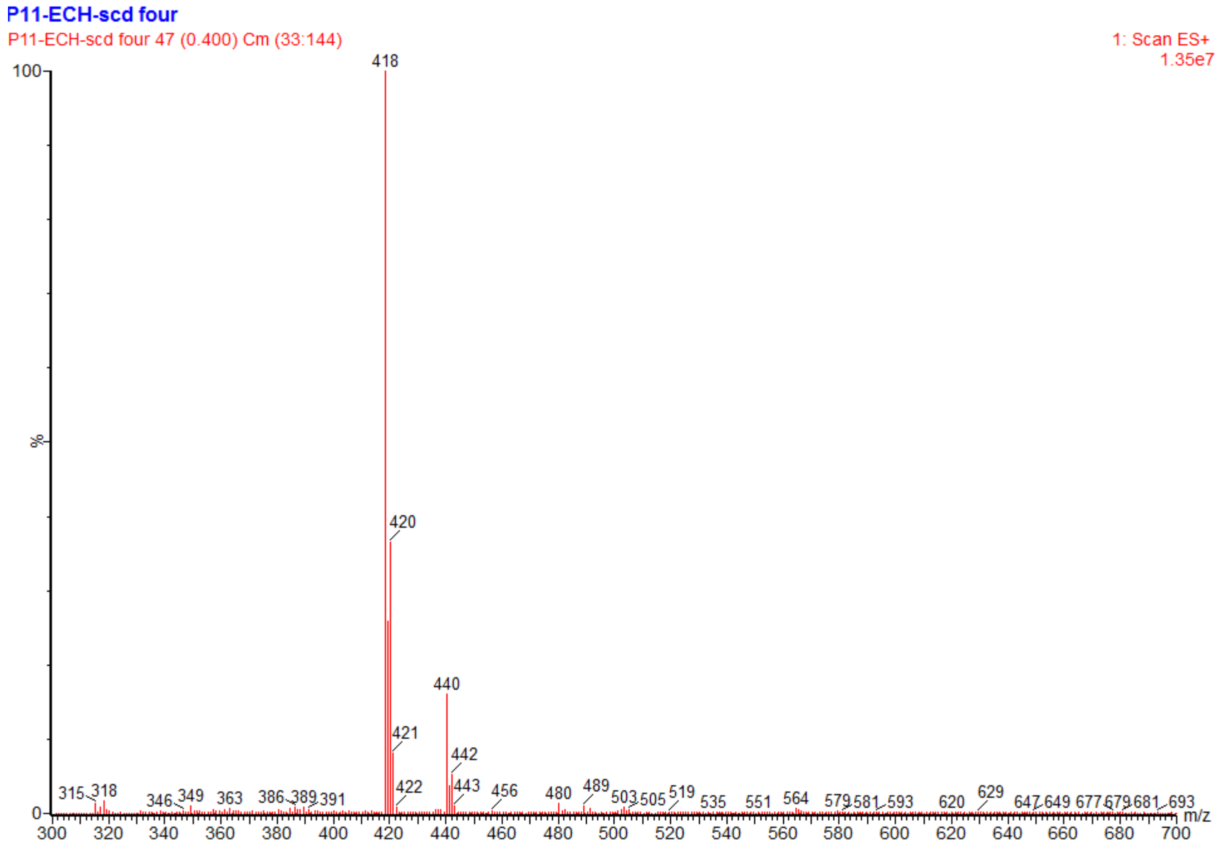

Plate III, A9 (green)

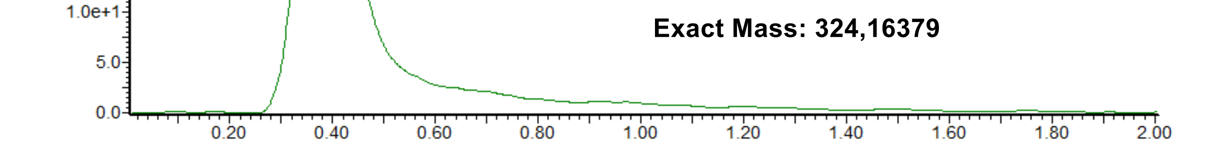

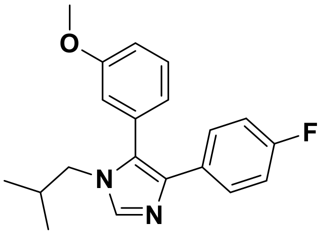

Exact Mass: 324,16379

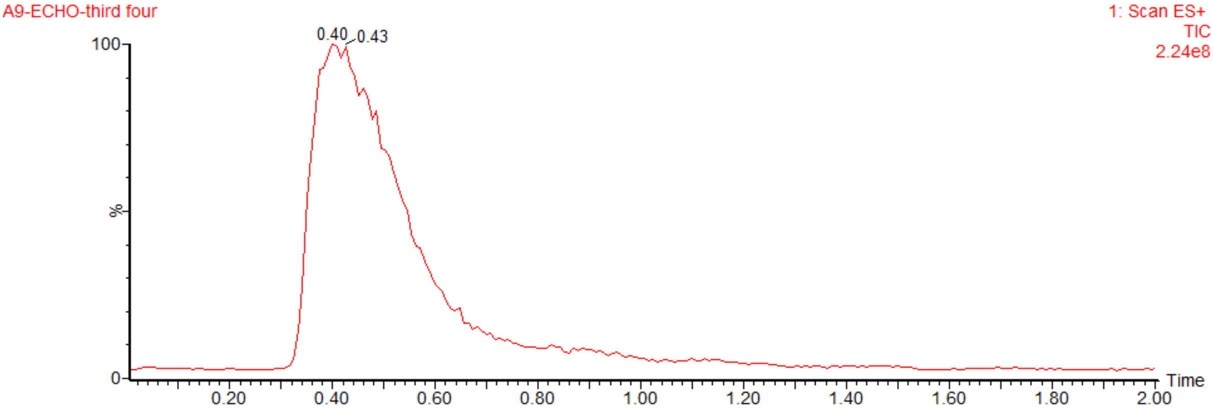

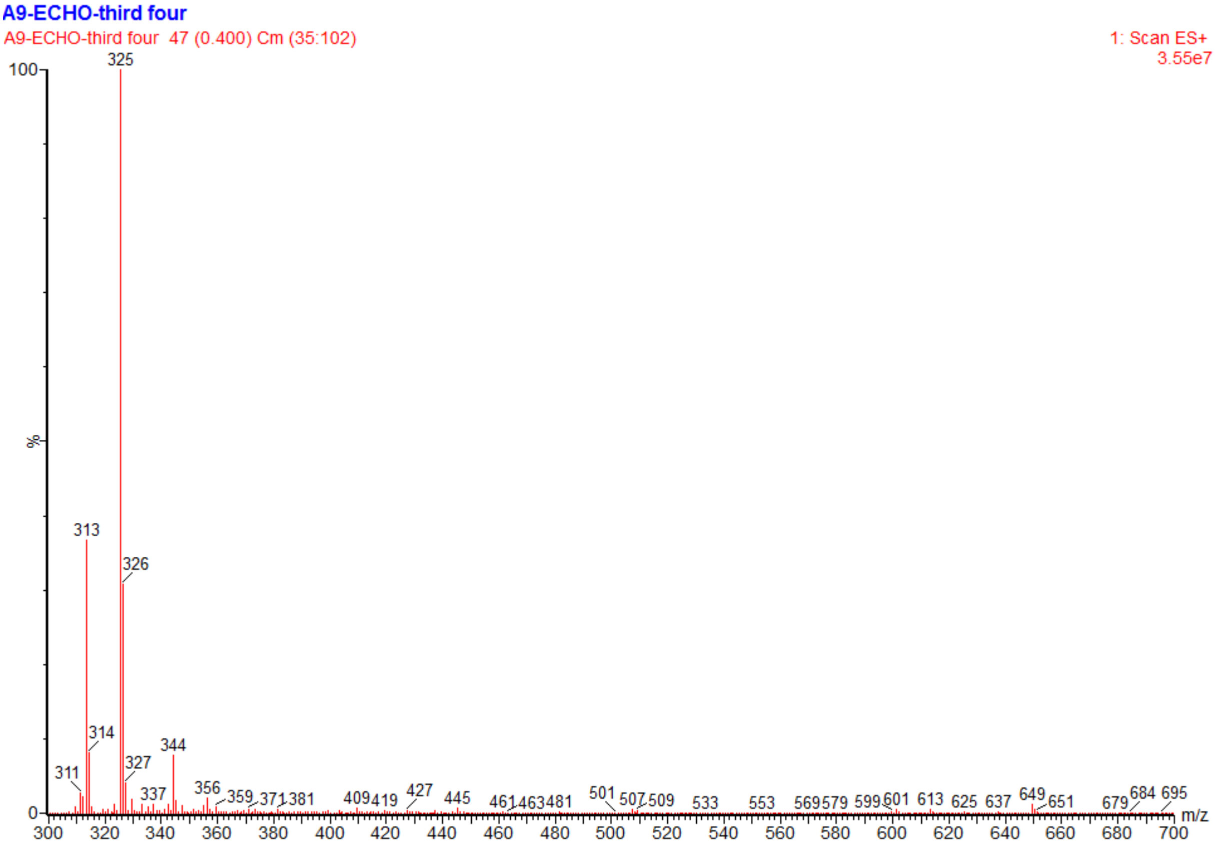

Plate III, B6 (yellow)

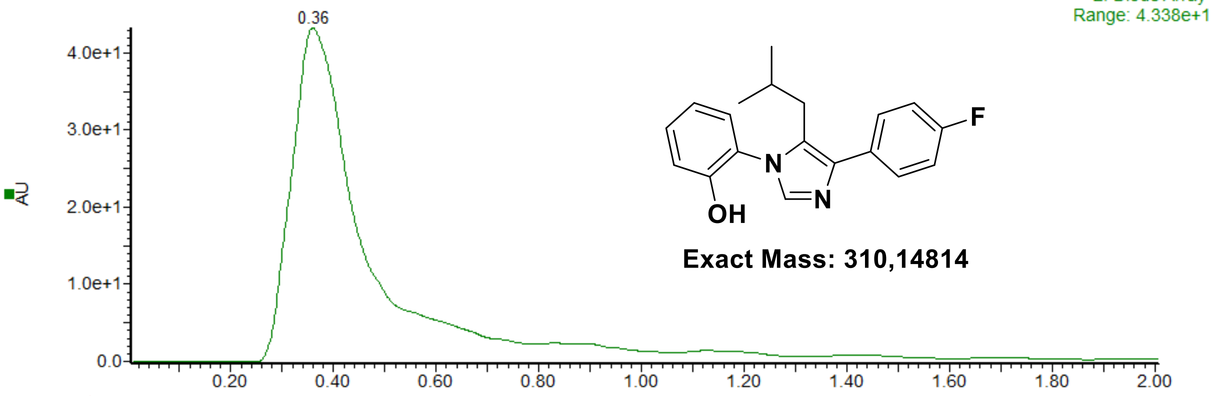

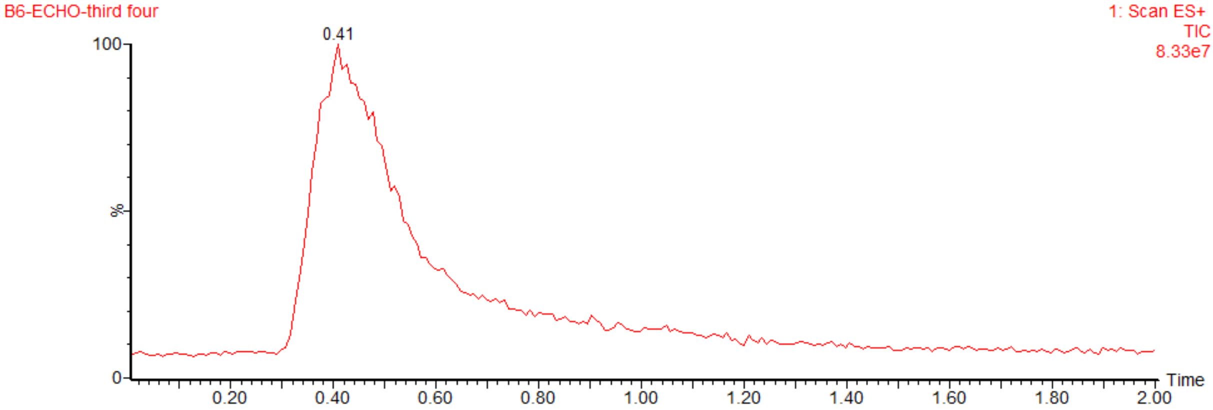

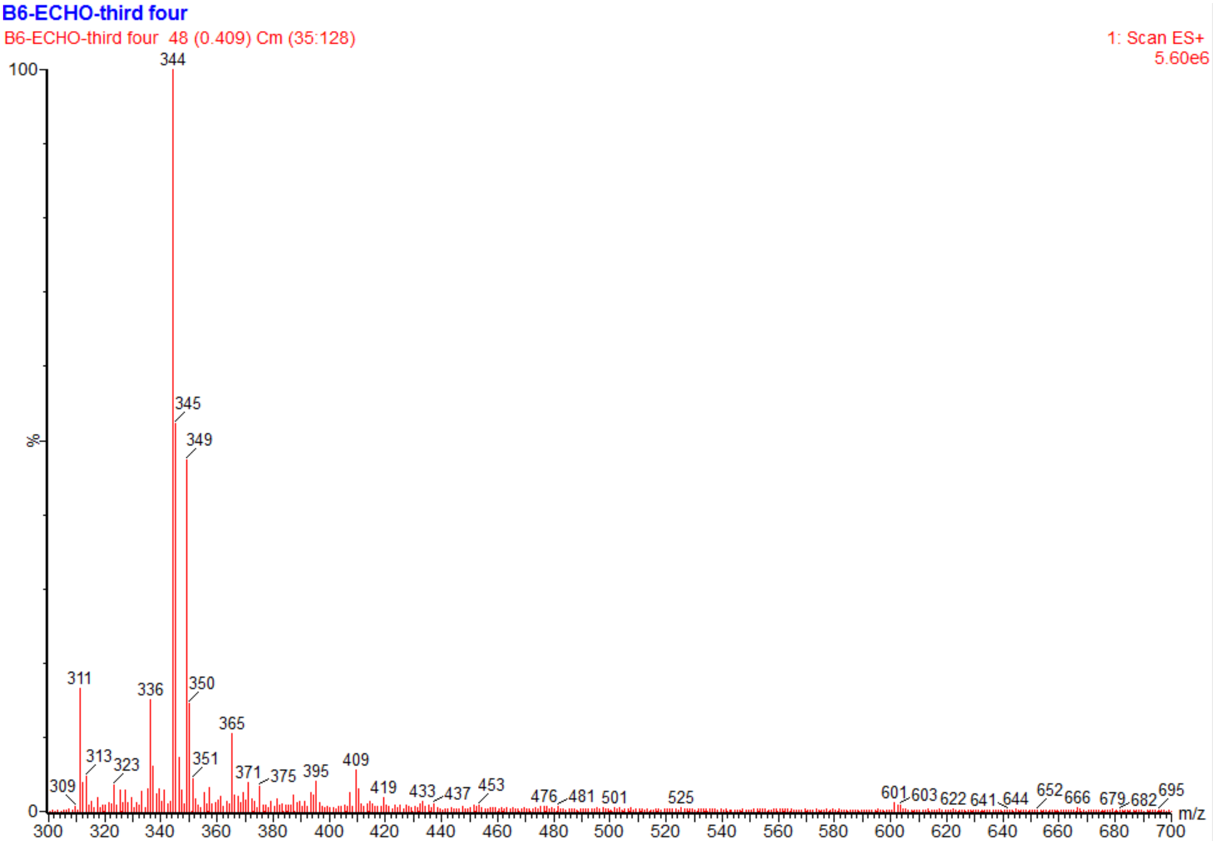

Plate III, D15 (green)

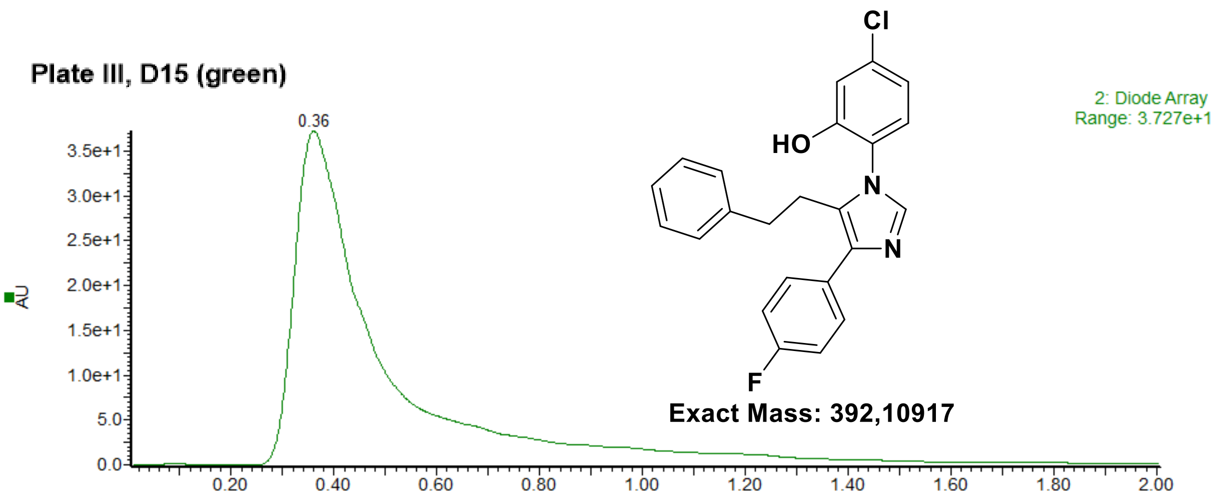

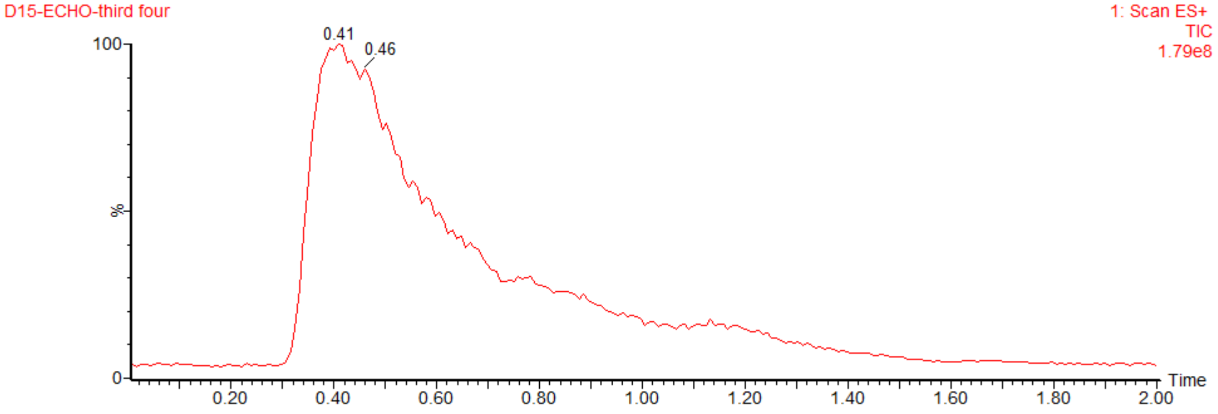

D15-ECHO-third four

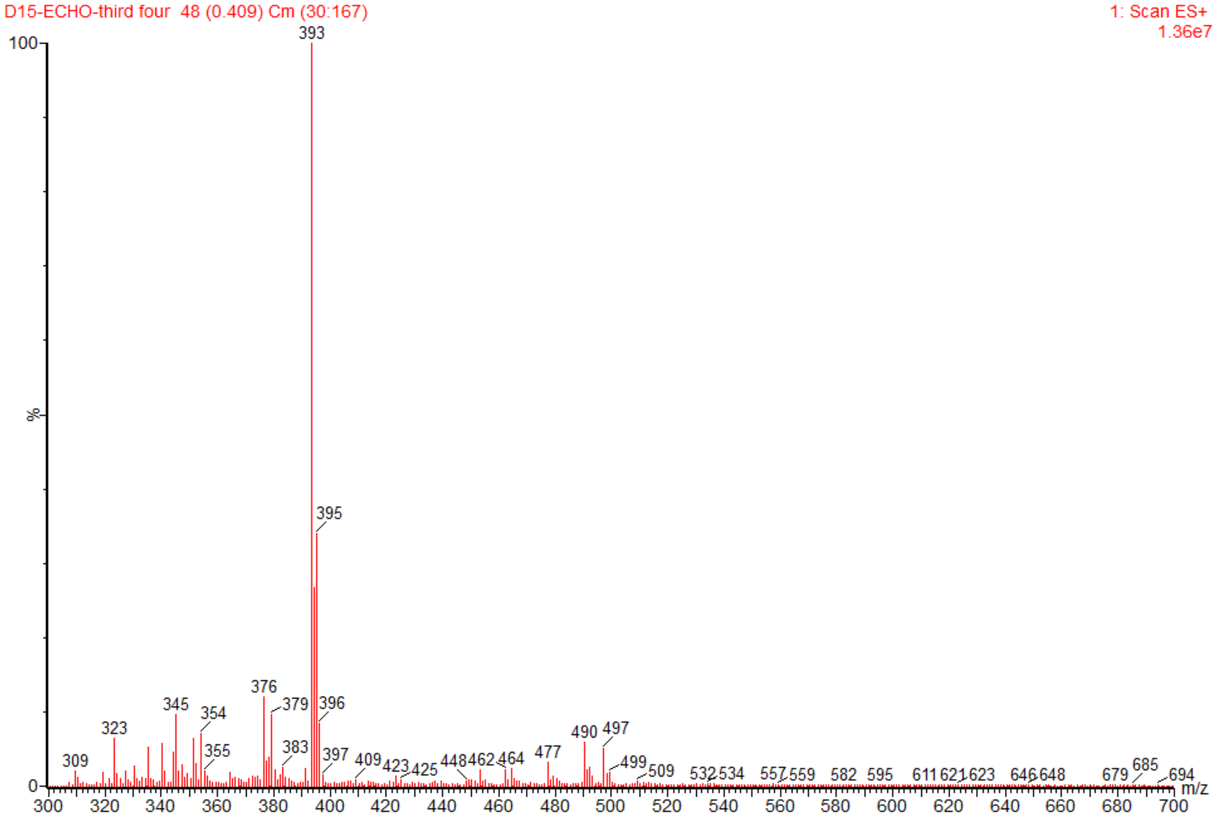

Plate IV, A15 (green)

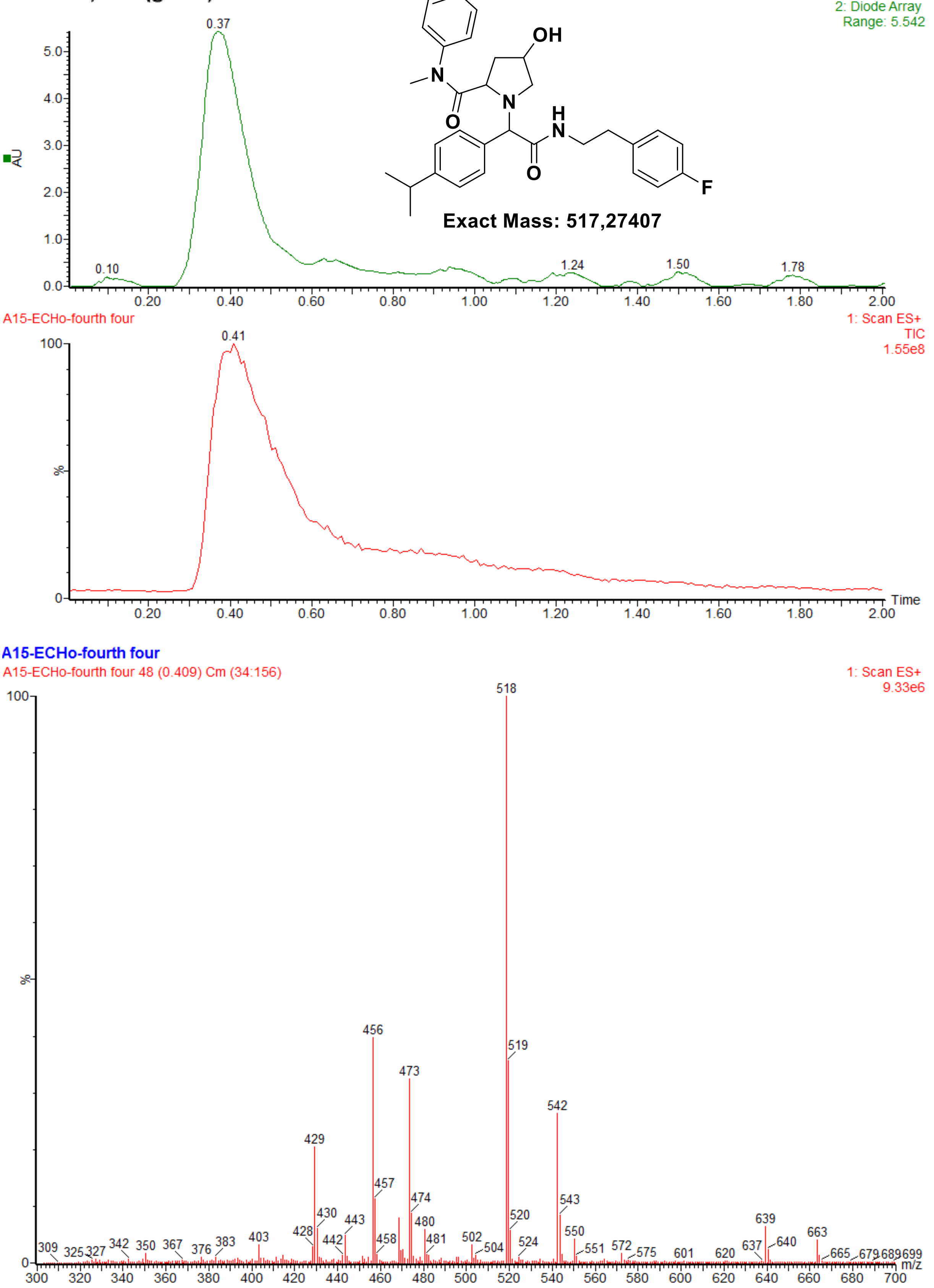

Plate IV, C20 (green)

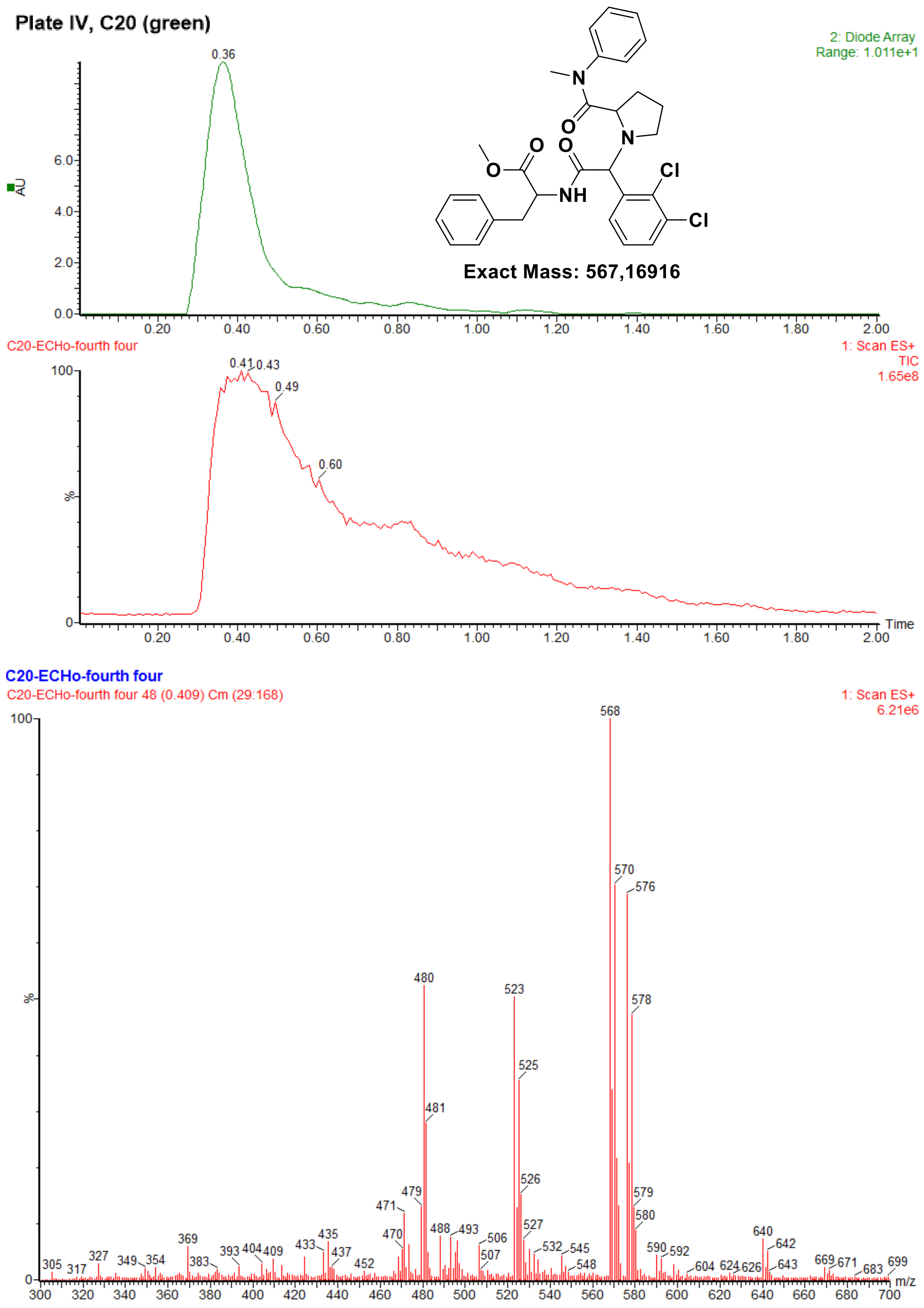

Plate IV, C22 (yellow)

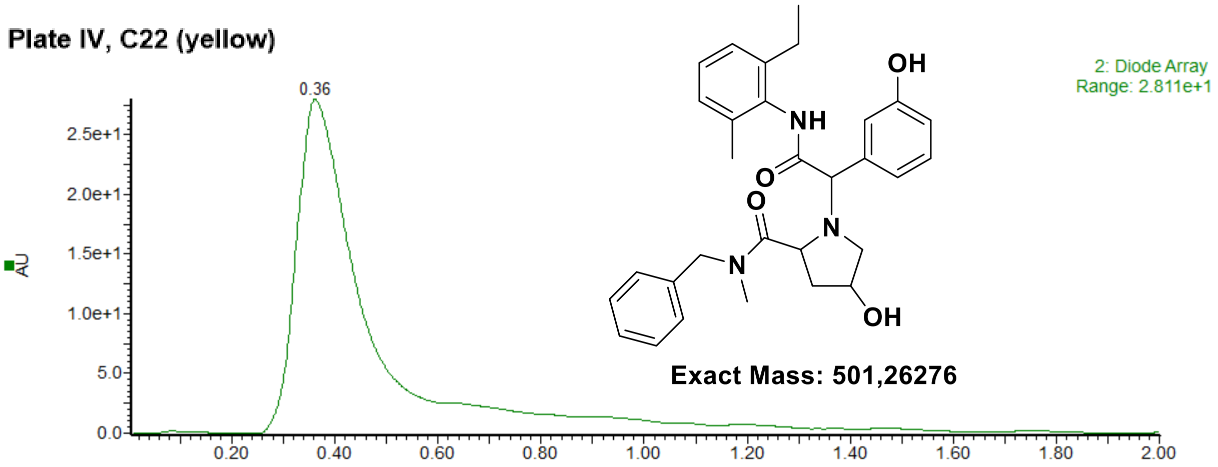

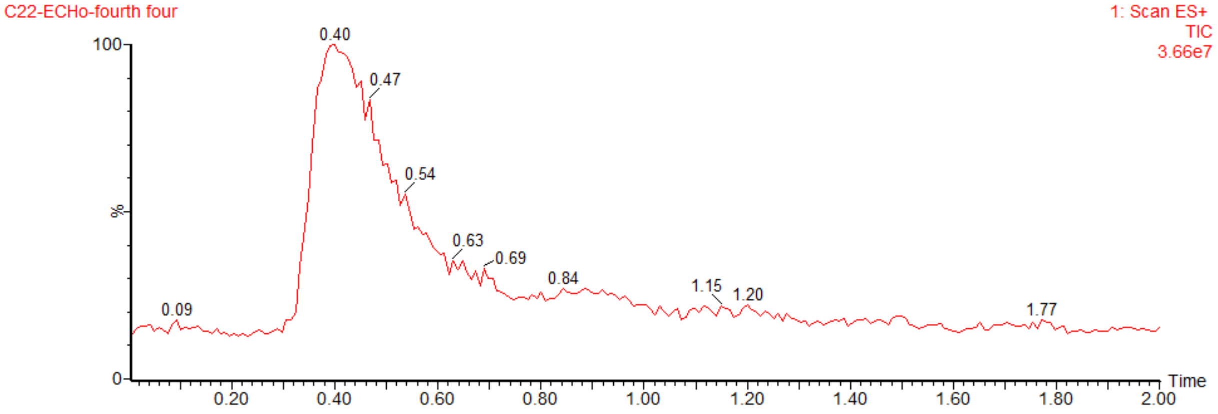

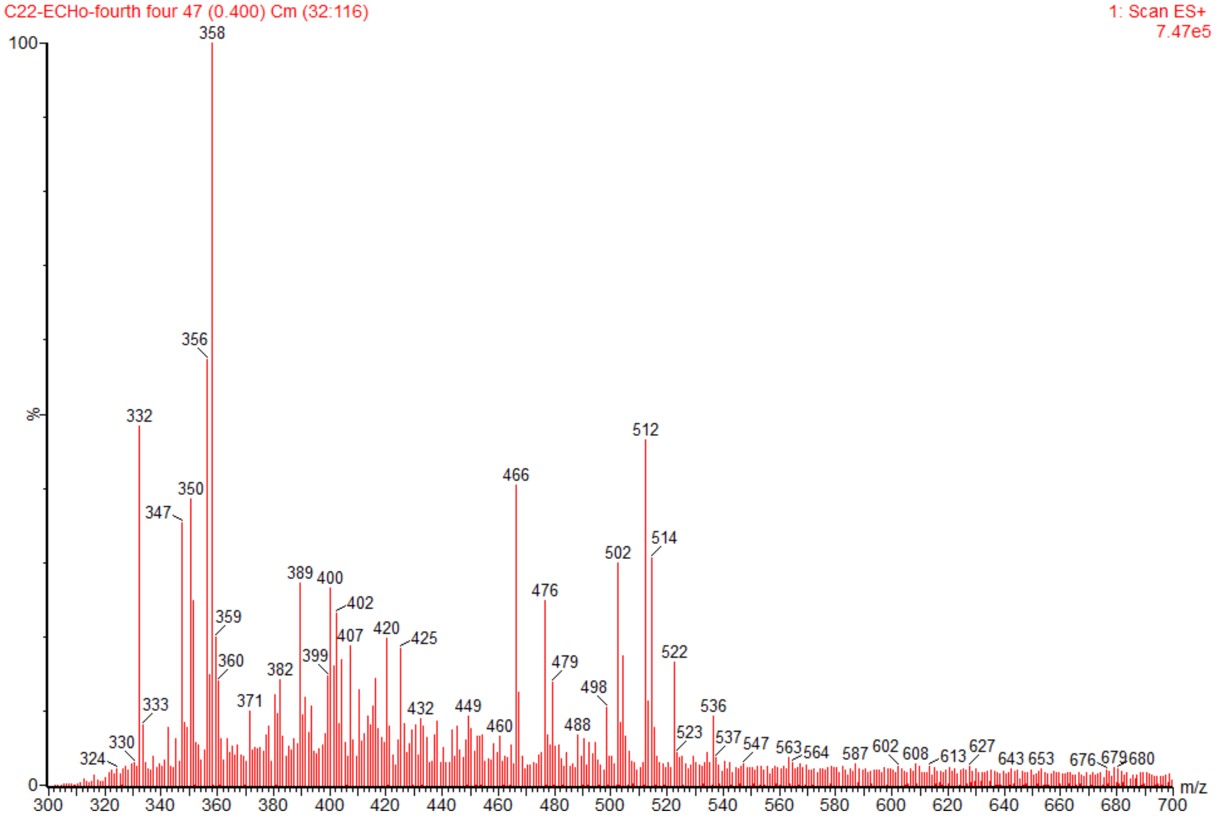

Plate IV, G4 (green)

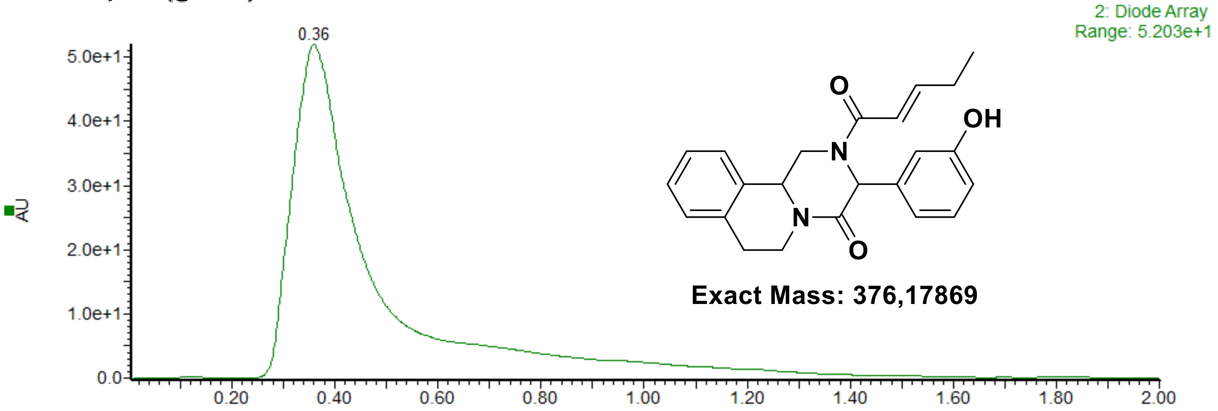

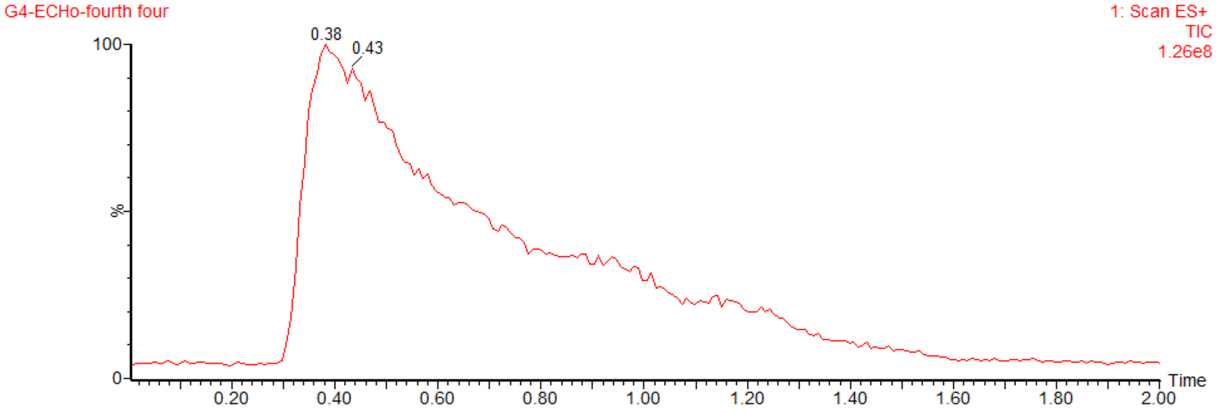

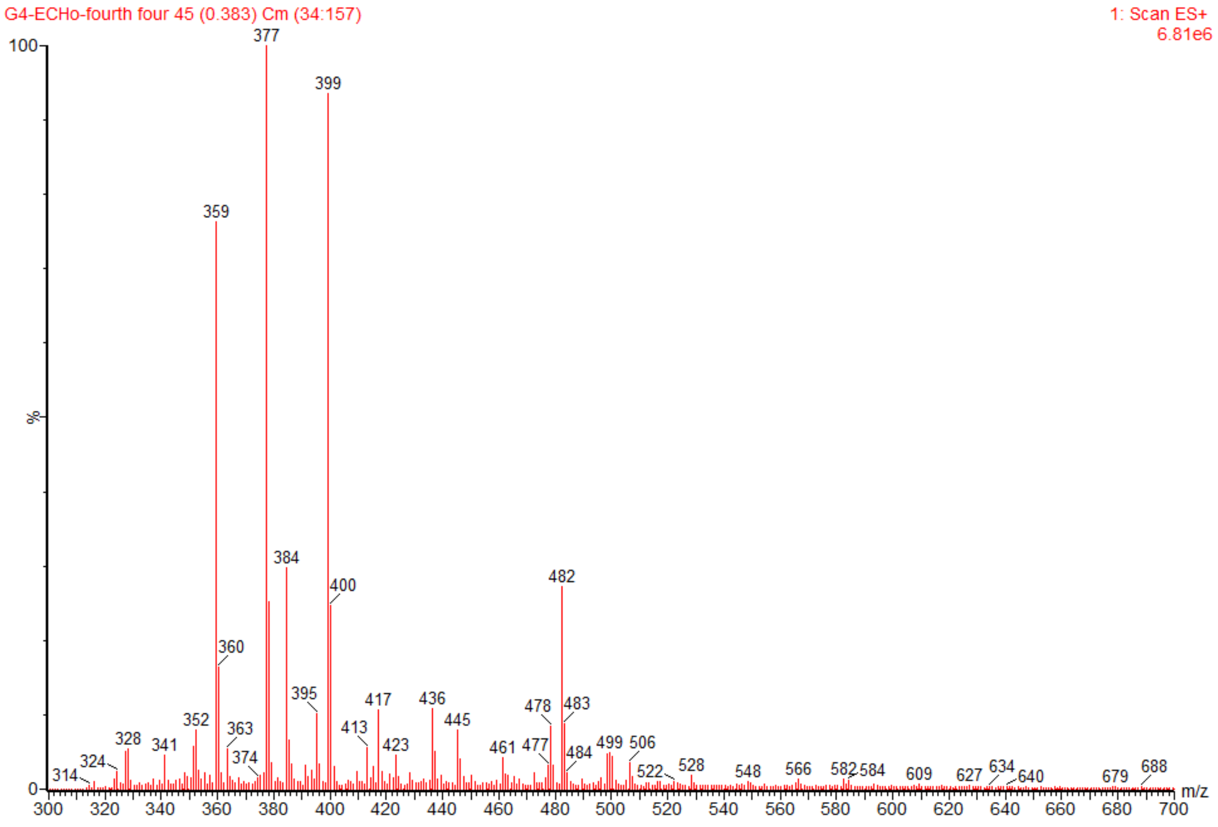

Plate IV, H12 (green)

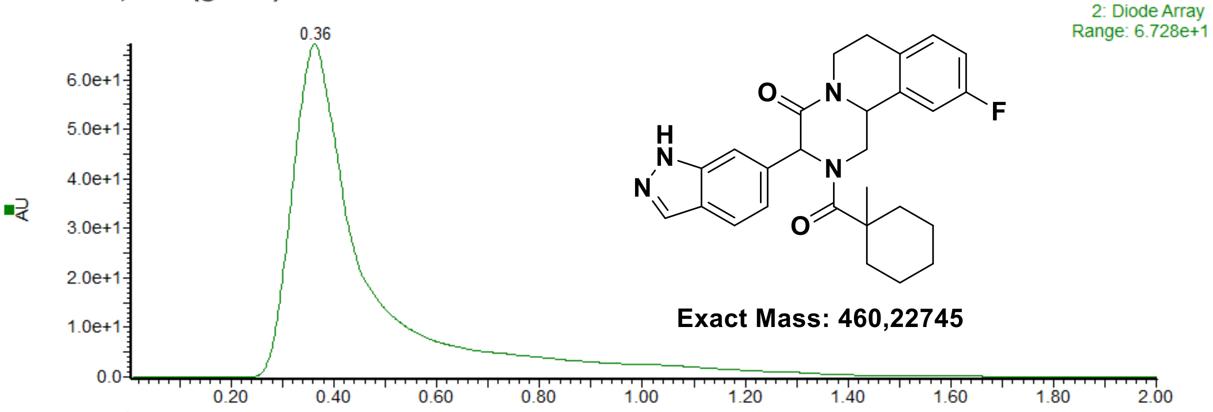

Exact Mass: 460,22745

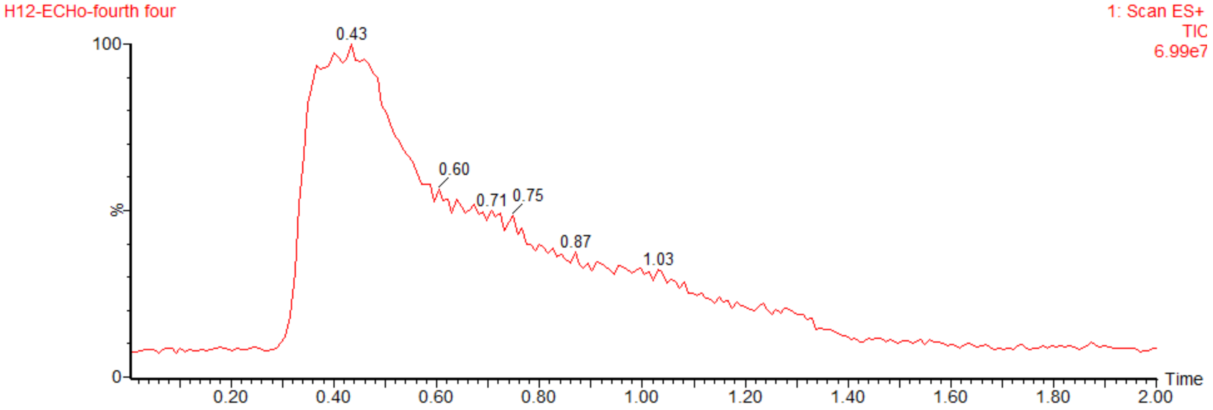

H12-ECHo-fourth four

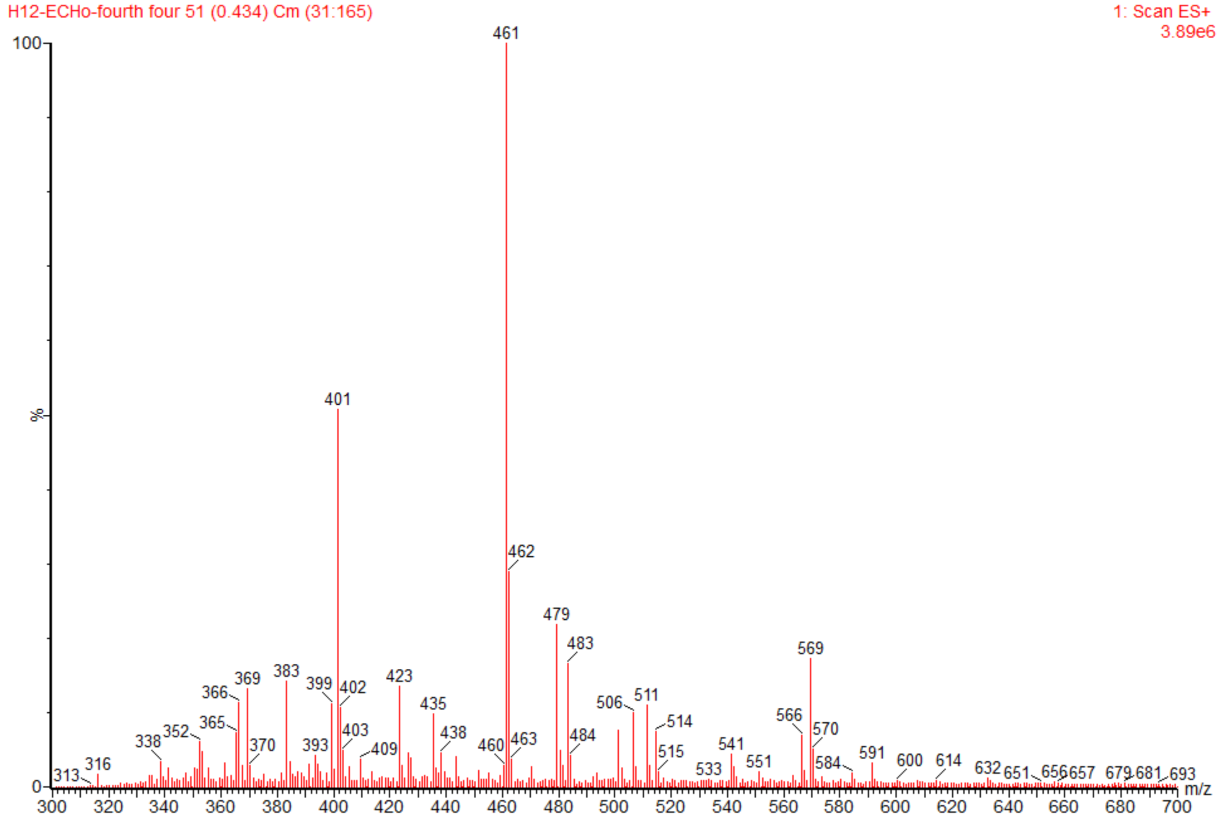

Plate IV, I21 (green)

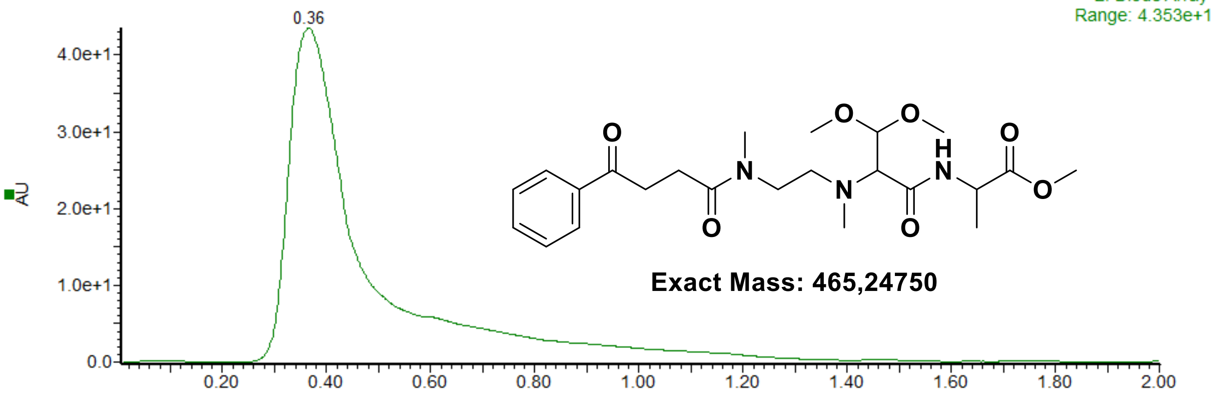

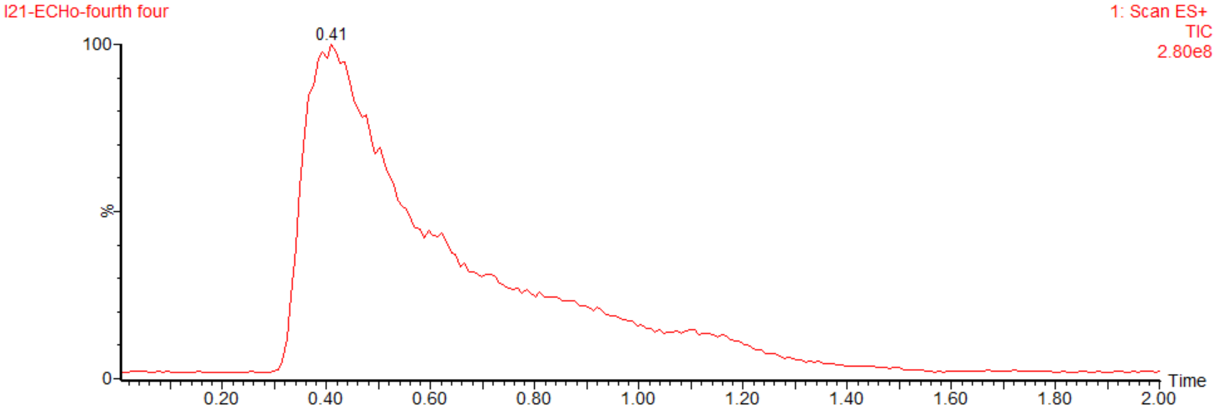

I21-ECHo-fourth four

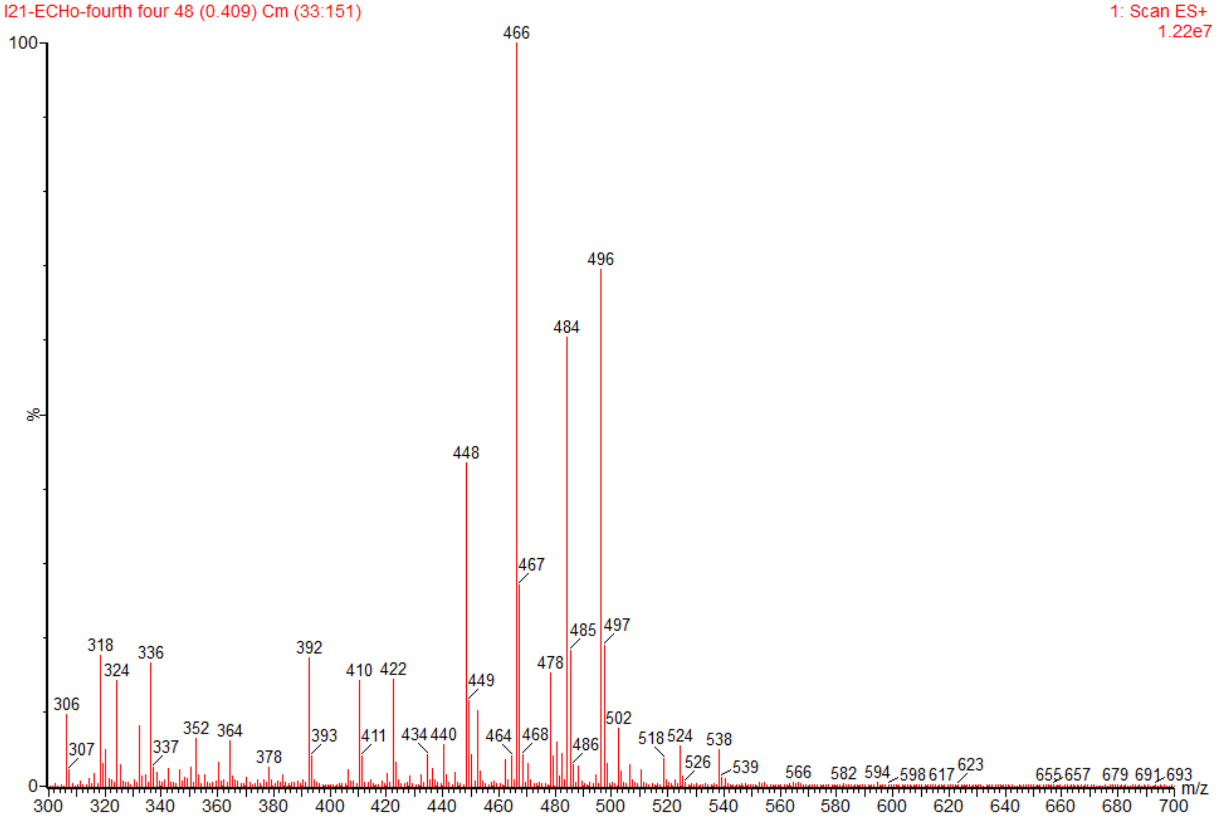

Plate IV, K2 (green)

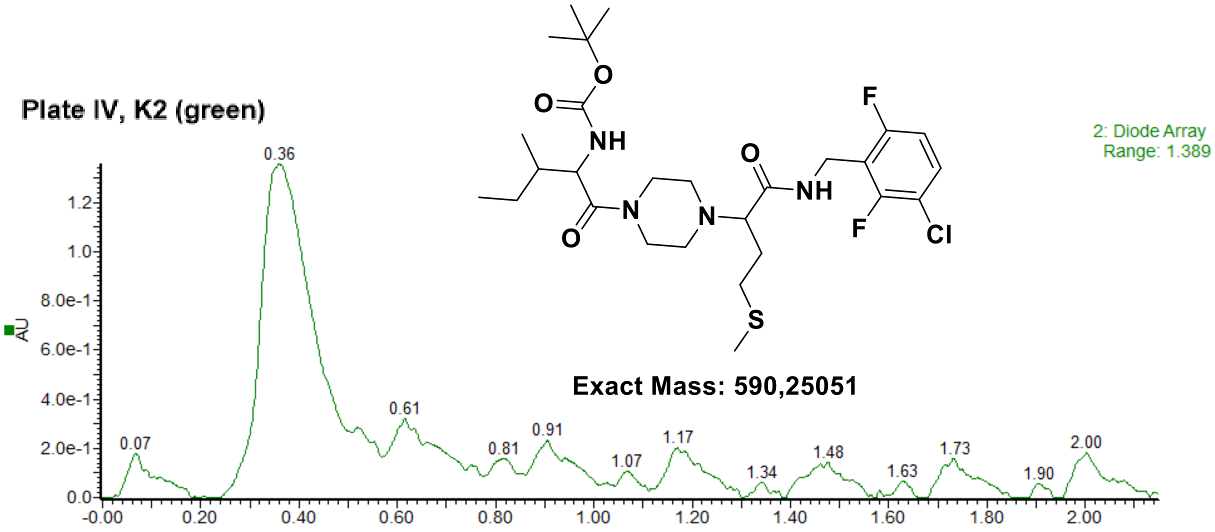

Exact Mass: 590,25051

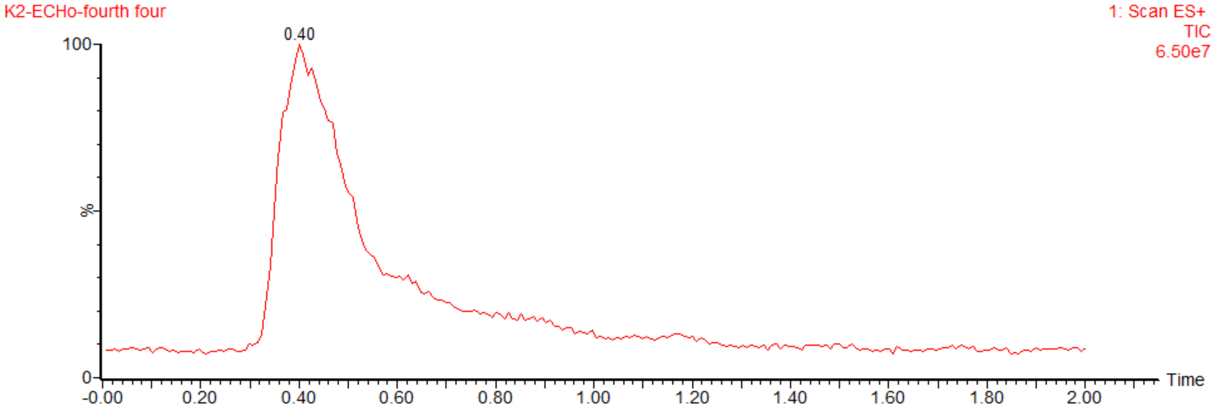

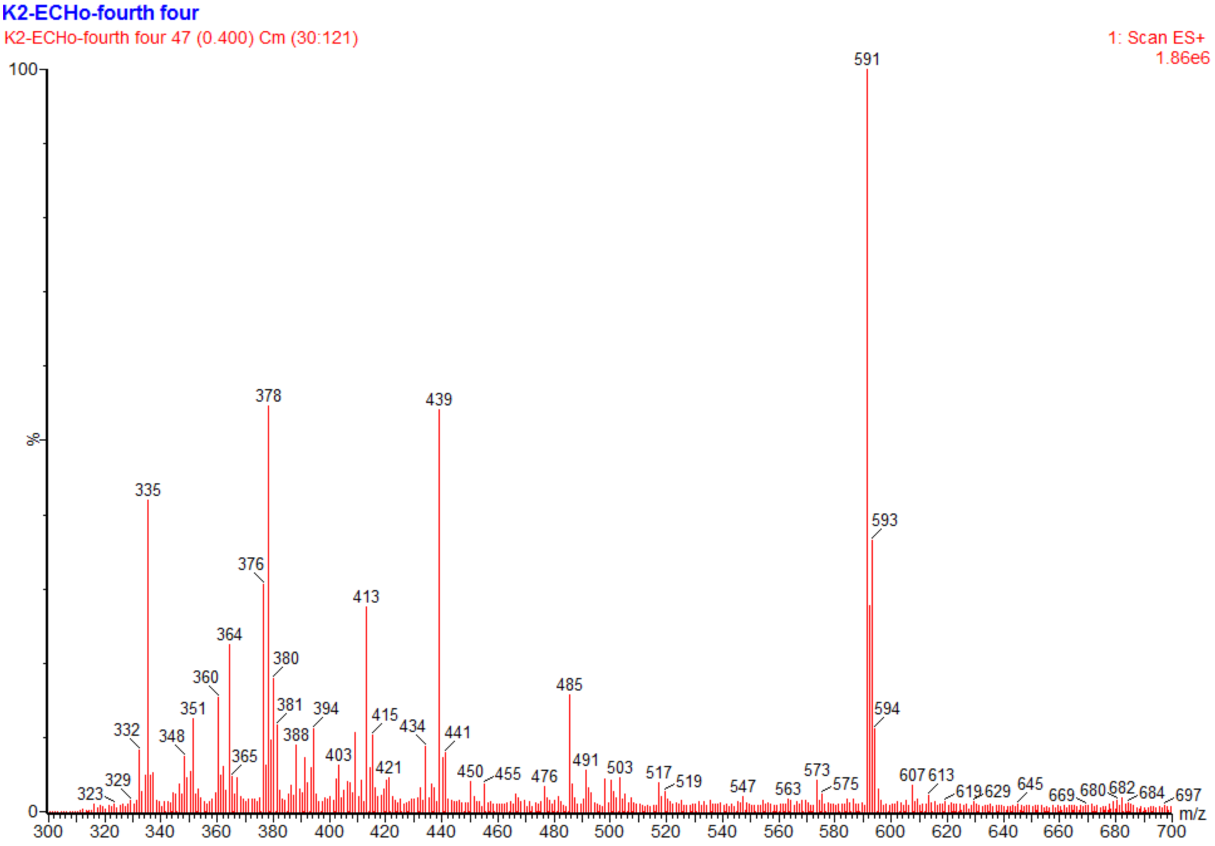

2.8. Automated analysis of mass spectrometry data

2.8.1. Preparation

Mass spectrometry (MS) data were automatically analyzed using in-house software written in Python. This software makes use of the mzXML file format that first needs to be created. For this purpose, mzXML files were converted from Waters RAW using the MSConvert tool (version 3.0) from the ProteoWizard project using the default settings. These resulting files were consecutively used in the in-house written program, which is documented in an online repository (https://bitbucket.org/ca_warmerdam/auto-ms-analysis). In addition to the mass spectrometry files, a txt file with the smiles and the location of the expected product per well on the plate were entered. These locations are used both to create an output matrix and as an identifier for matching the expected product to an mzXML file. The expected time range for the bulk of the peaks was set to 0.2-1.2 min. The expected M/Z range that was specified as 100-1000 corresponds to the range the mass spectrometer was set to detect. For running the software Python 3.6.6 was used with the additional packages which were in concordance with the requirements specified within the repository.

2.8.2. Processing mzXML files into spectra

Within the python software, mzXML files are first parsed into a queryable data structure using the Python XML parser module. Next, the MS data, comprised of scans that together represent the spectrum, are filtered in order to remove uninformative scans that are labelled with an msLevel of 0. In addition, scans that are outside of the specified time range are discarded. The contents of the scans are subsequently decoded as these are Base64 encoded by default, and the resulting values are thereafter decompressed using the decompression functionality within the zlib Python module. This results in a regular collection of pairs consisting of a mass-to-charge ratio with corresponding intensities. Intensity values were considered erroneous, and are thus removed, if they expand further than 5000 times the inter quantile range of the intensities within the specific well. Afterwards, the mass-to-charge ratios are collected in bins of size 1 around an integer value. Within this process, the intensity values are summed for every bin.

2.8.3. Prediction procedure

To assign a prediction of abundance for a product, from the peaks corresponding to all user-specified adduct masses (M+H, M+Na, M+K peaks) (± 0.5), the highest peak within a well is isolated. The intensity of this peak relative to the highest peak represents the initial prediction for a product. The values are being rounded to one decimal place. After having run the software, predictions with values equal to 1 were classified as green, predictions with values equal to 0 were classified as blue, and predictions with values between these thresholds were classified as yellow.

2.8.4. Python code

Please refer to our previous publication.[25]

2.9. Heat maps

Destination plate I

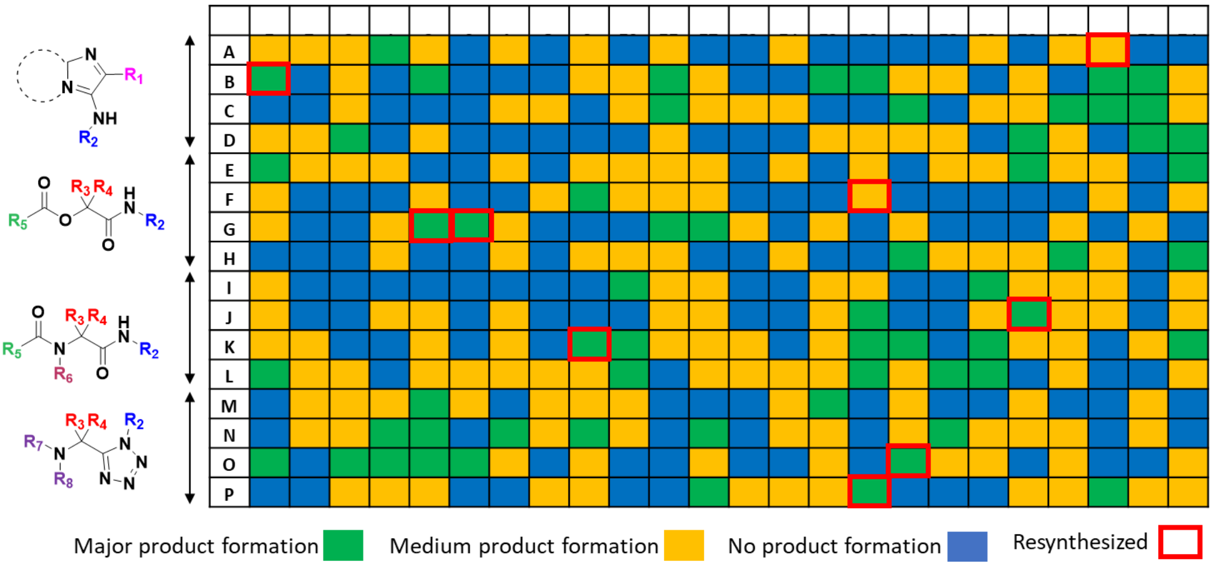

Destination plate II

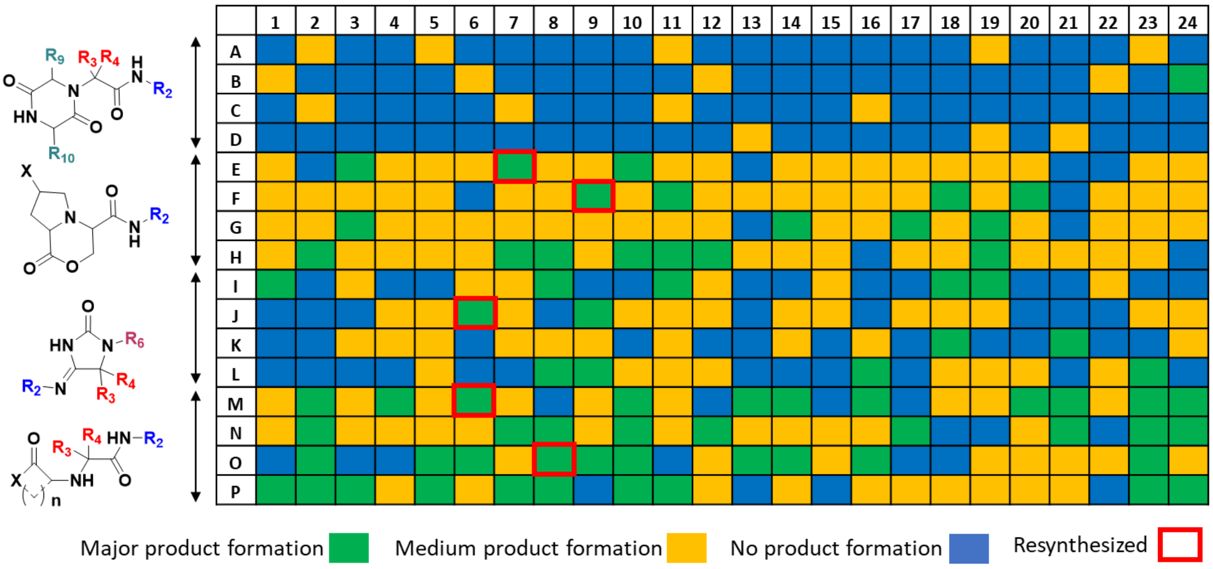

Destination plate III

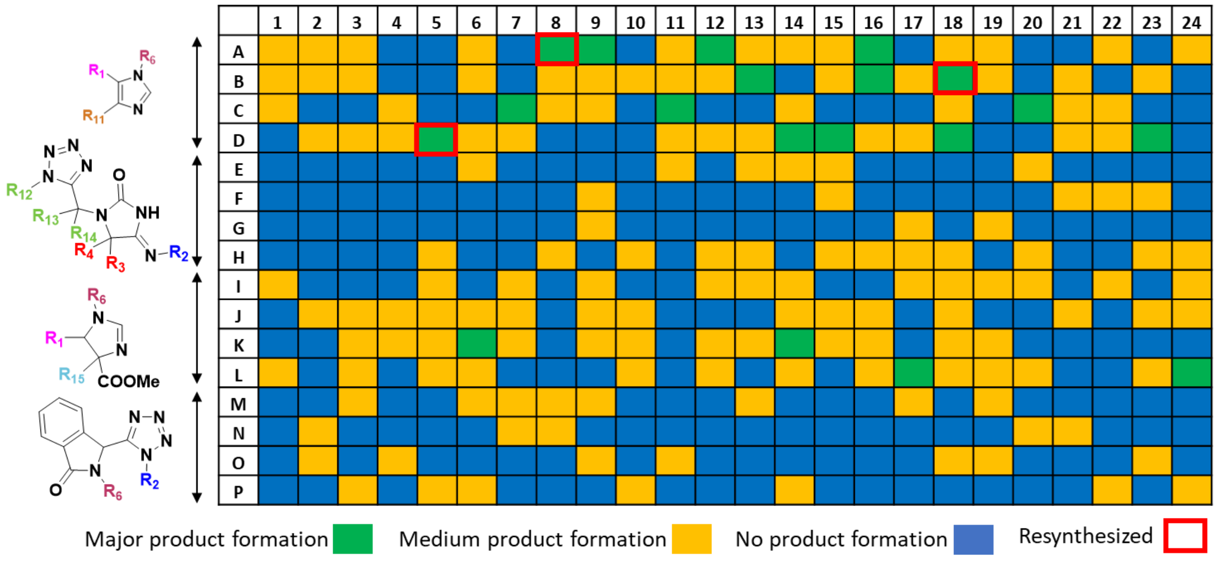

Destination plate IV

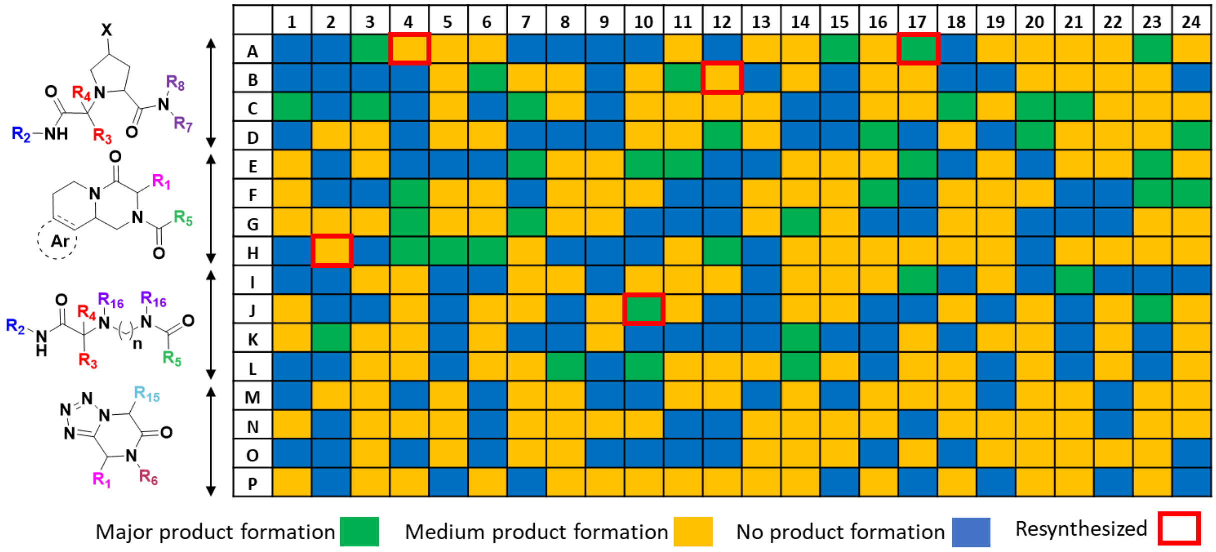

3. Statistical reaction analysis

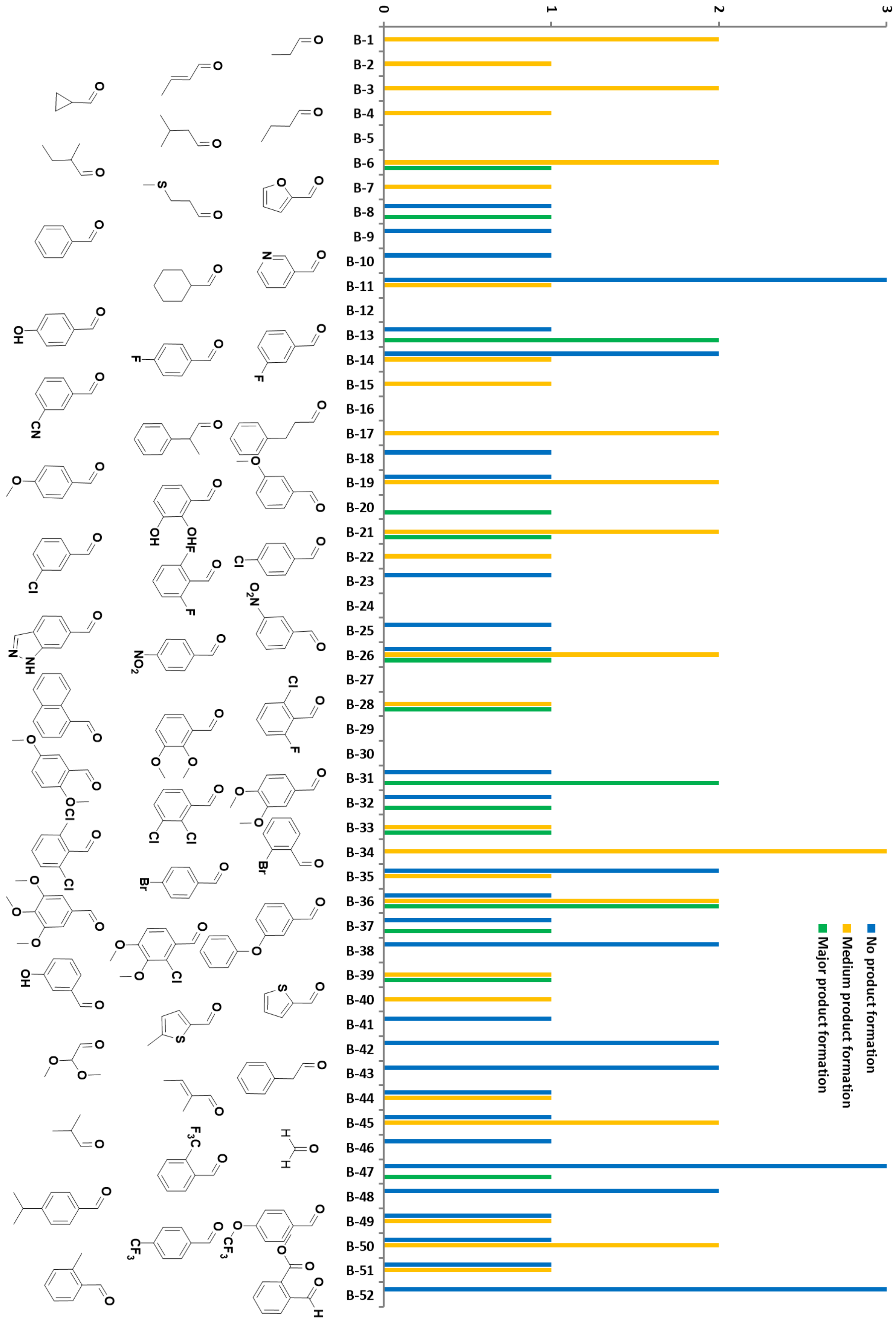

Scheme S1. Performance of aldehydes in GBB-3CR in destination plate I.

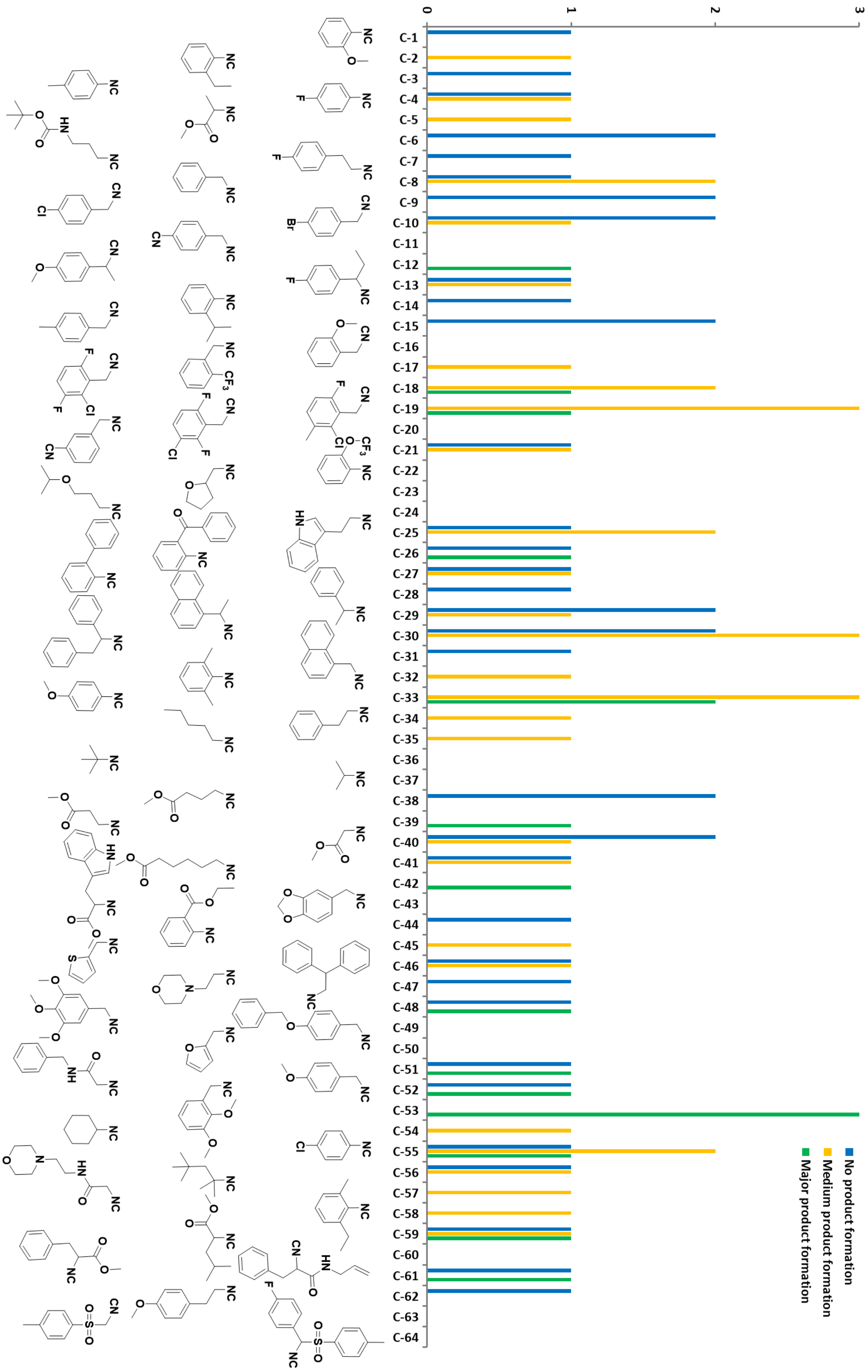

Scheme S2. Performance of isocyanides in GBB-3CR in destination plate I.

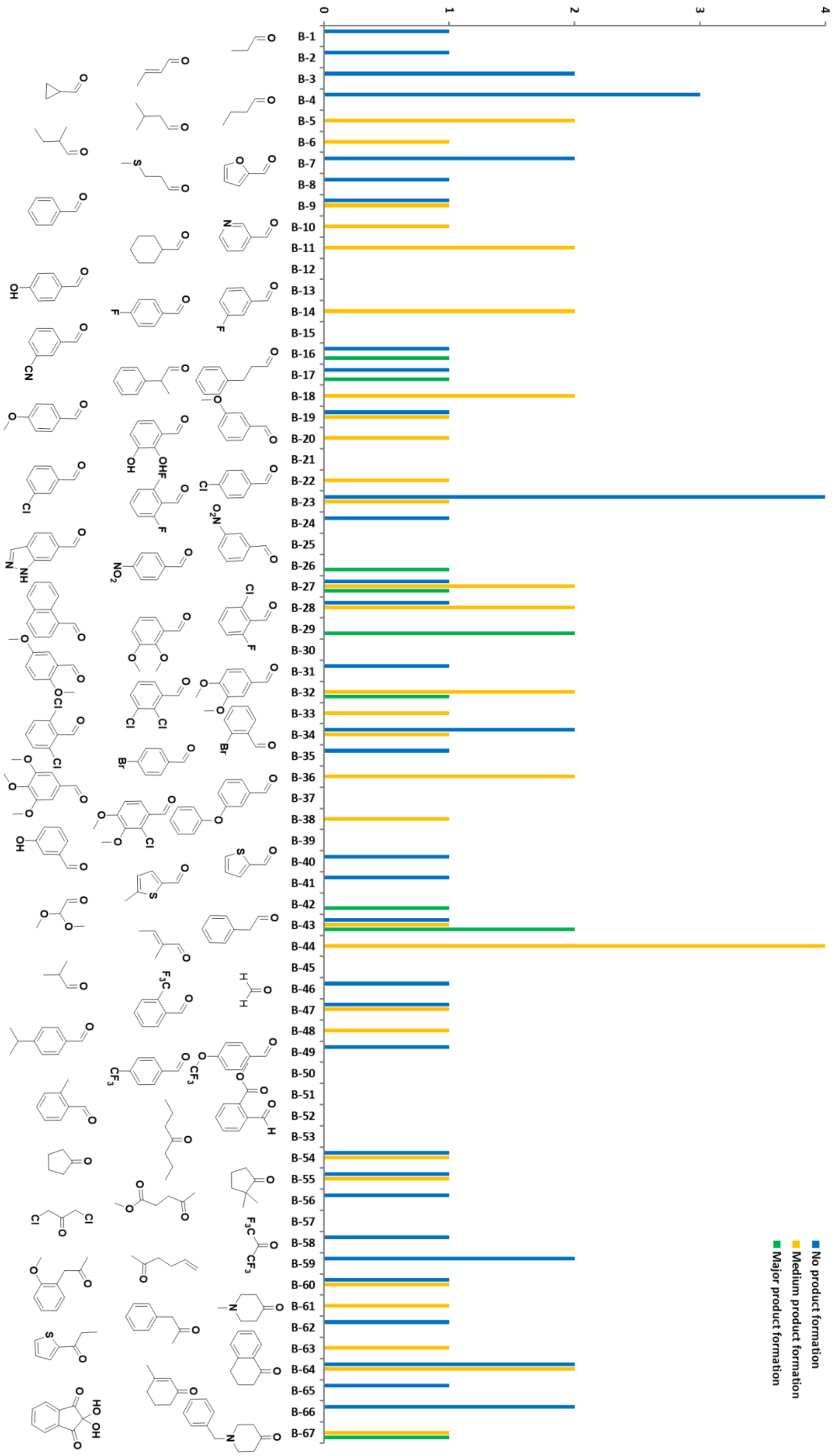

Scheme S3. Performance of oxo components in P-3CR in destination plate I.

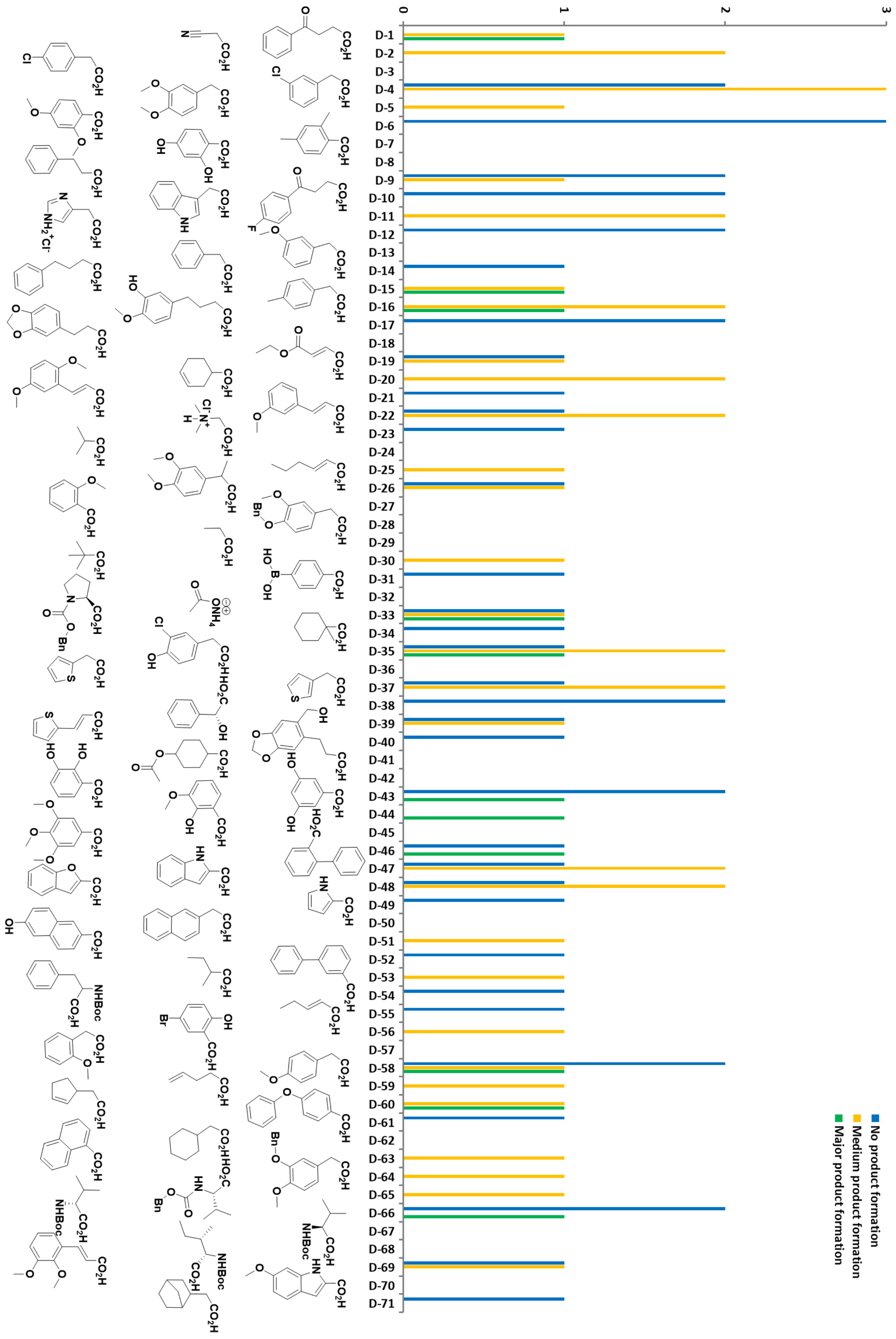

Scheme S4. Performance of carboxylic acids in P-3CR in destination plate I.

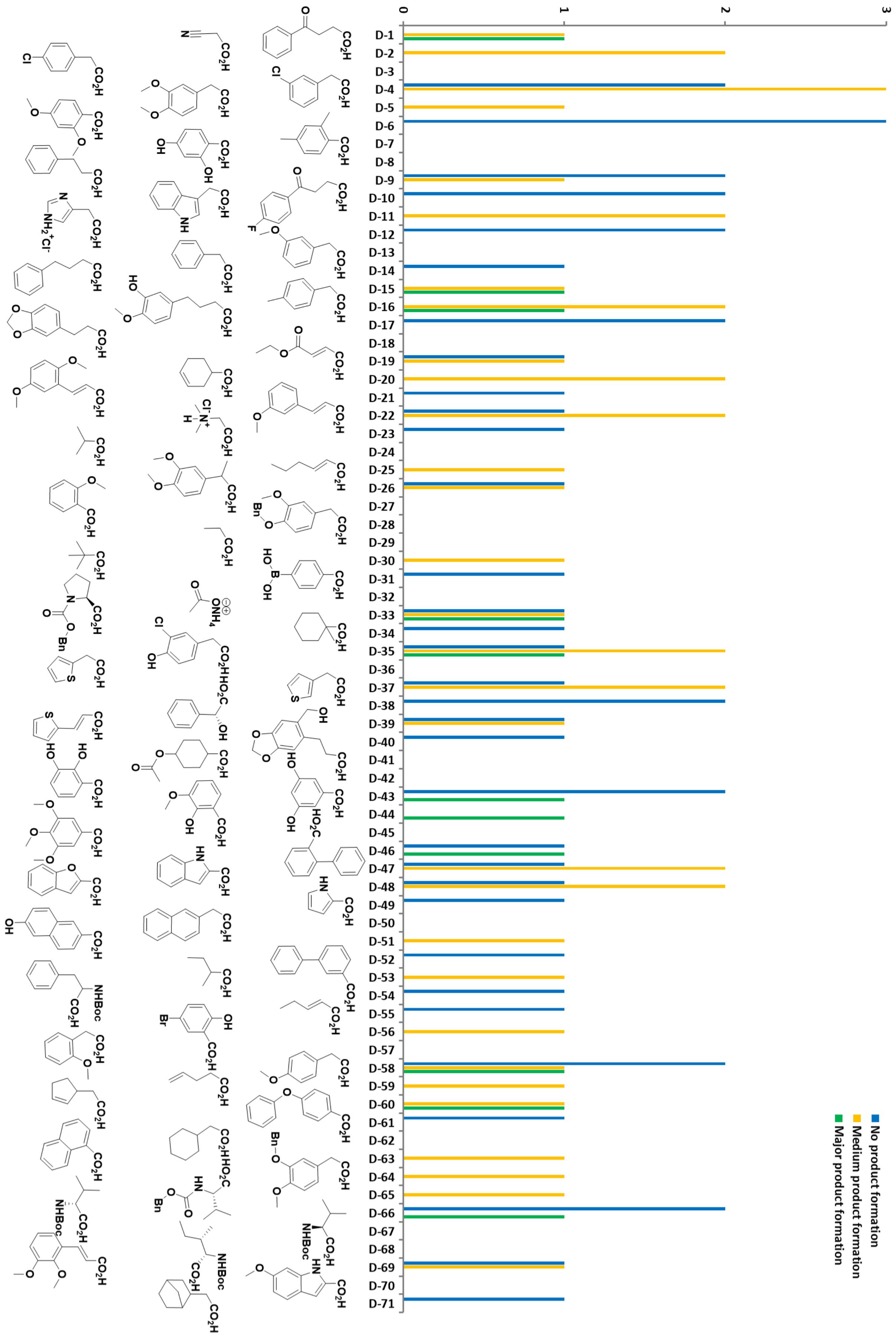

Scheme S5. Performance of isocyanides in P-3CR in destination plate I.

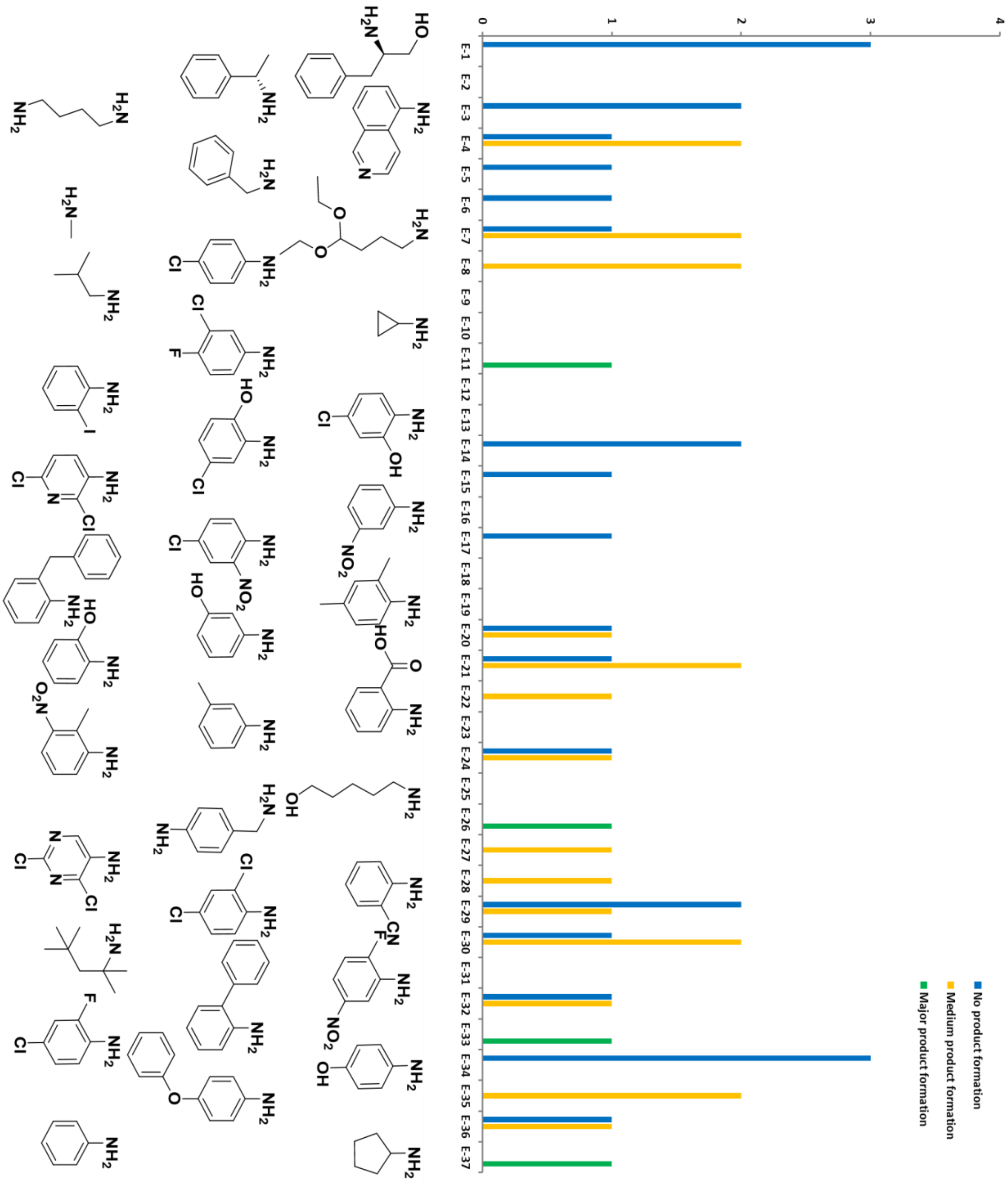

Scheme S6. Performance of amines in U-4CR in destination plate I, wells I1-J24.

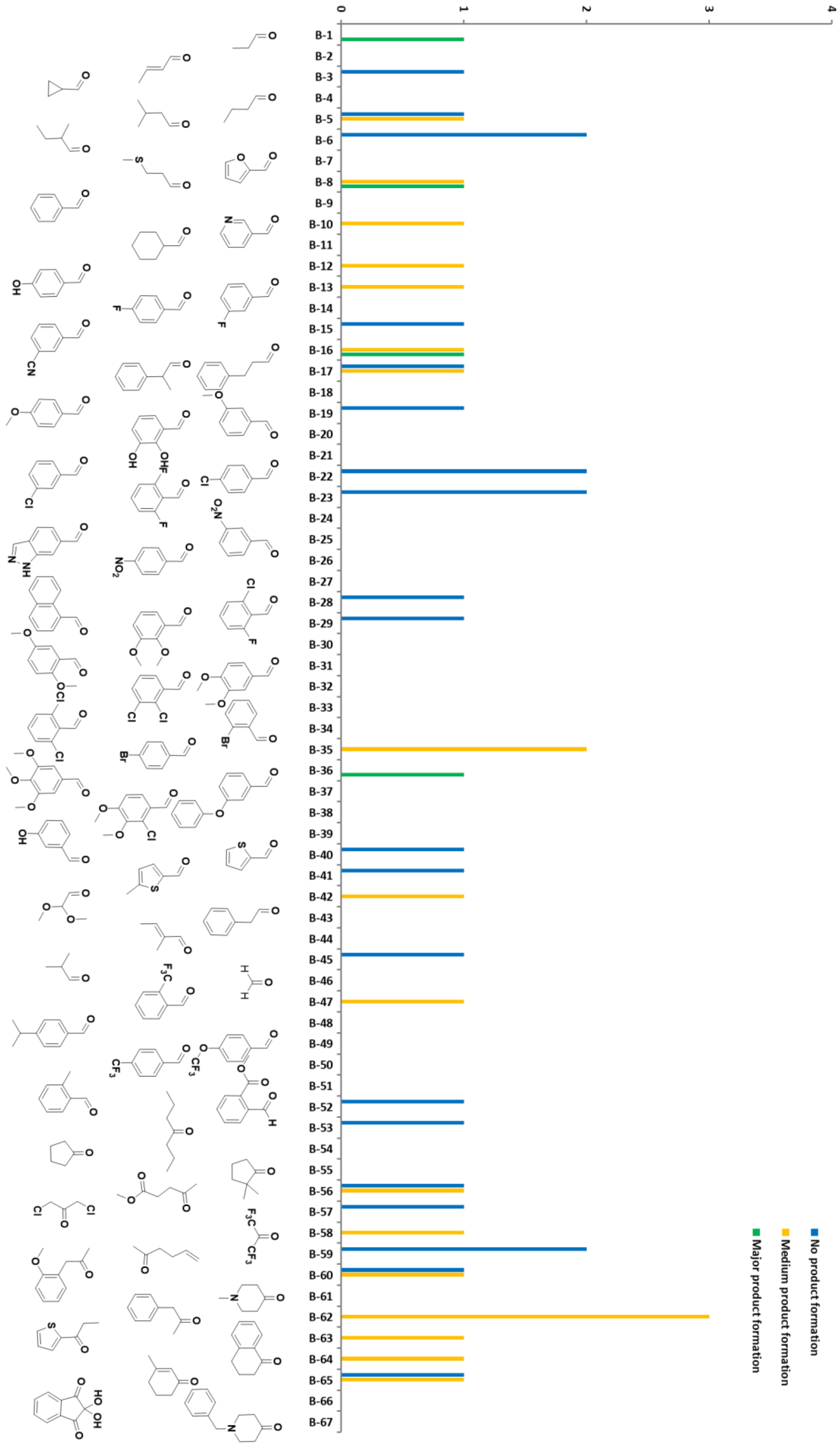

Scheme S7. Performance of oxo components in U-4CR in destination plate I, wells I1-J24.

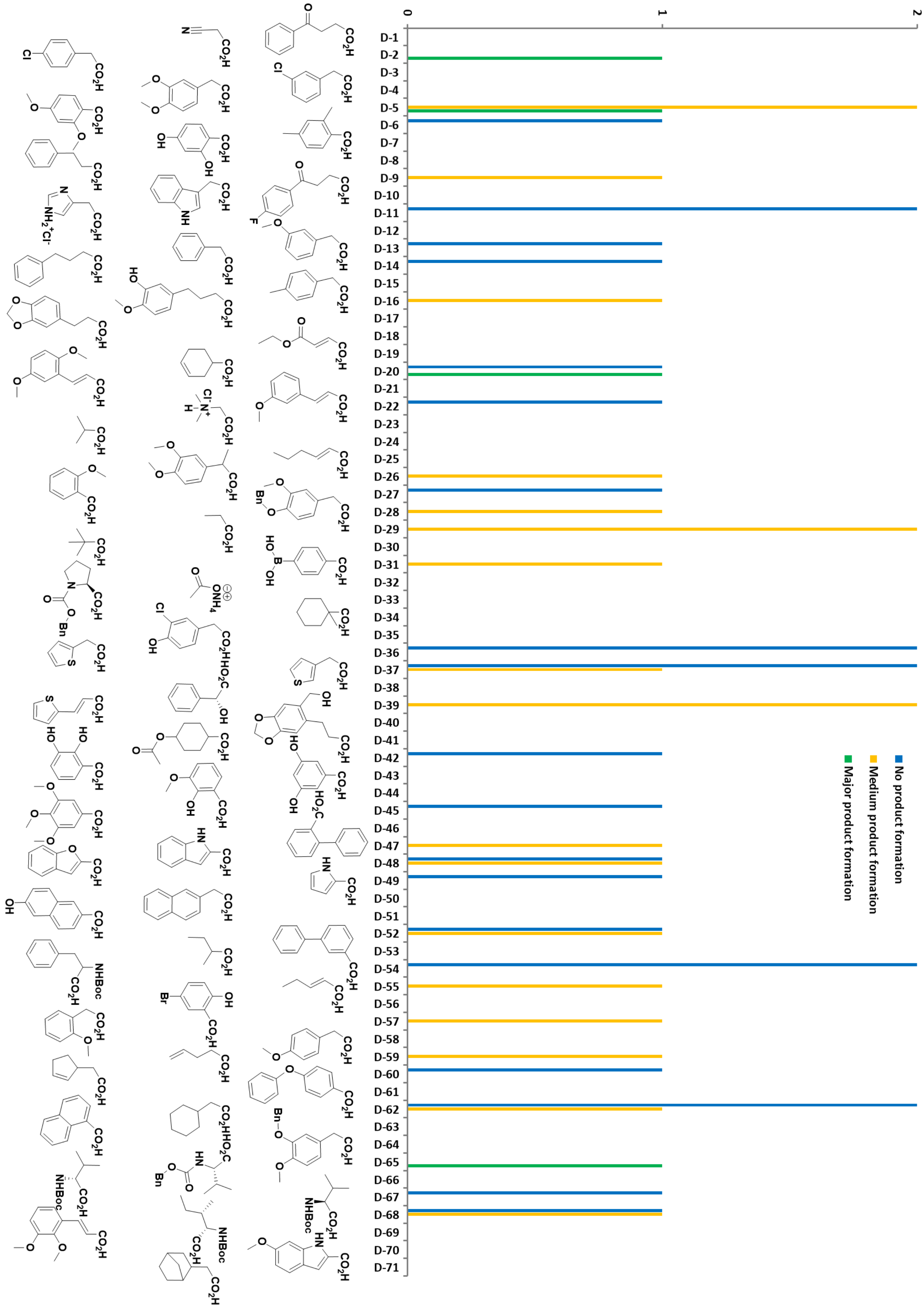

Scheme S8. Performance of carboxylic acids in U-4CR in destination plate I, wells I1-J24.

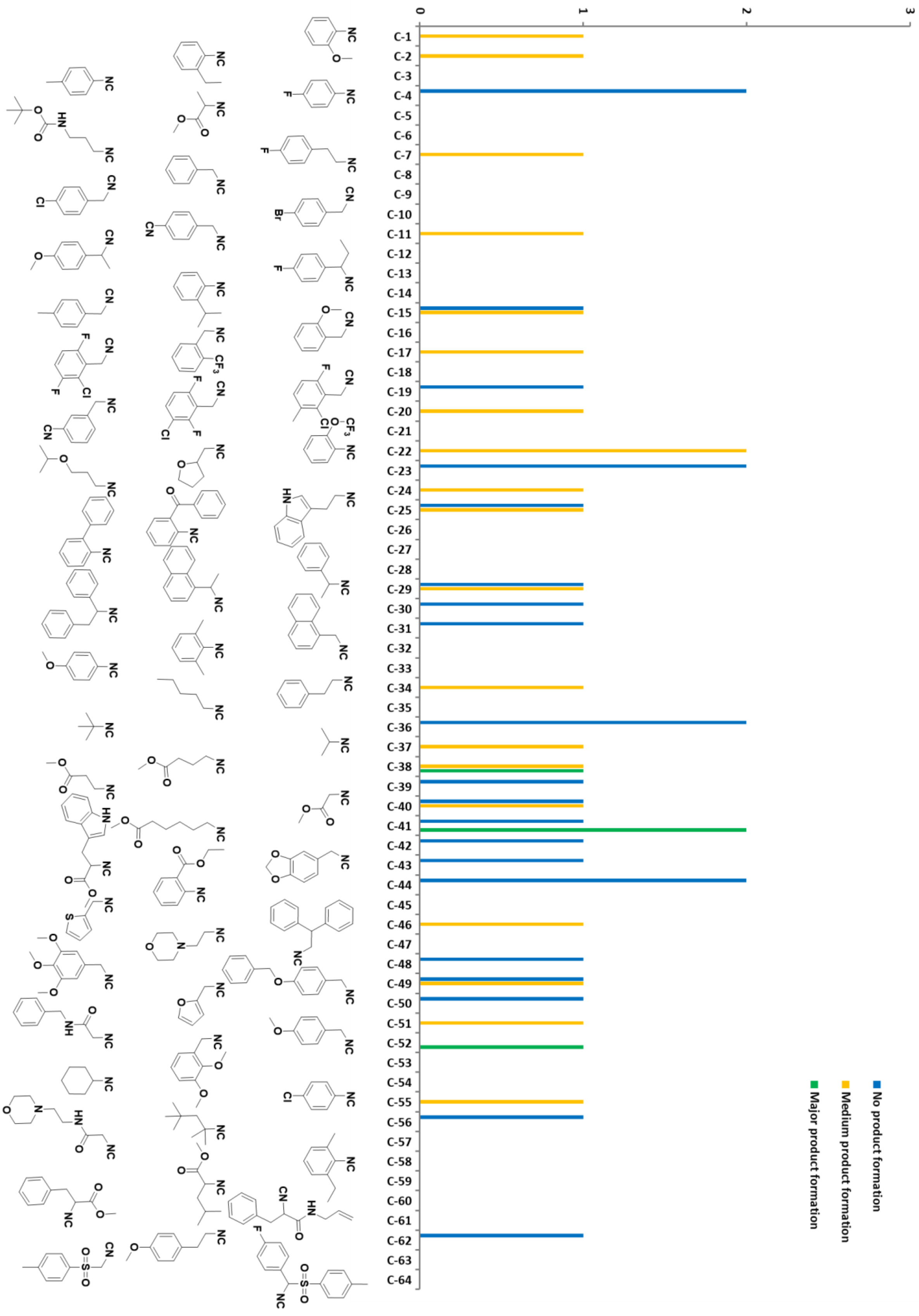

Scheme S9. Performance of isocyanides in U-4CR in destination plate I, wells I1-J24.

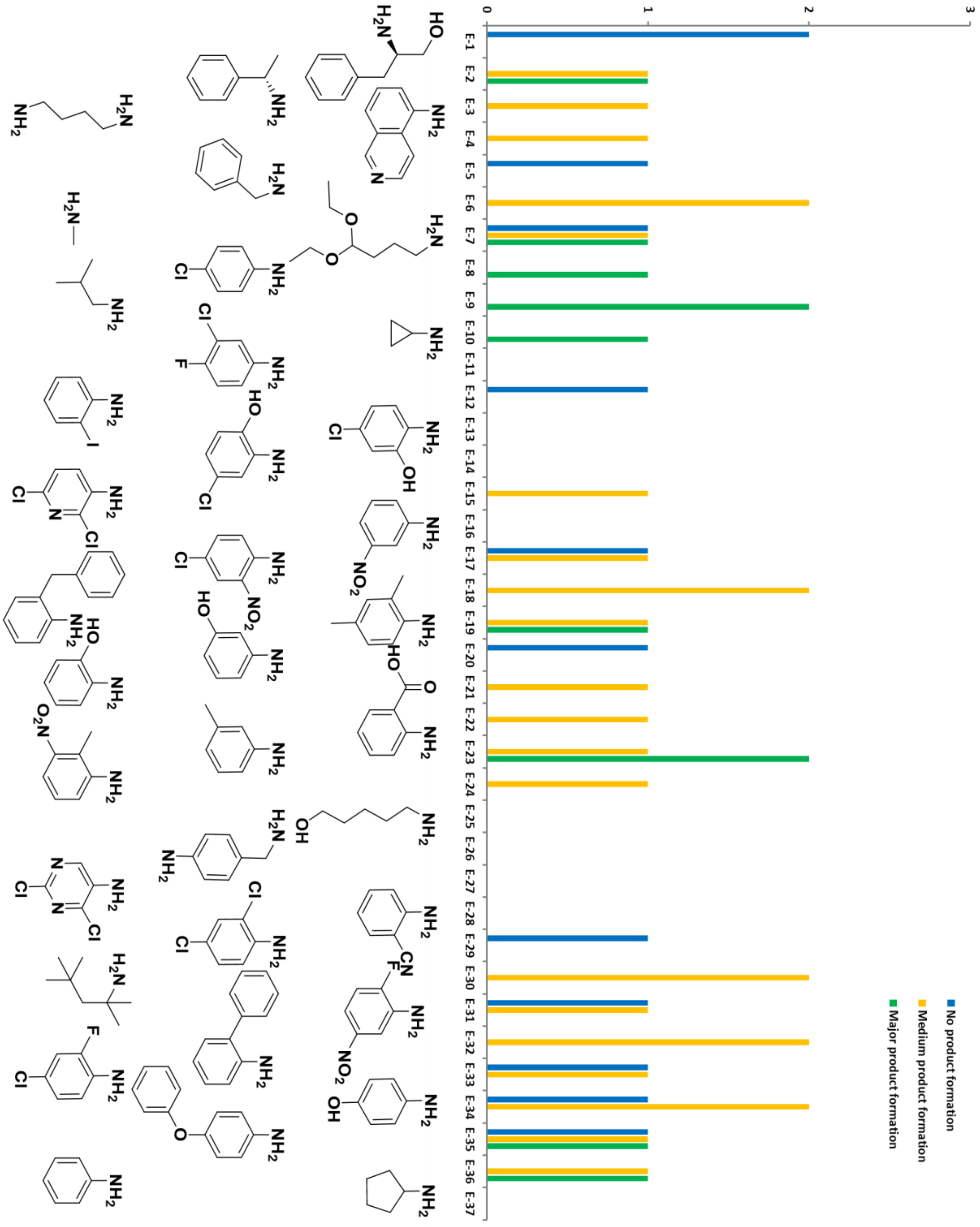

Scheme S10. Performance of amines in U-4CR with formaldehyde in destination plate I, wells K1-L24.

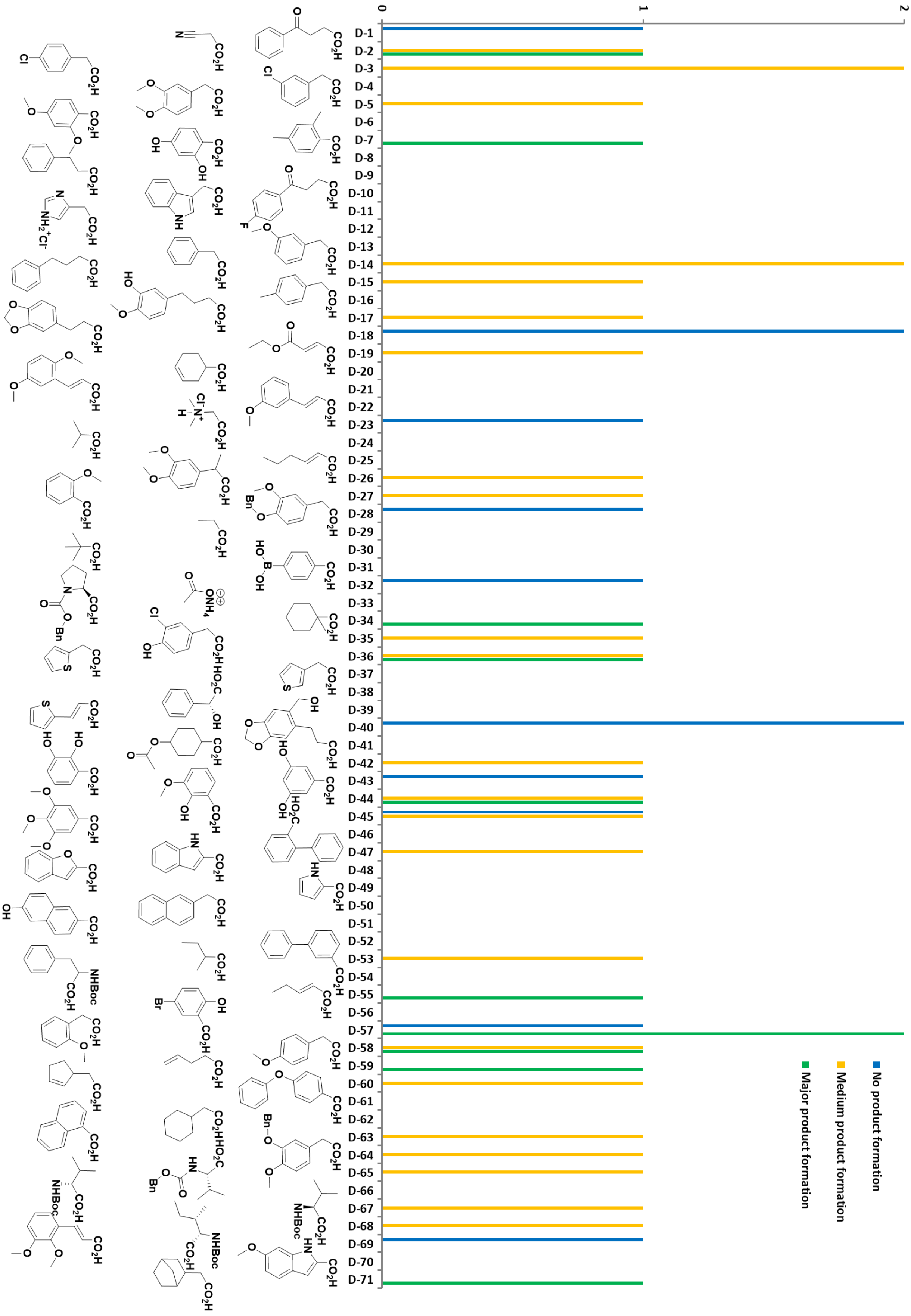

Scheme S11. Performance of carboxylic acids in U-4CR with formaldehyde in destination plate I, wells K1-L24.

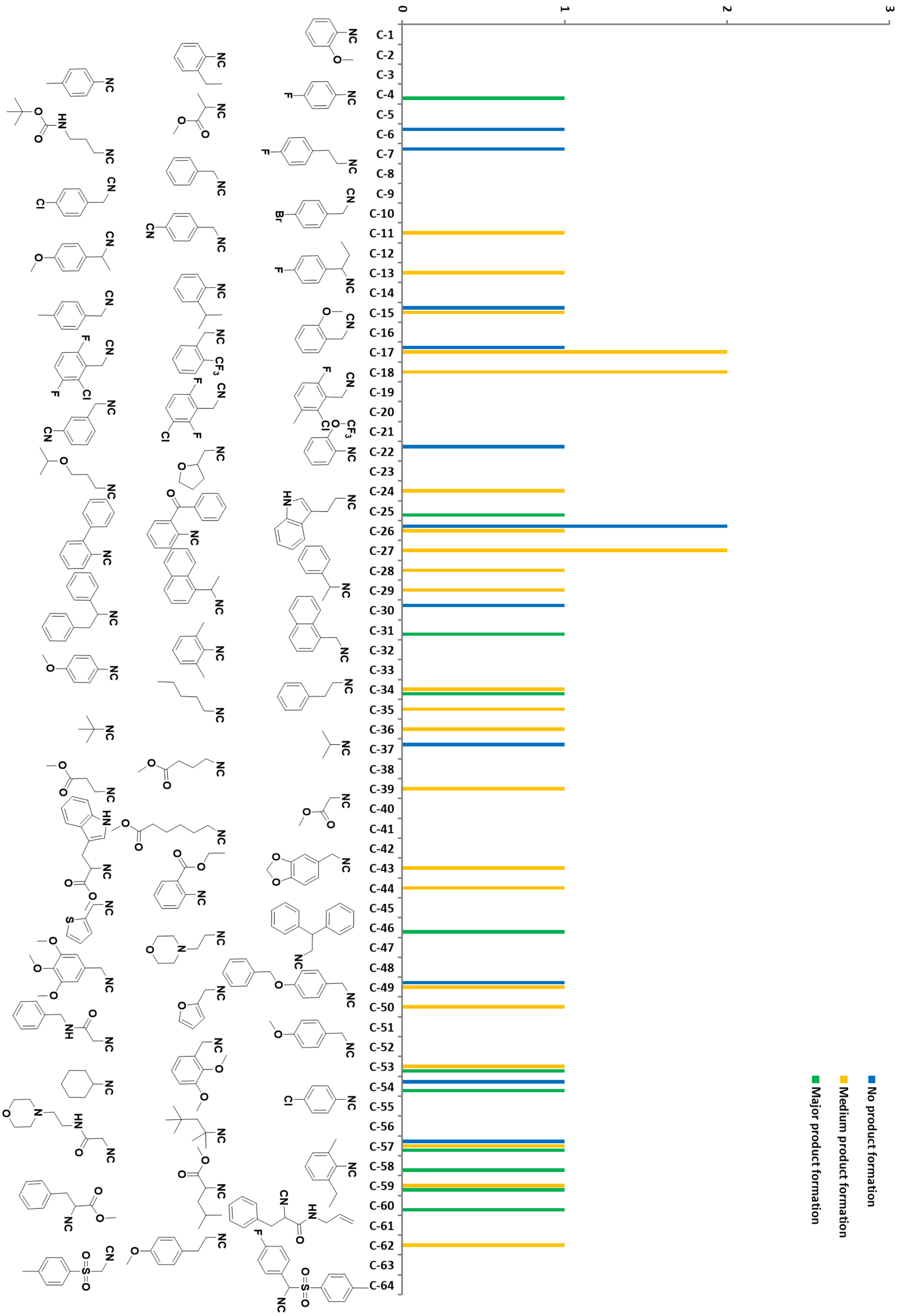

Scheme S12. Performance of isocyanides in U-4CR with formaldehyde in destination plate I, wells K1-L24.

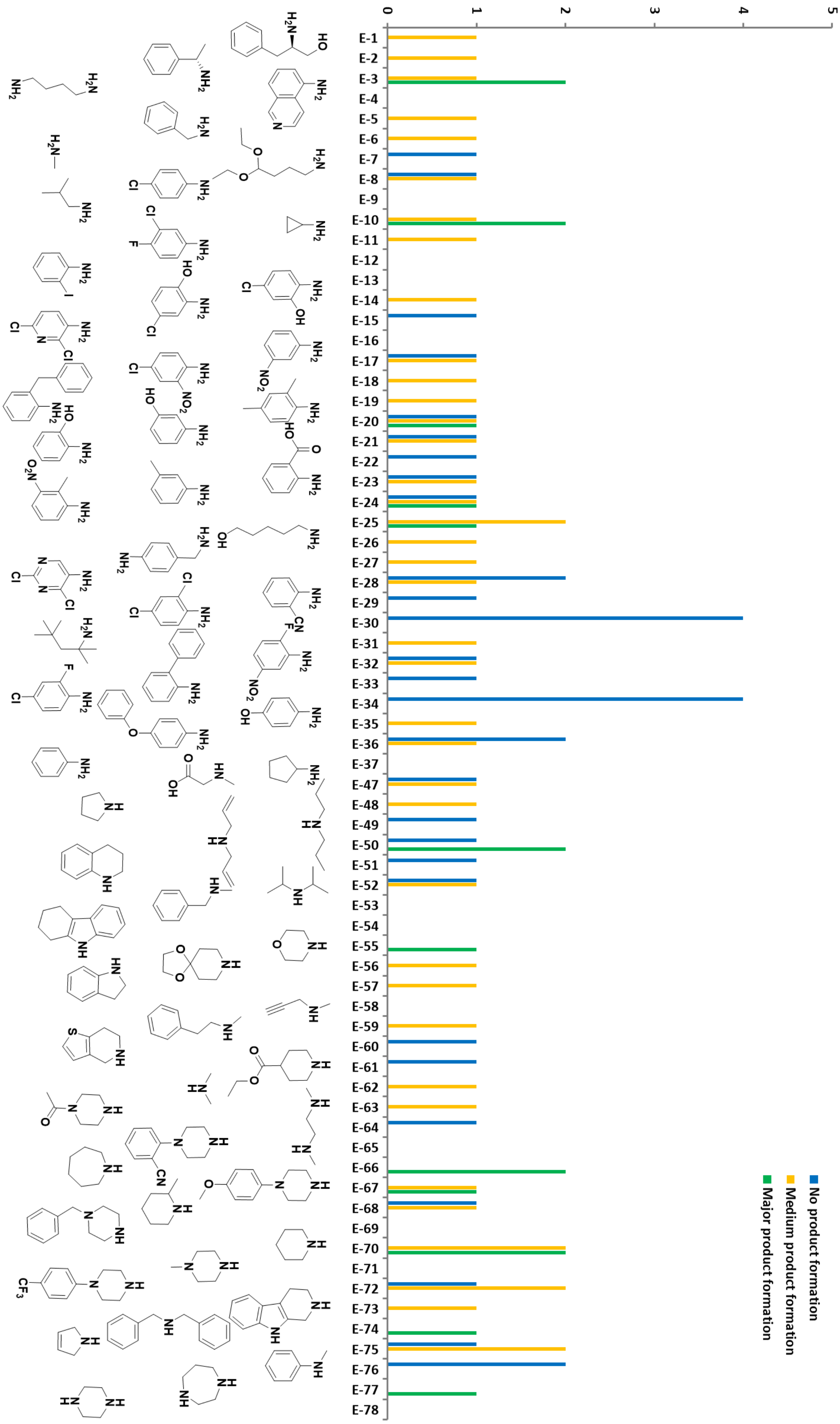

Scheme S13. Performance of amines in UT-4CR in destination plate I.

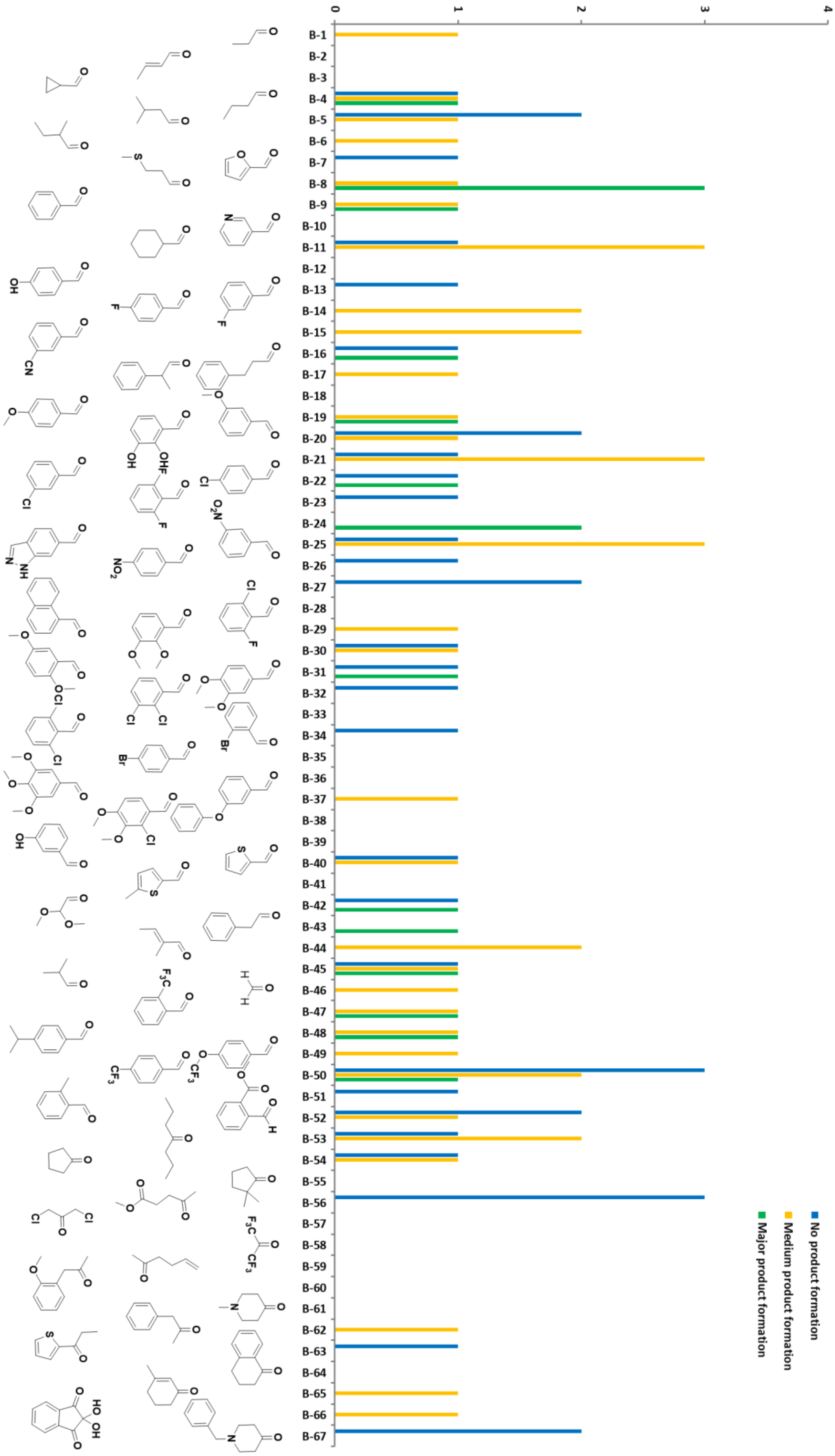

Scheme S14. Performance of oxo components in UT-4CR in destination plate I.

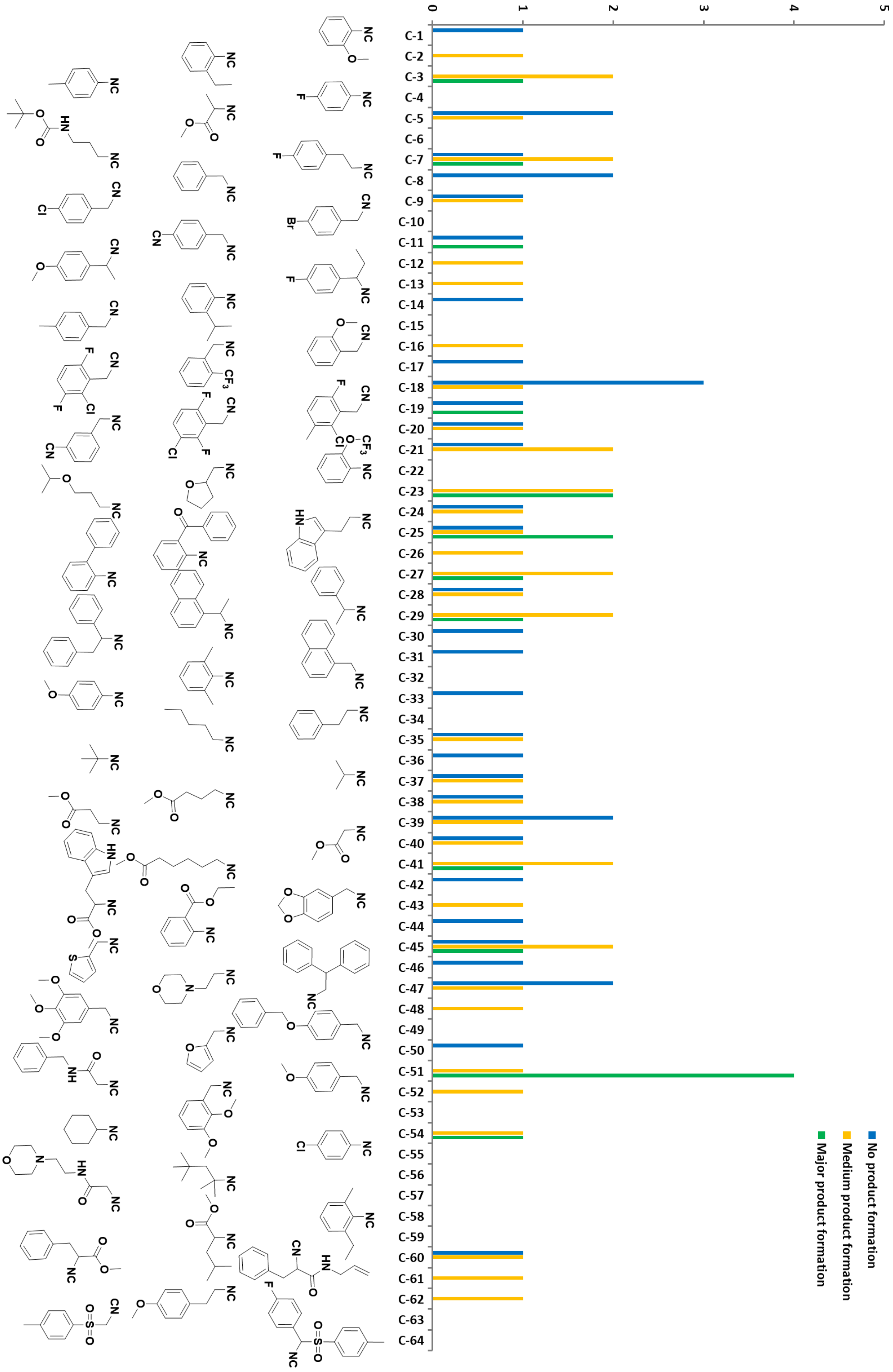

Scheme S15. Performance of isocyanides in UT-4CR in destination plate I.

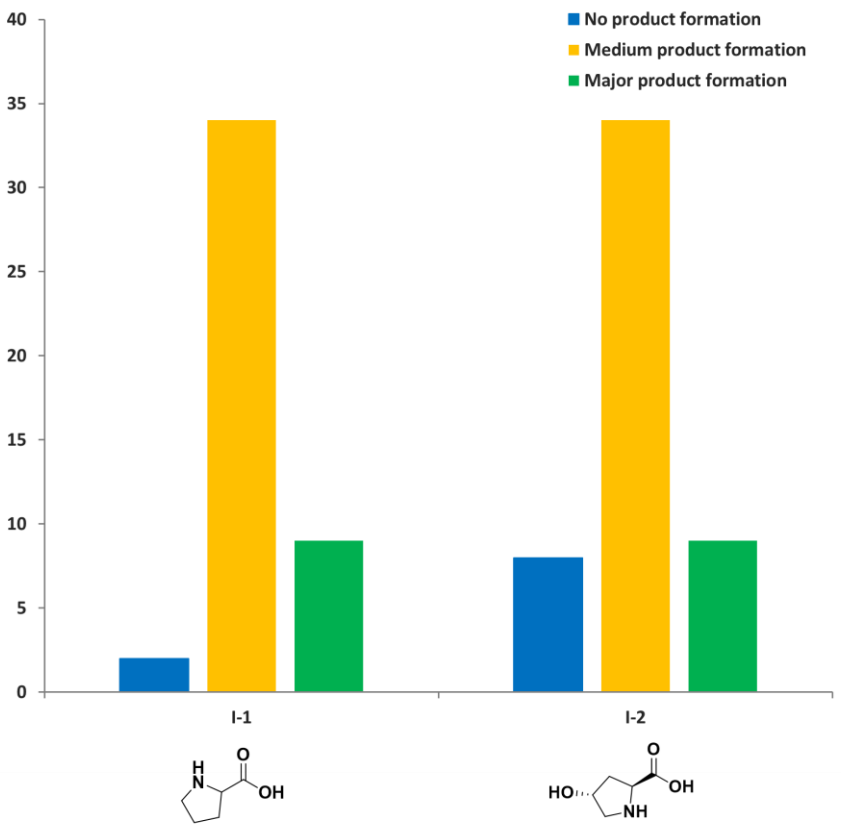

Scheme S16. Performance of prolines in U-5C-3CR in destination plate II.

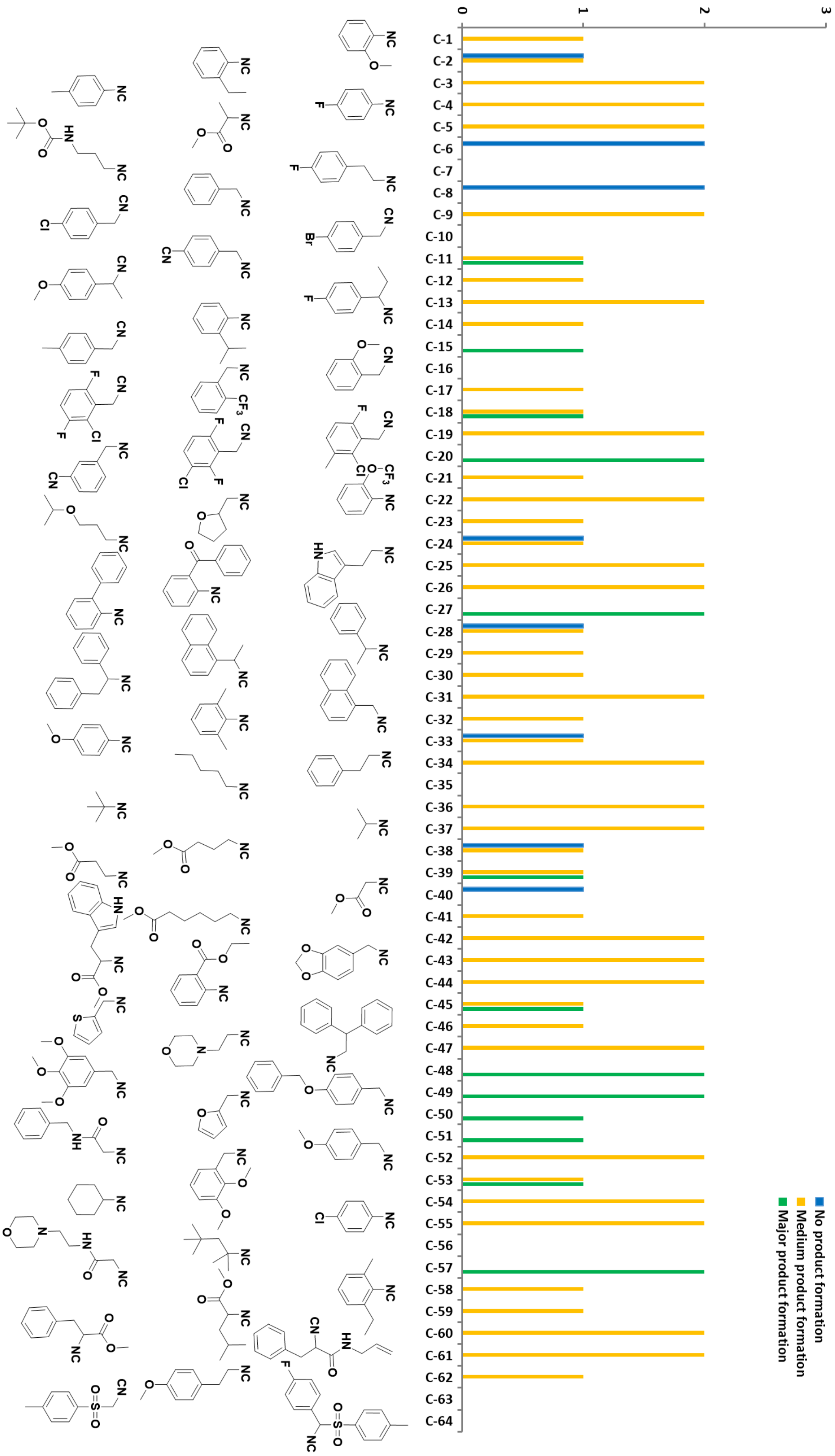

Scheme S17. Performance of isocyanides in U-5C-3CR with prolines in destination plate II.

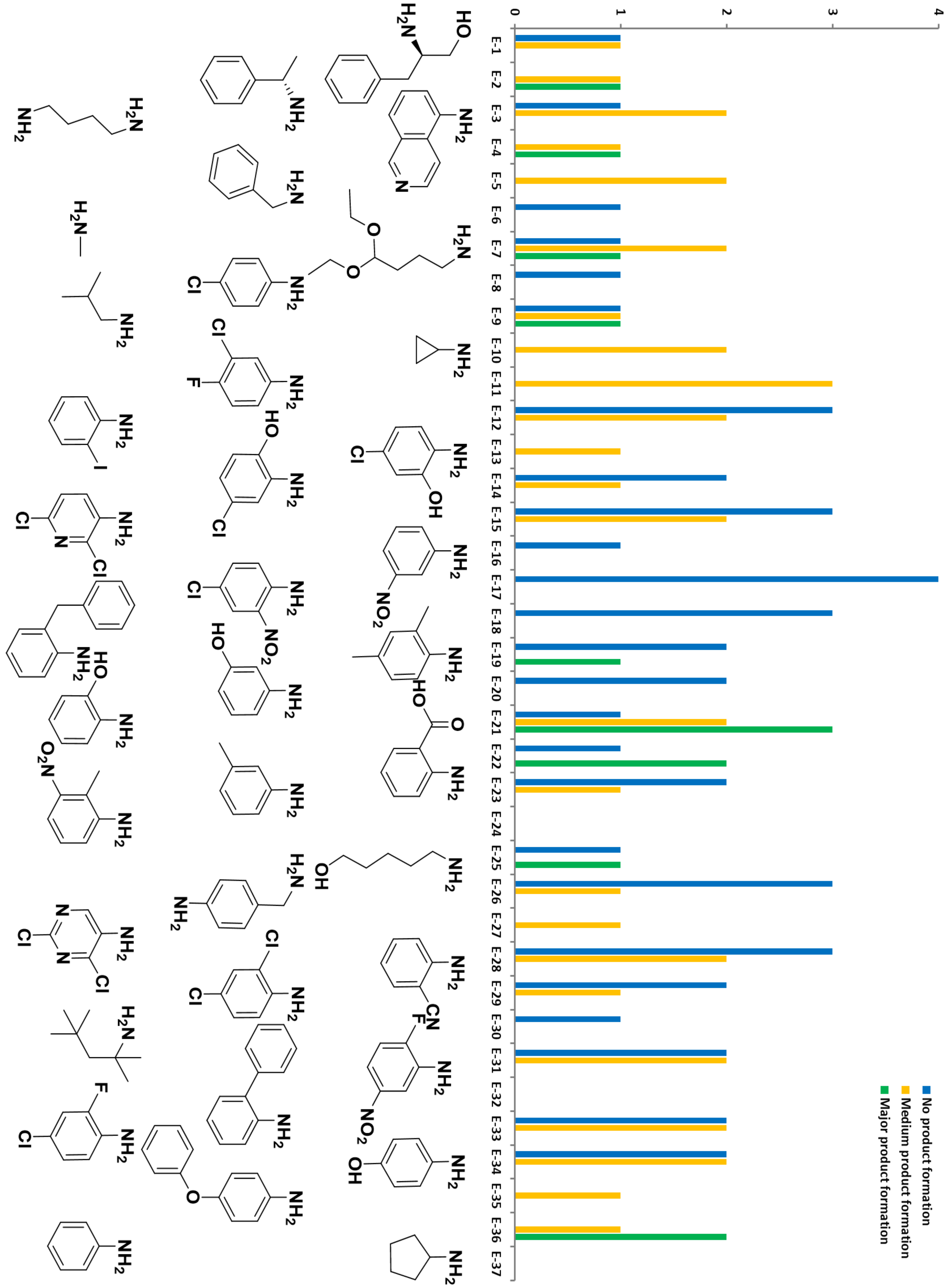

Scheme S18. Performance of primary amines in UH-4CR in destination plate II.

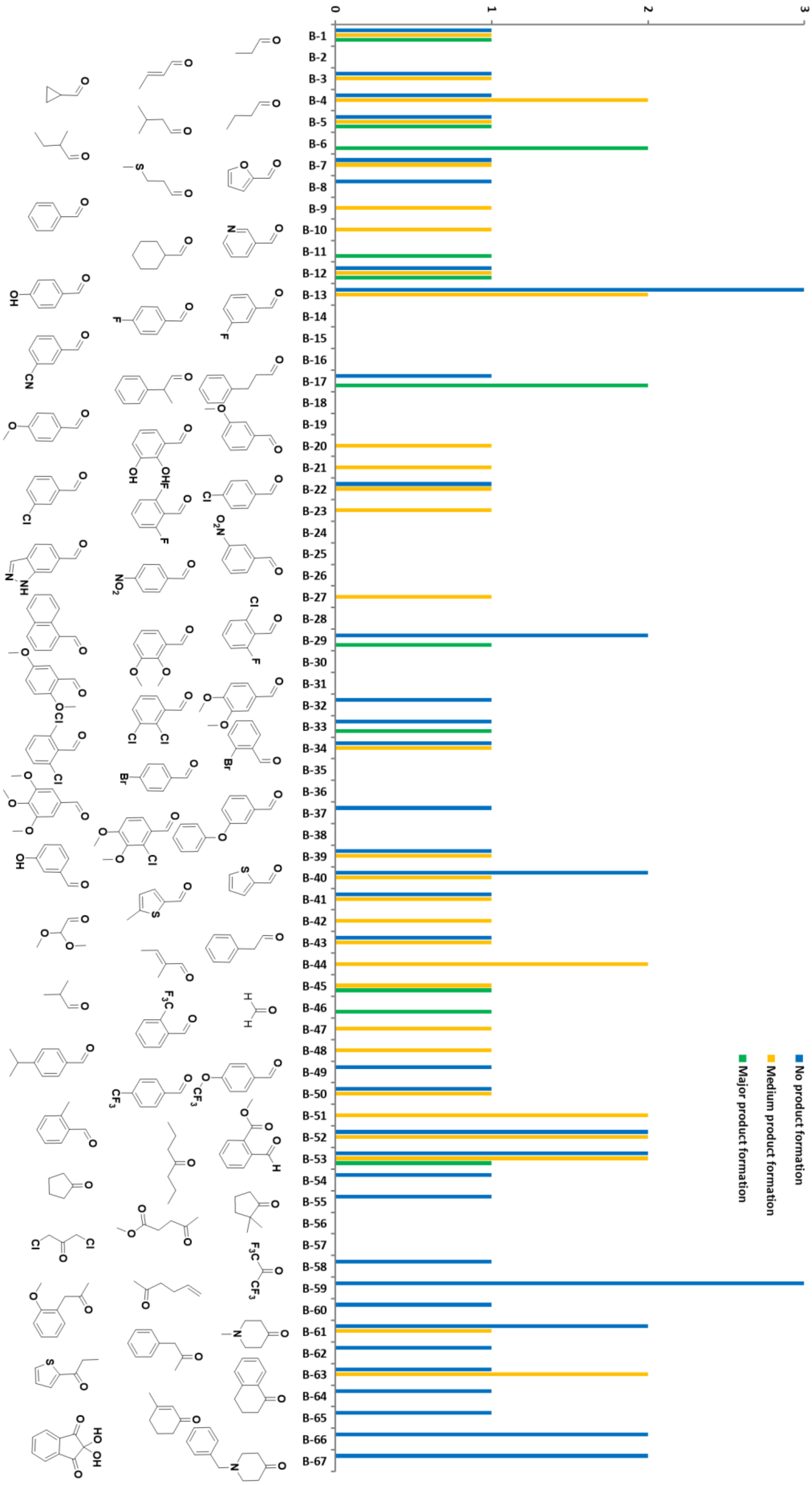

Scheme S19. Performance of oxo components in UH-4CR in destination plate II.

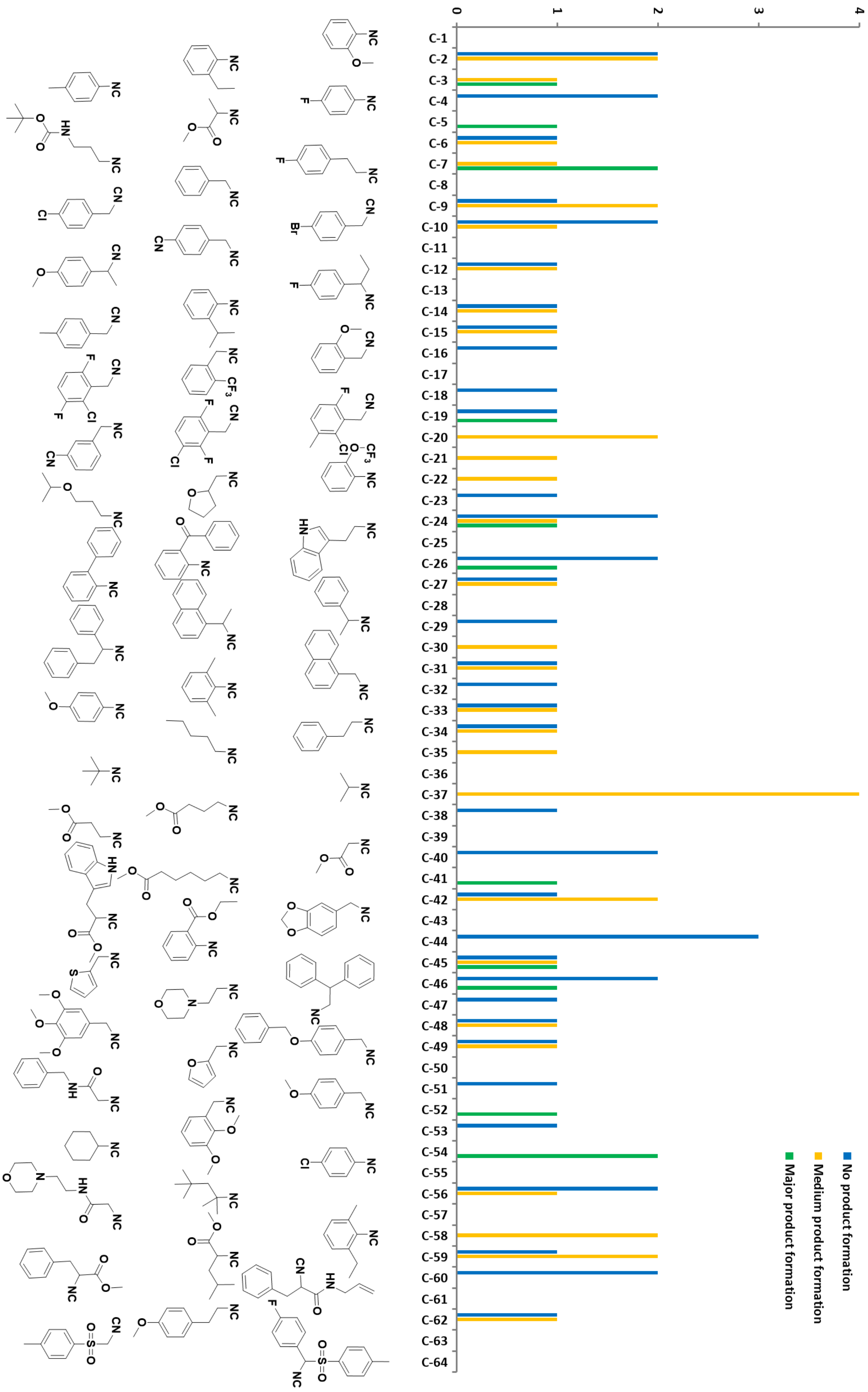

Scheme S20. Performance of isocyanides in UH-4CR in destination plate II.

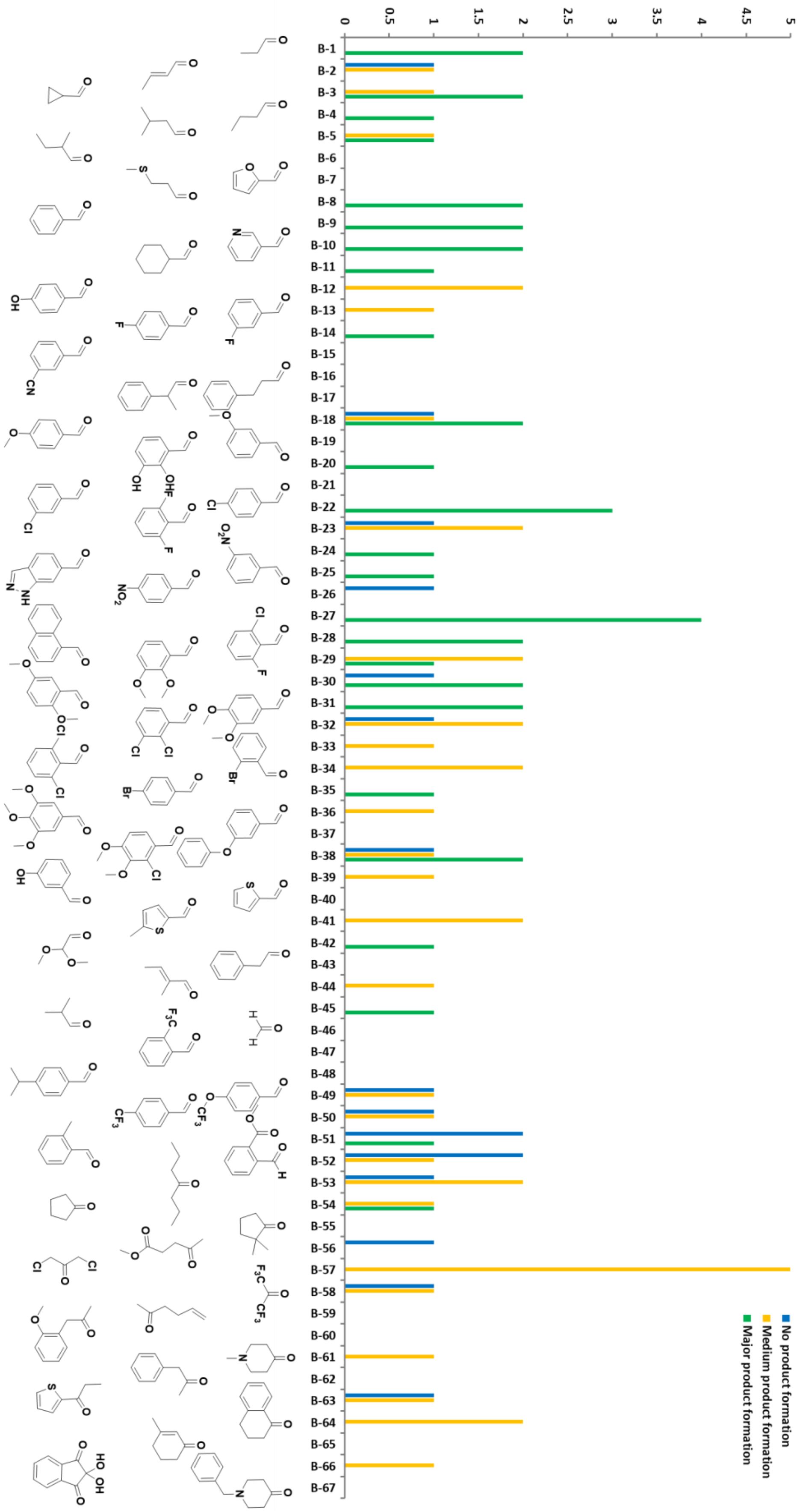

Scheme S21. Performance of oxo components in U-5C-3CR with α-amino acids with nucleophilic side chains in destination plate II.

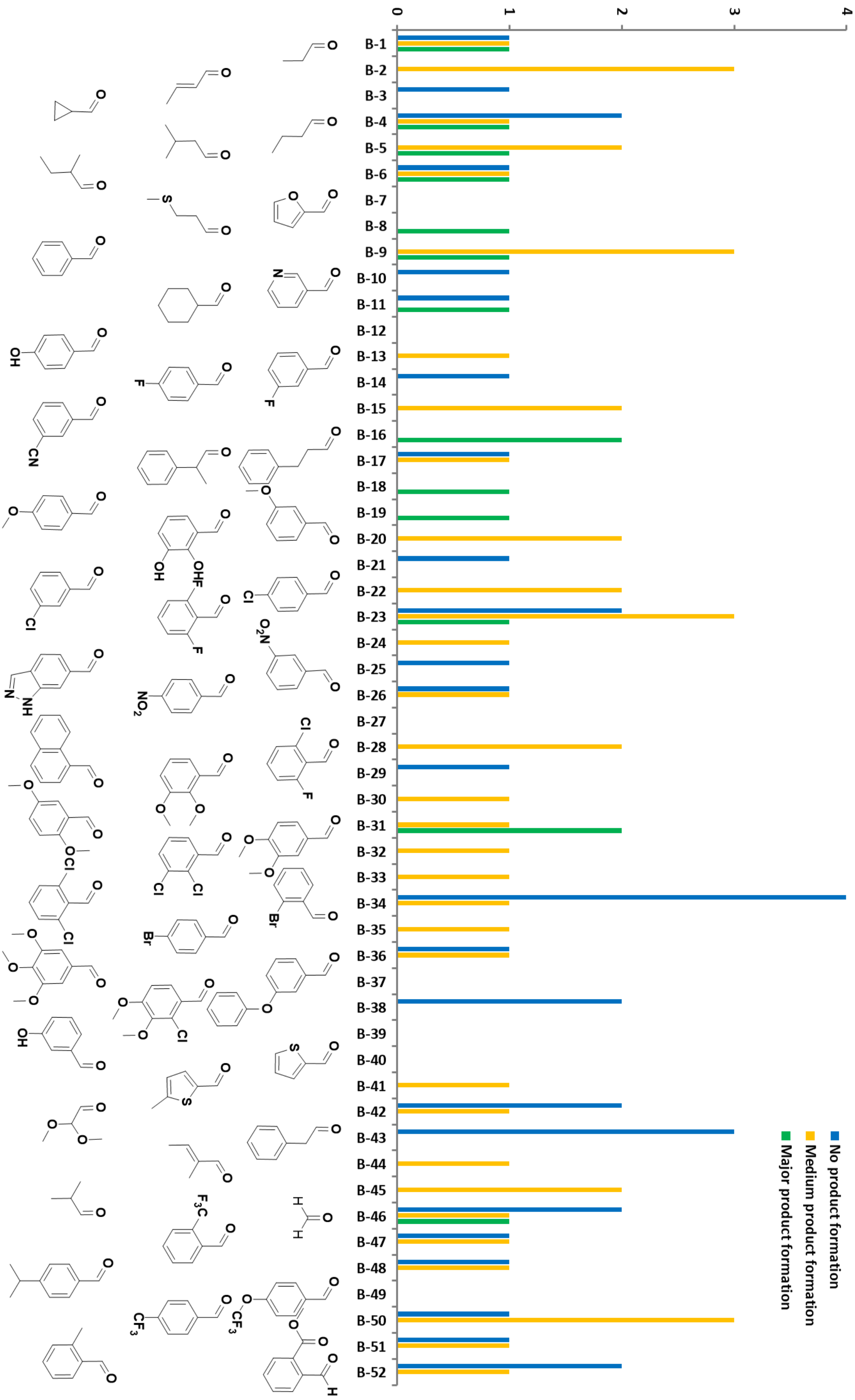

Scheme S22. Performance of aldehydes in vL-3CR in destination plate III.

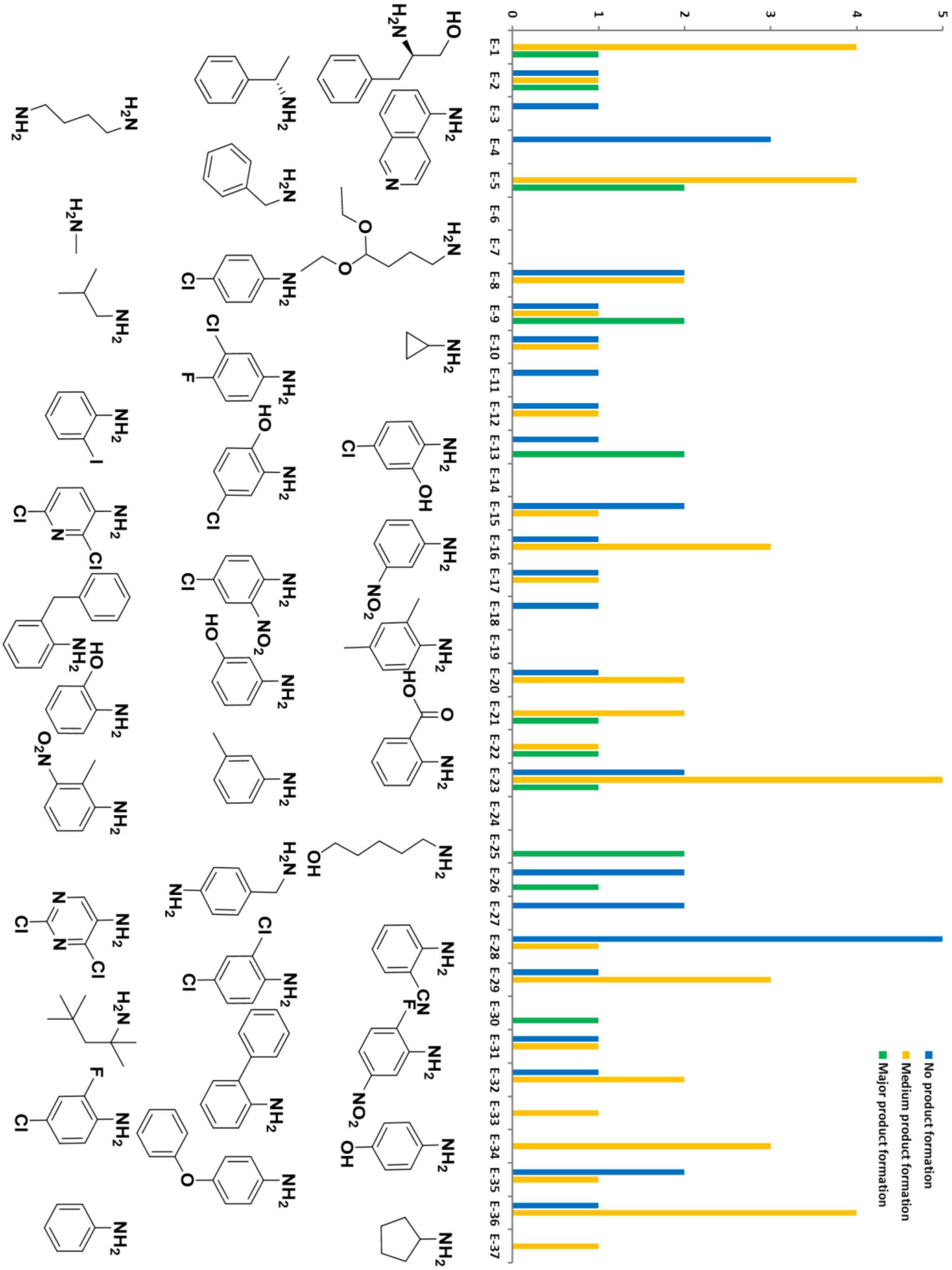

Scheme S23. Performance of amines in vL-3CR in destination plate III.

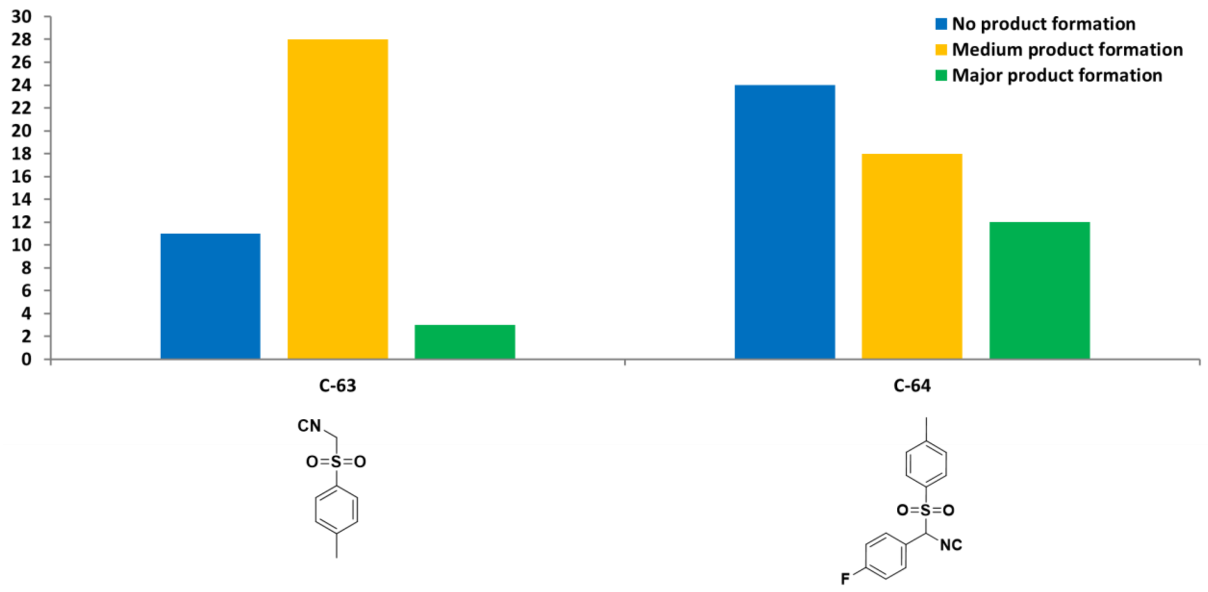

Scheme S24. Performance of tosmic isocyanides in vL-3CR in destination plate III.

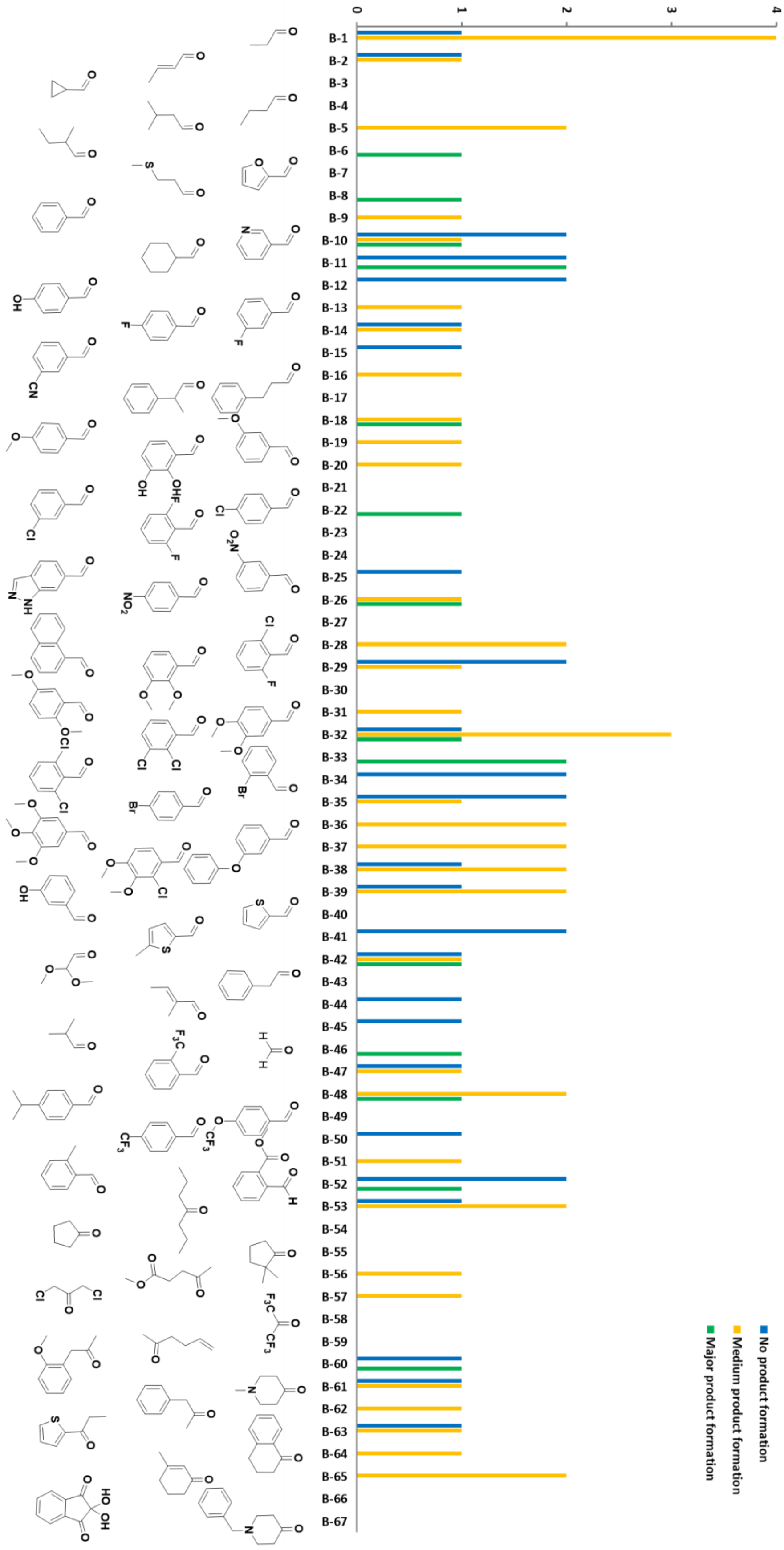

Scheme S25. Performance of oxo components in U-5C-4CR reaction with additional amine in destination plate IV.

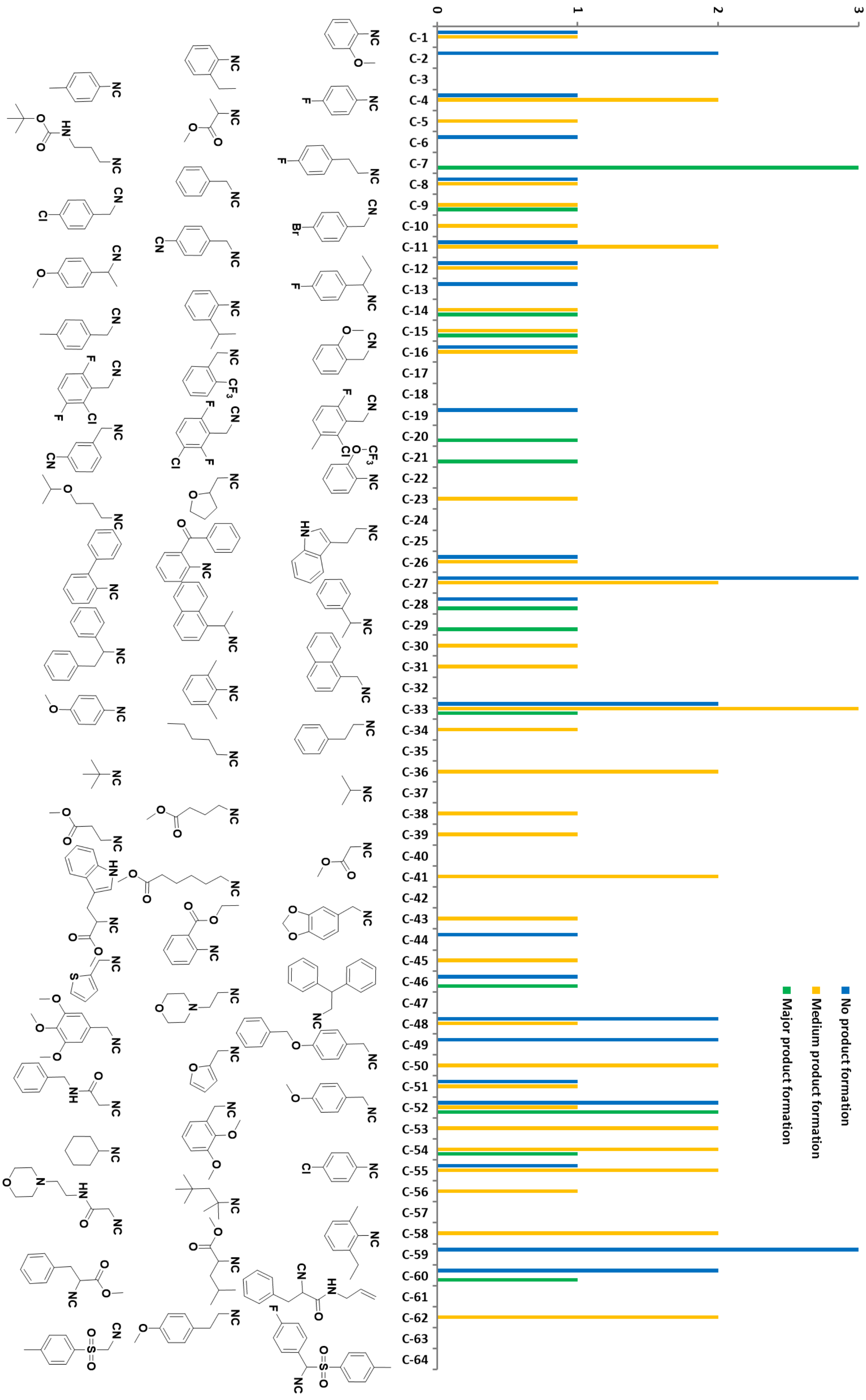

Scheme S26. Performance of isocyanides in U-5C-4CR reaction with additional amine in destination plate IV.

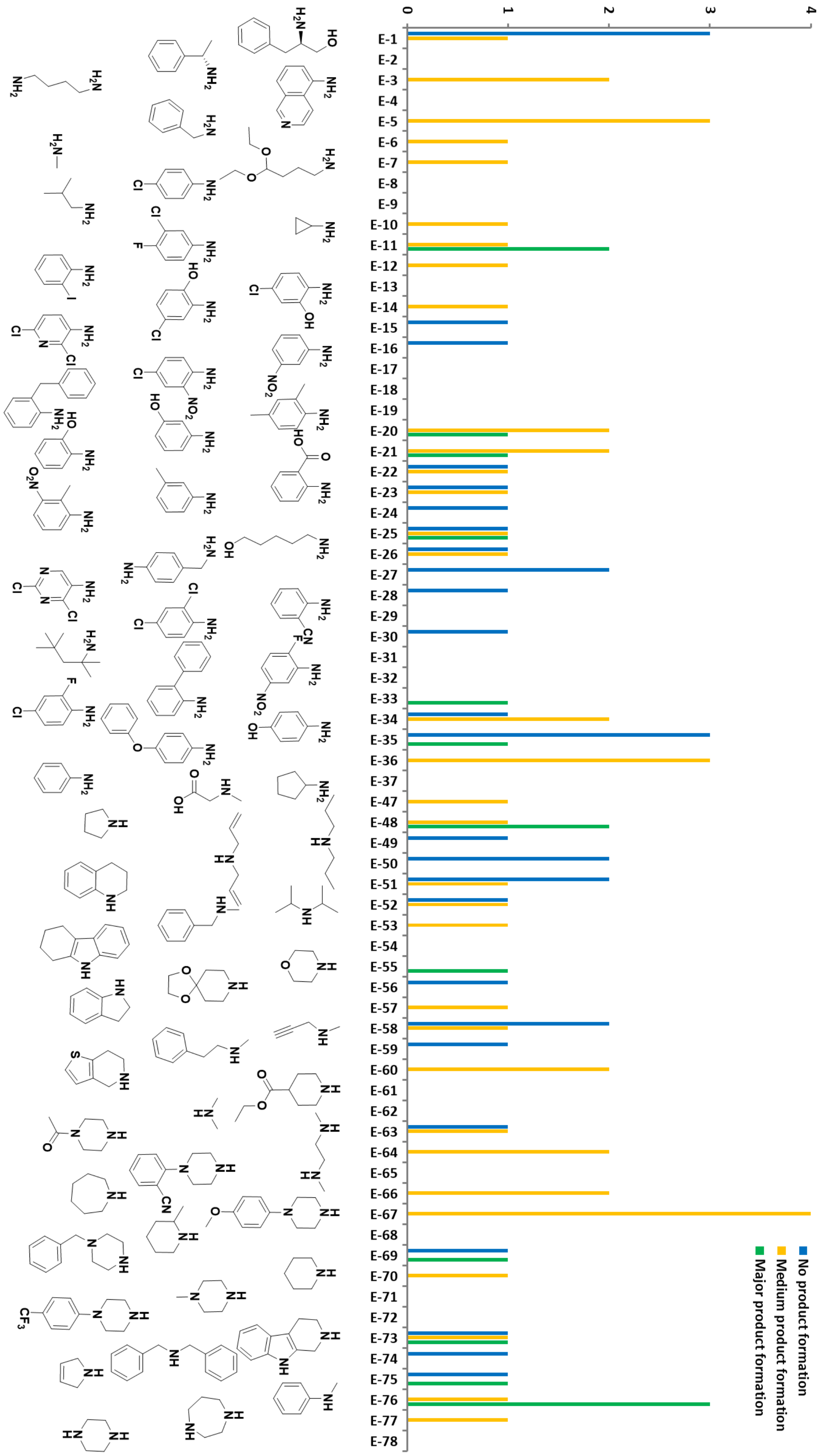

Scheme S27. Performance of amines in U-5C-4CR reaction with additional amine in destination plate IV.

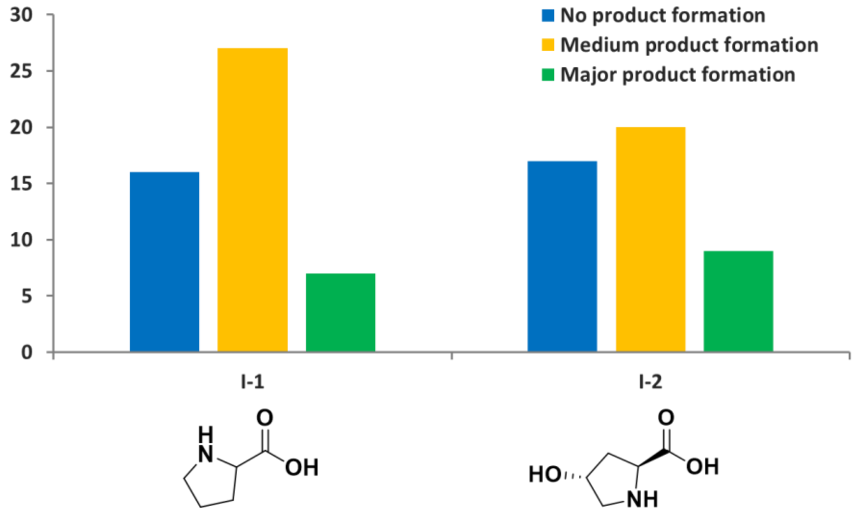

Scheme S28. Performance of prolines in U-5C-4CR reaction with additional amine in destination plate IV.

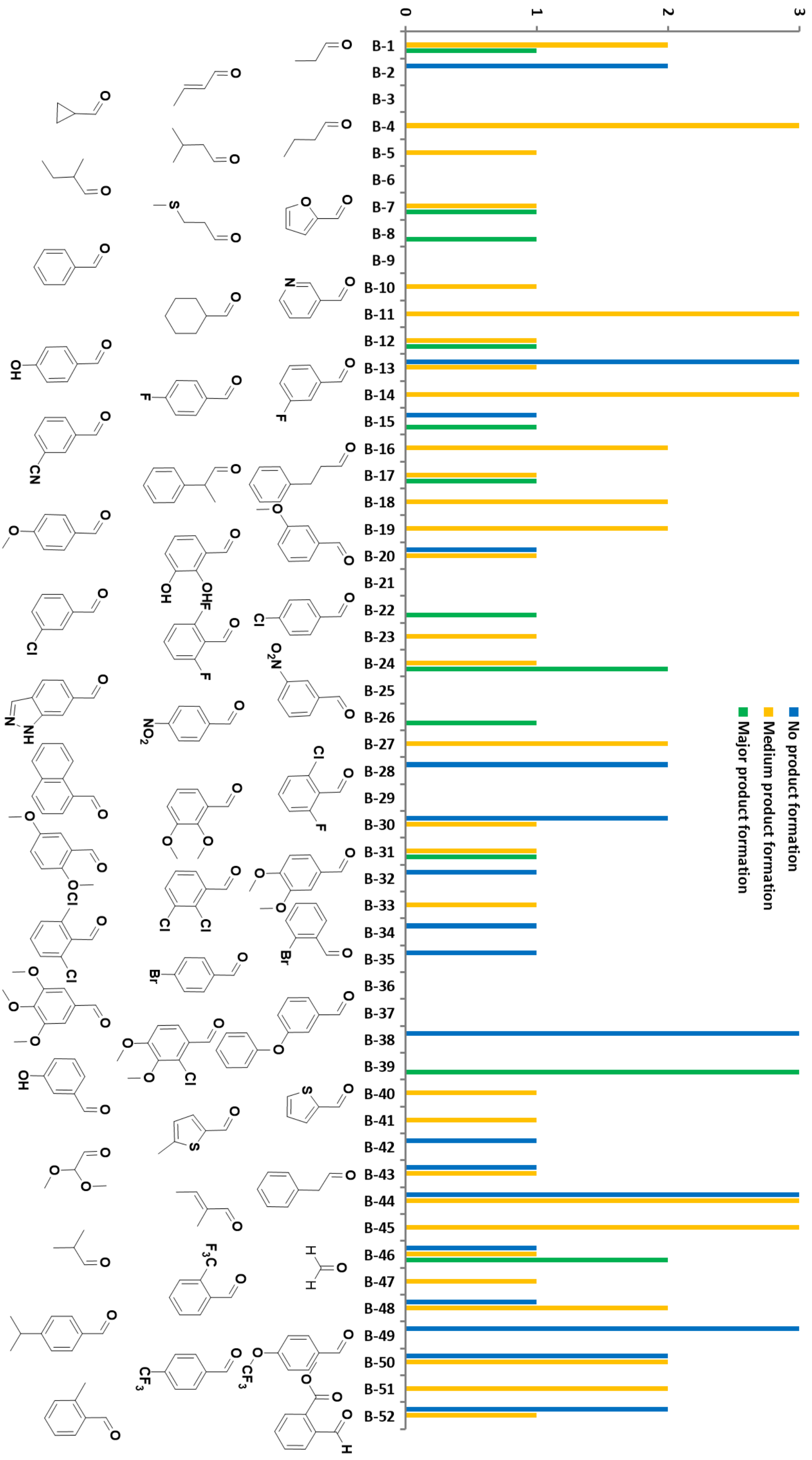

Scheme S29. Performance of aldehydes in UPS-4CR in destination plate IV.

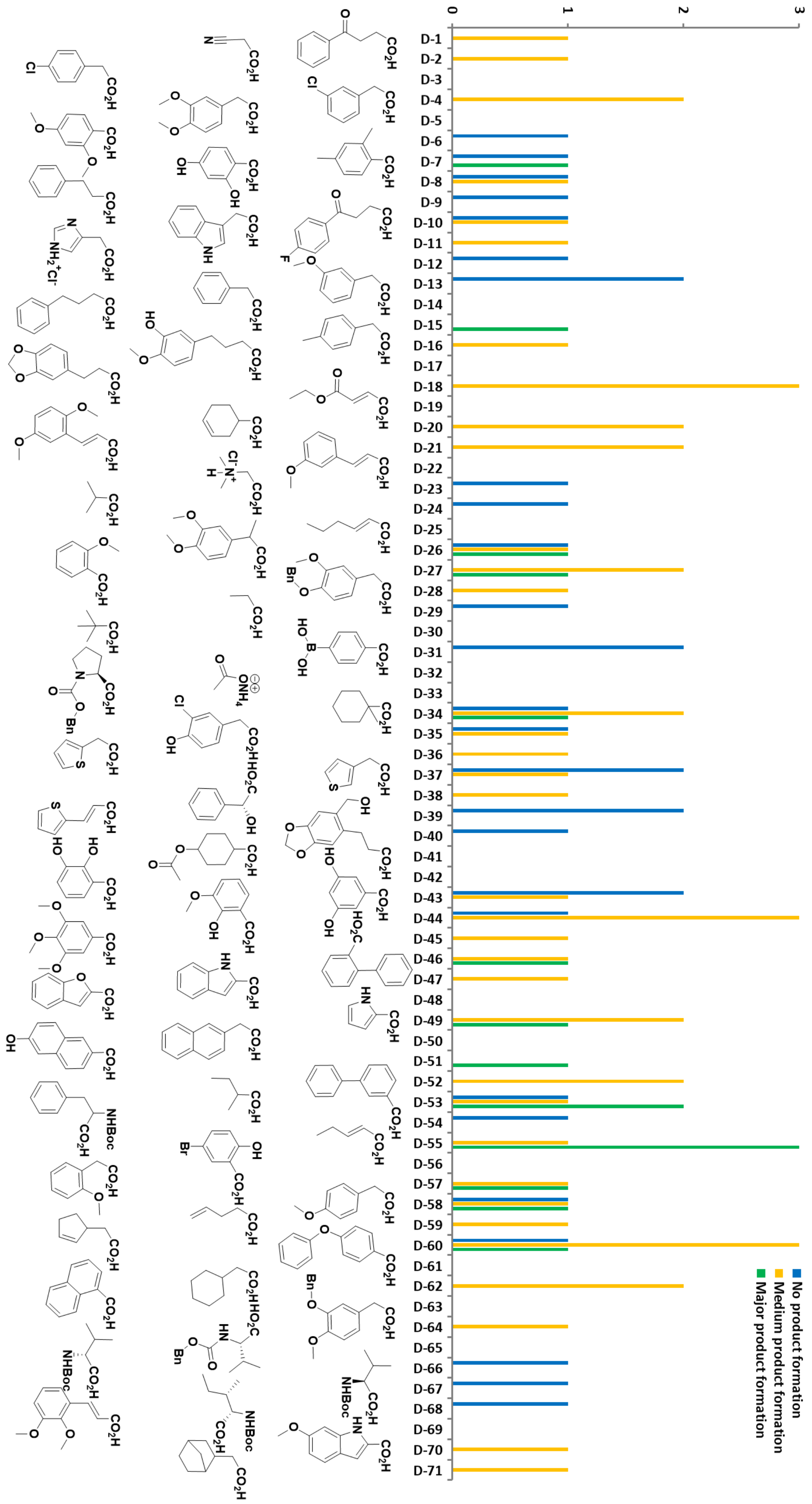

Scheme S30. Performance of carboxylic acids in UPS-4CR in destination plate IV.

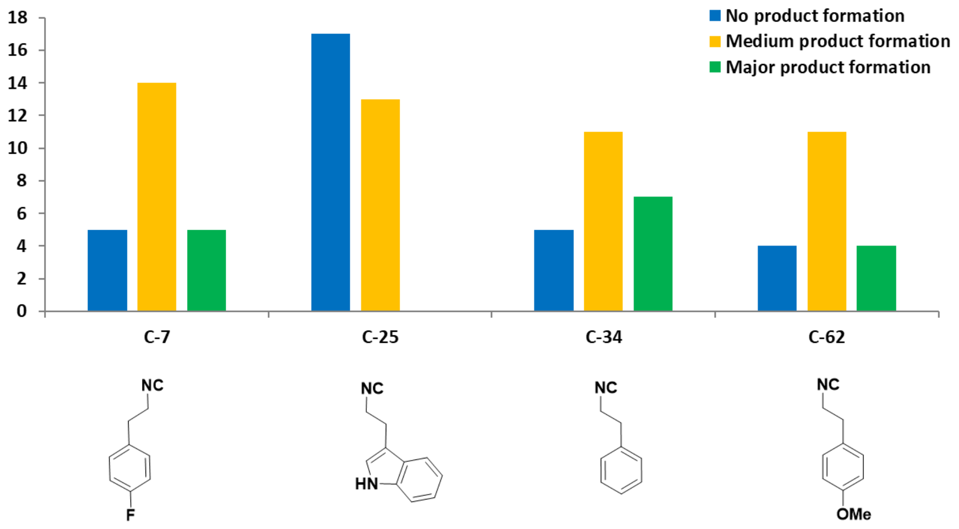

Scheme S31. Performance of hetero/phenylethyl isocyanide in UPS-4CR in destination plate IV.

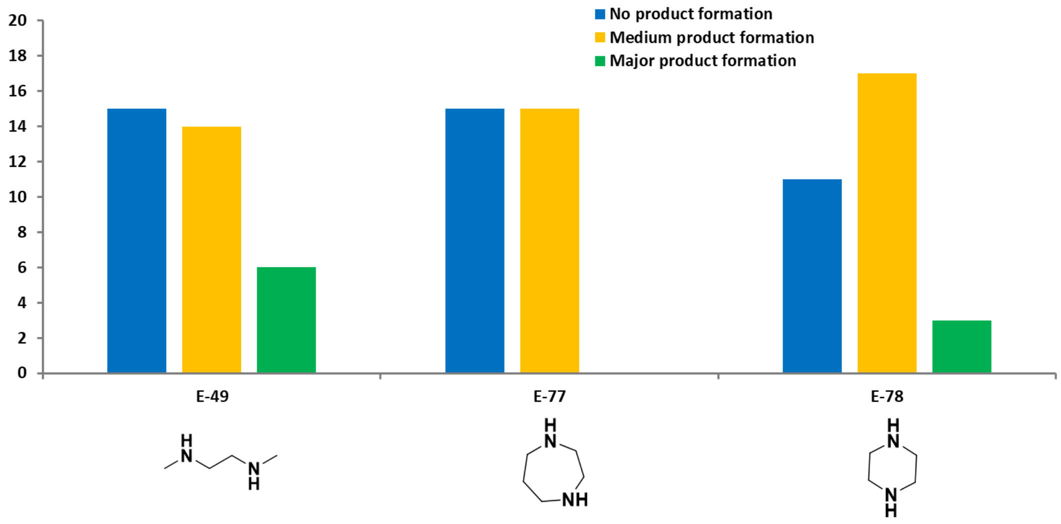

Scheme S32. Performance of bis-secondary amines in SU-4CR in destination plate IV.

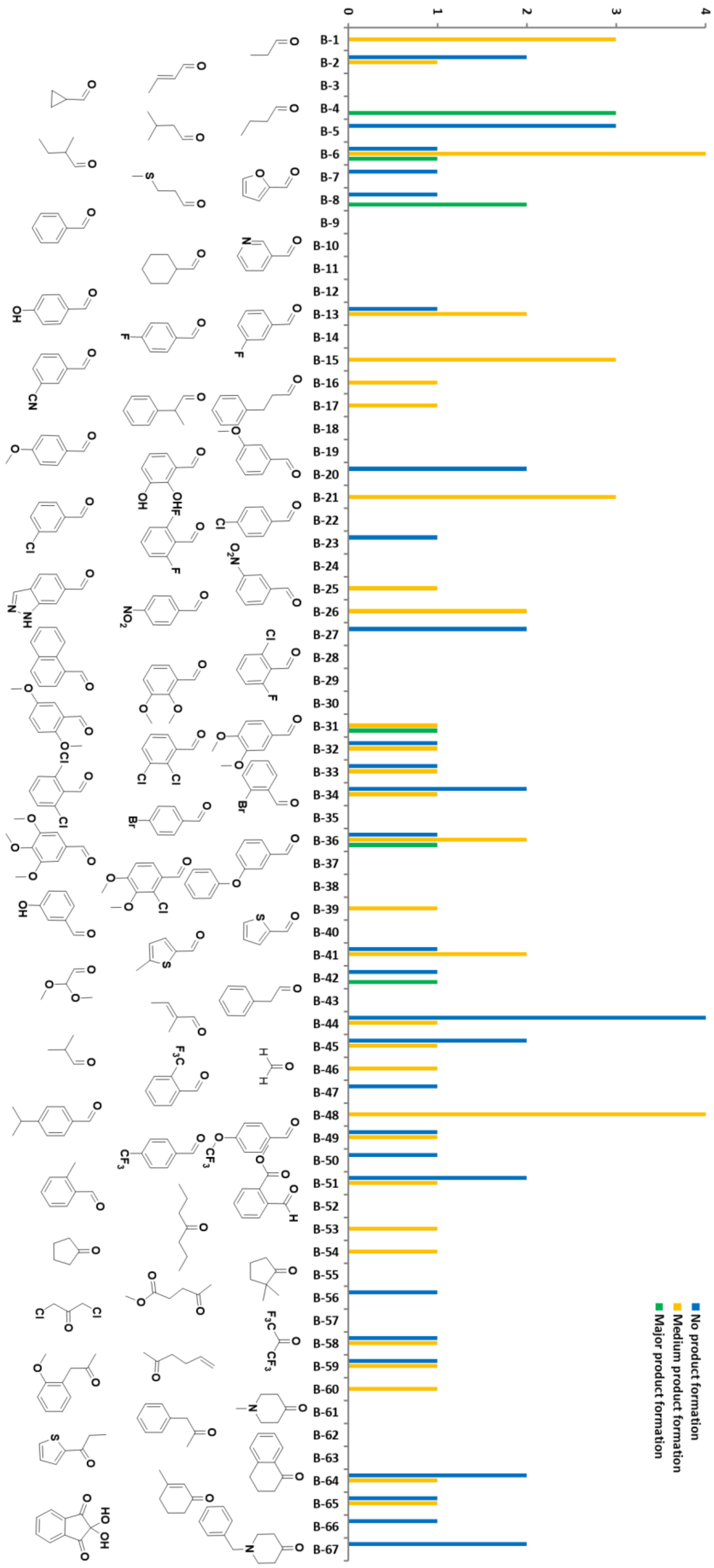

Scheme S33. Performance of oxo components in SU-4CR in destination plate IV.

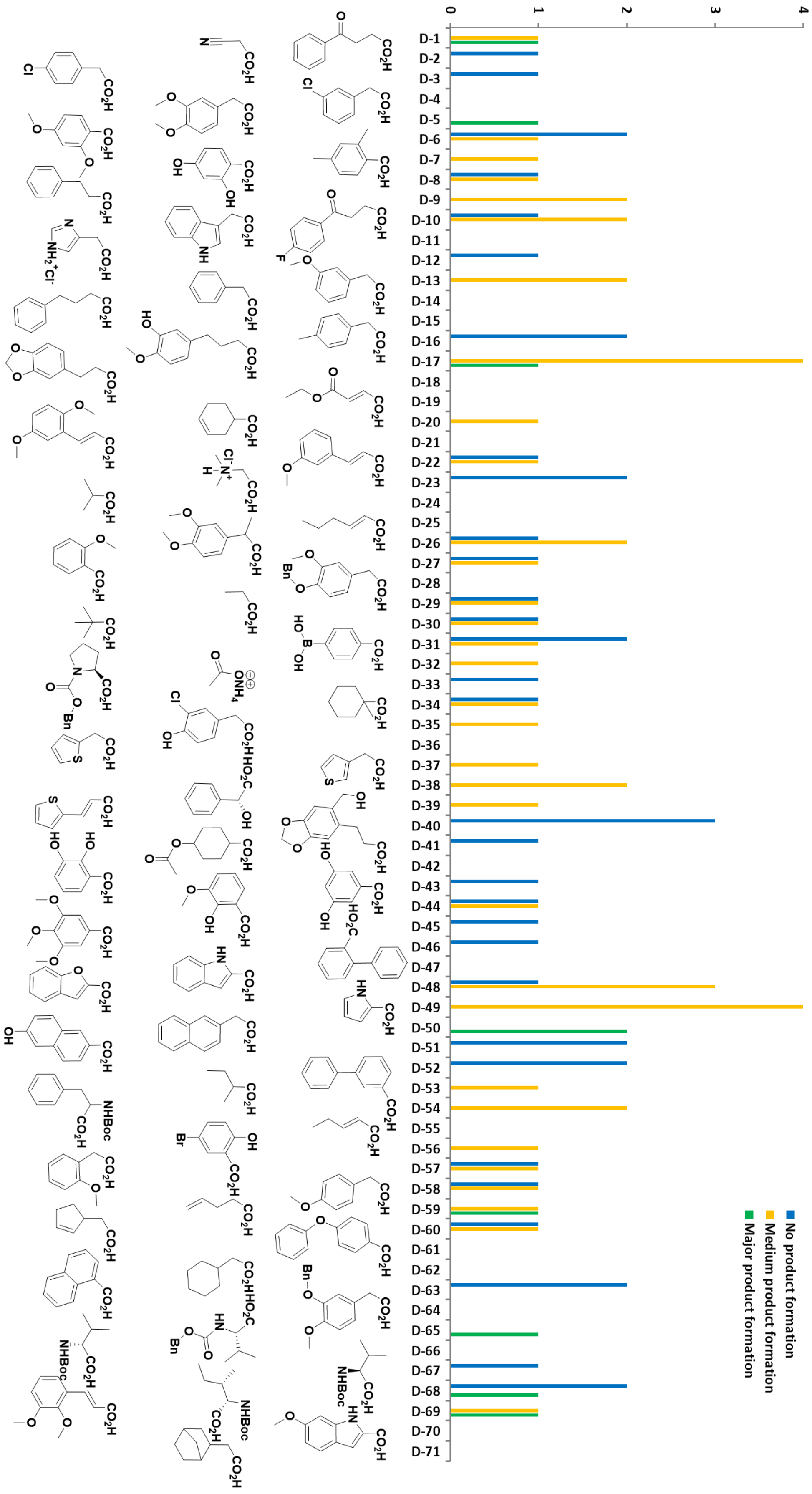

Scheme S34. Performance of carboxylic acids in SU-4CR in destination plate IV.

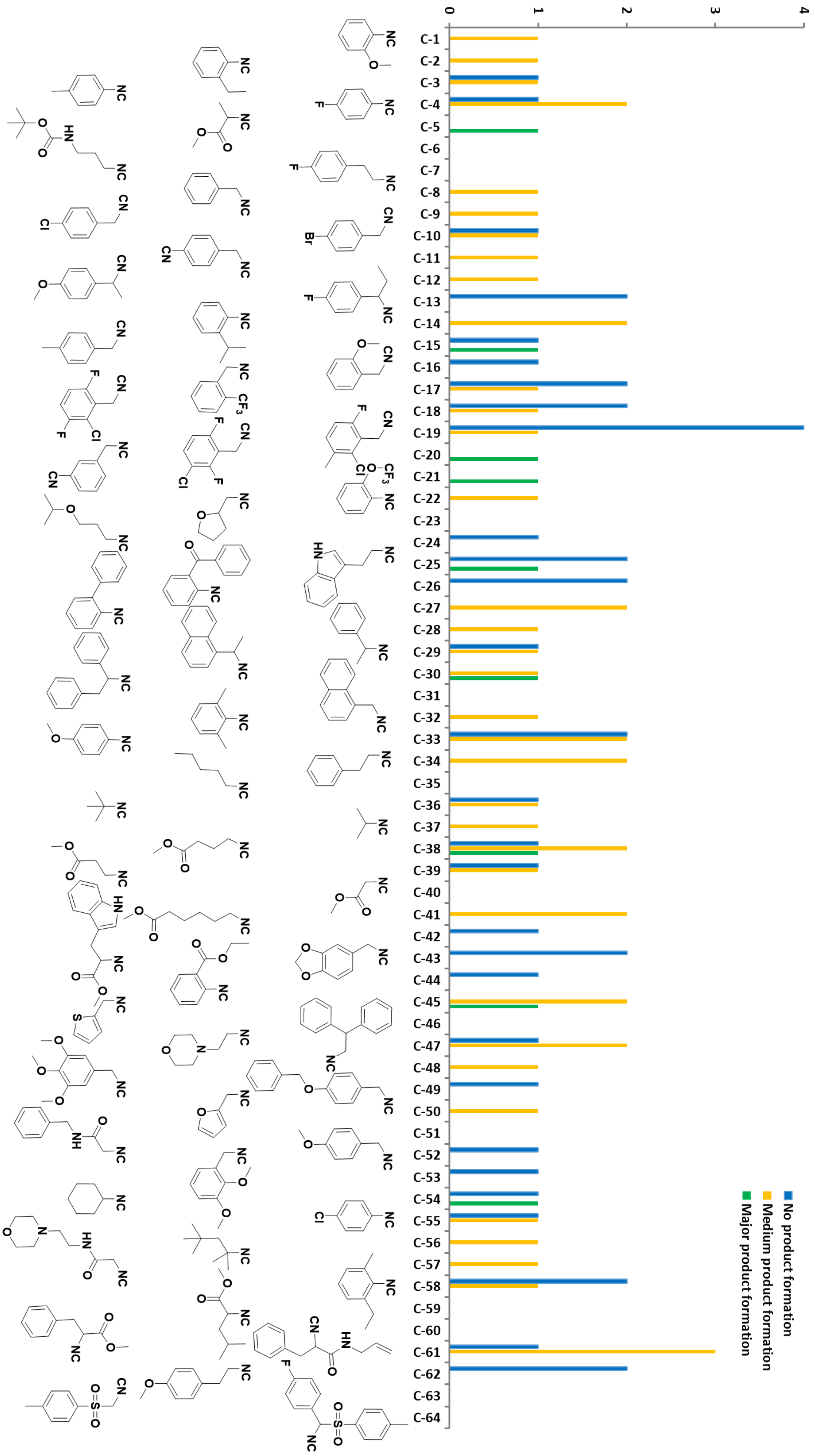

Scheme S35. Performance of isocyanides in SU-4CR in destination plate IV.

4. Milligram scale reactions

4.1. Synthetic procedures and analytical data

4.1.1. GBB-3CR

To a stirred solution of amidine (1 mmol) in MeOH (2 mL) was added aldehyde (1 mmol), isocyanide (1 mmol) and $Sc(OTf)_3$ (10 mol%). The reaction mixture was heated under microwave at 120 °C for 0.5 h. Afterwards, the solvent was evaporated and the crude product was purified with column chromatography on silica gel eluted with petroleum ether/ethyl acetate (5:1-2:1) affording the target products.

Methyl (2-(3-hydroxyphenyl)-8-methylimidazo[1,2-a]pyridin-3-yl)alaninate (Destination plate I, A22)

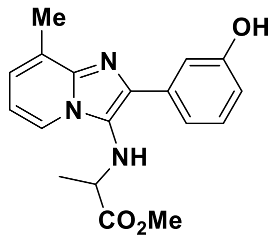

Yellow solid, 280mg, 86% yield; ^1H NMR (500 MHz, $CDCl_3$) δ 8.07 (d, J = 6.8 Hz, 1H), 7.73 (s, 1H), 7.30 – 7.26 (m, 1H), 7.20 (t, J = 7.8 Hz, 1H), 6.98 – 6.94 (m, 1H), 6.79 – 6.69 (m, 2H), 3.90 (d, J = 6.3 Hz, 1H), 3.85 – 3.75 (m, 1H), 3.59 (s, 3H), 2.61 (s, 3H), 1.29 (d, J = 6.9 Hz, 3H), 1.25 (s, 1H); ^{13}C NMR (126 MHz, $CDCl_3$) δ 174.7, 157.3, 141.9, 136.5, 134.5, 129.5, 127.0, 124.1, 123.8, 120.6, 118.7, 115.7, 115.3, 112.2, 55.6, 52.2, 18.8, 16.7; HRMS (ESI$^+$) m/z calculated for $C_{18}H_{20}N_3O_3$ $[M+H]^+$: 326.14992, found $[M+H]^+$: 326.14987.

N-(2,3-dimethoxybenzyl)-2-(3-fluorophenyl)-5-(trifluoromethyl)imidazo[1,2-a]pyridin-3-amine (Destination plate I, B1)

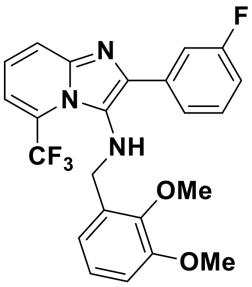

Yellow solid, 170mg, 38% yield; ^1H NMR (500 MHz, $CDCl_3$) δ 7.91 (d, J = 7.8 Hz, 1H), 7.88 – 7.83 (m, 1H), 7.75 (d, J = 8.8 Hz, 1H), 7.42 – 7.34 (m, 1H), 7.32 (d, J = 7.0 Hz, 1H), 7.17 – 7.11 (m, 1H), 7.05 – 6.96 (m, 2H), 6.85 (dd, J = 8.2, 1.5 Hz, 1H), 6.79 (d, J = 7.2 Hz, 1H), 4.04 (d, J = 6.0 Hz, 2H), 3.87 (s, 3H), 3.82 (s, 3H), 3.78 – 3.74 (m, 1H); ^{13}C NMR (126 MHz, $CDCl_3$) δ 162.9 (d, J = 244.4 Hz), 152.4, 147.4, 142.2, 137.7 (d, J = 2.7 Hz), 136.0 (d, J = 8.4 Hz), 132.3, 129.8 (d, J = 8.4 Hz), 128.7, 125.0 (q, J = 36.2 Hz), 123.9, 123.2 (d, J = 2.8 Hz), 122.3, 121.7, 121.3, 120.9 (q, J = 271.5 Hz), 114.7 – 114.2 (m), 111.8, 60.6, 55.6, 48.7 (d, J = 2.4 Hz); HRMS (ESI$^+$) m/z calculated for $C_{23}H_{20}F_4N_3O_2$ $[M+H]^+$: 446.14862, found [M+H]+: 446.14856.

4.1.2. P-3CR

To a stirred solution of aldehyde or ketone (1 mmol) in $CHCl_3$ (2 mL) was added carboxylic acid (1 mmol), and isocyanide (1 mmol). The reaction mixture was stirred at rt for 24 h. Afterwards, the solvent was evaporated and the crude product was purified with column chromatography on silica gel eluted with petroleum ether/ethyl acetate (2:1) affording the target products.

1-(Benzylcarbamoyl)cyclopentyl benzofuran-2-carboxylate (Destination plate I, F16)

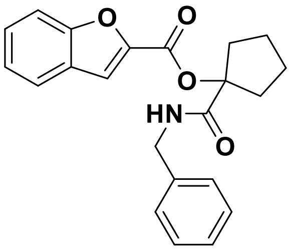

Yellow solid, 69mg, 19% yield; ^1H NMR (500 MHz, $CDCl_3$) δ 7.65 (d, J = 7.9 Hz, 1H), 7.56 – 7.53 (m, 1H), 7.53 – 7.51 (m, 1H), 7.47 – 7.42 (m, 1H), 7.33 – 7.27 (m, 3H), 7.27 – 7.20 (m, 3H), 6.32 (t, J = 5.9 Hz, 1H), 4.49 (d, J = 5.8 Hz, 2H), 2.53 – 2.42 (m, 2H), 2.33 – 2.19 (m, 2H), 1.93 – 1.79 (m, 4H); ^{13}C NMR (126 MHz, $CDCl_3$) δ 171.9, 158.3, 155.7, 145.1, 138.0, 128.6, 127.9, 127.5, 127.4, 126.7, 123.9, 122.8, 114.7, 112.3, 91.6, 43.5, 37.0, 24.8; HRMS (ESI$^+$) m/z calculated for $C_{22}H_{22}NO_4$ $[M+H]^+$: 364.15433,

found [M+H]+: 364.1546.

1-((4-Methoxy-4-oxobutyl)amino)-1-oxo-3-phenylpropan-2-yl 2-hydroxy-3-methoxybenzoate (Destination plate I, G5)

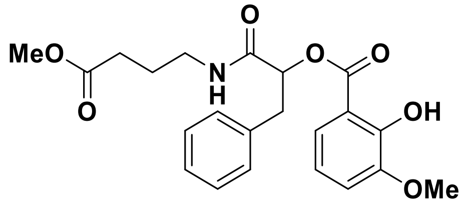

White solid, 228mg, 55% yield; ^{1}H NMR (500 MHz, CDCl$_3$) δ 10.50 (s, 1H), 7.41 (dd, *J* = 8.2, 1.6 Hz, 1H), 7.31 – 7.16 (m, 5H), 6.95 (dd, *J* = 8.2, 1.6 Hz, 1H), 6.92 – 6.82 (m, 1H), 6.82 – 6.73 (m, 1H), 5.58 (t, *J* = 5.8 Hz, 1H), 3.78 (s, 3H), 3.57 (s, 3H), 3.36 – 3.26 (m, 3H), 3.26 – 3.14 (m, 1H), 2.26 – 2.14 (m, 2H), 1.78 – 1.66 (m, 2H); ^{13}C NMR (126 MHz, CDCl$_3$) δ 173.4, 168.6, 168.3, 151.5, 147.9, 135.3, 129.2, 128.0, 126.7, 120.6, 118.4, 116.5, 111.7, 74.8, 55.6, 51.2, 38.4, 37.5, 30.9, 23.8; HRMS (ESI$^+$) m/z calculated for $C_{22}H_{26}NO_7$ [M+H]$^+$: 416.17038, found [M+H]$^+$: 416.17035.

1-([1,1'-Biphenyl]-2-ylamino)-1-oxo-3-phenylpropan-2-yl 2-(3-chloro-4-hydroxyphenyl)acetate (Destination plate I, G6)

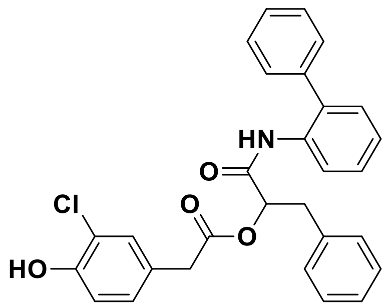

Brown oil, 218mg, 45% yield; ^{1}H NMR (500 MHz, CDCl$_3$) δ 8.39 (d, *J* = 8.2 Hz, 1H), 7.80 (s, 1H), 7.43 – 7.34 (m, 4H), 7.24 – 7.16 (m, 5H), 7.13 – 7.05 (m, 2H), 7.02 – 6.96 (m, 2H), 6.94 – 6.85 (m, 2H), 6.73 – 6.66 (m, 1H), 6.19 (s, 1H), 5.45 (t, *J* = 5.3 Hz, 1H), 3.22 – 3.11 (m, 2H), 3.11 – 2.98 (m, 2H); ^{13}C NMR (126 MHz, CDCl$_3$) δ 169.0, 166.7, 150.9, 137.6, 135.0, 133.5, 132.0, 129.9, 129.7, 129.6, 129.3, 129.0, 128.9, 128.5, 128.3, 127.8, 126.9, 125.5, 124.6, 120.6, 119.9, 116.4, 74.4, 39.5, 37.4; HRMS (ESI$^+$) m/z calculated for $C_{29}H_{25}ClNO_4$ [M+H]$^+$: 486.14666, found [M+H]$^+$: 486.14688.

4.1.3. U-4CR

To a stirred solution of amine (1 mmol) in MeOH (3 mL) was added aldehyde (1.2 mmol), carboxylic acid (1 mmol), and isocyanide (1 mmol). The reaction mixture was stirred at rt for 24 h. Afterwards, the solvent was evaporated and the crude product was purified with column chromatography on silica gel eluted with petroleum ether/ethyl acetate (9:1) affording the target products.

Methyl 4-(2-(N-(4-aminobenzyl)-2-cyanoacetamido)-4-(methylthio)butanamido)butanoate (Destination plate I, J20)

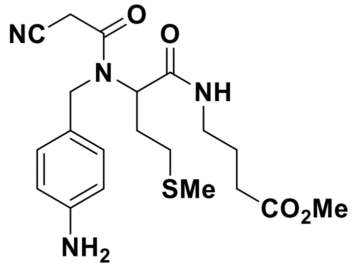

Brown oil, 92mg, 22% yield; ^{1}H NMR (500 MHz, CDCl$_3$) δ 6.95 (d, *J* = 8.2 Hz, 2H), 6.67 (d, *J* = 8.2 Hz, 2H), 6.61 (t, *J* = 6.4 Hz, 2H), 5.10 – 4.95 (m, 1H), 4.58 – 4.43 (m, 2H), 3.76 (br s, 2H), 3.69 (s, 4H), 3.57 – 3.40 (m, 2H), 3.30 – 3.20 (m, 2H), 2.53 – 2.41 (m, 2H), 2.36 – 2.31 (m, 3H), 2.06 (s, 4H), 1.94 – 1.85 (m, 1H), 1.85 – 1.77 (m, 2H); ^{13}C NMR (126 MHz, CDCl$_3$) δ 173.7, 169.2, 164.3, 146.4, 127.2, 124.8, 115.6, 113.9, 57.6, 51.8, 49.0, 38.9, 31.4, 30.7, 27.7, 26.1, 24.4, 15.3; HRMS (ESI$^+$) m/z calculated for $C_{20}H_{29}N_4O_4S$ [M+H]$^+$: 421.1904, found [M+H]$^+$: 421.19037.

N-(2-(cyclohexylamino)-2-oxoethyl)-N-isobutyl-1-methylcyclohexane-1-carboxamide (Destination plate I, K9)

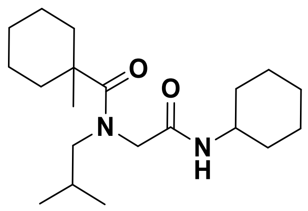

White solid, 245mg, 73% yield; ^{1}H NMR (500 MHz, CDCl$_3$) δ 6.49 (br s, 1H), 4.03 (s, 2H), 3.87 – 3.68 (m, 1H), 3.28 (d, *J* = 6.7 Hz, 2H), 2.17

– 2.03 (m, 3H), 1.92 – 1.80 (m, 2H), 1.74 – 1.65 (m, 2H), 1.64 – 1.44 (m, 6H), 1.41 – 1.30 (m, 5H), 1.26 – 1.23 (m, 3H), 1.20 – 1.10 (m, 3H), 0.94 – 0.85 (m, 6H); ^{13}C NMR (126 MHz, $CDCl_3$) δ 178.3, 168.6, 56.7, 53.1, 47.9, 43.2, 37.3, 32.8, 26.7, 25.8, 25.3, 24.7, 24.6, 23.2, 19.8; HRMS (ESI$^+$) m/z calculated for $C_{20}H_{37}N_2O_2$ [M+H]$^+$: 337.28495, found [M+H]$^+$: 337.2850.

4.1.4. UT-4CR

To a stirred solution of amine (1 mmol) in MeOH (2 mL) was added aldehyde (1 mmol), $TMSN_3$ (1 mmol), and isocyanide (1 mmol). The reaction mixture was stirred at rt for 24 h. Afterwards, the solvent was evaporated and the crude product was purified with column chromatography on silica gel eluted with petroleum ether/ethyl acetate (1:1) affording the target products.

N-((1-cyclohexyl-1H-tetrazol-5-yl)(1H-indazol-6-yl)methyl)cyclopropanamine (Destination plate I, O17)

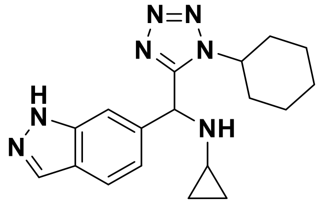

White solid, 300mg, 89% yield; ^{1}H NMR (500 MHz, $CDCl_3$) δ 11.77 (br s, 1H), 8.05 (s, 1H), 7.72 (d, *J* = 8.3 Hz, 1H), 7.56 (s, 1H), 7.15 (dd, *J* = 8.3, 1.5 Hz, 1H), 5.34 (s, 1H), 4.33 – 4.15 (m, 1H), 3.00 (s, 1H), 2.18 – 2.07 (m, 1H), 1.98 – 1.82 (m, 3H), 1.82 – 1.70 (m, 2H), 1.69 – 1.59 (m, 1H), 1.49 – 1.39 (m, 1H), 1.38 – 1.28 (m, 1H), 1.26 – 1.12 (m, 2H), 0.56 – 0.35 (m, 4H); ^{13}C NMR (126 MHz, $CDCl_3$) δ 155.0, 140.2, 136.9, 134.2, 123.0, 121.5, 120.4, 108.9, 58.0, 57.8, 32.6, 32.3, 28.7, 25.2, 25.1, 24.6, 6.6; HRMS (ESI$^+$) m/z calculated for $C_{18}H_{24}N_7$ [M+H]$^+$: 338.20877, found [M+H]$^+$: 338.20905.

N-allyl-N-(2-methyl-1-(1-(thiophen-2-ylmethyl)-1H-tetrazol-5-yl)propyl)prop-2-en-1-amine (Destination plate I, P16)

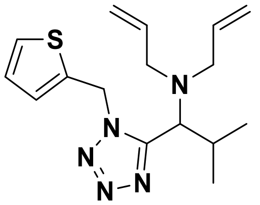

Off-white solid, 279mg, 88% yield; ^{1}H NMR (500 MHz, $CDCl_3$) δ 7.32 – 7.28 (m, 1H), 7.05 (d, *J* = 3.2 Hz, 1H), 6.99 – 6.94 (m, 1H), 5.87 – 5.76 (m, 3H), 5.57 (d, *J* = 15.7 Hz, 1H), 5.29 – 5.16 (m, 4H), 3.70 (d, *J* = 10.6 Hz, 1H), 3.62 – 3.49 (m, 2H), 2.76 – 2.59 (m, 2H), 2.53 – 2.36 (m, 1H), 1.10 (d, *J* = 6.6 Hz, 3H), 0.27 (d, *J* = 6.6 Hz, 3H); ^{13}C NMR (126 MHz, $CDCl_3$) δ 153.5, 136.8, 135.5, 128.0, 127.1, 127.1, 117.0, 59.4, 52.9, 45.6, 30.3, 20.3, 19.3; HRMS (ESI$^+$) m/z calculated for $C_{16}H_{24}N_5S$ [M+H]$^+$: 318.17469, found [M+H]$^+$: 318.17459.

4.1.5. U-5C-3CR of proline and hydroxy acetaldehyde

To a stirred solution of proline (1 mmol) in CF_3CH_2OH (10 mL) was added hydroxyacetaldehyde dimer (1 mmol) and isocyanide (1 mmol). The reaction mixture was stirred at rt for 24 h. Afterwards, the solvent was evaporated and the crude product was purified with column chromatography on silica gel eluted with petroleum ether/ethyl acetate (1:1) affording the target products.

(7S,8aR)-7-Hydroxy-1-oxo-N-(thiophen-2-ylmethyl)hexahydro-1H-pyrrolo[2,1-c][1,4]oxazine-4-carboxamide (Destination plate II, E7)

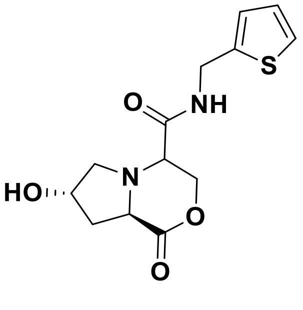

Red oil, 92mg, 31% yield; dr 1:1, mixture of diastereomers ^{1}H NMR (500 MHz, MeOD) δ 7.30 – 7.22 (m, 1H), 7.04 – 6.98 (m, 1H), 6.95 – 6.87 (m, 1H), 5.09 (s, 1H), 4.85 (s, 2H), 4.68 – 4.50 (m, 1H), 4.42 – 4.29 (m, 1H), 4.23 – 4.15 (m, 1H), 4.02 – 3.83 (m, 1H), 3.77 – 3.37 (m, 1H), 3.09 – 2.69 (m, 1H), 2.67 – 2.47 (m, 1H), 2.27 – 2.08 (m, 1H); mixture of diastereomers ^{13}C NMR (126 MHz, MeOD) δ 174.9, 174.7, 172.6, 171.1, 142.7, 140.3, 128.5, 127.7, 127.4, 126.7, 126.4, 125.9, 71.0, 68.2, 64.1, 63.7, 62.5, 62.3, 59.1, 57.0, 40.1, 38.7, 37.8, 37.5; HRMS (ESI$^+$) m/z calculated for $C_{13}H_{17}N_2O_4S$ [M+H]$^+$: 297.09035, found [M+H]$^+$: 297.09055.

(7S,8aR)-N-([1,1'-biphenyl]-2-yl)-7-hydroxy-1-oxohexahydro-1H-pyrrolo[2,1-c][1,4]oxazine-4-carboxamide (Destination plate II, F9)

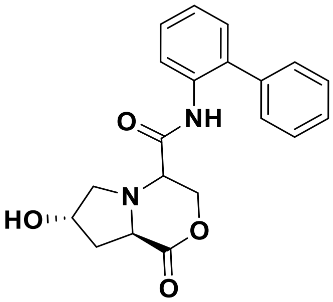

Off-white solid, 109mg, 31% yield; ^1H NMR (500 MHz, $CDCl_3$) δ 9.48 (s, 1H), 8.20 (d, *J* = 8.2 Hz, 1H), 7.54 – 7.47 (m, 2H), 7.46 – 7.41 (m, 1H), 7.41 – 7.35 (m, 3H), 7.28 – 7.25 (m, 1H), 7.24 – 7.20 (m, 1H), 4.59 (dd, *J* = 11.9, 4.8 Hz, 1H), 4.26 – 4.16 (m, 1H), 4.10 (t, *J* = 11.6 Hz, 1H), 3.76 (dd, *J* = 8.6, 4.8 Hz, 1H), 3.36 (dd, *J* = 11.3, 4.8 Hz, 1H), 2.86 – 2.73 (m, 1H), 2.64 – 2.52 (m, 1H), 2.45 (dd, *J* = 10.2, 4.2 Hz, 1H), 1.96 – 1.82 (m, 1H), 1.51 (s, 1H); ^{13}C NMR (126 MHz, $CDCl_3$) δ 171.8, 166.2, 138.3, 133.6, 133.0, 130.1, 129.4, 128.8, 128.4, 127.8, 125.0, 121.3, 69.5, 66.9, 62.4, 62.0, 55.6, 35.7; HRMS (ESI$^+$) m/z calculated for $C_{20}H_{21}N_2O_4$ [M+H]$^+$: 353.14958, found [M+H]$^+$: 353.14944.

4.1.6. UH-4CR

To a stirred solution of the primary amine (1 mmol) in MeOH/H_2O (1:1, 1 mL) was added aldehyde (1 mmol), isocyanide (1 mmol), KNCO (2 mmol) and Py·HCl (2 mmol). The reaction mixture was stirred at rt for 4 h. Afterwards, the solvent was evaporated and the crude product was purified with column chromatography on silica gel eluted with MeOH/DCM (4%) affording the target products.

(Z)-1-(5-Hydroxypentyl)-5-(1-phenylethyl)-4-((thiophen-2-ylmethyl)imino)imidazolidin-2-one (Destination plate II, J6)

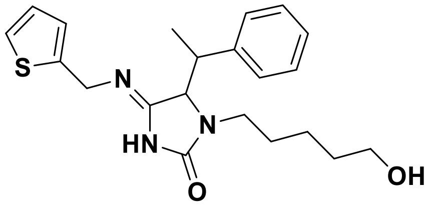

Brown solid, 100mg, 26% yield; ^1H NMR (500 MHz, $CDCl_3$) δ 7.31 – 7.27 (m, 2H), 7.26 – 7.22 (m, 2H), 7.21 – 7.18 (m, 2H), 6.97 – 6.86 (m, 2H), 5.04 (t, *J* = 5.5 Hz, 1H), 4.87 – 4.73 (m, 1H), 4.44 – 4.32 (m, 2H), 3.93 – 3.77 (m, 1H), 3.62 (t, *J* = 6.4 Hz, 2H), 3.47 (s, 1H), 3.17 – 3.06 (m, 1H), 2.99 (br s, 1H), 1.73 – 1.56 (m, 4H), 1.49 – 1.35 (m, 2H), 1.16 (d, *J* = 7.2 Hz, 3H); ^{13}C NMR (126 MHz, $CDCl_3$) δ 174.9, 167.9, 138.9, 138.7, 128.9, 127.8, 127.5, 126.8, 125.4, 65.3, 62.0, 40.6, 40.1, 37.7, 32.0, 27.9, 22.9, 11.1; HRMS (ESI$^+$) m/z calculated for $C_{21}H_{28}N_3O_2S$ [M+H]$^+$: 386.18967, found [M+H]$^+$: 386.1893.

4.1.7. Intramolecular U-5C-3CR of α-amino acids with nucleophilic side chains

To a stirred solution of α-amino acid with nucleophilic side chains (1 mmol) in CF_3CH_2OH (10 mL) was added aldehyde (1 mmol) and isocyanide (1 mmol). The reaction mixture was stirred at rt for 24 h. Afterwards, the solvent was evaporated and the crude product was purified with column chromatography on silica gel eluted with petroleum ether/ethyl acetate (50%-30%) affording the target products.

2-(Naphthalen-1-yl)-2-((2-oxotetrahydrofuran-3-yl)amino)-N-(2,4,4-trimethylpentan-2-yl)acetamide (Destination plate II, M6)

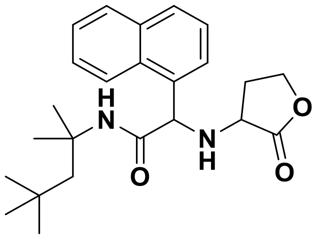

Brown oil, 242mg, 61% yield; dr 10:7, major diastereomer ^1H NMR (500 MHz, $CDCl_3$) δ 8.24 (d, *J* = 8.2 Hz, 1H), 7.84 – 7.79 (m, 2H), 7.58 – 7.34 (m, 4H), 6.26 (s, 1H), 5.06 (s, 1H), 4.16 – 4.08 (m, 1H), 3.97 – 3.87 (m, 1H), 3.43 – 3.35 (m, 1H), 2.83 (br s, 1H), 2.48 – 2.39 (m, 1H), 1.80 – 1.62 (m, 3H), 1.35 (s, 3H), 1.28 (s, 3H), 0.72 (s, 9H); mixture of diastereomers ^{13}C NMR (126 MHz, $CDCl_3$) δ = 176.8, 176.5, 170.2, 170.0, 134.5, 134.4, 134.0, 133.9, 131.2, 131.1, 128.8, 128.6, 128.5, 128.4, 126.3, 126.1, 125.7, 125.6, 125.0, 124.1, 123.3, 65.3, 65.1, 54.9, 54.8, 54.5, 52.6,

52.4, 31.2, 31.1, 31.0, 30.9, 30.4, 30.3, 28.2, 27.7; HRMS (ESI+) m/z calculated for $C_{24}H_{33}N_2O_3$ $[M+H]^+$: 397.24857, found $[M+H]^+$: 397.24844.

N-(4-chlorophenyl)-2-(naphthalen-1-yl)-2-(((S)-2-oxoazepan-3-yl)amino)acetamide (Destination plate II, O8)

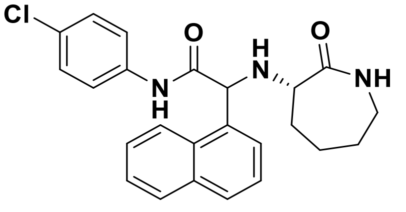

White solid, 168mg, 40% yield; ^{1}H NMR (500 MHz, $CDCl_3$) δ 9.98 (s, 1H), 8.15 (d, *J* = 8.2 Hz, 1H), 7.89 (dd, *J* = 8.0, 1.5 Hz, 1H), 7.84 (d, *J* = 8.2 Hz, 1H), 7.65 – 7.57 (m, 3H), 7.57 – 7.43 (m, 3H), 7.25 (d, *J* = 8.7 Hz, 2H), 6.11 (s, 1H), 5.05 (s, 1H), 3.45 (d, *J* = 10.6 Hz, 1H), 3.24 – 3.03 (m, 2H), 2.03 – 1.31 (m, 7H); ^{13}C NMR (126 MHz, $CDCl_3$) δ 177.9, 170.8, 136.8, 135.0, 134.2, 131.3, 129.0, 128.8, 128.7, 126.7, 126.0, 125.9, 125.4, 123.6, 120.7, 64.1, 59.8, 41.9, 32.5, 28.7, 28.1; HRMS (ESI+) m/z calculated for $C_{24}H_{25}ClN_3O_2$ $[M+H]^+$: 422.16298, found $[M+H]^+$: 422.1631.

4.1.8. vL-3CR

To a stirred solution of primary amine (1 mmol) in THF (2 mL) was added aldehyde (1 mmol), substituted tosmic isocyanide (1 mmol) and piperazine (1.3 mmol). The reaction mixture was stirred at rt for 24 h. Afterwards, the solvent was evaporated and the crude product was purified with column chromatography on silica gel eluted with petroleum ether/ethyl acetate (1:1) or MeOH/DCM (3%-8%) affording the target products.

4-((4-(4-Fluorophenyl)-5-(2-(methylthio)ethyl)-1H-imidazol-1-yl)methyl)aniline (Destination plate III, A8)

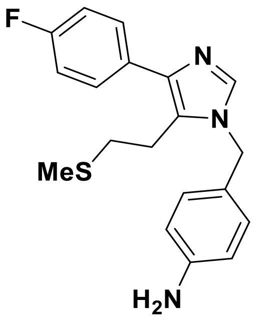

Light yellow solid, 181mg, 53% yield; ^{1}H NMR (500 MHz, $CDCl_3$) δ 7.66 – 7.57 (m, 2H), 7.49 (s, 1H), 7.08 (t, *J* = 8.8 Hz, 2H), 6.93 (d, *J* = 8.4 Hz, 2H), 6.65 (d, *J* = 8.4 Hz, 2H), 5.02 (s, 2H), 3.75 (s, 2H), 3.03 – 2.93 (m, 2H), 2.53 – 2.42 (m, 2H), 2.01 (s, 3H); ^{13}C NMR (126 MHz, $CDCl_3$) δ 161.7 (d, *J* = 245.4 Hz), 146.4, 138.2, 136.9, 131.3 (d, *J* = 3.2 Hz), 128.3 (d, *J* = 7.8 Hz), 128.1, 125.9, 125.4, 115.3 (d, *J* = 21.5 Hz), 115.2, 48.7, 33.1, 24.4, 15.5; HRMS (ESI+) m/z calculated for $C_{19}H_{21}FN_3S$ $[M+H]^+$: 342.14347, found $[M+H]^+$: 342.14343.

2-(5-(3,4-Dimethoxyphenyl)-4-(4-fluorophenyl)-1H-imidazol-1-yl)benzoic acid (Destination plate III, B18)

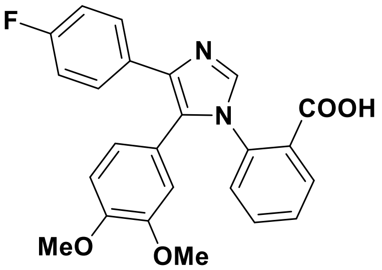

Light yellow solid, 150mg, 36% yield; ^{1}H NMR (500 MHz, $CDCl_3$) δ 11.23 (br s, 1H), 8.10 (s, 1H), 7.96 (dd, *J* = 7.8, 1.6 Hz, 1H), 7.41 (t, *J* = 7.6 Hz, 1H), 7.37 – 7.31 (m, 3H), 6.99 (d, *J* = 7.6 Hz, 1H), 6.74 – 6.67 (m, 5H), 3.82 (s, 3H), 3.50 (s, 3H); ^{13}C NMR (126 MHz, $CDCl_3$) δ 168.7, 161.8 (d, *J* = 247.1 Hz), 149.1, 148.7, 137.5, 134.1, 133.6, 132.8, 131.1, 131.0, 129.8, 129.3, 128.8 (d, *J* = 7.9 Hz), 128.6, 128.1 (d, *J* = 3.2 Hz), 123.6, 120.7, 115.2 (d, *J* = 21.5 Hz), 113.4, 110.9, 55.7, 55.6; HRMS (ESI+) m/z calculated for $C_{24}H_{20}FN_2O_4$ $[M+H]^+$: 419.14016, found $[M+H]^+$: 419.1400.

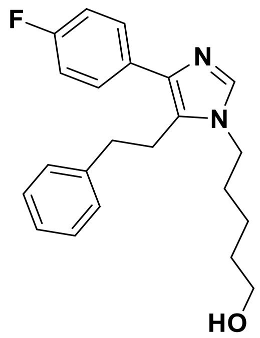

5-(4-(4-Fluorophenyl)-5-phenethyl-1H-imidazol-1-yl)pentan-1-ol (Destination plate III, D5)

Light yellow solid, 278mg, 79% yield; ^{1}H NMR (500 MHz, $CDCl_3$) δ 7.61 – 7.53 (m, 2H), 7.42 (s, 1H), 7.30 – 7.22 (m, 2H), 7.23 – 7.16 (m, 1H), 7.13 – 7.00 (m, 4H), 3.65 (t, *J* = 7.4 Hz, 2H), 3.58 (t, *J* = 6.4 Hz, 2H), 3.16 – 2.96 (m, 3H), 2.83 (t, *J* = 7.8 Hz, 2H), 1.74 – 1.63 (m, 2H), 1.58 – 1.48 (m, 2H), 1.41 – 1.30 (m, 2H); ^{13}C NMR (126 MHz, $CDCl_3$) δ 161.6 (d, *J* = 245.2 Hz), 140.3, 137.2, 135.8, 131.4 (d, *J* = 3.2 Hz), 128.5, 128.4 (d, *J* = 7.8 Hz), 128.2, 126.4, 115.1 (d, *J* = 21.4 Hz), 61.8, 44.7, 35.7, 32.0, 30.7, 25.8, 23.1; HRMS (ESI$^+$) m/z calculated for $C_{22}H_{26}FN_2O$ [M+H]$^+$: 353.20237, found [M+H]$^+$: 353.20251.

4.1.9. U-5C-4CR with additional amine

To a stirred solution of proline (1 mmol) in MeOH/H_2O (4:1, 10 mL) was added aldehyde (1 mmol), amine (1 mmol), and isocyanide (1 mmol). The reaction mixture was stirred at rt for 24 h. Afterwards, the solvent was evaporated and the crude product was purified with column chromatography on silica gel eluted with petroleum ether/ethyl acetate (100%-70%) or MeOH/DCM (3%-8%) affording the targeted compounds.

N-(4-chlorobenzyl)-2-((R)-2-(4-(4-methoxyphenyl)piperazine-1-carbonyl)pyrrolidin-1-yl)-2-(pyridin-3-yl)acetamide (Destination plate IV, A4)

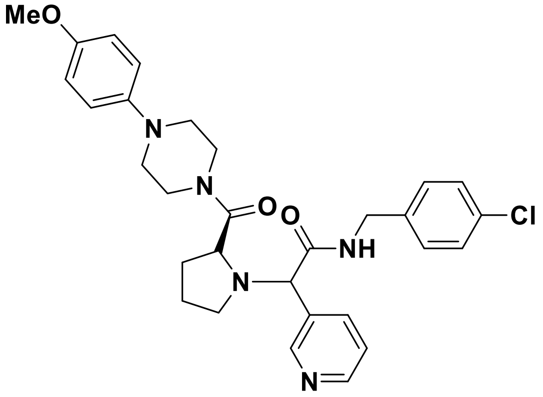

Brown solid, 181mg, 33% yield; dr 5:2, mixture of diastereomers ^{1}H NMR (500 MHz, $CDCl_3$) δ 9.38 (t, *J* = 6.1 Hz, 1H), 8.61 – 8.45 (m, 2H), 7.74 – 7.47 (m, 1H), 7.36 – 7.26 (m, 3H), 7.27 – 7.22 (m, 1H), 7.21 – 7.14 (m, 1H), 6.91 – 6.79 (m, 4H), 4.60 – 4.32 (m, 3H), 3.77 (s, 3H), 3.74 – 2.71 (m, 10H), 2.50 – 2.38 (m, 1H), 2.13 – 1.64 (m, 4H); mixture of diastereomers ^{13}C NMR (126 MHz, $CDCl_3$) δ 173.2, 172.1, 171.8, 170.9, 154.5, 154.4, 151.1, 150.6, 149.6, 149.2, 144.9, 137.4, 137.3, 137.2, 136.4, 132.9, 129.2, 129.0, 128.6, 123.3, 123.1, 118.9, 118.8, 114.5, 70.2, 67.3, 60.7, 58.7, 55.5, 54.9, 51.0, 50.9, 50.7, 50.6, 49.2, 45.1, 44.7, 42.6, 42.5, 42.1, 42.0, 30.8, 29.7, 24.4, 23.5; HRMS (ESI$^+$) m/z calculated for $C_{30}H_{35}ClN_5O_3$ [M+H]$^+$: 548.24229, found [M+H]$^+$: 548.24243.

(2S)-N-(3-chloro-4-fluorophenyl)-1-(2-((4-fluorophenethyl)amino)-1-(4-methoxyphenyl)-2-oxoethyl)pyrrolidine-2-carboxamide (Destination plate IV, A17)

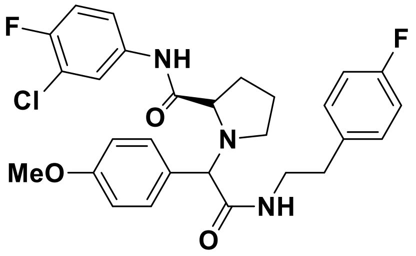

Light yellow oil, 111mg, 21% yield; dr 4:1, major diastereomer ^{1}H NMR (500 MHz, $CDCl_3$) δ 10.17 (s, 1H), 7.81 (dd, *J* = 6.7, 2.6 Hz, 1H), 7.49 – 7.40 (m, 1H), 7.19 – 6.74 (m, 9H), 5.72 – 5.57 (m, 1H), 4.08 (s, 1H), 3.75 (s, 3H), 3.65 – 2.67 (m, 7H), 2.10 – 1.68 (m, 4H); ^{13}C NMR (126 MHz, $CDCl_3$) δ 173.6, 172.1, 161.6 (d, *J* = 244.6 Hz), 159.9, 154.4 (d, *J* = 245.2 Hz), 134.9 (d, *J* = 3.3 Hz), 134.1 (d, *J* = 3.3 Hz), 130.3, 130.1, 128.0, 121.3, 118.9 (d, *J* = 6.8 Hz), 116.4 (d, *J* = 22.0 Hz), 115.3 (d, *J* = 21.1 Hz), 114.3, 71.8, 63.8, 55.2, 53.9, 40.7, 34.7, 31.0, 24.8; HRMS (ESI$^+$) m/z calculated for $C_{28}H_{29}ClF_2N_3O_3$ [M+H]$^+$: 528.18600, found [M+H]$^+$: 528.18604.

N-cyclohexyl-2-((R)-2-(indoline-1-carbonyl)pyrrolidin-1-yl)butanamide (Destination plate IV, B12, diastereomer A**)**

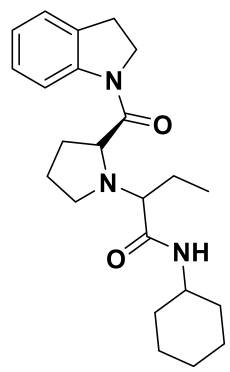

Light yellow oil, 19mg, 5% yield; ^{1}H NMR (500 MHz, $CDCl_3$) δ 8.26 (d, *J* = 8.0 Hz, 1H), 7.70 (d, *J* = 8.3 Hz, 1H), 7.24 – 7.16 (m, 2H), 7.05 – 6.99 (m, 1H), 4.16 – 4.07 (m, 1H), 4.05 – 3.98 (m, 1H), 3.82 (dd, *J* = 9.7, 2.3 Hz, 1H), 3.68 – 3.62 (m, 1H), 3.20 (t, *J* = 8.5 Hz, 2H), 3.16 – 3.11 (m, 2H), 2.78 – 2.69 (m, 1H), 2.21 – 2.14 (m, 1H), 2.02 – 1.96 (m, 1H), 1.91 – 1.85 (m, 4H), 1.79 – 1.75 (m, 1H), 1.71 – 1.68 (m, 2H), 1.58 – 1.52 (m, 1H), 1.37 – 1.29 (m, 2H), 1.25 – 1.20 (m, 2H), 1.15 – 1.10 (m, 2H), 0.95 (t, *J* = 7.4 Hz, 3H); ^{13}C NMR (126 MHz, $CDCl_3$) δ 173.8, 172.2, 143.1, 130.8, 127.5, 124.5, 123.8, 117.3, 67.7, 64.6, 48.9, 47.8, 47.4, 33.1, 32.3, 30.5, 28.2, 25.6, 24.8, 24.7, 24.3, 23.0, 10.9; HRMS (ESI$^+$) m/z calculated for $C_{23}H_{34}N_3O_2$ [M+H]$^+$: 383.26455, found [M+H]$^+$: 384.26465.

N-cyclohexyl-2-((R)-2-(indoline-1-carbonyl)pyrrolidin-1-yl)butanamide (Destination plate IV, B12, diastereomer B**)**

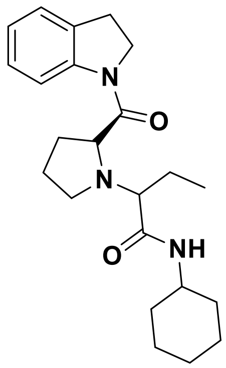

Light yellow oil, 42mg, 11% yield; ^{1}H NMR (500 MHz, $CDCl_3$) δ δ 8.26 (d, *J* = 8.1 Hz, 1H), 7.48 (d, *J* = 8.1 Hz, 1H), 7.24 – 7.17 (m, 2H), 7.06 – 7.00 (m, 1H), 4.14 – 4.07 (m, 2H), 3.87 (dd, *J* = 9.2, 3.5 Hz, 1H), 3.77 – 3.72 (m, 1H), 3.31 – 3.24 (m, 1H), 3.21 (t, *J* = 8.4 Hz, 2H), 3.17 – 3.13 (m, 1H), 2.84 (q, *J* = 8.4 Hz, 1H), 2.24 – 2.18 (m, 1H), 2.00 – 1.96 (m, 1H), 1.91 – 1.82 (m, 4H), 1.77 – 1.72 (m, 1H), 1.69 – 1.64 (m, 2H), 1.60 – 1.55 (m, 1H), 1.41 – 1.28 (m, 3H), 1.22 – 1.09 (m, 3H), 0.94 (t, *J* = 7.5 Hz, 3H); ^{13}C NMR (126 MHz, $CDCl_3$) δ 173.6, 172.7, 143.2, 130.9, 127.5, 124.5, 123.8, 117.2, 68.0, 60.4, 52.4, 47.6, 47.4, 33.1, 32.9, 30.5, 28.2, 25.5, 24.8, 24.4, 23.7, 11.3; HRMS (ESI$^+$) m/z calculated for $C_{23}H_{34}N_3O_2$ [M+H]$^+$: 383.26455, found [M+H]$^+$: 384.26436.

4.1.10. UPS-4CR

To a stirred solution of aminoacetaldehyde dimethyl acetal (1 mmol) in MeOH (3 mL) was added aldehyde (1.2 mmol), carboxylic acid (1 mmol), and hetero/phenylethyl isocyanide (1 mmol). The reaction mixture was stirred at rt for 24 h. Afterwards, the solvent was evaporated and the crude product was purified with column chromatography on silica gel eluted with petroleum ether/ethyl acetate (30%-20%) or MeOH/DCM (2%-3%) affording the Ugi-4CR compounds. The corresponding Ugi-4CR compound was refluxed in formic acid (1.2 equivalent) at 60 °C to give the Pictet-Spengler product which was purified with column chromatography on silica gel eluted with petroleum ether/ethyl acetate (50%-20%).

3-Isopropyl-2-(2-(thiophen-2-yl)acetyl)-2,3,6,7,12,12b-hexahydropyrazino [1',2':1,2]pyrido[3,4-b]indol-4(1H)-one (Destination plate IV, H2)

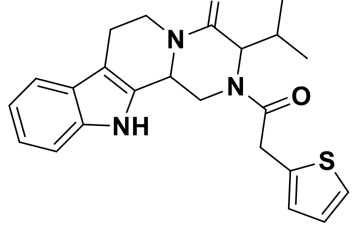

Yellow solid, 277mg, 68% yield; dr 3:1, major diastereomer ^{1}H NMR (500 MHz, DMSO-*d6*) δ 11.22 (s, 1H), 7.47 – 7.37 (m, 2H), 7.35 (dd, *J* = 5.0, 1.4 Hz, 1H), 7.11 (t, *J* = 7.5 Hz, 1H), 7.01 (t, *J* = 7.4 Hz, 1H), 6.94 – 6.87 (m, 2H), 5.20 – 5.10 (m, 1H), 4.76 – 4.64 (m, 1H), 4.48 (d, *J* = 9.2 Hz, 1H), 4.33 – 4.23 (m, 1H), 4.20 – 4.11 (m, 2H), 3.80 (d, *J* = 16.3 Hz, 1H), 3.04 – 2.91 (m, 1H), 2.78 – 2.64 (m, 2H), 2.34 – 2.12 (m, 1H), 1.02 (d, *J* = 6.7 Hz, 3H), 0.83 (d, *J* = 6.7 Hz, 3H); mixture of diastereomers ^{13}C NMR (126 MHz, DMSO-*d*$_6$) δ 168.8, 168.6, 167.4, 166.8, 136.6, 136.0, 132.2, 131.4, 126.5, 126.3, 125.3, 125.2, 125.0,

121.5, 118.9, 117.9, 111.3, 109.4, 108.6, 60.1, 52.1, 51.9, 43.8, 40.8, 33.4, 30.2, 29.9, 20.0, 19.9, 19.8, 19.6; HRMS (ESI+) m/z calculated for $C_{23}H_{26}N_3O_2S$ [M+H]+: 408.17402, found [M+H]+: 408.17404.

4.1.11. SU-4CR

To a stirred solution of bis-secondary amines (1 mmol) in MeOH (2 mL) was added aldehyde (1 mmol), carboxylic acid (1 mmol) and isocyanide (1 mmol). The reaction mixture was refluxed at 60 °C for 2 h. Afterwards, the solvent was evaporated and the crude product was purified with column chromatography on silica gel eluted with petroleum ether/ethyl acetate (100%-50%) affording the target products.

(E)-2-((2-(3-(2,3-Dimethoxyphenyl)-N-methylacrylamido)ethyl)(methyl)amino)-N-(thiophen-2-ylmethyl)pentanamide (Destination plate IV, J10)

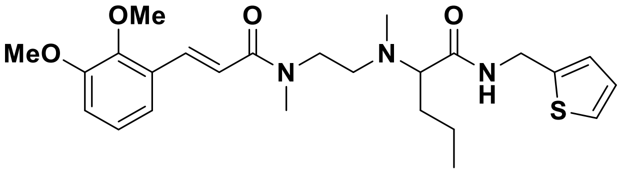

Brown oil, 114mg, 24% yield; mixture of rotamers ^1H NMR (500 MHz, $CDCl_3$) δ 7.89 (d, *J* = 15.6 Hz, 1H), 7.72 – 7.63 (m, 1H), 7.21 – 6.80 (m, 7H), 4.68 – 4.58 (m, 1H), 4.58 – 4.45 (m, 1H), 3.92 – 3.80 (m, 6H), 3.70 – 3.40 (m, 2H), 3.20 – 2.94 (m, 4H), 2.80 – 2.61 (m, 2H), 2.39 – 2.29 (m, 3H), 1.91 – 1.65 (m, 1H), 1.62 – 1.53 (m, 1H), 1.51 – 1.41 (m, 1H), 1.39 – 1.30 (m, 1H), 0.98 – 0.86 (m, 3H); mixture of rotamers ^{13}C NMR (126 MHz, $CDCl_3$) δ 172.7, 172.5, 167.0, 153.1, 148.2, 141.7, 141.3, 137.7, 137.4, 129.3, 126.6, 125.8, 125.5, 124.9, 124.6, 124.1, 124.0, 119.7, 119.5, 119.0, 118.7, 113.3, 113.2, 68.7, 67.2, 61.0, 60.9, 55.8, 53.3, 52.6, 48.3, 46.4, 38.9, 38.3, 37.8, 37.7, 35.6, 34.4, 29.6, 28.8, 20.6, 20.5, 14.2, 14.1.; HRMS (ESI+) m/z calculated for $C_{25}H_{35}N_3O_4S$ [M+H]+: 474.2421, found [M+H]+: 474.24194.

4.2. ^{1}H and ^{13}C NMR spectra

Methyl (2-(3-hydroxyphenyl)-8-methylimidazo[1,2-a]pyridin-3-yl)alaninate (Destination plate I, A22)

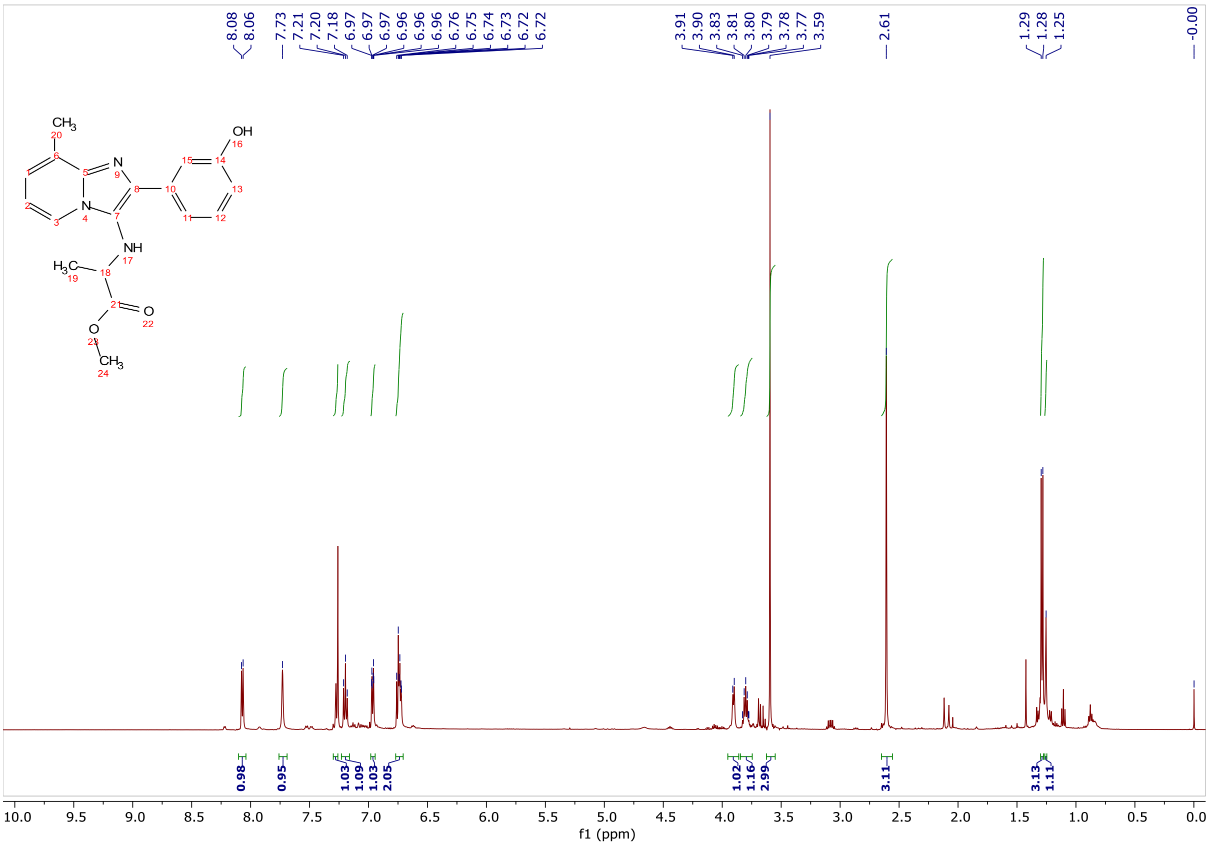

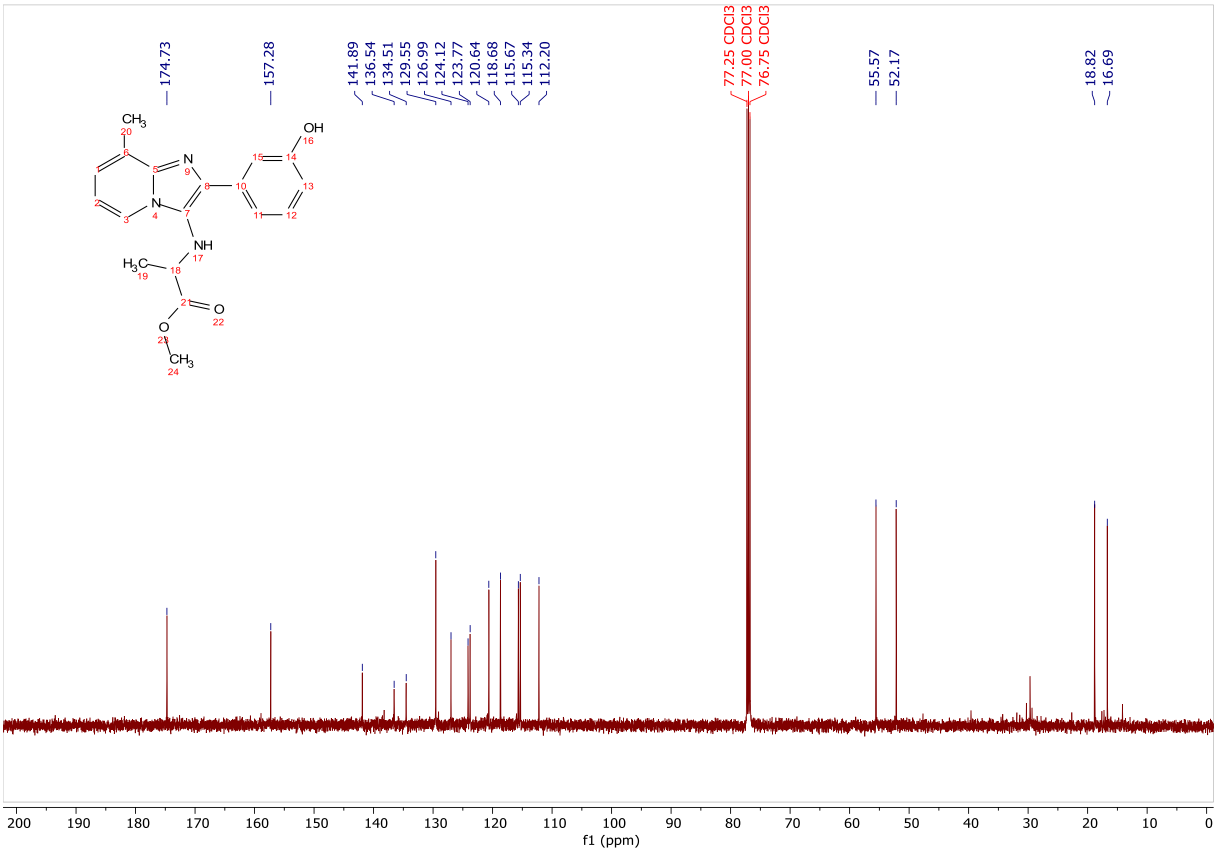

N-(2,3-dimethoxybenzyl)-2-(3-fluorophenyl)-5-(trifluoromethyl)imidazo[1,2-a]pyridin-3-amine (Destination plate I, B1)

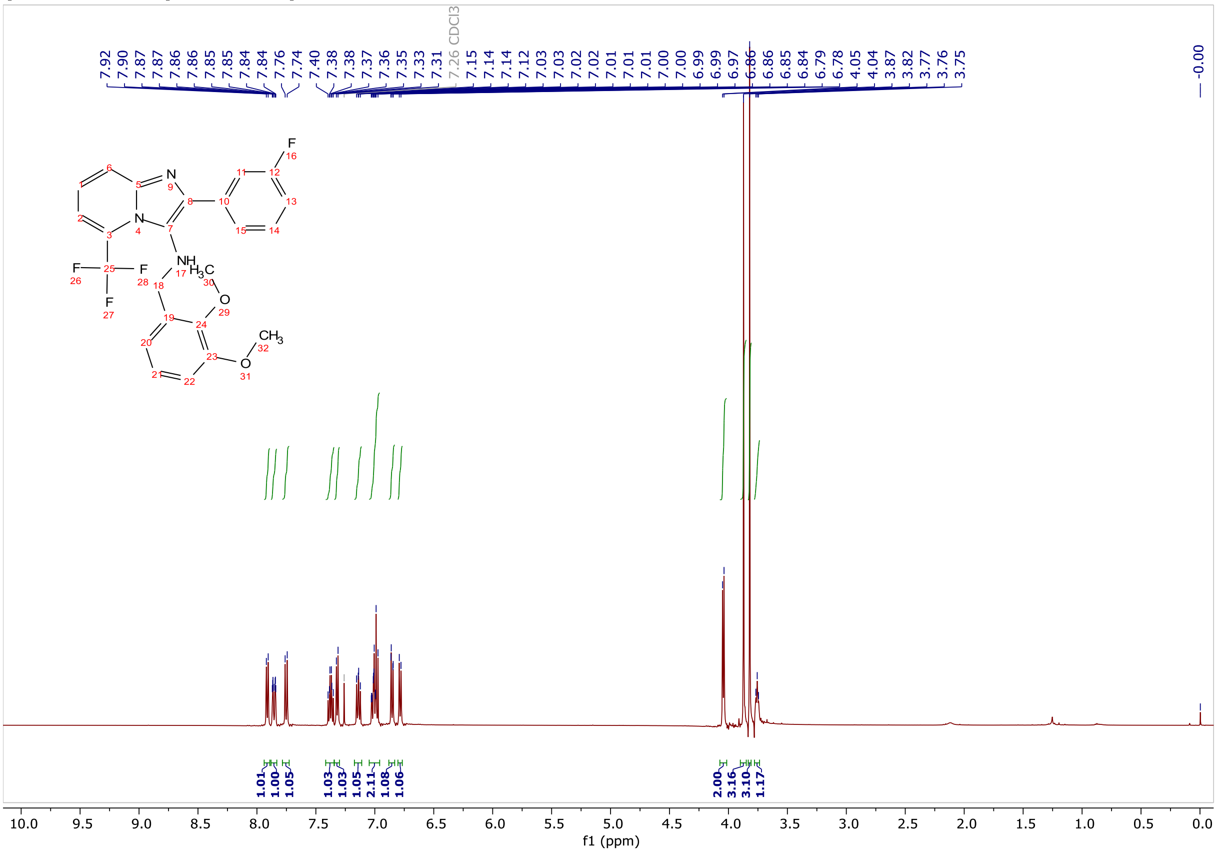

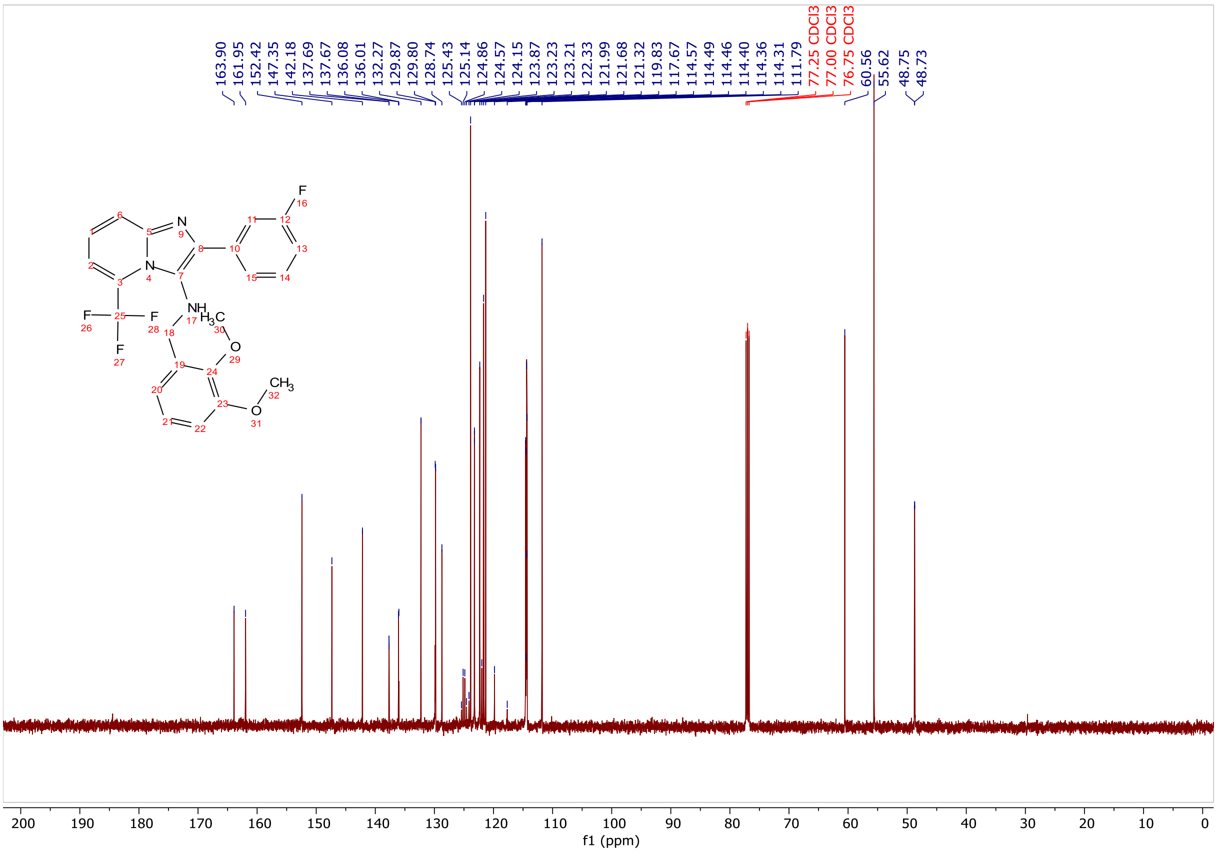

1-(Benzylcarbamoyl)cyclopentyl benzofuran-2-carboxylate (Destination plate I, F16)

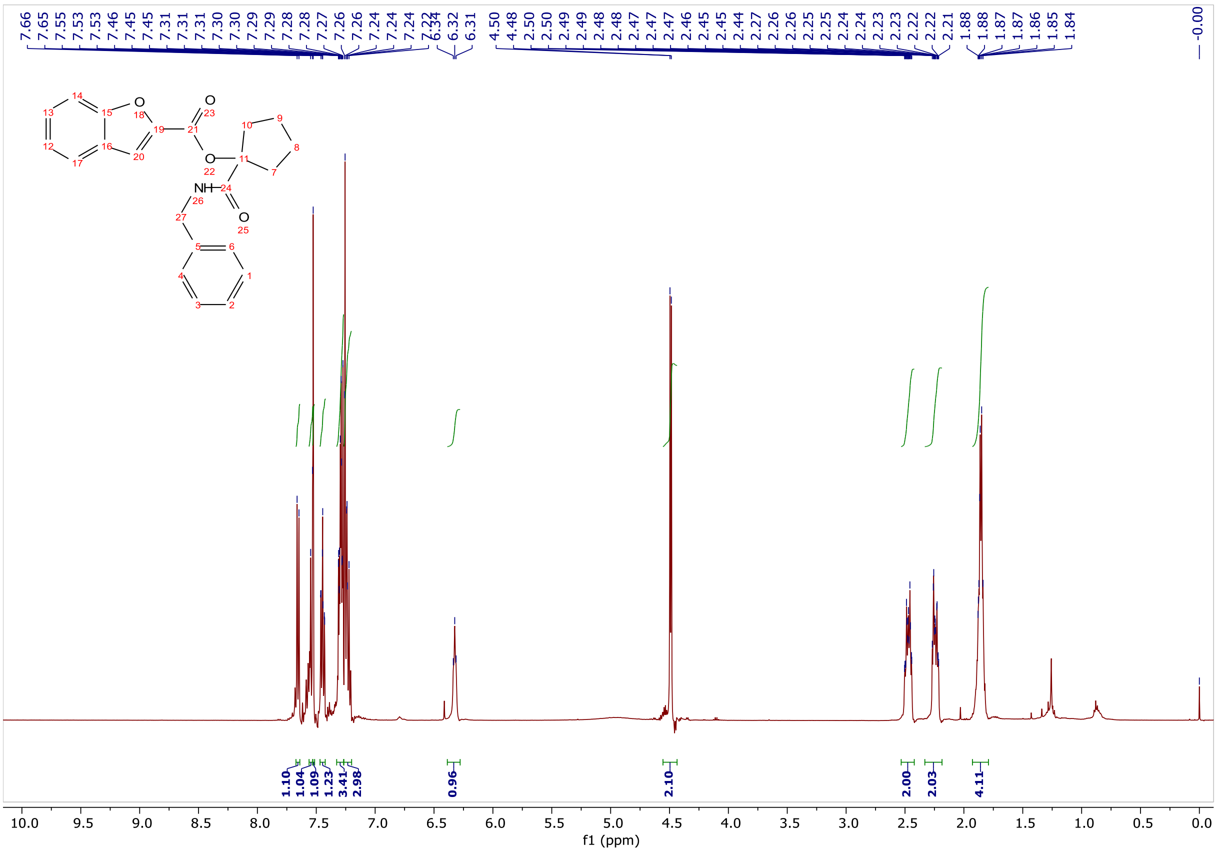

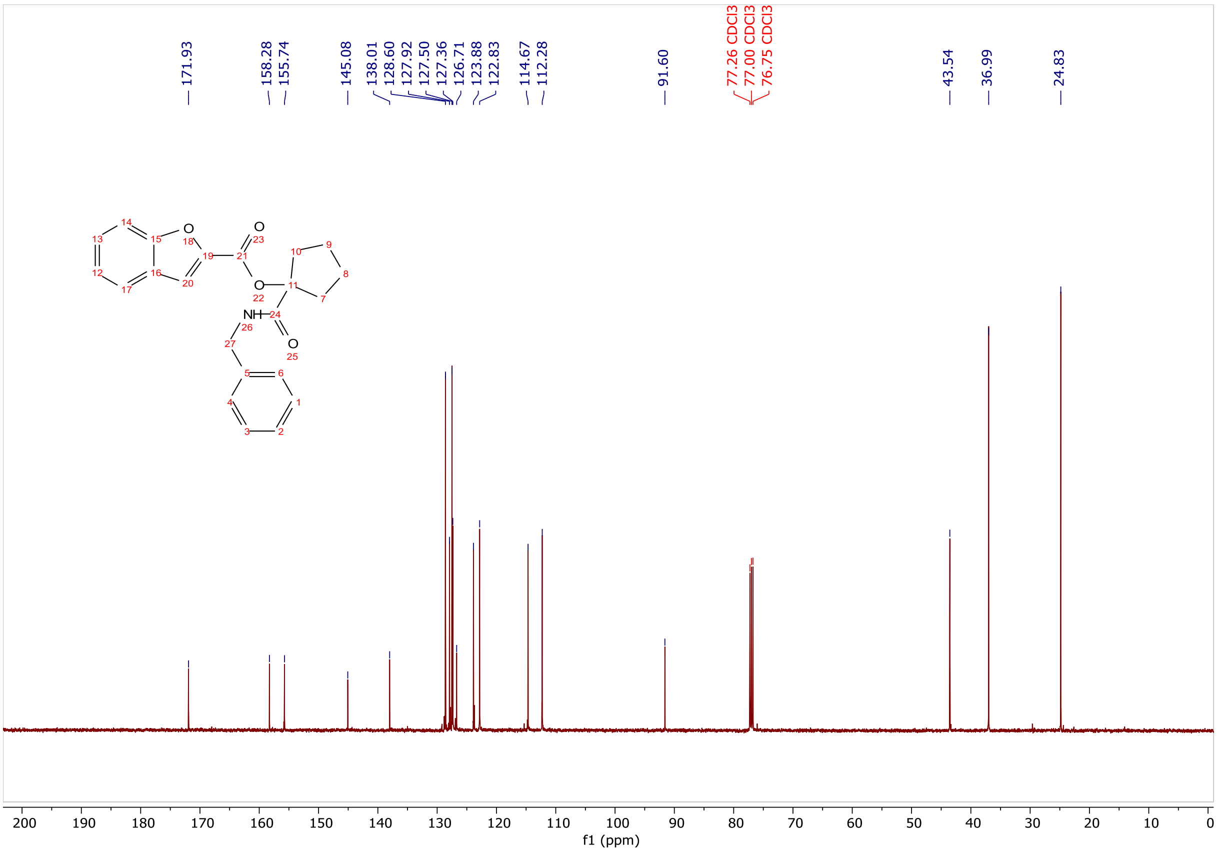

1-((4-Methoxy-4-oxobutyl)amino)-1-oxo-3-phenylpropan-2-yl 2-hydroxy-3-methoxybenzoate (Destination plate I, G5)

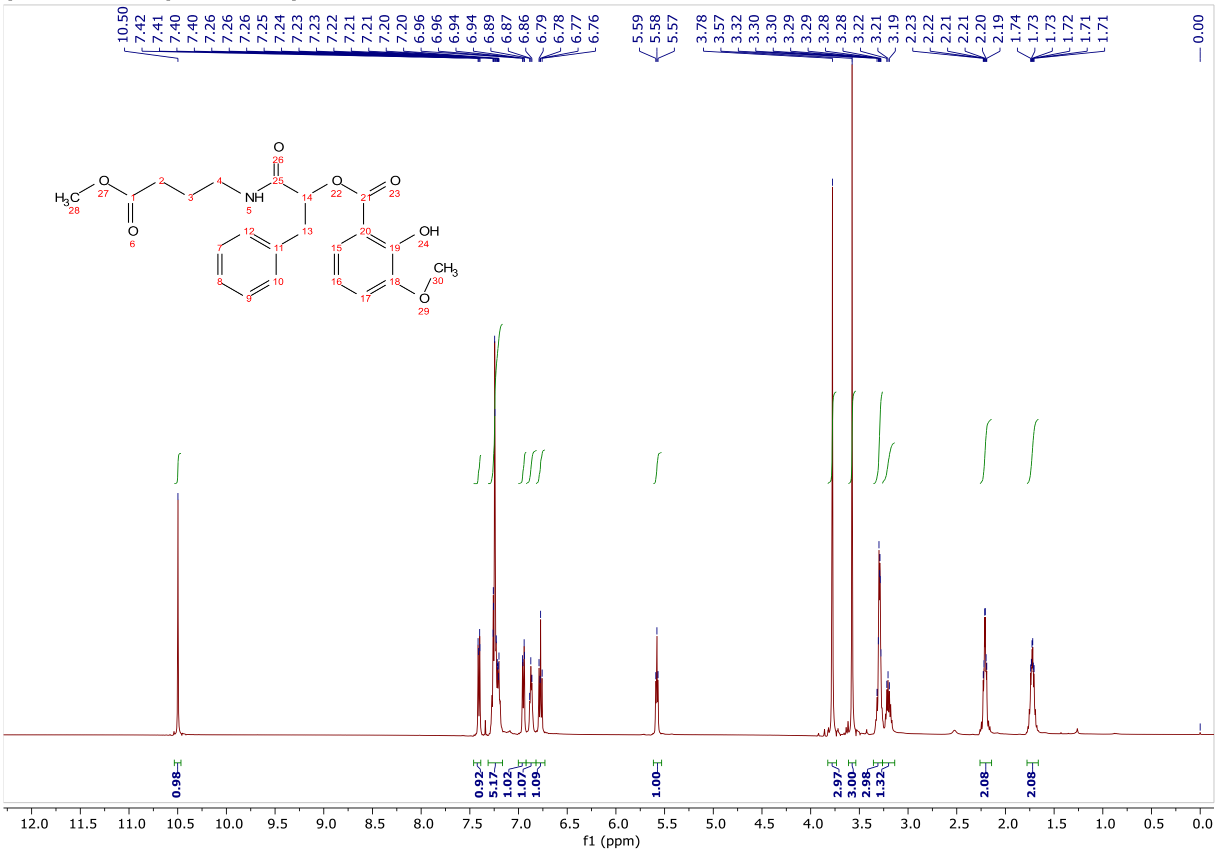

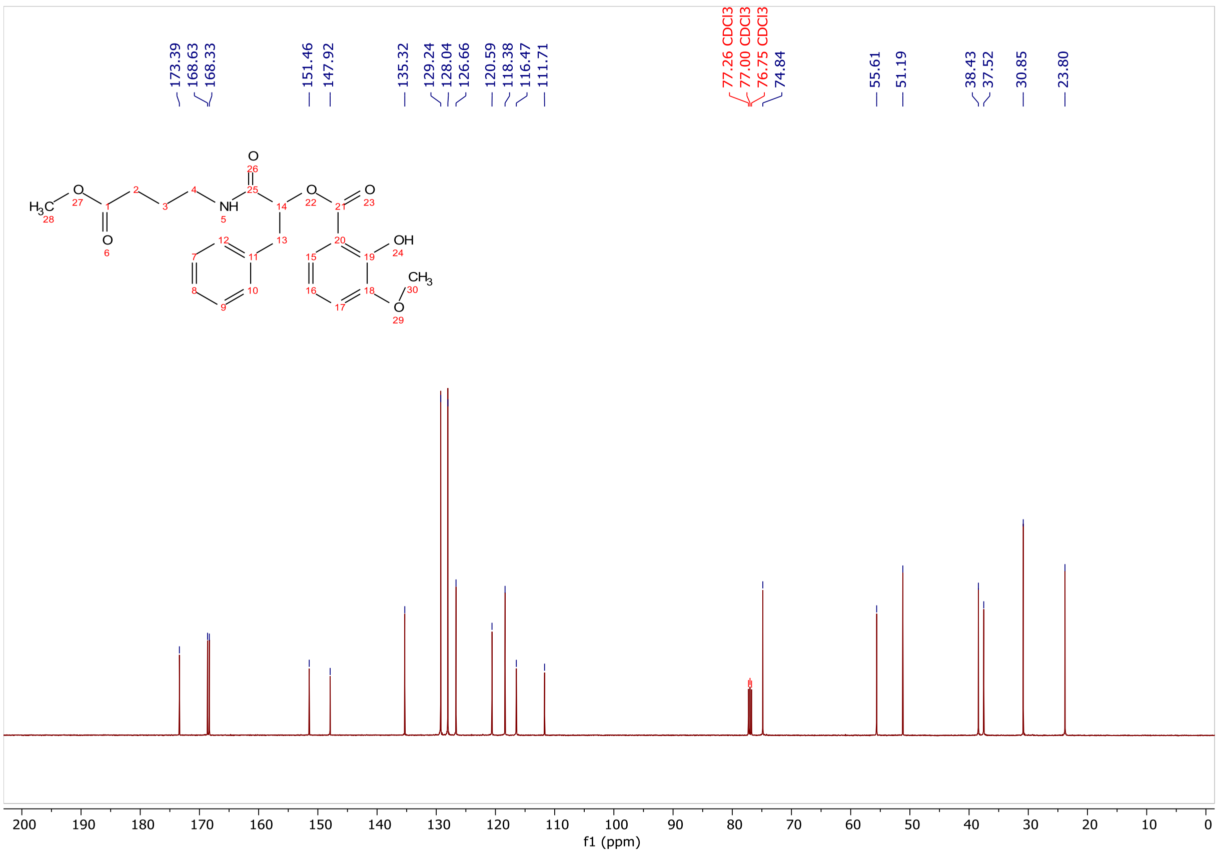

1-([1,1'-Biphenyl]-2-ylamino)-1-oxo-3-phenylpropan-2-yl 2-(3-chloro-4-hydroxyphenyl)acetate (Destination plate I, G6)

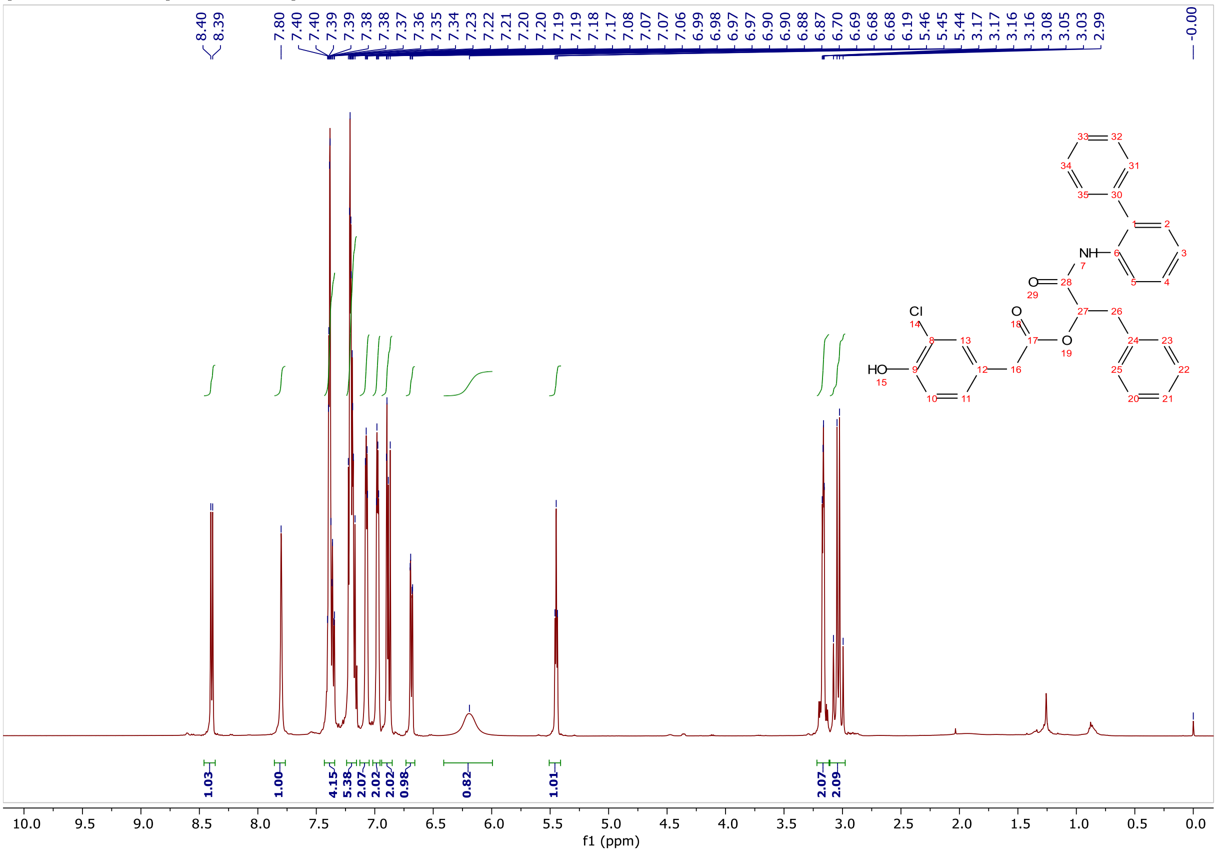

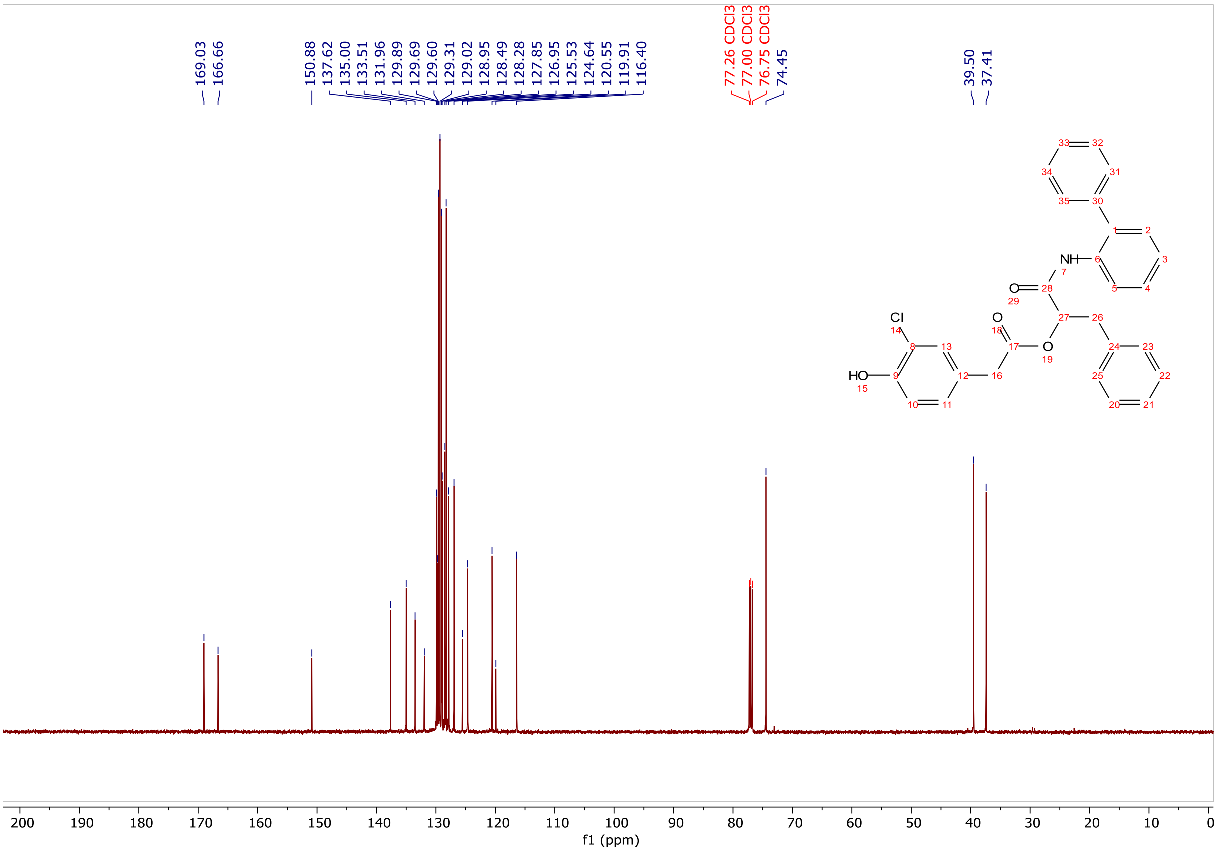

Methyl 4-(2-(N-(4-aminobenzyl)-2-cyanoacetamido)-4-(methylthio)butanamido)butanoate (Destination plate I, J20)

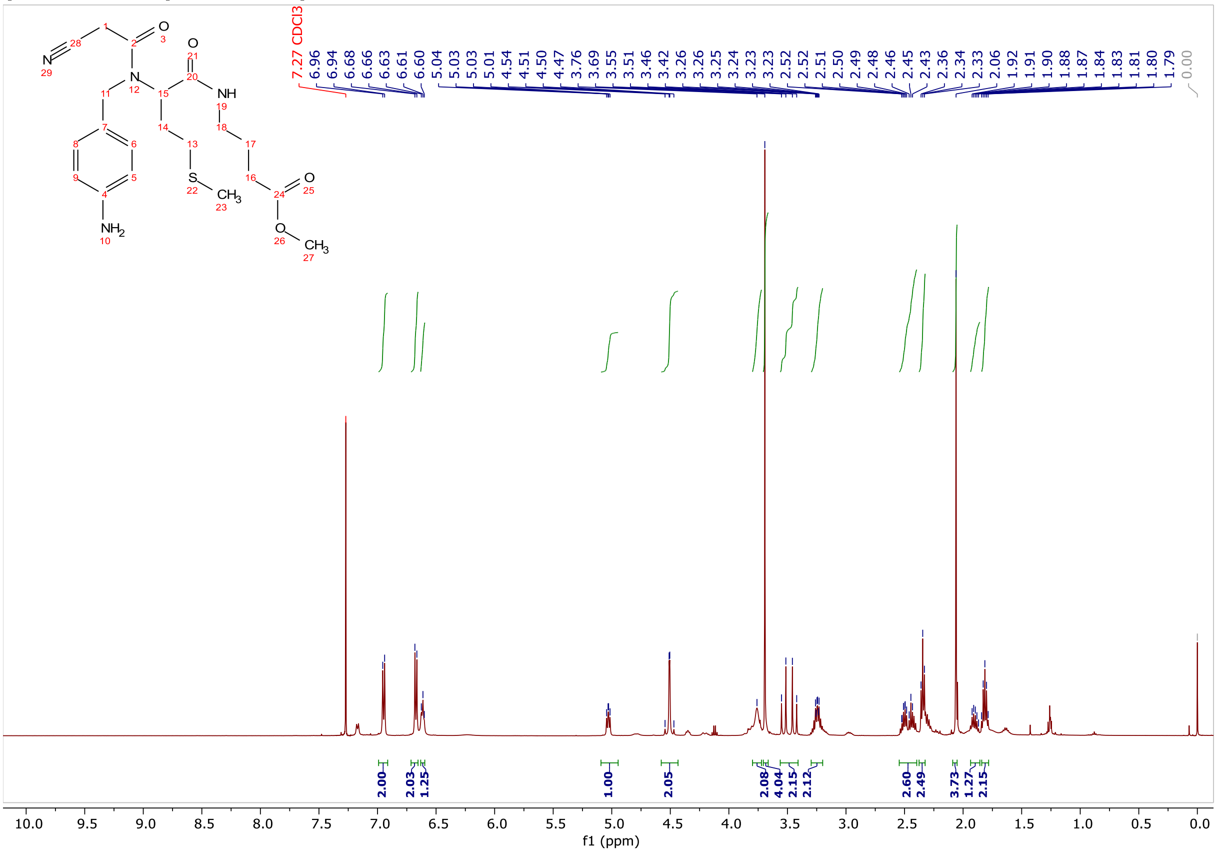

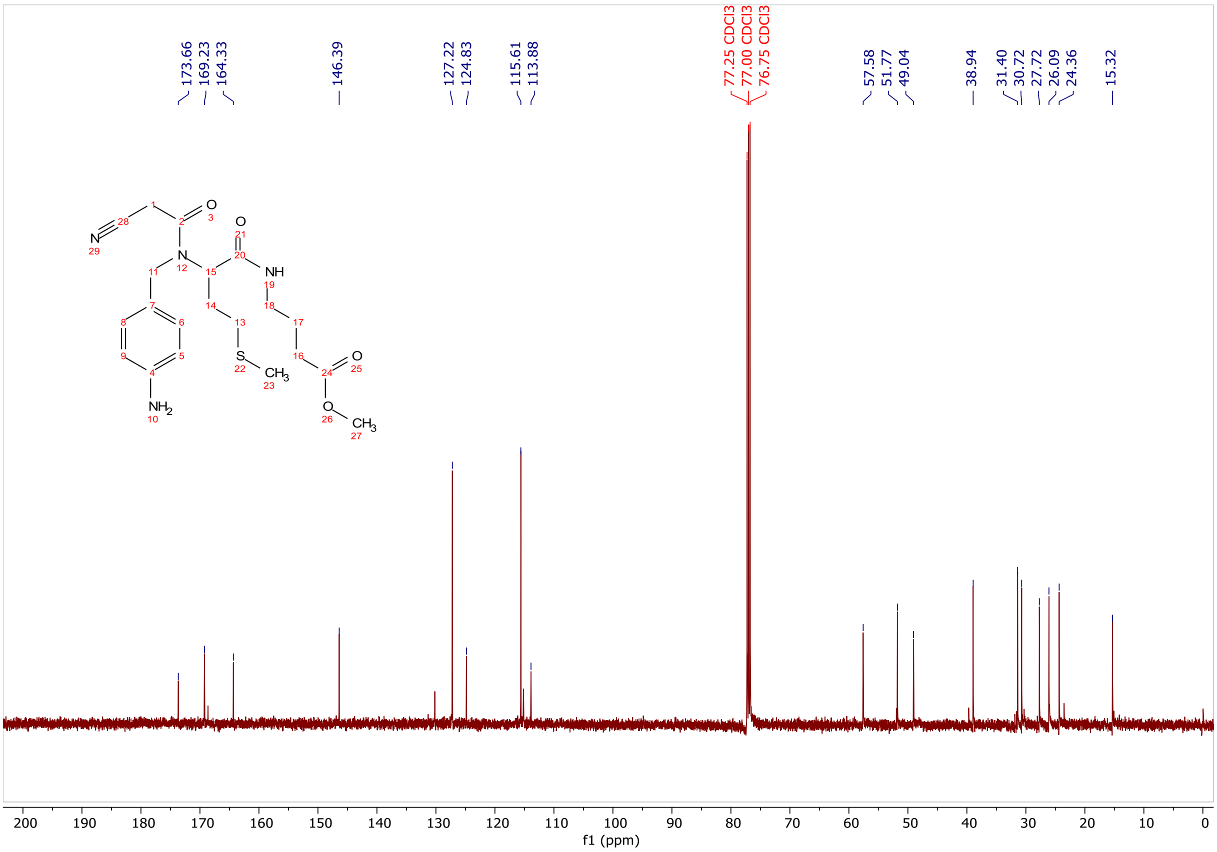

N-(2-(cyclohexylamino)-2-oxoethyl)-N-isobutyl-1-methylcyclohexane-1-carboxamide (Destination plate I, K9)

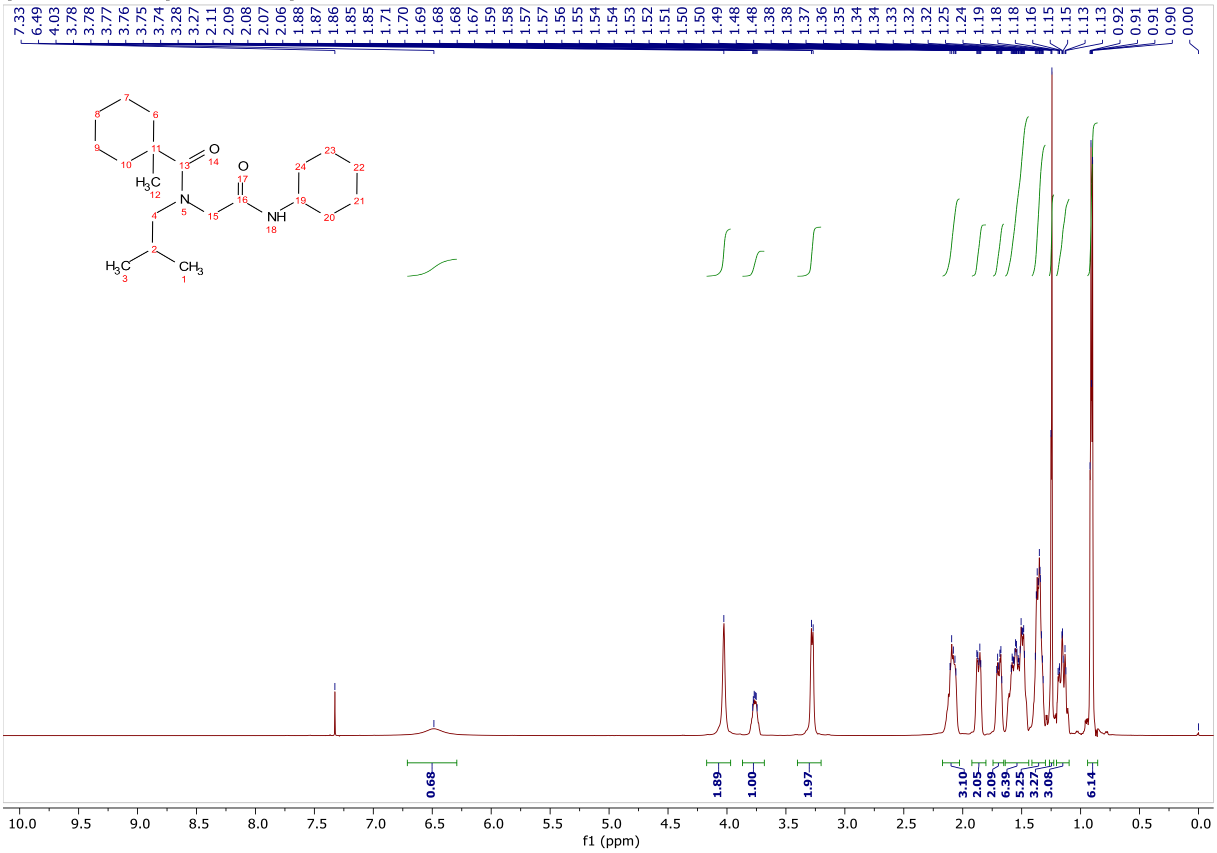

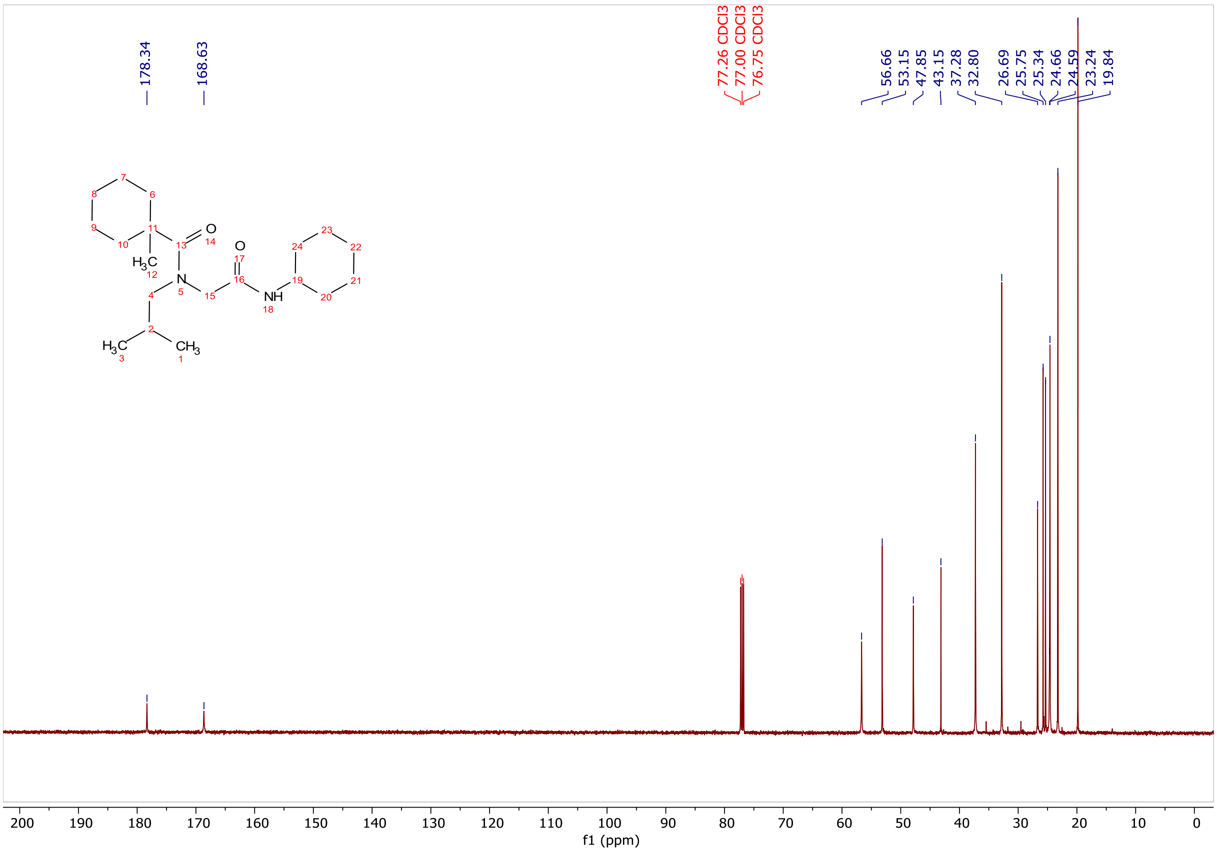

N-((1-cyclohexyl-1H-tetrazol-5-yl)(1H-indazol-6-yl)methyl)cyclopropanamine (Destination plate I, O17)

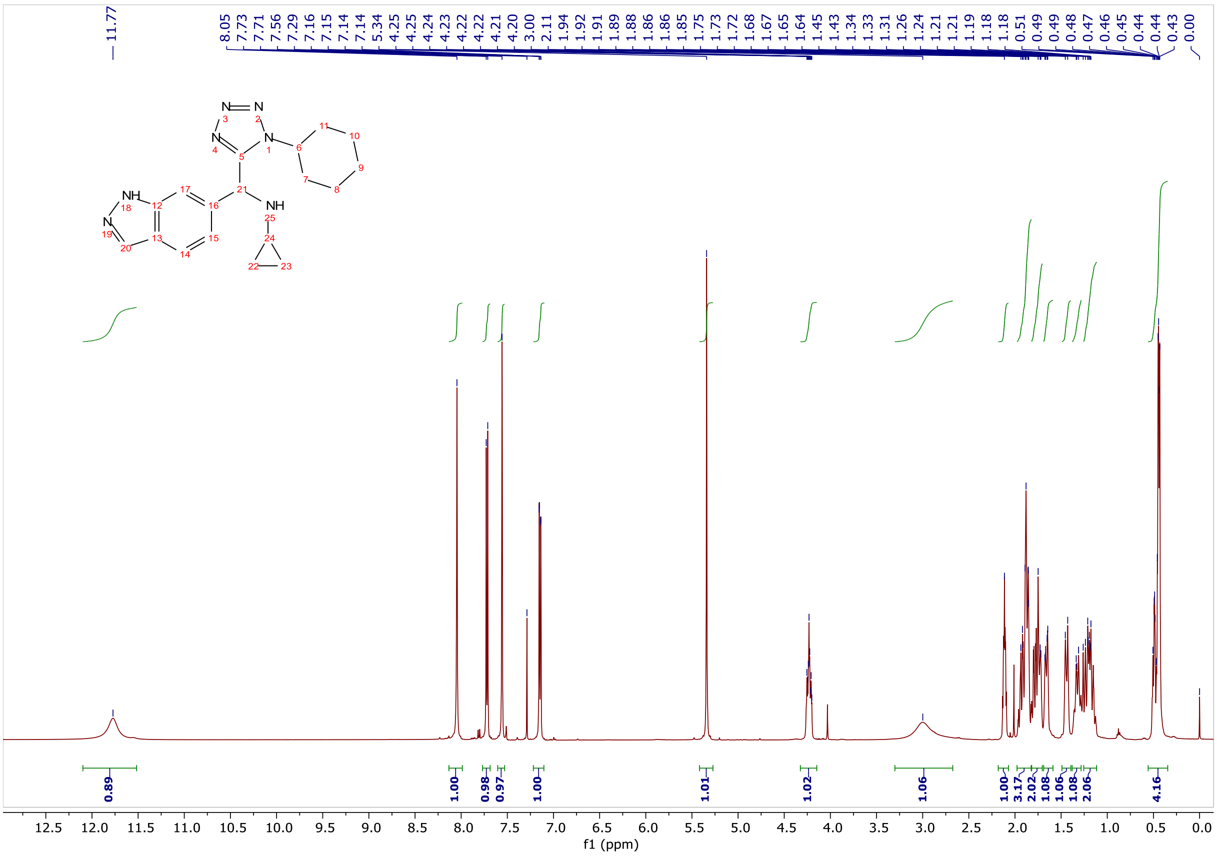

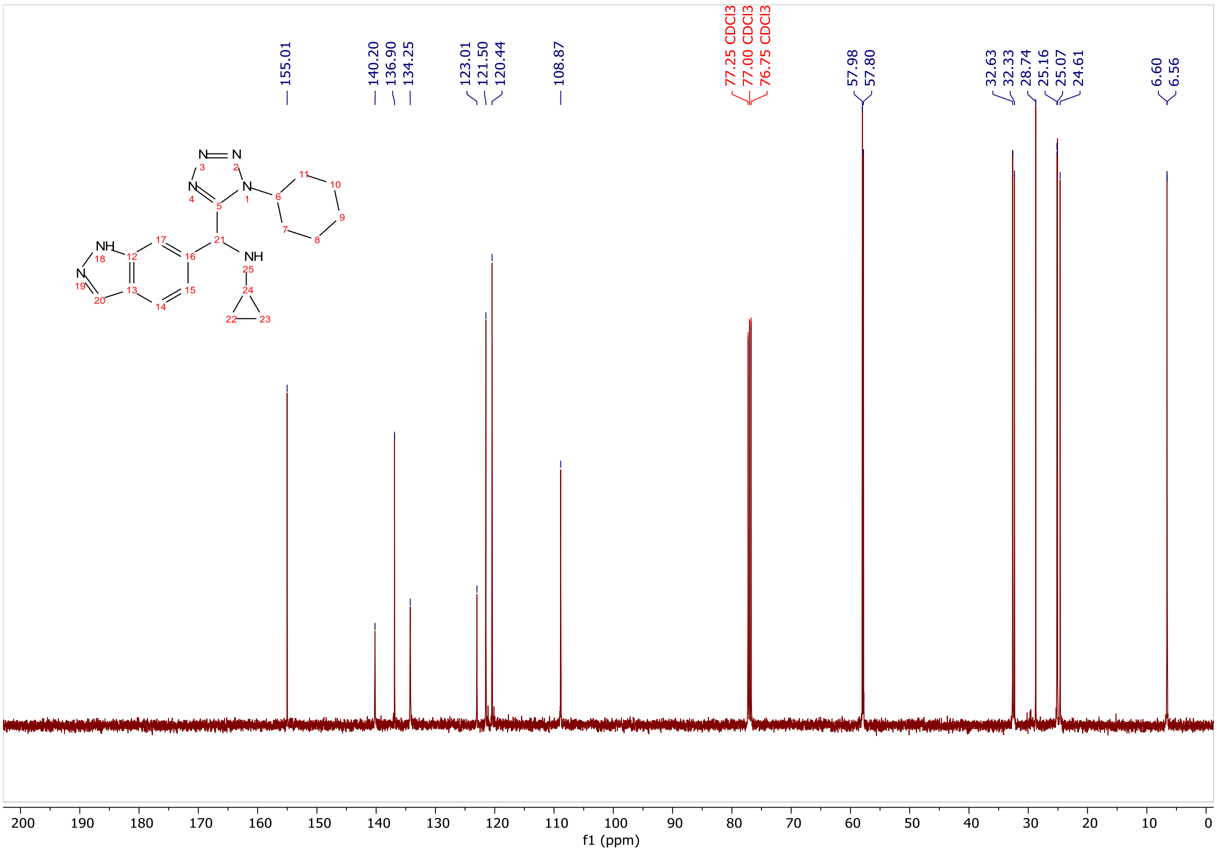

N-allyl-N-(2-methyl-1-(1-(thiophen-2-ylmethyl)-1H-tetrazol-5-yl)propyl)prop-2-en-1-amine (Destination plate I, P16)

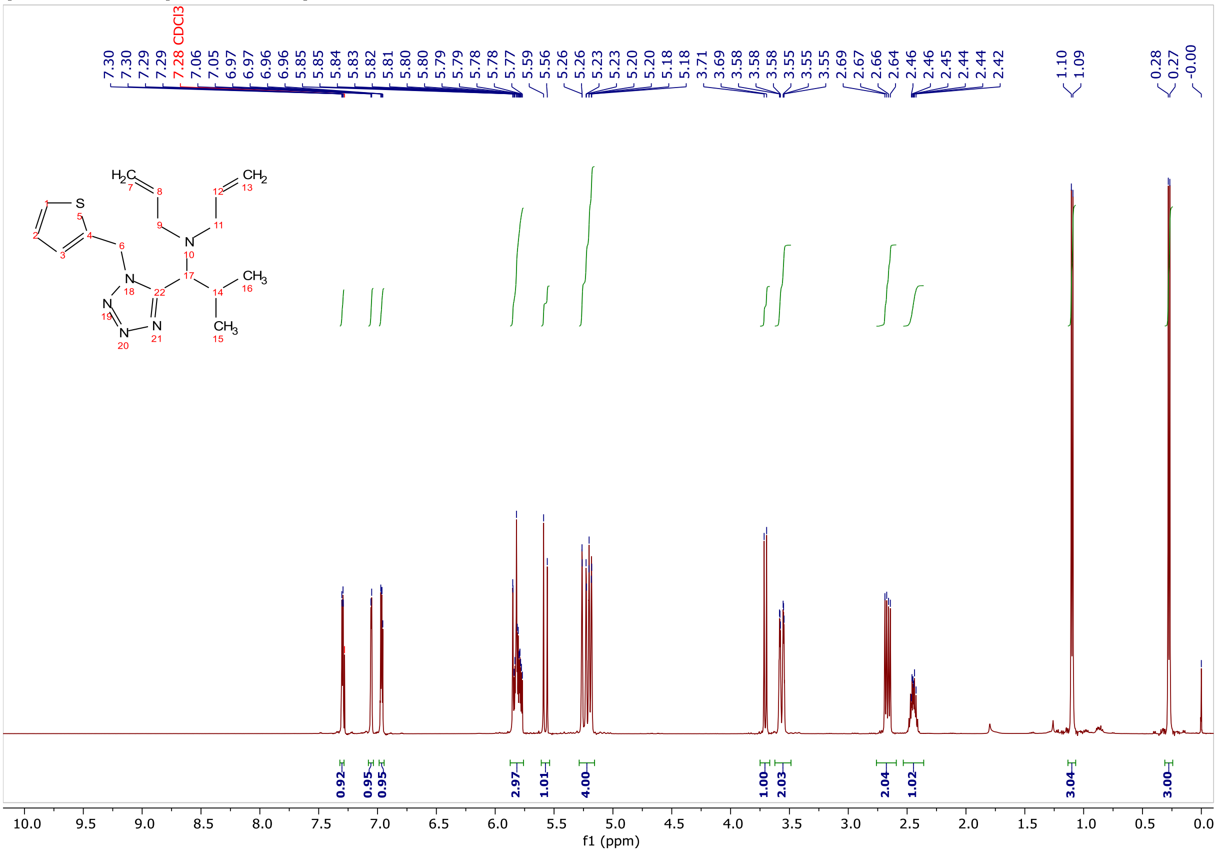

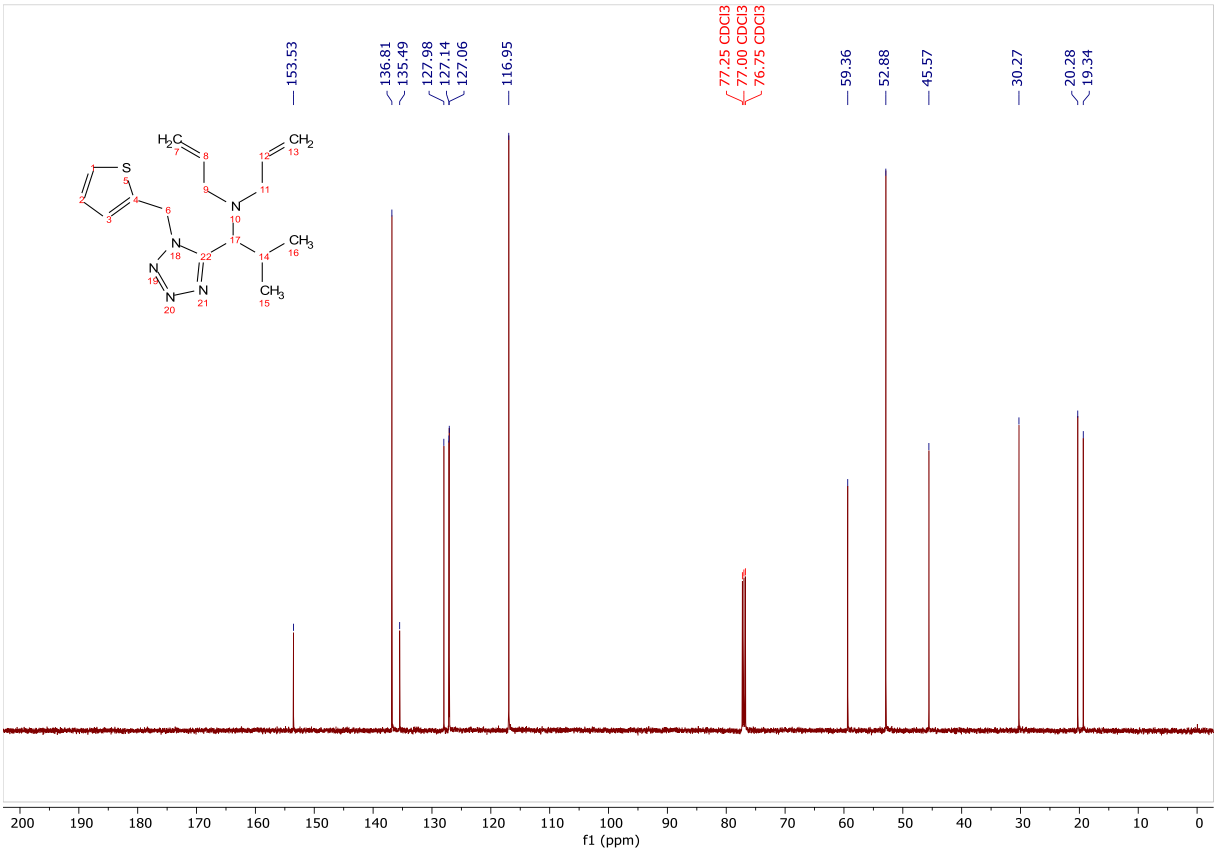

(7S,8aR)-7-Hydroxy-1-oxo-N-(thiophen-2-ylmethyl)hexahydro-1H-pyrrolo[2,1-c][1,4]oxazine-4-carboxamide (Destination plate II, E7)

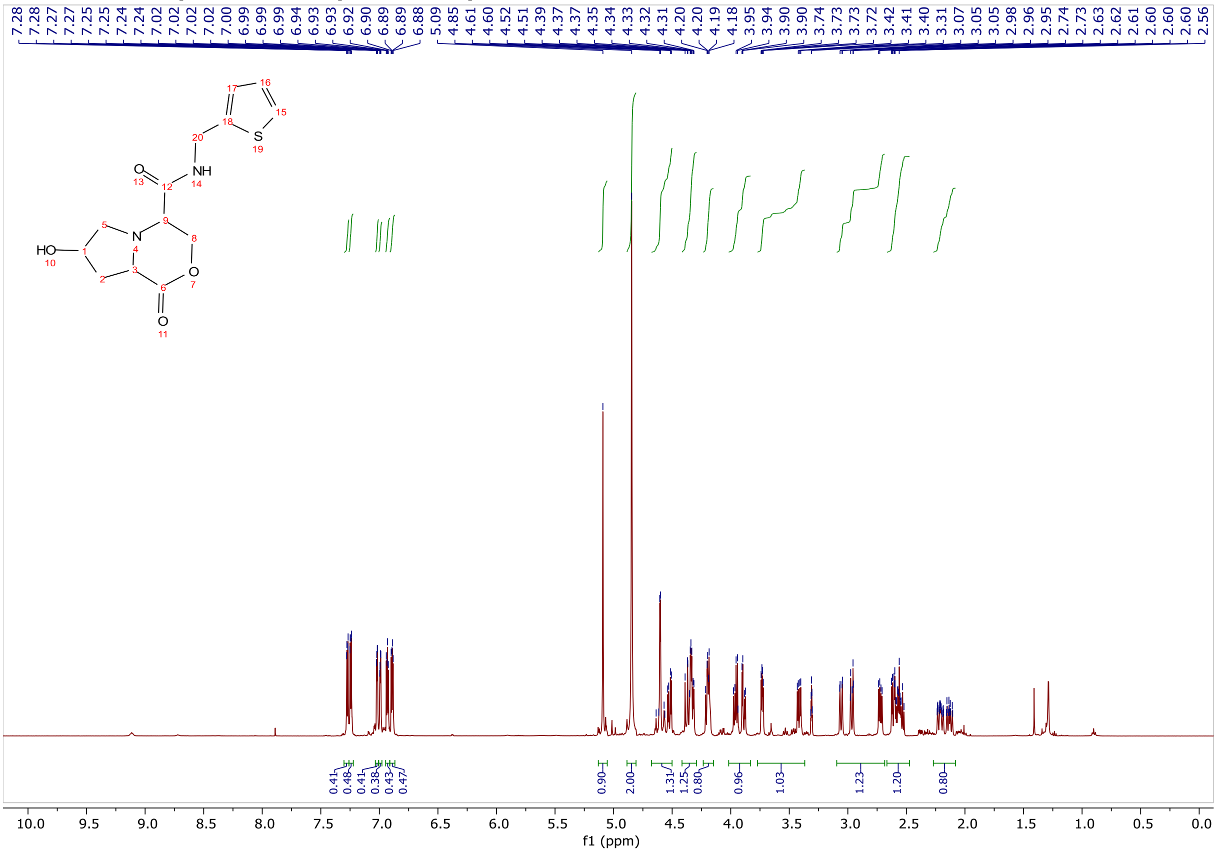

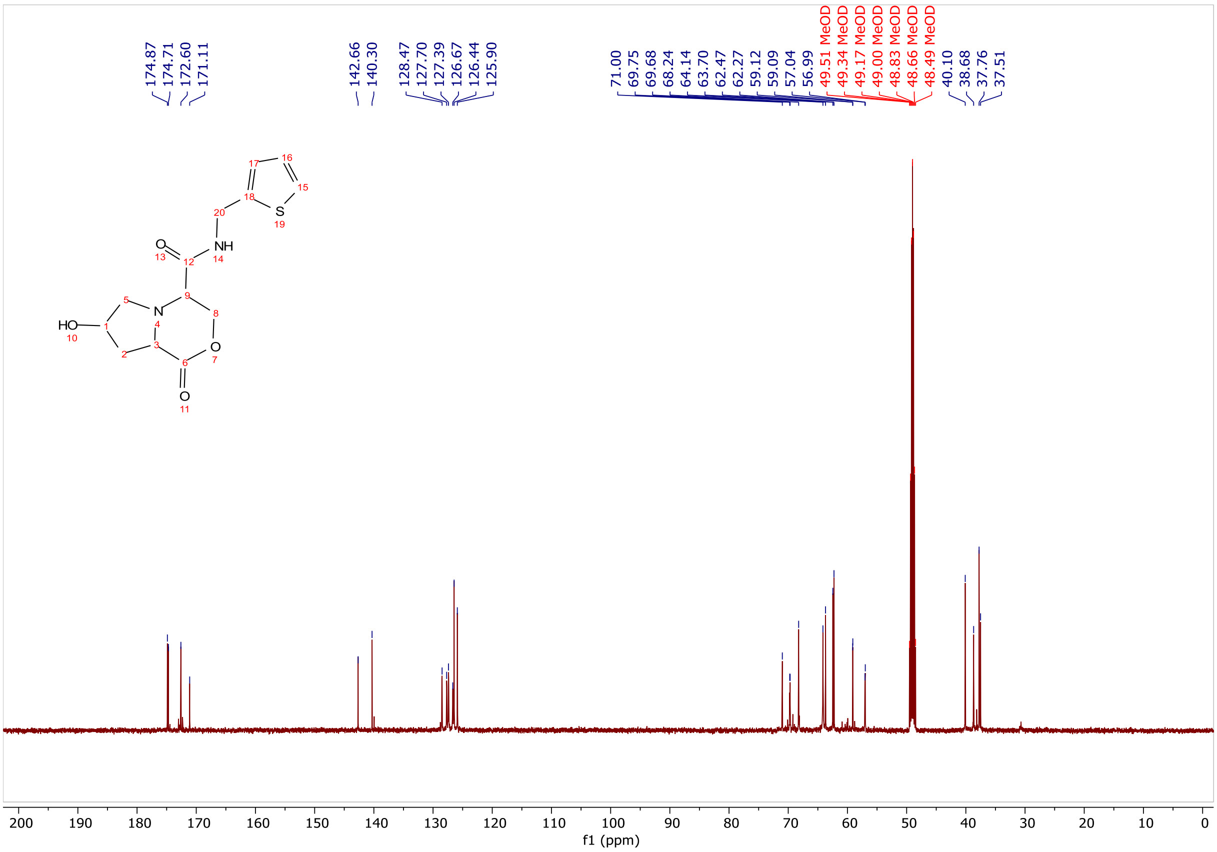

(7S,8aR)-N-([1,1'-biphenyl]-2-yl)-7-hydroxy-1-oxohexahydro-1H-pyrrolo[2,1-c][1,4]oxazine-4-carboxamide (Destination plate II, F9)

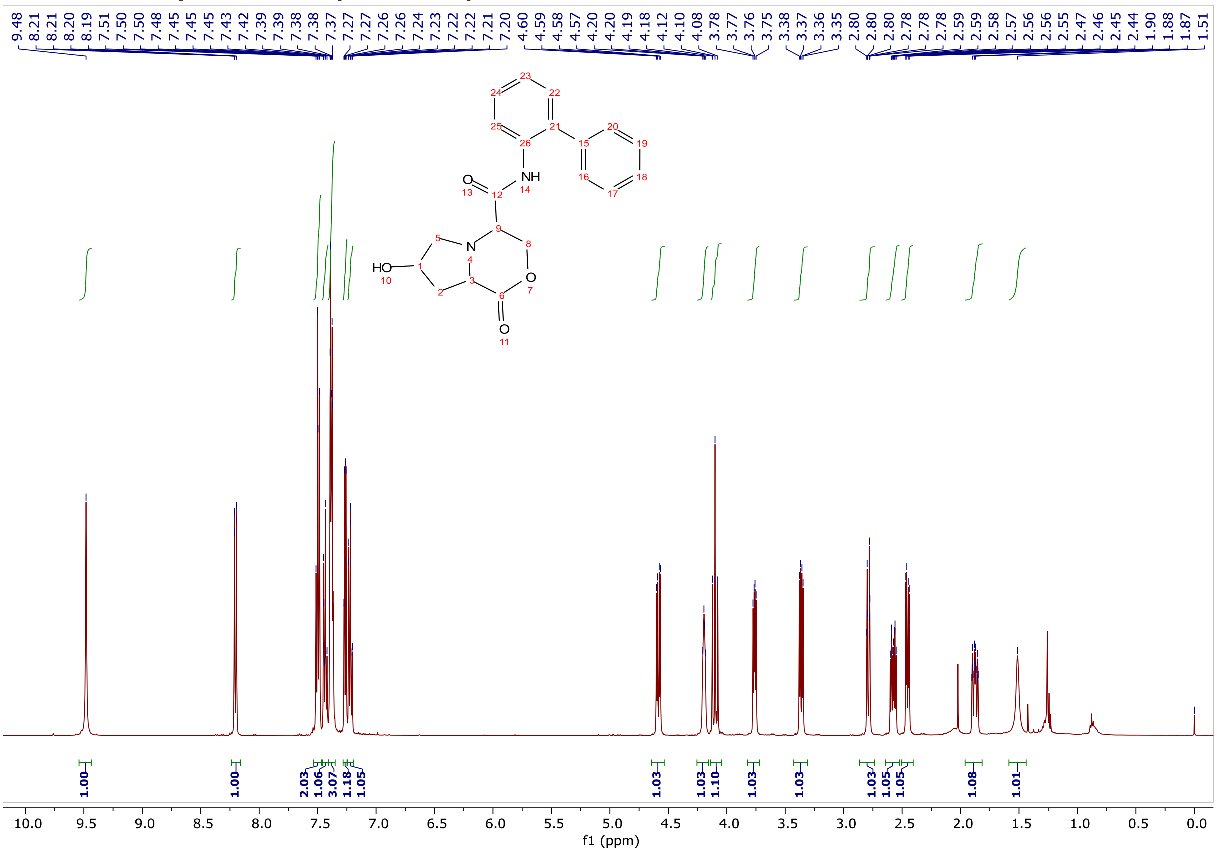

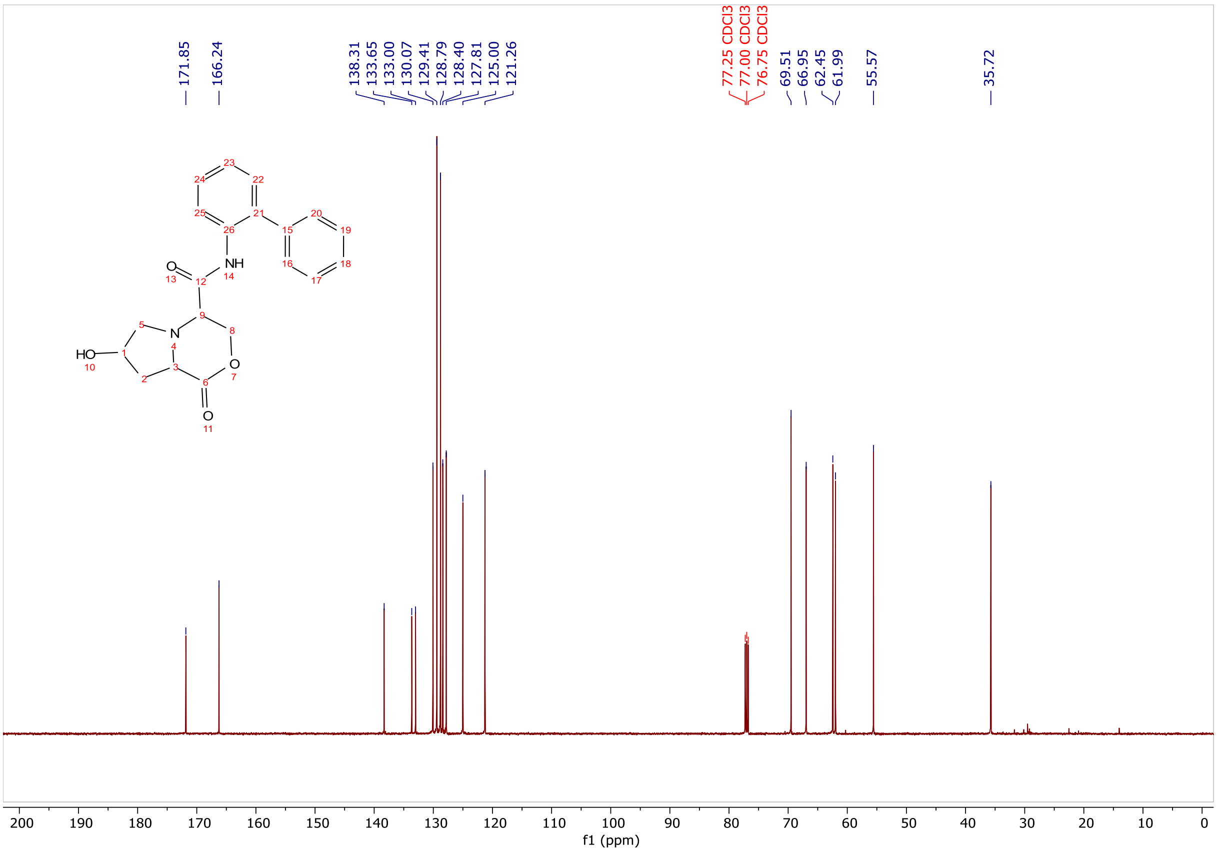

(Z)-1-(5-Hydroxypentyl)-5-(1-phenylethyl)-4-((thiophen-2-ylmethyl)imino)imidazolidin-2-one (Destination plate II, J6)

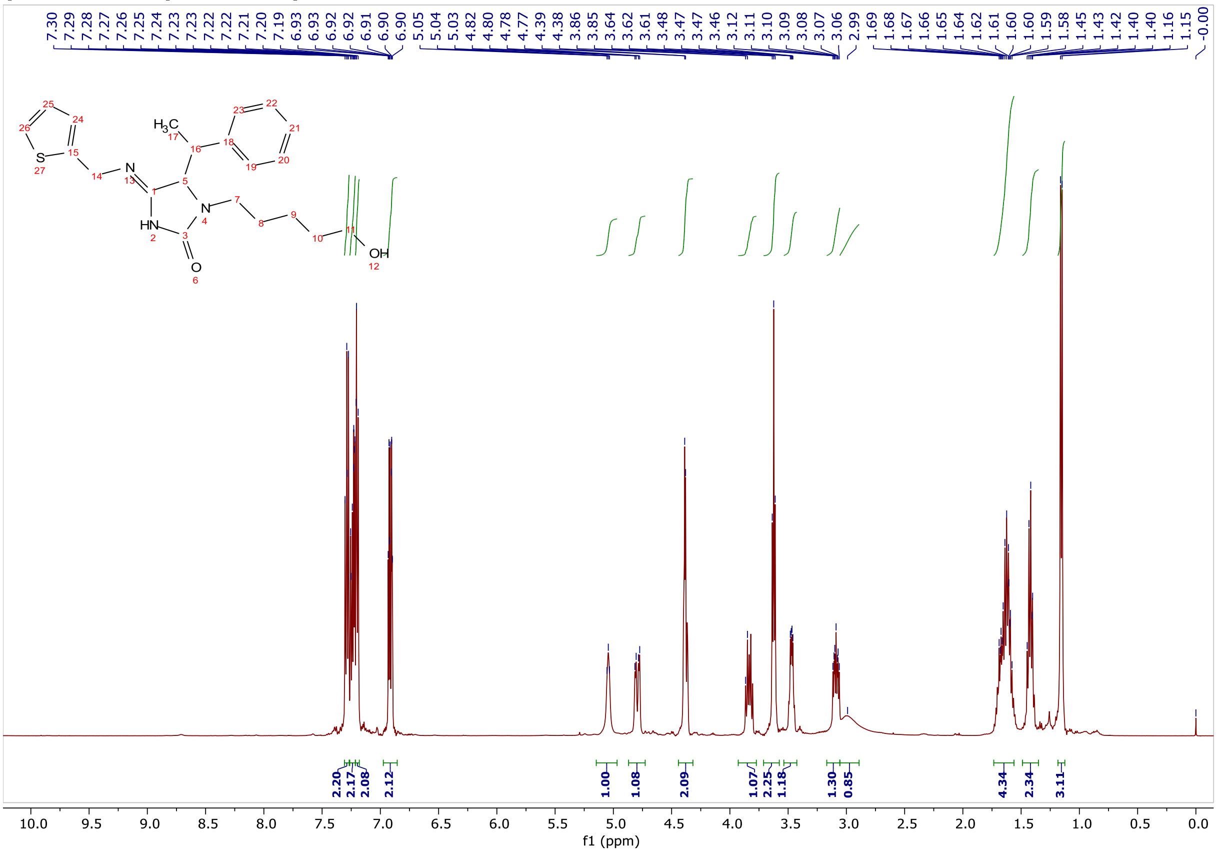

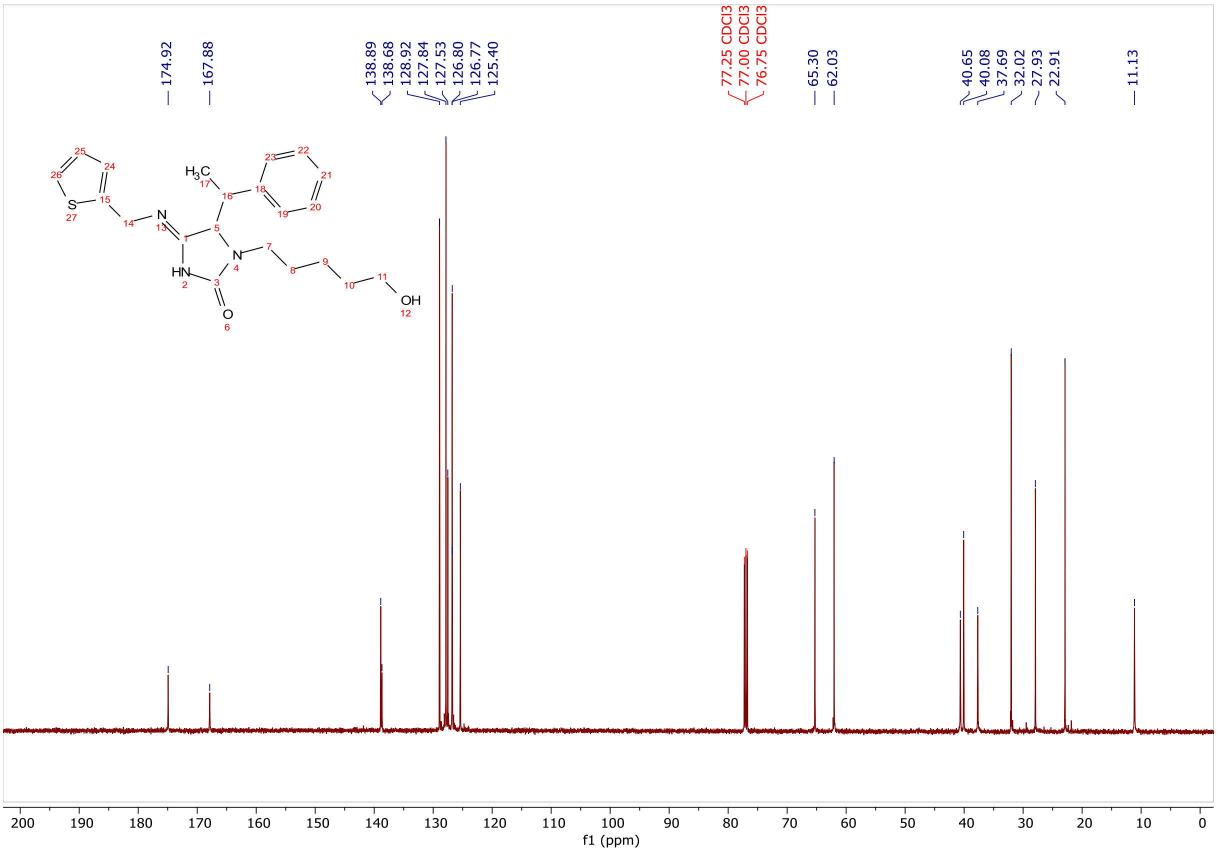

2-(Naphthalen-1-yl)-2-((2-oxotetrahydrofuran-3-yl)amino)-N-(2,4,4-trimethylpentan-2-yl)acetamide (Destination plate II, M6)

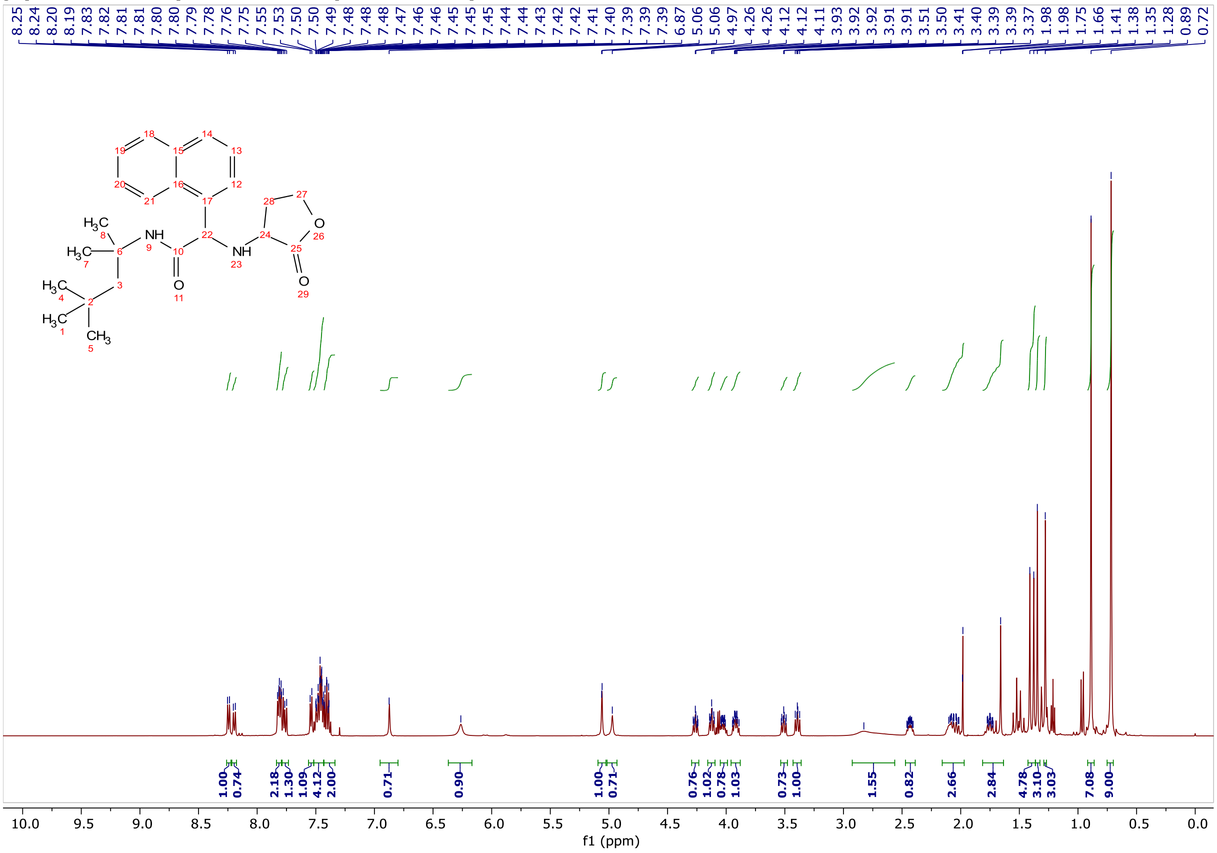

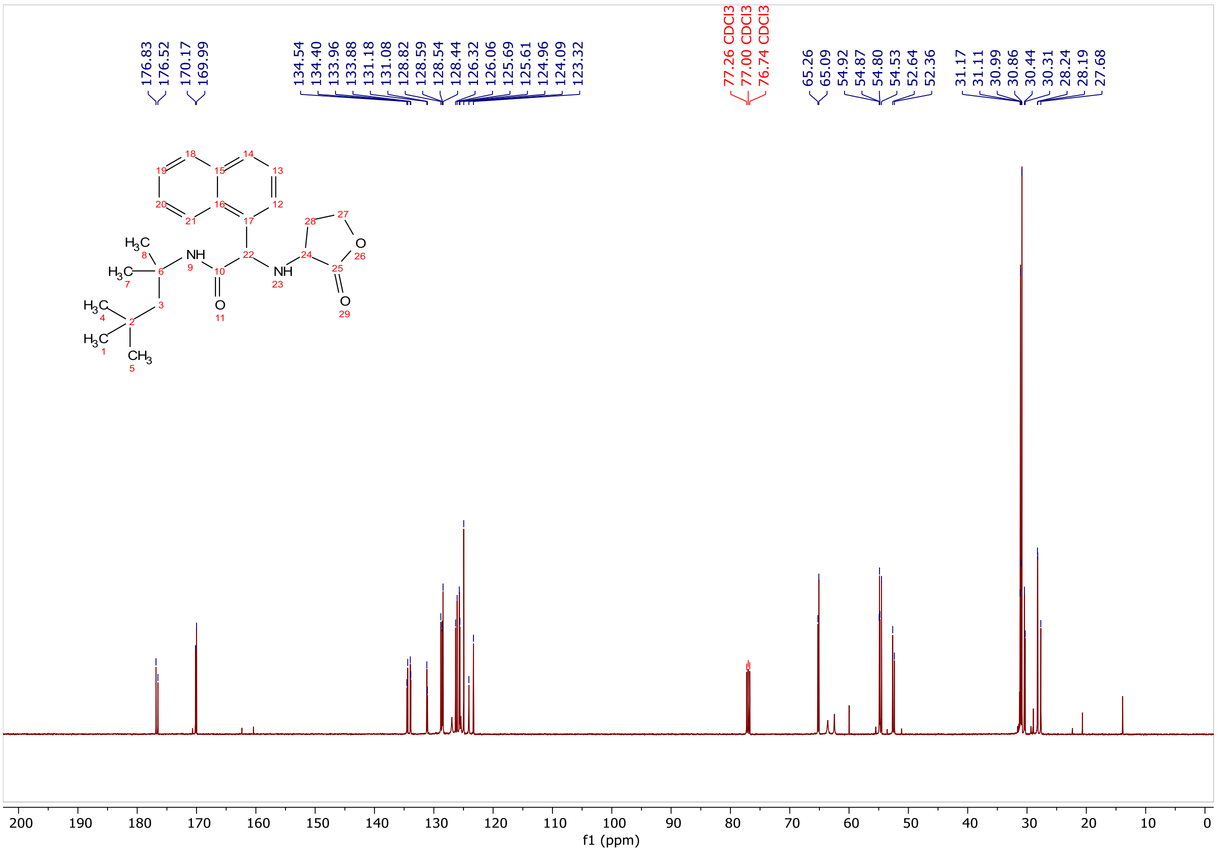

N-(4-chlorophenyl)-2-(naphthalen-1-yl)-2-(((S)-2-oxoazepan-3-yl)amino)acetamide (Destination plate II, O8)

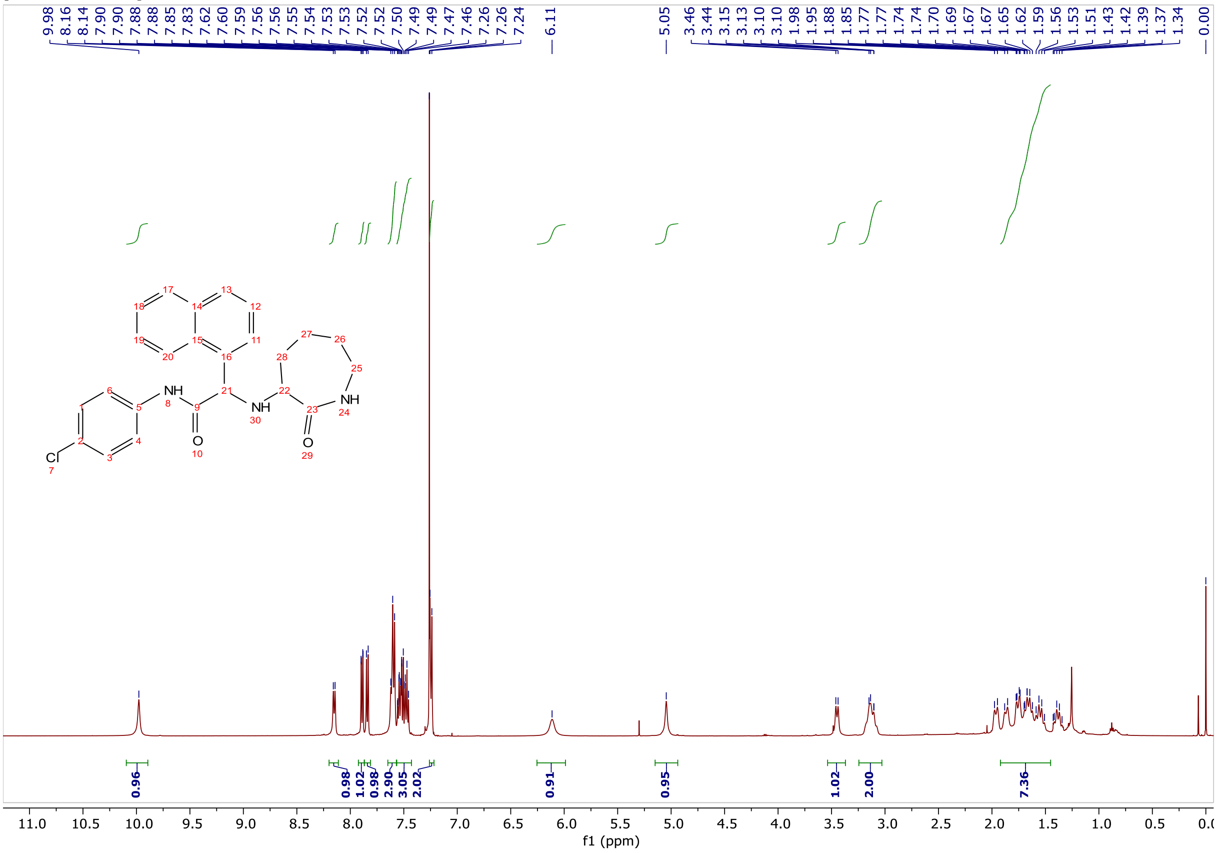

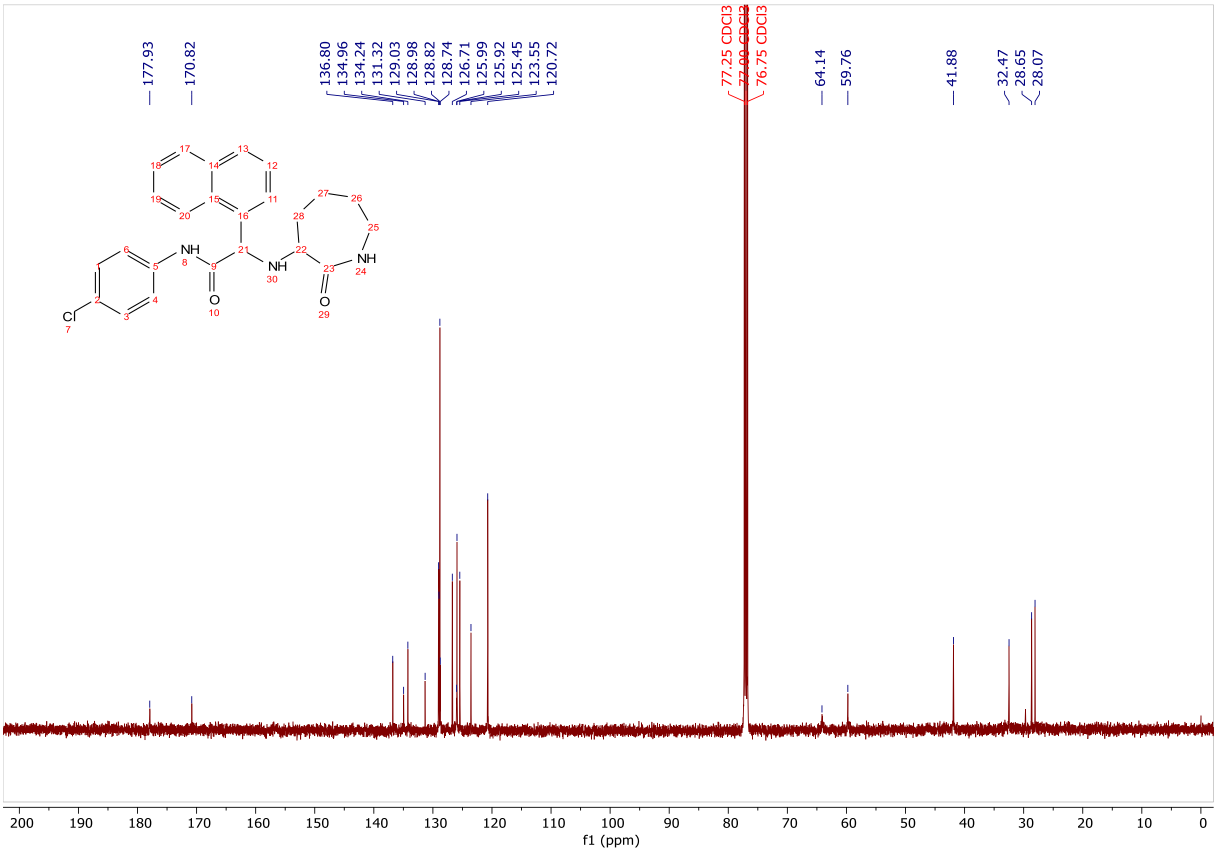

4-((4-(4-Fluorophenyl)-5-(2-(methylthio)ethyl)-1H-imidazol-1-yl)methyl)aniline (Destination plate III, A8)

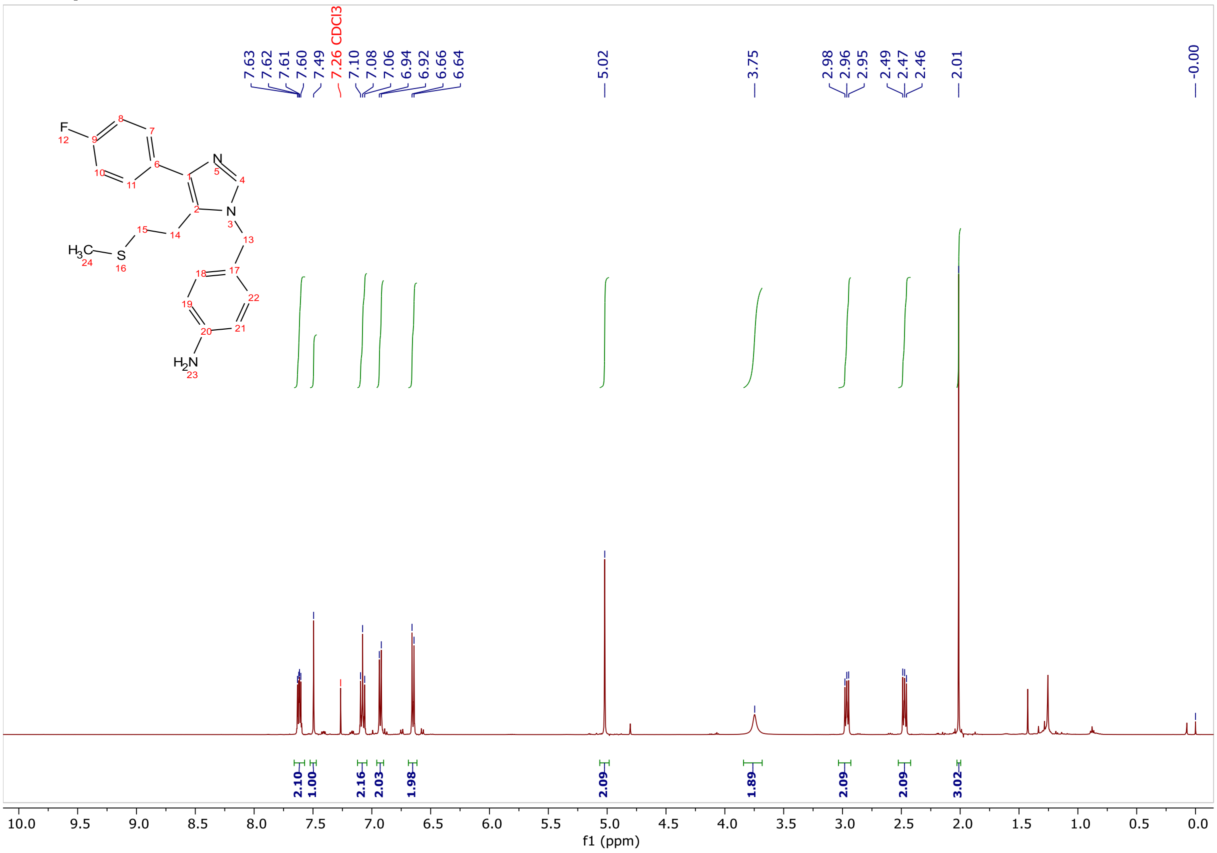

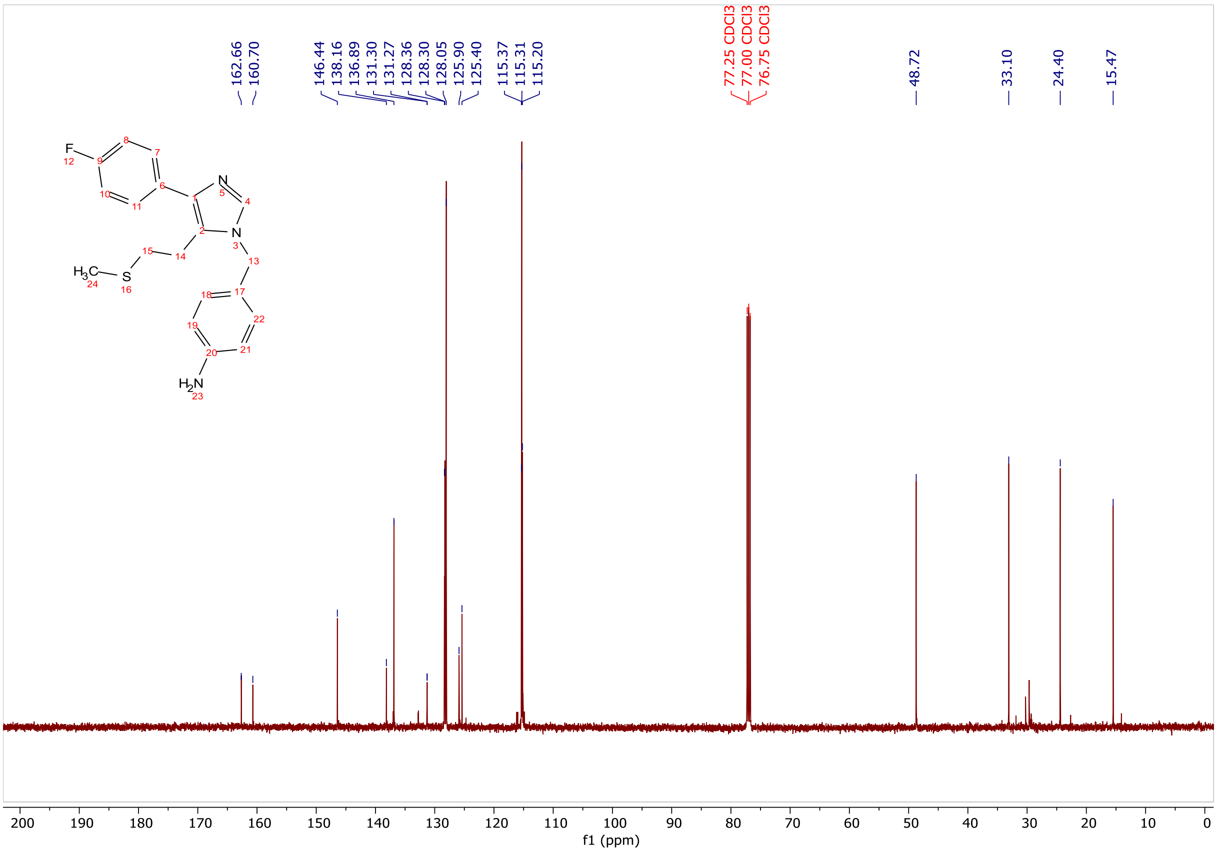

2-(5-(3,4-Dimethoxyphenyl)-4-(4-fluorophenyl)-1H-imidazol-1-yl)benzoic acid (Destination plate III, B18)

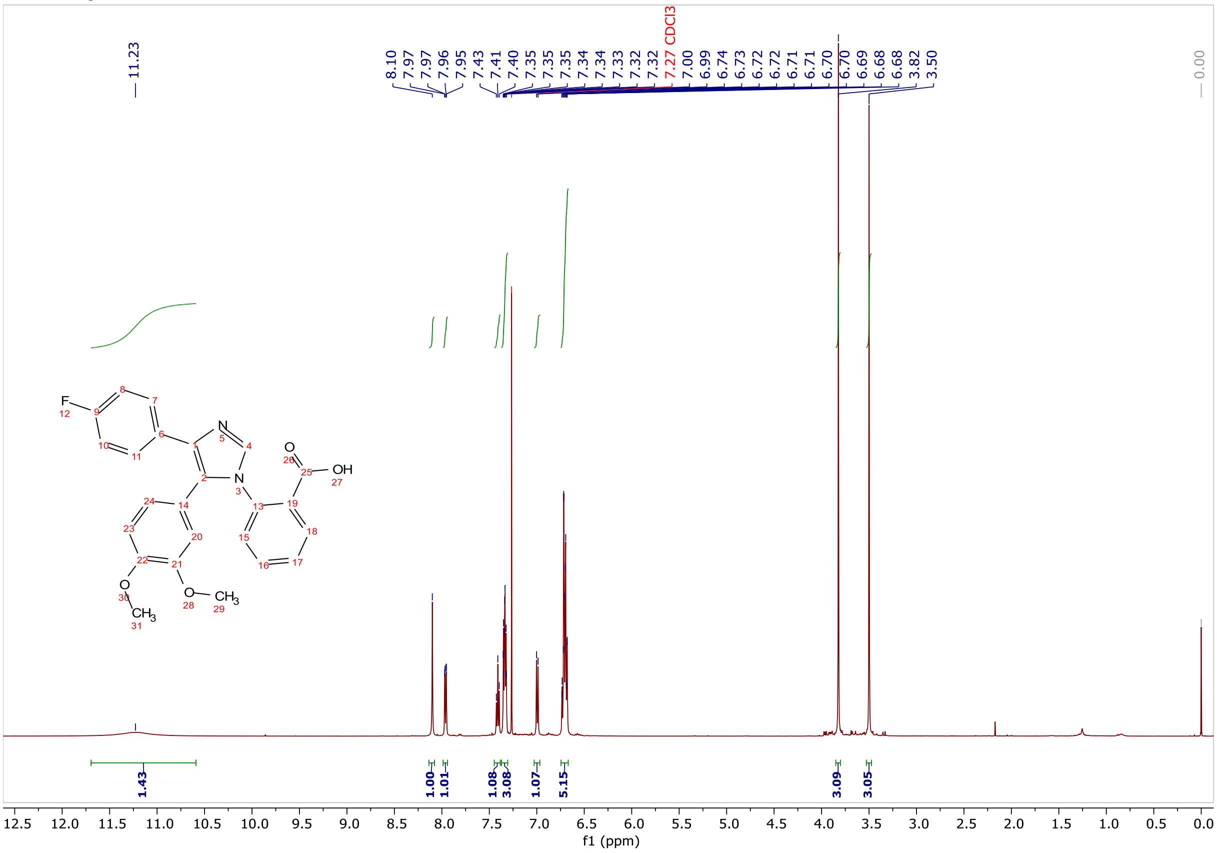

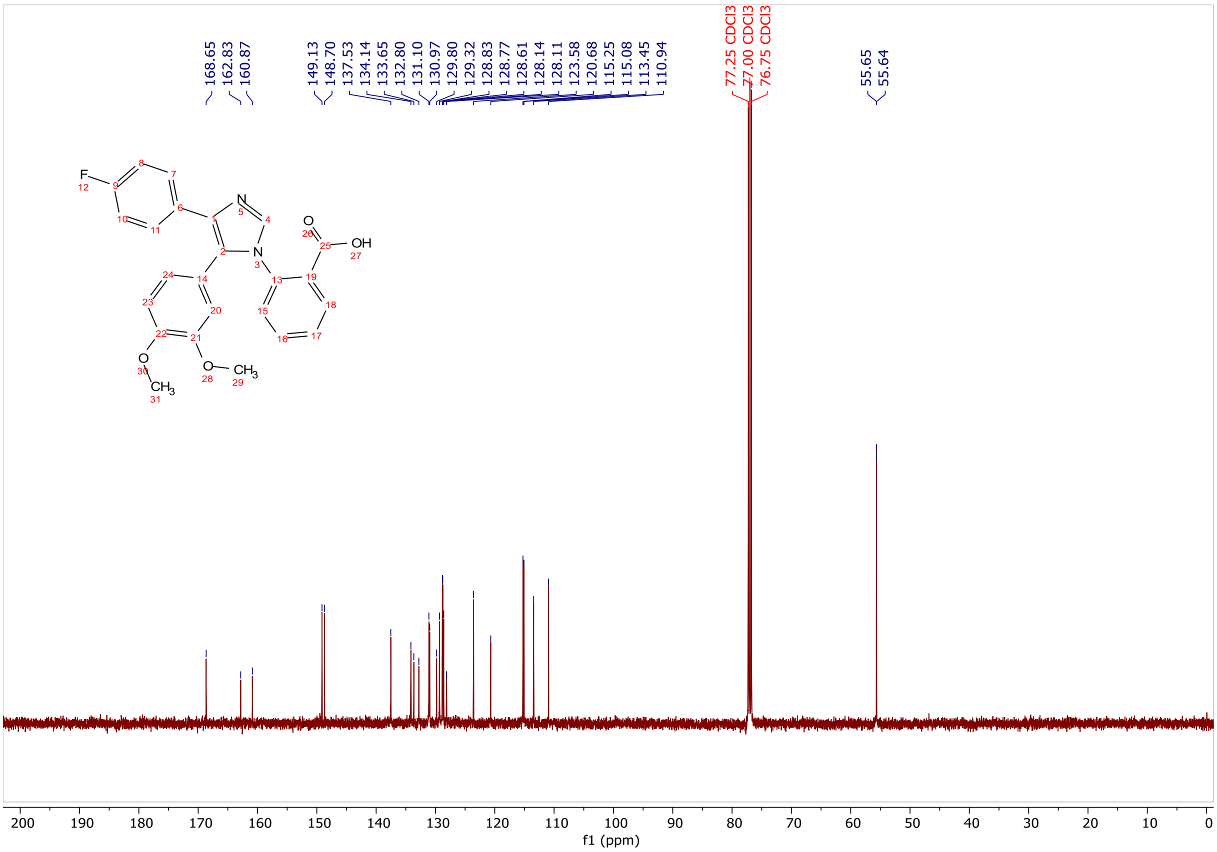

5-(4-(4-Fluorophenyl)-5-phenethyl-1H-imidazol-1-yl)pentan-1-ol

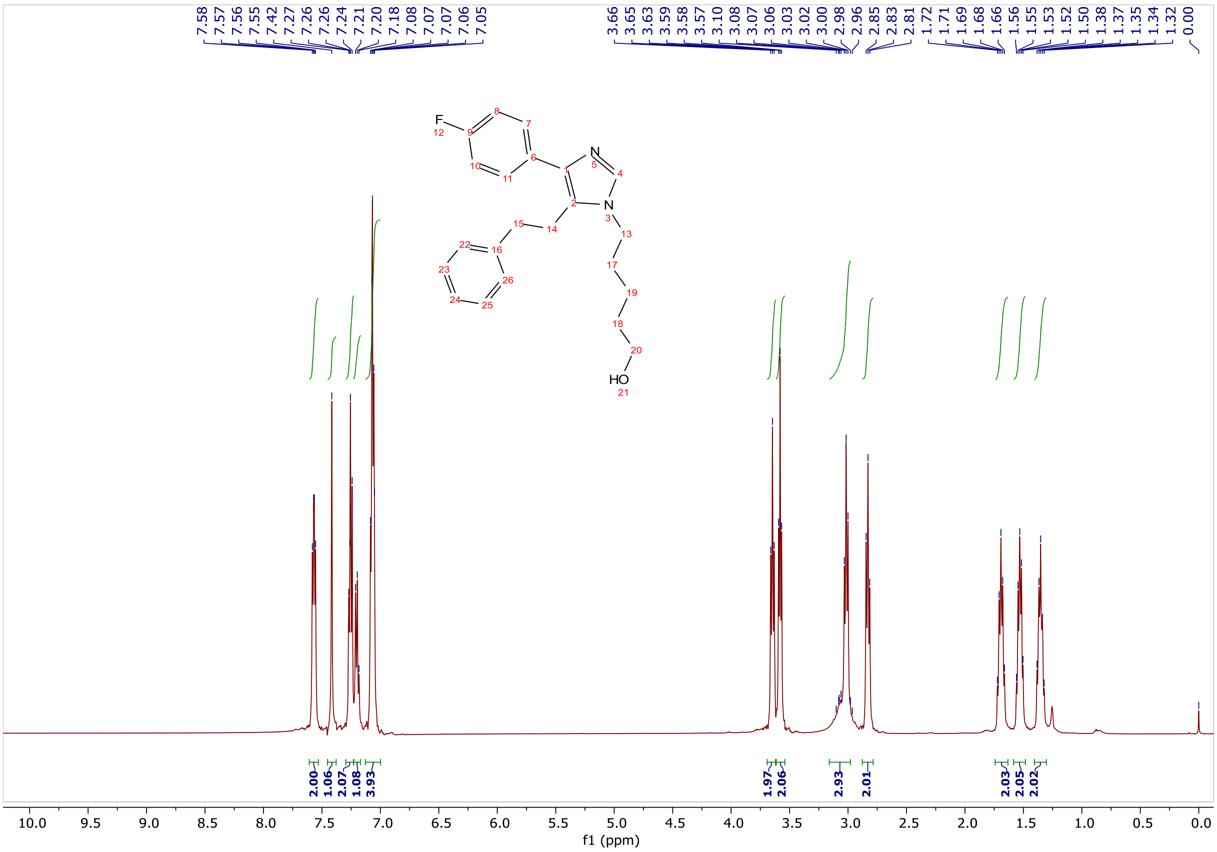

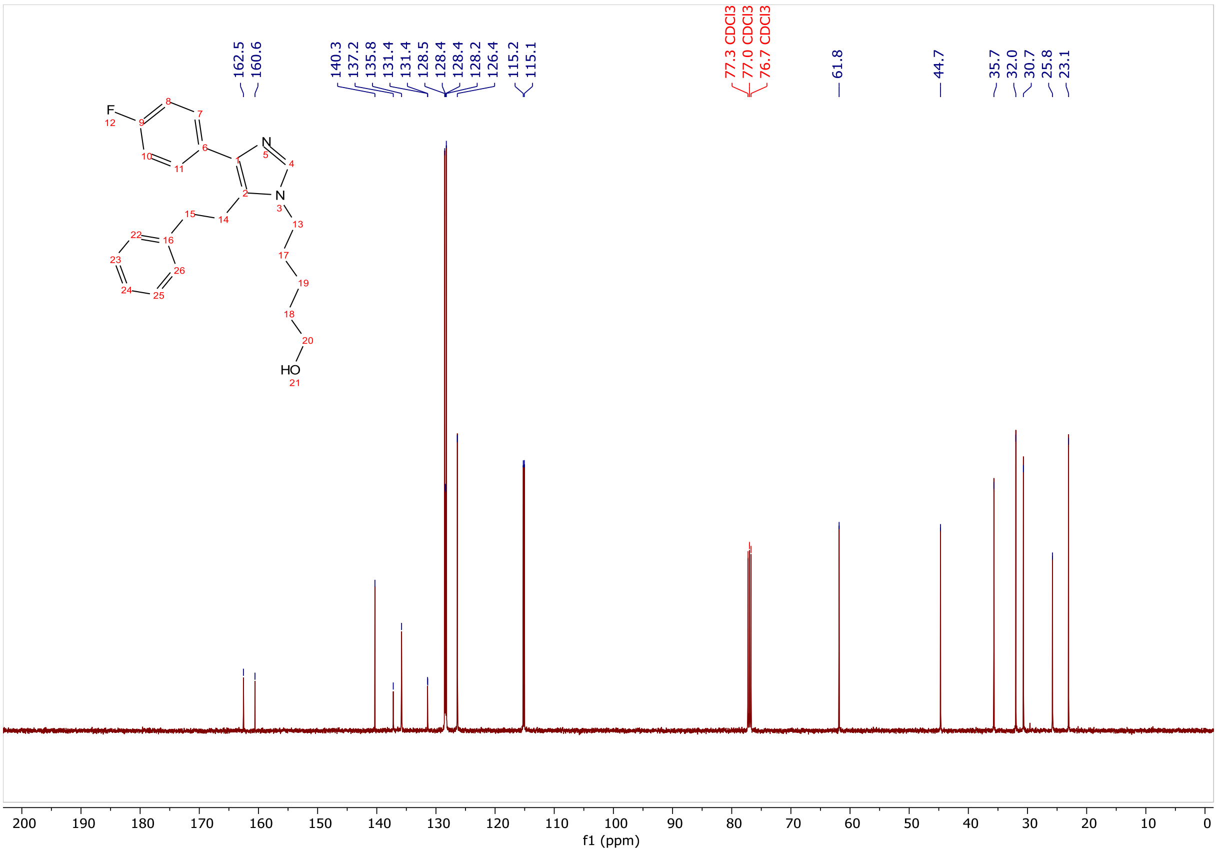

N-(4-chlorobenzyl)-2-((R)-2-(4-(4-methoxyphenyl)piperazine-1-carbonyl)pyrrolidin-1-yl)-2-(pyridin-3-yl)acetamide (Destination plate IV, A4)

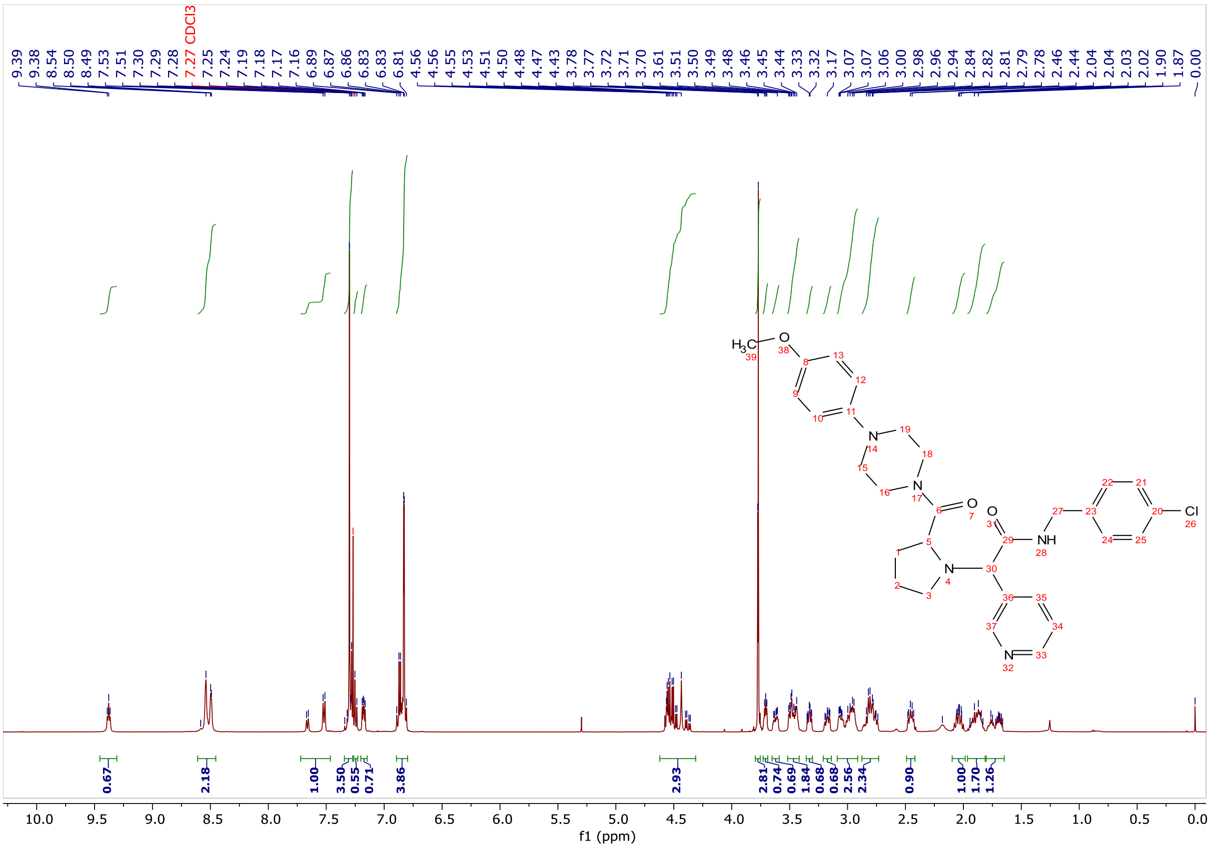

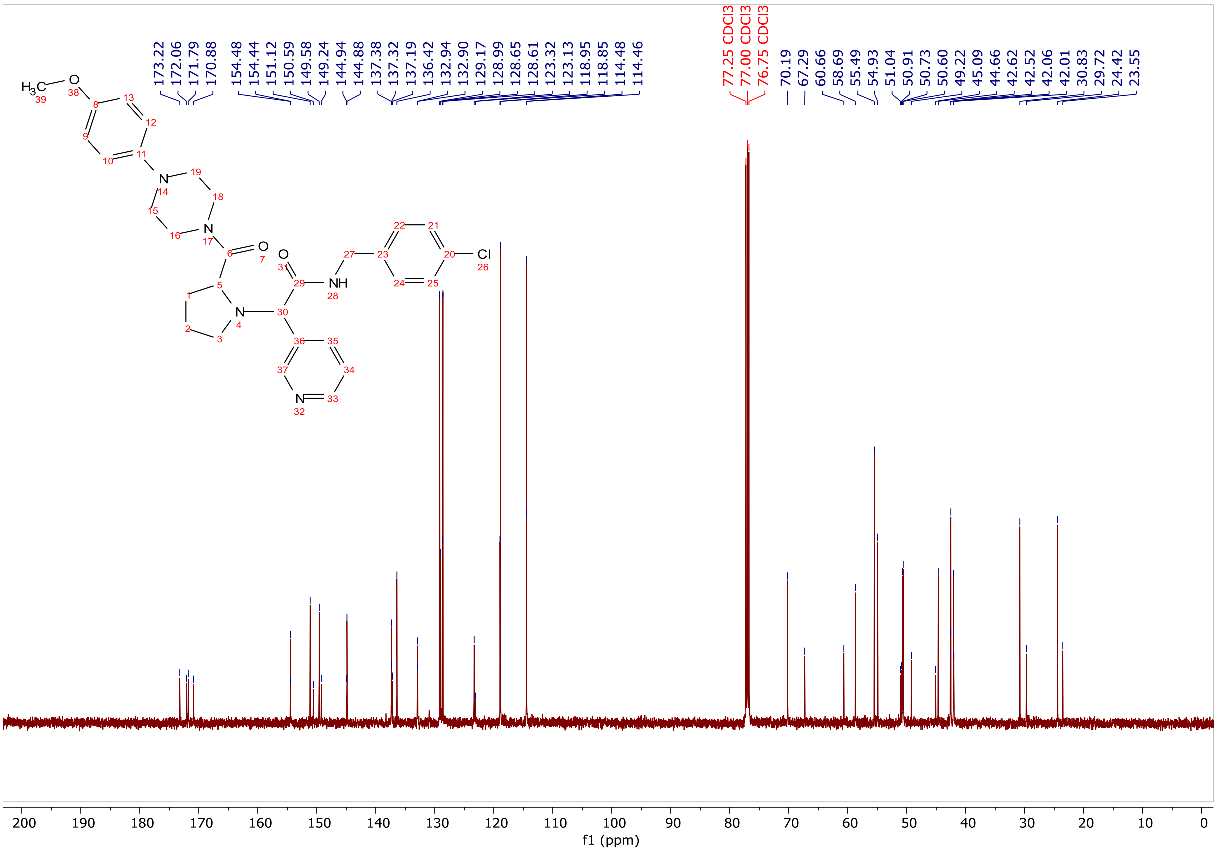

(2S)-N-(3-chloro-4-fluorophenyl)-1-(2-((4-fluorophenethyl)amino)-1-(4-methoxyphenyl)-2-oxoethyl)pyrrolidine-2-carboxamide (Destination plate IV, A17)

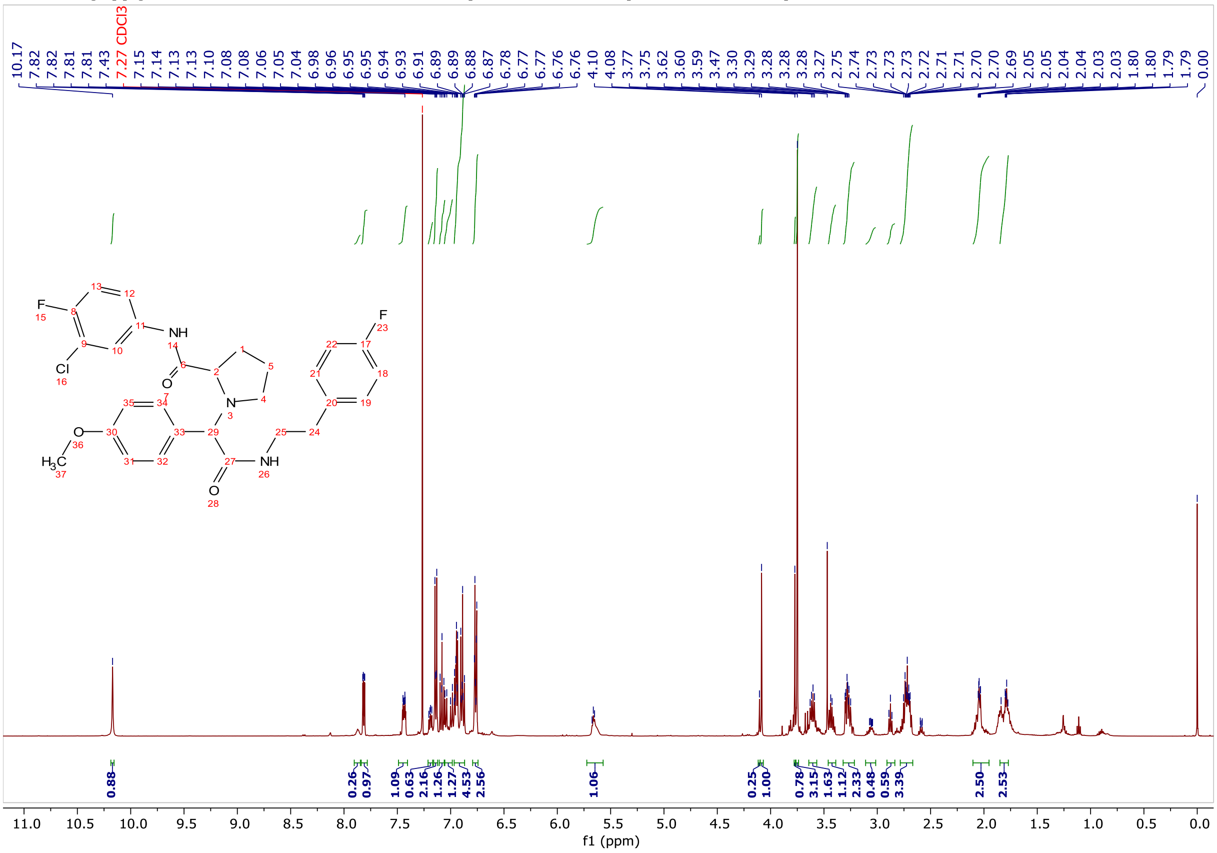

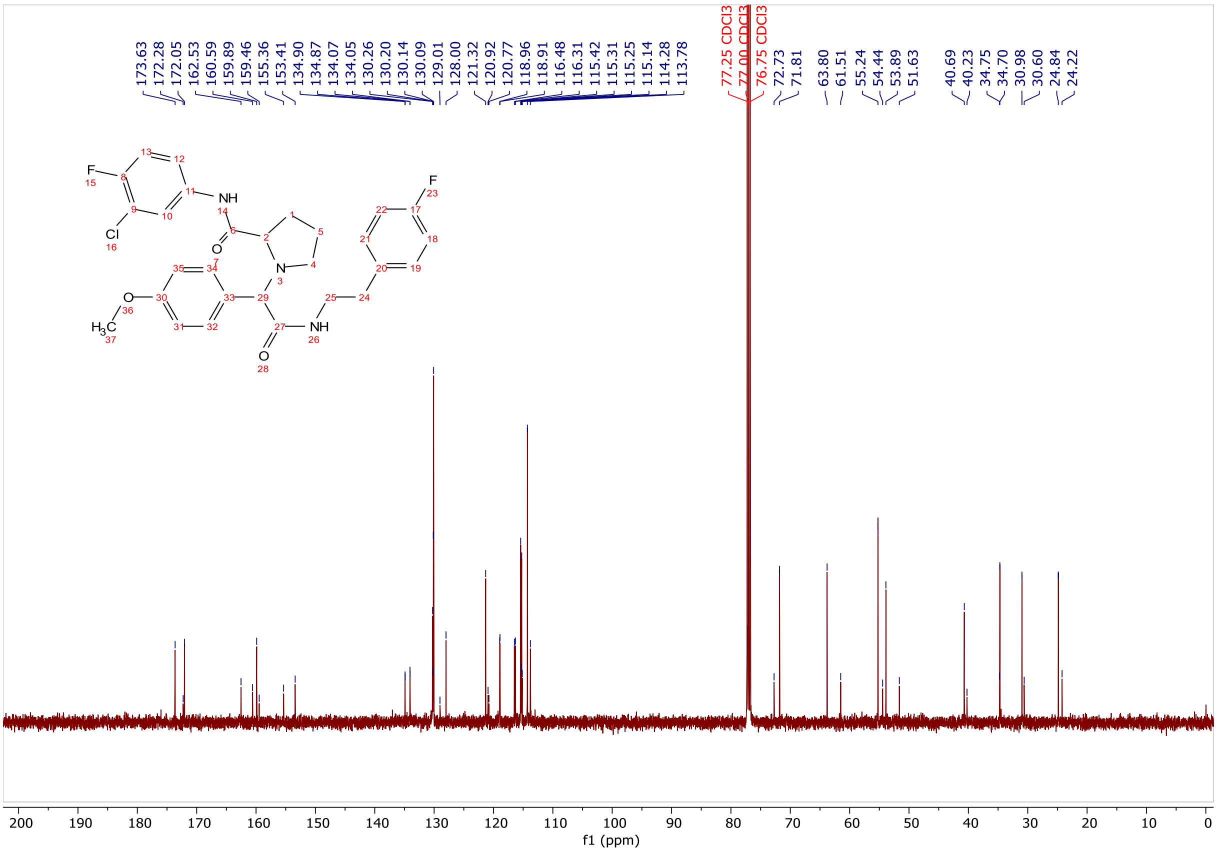

N-cyclohexyl-2-((R)-2-(indoline-1-carbonyl)pyrrolidin-1-yl)butanamide (Destination plate IV, B12, diastereomer A**)**

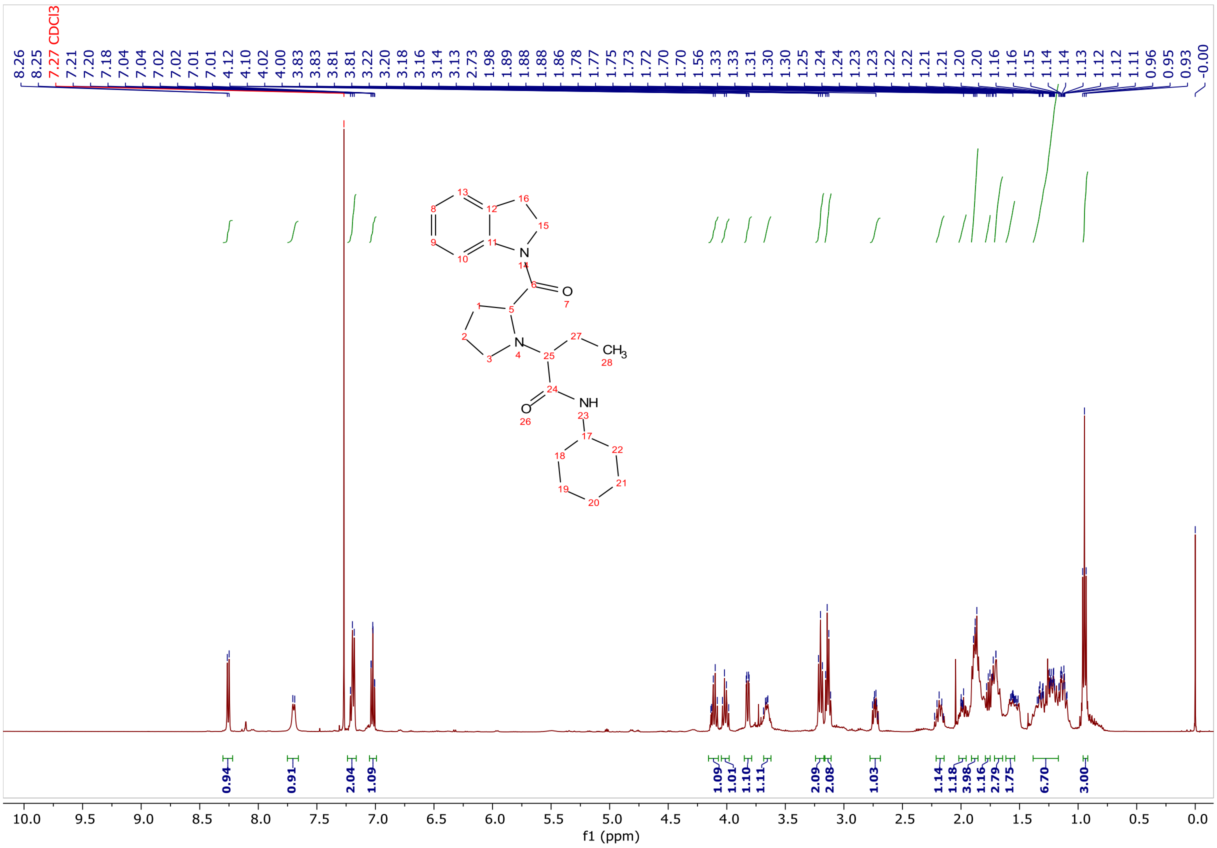

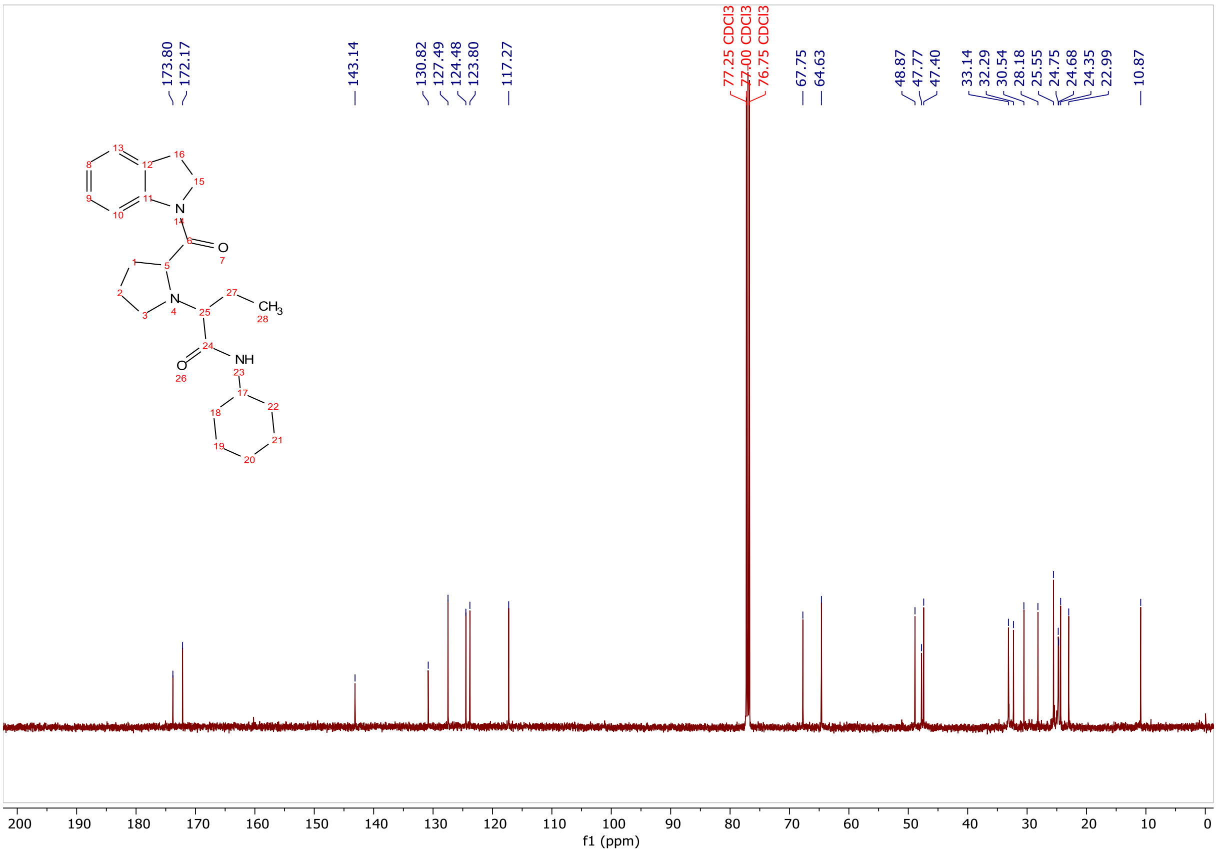

N-cyclohexyl-2-((R)-2-(indoline-1-carbonyl)pyrrolidin-1-yl)butanamide (Destination plate IV, B12, diastereomer B**)**

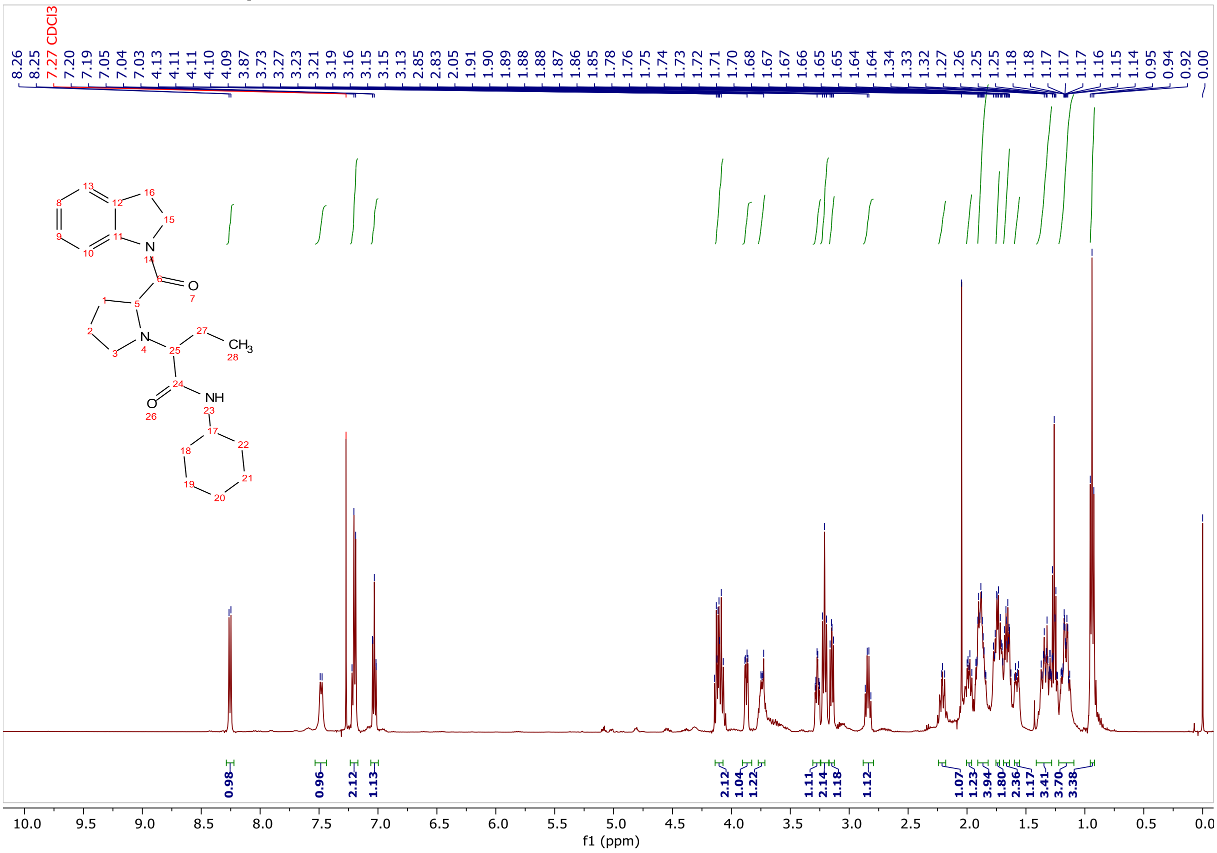

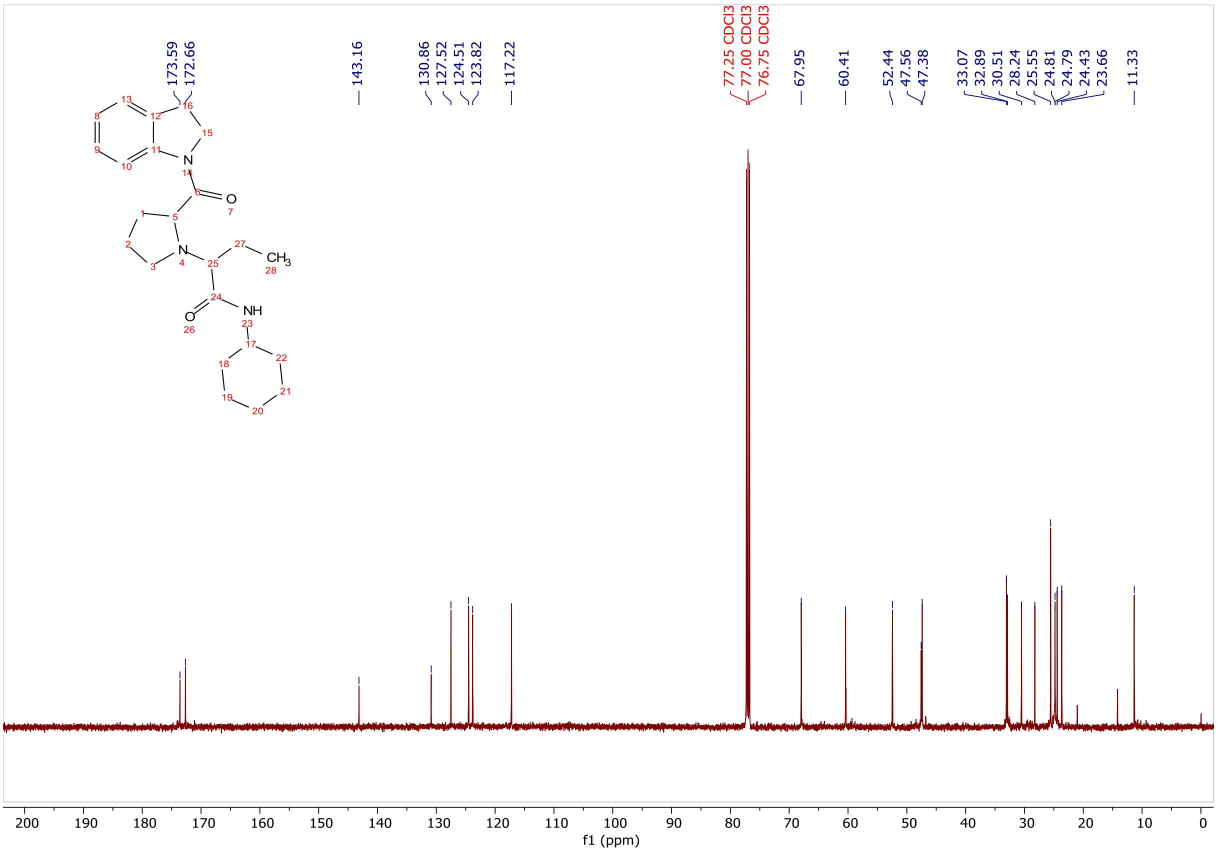

3-Isopropyl-2-(2-(thiophen-2-yl)acetyl)-2,3,6,7,12,12b-hexahydropyrazino [1',2':1,2]pyrido[3,4-b]indol-4(1H)-one (Destination plate IV, H2)

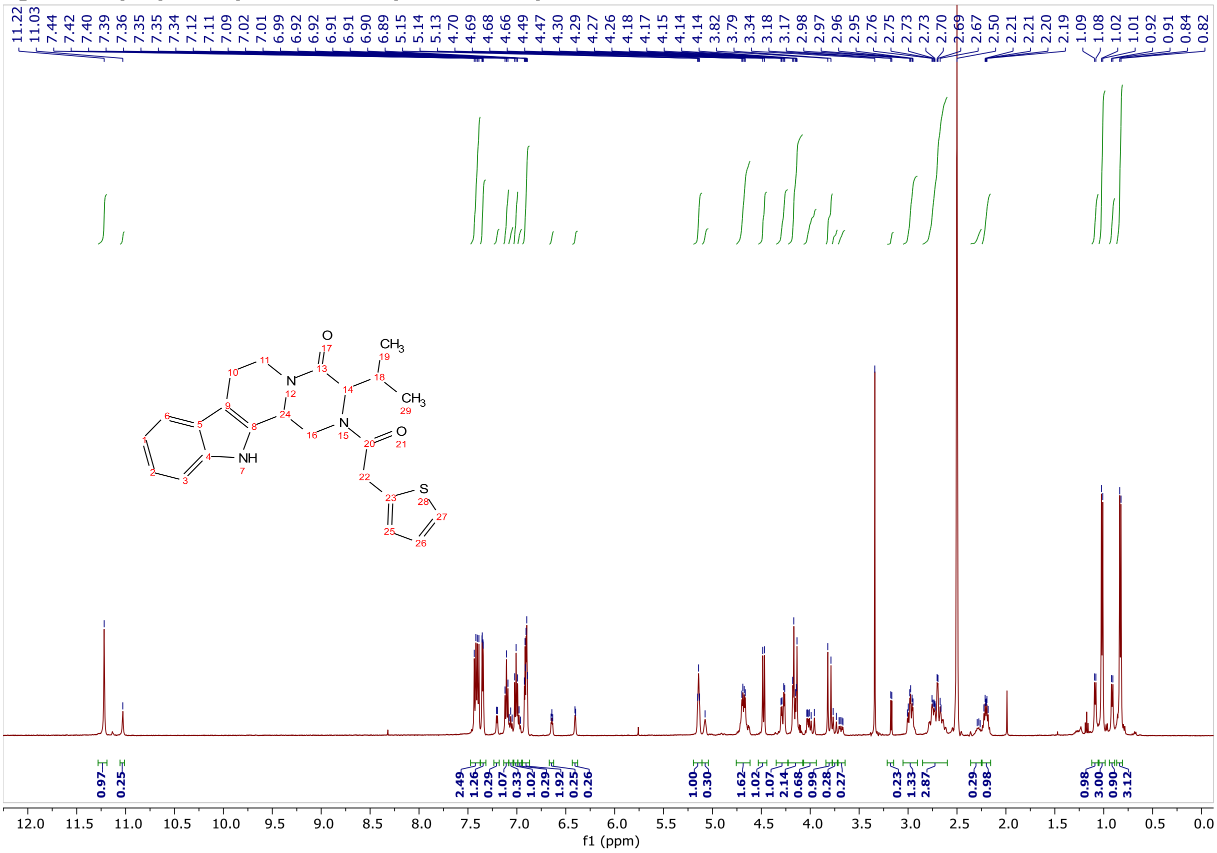

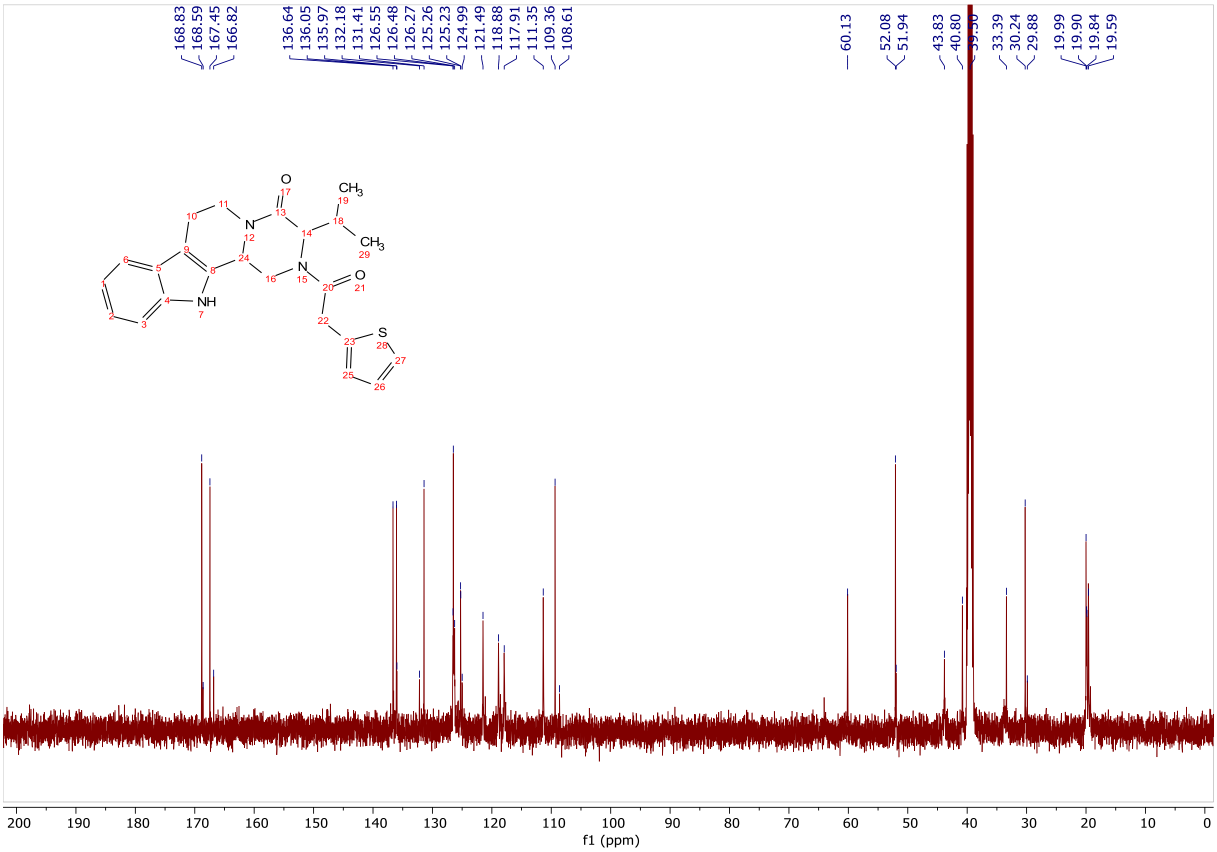

(E)-2-((2-(3-(2,3-Dimethoxyphenyl)-N-methylacrylamido)ethyl)(methyl)amino)-N-(thiophen-2-ylmethyl)pentanamide (Destination plate IV, J10)

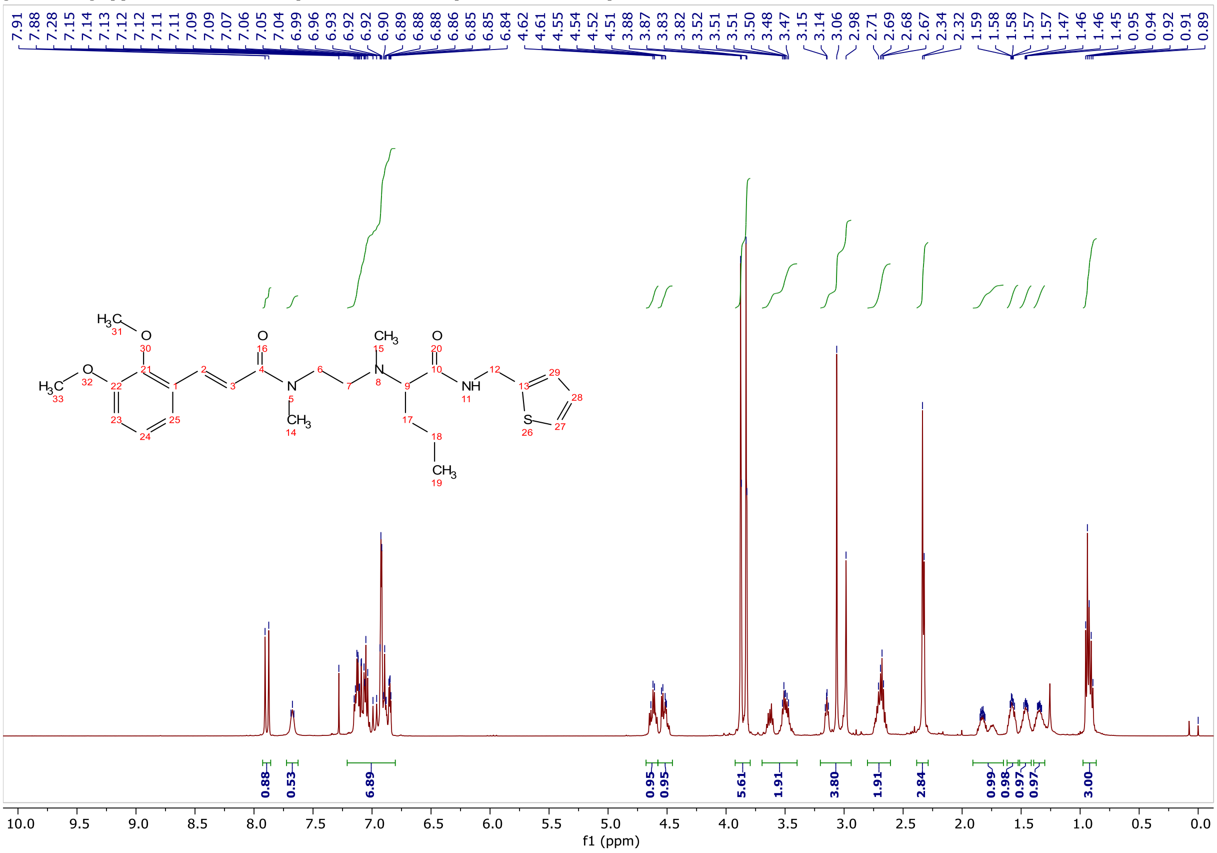

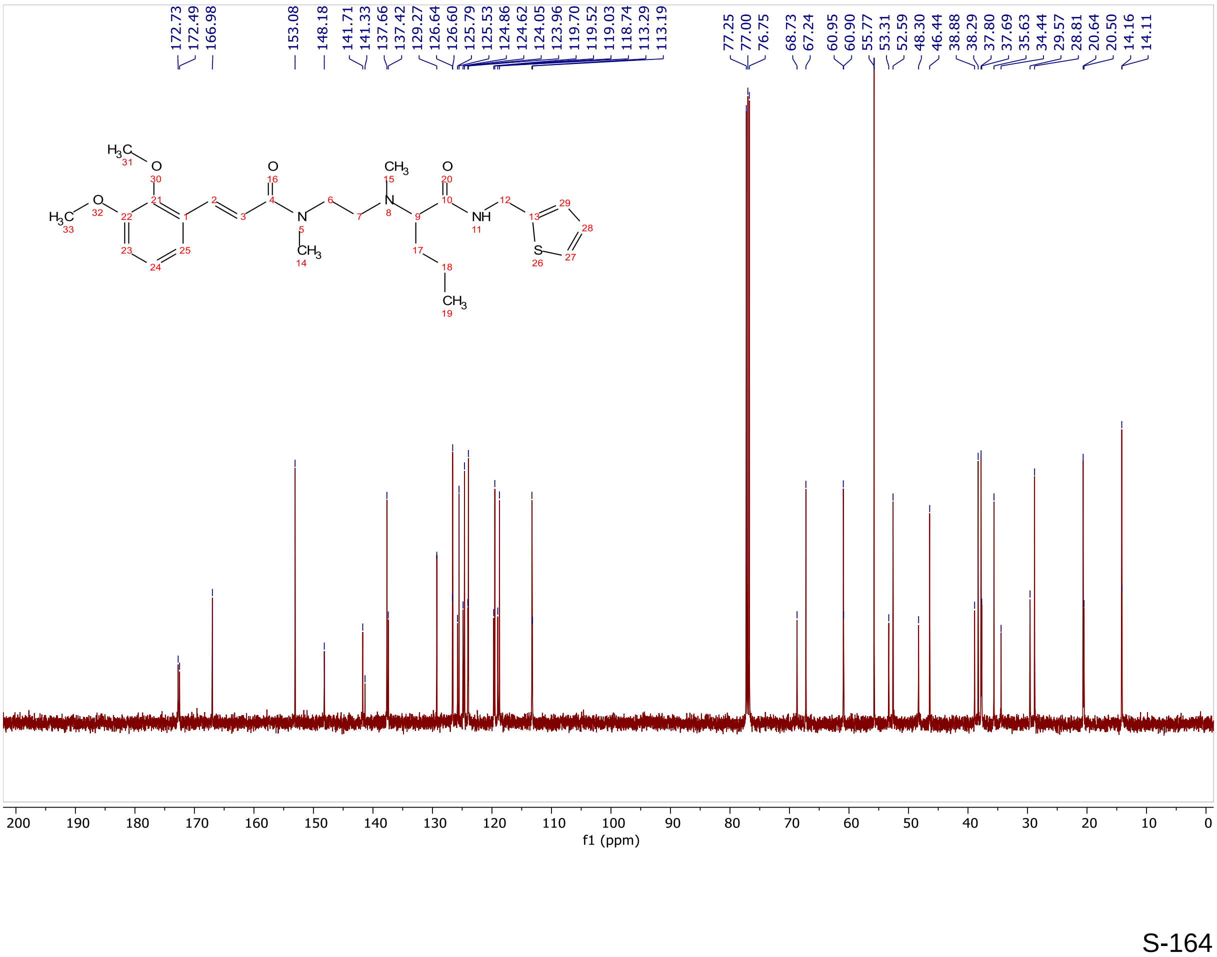

5. Computational chemistry

5.1. Dataset preparation

For our chemoinformatic analysis, we used 1536 SMILES strings as input format. The corresponding 2D coordinates were initially predicted with OpenBabel (v2.4.0)[26] and then converted to 3D with Mol3d (http://www.moloc.ch/). As a comparison dataset we fetched 1993 FDA-approved molecules from e-Drug3D.[27] Each molecule of this dataset consists in one 3D conformer calculated with CORINA (Molecular Networks GmbH). The most probable tautomeric form and ionic state at pH 7.4 were predicted with Filter and QUACPAC/Tautomers from OpenEye Scientific Software Inc. During the preparation of this dataset, we excluded 48 high-molecular weight polypeptides including four neuromuscular blocking agents (mivacurium, doxacurium, demecarium, cisatracurium), resulting in a final dataset of 1941 molecules. The list of the excluded molecules can be found in the supporting information (excluded.csv).

5.2. Molecular descriptors

Nano-scale library plates I-IV and FDA datasets were merged as a unique .sdf file format. Each entry was then parsed as a RDKIT objectsanitized for the calculation of 2D (e.g., MW, NHA, NHD, LogP and NumRotBonds) and 3D properties (TPSA and NPR1/2). Optimization of the 3D structure of the molecules was conducted with MMFF94s force field in MMFFOptimizeMolecule module in RDKIT (v2020.09.1, www.rdkit.org). The LogD calculation has been operated with Instant JChem, Chemaxon (v21.15.0). A comprehensive descriptive statistics of all 16 reactions and reference FDA datasets can be found in the supplementary files "statistics_echo.csv" and "statistics_FDA.csv".

5.3. Bioavailability filters

Lipinski, Ghose and Muegge profiles and CNS MPO score prediction were calculated for each molecule with InstantJChem (v21.4.0, ChemAxon (http://www.chemaxon.com). While Lipinski rule of 5 consider the pharmacokinetic aspects to be relevant for a drug like compound, the Muegge rule implements pharmacophore points to distinguish between drug-like and nondrug-like compounds. On the other hand, we have chosen the Ghose rule since the chemical and physical properties presented are suitable for the design of combinatorial libraries.[28] The commands are illustrated in **Table S5**. The summary of scaffolds with at least 50% of the molecules passing all three filters can be found in the file Summary_pc.xlsx.

5.4. Principal component analysis

Principal component analysis is a dimensional reduction technique that starting from a given dataset with high correlation between variables creates a new one with less correlation. In the new system of 2D axes created, the new variable with the highest variance is projected onto the first axis while the new variable, second in size in terms of variance, onto the second axis and so on. Complexity reduction is achieved by analyzing the main ones, by variance, among the new variables. Both PCA and physiochemical descriptor normalization were performed with "StandardScaler", embedded in Scikit learn (v0.22.2) which by definition standardizes features by removing the mean and scaling to unit variance. Finally, the new coordinates were plotted using two components with pyplot module in matplotlib (v3.4.2).

5.5. Normalized principal moments of inertia and cumulative distribution

NPR1/2 were calculated as described in "Molecular descriptors" paragraphs. Following a previous study by Morgan and coworkers,[29] the data were plotted in a triangular graph whose vertices determine a sphere (1.0, 1.0), a rod (0, 1.0), and a disc (0.5, 0.5), respectively. No Boltzmann average was applied. Subsequentially, the main triangle was again divided into four equilateral sub-triangles. Finally, using a python script written ad hoc, we counted the number of molecules falling in each specific area of each sub-triangle. The Euclidean distance of each dataset molecule from sphere, rod and disc was calculated using "linalg" module in numpy (v1.20.3). Finally, the cumulative distribution graphs were produced using the ecdfplot module in seaborn (v0.11.2).

5.6. Similarity analysis

For the similarity map, we compared the columns containing the Structure [Flexophore] fields, adopting a similarity limit of 85% and opting only to create the symmetric matrix. This method creates an additional column for each possible pariwise comparison with molecules having similar shape, size, flexibility and pharmacophore points corresponding in high Flexophore similarity. Next, molecules belonging to FDA and nano-scale library plates I-IV were binned separately and subsequentially compounds with similarity greater than 95% with at least one neighbor were connected with purple lines using the option “suppress/show connection lines” (April 2021 update) contained in the “Set Connection Line” display panel. Finally, the representation of chemical structures in the dendrograms was done with ChemDraw (v20.0, PerkinElmer Informatics).

5.7. t-distributed Stochastic Neighbor Embedding

t-distributed Stochastic Neighbor Embedding visualization of the chemical space was done with DataWarrior (v5.5.0, access date May 2021). By default, DataWarrior adopts a Barnes-Hut algorithm with a perplexity value of 20.0 and a maximum of 1000 iterations. SkeletonSpheres descriptors calculated from the Structure [SkelSpheres] field were provided for the two tSNE coordinates calculation.

5.8. Statistical Analysis

All cheminformatic statistical analysis between nano-scale library plates I-IV and FDA-approved molecules were performed with scikit-posthocs (v0.6.8), a scipy /statsmodels-based python package for parametric and nonparametric post hoc tests and plotting options.[30] A preliminary Kruskal-Wallis analysis of variance per each group was performed with stats module in scipy (v1.6.3) (**Table S8**), followed by Conover's test pairwise comparisons with holm p-values adjustment. On the other hand, for the rod, disc, and sphere distributions, a nonparametric two-sample, two-sided, nonparametric Kolmogorov-Smirnov test was run with stats module in scipy (v1.6.3).

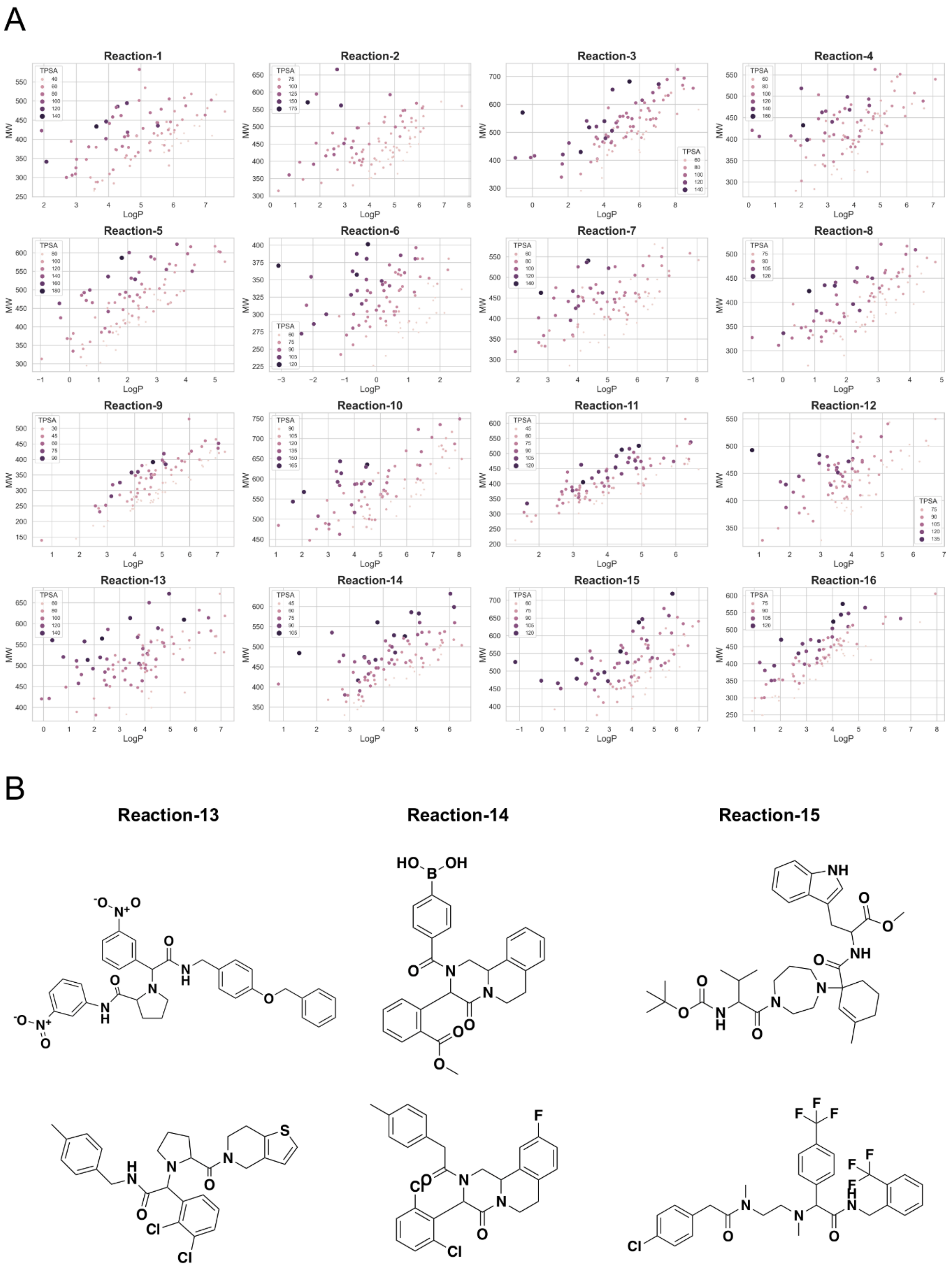

Fig. S7. (**A**) Plots of LogP vs MW for the nano-scale library plates I-IV. Data points were scaled and coloured from the most polar compound (dark purple) to the least polar compound (pink); (**B**) Exemplified outliers spotted in reactions 13,14 and 15 with lowest polarity and their chemical structures.

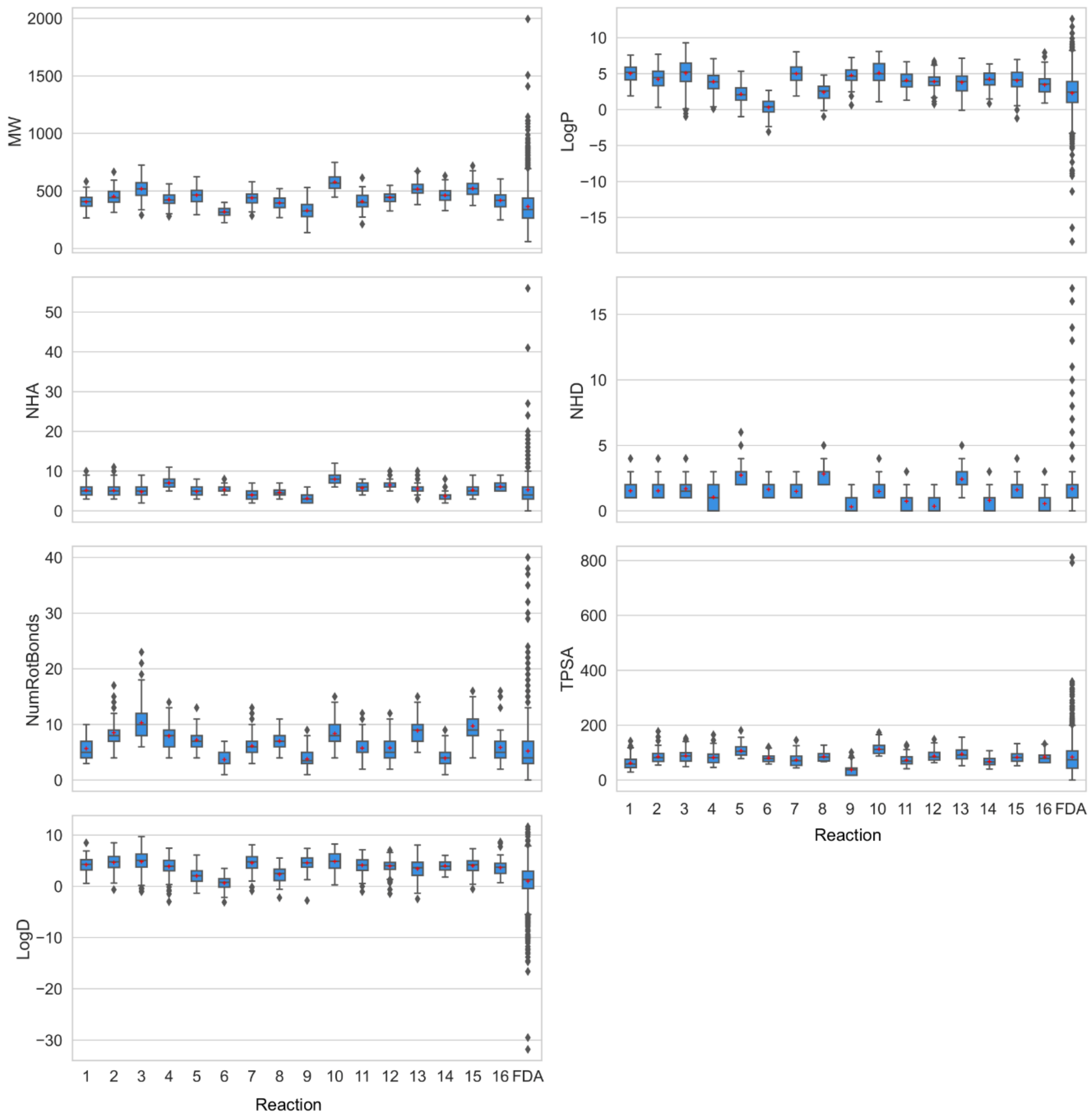

Fig. S8. Box plots distribution of seven physiochemical properties including molecular weight (MW), octanol/water logarithmic partition coefficient (LogP), octanol/water logarithmic partition coefficient at pH 7.4 (LogD), number of hydrogen bond donors (NHD), acceptors (NHA), number of rotable bonds (NumRotBonds) and topological polar surface area (TPSA). Median is indicated as a black line while the mean as a red cross.

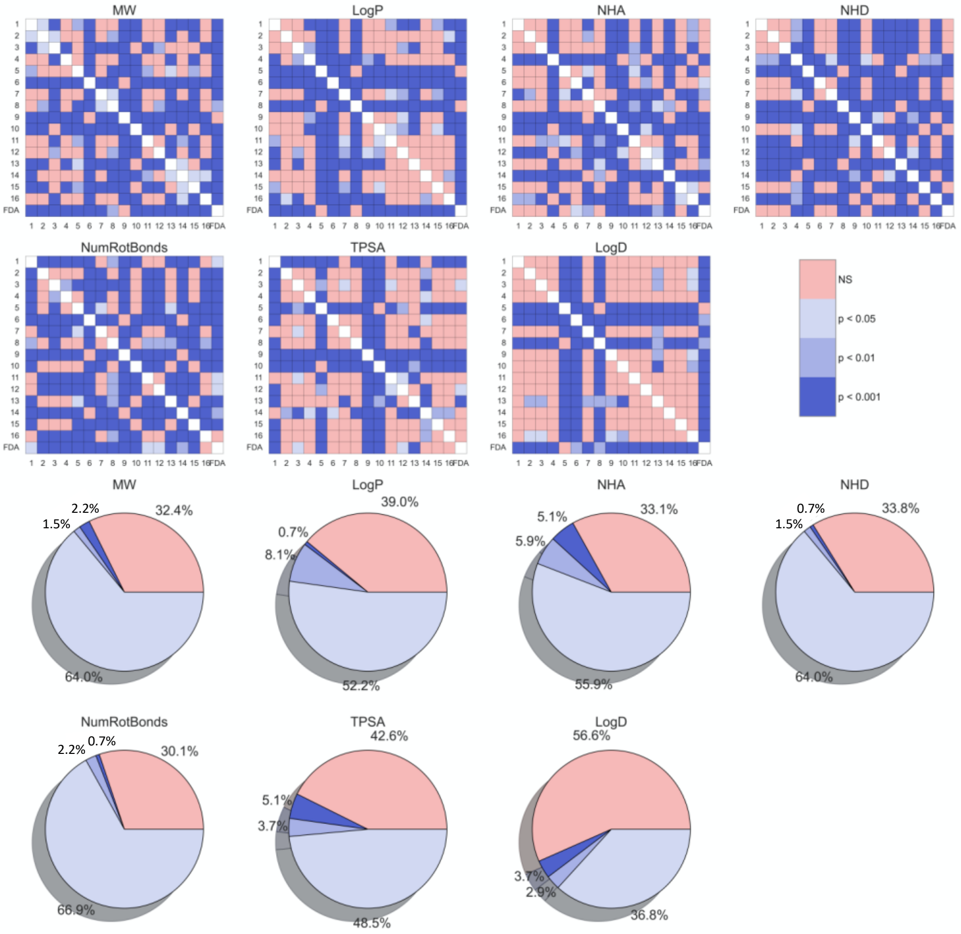

Fig. S9. (**A**) Kruskal-Wallis significance matrices for each possible pairwise combination. Depending on the p-value, the colour ranges from pink (not significant) to shades of purple; (**B**) pie chart representation showing the frequency of pairwise combinations within a specific p-value threshold.

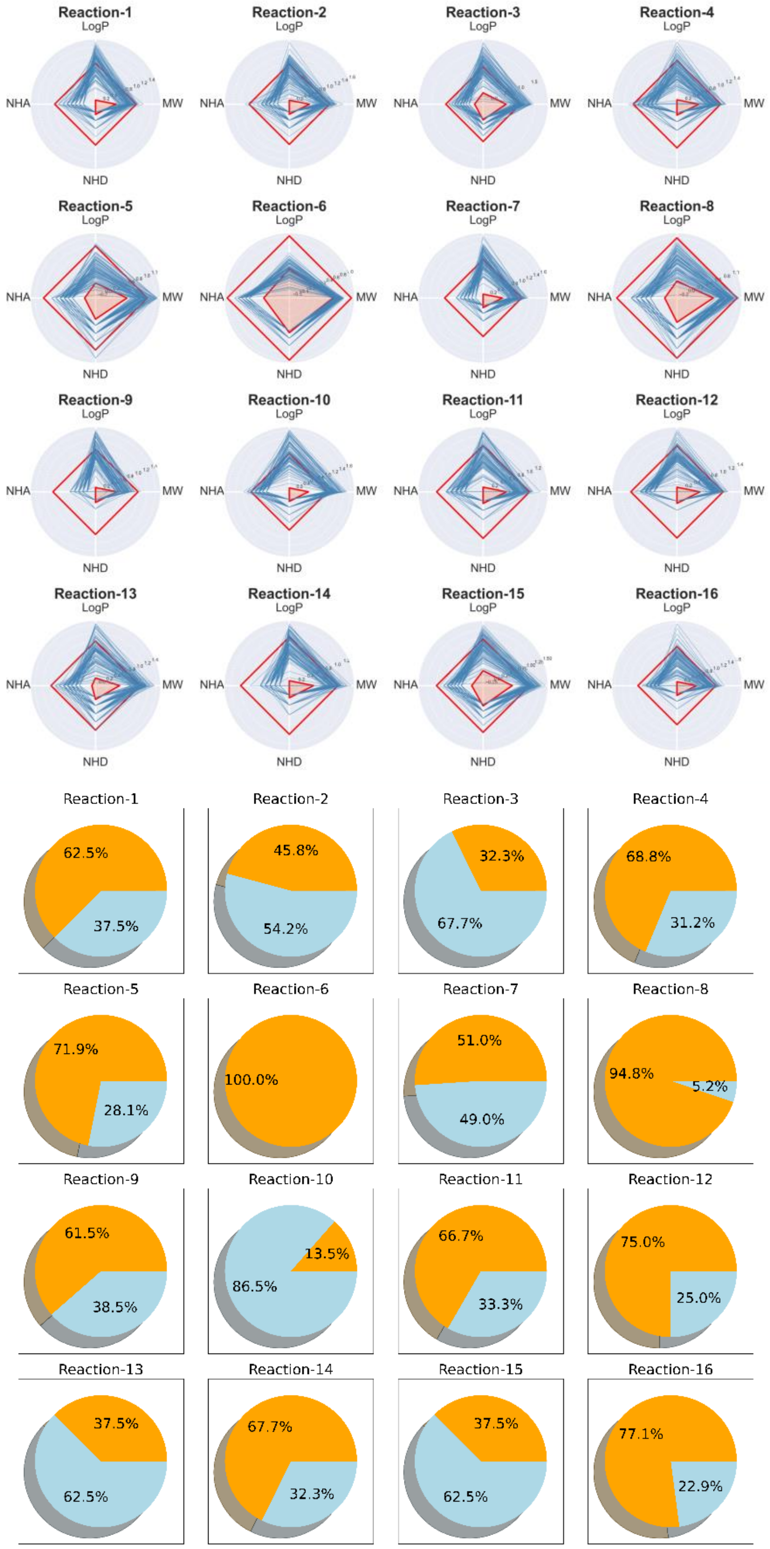

Fig. S10. Lipinski's rule of five (Ro5) analysis. (**A**) Spider plots of molecular weight (MW), number of hydrogen bond donors (NHD), acceptors (NHA) and logarithmic partition coefficient (LogP). The blue line represents each molecule with the density indicating the frequency while the limit of acceptability is represented by a red line. (**B**) Pie charts showing the fraction of compounds passing the Lipinski rule (light blue) and those that do not (orange).

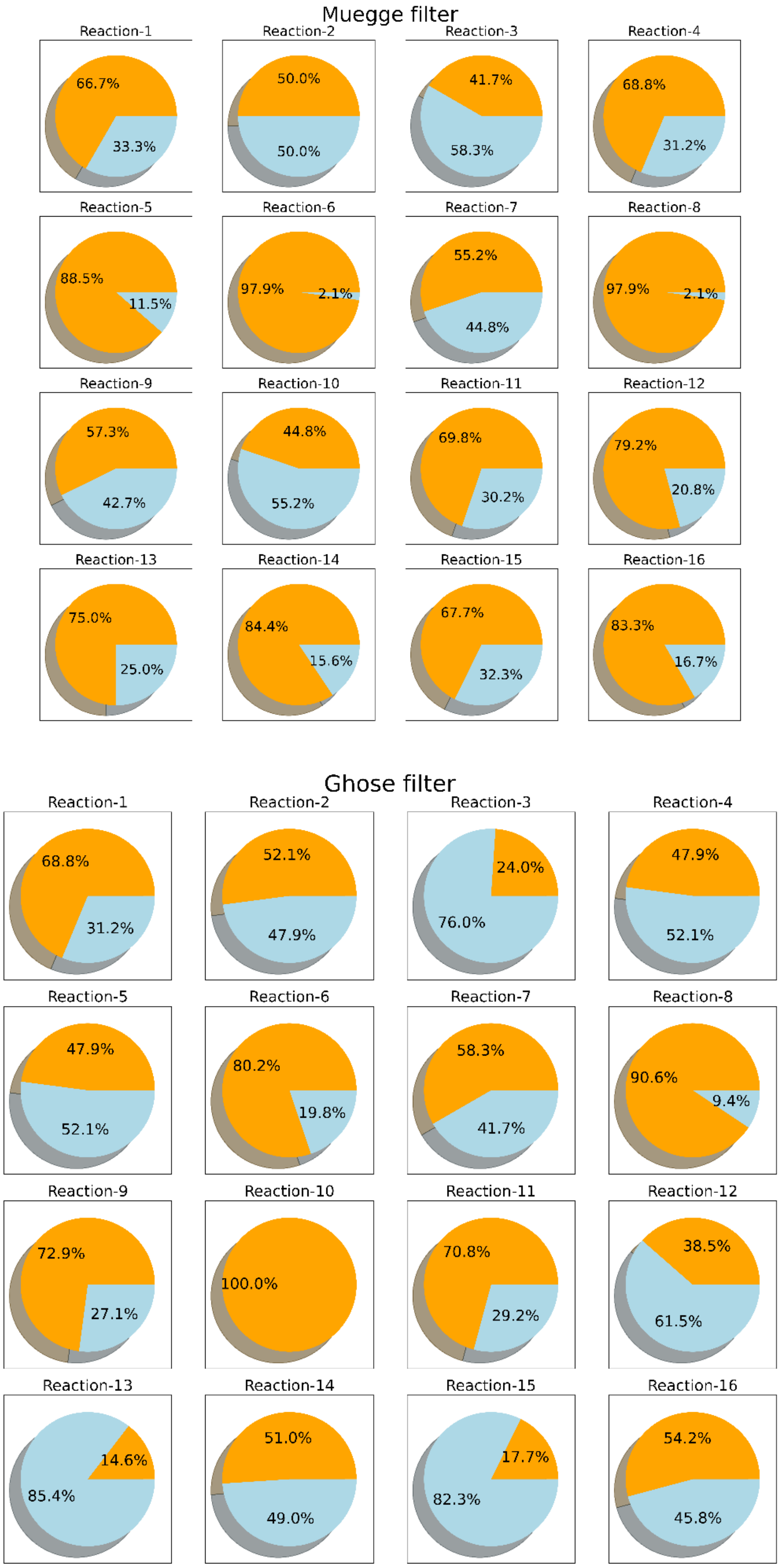

Fig. S11. Nano-scale plates I-IV bioavailability analysis with (**A**) Muegge and (**B**) Ghose filters. The colour description is the same as Fig. S8B.

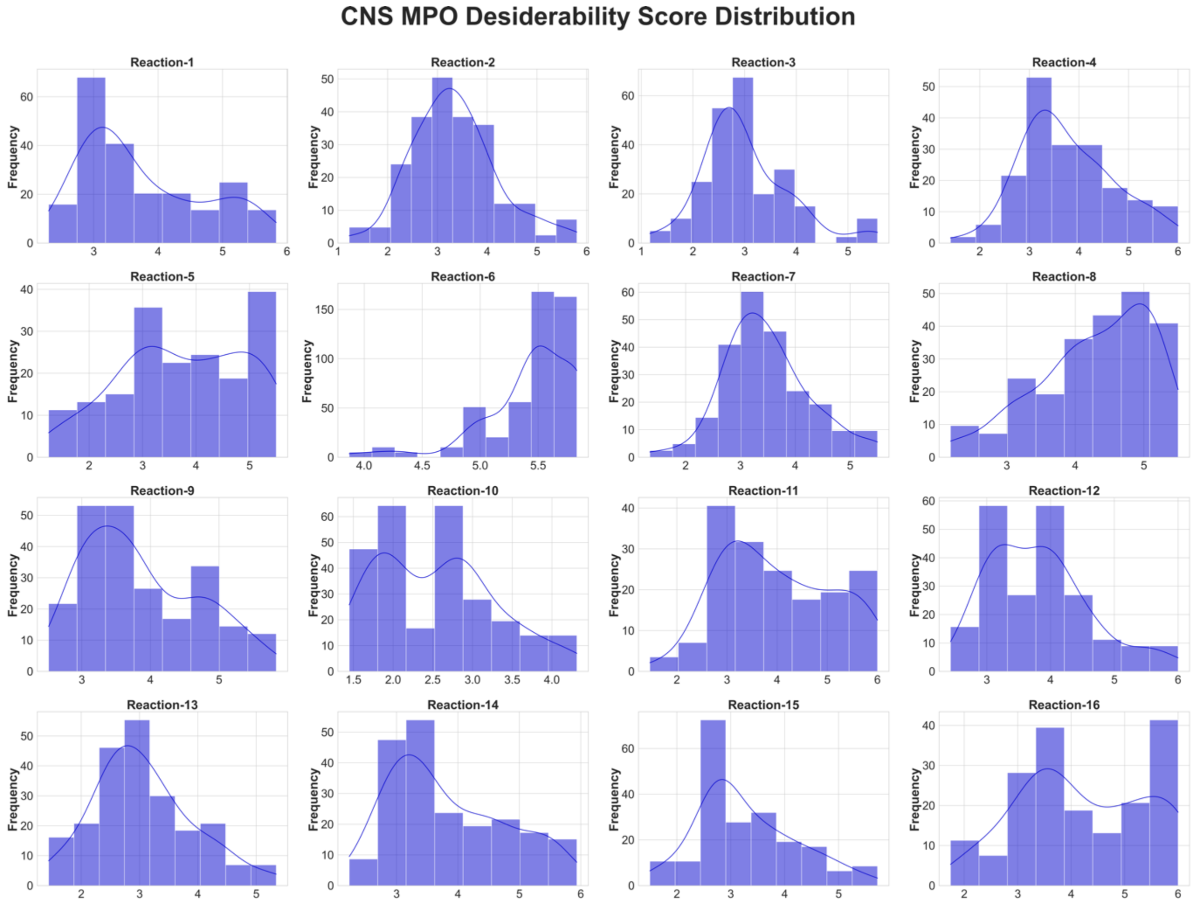

Fig. S12. Histograms of CNS MPO desirability score across the sixteen reactions. Kernel density estimation smoothing the distribution is displayed as a blue line. Y-axis (frequency) represents the number of observations divided by the bin width.

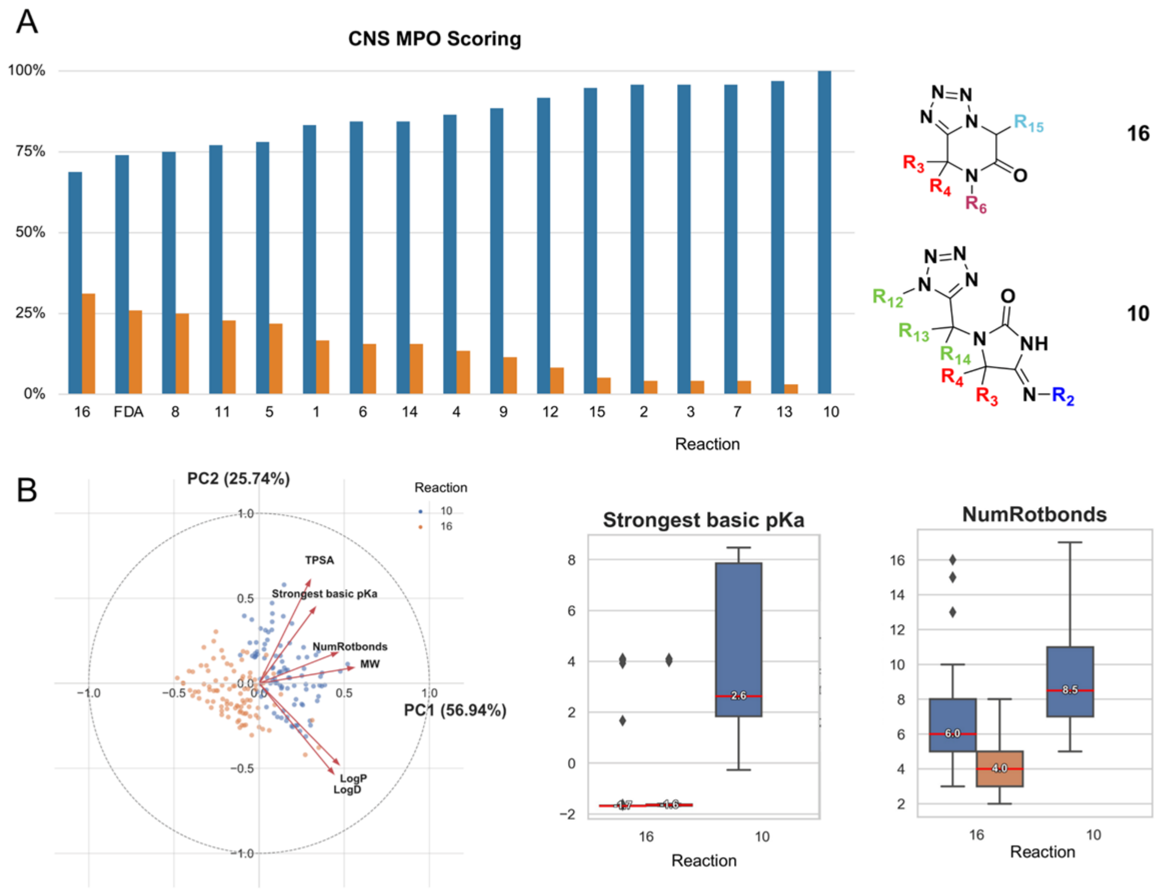

Fig. S13. CNS MPO scoring analysis. (**A**) Bar plot of the nano-scale library and FDA reference group ordered from highest to lowest score (orange bar, left to right). The blue bars indicate the percentage fraction of compounds with CNS MPO < 2. Framed to the side, the chemical structure of the top best (16) and worst scored (10) scaffold; (**B**) Principal component analysis plots based on the six physiochemical properties defining the CNS MPO score and box pots distributions of basicity and molecular flexibility quantified by the strongest basic pKa and number of rotatable bonds. The percent contribution of the two components is indicated on each axis while the eigenvalues are represented as red arrows. Median is indicated as a red line while outliers as black dots.

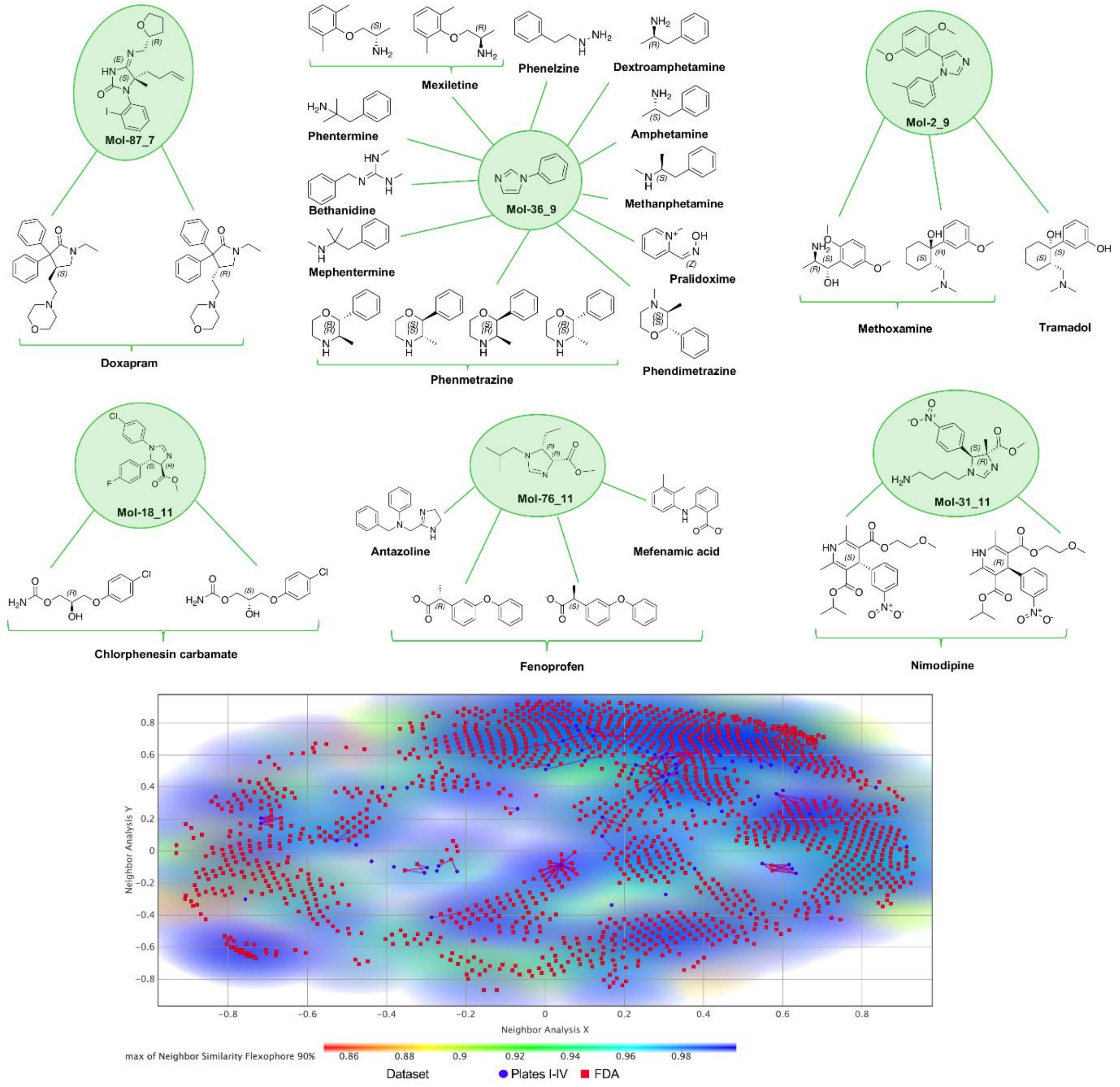

Fig. S14. Similarity analysis of the nano-scale plates I-IV. Upper part, lists some examples of similar molecules extracted from the similarity map. An underscore separates the compound identifier from the reaction number as shown in Table 1. Bottom part presents the similarity map employing Flexophore descriptor in DataWarrior. A purple line connects the molecules from plates I-IV (blue circles) with at least one neighbor (> 95% Flexophore similarity) of FDA drugs (red squares).

Table S1. PCA Eigenvectors.

	PC1	PC2	PC3	PC4	PC5	PC6
**Eigenvalue**	3.416	1.544	0.623	0.350	0.064	0.003
**Variability (%)**	56.940	25.735	10.381	5.830	1.067	0.047
**Cumulative %**	56.940	82.675	93.057	98.886	99.953	100.000

Table S2. PCA factor loadings.

	PC1	PC2	PC3	PC4	PC5
**MW**	0.963	0.108	0.047	-0.127	0.206
**LogP**	0.820	-0.562	0.069	-0.057	-0.044
**NumRotbonds**	0.791	0.210	-0.355	0.451	-0.015
**LogD**	0.767	-0.627	-0.015	-0.101	-0.086
**Strongest basic pKa**	0.570	0.519	0.625	0.107	-0.066
**TPSA**	0.285	0.575	-0.399	-0.548	-0.350

Table S3. PCA Contribution of the variables (%).

	PC1	PC2	PC3	PC4	PC5
**MW**	27.152	0.754	0.361	4.628	66.059
**LogP**	19.689	20.451	0.762	0.943	3.058
**NumRotbonds**	18.326	2.844	20.254	58.205	0.349
**LogD**	17.221	25.431	0.034	2.921	11.460
**Strongest basic pKa**	9.508	17.441	62.637	3.246	6.798
**TPSA**	8	33.079	15.952	30.057	12.276

Table S4. Pharmacophoric similarity matrix. Data below the average are indicated in red.

	1	2	3	4	5	6	7	8	9	10	11	12	13	14	15	16	FDA
**1**	0.58	0.34	0.32	0.43	0.2	0.27	0.38	0.38	0.5	0.36	0.45	0.29	0.39	0.31	0.34	0.3	0.31
**2**	0.34	0.56	0.49	0.33	0.36	0.3	0.41	0.42	0.26	0.39	0.41	0.4	0.47	0.45	0.45	0.38	0.27
**3**	0.32	0.49	0.55	0.31	0.43	0.3	0.47	0.44	0.25	0.45	0.3	0.43	0.54	0.51	0.48	0.38	0.26
**4**	0.43	0.33	0.31	0.55	0.18	0.2	0.33	0.34	0.38	0.41	0.39	0.47	0.35	0.28	0.35	0.43	0.27
**5**	0.2	0.36	0.43	0.18	0.72	0.23	0.39	0.41	0.14	0.36	0.17	0.39	0.47	0.58	0.45	0.28	0.21
**6**	0.27	0.3	0.3	0.2	0.23	0.78	0.29	0.33	0.22	0.25	0.23	0.21	0.35	0.3	0.3	0.22	0.25
**7**	0.38	0.41	0.47	0.33	0.39	0.29	0.59	0.46	0.38	0.54	0.37	0.5	0.47	0.48	0.4	0.37	0.3
**8**	0.38	0.42	0.44	0.34	0.41	0.33	0.46	0.55	0.37	0.41	0.37	0.4	0.5	0.46	0.41	0.35	0.3
**9**	0.5	0.26	0.25	0.38	0.14	0.22	0.38	0.37	0.57	0.34	0.45	0.22	0.34	0.26	0.31	0.21	0.31
**10**	0.36	0.39	0.45	0.41	0.36	0.25	0.54	0.41	0.34	0.65	0.33	0.57	0.45	0.44	0.4	0.49	0.28
**11**	0.45	0.41	0.3	0.39	0.17	0.23	0.37	0.37	0.45	0.33	0.6	0.24	0.35	0.26	0.31	0.24	0.3
**12**	0.29	0.4	0.43	0.47	0.39	0.21	0.5	0.4	0.22	0.57	0.24	0.74	0.42	0.49	0.37	0.53	0.22
**13**	0.39	0.47	0.54	0.35	0.47	0.35	0.47	0.5	0.34	0.45	0.35	0.42	0.62	0.52	0.52	0.37	0.3
**14**	0.31	0.45	0.51	0.28	0.58	0.3	0.48	0.46	0.26	0.44	0.26	0.49	0.52	0.74	0.47	0.38	0.27
**15**	0.34	0.45	0.48	0.35	0.45	0.3	0.4	0.41	0.31	0.4	0.31	0.37	0.52	0.47	0.56	0.36	0.27
**16**	0.3	0.38	0.38	0.43	0.28	0.22	0.37	0.35	0.21	0.49	0.24	0.53	0.37	0.38	0.36	0.72	0.22
**FDA**	0.31	0.27	0.26	0.27	0.21	0.25	0.3	0.3	0.31	0.28	0.3	0.22	0.3	0.27	0.27	0.22	0.24

Table S5. Pharmacokinetic filters from InstantJChem and the expression used to call them.

Description	Filter expression
**Lipinski rule of 5**	mass()<=500<br>logP()<=5<br>donorCount()<=5<br>acceptorCount()<=10
**Ghose rule**	logP()>=-0.4<br>logP()<=5.6<br>mass()>=160<br>mass()<=480<br>atomCount()>=20<br>atomCount()<=70<br>refractivity()>=40<br>refractivity()<=130
**Muegge rule**	mass >= 200 and mass <= 600 and<br>ringCount <= 7 and<br>atomCount('6') >= 5 and (atomCount - atomCount('6') - atomCount('1')) >= 2 and<br>(rotatableBondCount <= 15) and<br>donorCount <= 5 and<br>acceptorCount <= 10 and<br>logP >= -2 and logP <= 5 and<br>PSA <= 150
**CNS MPO**	logPvar = logP;<br>logDvar = logD("7.4");<br>massvar = mass;<br>psavar = psa;<br>donorCountvar = donorCount;<br>basicpKavar = basicpKa("1");<br><br>(logPvar <= 3) + (logPvar > 3 AND logPvar < 5) * (2.5 - logPvar / 2) +<br>(logDvar <= 2) + (logDvar > 2 AND logDvar < 4) * (2 - logDvar / 2) +<br>(massvar <= 360) + (massvar > 360 AND massvar < 500) * (500 / 140 - massvar / 140) +<br>(psavar > 40 AND psavar <= 90) + (psavar > 90 AND psavar < 120) * (4 - psavar / 30) +<br>(psavar > 20 AND psavar <= 40) * (psavar / 20 - 1) +<br>(donorCountvar <= 0.5) + (donorCountvar > 0.5 AND donorCountvar < 3.5) * (3.5 / 3 - donorCountvar / 3) +<br>(basicpKavar <= 8) + (basicpKavar > 8 AND basicpKavar < 10) * (5 - basicpKavar / 2)

Table S6. Molecules count into the shapes defined by the four sub-triangle areas.

Library	Rod	Hybrid	Disc	Sphere	Total
**Nano-scale synthesis plates I-IV**	624	515	373	24	1536
**FDA**	1255	304	361	21	1941

Table S7. Summary table of the Kolmogorov-Smirnov statistical analysis.

**Kolmogorov-Smirnov test**	
**Nano-scale synthesis plates I-IV / FDA**	
**Shape**	**p-values**
**Rod**	< 0.001
**Disc**	< 0.001
**Sphere**	< 0.001
**Hybrid**	< 0.001

References

1. Patil, P.; Ahmadian-Moghaddam, M.; Dömling, A., Isocyanide 2.0. *Green Chem*. **2020**, (22), 6902-6911.

2. Skorna, G.; Ugi, I., Isocyanide synthesis with diphosgene. *Angew. Chem. Int. Ed.* **1977**, 16 (4), 259-260.

3. Ugi, I.; Fetzer, U.; Eholzer, U.; Knupfer, H.; Offermann, K., Isonitrile syntheses. *Angew. Chem. Int. Ed.* **1965**, 4 (6), 472-484.

4. Ugi, I.; Meyr, R., o-Tolyl Isocyanide. *Org. Synth*. **1961**, 41, 101.

5. Gokel, G. W.; Widera, R. P.; Weber, W. P., Phase-Transfer Hofmann Carbylamine Reaction: tert-Butyl Isocyanide: Propane, 2-isocyano-2-methyl-. *Org. Synth*. **2003**, 55, 96-96.

6. Weber, W. P.; Gokel, G. W.; Ugi, I. K., Phase transfer catalysis in the Hofmann carbylamines reaction. *Angew. Chem. Int. Ed*. *Engl*. **1972**, 11 (6), 530-531.

7. Neochoritis, C. G.; Zarganes-Tzitzikas, T.; Stotani, S.; Dömling, A.; Herdtweck, E.; Khoury, K.; Dömling, A., Leuckart–Wallach route toward isocyanides and some applications. *ACS. Comb. Sci*. **2015**, 17 (9), 493-499.

8. Boltjes, A.; Dömling, A., The Groebke-Blackburn-Bienaymé Reaction. *Eur. J. Org. Chem.* **2019,** *2019* (42), 7007-7049.

9. Banfi, L.; Riva, R. J. O. r., The P asserini Reaction. *Org. React.* **2004,** *65*, 1-140.

10. Ugi, I. J. A. C. I. E. i. E., The α-addition of immonium ions and anions to isonitriles accompanied by secondary reactions. *Angew. Chem. Int. Ed.* **1962,** *1* (1), 8-21.

11. Patil, P.; Zhang, J.; Kurpiewska, K.; Kalinowska-Tłuścik, J.; Dömling, A. J. S., Hydrazine in the Ugi tetrazole reaction. *Synthesis.* **2016,** *48* (08), 1122-1130.

12. Cioc, R. C.; van Riempst, L. S.; Schuckman, P.; Ruijter, E.; Orru, R. V. J. S., Ugi four-center three-component reaction as a direct approach to racetams. *Synthesis.* **2017,** *49* (07), 1664-1674.

13. Srivastava, S.; Beck, B.; Herdtweck, E.; Khoury, K.; Doemling, A. J. H., A novel deltathiolactone scaffold by a versatile intramolecular multicomponent reaction. *Heterocycles.* **2009,** *77* (2), 731-738.

14. Shaabani, S.; Xu, R.; Ahmadianmoghaddam, M.; Gao, L.; Stahorsky, M.; Olechno, J.; Ellson, R.; Kossenjans, M.; Helan, V.; Dömling, A. J. G. C., Automated and accelerated synthesis of indole derivatives on a nano-scale. *Green Chem.* **2019,** *21* (2), 225-232.

15. Park, S. J.; Keum, G.; Kang, S. B.; Koh, H. Y.; Kim, Y.; Lee, D. H. J. T. l., A facile synthesis of N-carbamoylmethyl-α-aminobutyrolactones by the Ugi multicomponent condensation reaction. *Tetrahedron Lett.* **1998,** *39* (39), 7109-7112.

16. Van Leusen, A. J. C. I., Synthetic Applications of Tosylmethyl Isocyanide and Derivatives. New Synthons in Organic Chemistry. **1980**.

17. Patil, P.; Mishra, B.; Sheombarsing, G.; Kurpiewska, K.; Kalinowska-Tłuścik, J.; Dömling, A. J. A. c. s., Library-to-library synthesis of highly substituted α-aminomethyl tetrazoles via Ugi reaction. *ACS Comb. Sci.* **2018,** *20* (2), 70-74.

18. Bon, R. S.; Hong, C.; Bouma, M. J.; Schmitz, R. F.; de Kanter, F. J.; Lutz, M.; Spek, A. L.; Orru, R. V. J. O. l., Novel multicomponent reaction for the combinatorial synthesis of 2-imidazolines. *Org. Lett.* **2003,** *5* (20), 3759-3762.

19. Marcos, C. F.; Marcaccini, S.; Menchi, G.; Pepino, R.; Torroba, T. J. T. L., Studies on isocyanides: synthesis of tetrazolyl-isoindolinones via tandem Ugi four-component condensation/intramolecular amidation. *Tetrahedron Lett.* **2008,** *49* (1), 149-152.

20. Khoury, K.; Sinha, M. K.; Nagashima, T.; Herdtweck, E.; Dömling, A. J. A. C., Efficient assembly of iminodicarboxamides by a "truly" four-component reaction. *Angew. Chem.* **2012,** *124* (41), 10426-10429.

21. Wang, W.; Ollio, S.; Herdtweck, E.; Dömling, A., Polycyclic Compounds by Ugi− Pictet− Spengler Sequence. *J. Org. Chem.* **2011,** *76* (2), 637-644.

22. Giovenzana, G. B.; Tron, G. C.; Di Paola, S.; Menegotto, I. G.; Pirali, T. J. A. C., A Mimicry of Primary Amines by Bis-Secondary Diamines as Components in the Ugi Four-Component Reaction. *Angew. Chem.* **2006,** *118* (7), 1117-1120.

23. Patil, P.; Kurpiewska, K.; Kalinowska-Tłuścik, J.; Dömling, A. J. A. c. s., Ammonia-promoted one-pot tetrazolopiperidinone synthesis by Ugi reaction. *ACS Comb. Sci.* **2017,** *19* (5), 343-350.

24. Huang, Y.; Wolf, S.; Bista, M.; Meireles, L.; Camacho, C.; Holak, T. A.; Dömling, A. J. C. b.; design, d., 1, 4-Thienodiazepine-2, 5-diones via MCR (I): Synthesis, Virtual Space and p53-Mdm2 Activity. *Chem. Biol. Drug. Des*. **2010**, 76 (2), 116-129.

25. Osipyan, A.; Shaabani, S.; Warmerdam, R.; Shishkina, S. V.; Boltz, H.;, Automated, accelerated nanoscale synthesis of iminopyrrolidines. *Angew. Chem. Int. Ed*. **2020**, 59, 12423-12427.

26. Csizmadia, F., JChem: Java applets and modules supporting chemical database handling from web browsers. J. Chem. Inform. Comput. Sci. 2000, 40 (2), 323-324.

27. Pihan, E., Colliandre, L., Guichou, J. F., & Douguet, D.. e-Drug3D: 3D structure collections dedicated to drug repurposing and fragment-based drug design. *Bioinformatics*, **2012**, *28*(11), 1540-1541.

28. AK, G., Viswanadhan VN. Wendoloski JJ. A knowledgebased approach in designing combinatorial or medicinal chemistry libraries for drug discovery: I, A qualitative and quantitative characlerization of known drug databases. *J. Comb. Chem.* **1999**, 55-68.

29. Morgan, B. S.; Forte, J. E.; Culver, R. N.; Zhang, Y.; Hargrove, A. E., Discovery of key physicochemical, structural, and spatial properties of RNA-targeted bioactive ligands. *Angew. Chem.* **2017,** *129* (43), 13683-13687.

30. Terpilowski, M. A., scikit-posthocs: pairwise multiple comparison tests in Python. *J. Open Res. Softw.* **2019,** *4* (36), 1169.
